# Supplementary material for: Peptide-guided functionalization and macrocyclization of bioactive peptidosulfonamides by Pd(II)-catalyzed late-stage C–H activation
Source: Nat Commun. 2018 Aug 23;9:3383. doi: 10.1038/s41467-018-05440-w (PMC6107497; doi:10.1038/s41467-018-05440-w)
Supplement: Supplementary file 1 — Supplementary Information [file 41467_2018_5440_MOESM1_ESM.pdf]

A)

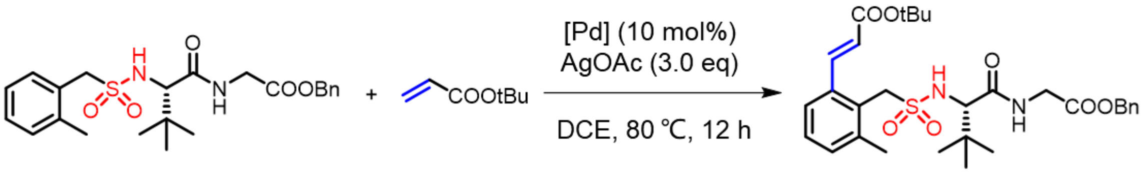

1a 2a 3aa

Entry	Catalyst	Oxidant	Time (h)	Yield (%)
1	$Pd(OAc)_2$	AgOAc	12	80
2	$PdCl_2$	AgOAc	12	trace
3	$Pd(PPh_3)_2Cl_2$	AgOAc	12	72
4	$PdCl_2$pddf	AgOAc	12	62
5	$Pd(TFA)_2$	AgOAc	12	68
6	$Pd(NO_3)_2$	AgOAc	12	trace
7	$Pd(acac)_2$	AgOAc	12	trace

B)

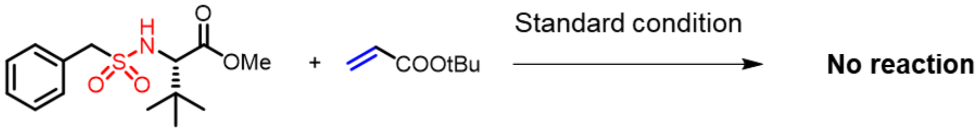

1a’

Supplementary Table 1. Optimization of reaction conditions for *ortho*-methylbenzylsulfonamide dipeptide conjugate **1a** and *tert*-butyl acrylate **2a.** Reaction conditions: **1a** (0.1 mmol), **2a** (0.4 mmol), Pd catalyst (0.01 mmol), AgOAc (0.4 mmol), DCE (2.0 ml), 80°C, 12 h.

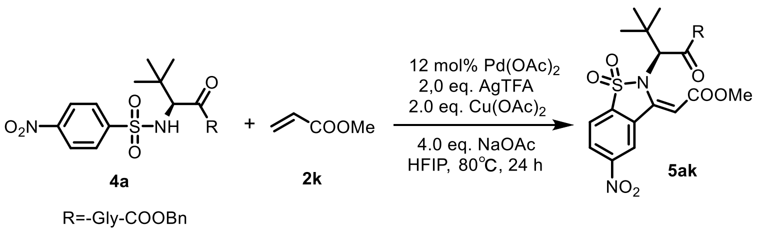

Entry	Changes from standard conditions	Conv[%]	Yield of **5ak**
1	-	95%	82%
2	$Pd(TFA)_2$	70%	62%
3	AgOAc	68%	65%
4	NaOPiv	65%	70%
5	Na_2CO_3	72%	72%
6	1,4-Benzoquinone	25%	12%
7	DCE	83%	68%
8	R=-OMe	<5%	-

Supplementary Table 2. Optimization of reaction conditions for substrate **4a** and methyl acrylate **2k.**

A)

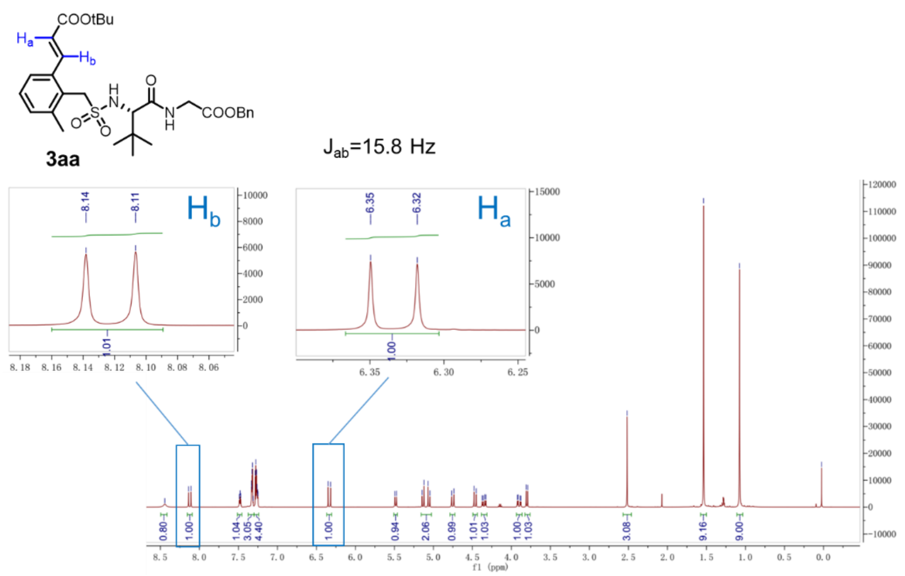

B)

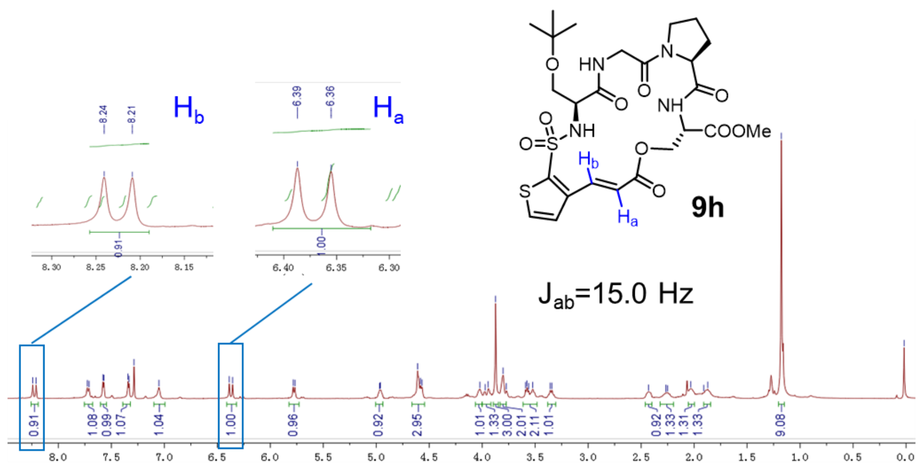

Supplementary Figure 1 A) Determination of the configuration of exocyclic double bond in product 3aa by 1H-NMR (400 MHz, CDCl3). The coupling constant was Jab=15.8 Hz, and therefore the double bond is in E-configuration. B) Determination of the configuration of exocyclic double bond in product **9h** by ^{1}H-NMR(400 MHz, $CDCl_3$). The coupling constant was J_{ab}=15.0 Hz, and therefore the double bond is in *E*-configuration. NMR spectra were recorded on Bruker AMX-400 instrument.

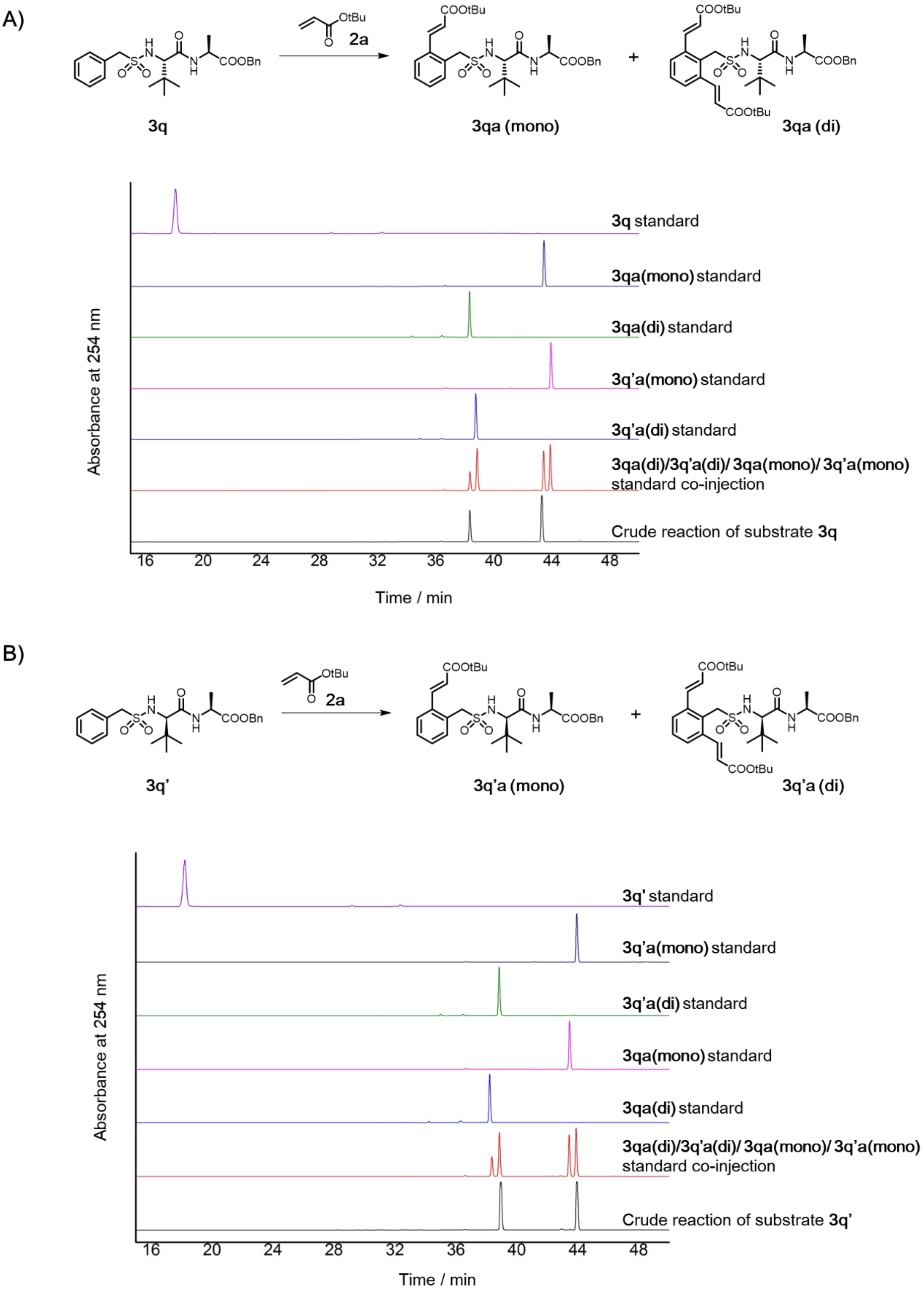

Supplementary Figure 2 Investigation of possible epimerization during reaction of substrates **3q** and **3q'**.A) substrates **3q** and **3q'** (0.10 mmol) were reacted with acrylate **2a** (0.40 mmol) in 2.0 ml DCE under standard conditions for 12 h. The reaction mixture were filtrated through a Celite pad, concentrated under reduced pressure and directly analyzed by RP-HPLC without further purification. B）HPLC analysis of crude reaction mixtures of **3q** and **3q'**. No epimerization was observed for either substrate. The NMR spectra of each compounds are provided in the supplementary figures 129-140.

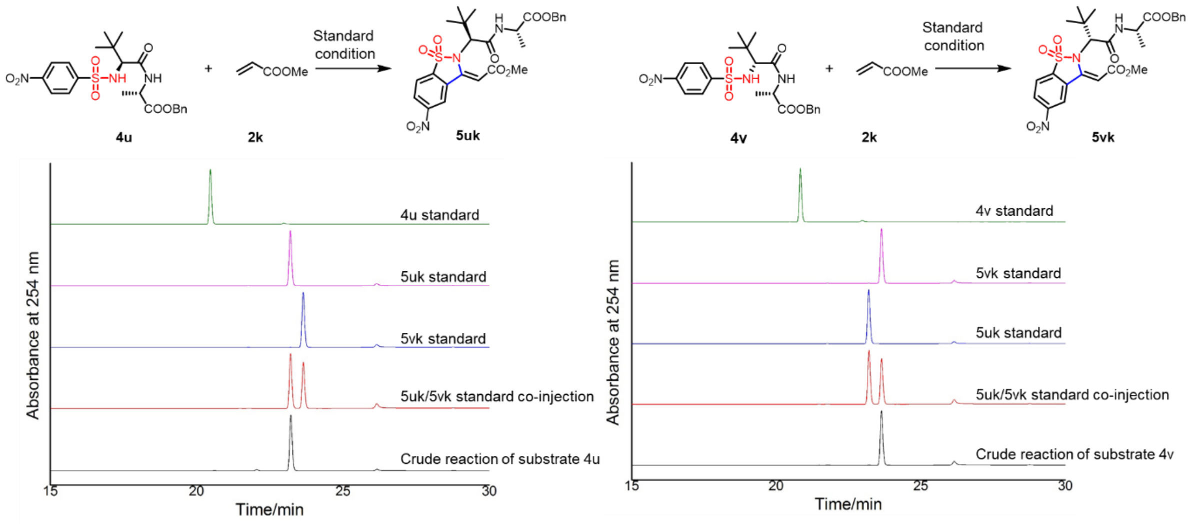

Supplementary Figure 3 Investigation of possible epimerization during reactions of substrate **4u** and **4v**. Substrates **4u** and **4v** (0.10 mmol) were reacted with methyl acrylate **2k** (0.40 mmol) in 2.0 ml HFIP under standard conditions for 24 h. The reaction mixture were filtrated through a Celite pad, concentrated under reduced pressure and directly analyzed by RP-HPLC without further purification. No epimerization was observed for either reaction. The NMR spectra of each compounds are provided in supplementary figures 237-242.

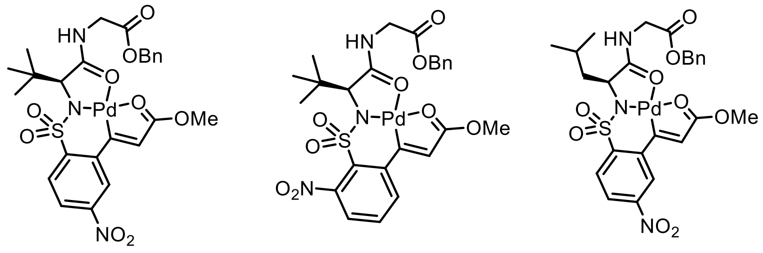

7ak 7jk 7ok

Supplementary Figure 4 Chemical structures of Pd complex **7ak**, **7jk** and **7ok**.

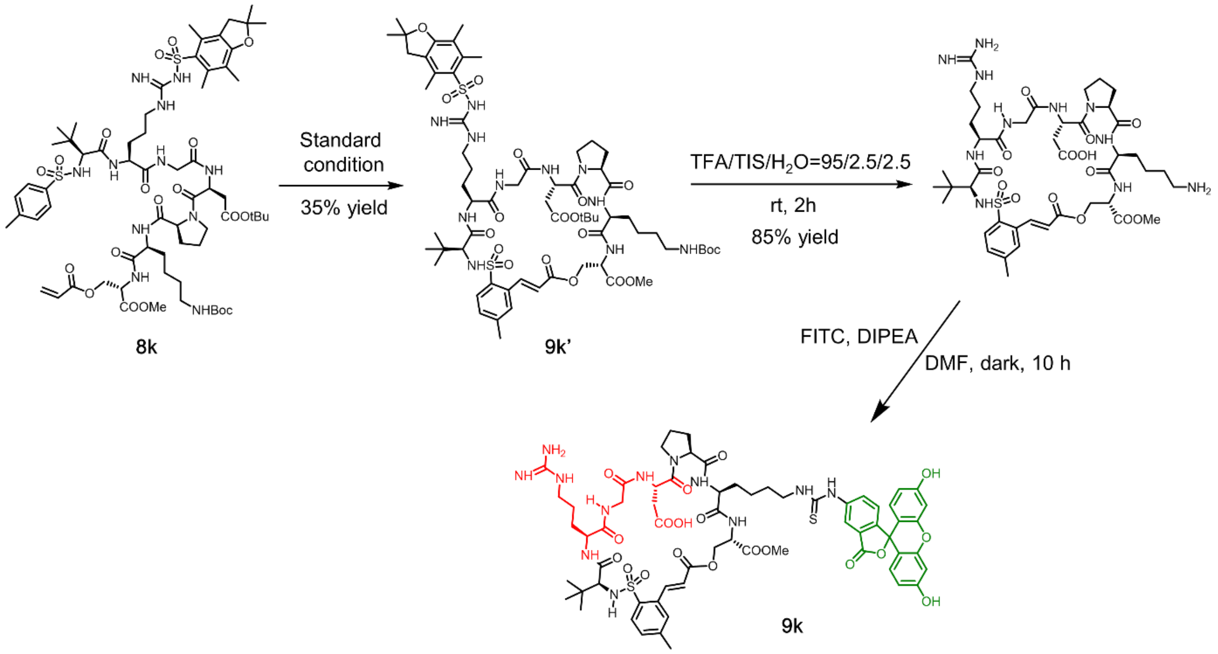

Supplementary Figure 5 Synthesis of FITC-labelled cyclic peptide **9k**. Linear peptide **8k** (2.0 mmol), $Pd(OAc)_2$ (0.2 mmol), AgOAc (4.0 mmol) and DCE (10 ml) was added to a 100 ml sealed reaction tube. The reaction mixture was heated and stirred at 80 ℃ for 24 h. Upon completion, the tube was cooled to room temperature and the reaction mixture was diluted by ethyl acetate (5 ml), filtered through a Celite pad. The filtrate was concentrated under reduced pressure. The resulting mixture was purified by column chromatography (ethyl acetate:methol= 8:1; R_f = 0.32) to afford cyclic peptide **9k'** a white solid in 35% yield. Cyclic peptide **9k'** was then deprotected using a cocktail of TFA/TIS/H_2O=95/2.5/2.5 at room temperature for 2 h. The crude product was concentrated and incubated with FITC(1.5 eq) and DIPEA(5.0 eq) in DMF at room temperature for 10 h under the atmosphere of N_2. Upon completion, the reaction mixture was purified by RP-HPLC to afford the FITC-labelled product **9k** as a yellow solid in 60% (two steps).

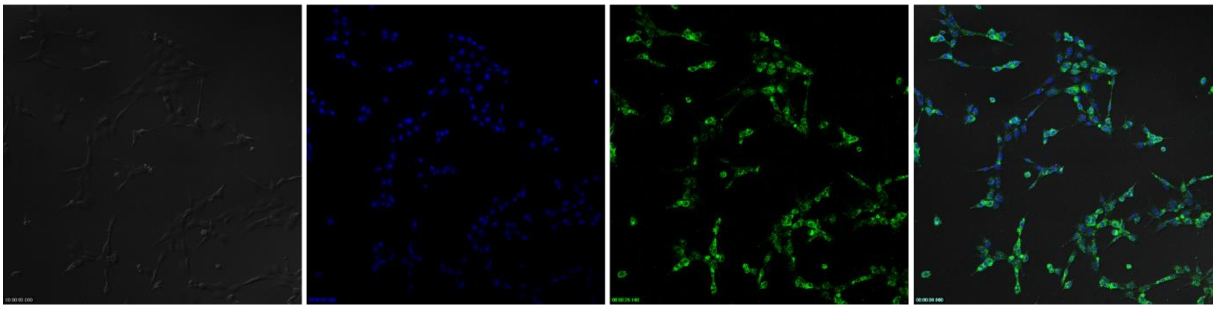

Supplementary Figure 6 FITC-labelled cyclic RGD peptide **9k** (2 μM) and its integrin binding assays analyzed by confocal microscopy. U87MG cell were grown and maintained in DMEM media with 10% FBS and 1% penicillin/streptomycin at 37 °C, 5% CO_2. Before staining experiment with peptides, the cells were seeded on the surface of MatTek glass bottom microwell dishes using 1 mL media. After 1 day, the cells were washed twice with warm DMEM media, incubated at 37 °C with 2 μM peptide **9k** for 90 min and fixed. The cells were subsequently stained with DAPI for 10 min before imaging by a Leica TCS SP8 confocal fluorescence microscope.

Supplementary Methods

Chemicals and instrumentation

All the solvents were obtained from Sigma-Aldrich, Alfa-Aesar and Acros, and used directly without further purification. Amino acids and derivatives were obtained from commercial sources. EDCI (N-(3-Dimethylaminopropyl)- N′-ethylcarbodiimide hydrochloride), palladium diacetate, silver trifluroacetate, HFIP (hexafluoro-2-propanol), cupric acetate and sodium acetate were commercially available and used without purification. Analytical thin layer chromatography was performed on 0.25 mm silica gel 60-F254. For flash chromatography, silica gel (60 Å, 300-400 mesh) was used. NMR spectra were recorded on Bruker AMX-400 instrument for ^{1}HNMR at 400 MHz and ^{13}CNMR at 100MHz, using TMS as internal standard. The following abbreviations were used to explain multiplicities: s = singlet, d = doublet, t = triplet, q = quartet, m = multiplet, br = broad. Coupling constants, J, were reported in Hertz unit (Hz). High-resolution mass spectra (HRMS) were recorded on an Agilent Mass spectrometer using ESI-TOF (electrospray ionization-time of flight). HPLC profiles were obtained on Agilent 1260 HPLC system

using commercially available columns.

General procedure for the synthesis of benzosulfonamide peptide conjugate

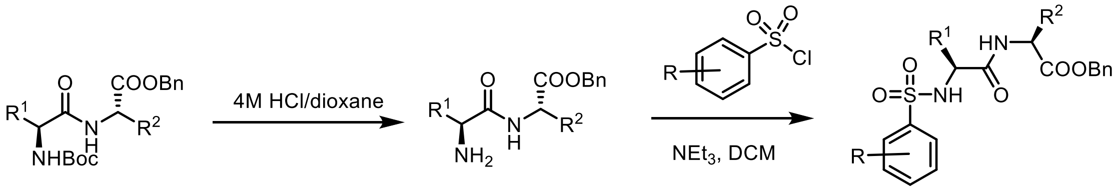

Typically, peptides were prepared by liquid phase peptide synthesis. The resulting peptide was subjected to 4M HCl/dioxane hydrolysis for 4h at 0 °C. Upon completion, the reaction mixture was concentrated and dissolved with DCM before the trimethylamine (TEA, 3.0 eq.) and benzenesulfonyl chloride (1.2 eq.) was added successively. The reaction mixture was further stirred at room temperature for 12h before water was added. The aqueous layer was extracted with DCM for 3 times. The organic layer was combined, dried over anhydrous Na_2SO_4, and concentrated under vacuum. The residue was purified with column chromatography to yield corresponding substrates.

General procedure for Pd-catalyzed olefination of benzylsulfonamide-peptide conjugates

To a 15 ml sealed reaction tube, benzylsulfonamide peptide conjugate (0.20 mmol), $Pd(OAc)_2$ (0.020 mmol), AgOAc (0.60 mmol) and DCE (4.0 ml) was added. The reaction mixture was stirred at 80 °C for 24 h, cooled to room temperature and diluted with ethyl acetate (5.0 ml). The resulting solution was filtered through a Celite pad, concentrated under reduced pressure and further purified by column chromatography. The resulting products were typically obtained as a white solid.

General procedure for Pd-catalyzed cyclization of benzosulfonamide-peptide conjugates

Typically, benzosulfonamide peptide conjugate (0.20 mmol), $Pd(OAc)_2$ (0.024 mmol), AgTFA (0.40 mmol), $Cu(OAc)_2$ (0.40 mmol) and HFIP (4.0 ml) was added to a 15 ml sealed reaction tube (a cylinder thick wall pressure-resistance tube purchased from Beijing Synthware Glass Inc.). The reaction mixture was stirred at 80 °C for 24 h, cooled to room temperature and then diluted with ethyl acetate (5.0 ml). The resulting solution was filtered through a Celite pad,

concentrated under reduced pressure and further purified by column chromatography. The resulting product was typically obtained as a white solid.

General procedure for Pd-catalyzed macrocyclization of peptidosulfonamides

Benzosulfonamide-peptide conjugate (0.20 mmol), $Pd(OAc)_2$ (0.02 mmol), AgOAc (0.40 mmol) and DCE (4.0 ml) was added to a 15 ml sealed reaction tube, and the reaction mixture was stirred at 80 °C for 24 h. Upon completion, the reaction mixture was diluted with ethyl acetate (5.0 ml), filtered through a Celite pad, concentrated under reduced pressure and further purified by column chromatography. The resulting product was typically obtained as a white solid.

Cell culture and staining experiments

U87MG cell were grown and maintained in DMEM media with 10% FBS and 1% penicillin/streptomycin at 37 °C, 5% CO_2. Before staining experiment with peptides, the cells were seeded on the surface of MatTek glass bottom microwell dishes using 1 mL media. After 1 day, the cells were washed twice with warm DMEM media, incubated at 37 °C with 2 µM peptide **9k** for 90 min and fixed. Images were taken using a Leica TCS SP8 confocal fluorescence microscope.

<u>Compound 1a</u>

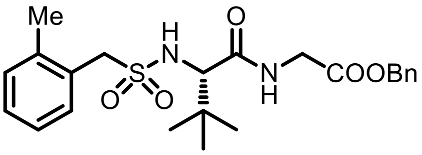

According to the general procedure, the crude residue was purified by flash column chromatography on silica gel (petroleum ether: ethyl acetate= 3:1; R_f = 0.3) to yield compound **1a** (1.02 g, 92% yield). ^{1}H NMR (500 MHz, $CDCl_3$) δ 7.38 – 7.31 (m, 6H), 7.22 – 7.14 (m, 3H), 6.15 (s, 1H), 5.23 – 5.07 (m, 3H), 4.29 (q, *J* = 13.9 Hz, 2H), 4.06 (ddd, *J* = 23.4, 18.3, 5.5 Hz, 2H), 3.61 (dd, *J* = 9.4, 3.1 Hz, 1H), 2.41 (s, 3H), 1.01 (s, 9H). ^{13}C NMR (125 MHz, $CDCl_3$) δ 170.5, 169.2, 138.5, 134.9, 131.6, 130.8, 128.8, 128.7, 128.5, 127.2, 126.2, 67.5, 65.3, 57.1, 41.4, 34.7, 26.5, 19.8. HRMS (ESI) $[M+Na]^+$ m/z calcd for $C_{23}H_{30}N_2O_5SNa$ 469.1773, found 469.1774.

Compound 3aa

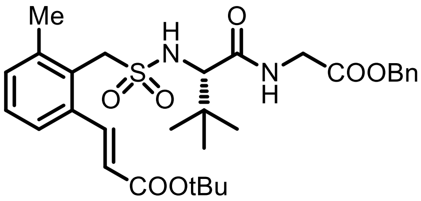

According to the general procedure, the crude residue was purified by flash column chromatography on silica gel (petroleum ether: ethyl acetate= 3:1; R_f= 0.36) to produce compound **3aa** (21 mg, 92% yield).^{1}H NMR (500 MHz, $CDCl_3$) δ 8.44 (s, 1H), 8.12 (d, *J* = 15.8 Hz, 1H), 7.55 – 7.43 (m, 1H), 7.35 – 7.31 (m, 3H), 7.29 – 7.24 (m, 4H), 6.33 (d, *J* =15.8 Hz, 1H), 5.48 (d, *J* = 10.0 Hz, 1H), 5.09 (dd, *J* = 37.6, 12.2 Hz, 2H), 4.75 (d, *J* = 14.4 Hz, 1H), 4.47 (d, *J* = 14.5 Hz, 1H), 4.35 (dd, *J* = 17.6, 6.7 Hz, 1H), 3.90 (dd, *J* = 17.6, 5.3 Hz, 1H), 3.80 (d, *J* = 10.0 Hz, 1H), 2.52 (s, 3H), 1.54 (s, 9H), 1.08 (s, 9H).^{13}C NMR (125 MHz, $CDCl_3$) δ 171.6, 169.3, 168.5, 143.1, 139.2, 135.8, 135.1, 132.4, 128.6, 128.6, 128.4, 128.3, 127.8, 124.7, 121.3, 81.6, 67.1, 65.4, 53.4, 41.3, 34.8, 28.2, 26.4, 20.3. HRMS (ESI) $[M+Na]^+$ m/z calcd for $C_{30}H_{40}N_2O_7SNa$ 595.2454, found 595.2504.

Compound 1b

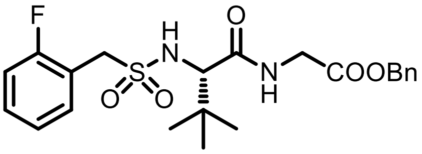

According to the general procedure, the crude residue was purified by flash column chromatography on silica gel (petroleum ether: ethyl acetate= 3:1; R_f= 0.4) to produce compound **1b** (0.98 g, 89% yield). ^{1}H NMR (400 MHz, $CDCl_3$) δ 7.49 (td, *J* = 7.5, 1.6 Hz, 1H), 7.34 (d, *J* = 4.0 Hz, 4H), 7.31 – 7.26 (m, 4H), 7.13 (td, *J* = 7.6, 1.0 Hz, 1H), 7.09 – 7.02 (m, 1H), 6.43 (t, *J* = 5.3 Hz, 1H), 5.30 (d, *J* = 9.4 Hz, 1H), 5.21 – 5.10 (m, 2H), 4.33 (dd, *J* = 36.8, 14.0 Hz, 2H), 4.16 – 3.99 (m, 2H), 3.68 (d, *J* = 9.5 Hz, 1H), 1.00 (s, 9H). ^{13}C NMR (100 MHz, $CDCl_3$) δ 170.5, 169.3, 162.6, 160.1, 134.9, 132.5, 132.5, 130.7, 130.7, 128.7, 128.7, 128.5, 124.4, 124.4, 116.5, 116.4, 115.8, 115.6, 67.4, 65.2, 52.4, 52.4, 41.4, 34.7, 26.5. HRMS (ESI) $[M+Na]^+$ m/z calcd for $C_{22}H_{27}FN_2O_5SNa$ 473.1522, found 473.1523.

Compound 3ba

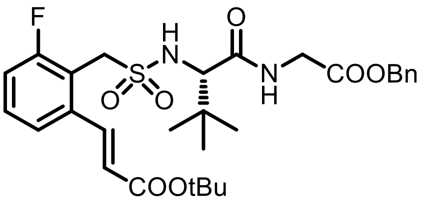

According to the general procedure, the crude residue was purified by flash column chromatography on silica gel (petroleum ether: ethyl acetate= 3:1; R_f= 0.52) to produce compound **3ba** (19 mg, 78% yield).^{1}H NMR (500 MHz, $CDCl_3$) δ 8.05 (t, *J* = 5.8 Hz, 1H), 7.99 (d, *J* = 15.8 Hz, 1H), 7.42 (d, *J* = 7.8

Hz, 1H), 7.36 – 7.28 (m, 4H), 7.25 (m, 2H), 7.13 (t, J = 8.6 Hz, 1H), 6.35 (d, J = 15.8 Hz, 1H), 5.56 (t, J =10.9 Hz, 1H), 5.09 (dd, J = 27.3, 12.2 Hz, 2H), 4.60 (dd, J = 38.8, 14.6 Hz, 2H), 4.33 (dd, J = 17.6, 6.6 Hz, 1H), 3.89 (dd, J = 17.6, 5.3 Hz, 1H), 3.75 (d, J = 10.0 Hz, 1H), 1.51 (s, 9H), 1.05 (s, 9H). ^{13}C NMR (125 MHz, $CDCl_3$) δ 171.3, 169.4, 167.7, 162.5, 160.5, 140.9, 137.3, 135.1, 130.2, 130.1, 128.6, 128.5, 128.3, 122.5, 116.9, 116.8, 81.7, 67.2, 65.5, 49.8, 41.4, 34.8, 28.1, 26.5. HRMS (ESI) $[M+Na]^+$ m/z calcd for $C_{29}H_{37}FN_2O_7SNa$ 599.2203, found 599.2207.

Compound 1c

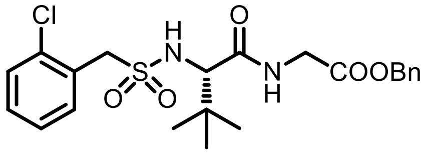

According to the general procedure, the crude residue was purified by flash column chromatography on silica gel (petroleum ether: ethyl acetate= 3:1; R_f= 0.3) to produce compound **1c** (1.20 g, 90 % yield). ^{1}H NMR (400 MHz, $CDCl_3$) δ 7.56 – 7.51 (m, 1H), 7.38 – 7.33 (m, 1H), 7.33 – 7.28 (m, 4H), 7.25 – 7.18 (m, 2H), 6.88 (d, J = 4.7 Hz, 1H), 5.61 (d, J = 9.5 Hz, 1H), 5.20 – 5.07 (m, 2H), 4.49 (q, J = 33.6, 13.9 Hz, 2H), 4.04 (qd, J = 18.1, 5.4 Hz, 2H), 3.78 (d, J = 9.5 Hz, 1H), 0.99 (s, 9H). ^{13}C NMR (100 MHz, $CDCl_3$) δ 170.9, 169.4, 135.4, 135.0, 132.0, 129.9, 129.8, 128.6, 128.5, 128.5, 127.3, 127.1, 67.3, 64.9, 56.2, 41.4, 34.6, 26.5. HRMS (ESI) $[M+Na]^+$ m/z calcd for $C_{21}H_{25}N_3O_7SNa$ 489.1227, found 489.1229.

Compound 3ca

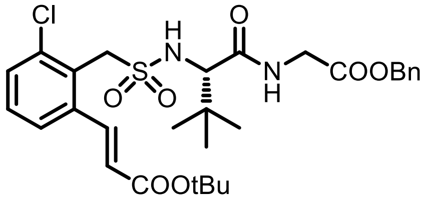

According to the general procedure, the crude residue was purified by flash column chromatography on silica gel (petroleum ether: ethyl acetate= 3:1; R_f= 0.44) to produce compound **3ca** (18 mg, 82% yield). ^{1}H NMR (500 MHz, $CDCl_3$) δ 8.21 (s, 1H), 8.06 (d, J = 15.8 Hz, 1H), 7.48 (dd, J = 23.3, 7.9 Hz, 2H), 7.33 – 7.23 (m, 6H), 6.32 (d, J = 15.8 Hz, 1H), 5.53 (d, J = 10.0 Hz, 1H), 5.08 (q, J = 12.2 Hz, 2H), 4.78 (dd, J = 39.5, 14.3 Hz, 2H), 4.34 (dd, J = 17.6, 6.7 Hz, 1H), 3.87 (dd, J = 17.6, 5.2 Hz, 1H), 3.76 (d, J = 10.1 Hz, 1H), 1.52 (s, 9H), 1.05 (s, 9H). ^{13}C NMR (125 MHz, $CDCl_3$) δ 171.4, 169.4, 167.9, 142.0, 137.6, 136.4, 135.0, 131.1, 129.6, 128.6, 128.5, 128.3, 127.4, 125.4, 122.7, 81.9, 67.2, 65.4, 53.6, 41.3, 34.8, 28.1, 26.5. HRMS (ESI) $[M+Na]^+$ m/z calcd for $C_{29}H_{37}ClN_2O_7SNa$ 615.1908, found 615.1914.

Compound 1d

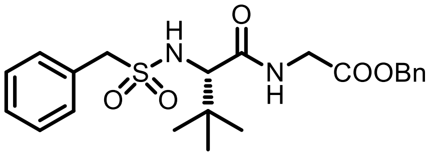

According to the general procedure, the crude residue was purified by flash column chromatography on silica gel (petroleum ether: ethyl acetate= 3:1; R_f= 0.35) to produce compound **1d** (0.90 g, 92% yield). ^{1}H NMR (400 MHz, $CDCl_3$) δ 7.38 (m, 3H), 7.32 (s, 4H), 7.31 – 7.27 (m, 3H), 6.75 (s, 1H), 5.43 (d, *J* = 6.5 Hz, 1H), 5.13 (q, *J* = 12.1 Hz, 2H), 4.26 (s, 2H), 4.00 (d, *J* = 5.3 Hz, 2H), 3.79 – 3.61 (m, 1H), 0.99 (s, 9H); ^{13}C NMR (100 MHz, $CDCl_3$) δ 170.9, 169.5, 135.1, 130.9, 128.7, 128.6, 128.5, 67.4, 65.0, 59.5, 41.4, 34.7, 26.6; HRMS (ESI) $[M+Na]^+$ m/z calcd for $C_{22}H_{28}N_2O_5SNa$ 455.1617, found 455.1618.

Compound 3da(mono)

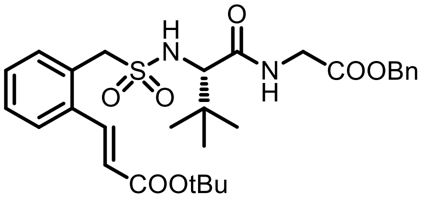

According to the general procedure, the crude residue was purified by flash column chromatography on silica gel (petroleum ether: ethyl acetate= 3:1; R_f= 0.47) to produce compound **3da(mono)** (13 mg, 9% yield).^{1}H NMR (400 MHz, $CDCl_3$) δ 8.03 (d, *J* = 15.8 Hz, 2H), 7.62 (dd, *J* = 6.1, 2.9 Hz, 1H), 7.45 – 7.34 (m, 4H), 7.33 – 7.18 (m, 5H), 6.34 (d, *J* = 15.8 Hz, 1H), 5.41 (d, *J* = 9.9 Hz, 1H), 5.09 (q, *J* = 12.2 Hz, 2H), 4.82 (d, *J* = 14.1 Hz, 1H), 4.30 (dd, *J* = 17.6, 6.4 Hz, 1H), 4.19 (d, *J* = 14.1 Hz, 1H), 3.91 (dd, *J* = 17.6, 5.4 Hz, 1H), 3.73 (d, *J* = 9.9 Hz, 1H), 1.51 (s, 9H), 1.04 (s, 9H).^{13}C NMR (100 MHz, $CDCl_3$) δ 171.5, 169.3, 168.0, 141.9, 135.1, 135.0, 132.0, 130.2, 129.1, 128.9, 128.6, 128.5, 128.4, 126.8, 121.4, 81.5, 67.2, 65.5, 56.8, 41.4, 34.8, 28.2, 26.4. HRMS (ESI) $[M+Na]^+$ m/z calcd for $C_{29}H_{38}N_2O_7SNa$ 581.2297, found 581.2316.

Compound 3da(di)

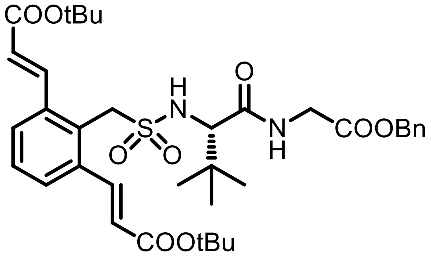

According to the general procedure, the crude residue was purified by flash column chromatography on silica gel (petroleum ether: ethyl acetate= 3:1; R_f= 0.50) to produce compound **3da(di)** (22 mg, 79% yield).^{1}H NMR (400 MHz, $CDCl_3$) δ 8.37 (t, *J* = 6.0 Hz, 1H), 8.13 (d, *J* = 15.8 Hz, 2H), 7.60 (d, *J* = 7.8 Hz, 2H), 7.37 (t, *J* = 7.8 Hz, 1H), 7.31 – 7.19 (m, 5H), 6.30 (d, *J* = 15.8 Hz, 2H), 5.53 (d, *J* = 10.1 Hz, 1H), 5.06 (q, *J* = 12.2 Hz, 2H), 4.65 (dd, *J* = 106.7, 14.7 Hz, 2H), 4.35 (dd, *J* = 17.5, 6.7 Hz, 1H), 3.84 (dd, *J* = 17.5, 6.7 Hz, 1H), 3.75 (d, *J* = 10.1 Hz, 1H), 1.53 (s, 18H), 1.05 (s, 9H).^{13}C NMR (100 MHz, $CDCl_3$) δ 171.6, 169.4, 136.6, 129.0, 128.6, 128.5, 128.3, 128.3, 127.9, 67.2, 65.4, 52.6, 41.3, 34.8, 28.2, 26.4. HRMS (ESI) $[M+Na]^+$ m/z calcd for $C_{36}H_{48}N_2O_9SNa$ 707.2978, found 707.3012.

Compound 1e

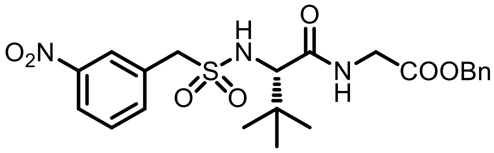

According to the general procedure, the crude residue was purified by flash column chromatography on silica gel (petroleum ether: ethyl acetate= 3:1; R_f= 0.35) to produce compound **1e** (0.82 g, 86% yield). ^{1}H NMR (400 MHz, $CDCl_3$) δ 8.27 (t, *J* = 1.8 Hz, 1H), 8.21 – 8.14 (m, 1H), 7.77 (d, *J* = 7.8 Hz, 1H), 7.50 (t, *J* = 8.0 Hz, 1H), 7.32 (s, 4H), 6.74 (t, *J* = 5.6 Hz, 1H), 5.44 (d, *J* = 9.6 Hz, 1H), 5.16 (s, 2H), 4.38 (dd, *J* = 30.5, 13.9 Hz, 2H), 4.12 (ddd, *J* = 23.3, 18.1, 5.7 Hz, 2H), 3.78 (d, *J* = 9.6 Hz, 1H), 1.01 (s, 9H). ^{13}C NMR (100 MHz, $CDCl_3$) δ 170.8, 169.6, 148.2, 137.1, 134.9, 131.1, 129.6, 128.6, 128.6, 128.5, 126.0, 123.6, 67.5, 65.2, 58.3, 41.3, 34.7, 26.5. HRMS (ESI) $[M+Na]^+$ m/z calcd for $C_{22}H_{27}N_3O_7SNa$ 500.1467, found 500.1466.

Compound 3ea(mono)

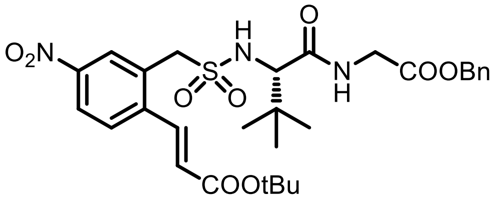

According to the general procedure, the crude residue was purified by flash column chromatography on silica gel (petroleum ether: ethyl acetate= 3:1; R_f = 0.41) to produce compound **3ea(mono)** (9 mg, 30% yield).^{1}H NMR (400 MHz, $CDCl_3$) δ 8.28 (d, *J* = 2.3 Hz, 1H), 8.22 (dd, *J* = 8.6, 2.3 Hz, 1H), 8.05 (d, *J* = 15.9 Hz, 1H), 7.91 (t, *J* = 5.9 Hz, 1H), 7.75 (d, *J* = 8.6 Hz, 1H), 7.31 (dd, *J* = 4.1, 2.4 Hz, 2H), 7.26 – 7.22 (m, 1H), 6.43 (d, *J* = 15.9 Hz, 1H), 5.58 (d, *J* = 10.0 Hz, 1H), 5.09 (dd, *J* = 27.3, 12.2 Hz, 2H), 4.93 (d, *J* = 14.1 Hz, 1H), 4.41 (dd, *J* = 17.6, 6.9 Hz, 1H), 4.26 (d, *J* = 14.1 Hz, 1H), 3.84 (dd, *J* = 17.6, 5.2 Hz, 1H), 3.72 (d, *J* = 10.0 Hz, 1H), 1.53 (s, 9H), 1.05 (s, 9H).^{13}C NMR (100 MHz, $CDCl_3$) δ 171.3, 169.6, 167.2, 148.2, 141.6, 139.8, 135.0, 130.7, 128.6, 128.3, 127.9, 126.9, 125.2, 123.9, 82.3, 67.4, 65.7, 56.4, 41.4, 34.8, 28.1, 26.5.HRMS (ESI) $[M+Na]^+$ m/z calcd for $C_{29}H_{37}N_3O_9SNa$ 626.2418, found 626.2458.

Compound 3ea(di)

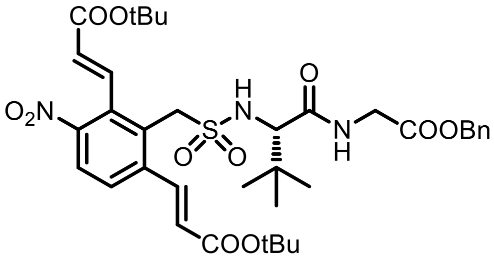

According to the general procedure, the crude residue was purified by flash column chromatography on silica gel (petroleum ether: ethyl acetate= 3:1; R_f = 0.49) to produce compound **3ea(di)** (16 mg, 45% yield).^{1}H NMR (400 MHz, $CDCl_3$) δ 8.12 (d, *J* = 15.8 Hz, 1H), 7.94 (d, *J* = 16.4 Hz, 1H), 7.87 (d, *J* = 8.6 Hz, 1H), 7.67 (d, *J* = 8.6 Hz, 1H), 7.31 (m, 2H), 7.27 – 7.25 (m, 1H), 6.37 (d, *J* = 15.8 Hz, 1H), 5.99 (d, *J* = 16.2 Hz, 1H), 5.51 (d, *J* = 10.2 Hz, 1H), 5.09 (q, *J* = 12.2 Hz, 2H), 4.76 (d, *J* = 14.4 Hz, 1H), 4.43 (dd, *J* = 17.6, 7.2 Hz, 2H), 3.80 (dd, *J* = 17.6, 5.0 Hz, 1H), 1.54 (s, 9H), 1.53 (s, 9H), 1.03 (s, 9H).^{13}C NMR (125 MHz, $CDCl_3$) δ 171.4, 169.6, 149.5, 140.8, 138.1, 134.9, 128.6, 128.3, 128.1, 124.3, 67.4, 65.3, 41.3, 34.8, 28.1, 26.4. HRMS (ESI) $[M+Na]^+$ m/z calcd for $C_{36}H_{47}N_3O_{11}SNa$ 752.2829, found 752.2854.

Compound 1f

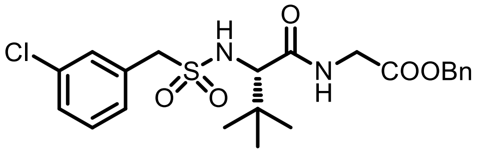

According to the general procedure, the crude residue was purified by flash column chromatography on silica gel (petroleum ether: ethyl acetate= 3:1; R_f= 0.32) to produce compound **1f** (1.21 g, 92% yield). ^{1}H NMR (500 MHz, $CDCl_3$) δ 7.41 (d, *J* = 1.7 Hz, 1H), 7.39 – 7.26 (m, 8H), 6.49 (d, *J* = 4.4 Hz, 1H), 5.37 – 5.26 (m, 1H), 5.23 – 5.15 (m, 2H), 4.29 – 4.20 (m, 2H), 4.18 – 4.04 (m, 2H), 3.71 – 3.67 (m, 1H), 1.02 (s, 9H). ^{13}C NMR (125 MHz, $CDCl_3$) δ 170.6, 169.4, 134.9, 134.3, 130.9, 130.7, 129.9, 129.0, 128.8, 128.6, 128.5, 67.5, 65.2, 58.8, 41.3, 34.7, 26.5. HRMS (ESI) $[M+Na]^+$ m/z calcd for $C_{22}H_{27}ClN_2O_5SNa$ 489.1227, found 489.1228.

Compound 3fa(mono)

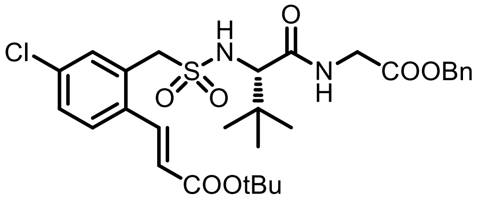

According to the general procedure, the crude residue was purified by flash column chromatography on silica gel (petroleum ether: ethyl acetate= 3:1; R_f = 0.39) to produce compound **3fa(mono)** (9 mg, 9% yield).^{1}H NMR (500 MHz, $CDCl_3$) δ 8.07 (t, *J* = 5.9 Hz, 1H), 7.98 (d, *J* = 15.8 Hz, 1H), 7.58 (d, *J* = 8.4 Hz, 1H), 7.43 (d, *J* = 2.1 Hz, 1H), 7.35(ddd, *J* = 6.4, 5.7, 1.7 Hz, 5H), 7.28 – 7.25 (m, 1H), 6.34 (d, *J* = 15.8 Hz, 1H), 5.46 (d, *J* = 10.0 Hz, 1H), 5.11 (dd, *J* = 33.1, 12.2 Hz, 2H), 4.85 (d, *J* = 14.1 Hz, 1H), 4.37 (dd, *J* = 17.6, 6.6 Hz, 1H), 4.14 (d, *J* = 14.2 Hz, 1H), 3.88 (dd, *J* = 17.6, 5.3 Hz, 1H), 3.74 (d, *J* = 10.0 Hz, 1H), 1.53 (s, 9H), 1.07 (s, 9H).^{13}C NMR (125 MHz, $CDCl_3$) δ 171.4, 169.4, 167.9, 140.7, 135.9, 135.0, 133.5, 131.9, 130.7, 129.3, 128.6, 128.5,

128.3, 128.0, 121.7, 81.7, 67.3, 65.5, 56.4, 41.4, 34.8, 28.1, 26.4. HRMS (ESI) $[M+Na]^+$ m/z calcd for $C_{29}H_{37}ClN_2O_7SNa$ 615.1908, found 615.1916.

Compound 3fa(di)

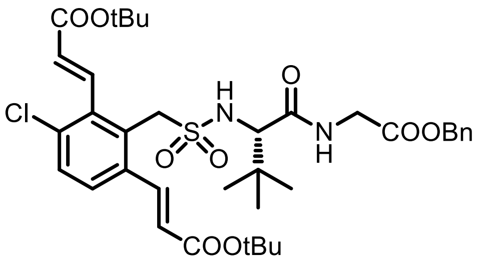

According to the general procedure, the crude residue was purified by flash column chromatography on silica gel (petroleum ether: ethyl acetate= 3:1; R_f= 0.46) to produce compound **3fa(di)** (73 mg, 80% yield).^{1}H NMR (500 MHz, $CDCl_3$) δ 8.31 (s, 1H), 8.09 (d, *J* = 15.7 Hz, 1H), 7.80 (d, *J* = 16.4 Hz, 1H), 7.49 (dd, *J* = 30.8, 8.5 Hz, 2H), 7.38 –7.24 (m, 6H), 6.34 (m, 2H), 5.50 (d, *J* = 10.1 Hz, 1H), 5.17 – 5.06 (m, 2H), 4.75 (d, *J* = 14.4 Hz, 1H), 4.54 – 4.36 (m, 2H), 3.84 (dd, *J* = 17.6, 5.1 Hz, 1H), 3.68 (s, 1H), 1.58 (s, 9H), 1.55 (s, 9H), 1.06 (s, 9H).^{13}C NMR (125 MHz, $CDCl_3$) δ 171.5, 169.5, 139.2, 135.0, 130.4, 129.0, 128.6, 128.5, 128.3, 67.3, 65.3, 41.3, 34.8, 28.2, 28.2, 26.4. HRMS (ESI) $[M+Na]^+$ m/z calcd for $C_{36}H_{47}ClN_2O_9SNa$ 741.2588, found 741.2605.

Compound 1g

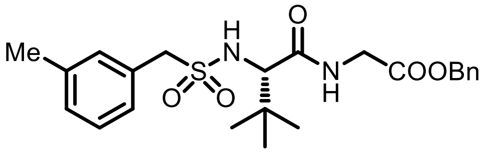

According to the general procedure, the crude residue was purified by flash column chromatography on silica gel (petroleum ether: ethyl acetate= 3:1; R_f = 0.36) to produce compound **1g** (0.93 g, 94% yield). ^{1}H NMR (400 MHz, $CDCl_3$) δ 7.39 – 7.29 (m, 4H), 7.25 – 7.15 (m, 3H), 7.14 – 7.08 (m, 1H), 6.39 (t, *J* = 5.3 Hz, 1H), 5.21 – 5.12 (m, 3H), 4.21 (s, 2H), 4.05 (dd, *J* = 7.6, 5.5 Hz, 2H), 3.65 (d, *J* = 9.4 Hz, 1H), 2.33 (s, 3H), 1.00 (s, 9H). ^{13}C NMR (100 MHz, $CDCl_3$) δ 170.6, 169.3, 138.4, 135.0, 131.6, 129.4, 128.7, 128.6, 128.5, 127.8, 67.4, 65.2, 59.5, 41.3, 34.7, 26.5, 21.3.HRMS (ESI) $[M+Na]^+$ m/z calcd for $C_{23}H_{30}N_2O_5SNa$ 469.1773, found 469.1774.

Compound 3ga(mono)

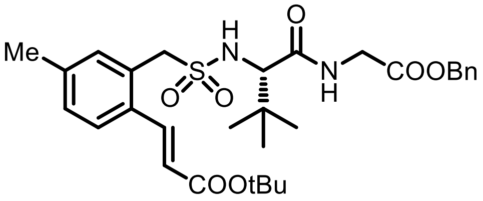

According to the general procedure, the crude residue was purified by flash column chromatography on silica gel (petroleum ether: ethyl acetate= 3:1; R_f = 0.43) to produce compound **3ga(mono)** (10 mg, 9% yield). ^{1}H NMR (500 MHz, $CDCl_3$) δ 8.15 (t, *J* = 5.9 Hz, 1H), 7.99 (d, *J* = 15.8 Hz, 1H), 7.54 (d, *J* = 8.0 Hz, 1H), 7.34 – 7.28 (m, 3H), 7.24 (dd, *J* = 9.2, 6.9 Hz, 2H), 7.18 (d, *J* = 8.0 Hz, 1H), 6.31 (d, *J* = 15.8 Hz, 1H), 5.42 (d, *J* = 9.9 Hz, 1H), 5.08 (dd, *J* = 29.1, 12.2 Hz, 2H), 4.83 (d, *J* = 14.1 Hz, 1H), 4.30 (dd, *J* = 17.6, 6.5 Hz, 1H), 4.15 (d, *J* = 14.2 Hz, 1H), 3.90 (dd, *J* = 17.6, 5.5 Hz, 1H), 3.74 (d, *J* = 10.0 Hz, 1H), 2.37 (s, 3H), 1.50 (s, 9H), 1.05 (s, 9H). ^{13}C NMR (125 MHz, $CDCl_3$) δ 171.5, 169.3, 168.3, 141.8, 140.7, 135.1, 132.6, 132.0, 130.0, 128.9, 128.6, 128.5, 128.4, 126.7, 120.1, 81.4, 67.2, 65.5, 56.8, 41.4, 34.8, 28.2, 26.5, 21.3. HRMS (ESI) $[M+Na]^+$ m/z calcd for $C_{30}H_{40}N_2O_7SNa$ 595.2454, found 595.2484.

Compound 3ga(di)

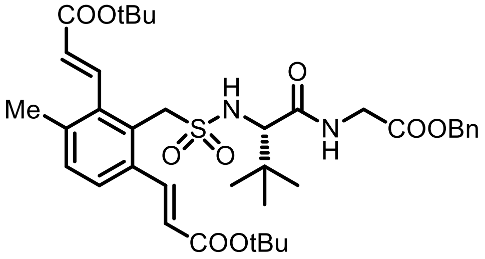

According to the general procedure, the crude residue was purified by flash column chromatography on silica gel (petroleum ether: ethyl acetate= 3:1; R_f = 0.50) to produce compound **3ga(di)** (66 mg, 80% yield). ^{1}H NMR (500 MHz, $CDCl_3$) δ 8.11 (d, *J* = 15.7 Hz, 1H), 7.92 (d, *J* = 16.4 Hz, 1H), 7.52 (d, *J* = 8.0 Hz, 1H), 7.33 (m, 3H), 7.26 (m, 3H), 6.32 (d, *J* = 15.7 Hz, 1H), 6.08 (d, *J* = 16.3 Hz, 1H), 5.46 (d, *J* = 10.1 Hz, 1H), 5.15 – 5.05 (m, 2H), 4.71 (d, *J* = 14.4 Hz, 1H), 4.49 (d, *J* = 14.0 Hz, 1H), 4.36 (dd, *J* = 17.6, 6.8 Hz, 1H), 3.88 (dd, *J* = 17.6, 5.2 Hz, 1H), 3.71 (s, 1H), 2.38 (s, 3H), 1.58 (s, 9H), 1.55 (s, 9H), 1.06 (s, 9H). ^{13}C NMR (125 MHz, $CDCl_3$) δ 171.6, 169.4, 135.1, 131.1, 128.6, 128.5, 128.3, 127.0, 67.2, 65.2, 41.3, 34.8, 28.2, 26.4, 21.5. HRMS (ESI) $[M+Na]^+$ m/z calcd for $C_{37}H_{50}N_2O_9SNa$ 721.3135, found 721.3143.

Compound 1h

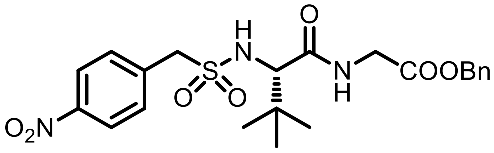

According to the general procedure, the crude residue was purified by flash column chromatography on silica gel (petroleum ether: ethyl acetate= 3:1; R_f = 0.35) to produce compound **1h** (0.92 g, 83% yield). ^{1}H NMR (400 MHz, $CDCl_3$) δ 8.18 (d, *J* = 8.8 Hz, 2H), 7.61 – 7.56 (m, 2H), 7.34 (t, *J* = 1.8 Hz, 4H), 6.48 (t, *J* = 5.4 Hz, 1H), 5.34 (d, *J* = 9.5 Hz, 1H), 5.23 – 5.10 (m, 2H), 4.36 (d, *J* = 1.3 Hz, 2H), 4.10 (ddd, *J* = 23.3, 18.1, 5.6 Hz, 2H), 3.68 (dd, *J* = 13.9, 7.7 Hz, 1H), 1.01 (s, 9H). ^{13}C NMR (100 MHz, $CDCl_3$) δ 170.6, 169.4, 148.0, 136.2, 134.9, 131.9,

128.7, 128.7, 128.5, 123.7, 67.6, 65.4, 58.8, 41.4, 34.8, 26.5. HRMS (ESI) $[M+Na]^+$ m/z calcd for $C_{22}H_{27}N_3O_7SNa$ 500.1467, found 500.1466.

Compound 3ha(mono)

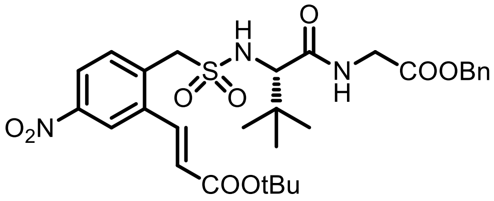

According to the general procedure, the crude residue was purified by flash column chromatography on silica gel (petroleum ether: ethyl acetate= 3:1; R_f = 0.41) to produce compound **3ha(mono)** (8 mg, 17% yield). ^{1}H NMR (400 MHz, $CDCl_3$) δ 8.45 (d, *J* = 2.3 Hz, 1H), 8.21 (dd, *J* = 8.4, 2.3 Hz, 1H), 8.03 (d, *J* = 15.8 Hz, 1H), 7.99 – 7.94 (m, 1H), 7.61 (d, *J* = 8.4 Hz, 1H), 7.37 – 7.29 (m, 3H), 7.24 (m, 1H), 6.47 (d, *J* = 15.8 Hz, 1H), 5.47 (d, *J* = 10.0 Hz, 1H), 5.08 (q, *J* = 12.2 Hz, 2H), 4.96 (d, *J* = 13.9 Hz, 1H), 4.39 (dd, *J* = 17.6, 6.8 Hz, 1H), 4.25 (d, *J* = 14.0 Hz, 1H), 3.83 (dd, *J* = 17.6, 5.2 Hz, 1H), 3.71 (d, *J* = 10.0 Hz, 1H), 1.53 (s, 9H), 1.05 (s, 9H). ^{13}C NMR (125 MHz, $CDCl_3$) δ 171.3, 169.5, 167.4, 148.2, 139.8, 136.9, 135.6, 133.0, 128.6, 128.6, 128.3, 124.3, 121.8, 82.3, 67.3, 65.6, 56.4, 41.4, 34.8, 28.1, 26.4. HRMS (ESI) $[M+Na]^+$ m/z calcd for $C_{29}H_{37}N_3O_9SNa$ 626.2148, found 626.2156.

Compound 3ha(di)

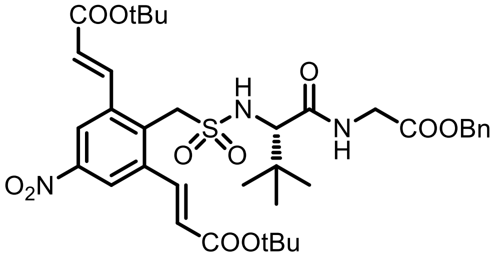

According to the general procedure, the crude residue was purified by flash column chromatography on silica gel (petroleum ether: ethyl acetate= 3:1; R_f = 0.50) to produce compound **3ha(di)** (38 mg, 70% yield). ^{1}H NMR (400 MHz, $CDCl_3$) δ 8.40 (s, 2H), 8.31 – 8.23 (m, 1H), 8.13 (d, *J* = 15.8 Hz, 1H), 7.30 (m, 2H), 7.24 – 7.21 (m, 2H), 6.43 (d, *J* = 15.8 Hz, 1H), 5.65 (d, *J* = 10.0 Hz, 1H), 5.06 (q, *J* = 12.2 Hz, 2H), 4.92 (d, *J* = 14.5 Hz, 1H), 4.54 (d, *J* = 14.5 Hz, 1H), 4.42 (dd, *J* = 17.5, 7.1 Hz, 1H), 3.77 (dd, *J* = 17.6, 5.2 Hz, 1H), 3.73 (d, *J* = 10.2 Hz, 1H), 1.55 (s, 18H), 1.05 (s, 9H). ^{13}C NMR (125 MHz, $CDCl_3$) δ 171.4, 169.6, 147.9, 138.6, 134.1, 128.6, 128.6, 128.2, 122.2, 67.3, 65.6, 52.7, 41.4, 34.8, 28.1, 26.4. HRMS (ESI) $[M+Na]^+$ m/z calcd for $C_{36}H_{47}N_3O_{11}SNa$ 752.2829, found 752.2823.

Compound 1i

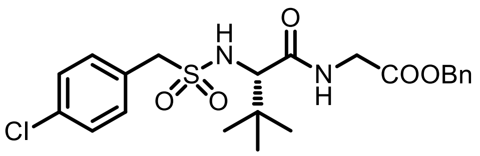

According to the general procedure, the crude residue was purified by flash column chromatography on silica gel (petroleum ether: ethyl acetate= 2:1; R_f = 0.48) to produce compound **1i** (0.93 g, 86% yield).^{1}H NMR (400 MHz, $CDCl_3$) δ 7.34 - 7.33 (m, 4H), 7.32 -7.30 (m, 4H), 6.44 (t, *J* = 5.4 Hz, 1H), 5.26 (d, *J* = 9.5 Hz, 1H), 5.21 – 5.11 (m, 2H), 4.22 (s, 1H), 4.06 (dd, *J* = 6.6, 5.7 Hz, 1H), 3.65 (d, *J* = 9.5 Hz, 1H), 0.99 (s, 9H). ^{13}C NMR (100 MHz, $CDCl_3$) δ 170.6, 169.4, 134.9, 134.8, 132.3, 128.8, 128.7, 128.5, 127.4, 67.5, 65.2, 58.8 41.3, 34.7, 26.5. HRMS (ESI) $[M+Na]^+$ m/z calcd for $C_{22}H_{27}ClN_2O_5SNa$ 489.1227, found 489.1228.

Compound 3ia(mono)

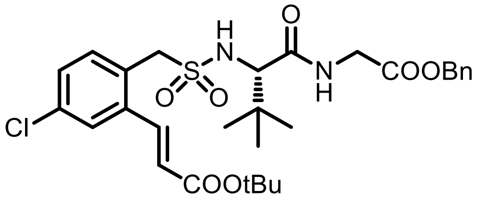

According to the general procedure, the crude residue was purified by flash column chromatography on silica gel (petroleum ether: ethyl acetate= 2:1; R_f = 0.52) to produce compound **3ia(mono)** (12 mg, 24% yield).^{1}H NMR (500 MHz, MeOD) δ 7.98 (d, *J* = 15.8 Hz, 1H), 7.73 (d, *J* = 1.7 Hz, 1H), 7.43 – 7.37 (m, 2H), 7.33 – 7.27 (m, 5H), 6.43 (d, *J* = 15.8 Hz, 1H), 5.13 (d, *J* = 1.2 Hz, 2H), 4.57 (d, *J* = 14.0 Hz, 1H), 4.32 (d, *J* = 14.1 Hz, 1H), 4.16 (d, *J* = 17.6 Hz, 1H), 3.98 (d, *J* = 17.6 Hz, 1H), 3.81 (s, 1H), 1.55 (s, 9H), 1.05 (s, 9H).^{13}C NMR (125 MHz, MeOD) δ 171.9, 169.6, 166.5, 140.3, 136.9, 135.7, 134.5, 134.2, 129.3, 128.1, 128.0, 127.9, 127.8, 126.2, 122.8, 80.9, 66.6, 65.0, 55.2, 40.6, 34.0, 27.0, 25.6. HRMS (ESI) $[M+Na]^+$ m/z calcd for $C_{29}H_{37}ClN_2O_7SNa$ 615.1908, found 615.1915.

Compound 3ia(di)

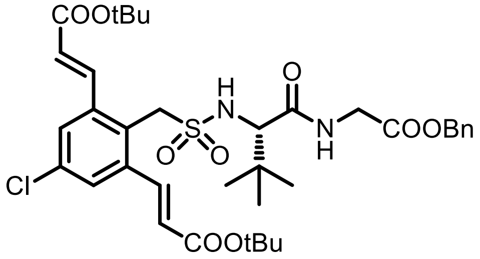

According to the general procedure, the crude residue was purified by flash column chromatography on silica gel (petroleum ether: ethyl acetate= 2:1; R_f = 0.61) to produce compound **3ia(di)** (36 mg, 54% yield).^{1}H NMR (400 MHz, $CDCl_3$) δ 8.28 (t, *J* = 6.0 Hz, 1H), 8.05 (d, *J* = 15.7 Hz, 2H), 7.56 (s, 2H), 7.32 – 7.28 (m, 2H), 7.25 – 7.21 (m, 2H), 6.29 (d, *J* = 15.7 Hz, 2H), 5.53 (d, *J* = 10.1 Hz, 1H), 5.06 (q, *J* = 12.2 Hz, 2H), 4.75 (d, *J* = 14.7 Hz, 1H), 4.44 (d, *J* = 14.7 Hz, 1H), 4.37 (dd, *J* = 17.5, 6.9 Hz, 1H), 3.80 (dd, *J* = 17.5, 5.2 Hz, 1H), 3.71 (d, *J* = 10.2 Hz, 1H), 1.53 (s, 18H), 1.04 (s, 9H).^{13}C NMR (100 MHz, $CDCl_3$) δ 171.5, 169.5, 138.3, 135.1, 128.6, 128.5, 128.3, 128.0, 126.4, 67.2, 65.5, 52.3, 41.4, 34.8, 28.1, 26.4.

HRMS (ESI) $[M+Na]^+$ m/z calcd for $C_{36}H_{47}ClN_2O_9SNa$ 741.2588, found 741.2608.

Compound 1j

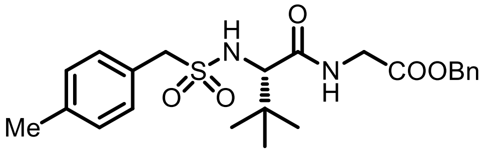

According to the general procedure, the crude residue was purified by flash column chromatography on silica gel (petroleum ether: ethyl acetate= 3:1; R_f= 0.32) to produce compound **1j** (0.62 g, 72% yield). ^{1}H NMR (400 MHz, $CDCl_3$) δ 7.34 (d, *J* = 0.7 Hz, 4H), 7.20 (dd, *J* = 56.2, 7.9 Hz, 4H), 6.46 (m, 1H), 5.21 (d, *J* = 9.5 Hz, 1H), 5.20 – 5.12 (m, 2H), 4.21 (s, 2H), 4.10 – 3.96 (m, 2H), 3.66 (d, *J* = 9.4 Hz, 1H), 2.31 (s, 3H), 0.99 (s, 9H). ^{13}C NMR (100 MHz, $CDCl_3$) δ 170.7, 169.3, 138.5, 134.9, 130.7, 129.4, 128.6, 128.6, 128.5, 125.7, 67.4, 65.1, 59.2, 41.3, 34.7, 26.5, 21.2. HRMS (ESI) $[M+Na]^+$ m/z calcd for $C_{23}H_{30}N_2O_5SNa$ 469.1773, found 469.1774.

Compound 3ja(mono)

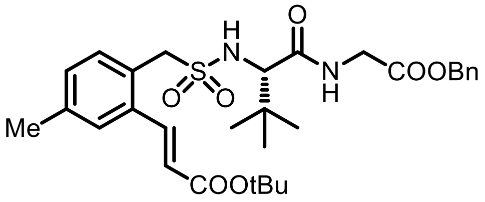

According to the general procedure, the crude residue was purified by flash column chromatography on silica gel (petroleum ether: ethyl acetate= 3:1; R_f= 0.38) to produce compound **3ja(mono)** (14 mg, 25% yield). ^{1}H NMR (500 MHz, MeOD) δ 8.03 (d, *J* = 15.8 Hz, 1H), 7.54 (s, 1H), 7.41 – 7.26 (m, 7H), 7.25 – 7.19 (m, 1H), 6.40 (d, *J* = 15.8 Hz, 1H), 5.12 (d, *J* = 1.1 Hz, 2H), 4.56 (d, *J* = 14.0 Hz, 1H), 4.30 (d, *J* = 14.0 Hz, 1H), 4.15 (d, *J* = 17.6 Hz, 1H), 3.99 (d, *J* = 17.6 Hz, 1H), 3.81 (d, *J* = 3.0 Hz, 2H), 2.39 (s, 3H), 1.55 (s, 9H), 1.05 (s, 9H) ppm. ^{13}C NMR (125 MHz, MeOD) δ 171.9, 169.6, 167.1, 141.9, 138.7, 135.7, 134.8, 132.6, 130.4, 128.1, 128.0, 127.9, 126.9, 126.1, 121.0, 80.6, 66.5, 64.9, 55.5, 40.6, 34.0, 27.0, 25.7, 19.8 ppm. HRMS (ESI) $[M+Na]^+$ m/z calcd for $C_{30}H_{40}N_2O_7SNa$ 595.2454, found 595.2463.

Compound 3ja(di)

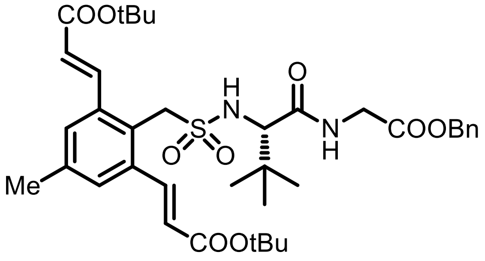

According to the general procedure, the crude residue was purified by flash column chromatography on silica gel (petroleum ether: ethyl acetate= 3:1; R_f= 0.44) to produce compound **3ja(di)**(25 mg, 39% yield). ^{1}H NMR (400

MHz, $CDCl_3$) δ 8.36 (t, *J* = 6.0 Hz, 1H), 8.10 (d, *J* = 15.7 Hz, 2H), 7.43 (s, 2H), 7.32 – 7.20 (m, 5H), 6.29 (d, *J* = 15.7 Hz, 2H), 5.50 (d, *J* = 10.0 Hz, 1H), 5.06 (q, *J* = 12.2 Hz, 2H), 4.61 (dd, *J* = 102.6, 14.7 Hz, 2H), 4.33 (dd, *J* = 17.5, 6.7 Hz, 1H), 3.84 (dd, *J* = 17.5, 5.4 Hz, 1H), 3.74 (d, *J* = 10.1 Hz, 1H), 2.37 (s, 3H), 1.53 (s, 18H), 1.05 (s, 9H). ^{13}C NMR (100 MHz, $CDCl_3$) δ 171.6, 169.4, 138.8, 136.4, 129.1, 128.6, 128.5, 128.3, 125.1, 67.1, 65.4, 52.4, 41.3, 34.8, 28.2, 26.4, 21.2. HRMS (ESI) $[M+Na]^+$ m/z calcd for $C_{37}H_{50}N_2O_9SNa$ 721.3135, found 821.3145.

Compound 3db(mono)

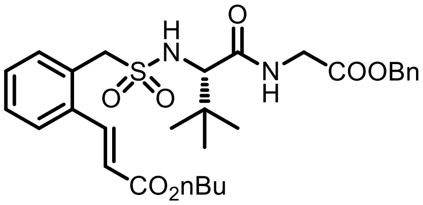

According to the general procedure, the crude residue was purified by flash column chromatography on silica gel (petroleum ether: ethyl acetate= 3:1; R_f = 0.34) to produce compound **3db(mono)** (12 mg, 32% yield). ^{1}H NMR (500 MHz, $CDCl_3$) δ 8.12 (d, *J* = 15.8 Hz, 1H), 7.83 (t, *J* = 5.8 Hz, 1H), 7.65 (dd, *J* = 5.6, 3.4 Hz, 1H), 7.41 (ddt, *J* = 8.9, 5.3, 2.4 Hz, 3H), 7.34 – 7.23 (m, 5H), 6.42 (d, *J* = 15.8 Hz, 1H), 5.37 (d, *J* = 9.9 Hz, 1H), 5.09 (q, *J* = 12.2 Hz, 2H), 4.83 (d, *J* = 14.1 Hz, 1H), 4.31 (dd, *J* = 17.6, 6.5 Hz, 1H), 4.26 – 4.13 (m, 3H), 3.92 (dd, *J* = 17.6, 5.4 Hz, 1H), 3.71 (d, *J* = 9.9 Hz, 1H), 1.74 – 1.64 (m, 2H), 1.43 (dq, *J* = 14.7, 7.4 Hz, 2H), 1.05 (s, 9H), 0.97 (t, *J* = 7.4 Hz, 3H). ^{13}C NMR (125 MHz, $CDCl_3$) δ 171.4, 169.4, 168.7, 142.8, 135.0, 134.8, 132.0, 130.4, 129.1, 129.0, 128.6, 128.5, 128.3, 126.8, 119.5, 67.2, 65.5, 65.0, 56.8, 41.4, 34.8, 30.7, 26.5, 19.2, 13.7. HRMS (ESI) $[M+Na]^+$ m/z calcd for $C_{29}H_{38}N_2O_7SNa$ 581.2297, found 581.2294.

Compound 3db(di)

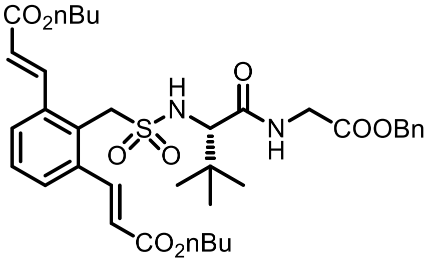

According to the general procedure, the crude residue was purified by flash column chromatography on silica gel (petroleum ether: ethyl acetate= 3:1; R_f = 0.41) to produce compound **3db(di)** (33 mg, 61% yield). ^{1}H NMR (500 MHz, $CDCl_3$) δ 8.23 – 8.16 (m, 3H), 7.64 (d, *J* = 7.8 Hz, 2H), 7.40 (t, *J* = 7.8 Hz, 1H), 7.32 – 7.21 (m, 5H), 6.39 (d, *J* = 15.7 Hz, 2H), 5.48 (d, *J* = 10.1 Hz, 1H), 5.06 (q, *J* = 12.2 Hz, 2H), 4.82 (d, *J* = 14.7 Hz, 1H), 4.52 (d, *J* = 14.7 Hz, 1H), 4.37 (dd, *J* = 17.5, 6.8 Hz, 1H), 4.22 (qt, *J* =

10.9, 6.7 Hz, 4H), 3.85 (dd, J = 17.5, 5.2 Hz, 1H), 3.73 (d, J = 10.1 Hz, 1H), 1.74 – 1.65 (m, 4H), 1.49 – 1.39 (m, 4H), 1.05 (s, 9H), 0.97 (t, J = 7.4 Hz, 6H). ^{13}C NMR (125 MHz, $CDCl_3$) δ 171.5, 169.4, 136.5, 135.0, 129.1, 128.5, 128.5, 128.2, 128.1, 67.2, 65.5, 64.9, 52.5, 41.4, 34.8, 30.7, 26.4, 19.2, 13.8. HRMS (ESI) $[M+Na]^+$ m/z calcd for $C_{36}H_{48}N_2O_9SNa$ 707.2978, found 707.2982.

Compound 3dc(mono)

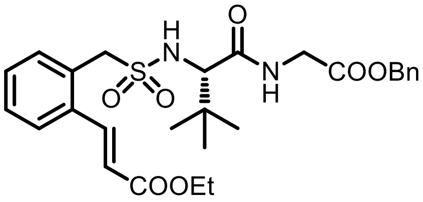

According to the general procedure, the crude residue was purified by flash column chromatography on silica gel (petroleum ether: ethyl acetate= 3:1; R_f= 0.32) to produce compound **3dc(mono)** (11mg, 21% yield).^{1}H NMR (400 MHz, $CDCl_3$) δ 8.12 (d, J = 15.8 Hz, 1H), 7.77 (t, J = 5.8 Hz, 1H), 7.66 – 7.60 (m, 1H), 7.47 – 7.29 (m, 6H), 7.28 – 7.23 (m, 2H), 6.41 (d, J = 15.8 Hz, 1H), 5.34 (d, J = 9.9 Hz, 1H), 5.14 – 5.04 (m, 2H), 4.82 (d, J = 14.1 Hz, 1H), 4.34 – 4.28 (m, 1H), 4.28 – 4.17 (m, 3H), 3.93 (dd, J = 17.6, 5.4 Hz, 1H), 3.71 (d, J = 9.9 Hz, 1H), 1.33 (t, J = 7.1 Hz, 3H), 1.05 (s, 9H).^{13}C NMR (100 MHz, $CDCl_3$) δ 171.4, 169.4, 168.6, 142.9, 135.1, 134.8, 132.1, 130.4, 129.2, 128.6, 128.5, 128.3, 126.8, 119.5, 67.2, 65.5, 61.1, 60.4, 56.8, 53.4, 41.4, 34.8, 26.5, 14.3. HRMS (ESI) $[M+Na]^+$ m/z calcd for $C_{27}H_{34}N_2O_7SNa$ 553.1984, found 553.1980.

Compound 3dc(di)

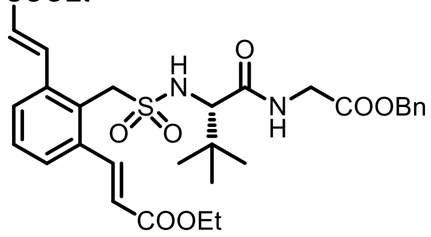

According to the general procedure, the crude residue was purified by flash column chromatography on silica gel (petroleum ether: ethyl acetate= 3:1; R_f= 0.32) to produce compound **3dc(di)** (36 mg, 73% yield).^{1}H NMR (400 MHz, $CDCl_3$) δ 8.20 (d, J = 15.8 Hz, 2H), 8.16 (m, 1H), 7.64 (d, J = 7.8 Hz, 2H), 7.40 (t, J = 7.8 Hz, 1H), 7.31 – 7.28 (m, 3H), 7.23 (dd, J = 6.6, 3.0 Hz, 2H), 6.38 (d, J = 15.8 Hz, 2H), 5.46 (d, J = 10.1 Hz, 1H), 5.06 (q, J = 12.2 Hz, 2H), 4.82 (d, J = 14.6 Hz, 1H), 4.52 (d, J = 14.7 Hz, 1H), 4.36 (dd, J = 17.5, 6.8 Hz, 1H), 4.27 (pd, J = 7.1, 3.7 Hz, 4H), 3.85 (dd, J = 17.5, 5.3 Hz, 1H), 3.73 (d, J

= 10.1 Hz, 1H), 1.35 (t, *J* = 7.1 Hz, 6H), 1.05 (s, 9H).^{13}C NMR (125 MHz, $CDCl_3$) δ 169.5, 136.5, 129.1, 128.6, 128.5, 128.2, 67.2, 65.5, 52.5, 41.4, 34.8, 26.5, 14.3. HRMS (ESI) $[M+Na]^+$ m/z calcd for $C_{32}H_{40}N_2O_9SNa$ 651.2352, found 651.2345.

Compound 3dd(mono)

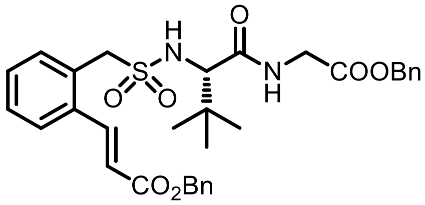

According to the general procedure, the crude residue was purified by flash column chromatography on silica gel (petroleum ether: ethyl acetate= 3:1; R_f= 0.33) to produce compound **3dd(mono)** (9 mg, 12% yield).^{1}H NMR (500 MHz, $CDCl_3$) δ 8.16 (d, *J* = 15.8 Hz, 1H), 7.65 – 7.59 (m, 2H), 7.47 – 7.28 (m, 12H), 7.26 (q, *J* = 2.6 Hz, 3H), 6.47 (d, *J* = 15.8 Hz, 1H), 5.35 (d, *J* = 9.9 Hz, 1H), 5.25 – 5.20 (m, 2H), 5.11 – 5.05 (m, 2H), 4.81 (d, *J* = 14.1 Hz, 1H), 4.25 (dd, *J* = 17.7, 6.5 Hz, 1H), 4.19 (d, *J* = 14.1 Hz, 1H), 3.84 (dd, *J* = 17.7, 5.4 Hz, 1H), 3.70 (d, *J* = 10.0 Hz, 1H), 1.03 (s, 9H).^{13}C NMR (125 MHz, $CDCl_3$) δ 171.4, 169.4, 136.3, 135.7, 135.0, 129.1, 128.6, 128.5, 128.5, 128.4, 128.2, 67.2, 66.8, 65.5, 52.5, 41.3, 34.8, 26.4. HRMS (ESI) $[M+Na]^+$ m/z calcd for $C_{32}H_{36}N_2O_7SNa$ 615.2141, found 615.2131.

Compound 3dd(di)

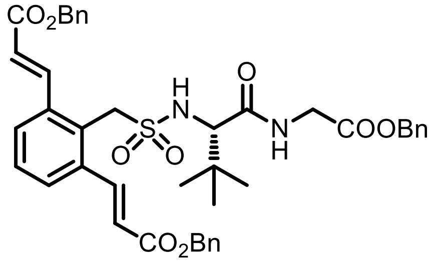

According to the general procedure, the crude residue was purified by flash column chromatography on silica gel (petroleum ether: ethyl acetate= 3:1; R_f= 0.40) to produce compound **3dd(di)** (33 mg, 71% yield).^{1}H NMR (500 MHz, $CDCl_3$) δ 8.29 (d, *J* = 15.8 Hz, 2H), 7.97 (t, *J* = 6.0 Hz, 1H), 7.66 (d, *J* = 7.8 Hz, 2H), 7.40 (m, 11H), 7.32 – 7.23 (m, 5H), 6.46 (d, *J* = 15.7 Hz, 2H), 5.50 (d, *J* = 10.1 Hz, 1H), 5.33 – 5.24 (m, 4H), 5.07 (q, *J* = 12.2 Hz, 2H), 4.83 (d, *J* = 14.6 Hz, 2H), 4.53 (d, *J* = 14.6 Hz, 1H), 4.32 (dd, *J* = 17.5, 6.8 Hz, 1H), 3.76 (dt, *J* = 7.7, 4.9 Hz, 2H), 1.07 (s, 9H).^{13}C NMR (125 MHz, $CDCl_3$) δ 171.3,

169.3, 168.4, 143.5, 135.6, 135.0, 134.7, 132.1, 130.5, 129.2, 129.0, 128.7, 128.6, 128.5, 128.5, 128.3, 128.2, 126.9, 67.2, 66.9, 65.5, 56.7, 41.4, 34.8, 26.4. HRMS (ESI) $[M+Na]^+$ m/z calcd for $C_{42}H_{44}N_2O_9SNa$ 775.2655, found 775.2659.

Compound 3de(mono)

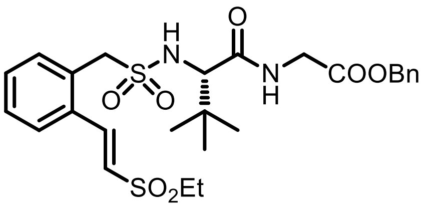

According to the general procedure, the crude residue was purified by flash column chromatography on silica gel (petroleum ether: ethyl acetate= 2:1; R_f= 027) to produce compound **3de(mono)** (13 mg, 43% yield).^{1}H NMR (500 MHz, $CDCl_3$) δ 8.01 (d, *J* = 15.3 Hz, 1H), 7.58 – 7.51 (m, 2H), 7.45 (m, 3H), 7.34 – 7.23 (m, 6H), 6.80 (d, *J* = 15.3 Hz, 1H), 5.42 (d, *J* = 10.1 Hz, 1H), 5.14 – 5.03 (m, 2H), 4.86 (d, *J* = 14.1 Hz, 1H), 4.36 (dd, *J* = 17.6, 7.0 Hz, 1H), 4.13 (d, *J* = 14.1 Hz, 1H), 3.85 (dd, *J* = 17.6, 5.1 Hz, 1H), 3.72 (d, *J* = 10.2 Hz, 1H), 3.11 (q, *J* = 7.4 Hz, 2H), 1.40 (t, *J* = 7.4 Hz, 3H), 1.06 (s, 9H). ^{13}C NMR (125 MHz, $CDCl_3$) δ 171.4, 169.4, 143.8, 135.1, 133.2, 132.6, 131.2, 129.2, 129.2, 128.6, 128.5, 128.3, 127.4, 125.3, 67.2, 65.5, 56.3, 49.4, 41.2, 34.8, 26.5, 7.2. HRMS (ESI) $[M+Na]^+$ m/z calcd for $C_{26}H_{34}N_2O_7S_2Na$ 573.1705, found 573.1711.

Compound 3de(di)

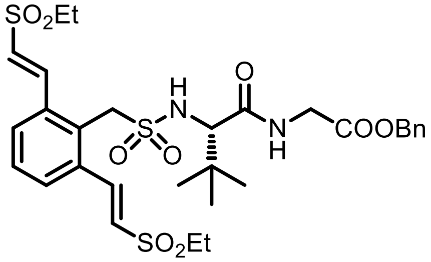

According to the general procedure, the crude residue was purified by flash column chromatography on silica gel (petroleum ether: ethyl acetate= 2:1; R_f= 0.32) to produce compound **3de(di)**(7 mg, 22% yield).^{1}H NMR (500 MHz, $CDCl_3$) δ 8.07 (s, 2H), 7.63 (d, *J* = 7.6 Hz, 3H), 7.48 (t, *J* = 7.7 Hz, 1H), 7.34 – 7.28 (m, 3H), 7.24 (m, 2H), 6.79 (d, *J* = 15.2 Hz, 2H), 5.56 (dd, *J* = 10.2, 3.2 Hz, 1H), 5.05 (q, *J* = 12.2 Hz, 2H), 4.88 (d, *J* = 14.7 Hz, 1H), 4.47 – 4.35 (m, 2H), 3.79 (dd, *J* = 17.6, 5.0 Hz, 1H), 3.72 (d, *J* = 11.4 Hz, 2H), 3.13 (q, *J* = 7.4 Hz, 4H), 1.41 (t, *J* = 7.4 Hz, 6H), 1.06 (s, 9H). ^{13}C NMR (125 MHz, $CDCl_3$) δ 171.2, 169.6, 135.0, 129.7, 129.5, 128.6, 128.6, 128.5, 128.2, 67.3, 65.6, 52.2, 49.4, 41.2, 34.8, 26.5, 7.1. HRMS

(ESI) [M+Na]$^+$ m/z calcd for $C_{30}H_{40}N_2O_9S_2Na$ 691.1794, found 691.1799.

Compound 3df(mono)

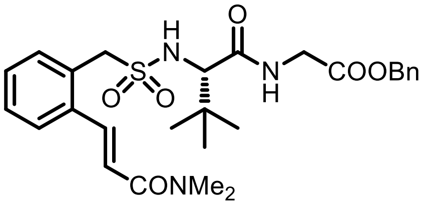

According to the general procedure, the crude residue was purified by flash column chromatography on silica gel (petroleum ether: ethyl acetate= 3:1; R_f= 0.33) to produce compound **3df(mono)** (9 mg, 27% yield).^{1}H NMR (500 MHz, $CDCl_3$) δ 9.33 (d, *J* = 5.4 Hz, 1H), 8.13 (d, *J* = 15.3 Hz, 1H), 7.58 (dd, *J* = 5.6, 3.3 Hz, 1H), 7.41 (m, 3H), 7.32 (m, 3H), 7.26 (m, 2H), 6.81 (d, *J* = 15.3 Hz, 1H), 5.44 (d, *J* = 9.9 Hz, 1H), 5.19 – 5.02 (m, 2H), 4.90 (d, *J* = 14.1 Hz, 1H), 4.37 (dd, *J* = 17.5, 6.6 Hz, 1H), 4.18 (d, *J* = 14.1 Hz, 1H), 3.90 (dd, *J* = 17.5, 5.2 Hz, 1H), 3.78 (d, *J* = 10.0 Hz, 1H), 3.23 (s, 3H), 3.08 (s, 3H), 1.06 (s, 9H). ^{13}C NMR (125 MHz, $CDCl_3$) δ 171.7, 169.6, 167.1, 141.2, 136.4, 135.2, 131.6, 129.7, 129.0, 129.0, 128.5, 128.4, 128.2, 126.8, 119.5, 67.0, 65.1, 60.4, 56.8, 41.4, 37.5, 36.1, 34.8, 26.4, 14.2. HRMS (ESI) [M+Na]$^+$ m/z calcd for $C_{27}H_{35}N_3O_6SNa$ 552.2144, found 552.2153.

Compound 3df(di)

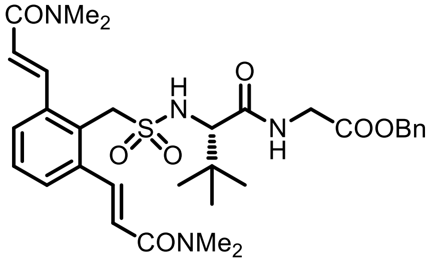

According to the general procedure, the crude residue was purified by flash column chromatography on silica gel (petroleum ether: ethyl acetate= 3:1; R_f= 0.38) to produce compound **3df(di)**(14 mg, 44% yield).^{1}H NMR (500 MHz, $CDCl_3$) δ 9.36 (t, *J* = 5.9 Hz, 1H), 8.06 (s, 2H), 7.54 (d, *J* = 7.6 Hz, 2H), 7.37 (dd, *J* = 9.2, 5.9 Hz, 2H), 7.32 – 7.22 (m, 6H), 6.78 (d, *J* = 15.3 Hz, 2H), 5.48 (d, *J* = 10.1 Hz, 1H), 5.06 (s, 2H), 4.79 (d, *J* = 14.6 Hz, 1H), 4.53 (d, *J* = 14.6 Hz, 1H), 4.34 (dd, *J* = 17.5, 6.8 Hz, 1H), 3.84 (dd, *J* = 17.5, 5.2 Hz, 1H), 3.77 (d, *J* = 10.1 Hz, 1H), 3.18 (s, 6H), 3.06 (s, 6H), 1.03 (s, 9H). ^{13}C NMR (125 MHz, $CDCl_3$) δ 171.7, 169.7, 135.2, 128.8, 128.5, 128.4, 128.2, 127.6, 67.0, 64.9, 53.0, 41.4, 37.6, 36.0, 34.7, 26.4. HRMS (ESI) [M+Na]$^+$ m/z calcd for $C_{32}H_{42}N_4O_7SNa$ 649.2672, found 649.2688.

Compound 3dg(mono)

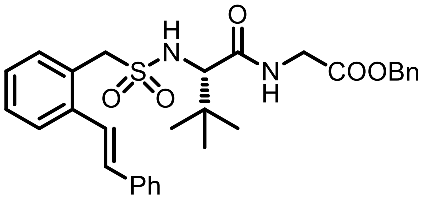

According to the general procedure, the crude residue was purified by flash column chromatography on silica gel (petroleum ether: ethyl acetate= 3:1; R_f= 0.36) to produce compound **3dg(mono)** (6 mg, 21% yield). ^{1}H NMR (500 MHz, MeOD) δ 7.71 (d, *J* = 7.6 Hz, 1H), 7.63 (d, *J* = 16.1 Hz, 1H), 7.59 (d, *J* = 7.3 Hz, 2H), 7.41 – 7.32 (m, 5H), 7.28 – 7.22 (m, 7H), 7.07 (d, *J* = 16.1 Hz, 1H), 5.07 (q, *J* = 12.2 Hz, 2H), 4.51 (d, *J* = 12.4 Hz, 1H), 4.41 (d, *J* = 13.9 Hz, 1H), 4.30 (s, 1H), 3.97 (dd, *J* = 50.2, 17.6 Hz, 2H), 3.80 (s, 1H), 1.03 (s, 9H). ^{13}C NMR (125 MHz, MeOD) δ 172.0, 169.5, 138.3, 137.6, 135.6, 132.5, 131.2, 128.4, 128.3, 128.1, 127.9, 127.4, 127.3, 127.1, 126.5, 126.0, 125.7, 66.6, 65.0, 56.1, 40.5, 34.0, 25.7. HRMS (ESI) $[M+Na]^+$ m/z calcd for $C_{30}H_{34}N_2O_5SNa$ 557.2086, found 557.2082.

Compound 3dg(di)

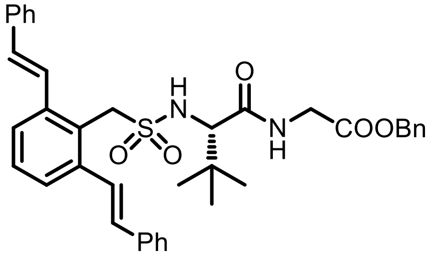

According to the general procedure, the crude residue was purified by flash column chromatography on silica gel (petroleum ether: ethyl acetate= 3:1; R_f= 0.42) to produce compound **3dg(di)** (20 mg, 62% yield). ^{1}H NMR (500 MHz, $CDCl_3$) δ 7.67 (d, *J* = 16.0 Hz, 2H), 7.56 – 7.49 (m, 6H), 7.37 (t, *J* = 7.6 Hz, 5H), 7.35 – 7.27 (m, 7H), 6.91 (d, *J* = 16.0 Hz, 2H), 5.49 (t, *J* = 5.6 Hz, 1H), 5.30 (d, *J* = 9.5 Hz, 1H), 5.08 (s, 2H), 4.55 (s, 2H), 3.83 (dd, *J* = 18.0, 6.3 Hz, 1H), 3.63 (dd, *J* = 18.0, 5.1 Hz, 1H), 3.52 (d, *J* = 9.5 Hz, 1H), 0.98 (s, 9H). ^{13}C NMR (125 MHz, $CDCl_3$) δ 170.5, 168.8, 139.9, 137.5, 133.0, 129.1, 128.9, 128.6, 128.5, 128.1, 127.8, 126.7, 126.5, 124.6, 67.3, 65.5, 54.2, 41.2, 34.8, 26.5. HRMS (ESI) $[M+Na]^+$ m/z calcd for $C_{38}H_{40}N_2O_5SNa$ 659.2556, found 659.2550.

Compound 3dh(mono)

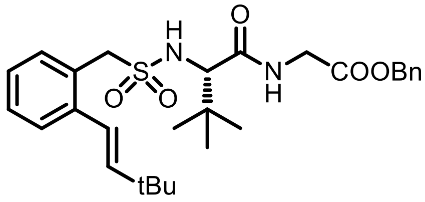

According to the general procedure, the crude residue was purified by flash column chromatography on silica gel (petroleum ether: ethyl acetate= 3:1; R_f= 0.41) to produce compound **3dh(mono)** (8 mg, 32% yield). ^{1}H NMR (500 MHz, $CDCl_3$) δ 7.41 – 7.30 (m, 5H), 7.25 – 7.20 (m, 1H), 6.93 (t, *J* = 8.8 Hz, 1H), 6.74 (d, *J* = 15.9 Hz, 1H), 6.16 (d, *J* = 15.9 Hz, 1H), 6.08 (t, *J* = 5.1 Hz, 1H), 5.25 – 5.10 (m, 3H), 4.41 (q, *J* = 14.2 Hz,

2H), 4.19 (dd, J = 18.3, 5.7 Hz, 1H), 4.02 (dd, J = 18.3, 4.9 Hz, 1H), 3.63 (d, J = 9.3 Hz, 1H), 1.14 (s, 9H), 1.02 (s,9H). ^{13}C NMR (125 MHz, CDCl$_3$) δ 170.4 169.1, 147.1, 141.7, 134.9, 130.1, 130.0, 128.7, 128.6, 122.3, 121.4, 113.6, 113.4, 67.5, 65.2, 50.4, 41.5, 34.8, 33.9, 29.5, 26.5. HRMS (ESI) [M+Na]$^+$ m/z calcd for $C_{28}H_{38}N_2O_5SNa$ 537.2399, found 537.2391.

Compound 3dh(di)

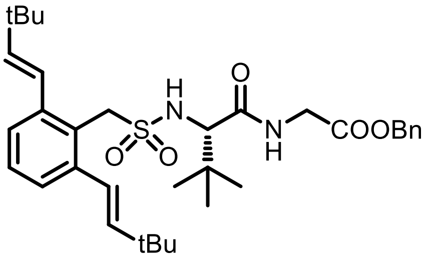

According to the general procedure, the crude residue was purified by flash column chromatography on silica gel (petroleum ether: ethyl acetate= 3:1; R_f= 0.52) to produce compound **3dh(di)** (10 mg, 32% yield).^{1}H NMR (500 MHz, CDCl$_3$) δ 7.38 – 7.32 (m, 5H), 7.29 (d, J = 7.7 Hz, 2H), 7.22 – 7.18 (m, 1H), 6.80 (d, J = 15.9 Hz, 2H), 6.04 (d, J = 15.9 Hz, 2H), 5.96 (t, J = 5.0 Hz, 1H), 5.27 – 5.12 (m, 3H), 4.45 (q, J = 14.1 Hz, 2H), 4.17 (dd, J = 18.4, 5.7 Hz, 1H), 3.93 (dd, J = 18.4, 4.7 Hz, 1H), 3.58 (d, J = 9.2 Hz, 1H), 1.14 (s, 18H), 1.02 (s, 9H). ^{13}C NMR (125 MHz, CDCl$_3$) δ 170.4, 169.1, 146.1, 139.5, 134.9, 131.8, 128.9, 128.7, 128.6, 126.9, 125.7, 122.0, 67.5, 65.3 57.0, 41.4, 34.8, 33.8, 29.5, 26.5. HRMS (ESI) [M+Na]$^+$ m/z calcd for $C_{34}H_{48}N_2O_5SNa$ 619.3182, found 619.3195.

Compound 3di

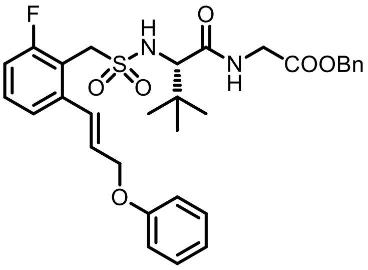

According to the general procedure, the crude residue was purified by flash column chromatography on silica gel (petroleum ether: ethyl acetate= 3:1; R_f = 0.52) to produce compound **3di** (10 mg, 72% yield).^{1}H NMR (400 MHz, CDCl$_3$) δ 7.38 – 7.24 (m, 9H), 7.15 (d, J = 15.8 Hz, 1H), 7.06 – 6.93 (m, 4H), 6.34 (dt, J = 15.8, 5.4 Hz, 1H), 6.11 (t, J = 5.4 Hz, 1H), 5.26 (d, J = 9.6 Hz, 1H), 5.15 (s, 2H), 4.75 (dd, J = 5.4, 1.5 Hz, 2H), 4.38 (q, J = 14.3 Hz, 2H), 4.05 (dd, J = 5.5, 2.0 Hz, 2H), 3.59 (d, J = 9.6 Hz, 1H), 1.01 (s, 9H). ^{13}C NMR (100 MHz, CDCl$_3$) δ 170.6, 169.1, 160.5, 158.6, 140.0, 135.0, 129.6, 128.7, 128.5, 122.6 121.2, 115.1, 114.8, 114.5, 68.7, 67.4, 65.2, 50.1, 41.4, 34.8, 26.5. HRMS (ESI) [M+Na]$^+$ m/z calcd for $C_{31}H_{35}FN_2O_6SNa$ 605.2098, found 605.2090.

Compound 3dj

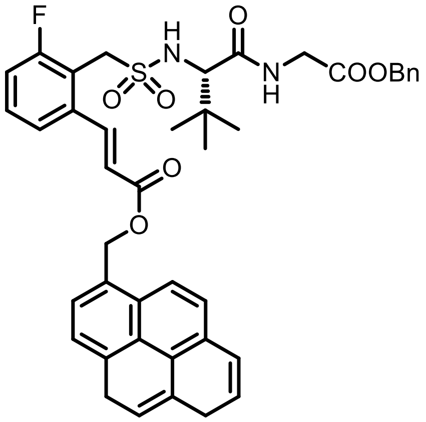

According to the general procedure, the crude residue was purified by flash column chromatography on silica gel (petroleum ether: ethyl acetate= 2:1; R_f= 0.52) to produce compound **3dj** (12 mg, 70% yield).^{1}H NMR (500 MHz, $CDCl_3$) δ 8.36 (d, *J* = 9.2 Hz, 1H), 8.30 – 8.02 (m, 8H), 7.56 – 7.29 (m, 8H), 7.27 – 7.07 (m, 3H), 6.52 (d, *J* = 15.8 Hz, 1H), 5.99 (s, 2H), 5.46 (d, *J* = 10.0 Hz, 1H), 5.21 (q, *J* = 12.1 Hz, 1H), 5.04 (s, 2H), 4.59 (dd, *J* = 44.6, 14.3 Hz, 2H), 4.33 (dd, *J* = 41.1, 14.0 Hz, 1H), 4.24 (dd, *J* = 17.7, 6.5 Hz, 1H), 3.77 (dd, *J* = 17.7, 5.3 Hz, 1H), 3.72 (d, *J* = 10.0 Hz, 1H), 1.04 (s, 9H). ^{13}C NMR (125 MHz, $CDCl_3$) δ 171.2, 169.3, 168.2, 162.6, 160.6, 142.6, 136.9, 135.0, 131.9, 131.2, 130.7, 130.2, 129.6, 128.6, 128.5, 128.3, 128.1, 127.8, 127.3, 126.3, 125.7, 125.6, 124.9 124.6, 122.8, 120.2, 117.3, 117.1, 67.2, 65.5, 49.6, 41.3, 34.7, 26.4. HRMS (ESI) $[M+Na]^+$ m/z calcd for $C_{42}H_{41}FN_2O_7SNa$ 759.2516, found 759.2510.

Compound 1k

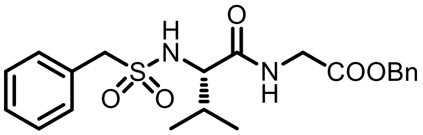

According to the general procedure, the crude residue was purified by flash column chromatography on silica gel (petroleum ether: ethyl acetate= 3:1; R_f= 0.40) to produce compound **1k**(0.82 g, 92% yield).^{1}H NMR (400 MHz, $CDCl_3$) δ 7.44 – 7.28 (m, 9H), 6.30 (d, *J* = 4.7 Hz, 1H), 5.22 – 5.14 (m, 2H), 5.13 – 5.07 (m, 1H), 4.26 (q, *J* = 13.9 Hz, 2H), 4.05 (d, *J* = 5.4 Hz, 2H), 3.67 (dd, *J* = 8.0, 5.6 Hz, 1H), 2.00 (td, *J* = 13.6, 6.9 Hz, 1H), 0.97 (d, *J* = 6.8 Hz, 3H), 0.90 (d, *J* = 6.8 Hz, 3H).^{13}C NMR (100 MHz, $CDCl_3$) δ 171.1, 169.4, 135.0, 130.9, 128.8, 128.7 128.5, 67.4, 62.5, 59.8, 41.4, 32.0, 19.1, 17.6. HRMS (ESI) $[M+Na]^+$ m/z calcd for $C_{21}H_{26}N_2O_5SNa$ 441.1460, found 441.1475.

Compound 3ka(mono)

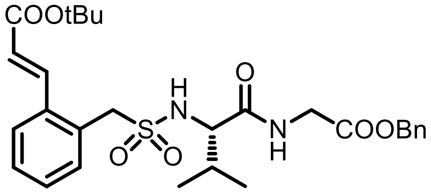

According to the general procedure, the crude residue was purified by flash column chromatography on silica gel (petroleum ether: ethyl acetate= 3:1; R_f= 0.46) to produce compound **3ka(mono)** (10 mg, 27% yield).^{1}H NMR (400 MHz, $CDCl_3$) δ 8.01 (d, *J* = 15.8 Hz, 1H), 7.66 – 7.58 (m, 2H), 7.44 – 7.24 (m, 7H), 6.32 (d, *J* = 15.8 Hz, 1H), 5.34 (d, *J* = 9.5 Hz, 1H), 5.10 (q, *J* = 12.2 Hz, 2H), 4.74 (d, *J* = 14.0 Hz, 1H), 4.29 – 4.18 (m, 2H), 3.96 (dd, *J* = 17.7, 5.5 Hz, 1H), 3.77 (dd, *J* = 9.4, 6.7 Hz, 1H), 2.03 (dq, *J* = 13.5, 6.7 Hz, 1H), 1.51 (s, 9H), 1.00 (dd, *J* = 6.8, 2.8 Hz, 6H). ^{13}C NMR (100 MHz, $CDCl_3$) δ 172.0 169.4, 167.5, 141.6, 135.2, 132.2, 130.1,

129.1, 128.7, 128.6, 128.5, 128.4, 126.9, 121.8, 81.3, 67.2, 62.9, 56.8, 41.4, 32.2, 28.2, 19.1, 18.3. HRMS (ESI) $[M+Na]^+$ m/z calcd for $C_{28}H_{36}N_2O_7SNa$ 567.2141, found 567.2132.

Compound 3ka(di)

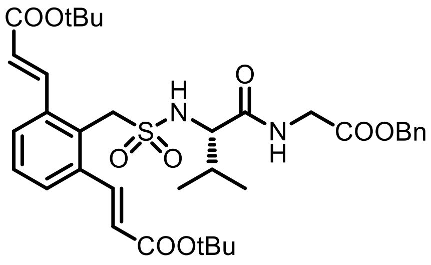

According to the general procedure, the crude residue was purified by flash column chromatography on silica gel (petroleum ether: ethyl acetate= 3:1; R_f= 0.53) to produce compound **3ka(di)** (23 mg, 61% yield).^{1}H NMR (400 MHz, $CDCl_3$) δ 8.19 (t, *J* = 6.0 Hz, 1H), 8.12 (d, *J* = 15.7 Hz, 2H), 7.59 (d, *J* = 7.8 Hz, 2H), 7.41 – 7.33 (m, 3H), 7.31 – 7.27 (m, 2H), 7.25 – 7.21 (m, 1H), 6.29 (d, *J* = 15.7 Hz, 2H), 5.41 (d, *J* = 9.9 Hz, 1H), 5.07 (dd, *J* = 31.8, 12.2 Hz, 2H), 4.76 (d, *J* = 14.6 Hz, 1H), 4.51 (d, *J* = 14.7 Hz, 1H), 4.33 (dd, *J* = 17.6, 6.8 Hz, 1H), 3.86 (dd, *J* = 17.6, 5.3 Hz, 1H), 3.79 (dd, *J* = 9.9, 7.1 Hz, 1H), 2.01 (dq, *J* = 13.5, 6.8 Hz, 1H), 1.53 (s, 18H), 1.03 (dd, *J* = 12.0, 6.7 Hz, 6H). ^{13}C NMR (100 MHz, $CDCl_3$) δ 172.3, 169.4, 136.7, 135.1, 129.0, 128.6, 128.5, 128.3, 127.8, 81.3, 67.2, 63.0, 52.7, 41.4, 32.4, 28.2, 19.1, 18.5. HRMS (ESI) $[M+Na]^+$ m/z calcd for $C_{35}H_{46}N_2O_9SNa$ 693.2822, found 693.2820.

Compound 1l

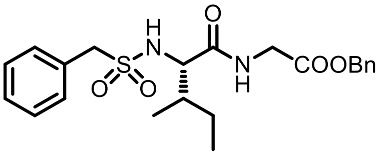

According to the general procedure, the crude residue was purified by flash column chromatography on silica gel (petroleum ether: ethyl acetate= 3:1; R_f = 0.31) to produce compound **1l** (0.78 g, 90% yield).^{1}H NMR (400 MHz, $CDCl_3$) δ 7.40 (m, 2H), 7.33 (m, 7H), 6.36 (s, 1H), 5.21 – 5.13 (m, 2H), 5.12 (s, 1H), 4.26 (q, *J* = 13.9 Hz, 2H), 4.05 (d, *J* = 5.3 Hz, 2H), 3.75 – 3.69 (m, 1H), 1.78 – 1.67 (m, 1H), 1.55 – 1.43 (m, 1H), 1.12 (ddd, *J* = 13.5, 9.5, 7.3 Hz, 1H), 0.92 (d, *J* = 6.8 Hz, 3H), 0.86 (t, *J* = 7.4 Hz, 3H). ^{13}C NMR (100 MHz, $CDCl_3$) δ 171.2, 169.4, 135.0, 130.9, 128.9, 128.7, 128.5, 67.4, 61.8, 59.7, 41.4, 38.6, 24.7, 15.3, 11.4. HRMS (ESI) $[M+Na]^+$ m/z calcd for $C_{22}H_{28}N_2O_5SNa$ 455.1617, found 455.1616.

Compound 3la(mono)

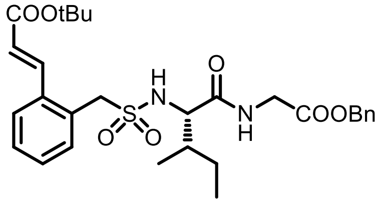

According to the general procedure, the crude residue was purified by flash column chromatography on silica gel (petroleum ether: ethyl acetate= 3:1; R_f = 0.37) to produce compound **3la(mono)** (9 mg, 35% yield).^{1}H NMR (400 MHz, $CDCl_3$) δ 8.02 (d, *J* = 15.8 Hz, 1H), 7.70 (t, *J* = 5.8 Hz, 1H), 7.65 – 7.59 (m, 1H), 7.45 – 7.24 (m, 7H), 6.33 (d, *J* = 15.8 Hz, 1H), 5.33 (d, *J* = 9.5 Hz, 1H), 5.10 (q, *J* = 12.2 Hz, 2H), 4.78 (d, *J* = 14.0 Hz, 1H), 4.31 – 4.18 (m, 2H), 3.94 (dd, *J* = 17.7, 5.5 Hz, 1H), 3.82 (dd, *J* = 9.5, 7.2 Hz, 1H), 1.84 – 1.72 (m, 1H), 1.66 (ddd, *J* = 14.8, 7.4, 3.8 Hz, 1H), 1.51 (s, 9H), 1.31 – 1.15 (m, 1H), 0.96 (d, *J* = 6.7 Hz, 3H), 0.89 (t, *J* = 7.4 Hz, 3H). ^{13}C NMR (100 MHz, $CDCl_3$) δ 172.0, 169.4, 167.6, 141.6, 135.1, 132.2, 130.1, 129.1, 128.8, 128.6, 128.5, 128.4, 126.9, 121.7, 81.3, 67.2, 61.9, 56.8, 41.4, 38.6, 28.1, 24.9, 15.3, 11.0. HRMS (ESI) $[M+Na]^+$ m/z calcd for $C_{29}H_{38}N_2O_7SNa$ 581.2297, found 581.2295.

Compound 3la(di)

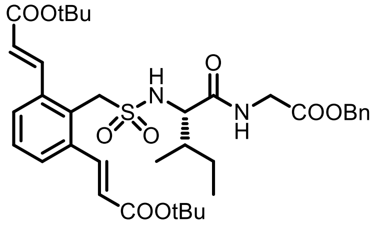

According to the general procedure, the crude residue was purified by flash column chromatography on silica gel (petroleum ether: ethyl acetate= 3:1; R_f = 0.42) to produce compound **3la(di)**(20 mg, 59% yield).^{1}H NMR (400 MHz, $CDCl_3$) δ 8.25 (t, *J* = 6.0 Hz, 1H), 8.12 (d, *J* = 15.7 Hz, 2H), 7.60 (d, *J* = 7.8 Hz, 2H), 7.41 – 7.19 (m, 6H), 6.29 (d, *J* = 15.7 Hz, 2H), 5.39 (d, *J* = 9.9 Hz, 1H), 5.07 (dd, *J* = 31.2, 12.2 Hz, 2H), 4.80 (d, *J* = 14.6 Hz, 1H), 4.52 (d, *J* = 14.7 Hz, 1H), 4.34 (dd, *J* = 17.5, 6.8 Hz, 1H), 3.90 – 3.80 (m, 2H), 1.78 – 1.68 (m, 2H), 1.53 (s, 18H), 1.24 (m, 1H), 0.98 (d, *J* = 6.7 Hz, 3H), 0.90 (t, *J* = 7.4 Hz, 3H). ^{13}C NMR (100 MHz, $CDCl_3$) δ 172.3, 169.4, 136.6, 135.1, 129.0, 128.6, 128.5, 128.3, 127.8, 81.3, 67.2, 61.9, 60.4, 52.7, 41.4, 38.6, 28.1, 24.9, 21.1, 15.3, 14.2, 10.9. HRMS (ESI) $[M+Na]^+$ m/z calcd for $C_{36}H_{48}N_2O_9SNa$ 707.2978, found 707.2976.

Compound 1m

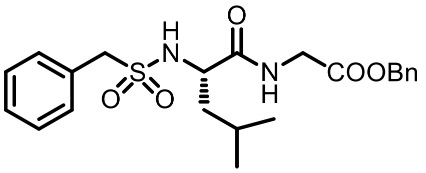

According to the general procedure, the crude residue was purified by flash column chromatography on silica gel (petroleum ether: ethyl acetate= 3:1; R_f= 0.34) to produce compound **1m** (1.21 g, 92% yield).^{1}H NMR (400 MHz, $CDCl_3$) δ 7.44 – 7.39 (m, 2H), 7.38 – 7.29 (m, 7H), 6.64 (t, *J* = 5.3 Hz, 1H), 5.27 (d, *J* = 8.6 Hz, 1H), 5.20 – 5.10 (m, 2H), 4.32 – 4.21 (m, 2H), 4.10 – 3.94 (m, 2H), 3.88 (td, *J* = 8.6, 6.2 Hz, 1H), 1.70 (dt, *J* = 13.5, 6.6 Hz, 1H), 1.58 – 1.41 (m, 2H), 0.87 (dd, *J* = 10.9, 6.6 Hz, 6H). ^{13}C NMR (100 MHz, $CDCl_3$) δ 172.5, 169.6, 135.0, 130.9, 129.0, 128.7, 128.5, 67.4, 59.7, 55.9, 42.6, 41.4, 24.3, 22.7, 22.0.HRMS (ESI) $[M+Na]^+$ m/z calcd for $C_{29}H_{38}N_2O_7SNa$ 581.2297, found 581.2294.

Compound 3ma(mono)

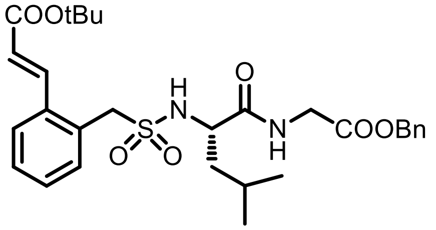

According to the general procedure, the crude residue was purified by flash column chromatography on silica gel (petroleum ether: ethyl acetate= 3:1; R_f= 0.40) to produce compound **3ma(mono)** (12 mg, 27% yield).^{1}H NMR (400 MHz, $CDCl_3$) δ 8.02 (d, *J* = 15.8 Hz, 1H), 7.73 (t, *J* = 5.8 Hz, 1H), 7.65 – 7.61 (m, 1H), 7.45 – 7.26 (m, 9H), 6.33 (d, *J* = 15.8 Hz, 1H), 5.17 (d, *J* = 7.7 Hz, 1H), 5.10 (dd, *J* = 25.4, 12.2 Hz, 2H), 4.79 (d, *J* = 14.1 Hz, 1H), 4.29 – 4.21 (m, 2H), 4.09 – 4.02 (m, 1H), 3.93 (dd, *J* = 17.7, 5.4 Hz, 1H), 1.80 (dt, *J* = 13.1, 6.5 Hz, 1H), 1.63 – 1.53 (m, 2H), 1.50 (s, 9H), 0.92 (t, *J* = 6.2 Hz, 6H). ^{13}C NMR (100 MHz, $CDCl_3$) δ 173.0, 169.3, 167.6, 141.6, 135.1, 132.2 130.9, 130.1, 129.1, 128.8, 128.6, 128.5, 128.4, 126.9, 121.6, 81.3, 67.2, 56.8, 55.9, 43.1, 41.5, 28.1, 24.3, 22.6, 21.8.HRMS (ESI) $[M+Na]^+$ m/z calcd for $C_{29}H_{38}N_2O_7SNa$ 581.2297, found 581.2292.

Compound 3ma(di)

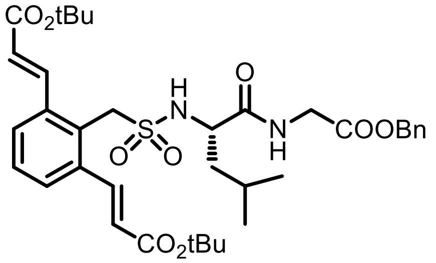

According to the general procedure, the crude residue was purified by flash column chromatography on silica gel (petroleum ether: ethyl acetate= 3:1; R_f= 0.51) to produce compound **3ma(di)** (23 mg, 61% yield).^{1}H NMR (400 MHz, $CDCl_3$) δ 8.29 (t, *J* = 6.0 Hz, 1H), 8.13 (d, *J* = 15.8 Hz, 2H), 7.61 (d, *J* = 7.8 Hz, 2H), 7.37 (t, *J* = 7.8 Hz, 1H), 7.32 – 7.20 (m, 4H), 6.29 (d, *J* = 15.7 Hz, 2H), 5.34 (d, *J* = 9.9 Hz, 1H), 5.06 (dd, *J* = 33.0, 12.2 Hz, 2H), 4.81 (d, *J* = 14.6 Hz, 1H), 4.51 (d, *J* = 14.7 Hz, 1H), 4.33 (dd, *J* = 17.6, 6.8 Hz, 1H), 4.19 – 4.08 (m, 1H), 3.84 (dd, *J* = 17.6, 5.3 Hz, 1H), 1.91 – 1.78 (m, 1H), 1.74 – 1.61 (m, 1H), 1.61 – 1.55 (m, 1H), 1.52 (s, 18H), 0.94 (dd, *J* = 12.1, 6.6 Hz,6H). ^{13}C NMR (100 MHz, $CDCl_3$) δ 173.4, 169.4,

136.6, 135.1, 129.0, 128.6, 128.3, 127.8, 81.3, 67.2, 55.9, 52.6, 43.4, 41.5, 28.2, 24.3, 22.6, 21.8.HRMS (ESI) $[M+Na]^+$ m/z calcd for $C_{36}H_{48}N_2O_9SNa$ 707.2978, found 707.2970.

Compound 1n

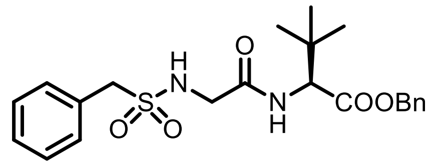

According to the general procedure, the crude residue was purified by flash column chromatography on silica gel (petroleum ether: ethyl acetate= 3:1; R_f= 0.32) to produce compound **1n** (0.95 g, 90% yield).^{1}H NMR (400 MHz, $CDCl_3$) δ 7.45 – 7.29 (m, 9H), 6.60 (s, 1H), 5.15 (q, *J* = 12.2 Hz, 2H), 5.04 (s, 1H), 4.46 (d, *J* = 9.3 Hz, 1H), 4.31 (s, 2H), 3.68 – 3.52 (m, 2H), 0.95 (s, 9H).

^{13}C NMR (100 MHz, $CDCl_3$) δ 171.0, 167.7, 135.0, 130.7, 129.0, 128.7, 128.6, 128.5, 67.1, 60.3, 59.2, 46.3, 35.0, 26.5.HRMS (ESI) $[M+Na]^+$ m/z calcd for $C_{29}H_{38}N_2O_7SNa$ 581.2297, found 581.2292.

Compound 3na(mono)

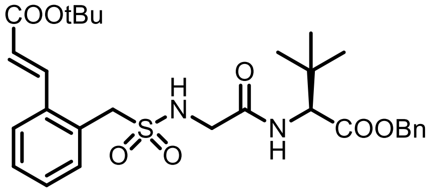

According to the general procedure, the crude residue was purified by flash column chromatography on silica gel (petroleum ether: ethyl acetate= 3:1; R_f= 0.40) to produce compound **3na1** (19 mg, 44% yield).^{1}H NMR (500 MHz, $CDCl_3$) δ 7.96 (d, *J* = 15.7 Hz, 1H), 7.69 – 7.63 (m, 1H), 7.48 – 7.44 (m, 1H), 7.44 – 7.39 (m, 2H), 7.39 – 7.32 (m, 5H), 6.77 (d, *J* = 9.2 Hz, 1H), 6.37 (d, *J* = 15.7 Hz, 1H), 5.30 (t, *J* = 5.7 Hz, 1H), 5.21 – 5.11 (m, 2H), 4.53 – 4.43 (m, 3H), 3.69 – 3.61 (m, 2H), 1.55 (s, 9H), 0.97 (s, 9H). ^{13}C NMR (125 MHz, $CDCl_3$) δ 170.9, 167.9, 165.9, 139.9, 135.1, 132.6, 130.1, 129.5, 128.6, 128.5, 127.9, 127.2, 123.3, 81.1, 67.0, 60.4, 56.2, 46.3, 34.9, 28.2, 26.5. HRMS (ESI) $[M+Na]^+$ m/z calcd for $C_{29}H_{38}N_2O_7SNa$ 581.2297, found 581.2291.

Compound 3na(di)

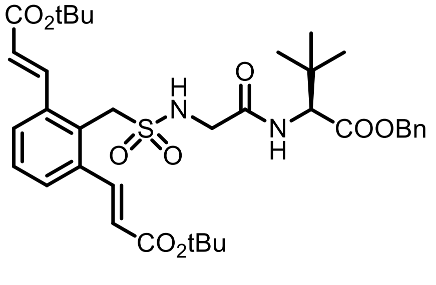

According to the general procedure, the crude residue was purified by flash column chromatography on silica gel (petroleum ether: ethyl acetate= 3:1; R_f= 0.48) to produce compound **3na(di)** (26 mg, 11% yield).^{1}H NMR (500 MHz,

$CDCl_3$) δ 8.04 (d, *J* = 15.7 Hz, 2H), 7.61 (d, *J* = 7.8 Hz, 2H), 7.39 (t, *J* = 7.8 Hz, 1H), 7.34 – 7.29 (m, 5H), 7.09 (d, *J* = 8.9 Hz, 1H), 6.32 (d, *J* = 15.7 Hz, 2H), 5.27 (t, *J* = 5.4 Hz, 1H), 5.12 (q, *J* = 12.2 Hz, 2H), 4.60 – 4.53 (m, 2H), 4.44 (d, *J* = 8.9 Hz, 1H), 3.78 (qd, *J* = 17.0, 5.4 Hz, 2H), 1.54 (s, 18H), 0.97 (s, 9H). ^{13}C NMR (125 MHz, $CDCl_3$) δ 170.9, 168.2, 165.9, 140.6, 136.7, 129.4, 128.5, 128.4, 124.4, 81.2, 67.0, 60.9, 52.3, 46.0, 34.6, 28.2, 26.7. HRMS (ESI) $[M+Na]^+$ m/z calcd for $C_{36}H_{48}N_2O_9SNa$ 707.2978, found 707.2977.

Compound 1o

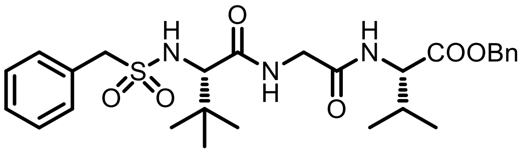

According to the general procedure, the crude residue was purified by flash column chromatography on silica gel (petroleum ether: ethyl acetate= 2:1; R_f = 0.33) to produce compound **1o** (1.12 g, 96% yield). ^{1}H NMR (400 MHz, $CDCl_3$) δ 7.60 (d, *J* = 8.6 Hz, 1H), 7.41 – 7.29 (m, 6H), 7.28 – 7.21 (m, 3H), 6.90 (s, 1H), 6.03 (d, *J* = 9.4 Hz, 1H), 5.14 (dd, *J* = 44.2, 12.2 Hz, 2H), 4.66 (dd, *J* = 8.6, 4.8 Hz, 1H), 4.30 – 4.20 (m, 2H), 4.16 (dd, *J* = 9.7, 4.6 Hz, 2H), 3.66 (d, *J* = 9.5 Hz, 1H), 2.22 – 2.11 (m, 1H), 0.97 (s, 9H), 0.86 (dd, *J* = 17.2, 6.9 Hz, 6H). ^{13}C NMR (100 MHz, $CDCl_3$) δ 171.9, 170.9, 168.1, 135.2, 130.8, 129.0, 128.6, 128.5, 67.4, 65.2, 59.5, 57.3, 43.3, 34.8, 31.6, 26.6, 18.9, 17.7. HRMS (ESI) $[M+Na]^+$ m/z calcd for $C_{27}H_{37}N_3O_6SNa$ 554.2301, found 554.2309.

Compound 3oa(mono)

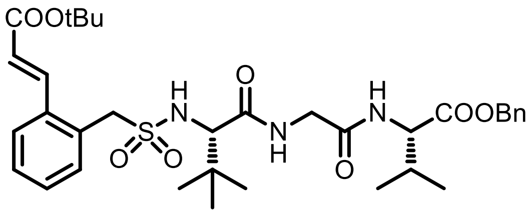

According to the general procedure, the crude residue was purified by flash column chromatography on silica gel (petroleum ether: ethyl acetate= 2:1; R_f = 0.42) to produce compound **3oa(mono)** (11 mg, 28% yield). ^{1}H NMR (400 MHz, $CDCl_3$) δ 7.98 (d, *J* = 15.8 Hz, 1H), 7.69 (t, *J* = 5.5 Hz, 1H), 7.62 – 7.54 (m, 1H), 7.45 – 7.38 (m, 1H), 7.38 – 7.27 (m, 7H), 6.74 (d, *J* = 8.6 Hz, 1H), 6.32 (d, *J* = 15.8 Hz, 1H), 5.51 (d, *J* = 9.9 Hz, 1H), 5.07 (dd, *J* = 32.4, 12.2 Hz, 2H), 4.59 (d, *J* = 14.1 Hz, 1H), 4.53 (dd, *J* = 8.6, 4.9 Hz, 1H), 4.35 (d, *J* = 14.1 Hz, 1H), 4.17 (dd, *J* = 16.2, 6.1 Hz, 1H), 3.88 (dd, *J* = 16.2, 5.2 Hz, 1H), 3.70 (d, *J* = 9.9 Hz, 1H), 2.18 – 2.05 (m, 1H), 1.52 (s, 9H), 1.04 (s, 9H), 0.83 (dd, *J* = 13.0, 6.9 Hz, 6H). ^{13}C NMR (100 MHz, $CDCl_3$) δ 171.6, 168.3, 167.4, 141.3, 135.3, 132.1, 130.0, 129.1, 128.6, 128.4, 126.9, 122.0, 81.3, 67.0, 65.5, 57.3, 56.5, 43.4, 34.8, 31.2, 28.2, 26.5, 18.8, 17.7. HRMS (ESI) $[M+Na]^+$ m/z calcd for $C_{34}H_{47}N_3O_8SNa$ 680.2982, found 680.2980.

Compound 3oa(di)

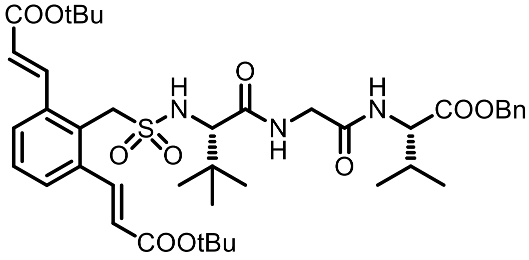

According to the general procedure, the crude residue was purified by flash column chromatography on silica gel (petroleum ether: ethyl acetate= 2:1; R_f = 0.49) to produce compound **3oa(di)** (20 mg, 36% yield).^{1}H NMR (400 MHz, $CDCl_3$) δ 8.33 (t, *J* = 6.0 Hz, 1H), 8.13 (d, *J* = 15.7 Hz, 2H), 7.58 (d, *J* = 7.8 Hz, 2H), 7.37 – 7.29 (m, 4H), 7.24 (m, 2H), 6.58 (d, *J* = 8.7 Hz, 1H), 6.29 (d, *J* = 15.7 Hz, 2H), 5.46 (d, *J* = 10.2 Hz, 1H), 5.05 (d, *J* = 12.2 Hz, 1H), 4.94 (d, *J* = 12.2 Hz, 1H), 4.64 (q, *J* = 14.7 Hz, 2H), 4.46 (dd, *J* = 8.6, 4.9 Hz, 1H), 4.22 (dd, *J* = 15.8, 6.7 Hz, 1H), 3.79 – 3.69 (m, 2H), 2.13 – 2.03 (m, 1H), 1.53 (s, 18H), 1.07 (s, 9H), 0.80 (dd, *J* = 9.0, 6.9 Hz, 6H). ^{13}C NMR (125 MHz, $CDCl_3$) δ 172.0, 171.4, 168.4, 136.6, 135.2, 129.0, 128.6, 128.4, 128.3, 127.6, 100.0, 66.9, 65.5, 57.2, 52.6, 43.6, 34., 31.1, 28.2, 26.5, 18.8, 17.7. HRMS (ESI) $[M+Na]^+$ m/z calcd for $C_{41}H_{57}N_3O_{10}SNa$ 806.3662, found 806.3658.

Compound 1p

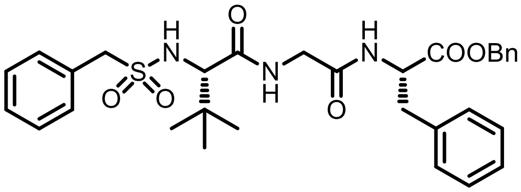

According to the general procedure, the crude residue was purified by flash column chromatography on silica gel (petroleum ether: ethyl acetate= 2:1; R_f = 0.28) to produce compound **1p** (1.31 g, 92% yield).^{1}H NMR (400 MHz, $CDCl_3$) δ 7.61 (d, *J* = 7.6 Hz, 1H), 7.31 (dt, *J* = 9.5, 3.1 Hz, 5H), 7.23 (ddd, *J* = 8.9, 7.3, 3.8 Hz, 6H), 7.17 – 7.09 (m, 3H), 6.98 (dd, *J* = 6.6, 2.9 Hz, 2H), 6.16 (d, *J* = 9.6 Hz, 1H), 5.03 (dd, *J* = 43.9, 12.1 Hz, 2H), 4.92 (dd, *J* = 13.9, 6.3 Hz, 1H), 4.19 (s, 2H), 4.17 – 4.03 (m, 2H), 3.77 (d, *J* = 9.6 Hz, 1H), 3.05 (ddd, *J* = 29.7, 13.8, 6.2 Hz, 2H), 0.92 (s, 9H). ^{13}C NMR (100 MHz, $CDCl_3$) δ 171.4, 171.0, 168.1, 135.7, 135.0, 130.9, 129.3, 129.2, 128.7, 128.6, 128.5, 127.0, 67.5, 64.8, 60.5, 59.4, 53.7, 43.1, 38.1, 34.8, 26.7, 21.1, 14.3. HRMS (ESI) $[M+Na]^+$ m/z calcd for $C_{31}H_{37}N_3O_6SNa$ 602.2301, found 602.2305.

Compound 3pa(mono)

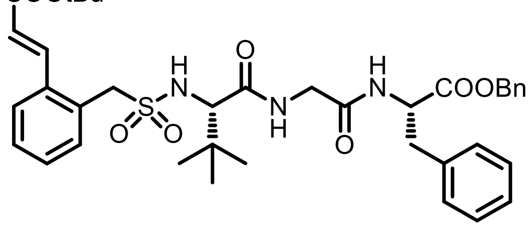

According to the general procedure, the crude residue was purified by flash column chromatography on silica gel (petroleum ether: ethyl acetate= 2:1; R_f = 0.34) to produce compound **3pa(mono)** (19 mg, 36% yield).^{1}H NMR (400 MHz, $CDCl_3$) δ 7.99 (d, *J* = 15.8 Hz, 1H), 7.76 (t, *J* = 5.4 Hz, 1H), 7.59 (dd, *J* = 5.9, 3.1 Hz, 1H), 7.43 – 7.27 (m, 7H), 7.24 – 7.12 (m, 5H), 6.94 (dd, *J* = 7.6, 1.6 Hz, 2H), 6.69 (d, *J* = 7.5 Hz, 1H), 6.33 (d, *J* = 15.8 Hz, 1H), 5.45 (d, *J* = 9.9

Hz, 1H), 4.99 (dd, J = 34.5, 12.1 Hz, 2H), 4.82 (dd, J = 13.4, 5.9 Hz, 1H), 4.54 (d, J = 14.1 Hz, 1H), 4.16 (m, 2H), 3.77 (dd, J = 16.3, 5.1 Hz, 1H), 3.68 (d, J = 9.9 Hz, 1H), 3.02 (d, J = 5.9 Hz, 2H), 1.53 (s, 9H), 1.01 (s, 9H). ^{13}C NMR (100 MHz, $CDCl_3$) δ 171.5, 171.0, 168.0, 167.6, 141.5, 135.5, 135.2, 135.0, 132.0, 130.0, 129.4, 129.1, 128.7, 128.6, 128.5, 127.1, 126.9, 122.0, 81.4, 67.2, 65.4, 56.4, 53.4, 43.2, 37.7, 34.7, 28.2, 26.5, 14.2. HRMS (ESI) $[M+Na]^+$ m/z calcd for $C_{38}H_{47}N_3O_8SNa$ 728.2982, found 728.2980.

Compound 3pa(di)

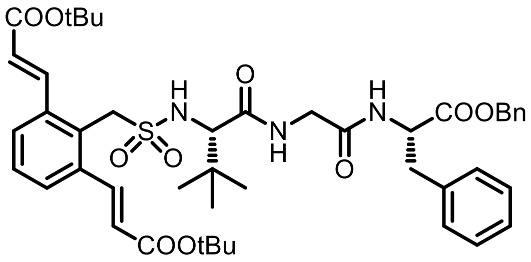

According to the general procedure, the crude residue was purified by flash column chromatography on silica gel (petroleum ether: ethyl acetate= 2:1; R_f = 0.39) to produce compound **3pa(di)** (11 mg, 21% yield)^{1}H NMR (500 MHz, $CDCl_3$) δ 8.39 (t, J = 6.0 Hz, 1H), 8.11 (d, J = 15.8 Hz, 2H), 7.59 (d, J = 7.8 Hz, 2H), 7.38 – 7.27 (m, 5H), 7.23 – 7.12 (m, 4H), 6.91 (d, J = 7.2 Hz, 2H), 6.54 (d, J = 7.7 Hz, 1H), 6.31 (d, J = 15.7 Hz, 2H), 5.37 (d, J = 10.1 Hz, 1H), 4.91 (dd, J = 57.8, 12.0 Hz, 2H), 4.77 (dd, J = 13.3, 5.7 Hz, 1H), 4.44 (dd, J = 106.0, 14.8 Hz, 2H), 4.22 (dd, J = 16.0, 6.7 Hz, 1H), 3.71 (d, J = 10.1 Hz, 1H), 3.63 (dd, J = 15.9, 5.4 Hz, 1H), 2.99 (d, J = 5.6 Hz, 2H), 1.54 (s, 18H), 1.04 (s, 9H).^{13}C NMR (125 MHz, $CDCl_3$) δ 171.8, 170.7, 168.1, 136.6, 135.4, 134.9, 129.5, 128.9, 128.7, 128.6, 128.5, 128.3, 127.9, 127.2, 67.1, 65.3, 53.2, 52.6, 43.5, 37.5, 34.8, 28.2, 26.4. HRMS (ESI) $[M+Na]^+$ m/z calcd for $C_{45}H_{57}N_3O_{10}SNa$ 854.3662, found 854.3659.

Compound 3q

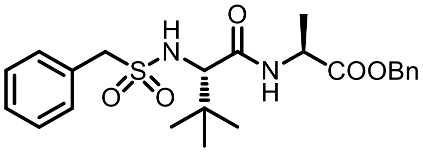

According to the general procedure, the crude residue was purified by flash column chromatography on silica gel (petroleum ether: ethyl acetate= 3:1; R_f = 0.39) to produce compound **3d** (1.1 g, 91% yield)^{1}H NMR (500 MHz, $CDCl_3$) δ 7.41 – 7.23 (m, 10H), 6.38 (s, 1H), 5.17 (s, 2H), 5.13 (d, J = 11.8 Hz, 1H), 4.61 (p, J = 7.2 Hz, 1H), 4.23 (q, J = 13.9 Hz, 2H), 3.62 (dd, J = 9.3, 2.2 Hz, 1H), 1.44 (d, J = 7.2 Hz, 3H), 1.00 (s, 9H).^{13}C NMR (125MHz, $CDCl_3$) δ 172.4, 169.7, 135.1, 130.9, 128.7, 128.6, 128.3, 67.5, 65.2, 59.5, 48.4, 34.8, 26.6, 18.0. HRMS (ESI) $[M+Na]^+$ m/z calcd for $C_{23}H_{30}N_2O_5SNa$ 469.1773, found 469.1772.

Compound 3qa(mono)

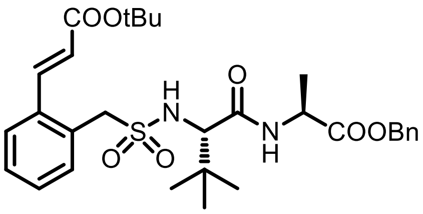

According to the general procedure, the crude residue was purified by flash column chromatography on silica gel (petroleum ether: ethyl acetate= 3:1; R_f= 0.43) to produce compound **3da(mono)** (11 mg, 21% yield)^{1}H NMR (500 MHz, $CDCl_3$) δ 8.33 (d, *J* = 7.0 Hz, 1H), 8.11 (d, *J* = 15.8 Hz, 1H), 7.63 (dd, *J* = 5.3, 2.4 Hz, 1H), 7.40 – 7.33 (m, 3H), 7.30 – 7.23 (m, 4H), 7.22 – 7.18 (m, 2H), 6.37 (d, *J* = 15.8 Hz, 1H), 5.45 (d, *J* = 9.9 Hz, 1H), 5.04 (dd, *J* = 46.0, 12.3 Hz, 2H), 4.84 (d, *J* = 14.2 Hz, 1H), 4.65 (p, *J* = 7.3 Hz, 1H), 4.08 (d, *J* = 14.3 Hz, 1H), 3.72 (d, *J* = 9.9 Hz, 1H), 1.53 (s, 9H), 1.49 (d, *J* = 7.4 Hz, 3H), 1.03 (s, 9H).^{13}C NMR (125 MHz, $CDCl_3$) δ 172.6, 170.7, 168.3, 142.0, 135.3, 134.9, 131.9, 130.2, 129.3, 129.0, 128.5, 128.3, 128.0, 126.7, 120.9, 81.5, 67.1, 65.3, 56.8, 48.5, 34.9, 28.2, 26.5, 16.9. HRMS (ESI) $[M+Na]^+$ m/z calcd for $C_{30}H_{40}N_2O_7SNa$ 595.2454, found 595.2452.

<u>Compound 3qa(di)</u>

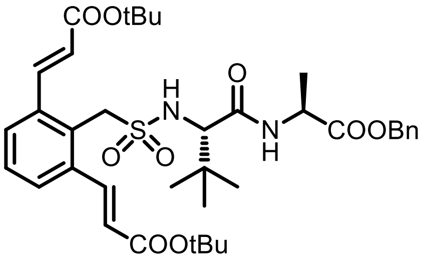

According to the general procedure, the crude residue was purified by flash column chromatography on silica gel (petroleum ether: ethyl acetate= 3:1; R_f= 0.53) to produce compound **3da(di)** (28 mg, 53% yield)^{1}H NMR (500 MHz, $CDCl_3$) δ 8.52 (d, *J* = 7.0 Hz, 1H), 8.15 (d, *J* = 15.7 Hz, 2H), 7.61 (d, *J* = 7.8 Hz, 2H), 7.36 (t, *J* = 7.8 Hz, 1H), 7.25 (dd, *J* = 5.4, 2.3 Hz, 3H), 7.20 – 7.14 (m, 2H), 6.32 (d, *J* = 15.7 Hz, 2H), 5.51 (d, *J* = 10.0 Hz, 1H), 5.02 (dd, *J* = 50.6, 12.4 Hz, 2H), 4.75 (d, *J* = 14.8 Hz, 1H), 4.64 (p, *J* = 7.3 Hz, 1H), 4.43 (d, *J* = 14.8 Hz, 1H), 3.73 (d, *J* = 10.0 Hz, 1H), 1.54 (s, 18H), 1.49 (d, *J* = 7.4 Hz, 3H), 1.03 (s, 9H).^{13}C NMR (125 MHz, $CDCl_3$) δ 172.7, 170.8, 135.2, 128.9, 128.5, 128.3, 128.2, 127.9, 67.1, 65.2, 52.4, 48.5, 34.9, 29.7, 28.2, 26.5, 16.8. HRMS (ESI) $[M+Na]^+$ m/z calcd for $C_{37}H_{50}N_2O_9SNa$ 721.3135, found 721.3132.

<u>Compound 3q'</u>

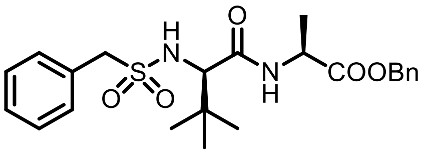

According to the general procedure, the crude residue was purified by flash column chromatography on silica gel (petroleum ether: ethyl acetate= 2:1; R_f= 0.37) to produce compound **3d'** (1.6 mg, 93% yield)^{1}H NMR (500 MHz, $CDCl_3$) δ 7.45 – 7.33 (m, 10H), 6.41 (d, *J* = 7.1 Hz, 1H), 5.26 (d, *J* = 9.4 Hz, 1H), 5.19 (dd, *J* = 38.8, 12.2 Hz, 2H), 4.62 (p, *J* = 7.2 Hz, 1H), 4.23 (s, 2H), 3.65 (d, *J* = 9.4 Hz, 1H), 1.43 (d, *J* = 7.2 Hz, 3H), 1.01 (s, 9H) ^{13}C NMR (125 MHz, $CDCl_3$) δ 172.2, 170.0, 135.1, 130.9, 128.8, 128.7, 128.6, 128.3, 67.4, 65.1, 59.7, 48.5, 34.8, 26.6, 18.1 HRMS (ESI) $[M+Na]^+$ m/z calcd for $C_{23}H_{30}N_2O_5SNa$ 469.1773, found 469.1770.

Compound 3q'a(mono)

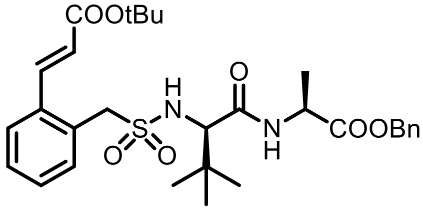

According to the general procedure, the crude residue was purified by flash column chromatography on silica gel (petroleum ether: ethyl acetate= 2:1; R_f= 0.41) to produce compound **3d'a(mono)** (13 mg, 19% yield) ^{1}H NMR (500 MHz, $CDCl_3$) δ 7.91 (d, *J* = 15.8 Hz, 1H), 7.75 (d, *J* = 7.3 Hz, 1H), 7.65 – 7.61 (m, 1H), 7.44 – 7.29 (m, 10H), 6.37 (d, *J* = 15.8 Hz, 1H), 5.42 (dd, *J* = 9.6, 3.5 Hz, 1H), 5.17 (dd, *J* = 30.6, 12.2 Hz, 2H), 4.64 (t, *J* = 7.3 Hz, 1H), 4.40 (d, *J* = 13.9 Hz, 1H), 4.22 (d, *J* = 13.9 Hz, 1H), 3.73 (d, *J* = 9.9 Hz, 1H), 1.53 (s, 9H), 1.42 (d, *J* = 7.3 Hz, 3H), 1.04 (s, 9H). ^{13}C NMR (125 MHz, $CDCl_3$) δ 171.9, 170.4, 167.5, 141.0, 134.7, 132.0, 130.8, 130.3, 129.3, 128.6, 128.5, 128.3, 126.8, 121.7, 81.6, 67.2, 65.3, 56.5, 48.6, 35.0, 29.7, 28.2, 26.5, 17.4. HRMS (ESI) $[M+Na]^+$ m/z calcd for $C_{30}H_{40}N_2O_7SNa$ 595.2454, found 595.2451.

.

Compound 3q'a(di)

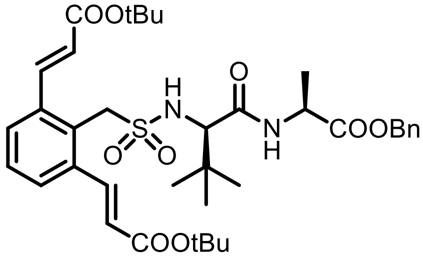

According to the general procedure, the crude residue was purified by flash column chromatography on silica gel (petroleum ether: ethyl acetate= 2:1; R_f= 0.49) to produce compound **3d'a(di)** (27 mg, 46% yield) ^{1}H NMR (500 MHz, $CDCl_3$) δ 8.12 (d, *J* = 7.4 Hz, 1H), 8.05 (d, *J* = 15.6 Hz, 2H), 7.60 (d, *J* = 7.8 Hz, 2H), 7.39 – 7.29 (m, 6H), 6.31 (d, *J* = 15.7 Hz, 2H), 5.58 (d, *J* = 10.2 Hz, 1H), 5.16 (dd, *J* = 31.6, 12.3 Hz, 2H), 4.62 (p, *J* = 7.3 Hz, 1H), 4.53 (d, *J* = 14.5 Hz, 1H), 4.34 (d, *J* = 14.4 Hz, 1H), 3.77 (d, *J* = 10.2 Hz, 1H), 1.53 (s, 18H), 1.40 (d, *J* = 7.3 Hz, 3H), 1.06 (s, 9H). ^{13}C NMR (125 MHz, $CDCl_3$) δ 171.8, 170.5, 141.1, 135.3, 129.2, 128.6, 128.4, 128.2, 127.4, 67.1, 65.3, 52.2, 48.6, 35.0, 28.2, 26.5, 17.2. HRMS (ESI) $[M+Na]^+$ m/z calcd for $C_{37}H_{50}N_2O_9SNa$ 721.3135, found 721.3132.

Compound 4a

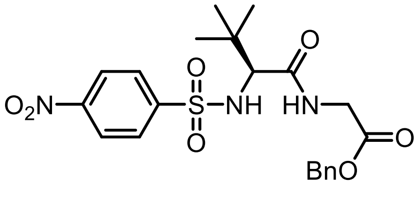

According to the general procedure, The crude residue was purified by flash column chromatography on silica gel (petroleum ether:ethyl acetate= 5:1; R_f=

0.43) to produce compound **4a** (1.5g, 89% yield).^{1}H NMR (400 MHz, $CDCl_3$) δ 8.40 – 8.18 (m, 2H), 8.07 – 7.90 (m, 2H), 7.48 – 7.28 (m, 5H), 5.92 (d, *J* = 5.0 Hz, 1H), 5.70 (d, *J* = 9.8 Hz, 1H), 5.13 (q, *J* = 12.1 Hz, 2H), 3.79 (dd, *J* = 7.8, 5.2 Hz, 2H), 3.47 (d, *J* = 9.8 Hz, 1H), 0.98 (s, 9H).^{13}C NMR (100 MHz, $CDCl_3$) δ 169.2, 169.0, 150.1, 145.4, 134.7, 128.8, 128.6, 124.2, 78.3 – 74.8, 67.7, 65.3, 41.0, 34.8, 26.5. HRMS (ESI) $[M+Na]^+$ m/z calcd for $C_{21}H_{25}N_3O_7SNa$ 486.1311, found 486.1317.

Compound 5ak

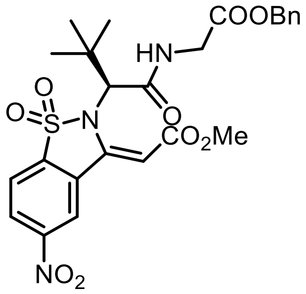

According to the general procedure, The crude residue was purified by flash column chromatography on silica gel (petroleum ether:ethyl acetate= 4:1; R_f = 0.46) to produce compound **5ak** (33 mg, 82% yield).^{1}H NMR (400 MHz, $CDCl_3$) δ 8.59 (d, *J* = 1.6 Hz, 1H), 8.50 (dd, *J* = 8.4, 1.7 Hz, 1H), 8.01 (d, *J* = 8.4 Hz, 1H), 7.46 – 7.27 (m, 5H), 7.02 (s, 1H), 6.05 (s, 1H), 5.94 (s, 1H), 5.15 (s, 2H), 4.19 (dd, *J* = 18.5, 5.3 Hz, 1H), 4.05 (dd, *J* = 18.5, 4.9 Hz, 1H), 3.84 (s, 3H), 1.28 (s, 9H).^{13}C NMR (100 MHz, $CDCl_3$) δ 169.5, 167.3, 165.2, 151.0, 142.1, 137.4, 135.1, 132.8, 129.3 – 128.0, 126.8, 122.7, 117.3, 94.9, 71.4, 67.2, 52.6, 41.7, 36.2, 28.5. HRMS (ESI) $[M+Na]^+$ m/z calcd for $C_{25}H_{27}N_3O_9SNa$ 568.1366, found 568.1357.

Compound 4b

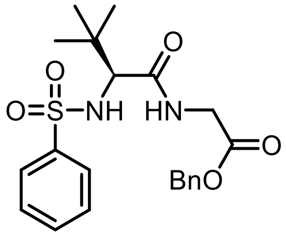

According to the general procedure, The crude residue was purified by flash column chromatography on silica gel (petroleum ether:ethyl acetate= 6:1; R_f = 0.43) to produce compound **4b** (1.2g, 92% yield). ^{1}H NMR (400 MHz, $CDCl_3$) δ 7.88 – 7.67 (m, 2H), 7.56 – 7.28 (m, 8H), 5.82 (s, 1H), 5.39 (d, *J* = 9.6 Hz, 1H), 5.16 (dd, *J* = 31.2, 12.1 Hz, 2H), 3.85 (dd, *J* = 18.5, 5.3 Hz, 1H), 3.49 (dd, *J* = 18.5, 4.7 Hz, 1H), 3.33 (d, *J* = 9.6 Hz, 1H), 0.96 (s, 9H).^{13}C NMR (100 MHz, $CDCl_3$) δ 169.5, 169.0, 139.5, 134.9, 132.7, 128.9, 128.8, 128.7, 128.6, 127.5, 79.2– 74.9, 67.4, 65.2, 41.2, 34.5, 26.4. HRMS (ESI) $[M+Na]^+$ m/z calcd for $C_{21}H_{26}N_2O_5SNa$ 441.1460, found 441.1452.

Compound 5bk

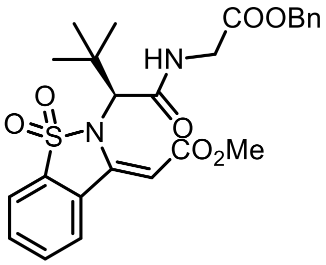

According to the general procedure, The crude residue was purified by flash column chromatography on silica gel (petroleum ether:ethyl acetate= 6:1; R_f= 0.43) to produce compound **5bk** (35 mg,79% yield).^{1}H NMR (400 MHz, $CDCl_3$) δ 7.93 – 7.78 (m, 1H), 7.78 – 7.72 (m, 1H), 7.69 (ddd, *J* = 5.3, 4.2, 1.9 Hz, 2H), 7.47 – 7.27 (m, 5H), 7.21 (t, *J* = 4.6 Hz, 1H), 6.09 (s, 1H), 5.84 (s, 1H), 5.13 (s, 2H), 4.22 – 4.01 (m, 2H), 3.80 (s, 3H), 1.31 (s, 9H). ^{13}C NMR (100 MHz, $CDCl_3$) δ 169.5, 167.8, 165.7, 143.9, 133.5, 132.5, 130.7, 128.5, 121.6, 121.0, 92.4, 78.9 – 75.8, 70.5, 67.1, 52.3, 41.7, 36.0, 28.5, 26.7. HRMS (ESI) $[M+Na]^+$ m/z calcd for $C_{25}H_{28}N_2O_7SNa$ 523.1515, found 523.1512.

Compound 4c

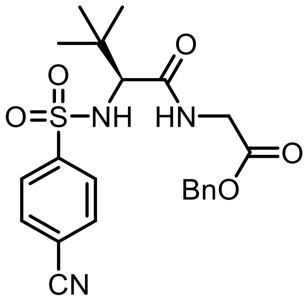

According to the general procedure, The crude residue was purified by flash column chromatography on silica gel (petroleum ether:ethyl acetate = 6:1; R_f= 0.45) to produce compound **4c** (1.6g, 85% yield). ^{1}H NMR (400 MHz, $CDCl_3$) δ 8.04 – 7.84 (m, 2H), 7.78 – 7.63 (m, 2H), 7.51 – 7.29 (m, 5H), 6.05 (t, *J* = 5.0 Hz, 1H), 5.75 (d, *J* = 9.8 Hz, 1H), 5.30 – 5.01 (m, 2H), 3.86 (dd, *J* = 18.4, 4.9 Hz, 1H), 3.73 (dd, *J* = 18.5, 5.3 Hz, 1H), 3.47 (d, *J* = 9.8 Hz, 1H), 0.96 (s, 9H). ^{13}C NMR (100 MHz, $CDCl_3$) δ 169.3, 169.1, 144.0, 134.8, 132.8, 128.8, 128.6, 128.0, 117.3, 116.3, 78.0 – 73.4, 67.7, 65.2, 41.1, 34.7, 26.5. HRMS (ESI) $[M+Na]^+$ m/z calcd for $C_{22}H_{25}N_3O_5SNa$ 466.1413, found 466.1407.

Compound 5ck

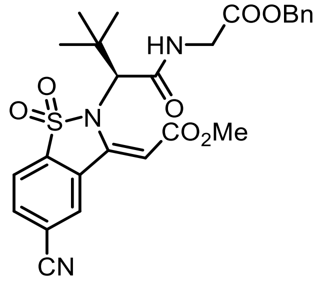

According to the general procedure, The crude residue was purified by flash column chromatography on silica gel (petroleum ether:ethyl acetate = 3:1; R_f= 0.48) to produce compound **5ck** (32mg, 85% yield). ^{1}H NMR (400 MHz, CD_3CN) δ 8.41 (s, 1H), 8.08 (dd, *J* = 8.0, 1.1 Hz, 1H), 8.03 (d, *J* = 8.0 Hz, 1H), 7.48 – 7.31 (m, 5H), 6.89 (s, 1H), 6.08 (s, 1H), 5.86 (s, 1H), 5.21 – 5.07 (m, 2H), 4.08 (dd, *J* = 17.7, 5.9 Hz, 1H), 3.95 – 3.85 (m, 1H), 3.81 (s, 3H), 1.23 (s, 9H). ^{13}C NMR (100 MHz, CD_3CN) δ 171.2 – 168.7, 167.5, 135.9, 128.5, 128.2, 128.1, 127.2, 121.6, 95.4, 71.1, 66.4, 52.2, 41.2, 36.0, 27.8. HRMS (ESI) $[M+Na]^+$ m/z calcd for $C_{26}H_{27}N_3O_7SNa$ 548.1467,

found 548.1462.

Compound 4d

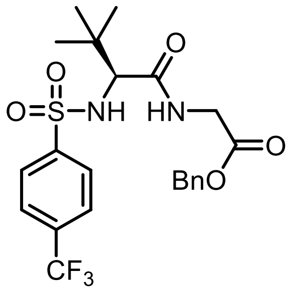

According to the general procedure, The crude residue was purified by flash column chromatography on silica gel (petroleum ether:ethyl acetate = 5:1; R_f = 0.41) to produce compound **4d** (1.6 g, 87% yield). ^{1}H NMR (400 MHz, $CDCl_3$) δ 7.95 (d, *J* = 8.2 Hz, 2H), 7.70 (d, *J* = 8.3 Hz, 2H), 7.56 – 7.28 (m, 5H), 5.97 (t, *J* = 4.7 Hz, 1H), 5.66 (d, *J* = 9.8 Hz, 1H), 5.14 (q, *J* = 12.1 Hz, 2H), 3.84 (dd, *J* = 18.5, 5.0 Hz, 1H), 3.59 (dd, *J* = 18.5, 5.0 Hz, 1H), 3.43 (d, *J* = 9.8 Hz, 1H), 0.97 (s, 9H). ^{13}C NMR (100 MHz, $CDCl_3$) δ 169.3, 169.0, 143.2, 134.8, 128.8, 128.7, 128.5, 128.0, 126.1, 126.0, 77.4, 77.0, 76.7, 67.6, 65.2, 41.1, 34.7, 26.4. HRMS (ESI) $[M+Na]^+$ m/z calcd for $C_{22}H_{25}F_3N_2O_5SNa$ 509.1334, found 509.1325.

Compound 5dk

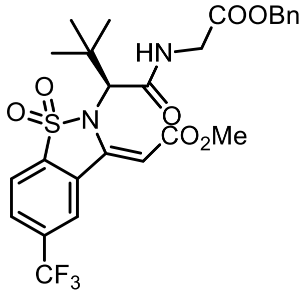

According to the general procedure, The crude residue was purified by flash column chromatography on silica gel (petroleum ether:ethyl acetate = 4:1; R_f = 0.47) to produce compound **5dk** (35 mg, 75% yield). ^{1}H NMR (400 MHz, $CDCl_3$) δ 8.09 – 7.83 (m, 3H), 7.55 – 7.31 (m, 5H), 7.12 (s, 1H), 6.08 (s, 1H), 5.90 (s, 1H), 5.17 (s, 2H), 4.19 (dd, *J* = 18.5, 5.3 Hz, 1H), 4.08 (dd, *J* = 18.5, 4.9 Hz, 1H), 3.84 (s, 3H), 1.31 (s, 9H). ^{13}C NMR (100 MHz, $CDCl_3$) δ 169.5, 167.4, 165.4, 142.7, 135.8, 131.7, 128.9, 128.6, 128.5, 128.4, 122.1, 119.0, 93.9, 82.1 – 74.4, 71.0, 67.2, 52.5, 41.7, 36.1, 29.3, 29.1, 28.5. HRMS (ESI) $[M+Na]^+$ m/z calcd for $C_{26}H_{27}F_3N_2O_7SNa$ 591.1389, found 591.1383.

Compound 4e

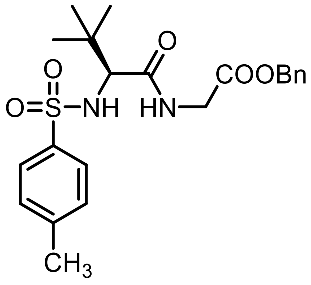

According to the general procedure, The crude residue was purified by flash column chromatography on silica gel (petroleum ether:ethyl acetate = 6:1; R_f = 0.46) to produce compound **4e** (1.6 g, 75% yield). ^{1}H NMR (400 MHz, $CDCl_3$) δ 7.69 (d, *J* = 8.3 Hz, 2H), 7.41 – 7.30 (m, 5H), 7.20 (d, *J* = 8.0 Hz, 2H), 6.09 (s, 1H), 5.47 (d, *J* = 9.3 Hz, 1H), 5.16 (q, *J* = 12.1 Hz, 2H), 3.88 (dd, *J* = 18.4, 5.3 Hz, 1H), 3.54 (dd, *J* = 18.4, 4.7 Hz, 1H), 3.38

(d, J = 9.5 Hz, 1H), 2.33 (s, 3H), 0.95 (s, 9H). ^{13}C NMR (100 MHz, $CDCl_3$) δ 169.8, 169.2, 143.5, 136.6, 134.9, 129.5, 128.7, 128.6, 127.5, 67.4, 65.1, 41.3, 34.5, 26.4, 21.5. HRMS (ESI) $[M+Na]^+$ m/z calcd for $C_{22}H_{28}N_2O_5SNa$ 455.1617, found 455.1611.

Compound 5ek

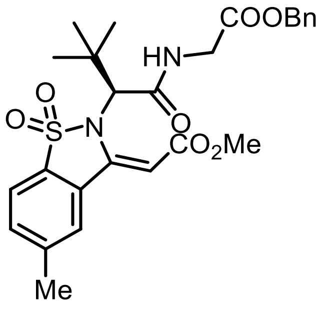

According to the general procedure, The crude residue was purified by flash column chromatography on silica gel (petroleum ether:ethyl acetate = 6:1; R_f = 0.46) to produce compound **5ek** (1.6 g, 80% yield). ^1H NMR (400 MHz, $CDCl_3$) δ 7.70 (d, J = 7.9 Hz, 1H), 7.53 (s, 1H), 7.48 (d, J = 8.0 Hz, 1H), 7.33 (dd, J = 8.2, 3.5 Hz, 5H), 6.08 (s, 1H), 5.81 (s, 1H), 5.13 (s, 2H), 4.09 (qd, J = 18.4, 5.1 Hz, 2H), 3.79 (s, 3H), 2.50 (s, 3H), 1.31 (s, 9H). ^{13}C NMR (100 MHz, $CDCl_3$) δ 169.6, 167.9, 144.7, 144.1, 133.1, 130.9, 128.7, 128.6, 128.5, 128.4, 124.2, 121.7, 120.8, 92.0, 70.5, 67.7, 67.1, 52.3, 41.7, 36.0, 28.5, 26.6, 22.0. HRMS (ESI) $[M+Na]^+$ m/z calcd for $C_{26}H_{29}N_3O_9SNa$ 537.1671, found 537.1668.

Compound 4f

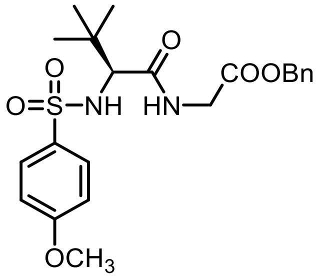

According to the general procedure, The crude residue was purified by flash column chromatography on silica gel (petroleum ether: ethyl acetate = 6:1; R_f = 0.44) to produce compound **4f** (1.5 g, 85% yield). ^1H NMR (400 MHz, $CDCl_3$) δ 7.77 – 7.71 (m, 2H), 7.41 – 7.32 (m, 5H), 6.93 – 6.86 (m, 2H), 5.87 (s, 1H), 5.33 (d, J = 9.4 Hz, 1H), 5.21 – 5.11 (m, 2H), 3.89 (dd, J = 18.5, 5.2 Hz, 1H), 3.79 (s, 3H), 3.61 (dd, J = 18.5, 4.8 Hz, 1H), 3.30 (d, J = 9.4 Hz, 1H), 0.95 (s, 9H). ^{13}C NMR (100 MHz, $CDCl_3$) δ 169.8, 169.1, 162.94, 134.9, 131.1, 129.7, 128.7, 128.5, 114.0, 67.4, 65.2, 55.58, 41.3, 34.5, 26.5. HRMS (ESI) $[M+Na]^+$ m/z calcd for $C_{22}H_{28}N_2O_6SNa$ 471.1566, found 471.1560.

Compound 5fk

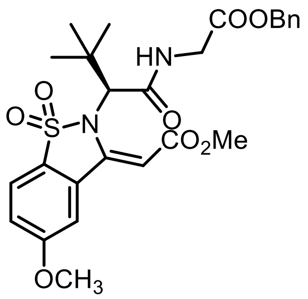

According to the general procedure, The crude residue was purified by flash column chromatography on silica gel (petroleum ether : ethyl acetate = 3:1; R_f = 0.44) to produce compound **5fk** (1.5 g, 76% yield). ^{1}H NMR (400 MHz, $CDCl_3$) δ 7.72 (d, *J* = 8.6 Hz, 1H), 7.33 (t, *J* = 4.4 Hz, 5H), 7.17 (dd, *J* = 8.6, 2.1 Hz, 1H), 7.12 (d, *J* = 2.0 Hz, 1H), 6.05 (s, 1H), 5.77 (s, 1H), 5.14 (s, 2H), 4.09 (dd, *J* = 8.2, 5.1 Hz, 2H), 3.92 (s, 3H), 3.80 (s, 3H), 1.32 (s, 9H). ^{13}C NMR (100 MHz, $CDCl_3$) δ 128.6, 128.5, 128.4, 122.6, 119.1, 105.5, 92.2, 70.7, 67.1, 56.1, 52.3, 41.7, 36.0, 29.3, 28.6, 27.2, 26.7. HRMS (ESI) $[M+Na]^+$ m/z calcd for $C_{26}H_{30}N_2O_8SNa$ 553.1621, found 553.1617.

Compound 4g

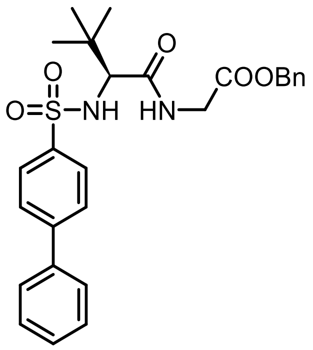

According to the general procedure, The crude residue was purified by flash column chromatography on silica gel (petroleum ether : ethyl acetate = 5:1; R_f = 0.41) to produce compound **4g** (1.6 g, 87% yield). ^{1}H NMR (400 MHz, $CDCl_3$) δ 7.94 – 7.83 (m, 2H), 7.72 – 7.62 (m, 2H), 7.61 – 7.52 (m, 2H), 7.52 – 7.38 (m, 3H), 7.38 – 7.29 (m, 3H), 7.22 (dd, *J* = 6.5, 3.0 Hz, 2H), 5.93 (t, *J* = 4.6 Hz, 1H), 5.52 (d, *J* = 9.6 Hz, 1H), 5.06 (d, *J* = 12.1 Hz, 1H), 4.91 (d, *J* = 12.1 Hz, 1H), 3.84 (dd, *J* = 18.5, 5.1 Hz, 1H), 3.55 (dd, *J* = 18.5, 4.8 Hz, 1H), 3.40 (d, *J* = 9.6 Hz, 1H), 0.98 (s, 9H). ^{13}C NMR (100 MHz, $CDCl_3$) δ 169.6, 169.0, 145.5, 139.1, 138.0, 134.7, 129.1, 128.6, 128.4, 128.1, 127.5, 127.3, 67.4, 65.2, 41.2, 34.6, 26.5. HRMS (ESI) $[M+Na]^+$ m/z calcd for $C_{27}H_{30}N_2O_5SNa$ 517.1773, found 517.1769.

Compound 5gk

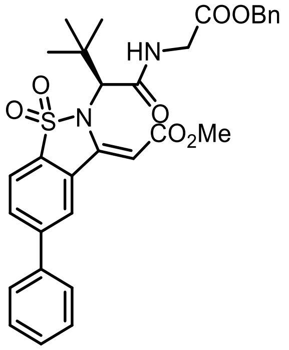

According to the general procedure, The crude residue was purified by flash column chromatography on silica gel (petroleum ether : ethyl acetate = 3:1; R_f= 0.43) to produce compound **5gk** (36 mg, 81% yield). ^{1}H NMR (400 MHz, CD_3CN) δ 8.21 (d, *J* = 1.0 Hz, 1H), 8.02 (dd, *J* = 8.1, 1.4 Hz, 1H), 7.95 (d, *J* = 8.1 Hz, 1H), 7.82 – 7.72 (m, 2H), 7.63 – 7.46 (m, 3H), 7.43 – 7.28 (m, 5H), 6.96 (d, *J* = 5.1 Hz, 1H), 6.21 (s, 1H), 5.99 (s, 1H), 5.20 – 5.05 (m, 2H), 4.12 – 4.02 (m, 1H), 3.94 – 3.85 (m, 1H), 3.80 (s, 3H), 1.29 (s, 9H) ^{13}C

NMR (100 MHz, CD_3CN) δ 169.6, 167.7, 165.9, 146.8, 144.1, 138.5, 136.0, 131.5, 129.2, 129.0, 128.5, 128.2, 128.1, 127.7, 120.9, 120.8, 117.3, 93.4, 70.1, 66.4, 52.0, 41.2, 35.8, 27.8. HRMS (ESI) $[M+Na]^+$ m/z calcd for $C_{25}H_{27}N_3O_9SNa$ 599.1828, found 599.1822.

Compound 4h

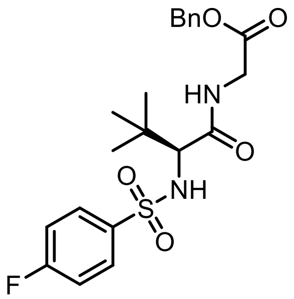

According to the general procedure, The crude residue was purified by flash column chromatography on silica gel (petroleum ether : ethyl acetate = 7:1; R_f = 0.48) to produce compound **4h** (1.2g, 77% yield). ^{1}H NMR (400 MHz, $CDCl_3$) δ 7.98 – 7.71 (m, 2H), 7.50 – 7.29 (m, 5H), 7.19 – 6.97 (m, 2H), 5.91 (d, J = 4.5 Hz, 1H), 5.46 (d, J = 9.6 Hz, 1H), 5.28 – 5.01 (m, 2H), 3.89 (dd, J = 18.5, 5.1 Hz, 1H), 3.65 (dd, J = 18.5, 5.0 Hz, 1H), 3.36 (d, J = 9.6 Hz, 1H), 0.96 (s, 5H). ^{13}C NMR (100 MHz, $CDCl_3$) δ 169.5, 169.7, 166.3, 163.8, 135.7, 134.8, 130.3, 130.2, 128.8, 128.7, 128.6, 77.7 – 76.5, 67.6, 65.2, 41.2, 34.6, 26.4. HRMS (ESI) $[M+Na]^+$ m/z calcd for $C_{21}H_{25}FN_5O_5SNa$ 459.1366, found 459.1357.

Compound 5hk

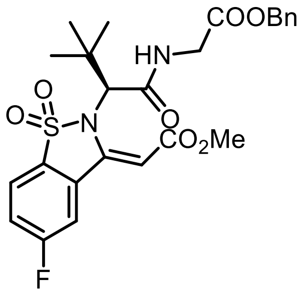

According to the general procedure, The crude residue was purified by flash column chromatography on silica gel (petroleum ether : ethyl acetate = 7:1; R_f = 0.48) to produce compound **5hk** (28 mg, 80% yield). ^{1}H NMR (400 MHz, $CDCl_3$) δ 7.82 (dd, J = 8.4, 4.6 Hz, 1H), 7.53 – 7.28 (m, 7H), 7.16 (t, J = 4.7 Hz, 1H), 6.05 (s, 1H), 5.74 (s, 1H), 5.14 (s, 2H), 4.10 (qd, J = 18.5, 5.1 Hz, 2H), 3.80 (s, 3H), 1.30 (s, 9H). ^{13}C NMR (100 MHz, $CDCl_3$) δ 169.5, 167.6, 166.9, 165.4, 164.4, 142.8, 135.1, 128.6, 128.5, 128.4, 123.6, 123.5, 120.2, 120.0, 109.0, 108.7, 93.3, 78.8 – 74.4, 70.9, 67.2, 52.4, 41.7, 36.0, 28.5. HRMS (ESI) $[M+Na]^+$ m/z calcd for $C_{25}H_{27}FN_2O_7SNa$ 541.1421, found 541.1409.

Compound 4i

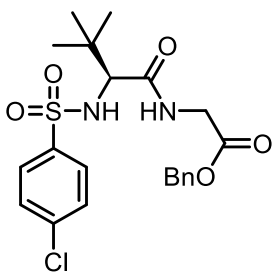

According to the general procedure, The crude residue was purified by flash column chromatography on silica gel (petroleum ether : ethyl acetate = 6:1; R_f = 0.41) to produce compound **4i** (1.1g, 87% yield). ^{1}H NMR (400 MHz, $CDCl_3$) δ 7.89 – 7.62 (m, 2H), 7.61 – 7.30 (m, 7H), 5.93 (s, 1H), 5.51 (d, *J* = 9.7 Hz, 1H), 5.18 (s, 1H), 3.87 (dd, *J* = 18.5, 5.1 Hz, 1H), 3.64 (dd, *J* = 18.5, 5.0 Hz, 1H), 3.36 (d, *J* = 9.7 Hz, 1H), 0.96 (s, 9H). ^{13}C NMR (100 MHz, $CDCl_3$) δ 169.4, 169.1, 139.2, 138.1 , 134.8 , 129.2 , 128.9 , 128.8 , 128.7, 128.6, 77.4, 77.0, 76.7, 67.6, 65.2 , 41.2 , 34.6 , 26.4. HRMS (ESI) $[M+Na]^+$ m/z calcd for $C_{21}H_{25}ClN_2O_5SNa$ 475.1070, found 475.1064.

Compound 5ik

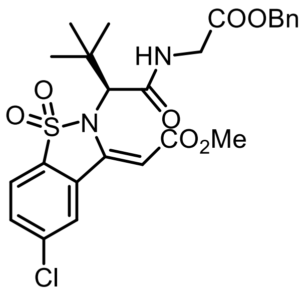

According to the general procedure, The crude residue was purified by flash column chromatography on silica gel (petroleum ether : ethyl acetate = 4:1; R_f = 0.43) to produce compound **5ik** (20 mg, 85% yield). ^{1}H NMR (400 MHz, $CDCl_3$) δ 7.75 (d, *J* = 8.3 Hz, 1H), 7.70 (d, *J* = 1.3 Hz, 1H), 7.63 (dd, *J* = 8.2, 1.5 Hz, 1H), 7.46 – 7.28 (m, 5H), 7.14 (s, 1H), 6.03 (s, 1H), 5.77 (s, 1H), 5.14 (s, 1H), 4.15 (dd, *J* = 18.4, 5.3 Hz, 1H), 4.05 (dd, *J* = 18.4, 5.0 Hz, 1H), 3.80 (s, 1H), 1.29 (s, 9H). ^{13}C NMR (100 MHz, $CDCl_3$) δ 169.5, 167.6, 165.4, 142.7, 140.3, 135.2, 132.6, 132.4, 131.2, 129.24 – 127.36, 122.3, 121.8, 93.4, 81.4 – 73.6, 70.9, 67.2, 52.4, 41.7, 36.1, 28.5. HRMS (ESI) $[M+Na]^+$ m/z calcd for $C_{25}H_{27}ClN_3O_7SNa$ 557.1125, found 557.1221.

Compound 4j

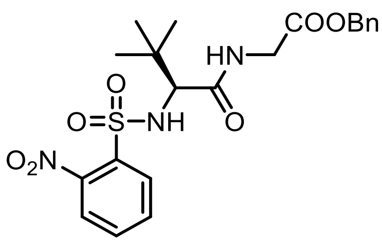

According to the general procedure, The crude residue was purified by flash column chromatography on silica gel (petroleum ether : ethyl acetate = 4:1; R_f = 0.38) to produce compound **4j** (1.1 g, 86% yield).

^{1}H NMR (400 MHz, $CDCl_3$) δ 8.03 (dd, *J* = 7.5, 1.7 Hz, 1H), 7.83 (dd, *J* = 7.7, 1.5 Hz, 1H), 7.63 – 7.53 (m, 2H), 7.42 – 7.30 (m, 4H), 6.24 (d, *J* = 8.8 Hz, 1H), 6.14 (s, 1H), 5.15 (q, *J* = 12.1 Hz, 2H), 3.76 (ddd, *J* = 45.2, 18.4, 5.2 Hz, 2H), 3.64 (d, *J* = 8.8 Hz, 1H), 1.01 (s, 9H). ^{13}C NMR (100 MHz, $CDCl_3$) δ 169.1, 169.0, 135.0, 133.6, 132.6, 130.5, 128.8, 128.7, 128.6, 125.6, 67.4, 66.2, 41.1, 34.6, 26.5. HRMS (ESI) $[M+Na]^+$ m/z calcd for $C_{21}H_{25}N_3O_7SNa$ 486.1311, found 486.1306.

Compound 5jk

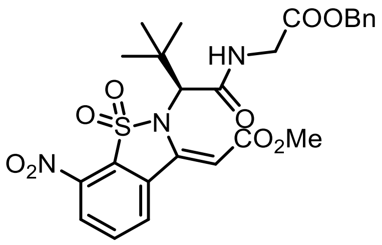

According to the general procedure, The crude residue was purified by flash column chromatography on silica gel (petroleum ether : ethyl acetate = 4:1; R_f = 0.38) to produce compound **5jk** (26 mg, 72% yield). ^{1}H NMR (400 MHz, $CDCl_3$) δ 8.47 (d, *J* = 8.0 Hz, 1H), 8.12 (d, *J* = 7.9 Hz, 1H), 7.91 (t, *J* = 8.1 Hz, 1H), 7.34 (m, 5H), 7.04 (t, *J* = 4.6 Hz, 1H), 6.04 (s, 1H), 5.94 (s, 1H), 5.13 (s, 2H), 4.10 (m, 2H), 3.81 (s, 3H), 1.36 (s, 9H). ^{13}C NMR (100 MHz, $CDCl_3$) δ 169.5, 167.7, 165.2, 142.5, 142.2, 135.1, 134.7, 134.4, 128.6, 128.5, 128.4, 127.5, 127.3, 94.4, 71.6, 67.2, 52.5, 41.7, 36.0, 28.9 26.9, 26.5. HRMS (ESI) $[M+Na]^+$ m/z calcd for $C_{25}H_{27}N_3O_9SNa$ 568.1366, found 568.1362.

Compound 4k

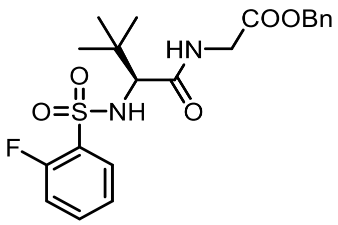

According to the general procedure, The crude residue was purified by flash column chromatography on silica gel (petroleum ether : ethyl acetate = 6:1; R_f = 0.48) to produce compound **4k** (1.3 g, 78% yield). ^{1}H NMR (400 MHz, $CDCl_3$) δ 7.80 (td, *J* = 7.5, 1.6 Hz, 1H), 7.61 – 7.24 (m, 6H), 7.24 – 7.04 (m, 2H), 6.06 (t, *J* = 4.9 Hz, 1H), 5.68 (d, *J* = 9.5 Hz, 1H), 5.14 (dd, *J* = 32.4, 12.1 Hz, 2H), 3.88 (dd, *J* = 18.4, 5.4 Hz, 1H), 3.53 (dd, *J* = 18.4, 4.8 Hz, 1H), 3.47 (d, *J* = 9.6 Hz, 1H), 0.98 (s, 9H). ^{13}C NMR (100 MHz, $CDCl_3$) δ 169.3, 169.1, 160.3, 157.8, 135.6 – 134.2, 130.2, 129.7 – 128.4, 124.2, 117.0, 116.8, 78.9 – 75.2, 67.4, 65.3, 41.2, 34.5, 26.4. HRMS (ESI) $[M+Na]^+$ m/z calcd for $C_{21}H_{25}FN_2O_5SNa$ 459.1366, found 459.1363.

Compound 5kk

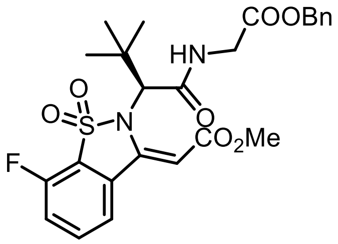

According to the general procedure, The crude residue was purified by flash column chromatography on silica gel (petroleum ether : ethyl acetate = 3:1; R_f = 0.43) to produce compound **5kk** (23 mg, 72% yield). ^{1}H NMR (400 MHz, $CDCl_3$) δ 7.75 – 7.59 (m, 1H), 7.54 (d, *J* = 7.9 Hz, 1H), 7.46 – 7.28 (m, 6H), 7.12 (s, 1H), 6.04 (s, 1H), 5.85 (s, 1H), 5.14 (s, 2H), 4.10 (qd, *J* = 18.4, 5.1 Hz, 2H), 3.80 (s, 3H), 1.32 (s, 9H). ^{13}C NMR (100 MHz, $CDCl_3$) δ 169.5, 167.6, 165.4, 143.2, 135.6, 128.6, 118.9, 117.2, 93.7, 82.4 – 75.9, 71.0, 67.1, 52.4, 41.7, 36.0, 28.6, 26.6. HRMS (ESI) $[M+Na]^+$ m/z calcd for $C_{25}H_{27}FN_2O_7SNa$ 541.1421, found 541.1416.

Compound 4l

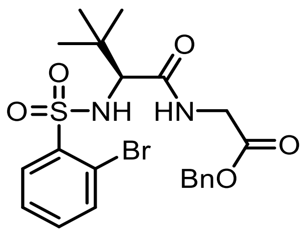

According to the general procedure, The crude residue was purified by flash column chromatography on silica gel (petroleum ether : ethyl acetate = 6:1; R_f = 0.43) to produce compound **4l** (1.4 g, 83% yield). ^{1}H NMR (400 MHz, $CDCl_3$) δ 8.02 (dd, *J* = 7.8, 1.8 Hz, 1H), 7.67 (dd, *J* = 7.8, 1.3 Hz, 1H), 7.49 – 7.11 (m, 7H), 6.01 (t, *J* = 5.0 Hz, 1H), 5.93 (d, *J* = 9.4 Hz, 1H), 5.13 (dd, *J* = 31.4, 12.1 Hz, 2H), 3.86 (dd, *J* = 18.4, 5.4 Hz, 1H), 3.51 (dd, *J* = 18.4, 4.8 Hz, 1H), 3.42 (d, *J* = 9.4 Hz, 1H), 0.97 (s, 9H). ^{13}C NMR (100 MHz, $CDCl_3$) δ 169.0, 138.8, 135.0, 133.6, 131.0, 129.7 – 128.0, 127.5, 120.6, 78.5 – 74.5, 67.4, 65.6, 41.2, 34.5, 26.4. HRMS (ESI) $[M+Na]^+$ m/z calcd for $C_{21}H_{25}BrN_2O_5SNa$ 519.0565, found 519.0562.

Compound 5lk

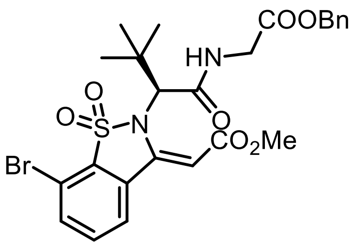

According to the general procedure, The crude residue was purified by flash column chromatography on silica gel (petroleum ether : ethyl acetate = 4:1; R_f = 0.41) to produce compound **5lk** (34 mg, 60% yield). ^{1}H NMR (400 MHz, $CDCl_3$) δ 7.86 – 7.79 (m, 1H), 7.74 (dd, *J* = 6.2, 2.7 Hz, 1H), 7.68 (ddd, *J* = 6.6, 3.7, 1.7 Hz, 1H), 7.41 – 7.27 (m, 5H), 7.21 (t, *J* = 4.6 Hz, 1H), 6.08 (d, *J* = 7.2 Hz, 1H), 5.84 (s, 1H), 5.13 (s, 3H), 4.09 (dd, *J* = 15.3, 5.1 Hz, 2H), 3.79 (s, 3H), 1.31 (s, 9H). ^{13}C NMR (100 MHz, $CDCl_3$) δ 169.5, 167.8, 165.7, 143.9, 135.2, 134.0, 133.5, 132.8, 132.1, 130.7, 128.4, 121.6, 121.0, 92.4, 78.9 – 75.6, 70.5, 67.1, 52.3, 41.7, 36.0, 28.5. HRMS (ESI) $[M+Na]^+$ m/z calcd for $C_{25}H_{27}BrN_2O_7SNa$ 601.6020, found 601.6015.

Compound 4m

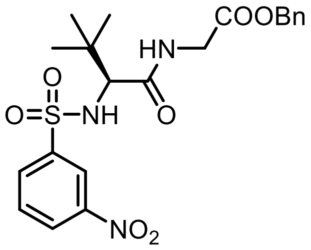

According to the general procedure, The crude residue was purified by flash column chromatography on silica gel (petroleum ether : ethyl acetate = 4:1; R_f = 0.41) to produce compound **4m** (34 mg, 62% yield). ^{1}H NMR (400 MHz, $CDCl_3$) δ 8.65 (t, *J* = 1.9 Hz, 1H), 8.28 (ddd, *J* = 8.2, 2.2, 1.0 Hz, 1H), 8.13 (ddd, *J* = 7.8, 1.6, 1.0 Hz, 1H), 7.60 (t, *J* = 8.0 Hz, 1H), 7.49 – 7.29 (m, 4H), 6.19 (t, *J* = 5.1 Hz, 1H), 5.78 (d, *J* = 9.9 Hz, 1H), 5.13 (q, *J* = 12.1 Hz, 2H), 3.77 (ddd, *J* = 51.3, 18.4, 5.2 Hz, 2H), 3.57 (d, *J* = 9.9 Hz, 1H), 0.98 (s, 9H). ^{13}C NMR (100 MHz, $CDCl_3$) δ 169.3, 169.0, 148.1, 142.0, 134.9, 132.9, 130.4, 128.7, 128.5, 127.1, 122.5, 78.6 – 74.9, 67.5, 65.3, 41.1, 34.8, 26.4. HRMS (ESI) $[M+Na]^+$ m/z calcd for $C_{21}H_{25}N_3O_7SNa$ 486.1311, found 486.1306.

Compound 5mk

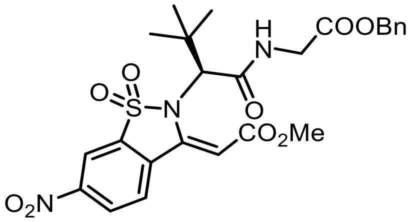

According to the general procedure, The crude residue was purified by flash column chromatography on silica gel (petroleum ether : ethyl acetate = 3:1; R_f = 0.43) to produce compound **5mk** (36 mg, 80% yield). ^{1}H NMR (400 MHz, $CDCl_3$) δ 8.66 (d, *J* = 1.6 Hz, 1H), 8.51 (dd, *J* = 8.8, 1.9 Hz, 2H), 7.92 (d, *J* = 8.7 Hz, 1H), 7.33 (dd, *J* = 12.8, 6.9 Hz, 5H), 7.04 (s, 1H), 6.00 (s, 1H), 5.94 (s, 1H), 5.14 (s, 2H), 4.18 (dd, *J* = 18.5, 5.2 Hz, 1H), 4.09 – 3.98 (m, 1H), 3.83 (s, 3H), 1.30 (s, 9H). ^{13}C NMR (100 MHz, $CDCl_3$) δ 142.2, 128.6, 128.4, 128.1, 123.0, 117.3, 96.0, 78.2 – 75.1, 71.7, 67.2, 52.7, 41.7, 28.6. HRMS (ESI) $[M+Na]^+$ m/z calcd for $C_{25}H_{27}N_3O_9SNa$ 568.1366, found 568.1363.

Compound 4n

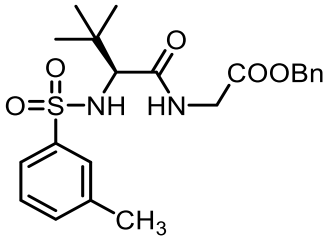

According to the general procedure, The crude residue was purified by flash column chromatography on silica gel (petroleum ether : ethyl acetate = 3:1; R_f = 0.43) to produce compound **4n** (1.4g, 84% yield). ^{1}H NMR (400 MHz, $CDCl_3$) δ 7.71 – 7.51 (m, 2H), 7.45 – 7.30 (m, 5H), 7.25 (dt, *J* = 18.0, 7.6 Hz, 2H), 5.88 (t, *J* = 4.7 Hz, 1H), 5.40

(t, J = 7.9 Hz, 1H), 5.16 (dd, J = 29.8, 12.1 Hz, 2H), 3.87 (dd, J = 18.5, 5.3 Hz, 1H), 3.52 (dd, J = 18.5, 4.6 Hz, 1H), 3.34 (d, J = 9.5 Hz, 1H), 2.35 (s, 3H), 0.96 (s, 9H). ^{13}C NMR (100 MHz, $CDCl_3$) δ 169.6, 169.1, 139.3, 139.2, 134.9, 133.4, 129.0 – 128.3, 127.8, 124.6, 67.4, 65.2, 43.5, 41.3, 34.5, 26.4, 21.3. HRMS (ESI) $[M+Na]^+$ m/z calcd for $C_{22}H_{28}N_2O_5SNa$ 455.1617, found 455.1623.

Compound 5nk

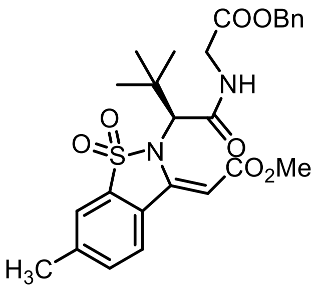

According to the general procedure, The crude residue was purified by flash column chromatography on silica gel (petroleum ether : ethyl acetate = 3:1; R_f = 0.43) to produce compound **5nk** (33 mg, 81% yield). ^{1}H NMR (400 MHz, CD_3CN) δ 7.87 (d, J = 8.3 Hz, 1H), 7.70 (s, 1H), 7.61 (d, J = 8.2 Hz, 1H), 7.48 – 7.27 (m, 5H), 6.88 (s, 1H), 6.03 (s, 1H), 5.97 (s, 1H), 5.19 – 5.02 (m, 2H), 4.04 (dd, J = 17.7, 5.8 Hz, 1H), 3.86 (dd, J = 17.7, 5.5 Hz, 1H), 3.77 (s, 3H), 2.51 (s, 3H), 1.27 (s, 9H). ^{13}C NMR (100 MHz, CD_3CN) δ 169.6, 167.7, 165.9, 144.4, 136.0, 134.9, 128.5, 128.2, 128.0, 122.3, 120.3, 117.3, 92.0, 69.7, 66.4, 51.9, 41.2, 35.7, 27.7, 20.5. HRMS (ESI) $[M+Na]^+$ m/z calcd for $C_{26}H_{30}N_2O_7SNa$ 537.1671, found 537.1667.

Compound 5aa

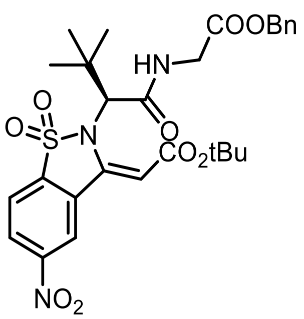

According to the general procedure, The crude residue was purified by flash column chromatography on silica gel (petroleum ether : ethyl acetate = 3:1; R_f = 0.43) to produce compound **5aa** (33 mg, 79% yield). ^{1}H NMR (500 MHz, $CDCl_3$) δ 8.60 (d, J = 1.5 Hz, 1H), 8.51 (dd, J = 8.4, 1.7 Hz, 1H), 8.02 (d, J = 8.4 Hz, 1H), 7.50 – 7.31 (m, 5H), 7.15 (d, J = 4.7 Hz, 1H), 5.95 (s, 1H), 5.90 (s, 1H), 5.17 (s, 2H), 4.23 (dd, J = 18.5, 5.3 Hz, 1H), 4.08 (dd, J = 18.5, 4.8 Hz, 1H), 1.59 (s, 9H), 1.27 (s, 9H). ^{13}C NMR (100 MHz, $CDCl_3$) δ 169.4, 167.2, 164.5, 150.9, 140.4, 137.4, 133.0, 130.0 – 127.7, 126.50, 122.6, 117.2, 97.5, 82.7, 79.1 – 75.1, 70.6, 67.2, 41.7, 36.3, 29.1, 28.3. HRMS (ESI) $[M+Na]^+$ m/z calcd for $C_{28}H_{33}N_3O_9SNa$ 610.1835, found 610.1831.

Compound 5ab

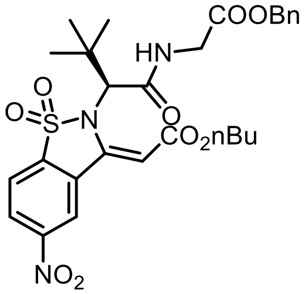

According to the general procedure, The crude residue was purified by flash column chromatography on silica gel (petroleum ether : ethyl acetate = 4:1; R_f = 0.46) to produce compound **5ab** (29 mg, 72% yield). ^{1}H NMR (400 MHz, $CDCl_3$) δ 8.60 (d, J = 1.5 Hz, 1H), 8.50 (dd, J = 8.4, 1.6 Hz, 1H), 8.01 (d, J = 8.4 Hz, 1H), 7.33 (d, J = 3.8 Hz,

5H), 7.04 (t, *J* = 4.7 Hz, 1H), 6.05 (s, 1H), 5.93 (s, 1H), 5.15 (s, 2H), 4.34 – 4.13 (m, 3H), 4.06 (dd, *J* = 18.4, 4.8 Hz, 1H), 1.86 – 1.59 (m, 2H), 1.45 (dd, *J* = 15.0, 7.5 Hz, 2H), 1.27 (s, 9H), 0.97 (dd, *J* = 15.0, 7.5 Hz, 3H). ^{13}C NMR (100 MHz, $CDCl_3$) δ 169.5, 167.2, 165.0, 150.9, 141.9, 137.5, 135.1, 132.9, 128.4 126.8, 122.6, 117.3, 95.4, 80.2 – 74.2, 71.1, 67.2, 65.6, 41.7, 36.2, 30.6, 28.5, 19.1, 13.7. HRMS (ESI) $[M+Na]^+$ m/z calcd for $C_{28}H_{33}N_3O_9SNa$ 610.1835, found 610.1832.

Compound 5ac

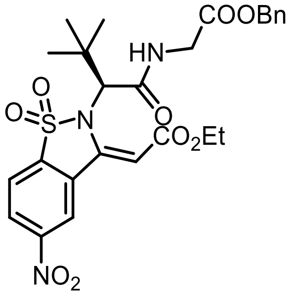

According to the general procedure, The crude residue was purified by flash column chromatography on silica gel (petroleum ether : ethyl acetate = 4:1; R_f = 0.46) to produce compound **5ac** (23 mg, 78% yield). ^{1}H NMR (400 MHz, $CDCl_3$) δ 8.60 (s, 1H), 8.51 (dd, *J* = 8.4, 1.4 Hz, 1H), 8.01 (d, *J* = 8.4 Hz, 1H), 7.49 – 7.28 (m, 5H), 7.05 (s, 1H), 6.05 (s, 1H), 5.93 (s, 1H), 5.15 (s, 2H), 4.47 – 4.25 (m, 2H), 4.20 (dd, *J* = 18.5, 5.3 Hz, 1H), 4.06 (dd, *J* = 18.4, 4.8 Hz, 1H), 1.37 (t, *J* = 7.1 Hz, 3H), 1.27 (s, 9H). ^{13}C NMR (100 MHz, $CDCl_3$) δ 169.5, 167.3, 164.8, 150.9, 141.8, 132.1 – 127.8, 126.8, 122.7, 117.30, 95.4, 80.8 – 73.2, 71.2, 67.2, 61.7, 41.7, 36.2, 28.5, 14.2. HRMS (ESI) $[M+Na]^+$ m/z calcd for $C_{26}H_{29}N_3O_9SNa$ 582.1522, found 582.1518.

Compound 5ad

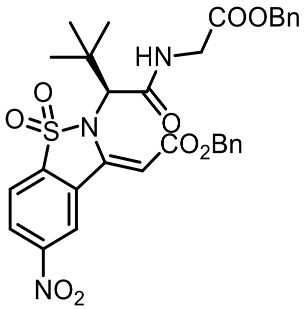

According to the general procedure, The crude residue was purified by flash column chromatography on silica gel (petroleum ether : ethyl acetate = 3:1; R_f = 0.41) to produce compound **5ad** (31 mg, 72% yield). ^{1}H NMR (400 MHz, $CDCl_3$) δ 8.58 (d, *J* = 1.6 Hz, 1H), 8.50 (dd, *J* = 8.4, 1.7 Hz, 1H), 8.00 (d, *J* = 8.4 Hz, 1H), 7.61 – 7.27 (m, 8H), 6.98 (t, *J* = 4.7 Hz, 1H), 6.03 (s, 1H), 5.96 (s, 1H), 5.25 (q, *J* = 12.2 Hz, 2H), 5.15 (s, 2H), 4.19 (dd, *J* = 18.5, 5.3 Hz, 1H), 4.05 (dd, *J* = 18.5, 4.8 Hz, 1H), 1.25 (s, 9H). ^{13}C NMR (100 MHz, $CDCl_3$) δ 169.5, 167.2, 164.6, 151.0, 142.3, 135.2, 131.0 – 127.7, 126.8, 122.7, 117.4, 94.8, 81.2 – 73.6, 71.3, 67.5, 67.2, 41.7, 36.2, 28.5. HRMS (ESI) $[M+Na]^+$ m/z calcd for $C_{31}H_{31}N_3O_9SNa$ 644.1679, found 644.1675.

Compound 5af

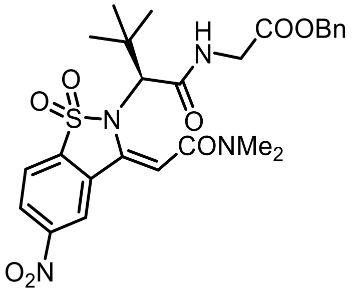

According to the general procedure, The crude residue was purified by flash column chromatography on silica gel (petroleum ether : ethyl acetate = 3:1; R_f = 0.43) to produce compound **5af** (30 mg, 85% yield). ^{1}H NMR (400 MHz, CD_3CN) δ 8.77 (d, *J* = 1.8 Hz, 1H), 8.46 (dd, *J* = 8.5, 1.9 Hz, 1H), 8.06 (d, *J* = 8.5 Hz, 1H), 7.67 (s, 1H), 7.51 – 7.23 (m, 5H), 6.41 (s, 1H), 5.27 – 5.08 (m, 2H), 5.05 (s, 1H), 4.18 – 4.00 (m, 1H), 3.91 (dd, *J* = 17.8, 5.5 Hz, 1H), 3.14 (s, 3H), 3.05 (s, 3H), 1.16 (s, 9H). ^{13}C NMR (100 MHz, CD_3CN) δ 169.5, 167.0, 165.6, 151.3, 136.6, 136.0, 135.3, 132.7, 128.5, 128.1, 128.0, 126.4, 122.1, 118.0, 117.3, 100.1, 67.9, 66.4, 41.2, 37.8, 35.6, 34.4, 27.3. HRMS (ESI) $[M+Na]^+$ m/z calcd for $C_{26}H_{30}N_4O_8SNa$ 581.1682, found 581.1677.

Compound 5ag

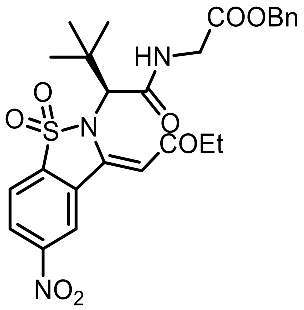

According to the general procedure, The crude residue was purified by flash column chromatography on silica gel (petroleum ether : ethyl acetate = 3:1; R_f = 0.41) to produce compound **5ag** (24 mg, 72% yield). ^{1}H NMR (400 MHz, $CDCl_3$) δ 10.22 (d, *J* = 1.8 Hz, 1H), 8.53 (dd, *J* = 8.5, 2.0 Hz, 1H), 8.06 (d, *J* = 8.5 Hz, 1H), 7.65 – 7.22 (m, 5H), 7.17 (t, *J* = 5.2 Hz, 1H), 6.61 (s, 1H), 5.09 (dd, *J* = 32.2, 12.2 Hz, 2H), 4.32 (s, 1H), 4.11 (dd, *J* = 18.1, 5.8 Hz, 1H), 3.94 (dd, *J* = 18.1, 5.1 Hz, 1H), 2.60 (qd, *J* = 7.3, 4.3 Hz, 2H), 1.29 (s, 9H), 1.14 (t, *J* = 7.3 Hz, 3H). ^{13}C NMR (100 MHz, $CDCl_3$) δ 199.4, 168.7, 166.8, 151.5, 138.2, 135.8, 135.0, 130.0, 128.6, 128.5, 128.3, 127.0, 125.1, 122.4, 109.8, 77.7 – 75.7, 67.2, 62.3, 41.4, 39.1, 36.7, 29.1, 8.4. HRMS (ESI) $[M+Na]^+$ m/z calcd for $C_{26}H_{29}N_3O_8SNa$ 566.1573, found 566.1558.

Compound 4o

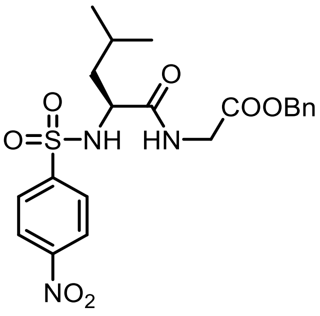

According to the general procedure, The crude residue was purified by flash column chromatography on silica gel (petroleum ether : ethyl acetate = 3:1; R_f = 0.43) to produce compound **4o** (1.4g, 82% yield). ^{1}H NMR (400 MHz, DMSO) δ 8.55 (t, *J* = 5.8 Hz, 1H), 8.45 (s, 1H), 8.41 – 8.31 (m, 2H), 8.10 – 7.92 (m, 2H), 7.46 – 7.24 (m, 5H), 5.20

– 4.99 (m, 2H), 3.87 (s, 1H), 3.69 (d, *J* = 5.8 Hz, 2H), 1.57 (dq, *J* = 13.1, 6.6 Hz, 1H), 1.45 – 1.18 (m, 2H), 0.81 (d, *J* = 6.6 Hz, 3H), 0.73 (d, *J* = 6.6 Hz, 3H). ^{13}C NMR (100 MHz, DMSO) δ 171.6, 169.8, 149.8, 147.1, 136.2, 128.9, 128.6, 128.4, 124.6, 66.3, 55.0, 42.0, 40.9, 40.0, 24.2, 23.2, 21.8. HRMS (ESI) $[M+Na]^+$ m/z calcd for $C_{21}H_{25}N_3O_7SNa$ 486.1311, found 486.1314.

Compound 5ok

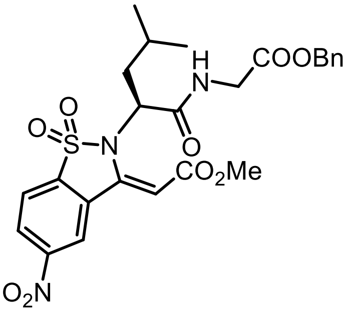

According to the general procedure, The crude residue was purified by flash column chromatography on silica gel (petroleum ether : ethyl acetate =4:1; R_f = 0.45) to produce compound **5ok** (1.4g, 78% yield). ^{1}H NMR (400 MHz, $CDCl_3$) δ 8.63 (d, *J* = 1.7 Hz, 1H), 8.54 (dd, *J* = 8.4, 1.8 Hz, 1H), 8.05 (d, *J* = 8.4 Hz, 1H), 7.58 – 7.31 (m, 5H), 6.97 (t, *J* = 4.7 Hz, 1H), 6.03 (s, 1H), 5.18 (s, 2H), 4.44 – 4.02 (m, 2H), 3.82 (s, 3H), 2.38 – 2.15 (m, 1H), 1.73 (s, 2H), 1.04 (d, *J* = 6.6 Hz, 3H), 0.99 (d, *J* = 6.6 Hz, 3H). ^{13}C NMR (100 MHz, $CDCl_3$) δ 169.3, 164.7, 151.2, 135.1, 132.9, 130.1 – 127.6, 126.9, 122.9, 117.5, 96.1, 81.6 – 74.7, 67.2, 52.5, 41.8, 25.8, 22.5. HRMS (ESI) $[M+Na]^+$ m/z calcd for $C_{25}H_{27}N_3O_9SNa$ 568.1366, found 568.1353.

Compound 4p

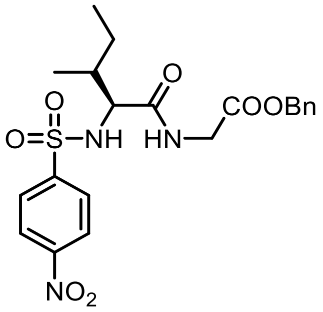

According to the general procedure, The crude residue was purified by flash column chromatography on silica gel (petroleum ether : ethyl acetate =5:1; R_f = 0.41) to produce compound **4p** (1.3g, 78% yield). ^{1}H NMR (400 MHz, DMSO) δ 8.48 (t, *J* = 5.8 Hz, 1H), 8.40 – 8.28 (m, 3H), 8.08 – 7.89 (m, 2H), 7.39 – 7.24 (m, 5H), 5.07 (d, *J* = 0.7 Hz, 2H), 3.74 – 3.52 (m, 3H), 1.68 – 1.55 (m, 1H), 1.49 (ddd, *J* = 13.4, 7.5, 3.3 Hz, 1H), 1.20 – 1.03 (m, 1H), 0.77 (dd, *J* = 14.1, 7.1 Hz, 6H). ^{13}C NMR (100 MHz, DMSO) δ 170.6, 169.7, 149.8, 147.1, 136.2, 124.5, 66.3, 61.0, 40.8, 40.0, 37.2, 24.6, 15.4, 10.9. HRMS (ESI) $[M+Na]^+$ m/z calcd for $C_{21}H_{25}N_3O_7SNa$ 486.1311, found 486.1315.

Compound 5pk

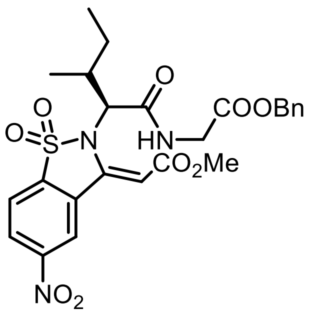

According to the general procedure, The crude residue was purified by flash column chromatography on silica gel (petroleum ether : ethyl acetate =4:1; R_f = 0.42) to produce compound **5pk** (31 mg, 72% yield). ^{1}H NMR (400 MHz, $CDCl_3$) δ 8.59 (d, *J* = 1.7 Hz, 1H), 8.52 (dd, *J* = 8.5, 1.8 Hz, 1H), 8.02 (d, *J* = 8.4 Hz, 1H), 7.71 (s, 1H), 7.51 – 7.28 (m, 5H), 5.89 (s, 1H), 5.37 (d, *J* = 11.2 Hz, 1H), 5.14 (d, *J* = 6.1 Hz, 2H), 4.21 (dd, *J* = 18.4, 5.5 Hz, 1H), 4.15 – 4.02 (m, 1H), 3.85 (s, 3H), 1.10 (d, *J* = 6.4 Hz, 1H), 0.98 (d, *J* = 6.5 Hz, 3H), 0.90 (dt, *J* = 14.6, 7.3 Hz, 5H). ^{13}C NMR (100 MHz, CD_3CN) δ 169.6 – 169.0, 167.7, 128.5, 128.2, 128.0, 127.7, 122.4, 118.5, 117.34 (s), 95.2, 68.3, 66.5, 52.2, 41.3, 34.3, 25.4, 15.4, 10.3. HRMS (ESI) $[M+Na]^+$ m/z calcd for $C_{25}H_{27}N_3O_9SNa$ 568.1366, found 568.1363.

Compound 4q

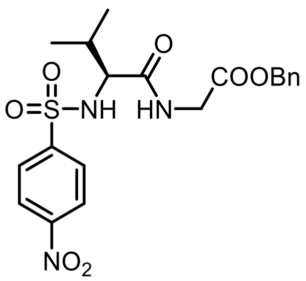

According to the general procedure, The crude residue was purified by flash column chromatography on silica gel (petroleum ether : ethyl acetate =5:1; R_f = 0.46) to produce compound **4q** (1.4 g, 78% yield). ^{1}H NMR (400 MHz, DMSO) δ 8.46 (t, *J* = 5.8 Hz, 1H), 8.35 (dq, *J* = 9.4, 2.3 Hz, 3H), 8.04 – 7.92 (m, 2H), 7.43 – 7.22 (m, 5H), 5.16 – 4.97 (m, 2H), 3.67 (d, *J* = 5.8 Hz, 2H), 3.62 (dd, *J* = 9.3, 7.3 Hz, 1H), 1.84 (dq, *J* = 13.7, 6.8 Hz, 1H), 0.81 (d, *J* = 6.8 Hz, 6H). ^{13}C NMR (100 MHz, DMSO) δ 170.7, 169.8, 149.8, 147.1, 136.2, 128.9, 128.6, 128.5, 124.6, 66.3, 62.3, 44.5 – 37.3, 31.3, 19.4, 18.7. HRMS (ESI) $[M+Na]^+$ m/z calcd for $C_{20}H_{23}N_3O_7SNa$ 472.1154, found 472.1151.

Compound 5qk

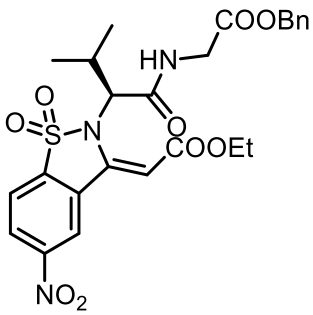

According to the general procedure, The crude residue was purified by flash column chromatography on silica gel (petroleum ether : ethyl acetate =4:1; R_f = 0.46) to produce compound **5qk** (28 mg, 77% yield). ^{1}H NMR (400 MHz, CD_3CN) δ 8.78 (d, *J* = 1.7 Hz, 1H), 8.51 (dd, *J* = 8.5, 1.9 Hz, 1H), 8.13 – 7.95 (m, 1H), 7.55 (s, 1H), 7.46 – 7.22 (m, 5H), 6.20 (s, 1H), 5.12 (d, *J* = 1.2 Hz, 2H), 5.07 (s, 1H), 4.08 (dd, *J* = 17.8, 5.7 Hz, 1H), 3.95 (dd, *J* = 17.8, 5.6 Hz, 1H), 3.81 (s, 3H), 0.97 (d, *J* = 6.5 Hz, 3H), 0.91 (d, *J* = 6.7 Hz, 3H). ^{13}C NMR (100 MHz, CD_3CN) δ 169.9, 168.3, 166.3, 152.2, 142.5, 136.6, 133.0, 129.0, 128.7, 128.6, 128.2, 123.0, 119.0, 96.2, 70.1, 67.0, 52.7, 41.9, 29.1, 20.1, 19.4. HRMS (ESI) $[M+Na]^+$ m/z calcd for

$C_{24}H_{25}N_3O_9SNa$ 554.1209, found 554.1203.

Compound 4r

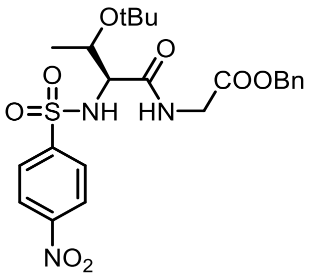

According to the general procedure, The crude residue was purified by flash column chromatography on silica gel (petroleum ether : ethyl acetate =5:1; R_f = 0.43) to produce compound **4r** (1.6g, 83% yield). ^{1}H NMR (400 MHz, $CDCl_3$) δ 8.46 – 8.30 (m, 2H), 8.13 – 7.94 (m, 2H), 7.66 (t, *J* = 5.0 Hz, 1H), 7.47 – 7.31 (m, 5H), 6.13 (d, *J* = 4.8 Hz, 1H), 5.22 – 5.11 (m, 2H), 4.18 (qd, *J* = 6.5, 4.2 Hz, 1H), 4.05 (dd, *J* = 18.4, 5.0 Hz, 1H), 3.97 (dd, *J* = 18.4, 5.4 Hz, 1H), 3.63 (t, *J* = 4.4 Hz, 1H), 1.28 (s, 9H), 1.12 (d, *J* = 6.4 Hz, 3H). ^{13}C NMR (100 MHz, $CDCl_3$) δ 168.9, 168.0, 150.2, 144.8, 134.9, 128.7, 128.5, 124.5, 76.1, 68.7, 67.3 (, 59.4, 41.7, 28.0, 16.4. HRMS (ESI) $[M+Na]^+$ m/z calcd for $C_{23}H_{29}N_3O_8SNa$ 530.1573, found 530.1570.

Compound 5rk

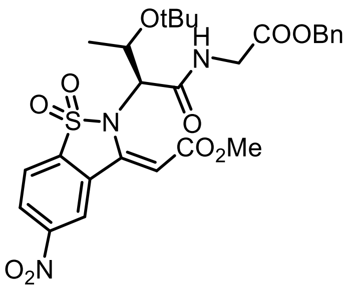

According to the general procedure, The crude residue was purified by flash column chromatography on silica gel (petroleum ether : ethyl acetate =4:1; R_f = 0.42) to produce compound **5rk** (28 mg, 75% yield). ^{1}H NMR (400 MHz, CD_3CN) δ 8.79 (d, *J* = 1.7 Hz, 1H), 8.49 (dd, *J* = 8.5, 1.9 Hz, 1H), 8.13 – 8.00 (m, 1H), 7.77 (d, *J* = 5.1 Hz, 1H), 7.47 – 7.24 (m, 5H), 6.25 (s, 1H), 5.22 – 5.05 (m, 3H), 4.68 (dd, *J* = 8.6, 5.9 Hz, 1H), 4.15 (dd, *J* = 17.8, 5.8 Hz, 1H), 3.99 (dd, *J* = 17.8, 5.5 Hz, 1H), 3.80 (s, 3H), 1.20 (d, *J* = 5.9 Hz, 3H), 0.90 (s, 9H). ^{13}C NMR (100 MHz, CD_3CN) δ 169.9, 167.3, 166.3, 152.1, 144.9, 138.2, 136.6, 134.2, 129.1, 128.8, 128.7, 128., 122.8, 118.7, 117.9, 97.5, 75.0, 69.4, 67.1, 52.6, 41.9, 28.6, 21.5. HRMS (ESI) $[M+Na]^+$ m/z calcd for $C_{27}H_{31}N_3O_{10}SNa$ 612.1628, found 612.1625.

Compound 4s

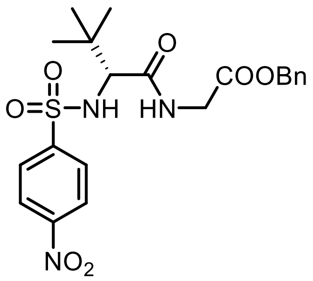

According to the general procedure, The crude residue was purified by flash column chromatography on silica gel (petroleum ether : ethyl acetate =5:1; R_f = 0.45) to produce compound **4s** (1.2g, 78% yield). ^{1}H NMR (400 MHz, $CDCl_3$) δ 8.41 – 8.18 (m, 2H), 8.12 – 7.86 (m, 2H), 7.53 – 7.29 (m, 5H), 5.99 (t, *J* = 4.9 Hz, 1H), 5.76 (d, *J* = 9.8 Hz, 1H), 5.13 (q, *J* = 12.1 Hz, 2H), 3.94 – 3.63 (m, 2H), 3.48 (d, *J* = 9.8 Hz, 1H), 0.98 (s, 9H). ^{13}C NMR (100 MHz, $CDCl_3$) δ 169.2, 169.0, 150.1, 145.5, 134.7, 128.7, 124.2, 67.7, 65.3, 41.1, 34.8, 26.5. HRMS (ESI) $[M+Na]^+$ m/z calcd for $C_{21}H_{25}N_3O_7SNa$ 486.1311, found 486.1304.

Compound 5sk

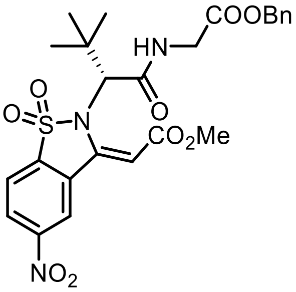

According to the general procedure, The crude residue was purified by flash column chromatography on silica gel (petroleum ether : ethyl acetate =6:1; R_f = 0.45) to produce compound **5sk** (32 mg, 71% yield). ^{1}HNMR (400 MHz, CD_3CN) δ 8.79 (d, *J* = 1.8 Hz, 1H), 8.53 (dd, *J* = 8.5, 1.9 Hz, 1H), 8.11 (d, *J* = 8.5 Hz, 1H), 7.51 – 7.27 (m, 5H), 6.91 (s, 1H), 6.24 (s, 1H), 5.80 (s, 1H), 5.31 – 5.00 (m, 2H), 4.10 (dd, *J* = 17.7, 5.9 Hz, 1H), 3.90 (dd, *J* = 17.7, 5.5 Hz, 1H), 3.82 (s, 3H), 1.23 (s, 9H). ^{13}C NMR (100 MHz, CD_3CN) δ 169.5, 167.3, 165.5, 151.4, 142.4, 137.9 – 137.0, 136.0, 132.8, 128.5, 128.2, 128.1, 127.5, 122.3, 118.4, 96.3, 71.5, 66.5, 52.2, 41.2, 36.0, 27.8. HRMS (ESI) $[M+Na]^+$ m/z calcd for $C_{25}H_{27}N_3O_9SNa$ 568.1366, found 568.1363.

Compound 4t

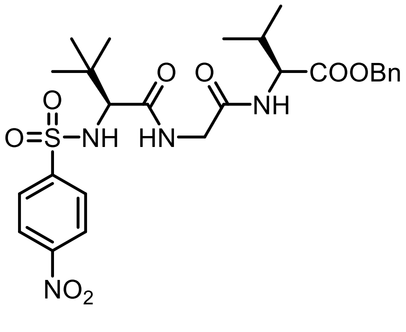

According to the general procedure, The crude residue was purified by flash column chromatography on silica gel (petroleum ether : ethyl acetate =6:1; R_f = 0.46) to produce compound **4t** (1.1 g, 72% yield). ^{1}H NMR (400 MHz, DMSO) δ 8.38 – 8.29 (m, 2H), 8.22 – 8.04 (m, 3H), 8.04 – 7.97 (m, 2H), 7.43 – 7.30 (m, 4H), 5.22 – 5.06 (m, 2H), 4.20 (dd, *J* = 8.1, 6.1 Hz, 1H), 3.67 (d, *J* = 10.1 Hz, 1H), 3.55 (dd, *J* = 16.7, 5.8 Hz, 1H), 3.43 – 3.28 (m, 2H), 2.51 (dd, *J* = 3.5, 1.7 Hz, 1H), 2.02 (dq, *J* = 13.5, 6.8 Hz, 1H), 0.90 (s, 9H), 0.82 (d, *J* = 6.8 Hz, 6H). ^{13}C NMR (100 MHz, DMSO) δ 171.6, 169.1, 149.7, 146.9, 136.2, 128.8, 128.5, 124.3, 66.3, 64.5, 57., 41.4, 34.4, 30.4, 26.8, 19.2, 18.4. ^{1}HRMS (ESI) $[M+Na]^+$ m/z calcd for $C_{26}H_{34}N_4O_8SNa$ 585.1995, found 585.1983.

Compound 5tk

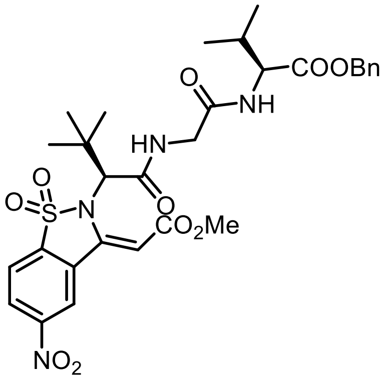

According to the general procedure, The crude residue was purified by flash column chromatography on silica gel (petroleum ether : ethyl acetate =3:1; R_f = 0.36) to produce compound **5tk** (22 mg, 65% yield). ^{1}H NMR (400 MHz, CD_3CN) δ 9.35 (d, *J* = 7.7 Hz, 1H), 8.33 (d, *J* = 2.7 Hz, 1H), 8.25 (dd, *J* = 9.0, 2.7 Hz, 1H), 7.49 (d, *J* = 9.0 Hz, 1H), 7.45 – 7.27 (m, 5H), 6.87 (d, *J* = 8.4 Hz, 1H), 5.15 (q, *J* = 12.4 Hz, 2H), 4.84 (s, 1H), 4.64 (d, *J* = 16.6 Hz, 1H), 4.39 (dd, *J* = 8.5, 5.5 Hz, 1H), 4.30 (d, *J* = 16.6 Hz, 1H), 3.65 (d, *J* = 5.1 Hz, 3H), 3.63 (d, *J* = 7.8 Hz, 1H), 2.13 – 2.08 (m, 1H), 1.13 (s, 9H), 0.92 – 0.83 (m, 6H). ^{13}C NMR (100 MHz, CD_3CN) δ 171.2, 170.6, 169.0, 167.9, 158.4, 147.1, 145.0, 136.1, 131.2, 130.5, 128.5, 128.2, 126.0, 124.4, 123.9, 84.2, 66.5, 60.0, 57.8, 51.5, 50.2, 32.4, 30.7, 25.6, 18.3, 17.1. HRMS (ESI) $[M+H]^+$ m/z calcd for $C_{30}H_{37}N_4O_{10}S$ 645.2230, found 645.2212; $[M+Na]^+$ m/z calcd for $C_{30}H_{36}N_4O_{10}SNa$ 667.2050, found 667.2034.

Compound 4u

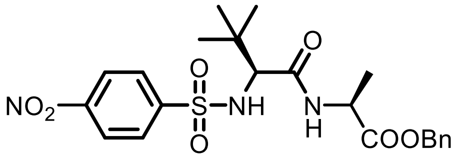

According to the general procedure, The crude residue was purified by flash column chromatography on silica gel (petroleum ether : ethyl acetate =3:1; R_f = 0.39) to produce compound **4u** (1.3 g, 85% yield).^{1}H NMR (500 MHz, $CDCl_3$) δ 8.29 (d, *J* = 8.8 Hz, 2H), 8.03 (d, *J* = 8.8 Hz, 2H), 7.42 – 7.23 (m, 5H), 6.22 (s, 1H), 5.95 (dd, *J* = 9.7, 3.6 Hz, 1H), 5.16 (dd, *J* = 41.1, 12.2 Hz, 2H), 4.38 (t, *J* = 7.2 Hz, 1H), 3.49 (d, *J* = 9.8 Hz, 1H), 1.05 (d, *J* = 7.2 Hz, 3H), 0.98 (s, 9H). ^{13}C NMR (125 MHz, $CDCl_3$) δ 172.1, 168.6, 150.0, 145.8, 134.8, 128.8 – 128.5, 128.3, 124.2, 67.6, 65.1, 48.1, 34.9, 26.5, 18.1. $[M+Na]^+$ m/z calcd for $C_{22}H_{27}N_3O_7SNa$ 500.1467, found 500.1474.

Compound 5uk

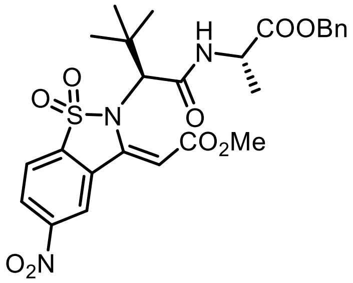

According to the general procedure, The crude residue was purified by flash column chromatography on silica gel (petroleum ether : ethyl acetate =4:1; R_f = 0.41) to produce compound **5uk** (33 mg, 85% yield). ^{1}H NMR (500 MHz, CD_3CN) δ 8.78 (d,

J = 1.7 Hz, 1H), 8.53 (dd, J = 8.5, 1.8 Hz, 1H), 8.11 (d, J = 8.5 Hz, 1H), 7.45 – 7.32 (m, 5H), 6.98 (d, J = 6.6 Hz, 1H), 6.25 (s, 1H), 5.62 (s, 1H), 5.22 – 5.06 (m, 2H), 4.55 (t, J = 7.2 Hz, 1H), 3.83 (s, 3H), 1.37 (d, J = 7.2 Hz, 3H), 1.18 (s, 9H). ^{13}C NMR (125 MHz, CD_3CN) δ 172.3, 166.4, 165.5, 151.4, 142.6, 136.1, 132.9, 128.5, 128.2, 128.0, 127.5, 122.2, 118.3, 117.3, 96.8, 71.7, 66.6, 52.2, 48.3, 36.3, 27.8, 16.8. $[M+Na]^+$ m/z calcd for $C_{26}H_{29}N_3O_9SNa$ 582.1522, found 582.1531.

Compound 4v

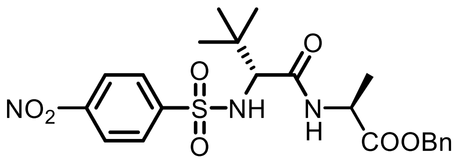

According to the general procedure, The crude residue was purified by flash column chromatography on silica gel (petroleum ether : ethyl acetate =5:1; R_f = 0.43) to produce compound **4v** (1.5 g, 88% yield).^{1}H NMR (500 MHz, $CDCl_3$) δ 8.26 (d, J = 8.9 Hz, 2H), 8.00 (d, J = 8.9 Hz, 2H), 7.51 – 7.32 (m, 5H), 5.96 (d, J = 7.3 Hz, 1H), 5.64 (d, J = 9.8 Hz, 1H), 5.14 (dd, J = 84.0, 12.1 Hz, 1H), 4.18 (p, J = 7.2 Hz, 1H), 3.40 (d, J = 9.8 Hz, 1H), 1.29 (d, J = 7.1 Hz, 3H), 1.01 (s, 9H).^{13}C NMR (125 MHz, $CDCl_3$) δ 172.0, 168.0, 150.0, 145.2, 134.8, 129.1 – 128.6, 128.3, 124.2, 67.7, 65.2, 47.9, 34.8, 26.5, 18.4. $[M+Na]^+$ m/z calcd for $C_{22}H_{27}N_3O_7SNa$ 500.1467, found 500.1474.

Compound 5vk

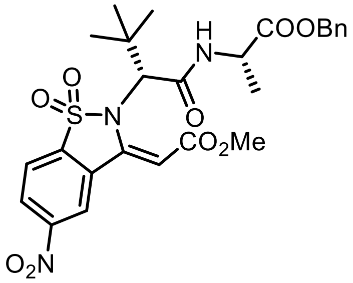

According to the general procedure, The crude residue was purified by flash column chromatography on silica gel (petroleum ether : ethyl acetate =4:1; R_f = 0.46) to produce compound **5vk** (33 mg, 82% yield). ^{1}H NMR (500 MHz, CD_3CN) δ 8.77 (d, J = 1.7 Hz, 1H), 8.52 (dd, J = 8.5, 1.8 Hz, 1H), 8.09 (d, J = 8.5 Hz, 1H), 7.39 – 7.29 (m, 5H), 7.06 (d, J = 6.9 Hz, 1H), 6.23 (s, 1H), 5.72 (s, 1H), 5.09 (s, 2H), 4.46 (dd, J = 14.4, 7.2 Hz, 1H), 3.82 (s, 3H), 1.38 (d, J = 7.2 Hz, 3H), 1.23 (s, 9H). ^{13}C NMR (125 MHz, CD_3CN) δ 172.1, 166.6, 165.5, 151.4, 142.3, 137.3, 137.3, 136.0, 132.8, 128.42, 128.1, 127.9, 127.5, 127.0, 120.5, 118.3, 117.3, 96.5, 71.6, 66.5, 52.2, 48.5, 35.9, 27.9. $[M+Na]^+$ m/z calcd for $C_{26}H_{29}N_3O_9SNa$ 582.1522, found 582.1531.

Compound 8a

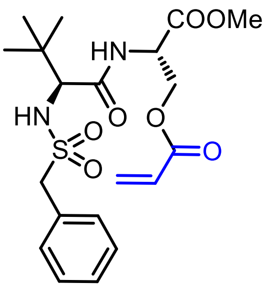

According to the general procedure, the crude residue was purified by flash column chromatography on silica gel (petroleum ether: ethyl acetate= 2:1; R_f = 0.39) to produce compound **8a** (0.45 g, 92% yield)^{1}H NMR (400 MHz, CD_3CN) δ 7.37 (s, 1H), 7.30 (d, *J* = 7.4 Hz, 1H), 6.30 (dd, *J* = 17.3, 1.4 Hz, 1H), 6.03 (dd, *J* = 17.3, 10.5 Hz, 1H), 5.82 (dd, *J* = 10.5, 1.4 Hz, 1H), 5.70 (d, *J* = 9.7 Hz, 1H), 4.80 (ddd, *J* = 7.6, 5.2, 3.8 Hz, 1H), 4.46 (qd, *J* = 11.6, 4.5 Hz, 1H), 4.22 – 4.12 (m, 1H), 3.77 (d, *J* = 9.7 Hz, 1H), 3.70 (s, 1H), 1.00 (s, 9H).^{13}C NMR (100 MHz, CD_3CN) δ 170.9, 169.9, 165.9, 131.9, 131.5, 130.2, 129.0, 128.9, 128.2, 64.8, 63.7, 59.2, 52.8, 52.3, 34.9, 26.4. HRMS (ESI) $[M+Na]^+$ m/z calcd for $C_{20}H_{28}N_2O_7SNa$ 463.1515, found 463.1516

Compound 9a

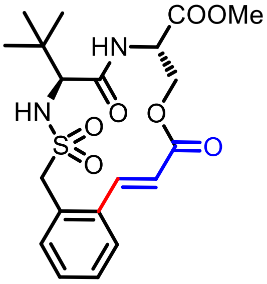

According to the general procedure, the crude residue was purified by flash column chromatography on silica gel (petroleum ether: ethyl acetate= 2:1; R_f = 0.30) to produce compound **9a** (11 mg, 53% yield). ^{1}H NMR (400 MHz, CD_3CN) δ 8.06 (d, *J* = 15.9 Hz, 1H), 7.76 (dt, *J* = 6.7, 2.9 Hz, 1H), 7.52 – 7.33 (m, 3H), 7.01 (d, *J* = 9.7 Hz, 1H), 6.32 (d, *J* = 15.9 Hz, 1H), 5.72 (d, *J* = 8.2 Hz, 1H), 5.22 (ddd, *J* = 11.8, 9.8, 4.1 Hz, 1H), 4.82 (d, *J* = 13.9 Hz, 1H), 4.70 (dd, *J* = 11.1, 4.2 Hz, 1H), 4.34 (d, *J* = 14.0 Hz, 1H), 4.05 (dd, *J* = 11.6, 11.2 Hz, 1H), 3.90 (d, *J* = 8.3 Hz, 1H), 3.73 (s, 3H), 1.07 (s, 9H) .^{13}C NMR (100 MHz, CD3CN) δ 170.9, 169.7, 165.8, 144.1, 134.8, 133.3, 130.9, 130.2, 129.6, 126.7, 118.6, 66.8, 62.9, 57.6, 52.7, 51.4, 34.8, 26.9. HRMS (ESI) $[M+Na]^+$ m/z calcd for $C_{20}H_{26}N_2O_7SNa$ 461.1358, found 463.1359

Compound 8b

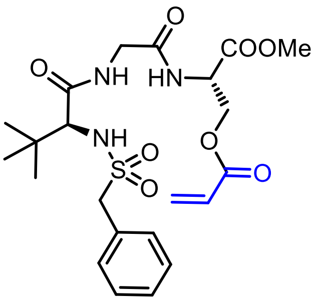

According to the general procedure, the crude residue was purified by flash column chromatography on silica gel (petroleum ether: ethyl acetate= 1:1; R_f= 0.33) to produce compound **8b** (630 mg, 86% yield). ^{1}H NMR (500 MHz, CD_3CN) δ 7.52 – 7.34 (m, 5H), 7.16 (m, 1H), 7.07 (m, 1H), 6.38 (dd, *J* = 17.3, 1.4 Hz, 1H), 6.13 (dd, *J* = 17.3, 10.5 Hz, 1H), 5.92 (dd, *J* = 10.5, 1.3 Hz, 1H), 5.67 (m, 1H), 4.76 (tt, *J* = 8.1, 4.1 Hz, 1H), 4.44 –

4.35 (m, 2H), 4.32 – 4.24 (m, 2H), 3.91 (ddd, *J* = 14.3, 5.8, 2.5 Hz, 2H), 3.72 – 3.69 (m, 1H), 3.68 (s, 3H), 1.01 (s, 9H).^{13}C NMR (125 MHz, CD_3CN) δ 170.8, 169.6, 168.9, 165.4, 131.4, 130.9, 129.7, 128.4, 128.3, 127.8, 64.8, 63.4, 58.5, 52.2, 51.5, 42.1, 39.4, 36.9, 34.1, 25.9. HRMS (ESI) $[M+Na]^+$ m/z calcd for $C_{22}H_{31}N_3O_8SNa$ 520.1730, found 520.1730

Compound 9b

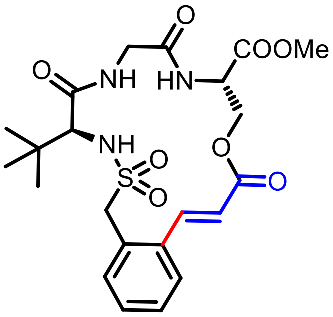

According to the general procedure, the crude residue was purified by flash column chromatography on silica gel (petroleum ether: ethyl acetate= 1:1; R_f= 0.39) to produce compound **9b** (9 mg, 57% yield).^{1}H NMR (500 MHz, CD_3CN) δ 8.09 (d, *J* = 15.8 Hz, 1H), 7.74 (d, *J* = 7.1 Hz, 1H), 7.53 – 7.43 (m, 3H), 7.30 (d, *J* = 7.3 Hz, 1H), 7.23 – 7.14 (m, 1H), 6.38 (d, *J* = 15.8 Hz, 1H), 5.71 (d, *J* = 6.8 Hz, 1H), 4.82 (ddd, *J* = 7.5, 6.3, 3.3 Hz, 1H), 4.64 (d, *J* = 14.1 Hz, 1H), 4.56 (dd, *J* = 11.3, 6.2 Hz, 1H), 4.49 (d, *J* = 14.1 Hz, 1H), 4.42 (dd, *J* = 11.3, 3.3 Hz, 1H), 4.24 (dd, *J* = 16.5, 8.1 Hz, 1H), 3.87 (d, *J* = 6.8 Hz, 1H), 3.73 (s, 3H), 3.70 (d, *J* = 4.1 Hz, 1H), 1.07 (s, 9H).^{13}C NMR (125 MHz, CD_3CN) δ 170.7, 169.2, 168.9, 165.1, 142.4, 134.7, 133.4 130.2 129.1, 128.8, 127.0, 120.3, 64.7, 63.4, 56.8, 52.3, 52.0, 42.7, 34.0, 26.2. HRMS (ESI) $[M+Na]^+$ m/z calcd for $C_{22}H_{29}N_3O_8SNa$ 518.1573, found 518.1538

Compound 8c

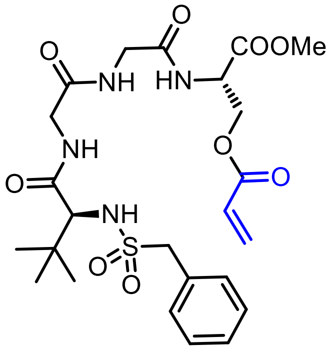

According to the general procedure, the crude residue was purified by flash column chromatography on silica gel (petroleum ether: ethyl acetate= 1:2; R_f= 0.30) to produce compound **8c** (610 mg, 82% yield)^{1}H NMR (500 MHz, CD_3CN) δ 7.42 – 7.34 (m, 5H), 7.30 – 7.21 (m, 2H), 7.11 (t, *J* = 5.6 Hz, 1H), 6.37 (dd, *J* = 17.3, 1.2 Hz, 1H), 6.11 (dd, *J* = 17.3, 10.5 Hz, 1H), 5.89 (dd, *J* = 10.5, 1.2 Hz, 1H), 5.78 (d, *J* = 9.1 Hz, 1H), 4.75 (dt, *J* = 8.1, 4.7 Hz, 1H), 4.40 – 4.38 (m, 2H), 4.30 (q, *J* = 13.8 Hz, 2H), 3.89 (dd, *J* = 16.6, 5.7 Hz, 1H), 3.84 – 3.77 (m, 3H), 3.70 (d, *J* = 8.7 Hz, 1H), 3.69 (s, 3H), 0.98 (s, 9H).^{13}C NMR (125 MHz, CD_3CN) δ 171.4, 169.8, 169.2, 169.1, 165.5, 131.5, 130.9, 129.6, 128.5, 128.3, 127.8, 64.5, 63.4, 58.4, 52.3, 51.5, 42.7, 42.2, 34.1, 25.9. HRMS (ESI) $[M+Na]^+$ m/z calcd for $C_{24}H_{34}N_4O_9SNa$ 577.1944, found 577.1943

Compound 9c

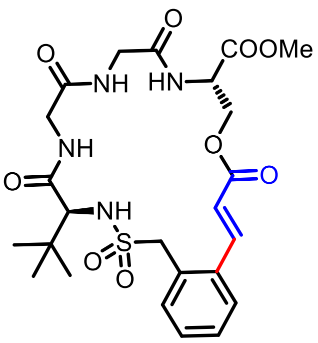

According to the general procedure, the crude residue was purified by flash column chromatography on silica gel (petroleum ether: ethyl acetate= 1:2; R_f= 0.25) to produce compound **9c** (9 mg, 45% yield)^{1}H NMR (500 MHz, CD_3CN) δ 8.20 (d, *J* = 15.9 Hz, 1H), 7.84 – 7.78 (m, 1H), 7.57 (d, *J* = 6.2 Hz, 1H), 7.51 – 7.44 (m, 3H), 7.27 (dd, *J* = 19.1, 7.8 Hz, 2H), 6.40 (d, *J* = 15.9 Hz, 1H), 6.04 (d, *J* = 6.7 Hz, 1H), 4.96 (ddd, *J* = 10.4, 9.0, 4.1 Hz, 1H), 4.78 (dd, *J* = 11.3, 4.1 Hz, 1H), 4.66 (s, 2H), 4.45 – 4.38 (m, 1H), 4.01 – 3.88 (m, 2H), 3.82 (d, *J* = 4.4 Hz, 1H), 3.81 – 3.77 (m, 1H), 3.76 (s, 3H), 3.53 (dd, *J* = 17.2, 5.6 Hz, 1H), 1.09 (s, 9H).^{13}C NMR (125 MHz, CD_3CN) δ 172.1, 169.5, 169.3, 165.7, 143.0, 134.6, 133.3, 130.4, 129.3, 128.5, 126.8, 118.9, 66.5, 61.6, 54.9, 52.3, 51.1, 42.9, 42.3, 33.8, 26.0. HRMS (ESI) $[M+Na]^+$ m/z calcd for $C_{24}H_{32}N_4O_9SNa$ 575.1788, found 575.1755

Compound 8d

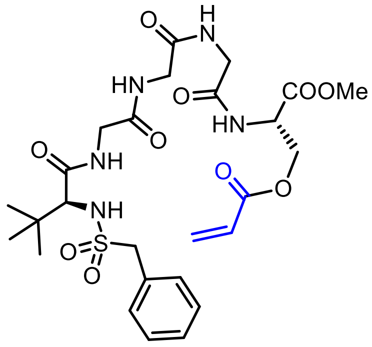

According to the general procedure, the crude residue was purified by flash column chromatography on silica gel (methanol : ethyl acetate= 1: 20; R_f= 0.39) to produce compound **8d** (410 mg, 68% yield).^{1}H NMR (500 MHz, CD_3CN) δ 7.47 – 7.38 (m, 5H), 7.32 (d, *J* = 3.9 Hz, 1H), 7.22 (dt, *J* = 17.1, 7.5 Hz, 3H), 6.40 (dd, *J* = 17.3, 1.3 Hz, 1H), 6.15 (dd, *J* = 17.3, 10.5 Hz, 1H), 5.93 (dd, *J* = 10.5, 1.3 Hz, 1H), 5.85 (d, *J* = 7.3 Hz, 1H), 4.81 – 4.72 (m, 1H), 4.43 (d, *J* = 4.8 Hz, 1H), 4.40 (t, *J* = 4.6 Hz, 1H), 4.34 (s, 2H), 3.91 (m, 2H), 3.85 – 3.76 (m, 4H), 3.73 (m, 4H), 1.00 (s, 9H). ^{13}C NMR (125 MHz, CD_3CN) δ 171.8, 169.7, 169.7, 169.4, 169.4, 169.3, 165.5, 131.4, 131.0, 129.6, 128.5, 128.4, 127.8, 64.4, 63.4, 63.3, 58.4, 52.3, 52.3, 51.5, 51.4, 42.9, 42.7, 42.7, 42.2, 42.1, 39.4, 37.0, 34.0, 25.9. HRMS (ESI) $[M+Na]^+$ m/z calcd for $C_{26}H_{37}N_5O_{10}SNa$ 634.2159, found 634.2151

Compound 9d

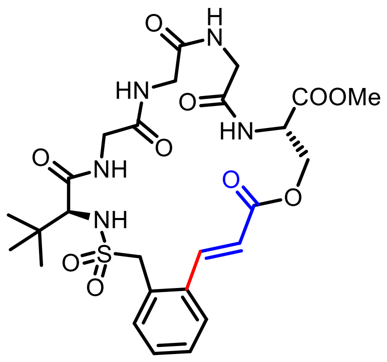

According to the general procedure, the crude residue was purified by flash column chromatography on silica gel (methanol: ethyl acetate= 1:20; R_f= 0.25) to produce compound **9d** (11 mg, 34% yield)^{1}H NMR (500 MHz, CD_3CN) δ 8.12 (d, *J* = 15.9 Hz, 1H), 7.77 (d, *J* = 7.1 Hz, 1H), 7.72 (d, *J* = 6.1 Hz, 1H), 7.42 (m, 4H), 7.38 – 7.33 (m, 1H), 7.31 (m, 1H), 6.40 (d, *J* = 15.9 Hz, 1H), 6.02 (d, *J* = 10.5 Hz, 1H),

5.17 (ddd, *J* = 8.5, 4.2, 2.3 Hz, 1H), 4.69 (d, *J* = 13.6 Hz, 1H), 4.53 (dd, *J* = 11.3, 4.5 Hz, 1H), 4.45 (dd, *J* = 17.1, 7.6 Hz, 1H), 4.37 (dd, *J* = 11.3, 2.3 Hz, 1H), 4.19 (d, *J* = 13.6 Hz, 1H), 4.13 (dd, *J* = 17.4, 7.4 Hz, 1H), 3.96 (d, *J* = 10.6 Hz, 1H), 3.89 (s, 3H), 3.79 (dd, *J* = 17.1, 2.4 Hz, 1H), 3.67 (dd, *J* = 17.1, 5.1 Hz, 2H), 3.60 (dd, *J* = 16.8, 6.4 Hz, 1H), 1.07 (s, 9H). ^{13}C NMR (125 MHz, CD_3CN) δ 171.2, 171.0, 170.5, 169.2, 168.9, 165.4, 142.2, 134.6, 133.8, 130.2 129.2, 129.0, 126.9, 119.7, 100.0, 65.3, 64.8, 55.9, 53.0, 51.3, 43.5, 42.0, 41.7, 33.9, 25.7. HRMS (ESI) $[M+Na]^+$ m/z calcd for $C_{26}H_{35}N_5O_{10}SNa$ 632.2002, found 632.1999

Compound 8e

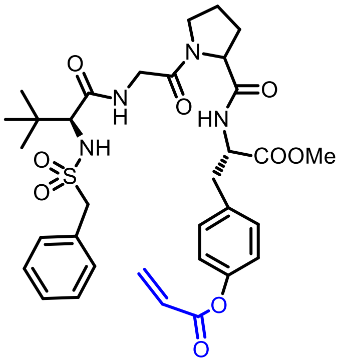

According to the general procedure, the crude residue was purified by flash column chromatography on silica gel (petroleum ether: ethyl acetate= 1:1; R_f = 0.39) to produce compound **8e** (390 mg, 72% yield) ^{1}H NMR (500 MHz, $CDCl_3$) δ 7.43 – 7.36 (m, 2H), 7.33 (d, *J* = 3.0 Hz, 3H), 7.24 (d, *J* = 7.6 Hz, 1H), 7.13 (d, *J* = 8.4 Hz, 2H), 7.02 (d, *J* = 8.4 Hz, 2H), 6.86 (m, 1H), 6.60 (d, *J* = 17.3 Hz, 1H), 6.30 (dd, *J* = 17.3, 10.5 Hz, 1H), 6.01 (d, *J* = 10.5 Hz, 1H), 5.60 – 5.36 (m, 1H), 4.83 (dd, *J* = 13.4, 7.5 Hz, 1H), 4.52 (d, *J* = 8.0 Hz, 1H), 4.27 – 4.20 (m, 2H), 4.12 (dd, *J* = 17.5, 5.0 Hz, 1H), 3.85 (d, *J* = 17.5 Hz, 1H), 3.75 (d, *J* = 9.4 Hz, 1H), 3.71 (s, 3H), 3.48 – 3.40 (m, 1H), 3.35 (dd, *J* = 17.3, 8.7 Hz, 1H), 3.16 (dd, *J* = 14.0, 3.6 Hz, 1H), 2.99 (dd, *J* = 14.1, 7.3 Hz, 1H), 2.27 (m, 1H), 2.04 – 1.94 (m, 2H), 1.89 – 1.80 (m, 1H), 1.01 (s, 9H). ^{13}C NMR (125 MHz, $CDCl_3$) δ 171.8, 170.5, 167.5, 164.8, 149.5, 133.8, 133.1, 130.8, 130.2, 128.6, 127.7, 121.4, 65.0, 59.9, 59.5, 52.9, 52.4, 46.3, 42.0, 37.0, 34.8, 27.5, 26.6 24.7. HRMS (ESI) $[M+Na]^+$ m/z calcd for $C_{33}H_{42}N_4O_9SNa$ 693.2570, found 639.2567

Compound 9e

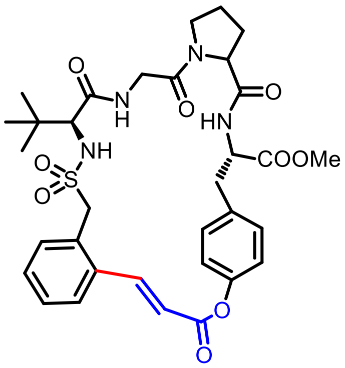

According to the general procedure, the crude residue was purified by flash column chromatography on silica gel (petroleum ether: ethyl acetate= 1:1; R_f = 0.45) to produce compound **9e** (11 mg, 72% yield) ^{1}H NMR (500 MHz, CD_3CN) δ 8.29 (d, *J* = 15.8 Hz, 1H), 7.91 – 7.86 (m, 1H), 7.55 – 7.48 (m, 3H), 7.44 (d, *J* = 7.3 Hz, 1H), 7.11 (dd, *J* = 43.0, 8.5 Hz, 4H), 6.62 (d, *J* = 15.8 Hz, 1H), 6.45 (d, *J* = 8.0 Hz, 1H), 5.91 (m, 1H), 4.73 (m, 1H), 4.55 (m, 3H), 4.39 (d, *J* = 13.8 Hz, 1H), 3.89 (d, *J* = 10.0 Hz, 1H),

3.80 (s, 3H), 3.51 (dd, J = 17.8, 1.7 Hz, 2H), 3.41 (dd, J = 17.7, 9.1 Hz, 1H), 3.24 (dd, J = 13.8, 5.6 Hz, 1H), 3.14 (dd, J = 13.8, 4.9 Hz, 1H), 2.34 (dt, J = 12.0, 3.7 Hz, 1H), 2.03 (dd, J = 8.3, 4.0 Hz, 2H), 1.84 – 1.74 (m, 1H), 1.06 (s, 9H).^{13}C NMR (126 MHz, CD_3CN) δ 171.4, 170.3, 170.0, 168.5, 165.4, 149.9, 143.8, 134.3, 134.1, 133.1, 130.7, 130.6, 129.2, 126.8, 121.6, 118.8, 64.9, 59.6, 55.5, 52.9, 52.1, 46.1, 41.1, 35.5, 34.3, 26.0, 24.7. HRMS (ESI) $[M+Na]^+$ m/z calcd for $C_{33}H_{40}N_4O_9SNa$ 691.2414, found 691.2410

Compound 8f

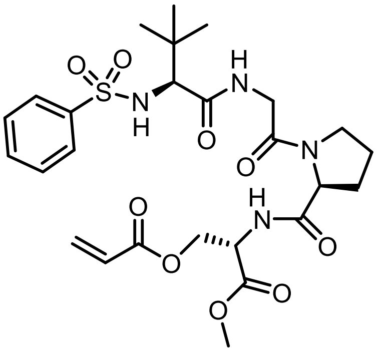

According to the general procedure, The crude residue was purified by flash column chromatography on silica gel (ethyl acetate : methol =10:1; R_f = 0.41) to produce compound **8f** (500 mg, 89% yield). ^{1}H NMR (400 MHz, $CDCl_3$) δ 7.89 (d, J = 7.5 Hz, 2H), 7.64 (t, J = 7.5 Hz, 1H), 7.52 – 7.38 (m, 3H), 7.12 (s, 1H), 6.35 (d, J = 17.3 Hz, 1H), 6.03 (dd, J = 17.3, 10.5 Hz, 1H), 5.84 (d, J = 10.5 Hz, 1H), 5.62 (d, J = 10.3 Hz, 1H), 4.97 – 4.77 (m, 2H), 4.58 (dd, J = 11.4, 3.8 Hz, 1H), 4.30 (dd, J = 11.4, 3.5 Hz, 1H), 3.86 – 3.74 (s, 3H), 3.69 (t, J = 11.9 Hz, 1H), 3.51 (dd, J = 11.7, 6.0 Hz, 1H), 3.36 (dd, J = 17.0, 7.8 Hz, 1H), 3.27 (dd, J = 18.1, 2.2 Hz, 1H), 2.26 (ddd, J = 27.4, 13.9, 6.0 Hz, 2H), 2.18 – 1.97 (m, 2H), 1.85 (s, 1H), 0.88 (s, 9H). ^{13}C NMR (100 MHz, $CDCl_3$) δ 171.5, 169.7, 166.9, 165.4, 139.3, 133.1, 131.9, 129.1, 127.5, 79.1 – 74.1, 64.0, 63.6, 59.8, 52.9, 51.7, 46.5, 42.5, 34.3, 28.8, 26.3, 24.5. HRMS (ESI) $[M+Na]^+$ m/z calcd for $C_{26}H_{36}N_4O_9SNa$ 603.2101, found 603.2112.

Compound 9f

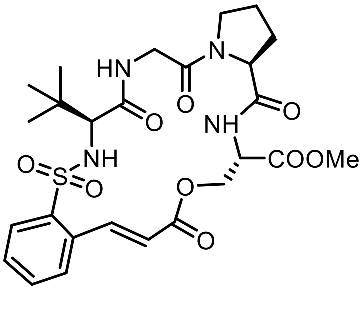

According to the general procedure, The crude residue was purified by flash column chromatography on silica gel (ethyl acetate: methol =8:1; R_f = 0.43) to produce compound **9f** (23 mg, 67% yield).^{1}H NMR (400 MHz, $CDCl_3$) δ 8.75 (d, 1H), 7.90 – 7.79 (m, 2H), 7.54 (dd, J = 10.5, 4.3 Hz, 1H), 7.41 (dd, J = 15.3, 7.3 Hz, 3H), 6.79 (s, 1H), 6.62 (s, 1H), 5.94 (d, J = 1.1 Hz, 1H), 5.76 (d, J = 10.3 Hz, 1H), 4.74 – 4.65 (m, 1H), 3.86 (dd, J = 18.0, 4.3 Hz, 1H), 3.82 (s, 3H), 3.80 (s, 1H), 3.57 (d, J = 10.3 Hz, 1H), 3.52 – 3.45 (m, 2H), 3.37 (dd, J = 11.4, 5.4 Hz, 1H), 2.31 – 2.15 (m, 2H), 2.04 (td, J = 10.7, 4.9 Hz, 2H), 0.92 (s,

9H). ^{13}C NMR (100 MHz, $CDCl_3$) δ 172.2, 170.0, 169.3, 167.1, 166.2, 164.1, 139.6, 132.7, 131.3, 129.0, 127.4, 109.6, 78.3-75.4, 64.9, 64.6, 60.9, 58.9, 53.0, 52.5, 46.4, 45.9, 42.2, 34.5, 28.4, 26.4, 24.8. HRMS (ESI) $[M+Na]^+$ m/z calcd for $C_{26}H_{34}N_4O_9SNa$ 601.1944, found 601.1951.

Compound8g

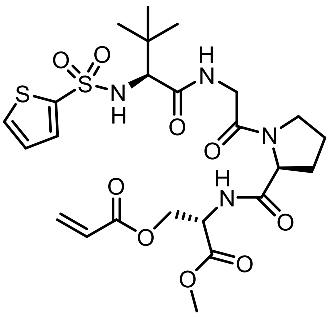

According to the general procedure, The crude residue was purified by flash column chromatography on silica gel (ethyl acetate : methol =10:1; R_f = 0.52) to produce compound **8g** (560 mg, 83% yield).^{1}H NMR (400 MHz, $CDCl_3$) δ 7.60 (d, *J* = 1.1 Hz, 2H), 7.60 (s, 1H), 7.25 (d, *J* = 7.9 Hz, 1H), 7.05 (m, 1H), 6.63 (s, 1H), 6.42 (dd, *J* = 17.3, 1.2 Hz, 1H), 6.12 (dd, *J* = 17.3, 10.5 Hz, 1H), 5.90 (dd, *J* = 10.5, 1.2 Hz, 1H), 5.61 (d, *J* = 9.9 Hz, 1H), 4.86 (dt, *J* = 7.8, 3.8 Hz, 1H), 4.63 (d, *J* = 5.5 Hz, 1H), 4.54 (dd, *J* = 11.4, 4.0 Hz, 1H), 4.48 (dd, *J* = 11.5, 3.6 Hz, 1H), 3.94 (dd, *J* = 17.8, 4.2 Hz, 1H), 3.57 (d, *J* = 9.8 Hz, 3H), 3.54 (d, *J* = 2.4 Hz, 2H), 3.50 (m, 1H), 3.37 (dd, *J* = 16.1, 8.9 Hz, 1H), 2.32 (m, 2H), 2.14 (dt, *J* = 16.0, 8.5 Hz, 1H), 2.01 (m, 2H), 0.96 (s, 9H). ^{13}C NMR (100 MHz, $CDCl_3$) δ 170.9, 169.7, 169.3, 167.1, 165.6, 140.4, 132.9, 132.5, 132.1, 127.6, 127.5, 65.2, 63.5, 60.1, 53.0, 52.0, 46.4, 42.3, 34.5, 28.2, 26.5, 24.7. HRMS (ESI) $[M+Na]^+$ m/z calcd for $C_{24}H_{34}N_4O_9S_2Na$ 609.1665, found 609.1671.

Compound 9g

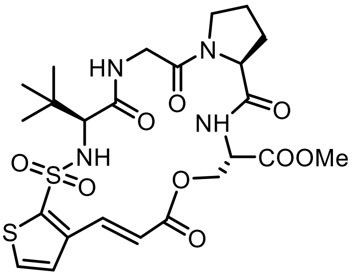

According to the general procedure, The crude residue was purified by flash column chromatography on silica gel (ethyl acetate: methol =10:1; R_f = 0.49) to produce compound **9g** (23 mg, 58% yield).^{1}H NMR (500 MHz, $CDCl_3$) δ 8.76 (s, 1H), 7.56 (ddd, *J* = 6.2, 4.4, 1.2 Hz, 2H), 7.01 (dd, *J* = 4.9, 3.8 Hz, 1H), 6.63 (s, 1H), 6.55 (s, 1H), 5.96 (d, *J* = 1.2 Hz, 1H), 5.67 (d, *J* = 10.0 Hz, 1H), 4.75 – 4.56 (m, 1H), 3.98 – 3.90 (m, 1H), 3.90 – 3.87 (s, 3H), 3.62 (dd, *J* = 17.9, 3.5 Hz, 1H), 3.56 (d, *J* = 10.0 Hz, 2H), 3.54 – 3.49 (m, 1H), 3.41 (dd, *J* = 16.2, 8.9 Hz, 1H), 2.35 (ddd, *J* = 15.2, 7.6, 4.6 Hz, 1H), 2.28 – 2.16 (m, 1H), 2.13 – 1.99 (m, 3H), 0.99 (d, *J* =

6.1 Hz, 9H). ^{13}C NMR (125 MHz, $CDCl_3$) δ 169.6, 169.3, 167.2, 164.3, 140.5, 132.7, 132.3, 131.1, 127.4, 109.5, 65.2, 61.0, 53.1, 46.4, 42.2, 34.6, 28.1, 26.5, 24.8. HRMS (ESI) $[M+Na]^+$ m/z calcd for $C_{24}H_{32}N_4O_9S_2Na$ 607.1508, found 607.1512.

Compound 8h

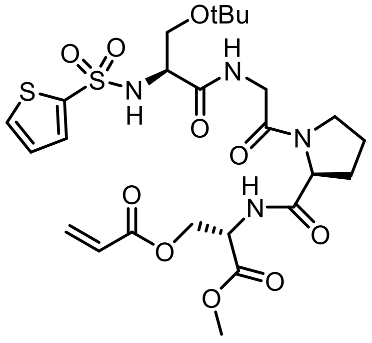

According to the general procedure, The crude residue was purified by flash column chromatography on silica gel (ethyl acetate: methol =20:1; R_f = 0.49) to produce compound **8h** (430 mg, 78% yield). ^1H NMR (500 MHz, $CDCl_3$) δ 8.80 (s, 1H), 7.98 – 7.87 (m, 2H), 7.77 (d, *J* = 3.8 Hz, 1H), 7.59 (t, *J* = 7.4 Hz, 1H), 6.63 – 6.45 (m, 1H), 5.96 – 5.83 (m, 2H), 4.64 (dd, *J* = 8.0, 2.1 Hz, 1H), 4.13 (d, *J* = 7.1 Hz, 1H), 4.07 – 4.03 (m, 1H), 4.03 – 3.95 (m, 2H), 3.88 – 3.81 (m, 4H), 3.81 – 3.74 (m, 3H), 3.62 – 3.50 (m, 1H), 3.43 (dd, *J* = 16.5, 9.2 Hz, 1H), 3.38 – 3.27 (m, 1H), 2.36 (ddd, *J* = 12.1, 6.2, 2.9 Hz, 1H), 2.22 – 2.08 (m, 2H), 2.08 – 2.01 (m, 2H), 2.01 – 1.92 (m, 2H), 1.15 (s, 9H). ^{13}C NMR (125 MHz, $CDCl_3$) δ 170.5, 169.6, 168.9, 167.5, 165.5, 139.8, 132.8, 132.5, 131.9, 127.6, 74.6, 63.4, 62.0, 59.9, 56.1, 52.9, 52.0, 46.3, 42.5, 27.3, 24.8. HRMS (ESI) $[M+Na]^+$ m/z calcd for $C_{25}H_{36}N_4O_{10}S_2Na$ 639.1771, found 639.6782.

Compound 9h

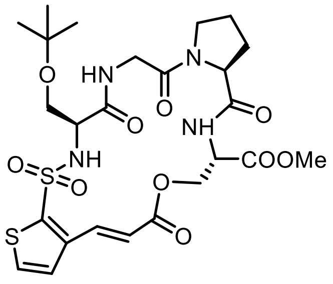

According to the general procedure, The crude residue was purified by flash column chromatography on silica gel (ethyl acetate: methol =10:1; R_f = 0.37) to produce compound **9h** (23 mg, 53% yield). ^1H NMR (500 MHz, $CDCl_3$) δ 8.22 (d, *J* = 15.9 Hz, 1H), 7.72 (d, *J* = 8.0 Hz, 1H), 7.57 (d, *J* = 5.0 Hz, 1H), 7.34 (d, *J* = 5.1 Hz, 1H), 7.05 (s, 1H), 6.37 (d, *J* = 16.0 Hz, 1H), 5.78 (d, *J* = 8.3 Hz, 1H), 4.96 (d, *J* = 4.1 Hz, 1H), 4.71 – 4.53 (m, 3H), 4.02 (s, 1H), 3.96 (d, *J* = 14.2 Hz, 1H), 3.87 (s, 3H), 3.79 (d, *J* = 15.0 Hz, 2H), 3.56 (dd, *J* = 21.0, 12.7 Hz, 2H), 3.35 (d, *J* = 8.0 Hz, 1H), 2.43 (s, 1H), 2.26 (d, *J* = 7.9 Hz, 1H), 2.03 (s, 1H),

1.89 (d, J = 19.0 Hz, 1H), 1.18 (s, 9H). ^{13}C NMR (125 MHz, $CDCl_3$) δ 170.6, 169.7, 168.4, 167.4, 165.7, 139.5, 138.7, 135.1, 131.2, 126.8, 121.8, 74.3, 63.5, 62.6, 59.9, 56.7, 53.1, 51.7, 46.4, 42.6, 27.4, 26.9, 25.1. HRMS (ESI) $[M+Na]^+$ m/z calcd for $C_{25}H_{34}N_4O_{10}S_2Na$ 637.1614, found 637.1620.

Compound 8i

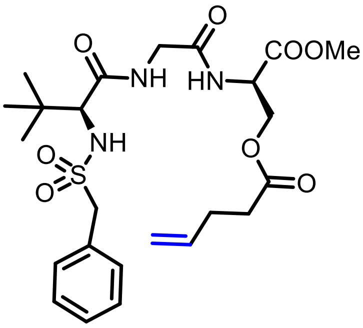

According to the general procedure, the crude residue was purified by flash column chromatography on silica gel (methanol: ethyl acetate= 1:30; R_f = 0.39) to produce compound **8i** (340 mg, 66% yield)^{1}H NMR (500 MHz, $CDCl_3$) δ 7.40 (m, 2H), 7.37 – 7.32 (m, 3H), 7.24 (d, J = 7.6 Hz, 1H), 6.61 (t, J = 4.7 Hz, 1H), 5.80 (dd, J = 17.0, 10.4 Hz, 1H), 5.53 (d, J = 9.3 Hz, 1H), 5.09 – 5.01 (m, 2H), 4.86 (dt, J = 7.6, 3.8 Hz, 1H), 4.43 (ddd, J = 27.1, 11.5, 3.9 Hz, 2H), 4.33 – 4.23 (m, 2H), 4.14 (dd, J = 17.1, 5.1 Hz, 1H), 4.05 (dd, J = 17.1, 5.0 Hz, 1H), 3.76 (s, 3H), 3.60 (d, J = 9.3 Hz, 1H), 2.46 – 2.28 (m, 4H), 1.01 (s, 9H).^{13}C NMR (125 MHz, $CDCl_3$) δ 172.6, 170.9, 169.6, 168.2, 136.4, 130.8, 128.6, 115.7, 65.3, 63.4, 59.5, 53.1, 52.0, 43.0, 34.7, 33.1, 28.6, 26.6. HRMS (ESI) $[M+Na]^+$ m/z calcd for $C_{24}H_{35}N_3O_8SNa$ 548.2043, found 548.2042

Compound 9i

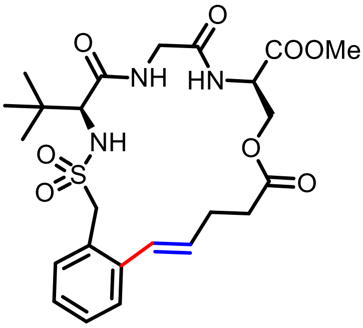

According to the general procedure, the crude residue was purified by flash column chromatography on silica gel (methanol: ethyl acetate= 1:30; R_f = 0.30) to produce compound **9i** (11 mg, 52% yield)^{1}H NMR (500 MHz, $CDCl_3$) δ 7.42 (t, J = 6.4 Hz, 2H), 7.35 (t, J = 6.6 Hz, 2H), 7.26 (t, J = 7.2 Hz, 1H), 6.78 (d, J = 15.9 Hz, 1H), 6.66 (m, 1H), 6.15 (dt, J = 10.9, 5.2 Hz, 1H), 5.51 (d, J = 8.7 Hz, 1H), 4.58 – 4.53 (m, 1H), 4.52 – 4.38 (m, 4H), 4.12 (dd, J = 16.5, 6.3 Hz, 1H), 3.85 (dd, J = 16.8, 5.7 Hz, 1H), 3.68 (s, 3H), 3.31 (d, J = 8.7 Hz, 1H), 2.70 – 2.55 (m, 3H), 2.49 (m, 1H), 0.99 (s, 9H).^{13}C NMR (125 MHz, $CDCl_3$) δ 172.7, 171.7, 169.3, 169.1, 139.4, 132.5, 132.4, 129.3, 128.5, 127.4, 127.3, 125.4, 66.3, 63.3, 57.6, 52.5, 52.3, 43.3, 34.2, 32.7, 26.9, 26.5. HRMS (ESI) $[M+Na]^+$ m/z calcd for $C_{24}H_{33}N_3O_8SNa$ 546.1886, found 546.1882

Compound 8j

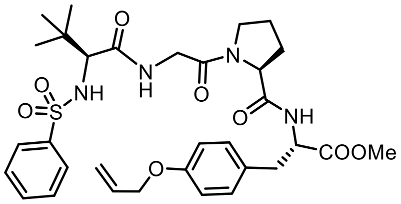

According to the general procedure, The crude residue was purified by flash column chromatography on silica gel (ethyl acetate: methol =20:1; R_f = 0.39) to produce compound **9h** (23 mg, 53% yield).^{1}H NMR (500 MHz, DMSO) δ 8.26 (d, *J* = 7.5 Hz, 1H), 8.07 – 7.86 (m, 1H), 7.82 – 7.72 (m, 2H), 7.66 – 7.40 (m, 4H), 7.12 (dd, *J* = 13.6, 8.6 Hz, 2H), 6.84 (dd, *J* = 8.7, 2.9 Hz, 2H), 6.09 – 5.84 (m, 1H), 5.44 – 5.28 (m, 1H), 5.24 (dd, *J* = 10.5, 1.6 Hz, 1H), 4.51 (d, *J* = 5.2 Hz, 2H), 4.46 – 4.27 (m, 1H), 3.80 – 3.59 (m, 2H), 3.54 (dd, *J* = 38.8, 7.0 Hz, 3H), 3.46 – 3.30 (m, 4H), 3.16 – 2.96 (m, 1H), 2.91 (ddd, *J* = 22.2, 10.9, 5.5 Hz, 2H), 2.51 (dt, *J* = 3.5, 1.7 Hz, 3H), 2.14 – 1.90 (m, 1H), 1.89 – 1.66 (m, 3H), 0.89 (s, 9H). ^{13}C NMR (125 MHz, DMSO) δ 172.3, 172.2, 171.9, 169.5, 167.3, 166.9, 157.4, 141.5, 141.4, 134.3, 132.5, 130.6, 130.5 – 130.3, 129.7, 129.1, 127.1, 127.1 – 126.9, 125.9, 117.7, 114.8, 68.5, 64.48 (s), 59.6, 54.3, 53.9, 52.3, 47.1, 46.2, 36.2, 34.4, 32.2, 29.5, 27.0, 24.5, 22.2, 9.1. HRMS (ESI) $[M+Na]^+$ m/z calcd for $C_{32}H_{42}N_4O_8SNa$ 665.2621, found 665.2628.

Compound 9j

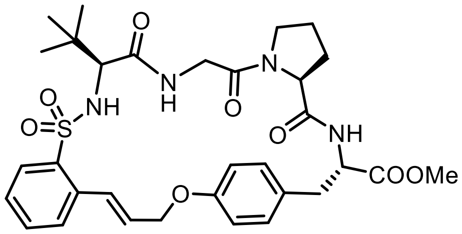

According to the general procedure, The crude residue was purified by flash column chromatography on silica gel (ethyl acetate: methol =10:1; R_f = 0.35) to produce compound **9j** (23 mg, 60% yield). ^{1}H NMR (500 MHz, $CDCl_3$) δ 8.08 (d, *J* = 7.8 Hz, 1H), 8.04 – 7.98 (m, 1H), 7.86 (d, *J* = 15.8 Hz, 1H), 7.54 (dd, *J* = 4.0, 1.2 Hz, 2H), 7.44 (ddd, *J* = 8.5, 5.3, 3.3 Hz, 2H), 7.08 (d, *J* = 8.6 Hz, 2H), 6.94 (d, *J* = 8.6 Hz, 2H), 6.29 (dt, *J* = 15.7, 5.5 Hz, 1H), 6.10 (d, *J* = 5.7 Hz, 1H), 5.30 (d, *J* = 9.5 Hz, 1H), 4.80 (tt, *J* = 12.0, 6.0 Hz, 2H), 4.78 – 4.71 (m, 1H), 4.50 (d, *J* = 7.4 Hz, 1H), 4.24 (dd, *J* = 17.8, 6.8 Hz, 1H), 3.88 (s, 1H), 3.83 (s, 3H), 3.30 (dddd, *J* = 29.1, 24.6, 16.4, 5.7 Hz, 5H), 2.92 (dd, *J* = 14.3, 8.8 Hz, 1H), 2.40 (dd, *J* = 12.5, 6.6 Hz, 1H), 2.20 – 2.07 (m, 1H), 1.84 – 1.68 (m, 2H), 1.01 (d, *J* = 24.9 Hz, 9H). ^{13}C NMR (125 MHz, $CDCl_3$) δ 172.1, 170.1, 169.7, 167.2 157.2, 137.8, 136.2, 133.1, 131.0, 130.5, 129.7, 129.1, 128.5, 128.4, 128.2, 115.8, 114.7, 68.2, 64.4, 59.4, 53.2, 52.6, 46.1, 41.7, 36.4, 34.7, 27.1 – 25.9, 24.9. HRMS (ESI) $[M+Na]^+$ m/z calcd for $C_{32}H_{40}N_4O_8SNa$ 663.2465, found 663.2451.

Compound 9k'

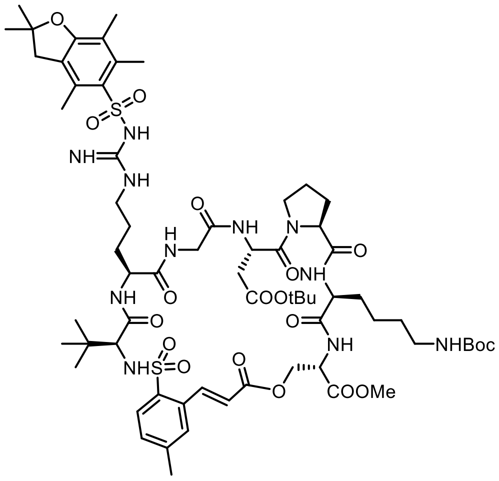

According to the general procedure, The crude residue was purified by flash column chromatography on silica gel (ethyl acetate: methol =6:1; R_f = 0.45) to produce compound **9k'** (23 mg, 60% yield).^{1}H NMR (500 MHz, $CDCl_3$) δ 7.83 (s, 1H), 7.77 (d, *J* = 7.2 Hz, 2H), 7.50 (d, *J* = 13.9 Hz, 1H), 7.46 – 7.28 (m, 1H), 7.26 (dd, *J* = 12.6, 5.2 Hz, 2H), 6.47 (dd, *J* = 64.0, 26.9 Hz, 4H), 6.12 (dd, *J* = 16.8, 10.6 Hz, 1H), 5.86 (d, *J* = 10.3 Hz, 1H), 5.08 (s, 1H), 4.87 – 4.68 (m, 2H), 4.66 – 4.31 (m, 5H), 4.14 (dd, *J* = 14.3, 7.1 Hz, 1H), 3.98 (d, *J* = 6.1 Hz, 1H), 3.87 (s, 1H), 3.82 – 3.68 (m, 5H), 3.45 – 3.29 (m, 2H), 3.24 – 2.87 (m, 6H), 2.57 (d, *J* = 31.3 Hz, 7H), 2.40 (d, *J* = 6.1 Hz, 4H), 2.23 (s, 1H), 2.11 (s, 4H), 1.99 (s, 2H), 1.69 (s, 2H), 1.54 – 1.32 (m, 27H), 0.97 – 0.72 (m, 9H). ^{13}C NMR (125 MHz, $CDCl_3$) δ 172.1, 169.8, 165.9, 158.9, 156.5, 156.0, 143.8, 138.4, 135.9, 132.0, 129.5, 127.7, 124.8, 117.6, 86.5, 82.3, 79.1, 65.2, 63.6, 61.2, 53.5, 52.8, 51.7, 47.9, 47.2, 43.4, 40.3, 38.0, 34.2, 30.3, 29.7, 29.4, 28.6, 28.5, 28.0, 26.6, 25.3, 24.7, 23.4, 21.6, 19.4, 18.0, 12.5. HRMS (ESI) $[M+Na]^+$ m/z calcd for $C_{65}H_{97}N_{11}O_{19}S_2Na$ 1422.6301, found 1422.6293.

Compound 7ak

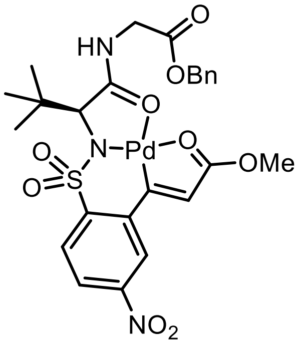

According to the general procedure, The crude residue was purified by flash column chromatography on silica gel (Petrol ether:ethyl acetate =4:1; R_f = 0.45) to produce compound **7ak** (400 mg, 80% yield).^{1}H NMR (400 MHz, $CDCl_3$) δ 8.36 (dd, J = 6.7, 4.7 Hz, 1H), 8.31 (d, J = 2.2 Hz, 1H), 8.23 (dd, J = 8.6, 2.2 Hz, 1H), 8.08 (d, J = 8.6 Hz, 1H), 7.32 (d, J = 3.3 Hz, 4H), 6.19 (s, 1H), 5.15 (s, 2H), 4.49 (dd, J = 17.9, 7.2 Hz, 1H), 4.42 (s, 1H), 4.10 (dd, J = 17.9, 4.5 Hz, 1H), 3.96 (s, 3H), 1.09 (s, 9H). ^{13}C NMR (100 MHz, $CDCl_3$) δ 183.4, 181.9, 171.9, 168.2, 148.5, 147.3, 138.7, 134.9, 128.6, 128.5, 128.4, 125.6, 124.4, 122.2, 120.4, 77.9 – 76.2, 70.7, 67.4, 54.7, 4.1, 36.6, 26.9. HRMS (ESI) [M+Na]+ m/z calcd for $C_{25}H_{27}N_3O_9PdSNa$ 670.0401, found 670.0402.

Compound 7jk

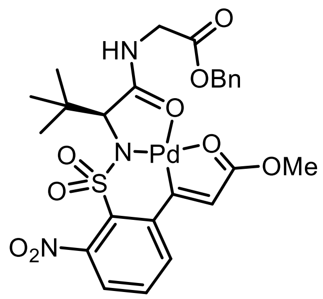

According to the general procedure, The crude residue was purified by flash column chromatography on silica gel (Petrol ether:ethyl acetate =3:1; R_f = 0.43) to produce compound **7jk** (380 mg, 78% yield).^{1}H NMR (400 MHz, $CDCl_3$) δ 8.28 – 8.22 (m, 1H), 7.59 (dd, J = 7.3, 1.8 Hz, 1H), 7.47 (d, J = 7.9 Hz, 1H), 7.45 – 7.41 (m, 1H), 7.40 – 7.28 (m, 5H), 6.08 (s, 1H), 5.17 (d, J = 3.6 Hz, 2H), 4.50 (dd, J = 17.5, 7.5 Hz, 1H), 4.36 (s, 1H), 4.01 (d, J = 5.1 Hz, 1H), 3.97 (s, 3H), 1.02 (s, 9H). ^{13}C NMR (100 MHz, $CDCl_3$) δ 183.12, 181.44, 173.24, 168.32, 147.24, 140.24, 135.28, 134.34, 130.90, 128.66, 128.44, 128.24, 128.07, 123.94, 122.54, 70.93, 67.17, 54.65, 41.90, 36.17, 26.81. HRMS (ESI) $[M+Na]^+$ m/z calcd for $C_{25}H_{27}N_3O_9PdSNa$ 670.0401, found 670.0398.

Compound 7ok

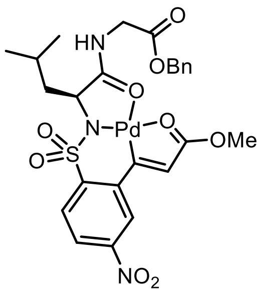

According to the general procedure, The crude residue was purified by flash column chromatography on silica gel (Petrol ether:ethyl acetate =4:1; R_f = 0.46) to produce compound **7ok** (420 mg, 83% yield).^{1}H NMR (400 MHz, $CDCl_3$) δ 8.30 (d, J = 2.2 Hz, 1H), 8.23 (dd, J = 8.6, 2.2 Hz, 1H), 8.10 (d, J = 8.6 Hz, 1H), 7.95 – 7.82 (m, 1H), 7.45 – 7.28 (m, 5H), 6.18 (s, 1H), 5.17 (s, 2H), 4.71 (dd, J = 9.9, 3.8 Hz, 1H), 4.36 (dd, J = 18.1, 6.7 Hz, 1H), 4.07 (dd, J = 18.1, 4.4 Hz, 1H), 3.97 (s, 3H), 1.85 – 1.74 (m, 1H), 1.55 (dddd, J = 17.0, 12.7, 8.6, 5.2 Hz, 2H), 0.94 (d, J = 6.4 Hz, 3H), 0.82 (d, J = 6.5 Hz, 3H). ^{13}C NMR (100 MHz, $CDCl_3$) δ 184.1, 183.3, 171.9, 168.2, 148.4, 147.6, 138.6, 134.9, 128.6, 128.4, 125.6, 124.5, 122.3, 120.3, 78.3 – 75.3, 67.5, 61.5, 54.7, 46.5, 41.9, 24.5, 23.4, 21.9. HRMS (ESI) $[M+Na]^+$ m/z calcd for $C_{25}H_{27}N_3O_9PdSNa$ 670.0401, found 670.0402.

Compound 7mk

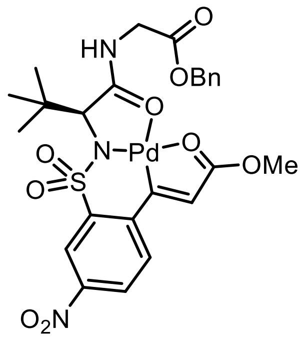

According to the general procedure, The crude residue was purified by flash column chromatography on silica gel (Petrol ether:ethyl acetate =4:1; R_f = 0.51) to produce compound **7mk** (460 mg, 87% yield).^{1}H NMR (400 MHz, $CDCl_3$) δ 8.74 (d, *J* = 2.4 Hz, 1H), 8.36 – 8.24 (m, 1H), 8.24 – 8.15 (m, 1H), 7.65 (dd, *J* = 17.7, 13.7 Hz, 1H), 7.33 (d, *J* = 2.5 Hz, 5H), 6.15 (s, 1H), 5.27 – 5.11 (m, 2H), 4.46 (dd, *J* = 17.9, 7.0 Hz, 1H), 4.42 (s, 1H), 4.22 – 4.04 (m, 1H), 3.96 (s, 3H), 1.09 (s, 9H). ^{13}C NMR (100 MHz, $CDCl_3$) δ 183.2, 182.1, 172.0, 168.1, 147.6, 144.0, 143.0, 134.9, 128.6, 126.6, 125.1, 123.2, 119.7, 78.3 – 75.9, 70.8, 67.5, 54.7, 42.2, 36.6, 26.9. HRMS (ESI) $[M+Na]^+$ m/z calcd for $C_{25}H_{27}N_3O_9PdSNa$ 670.0401, found 670.0400.

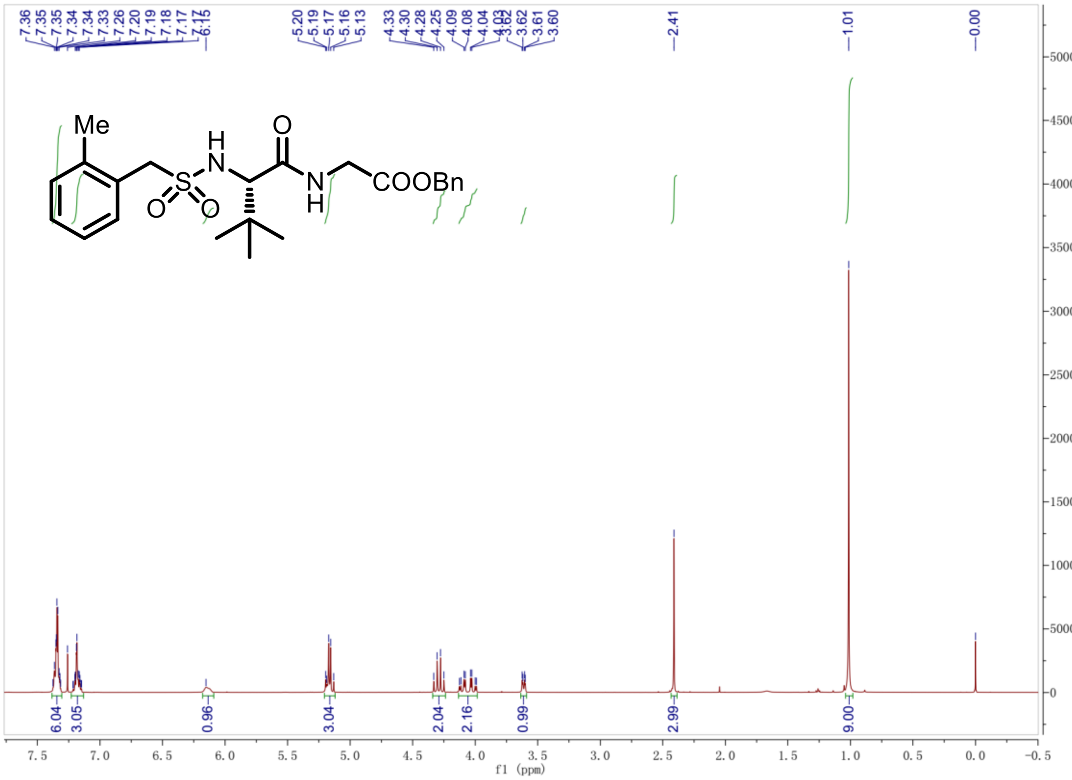

Supplementary Figure 7. ^{1}H NMR (500 MHz, $CDCl_3$) spectrum of compound **1a**

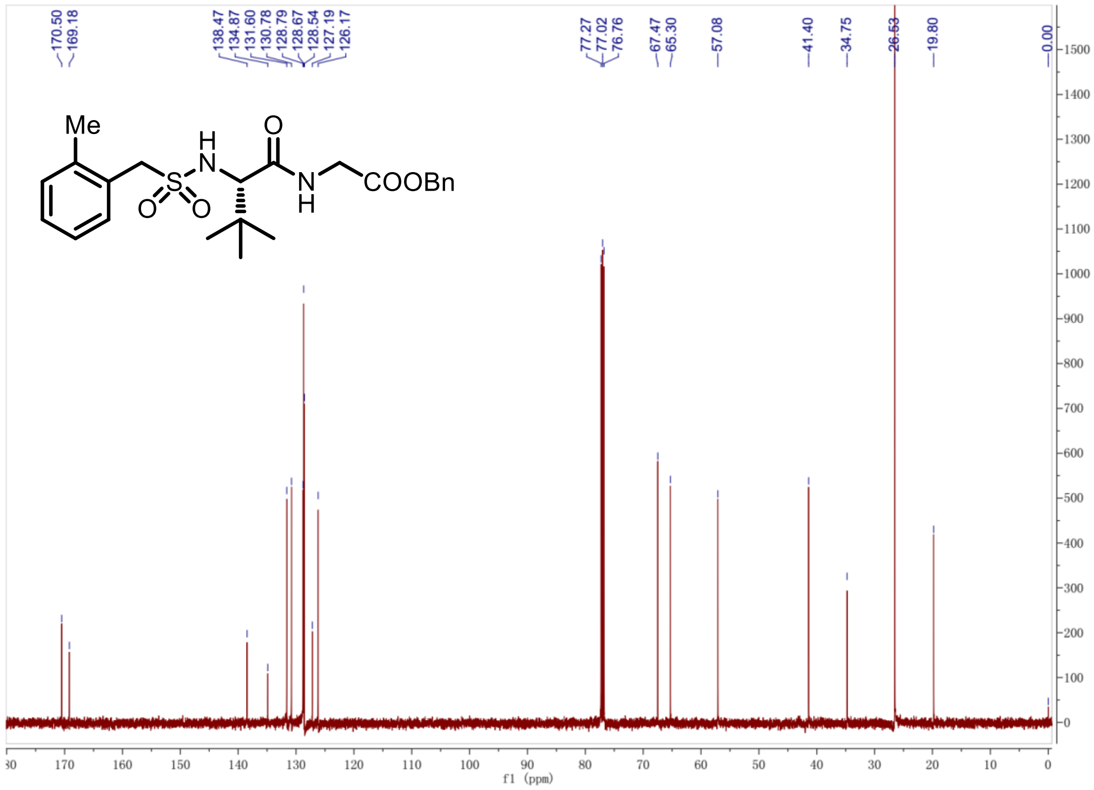

Supplementary Figure 8. ^{13}C NMR (125 MHz, $CDCl_3$) spectrum of compound **1a**

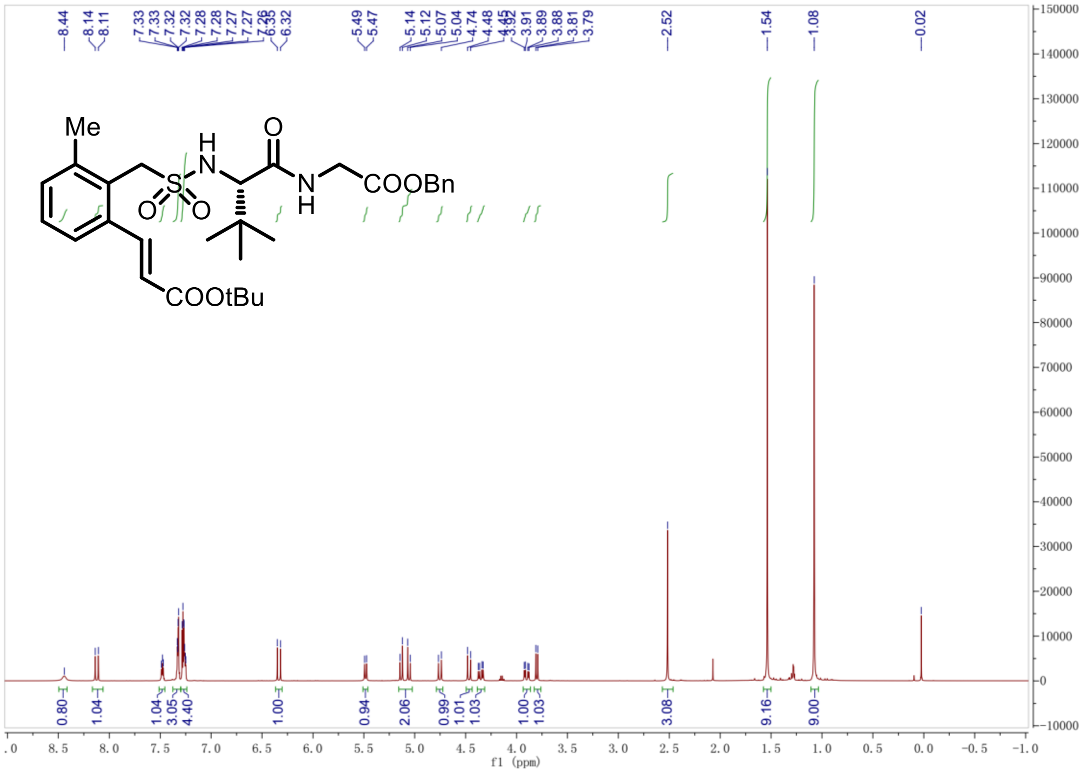

Supplementary Figure 9. ^{1}H NMR (500 MHz, $CDCl_3$) spectrum of compound **3aa**

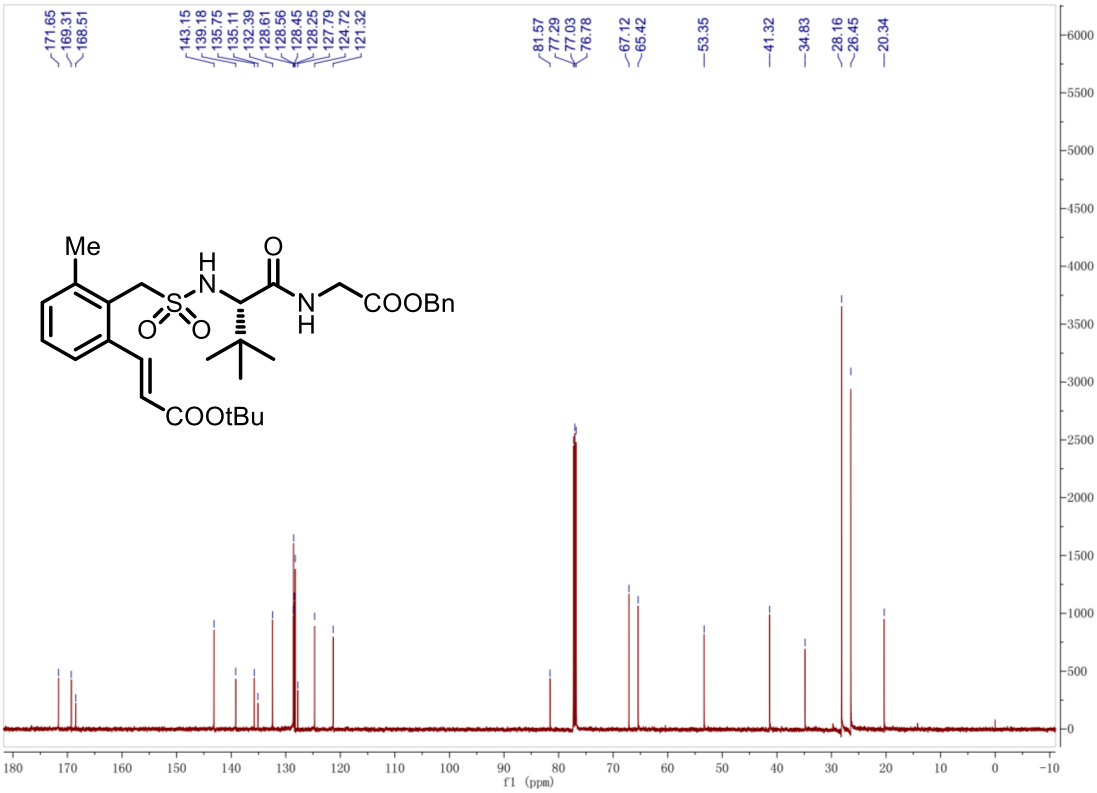

Supplementary Figure 10. ^{13}C NMR (125 MHz, $CDCl_3$) spectrum of compound **3aa**

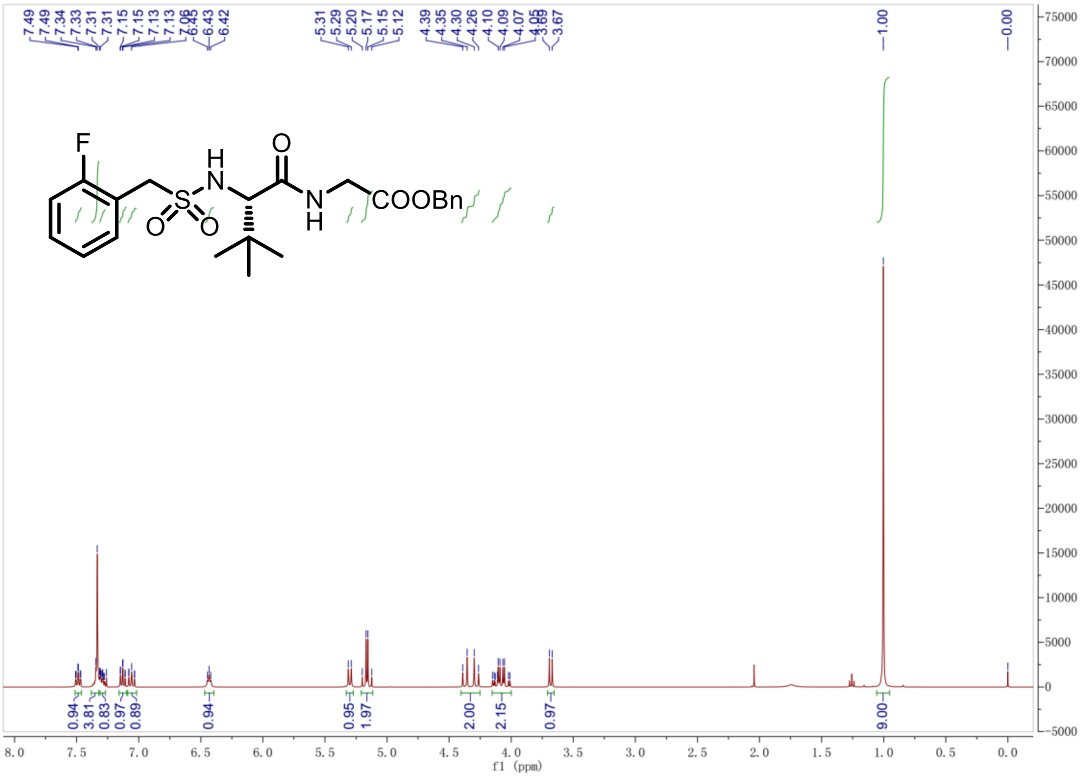

Supplementary Figure 11. ^{1}H NMR (400 MHz, $CDCl_3$) spectrum of compound **1b**

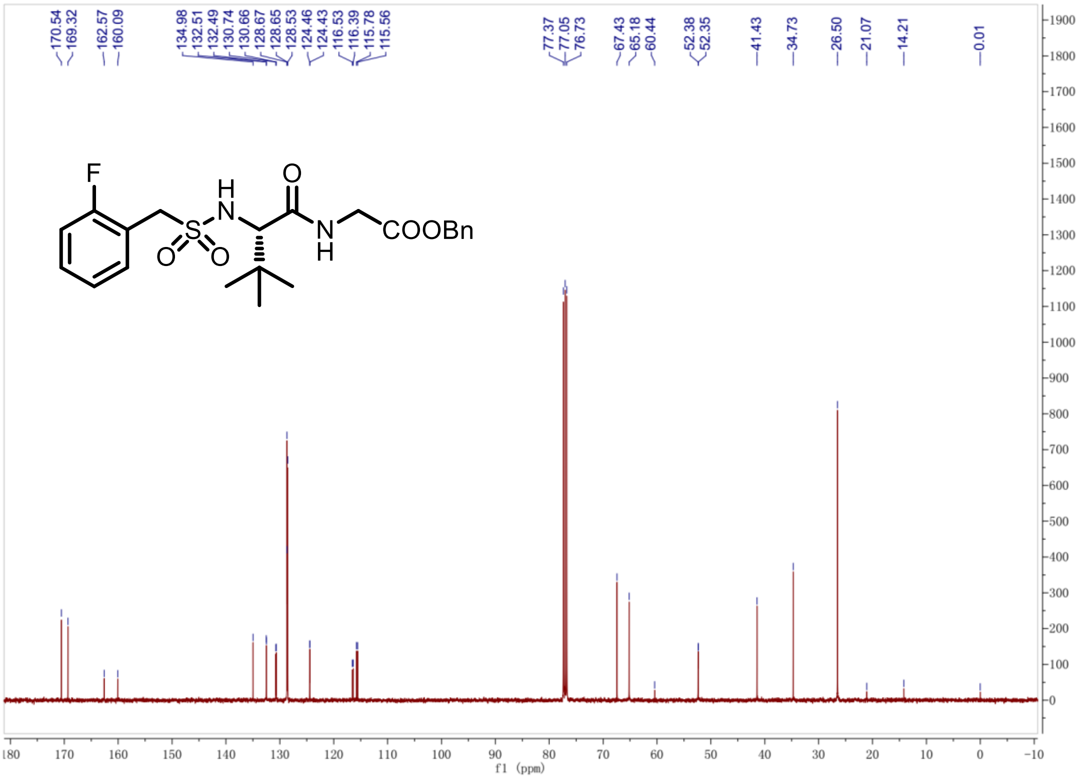

Supplementary Figure 12. ^{13}C NMR (100 MHz, $CDCl_3$) spectrum of compound **1b**

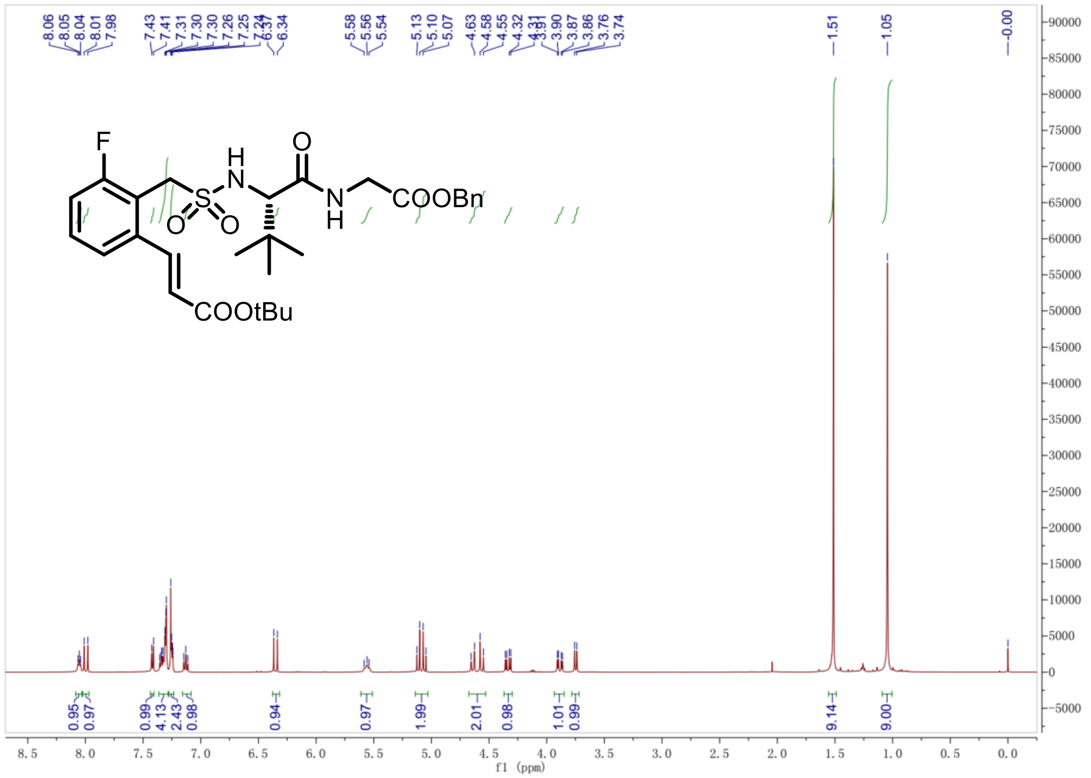

Supplementary Figure 13. ^{1}H NMR (500 MHz, $CDCl_3$) spectrum of compound **3ba**

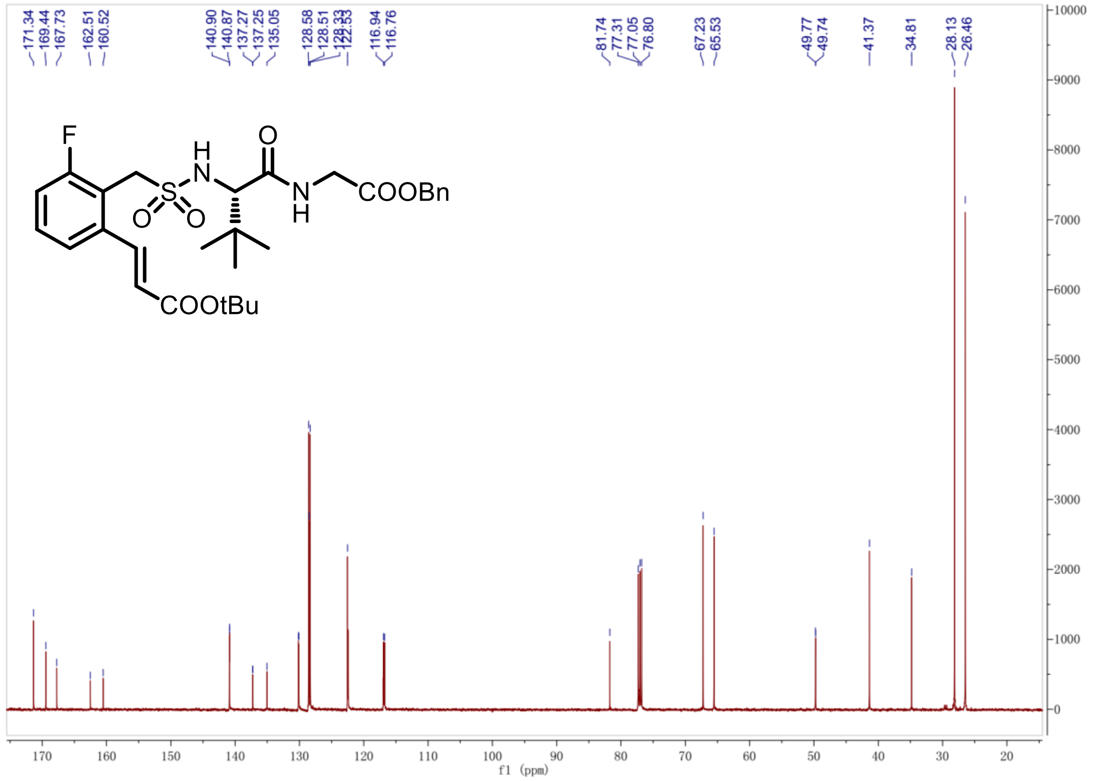

Supplementary Figure 14. ^{13}C NMR (125 MHz, $CDCl_3$) spectrum of compound **3ba**

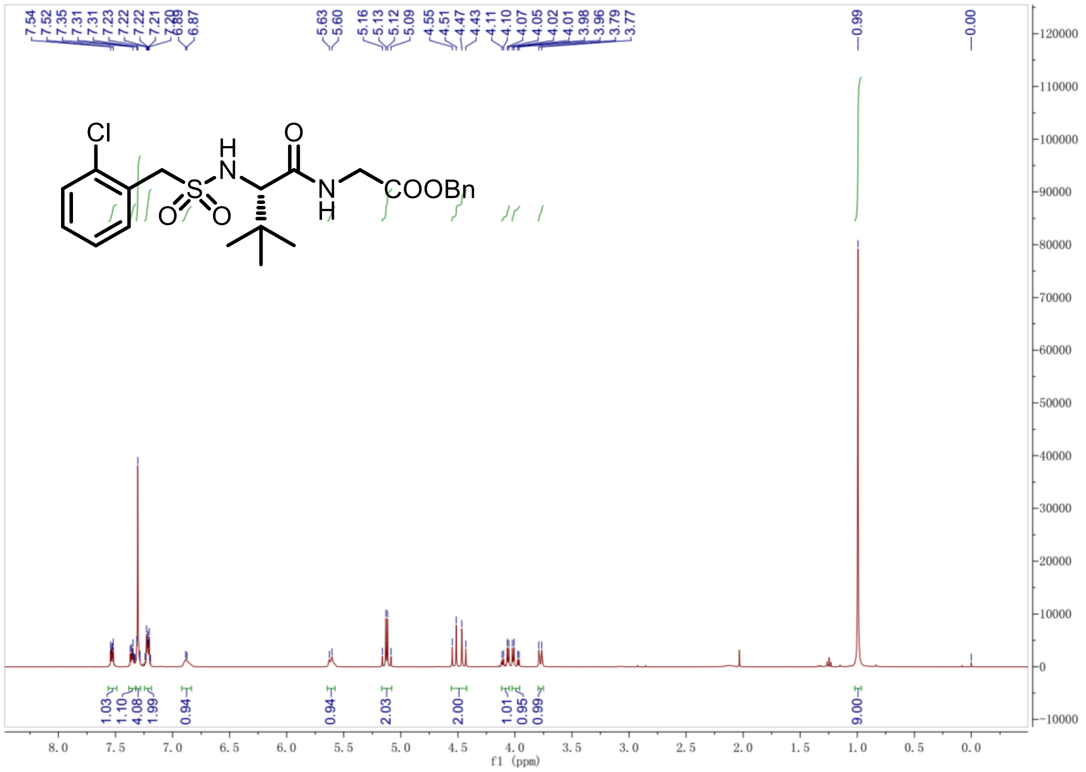

Supplementary Figure 15. ^{1}H NMR (400 MHz, $CDCl_3$) spectrum of compound **1c**

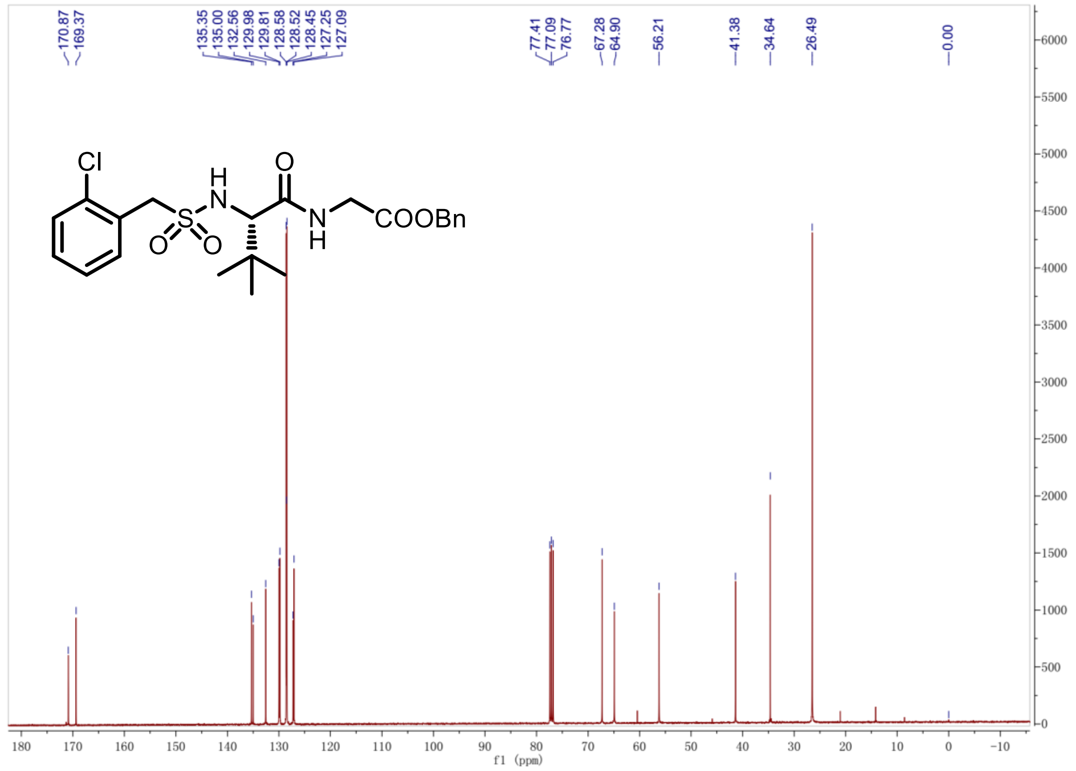

Supplementary Figure 16. ^{13}C NMR (100 MHz, $CDCl_3$) spectrum of compound **1c**

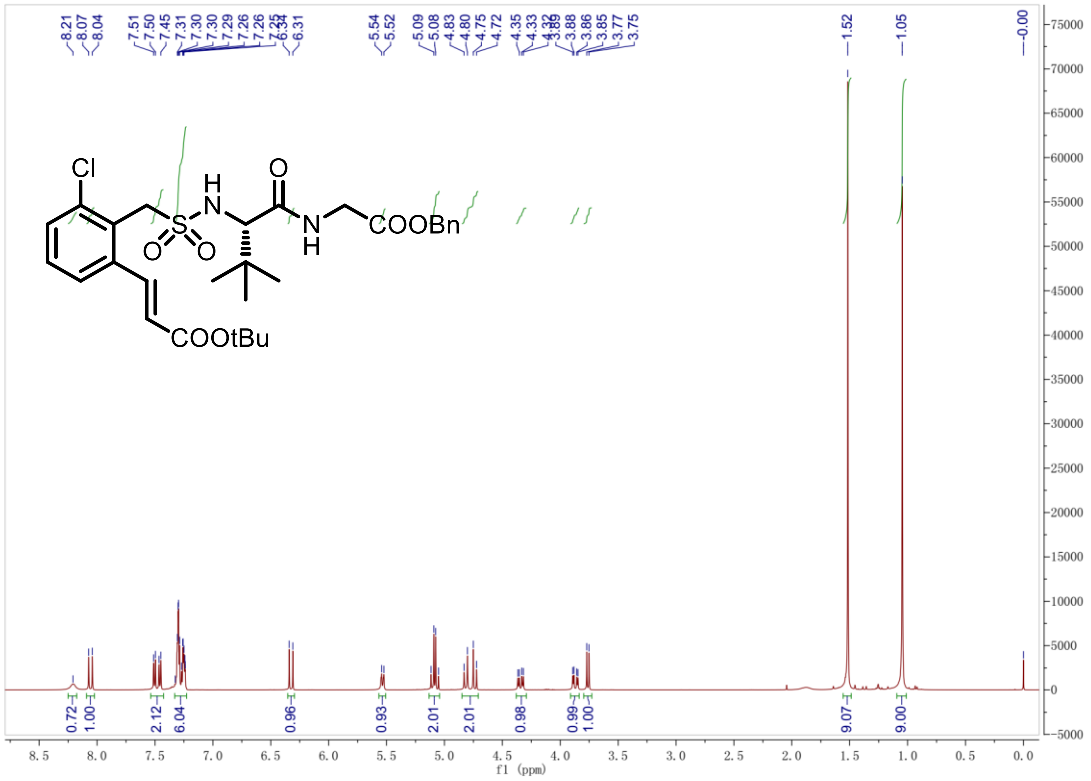

Supplementary Figure 17. ^{1}H NMR (500 MHz, $CDCl_3$) spectrum of compound **3ca**

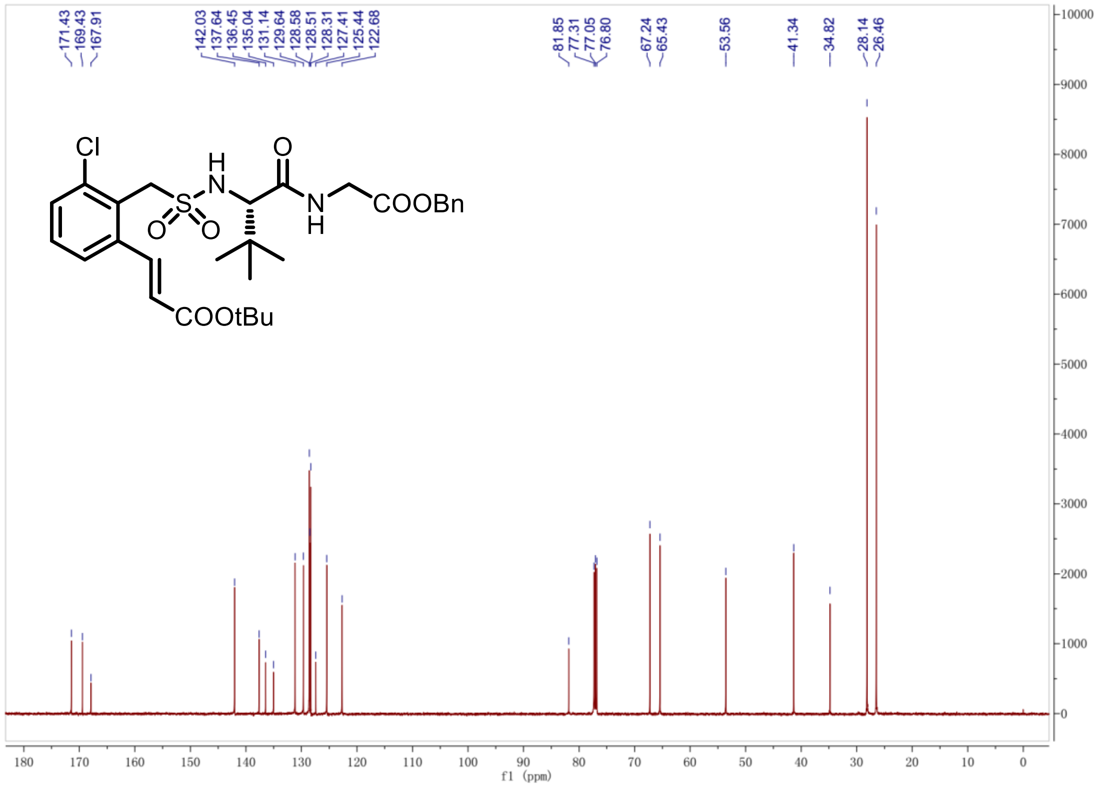

Supplementary Figure 18. ^{13}C NMR (125 MHz, $CDCl_3$) spectrum of compound **3ca**

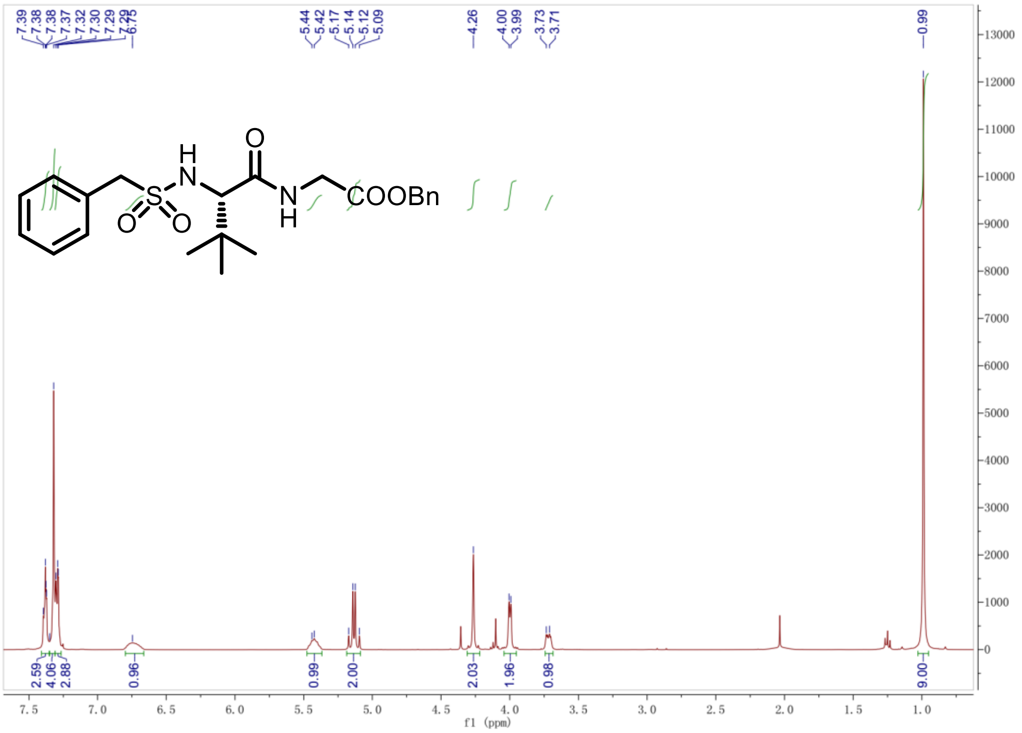

Supplementary Figure 19. ^{1}H NMR (400 MHz, $CDCl_3$) spectrum of compound **1d**

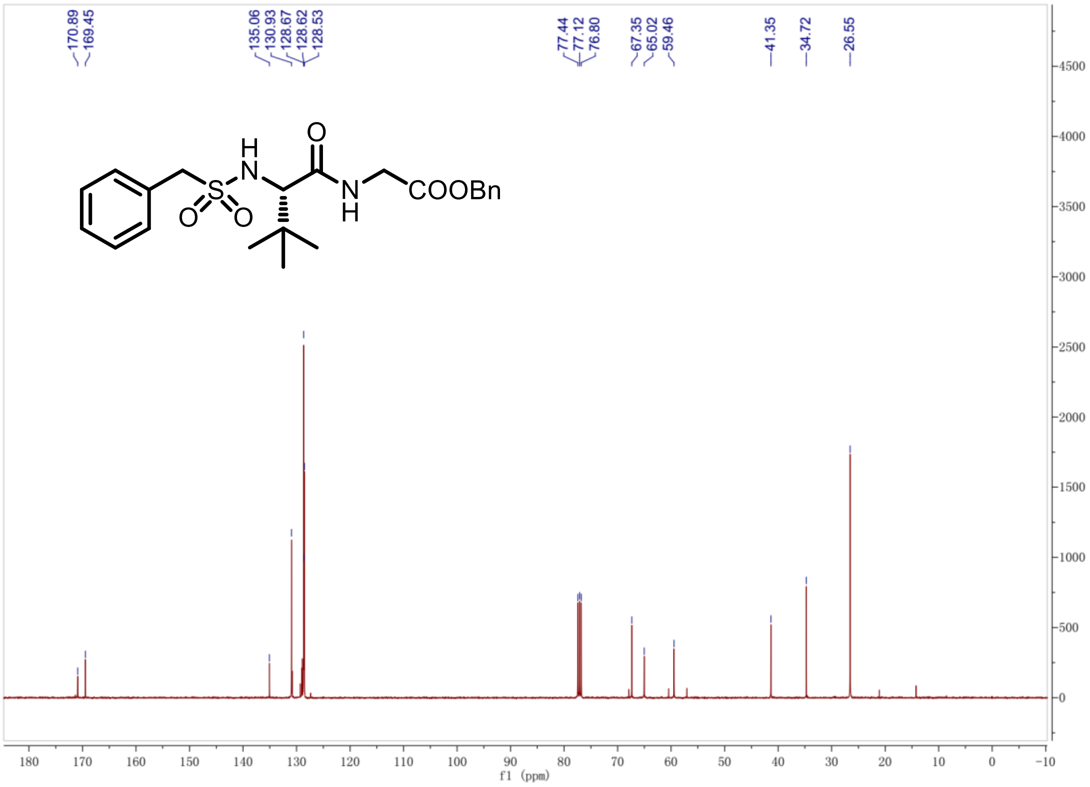

Supplementary Figure 20. ^{13}C NMR (100 MHz, $CDCl_3$) spectrum of compound **1d**

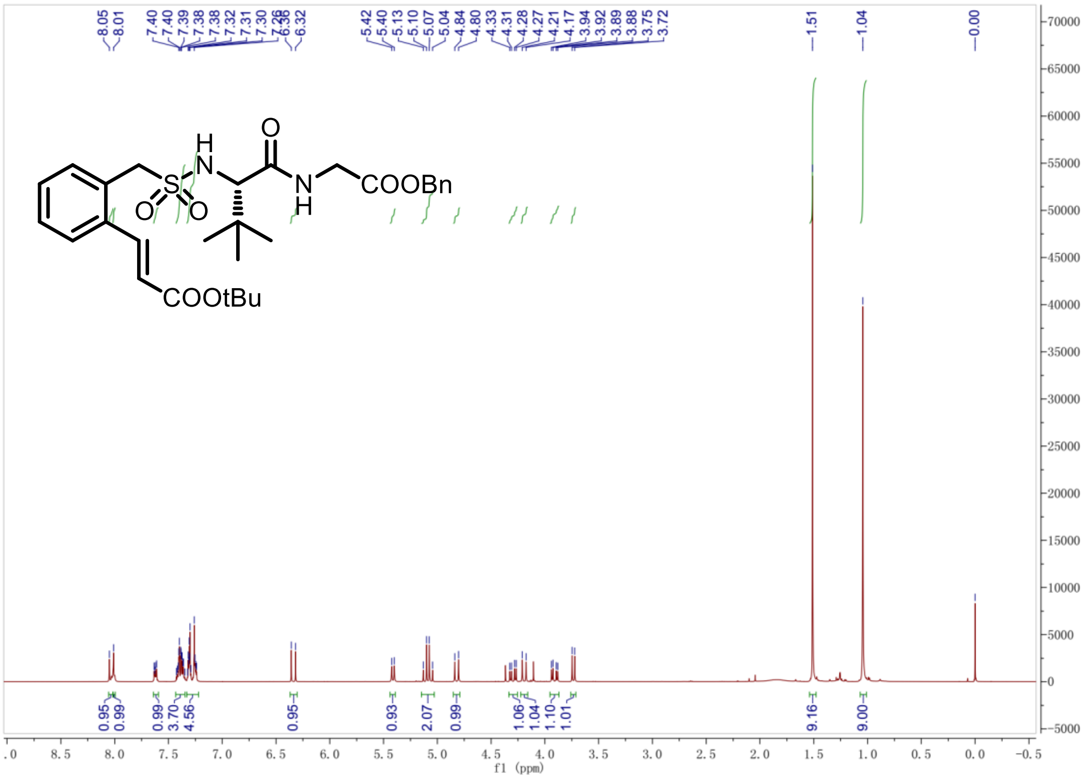

Supplementary Figure 21. ^{1}H NMR (400 MHz, $CDCl_3$) spectrum of **3da** (mono)

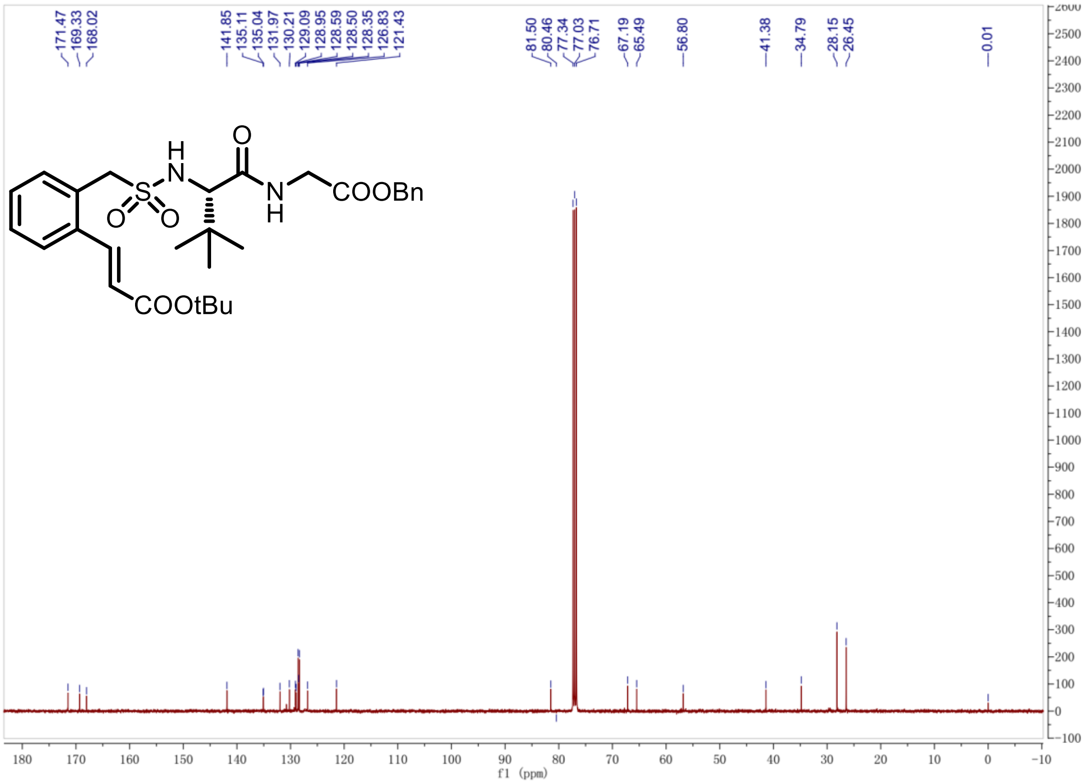

Supplementary Figure 22. ^{13}C NMR (100 MHz, $CDCl_3$) spectrum of **3da** (mono)

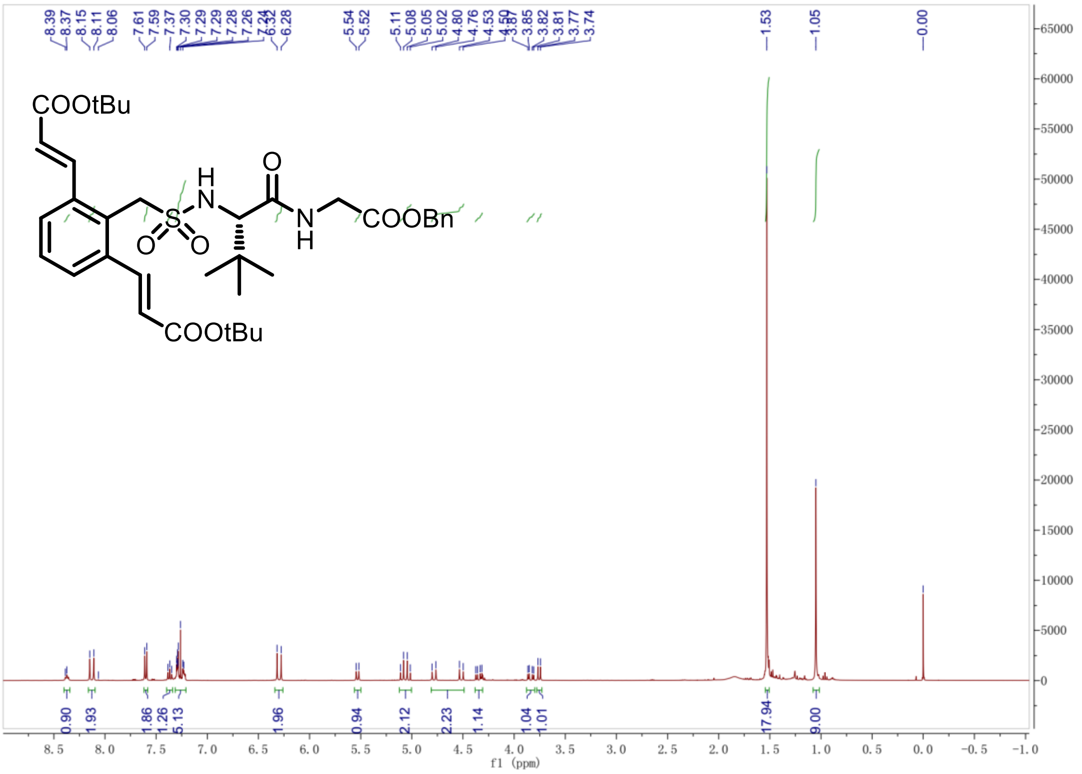

Supplementary Figure 23. ^{1}H NMR (400 MHz, $CDCl_3$) spectrum of **3da** (di)

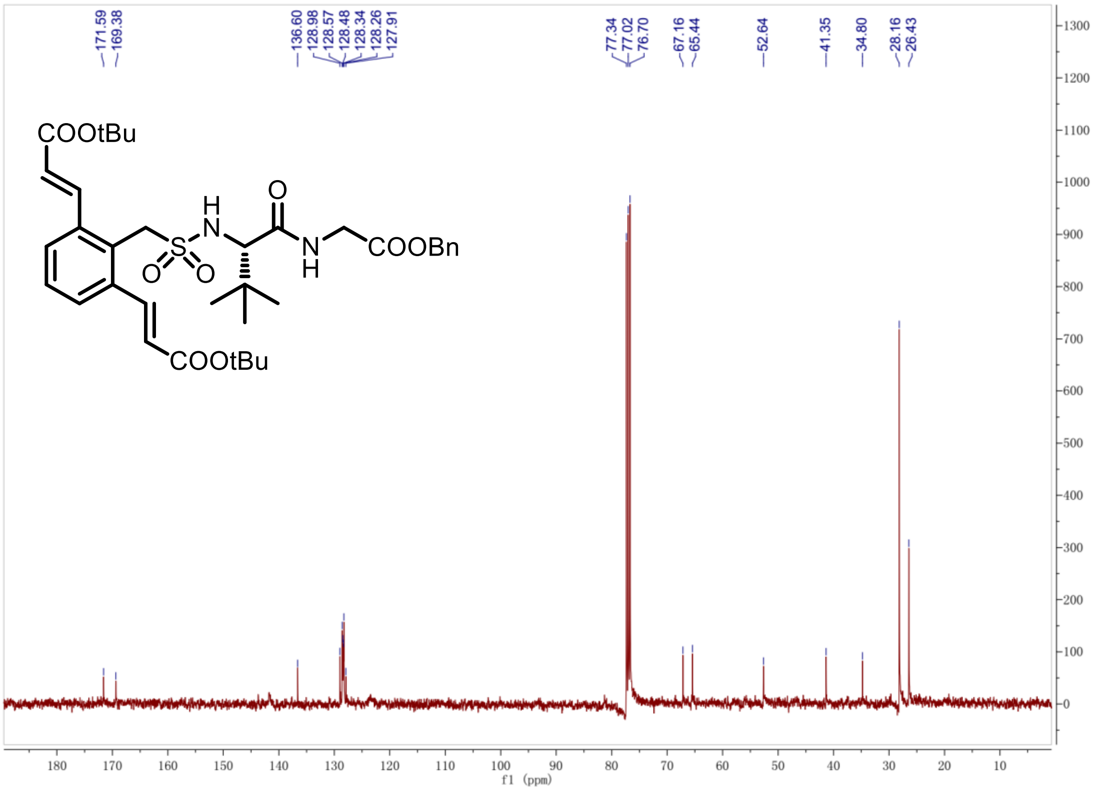

Supplementary Figure 24. ^{13}C NMR (100 MHz, $CDCl_3$) spectrum of **3da** (di)

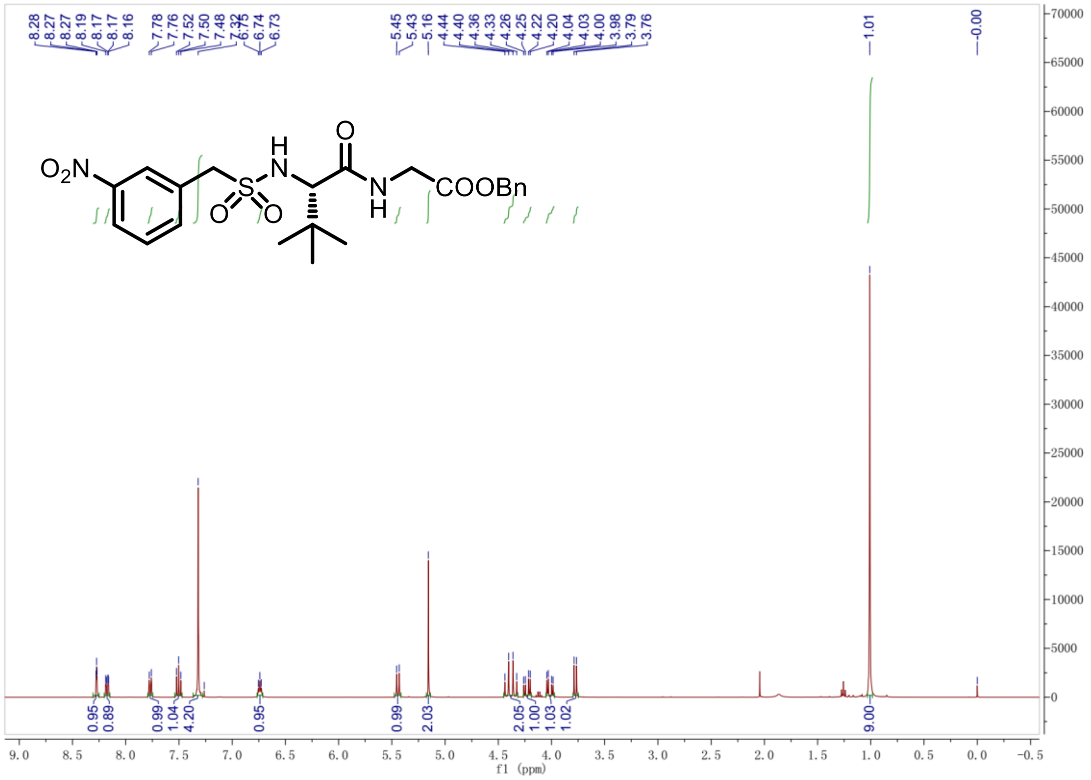

Supplementary Figure 25. ^{1}H NMR (400 MHz, $CDCl_3$) spectrum of compound **1e**

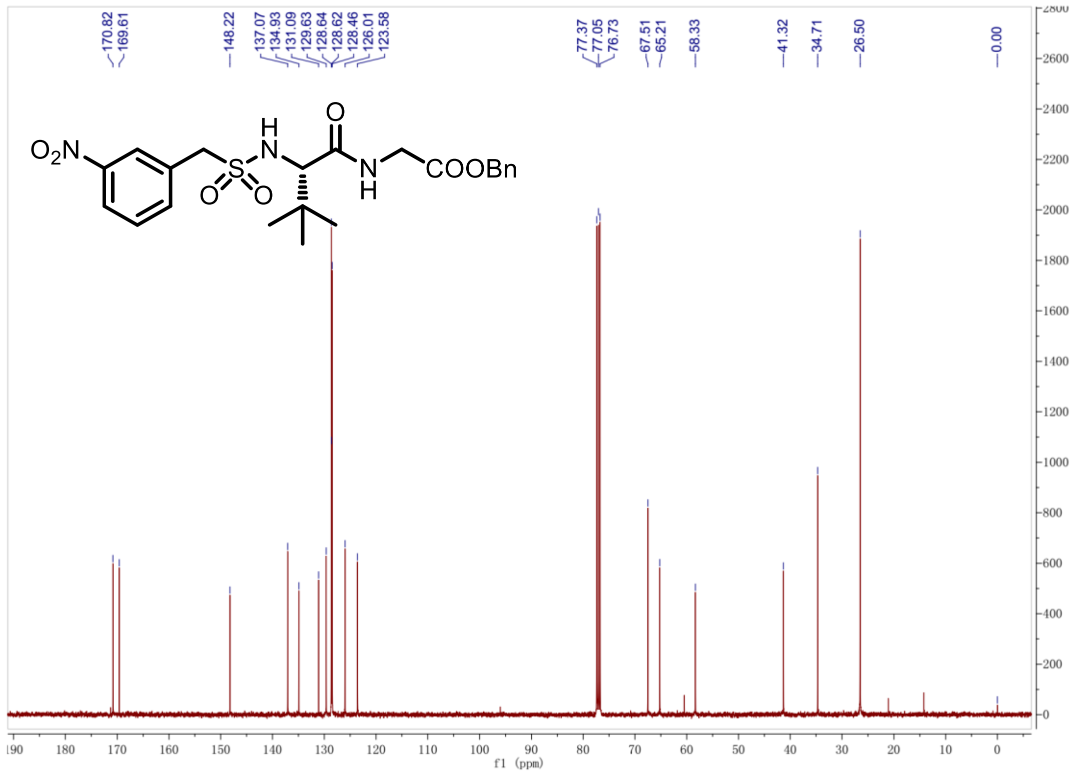

Supplementary Figure 26. ^{13}C NMR (100 MHz, $CDCl_3$) spectrum of compound **1e**

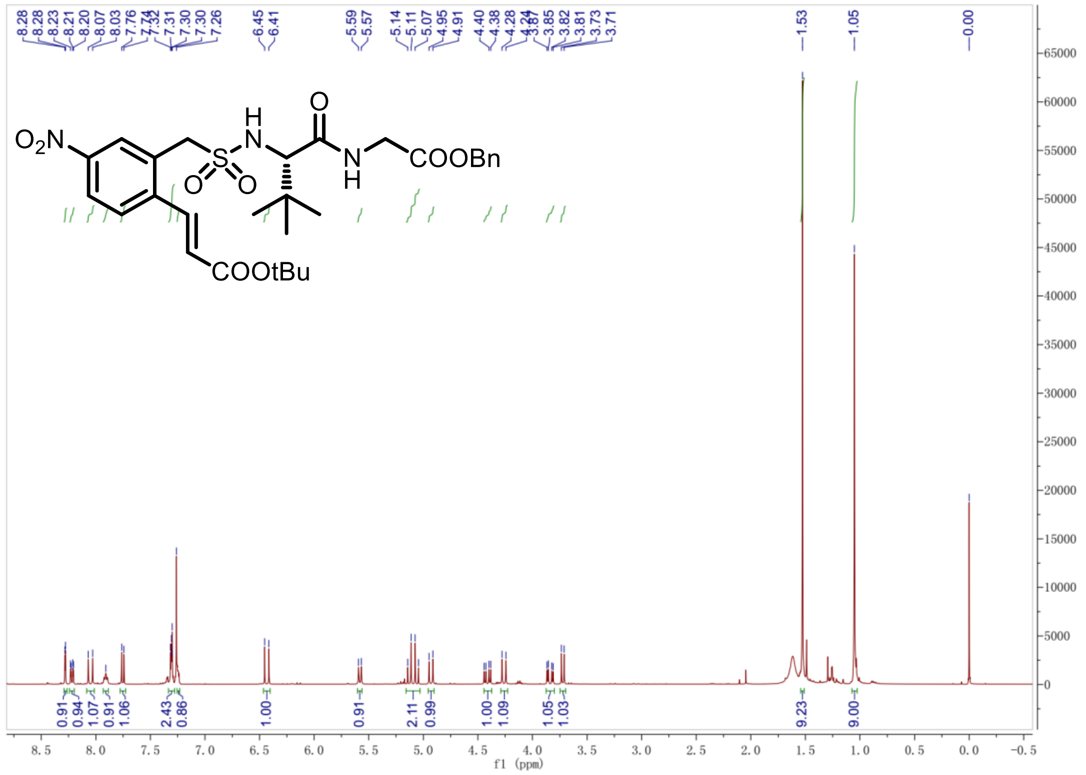

Supplementary Figure 27. ^{1}H NMR (400 MHz, $CDCl_3$) spectrum of **3ea** (mono)

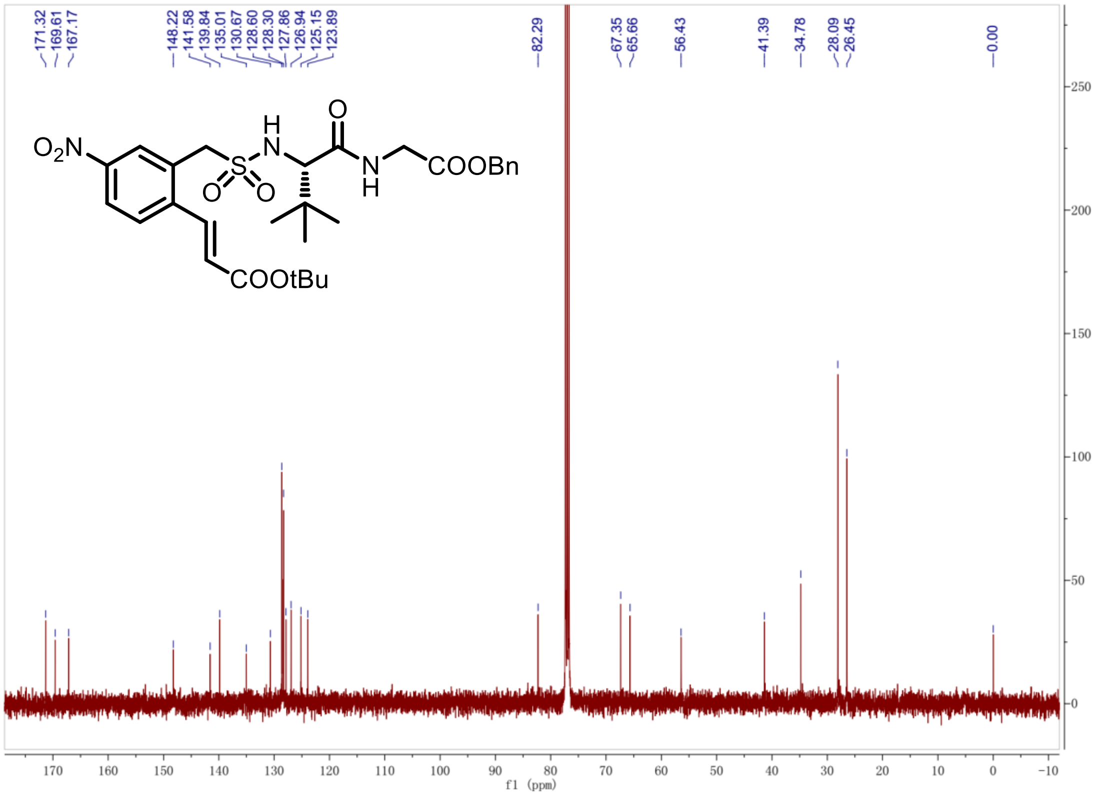

Supplementary Figure 28. ^{13}C NMR (100 MHz, $CDCl_3$) spectrum of **3ea** (mono)

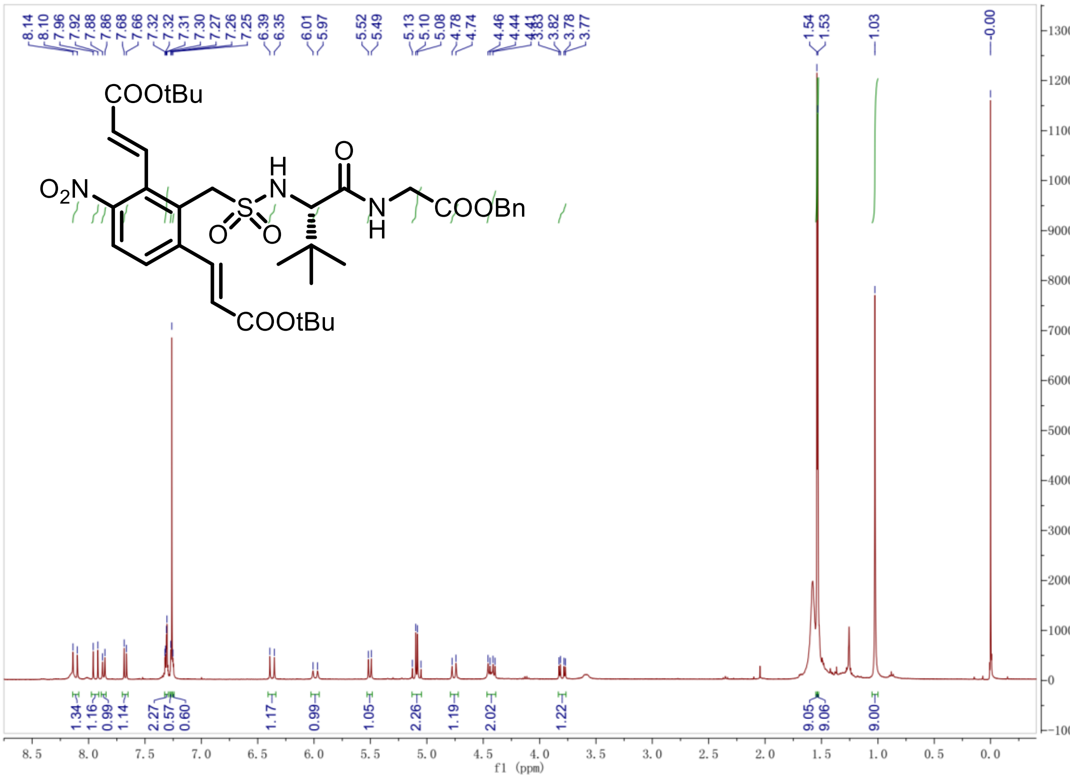

Supplementary Figure 29. ^{1}H NMR (400 MHz, $CDCl_3$) spectrum of **3ea** (di)

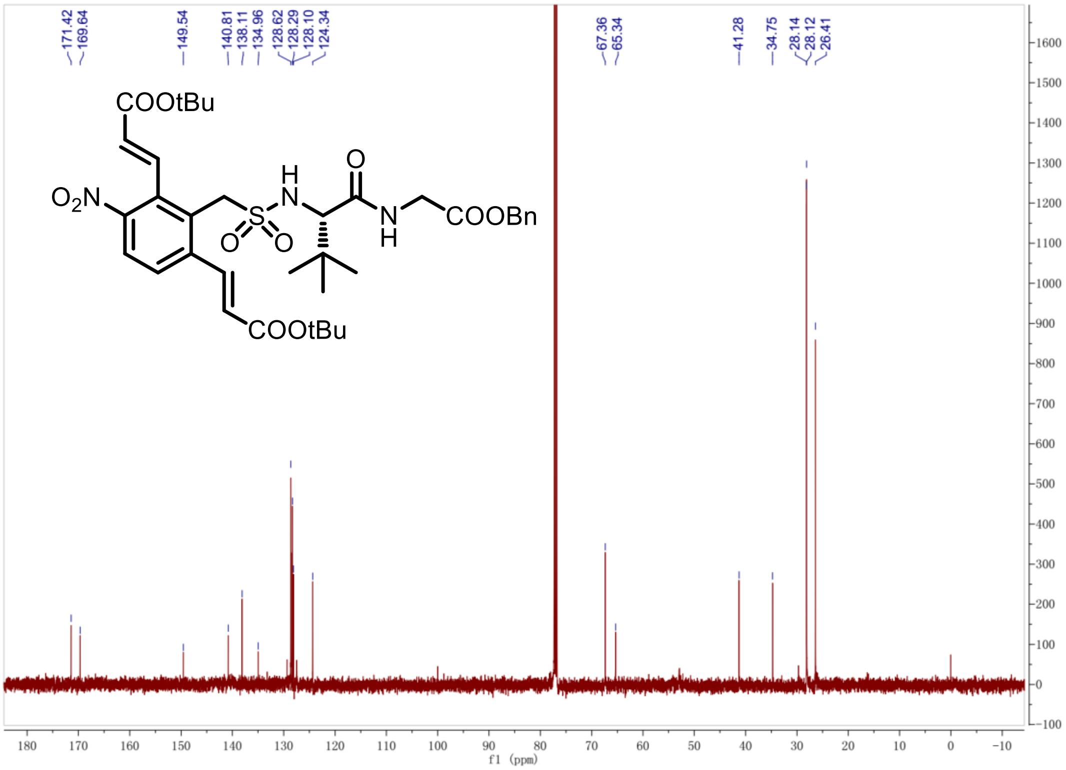

Supplementary Figure 30. ^{13}C NMR (100 MHz, $CDCl_3$) spectrum of **3ea** (di)

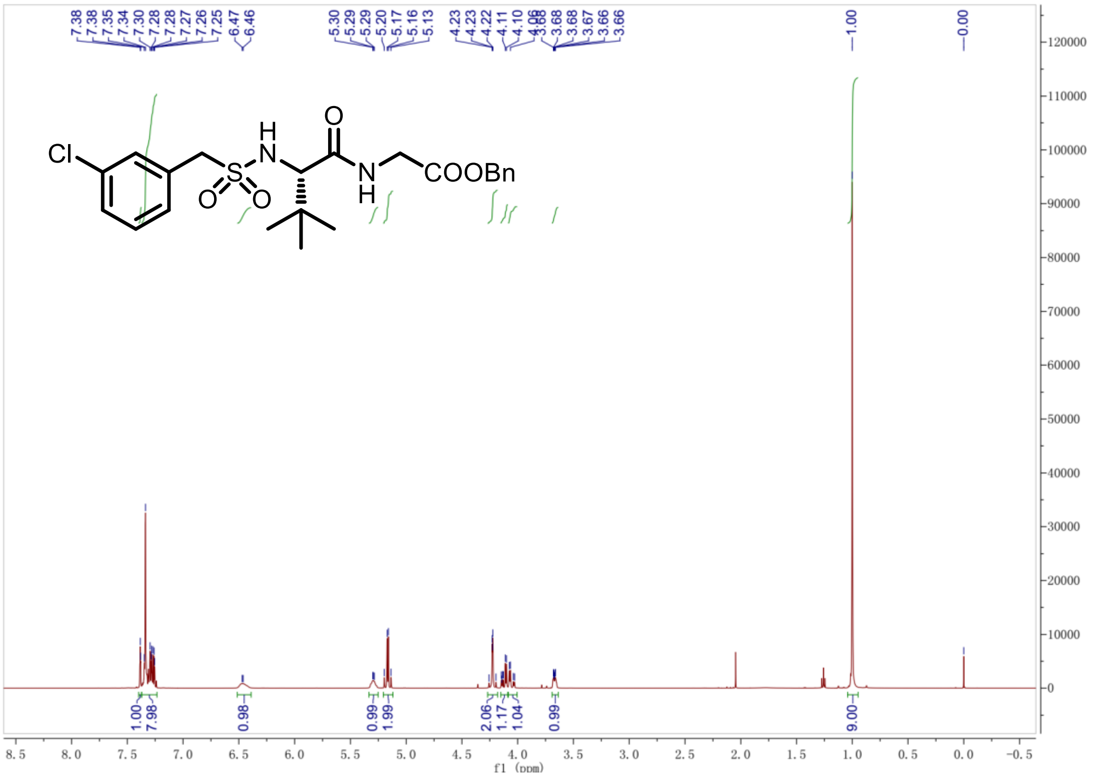

Supplementary Figure 31. ^{1}H NMR (500 MHz, $CDCl_3$) spectrum of compound **1f**

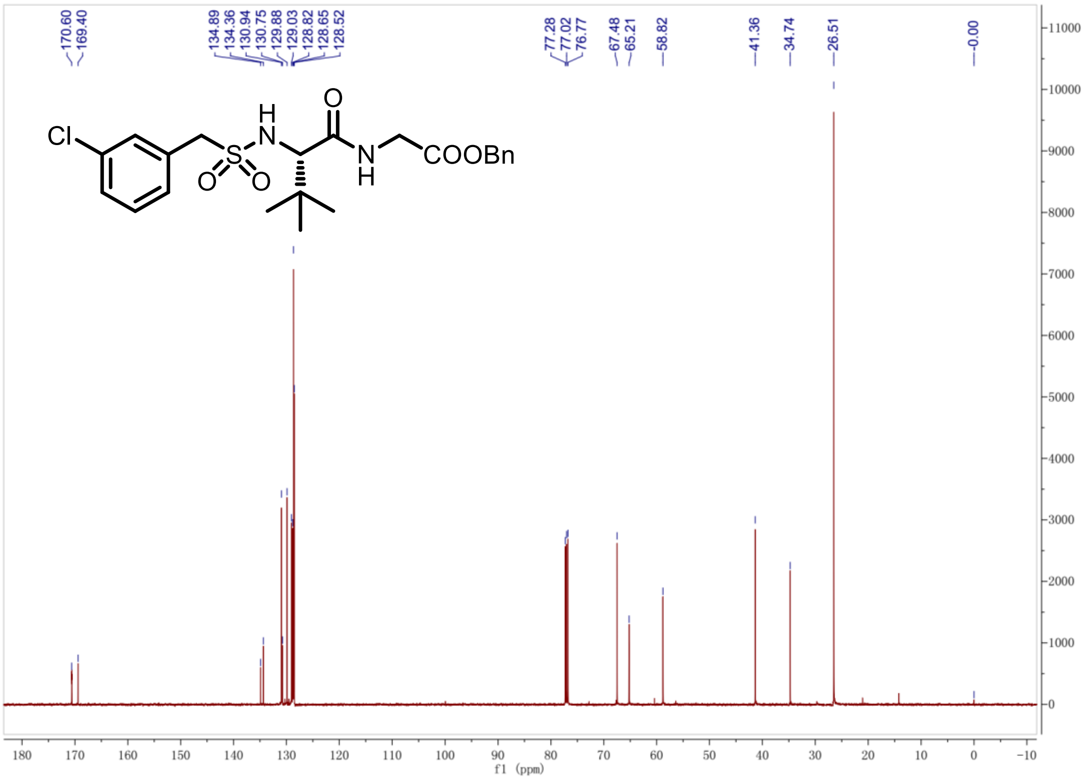

Supplementary Figure 32. ^{13}C NMR (125 MHz, $CDCl_3$) spectrum of compound **1f**

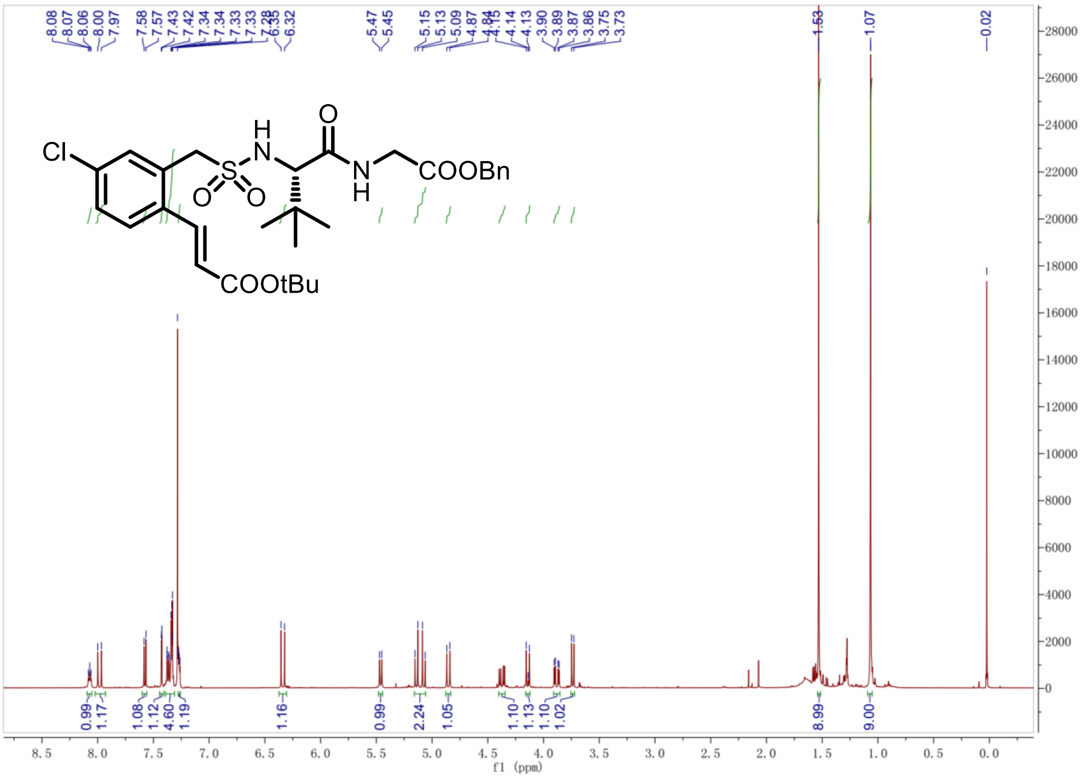

Supplementary Figure 33. ^{1}H NMR (500 MHz, $CDCl_3$) spectrum of **3fa** (mono)

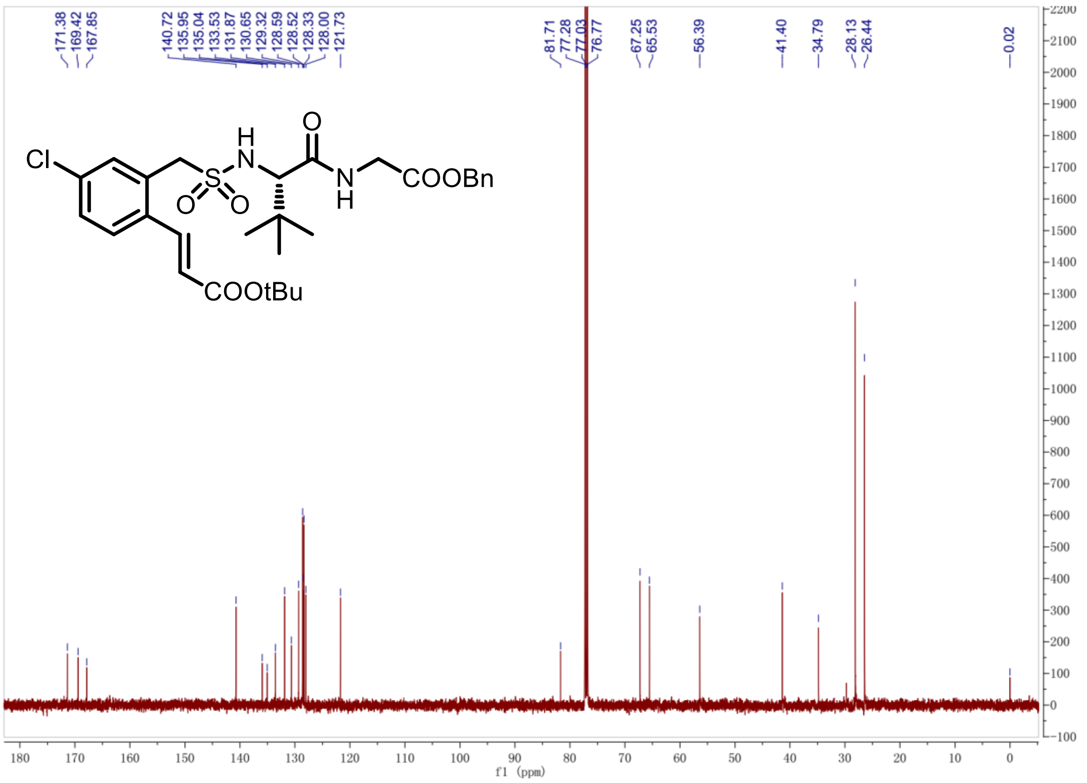

Supplementary Figure 34. ^{13}C NMR (125 MHz, $CDCl_3$) spectrum of **3fa** (mono)

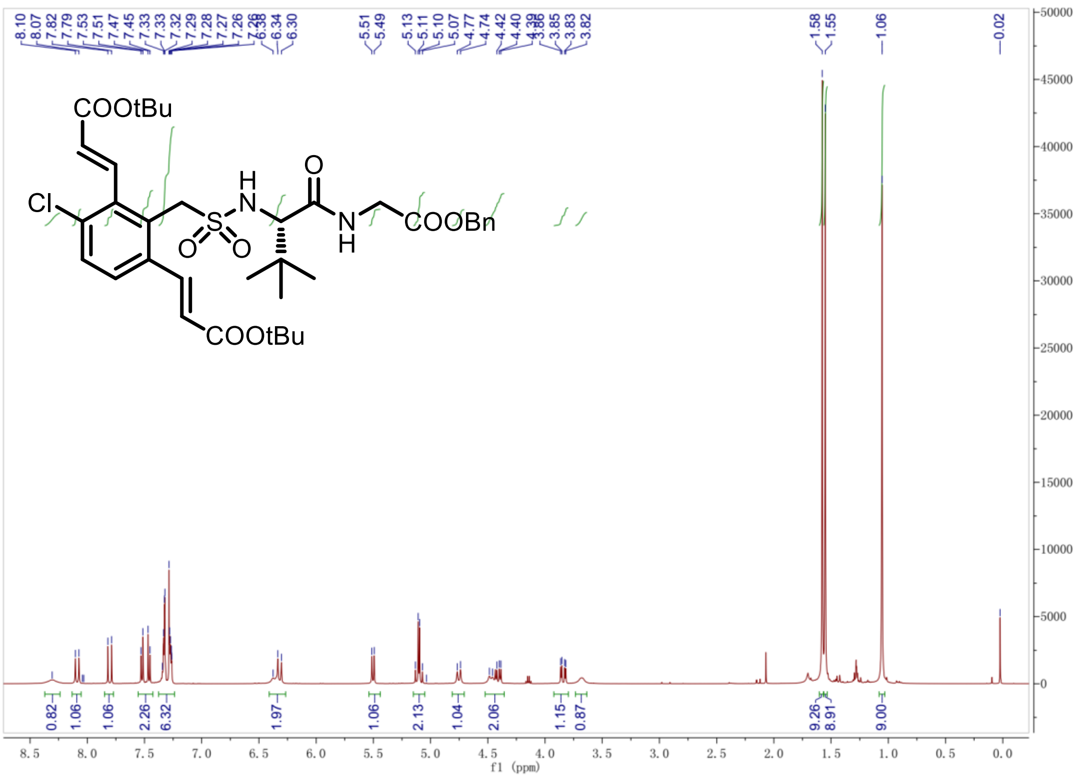

Supplementary Figure 35. ^{1}H NMR (500 MHz, $CDCl_3$) spectrum of **3fa** (di)

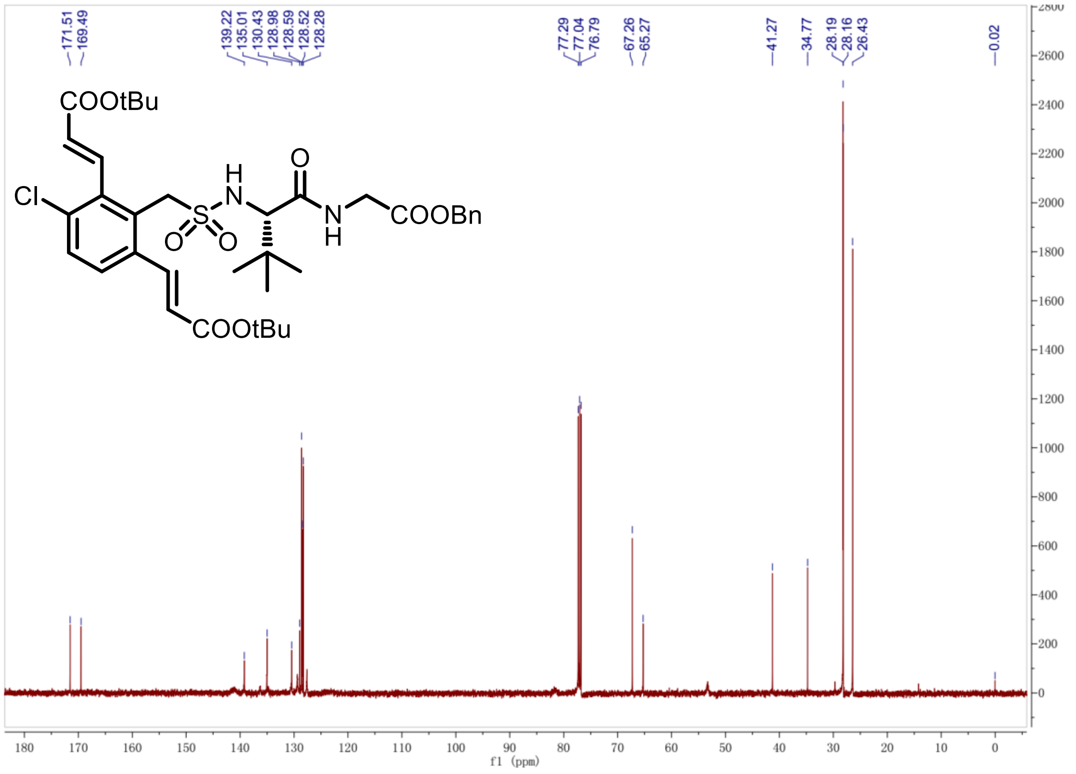

Supplementary Figure 36. ^{13}C NMR (125 MHz, $CDCl_3$) spectrum of **3fa** (di)

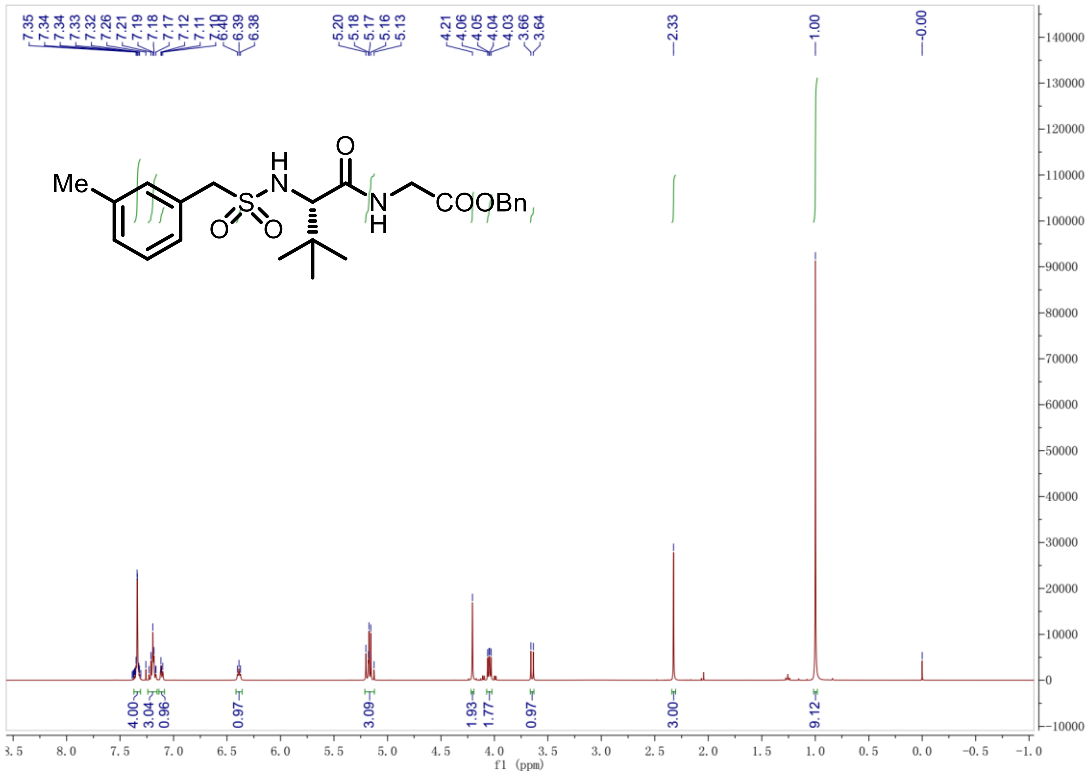

Supplementary Figure 37. ^{1}H NMR (400 MHz, $CDCl_3$) spectrum of compound **1g**

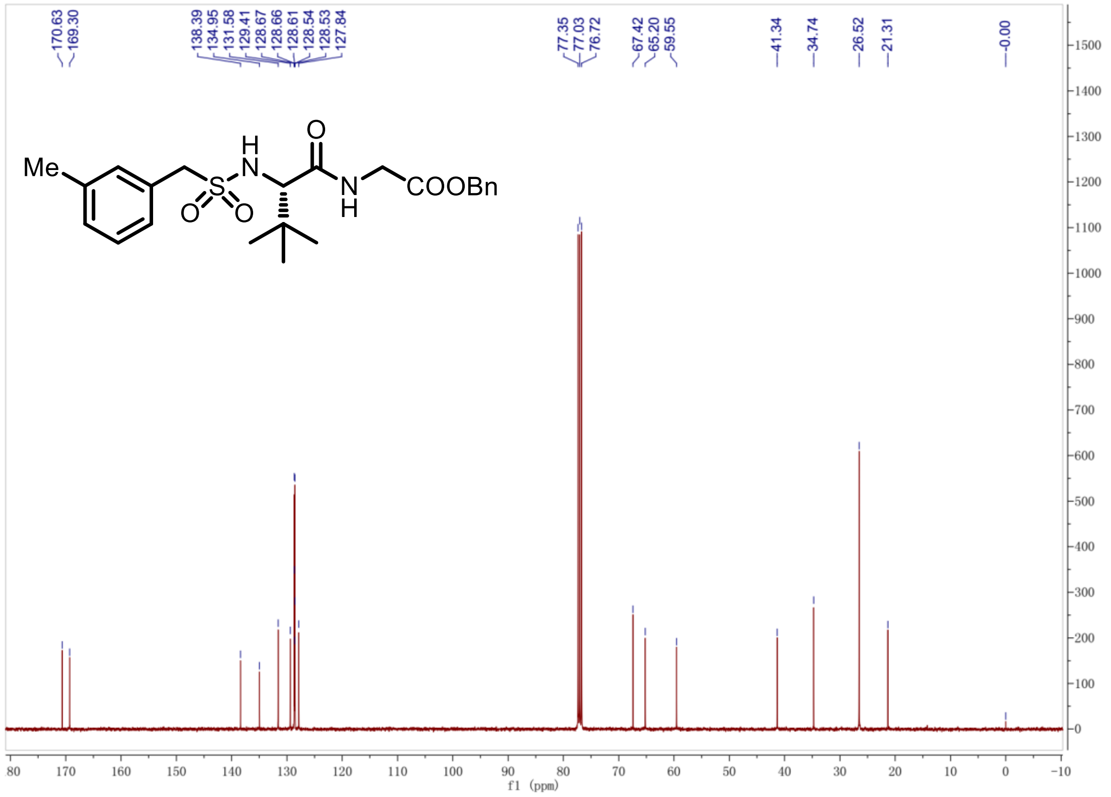

Supplementary Figure 38. ^{13}C NMR (100 MHz, $CDCl_3$) spectrum of compound **1g**

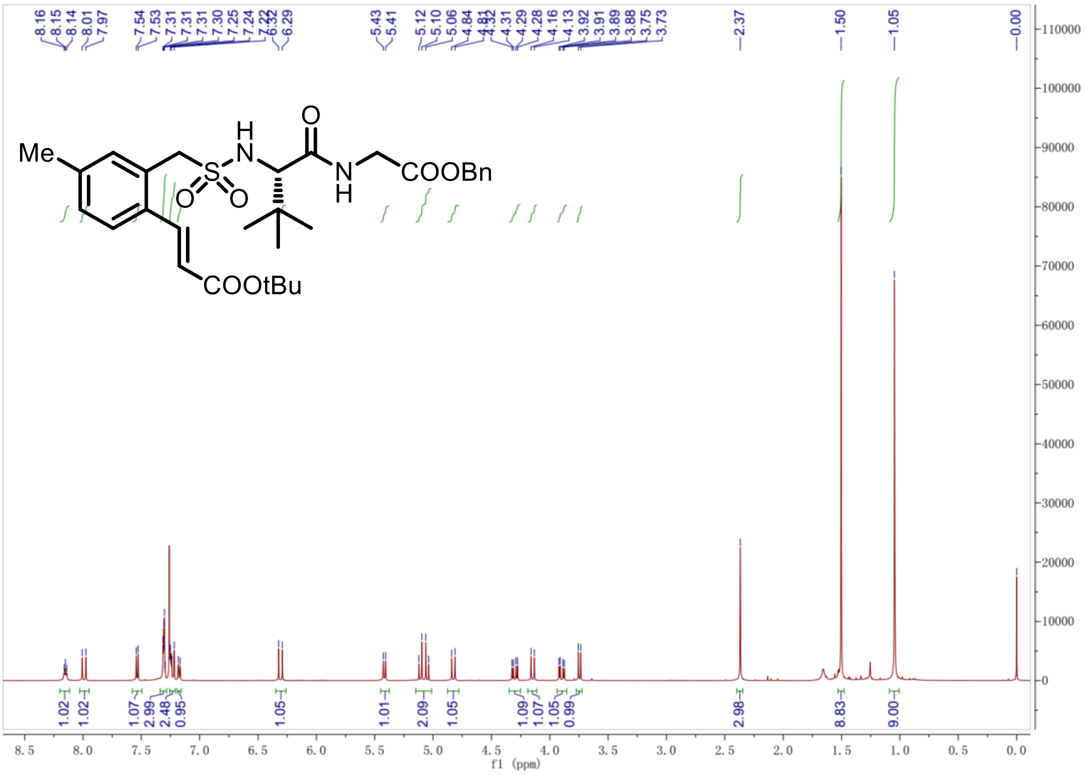

Supplementary Figure 39. ^{1}H NMR (500 MHz, $CDCl_3$) spectrum of **3ga** (mono)

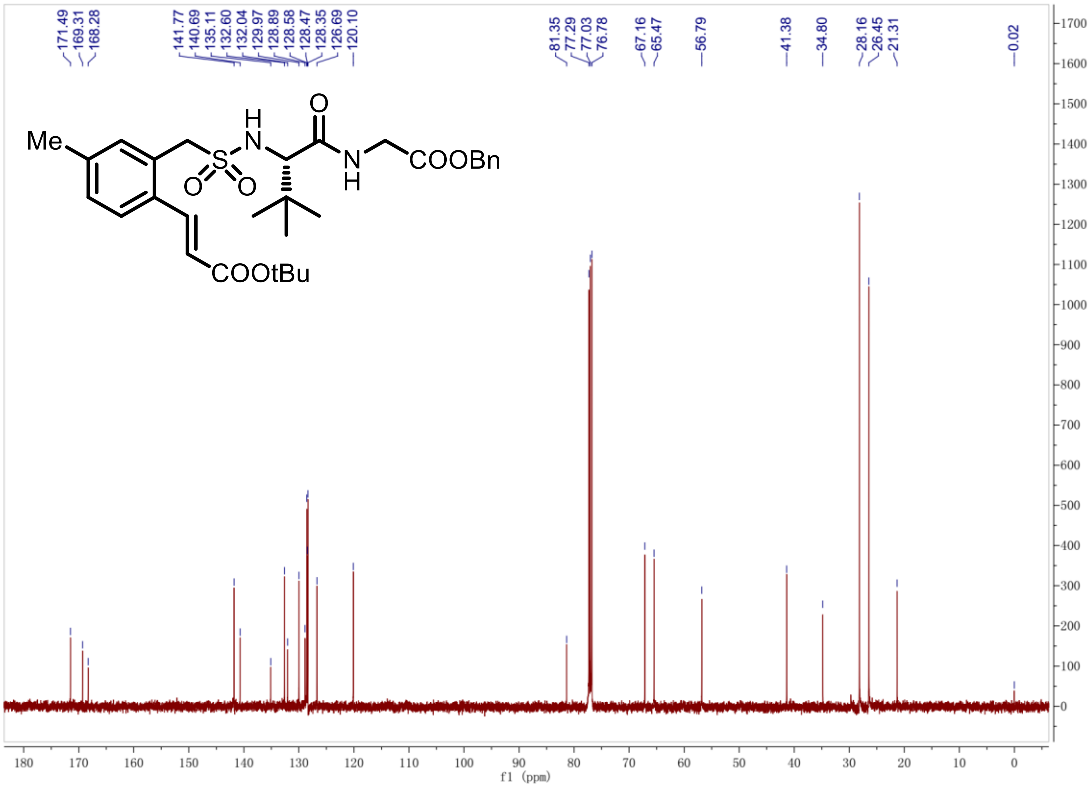

Supplementary Figure 40. ^{13}C NMR (125 MHz, $CDCl_3$) spectrum of **3ga** (mono)

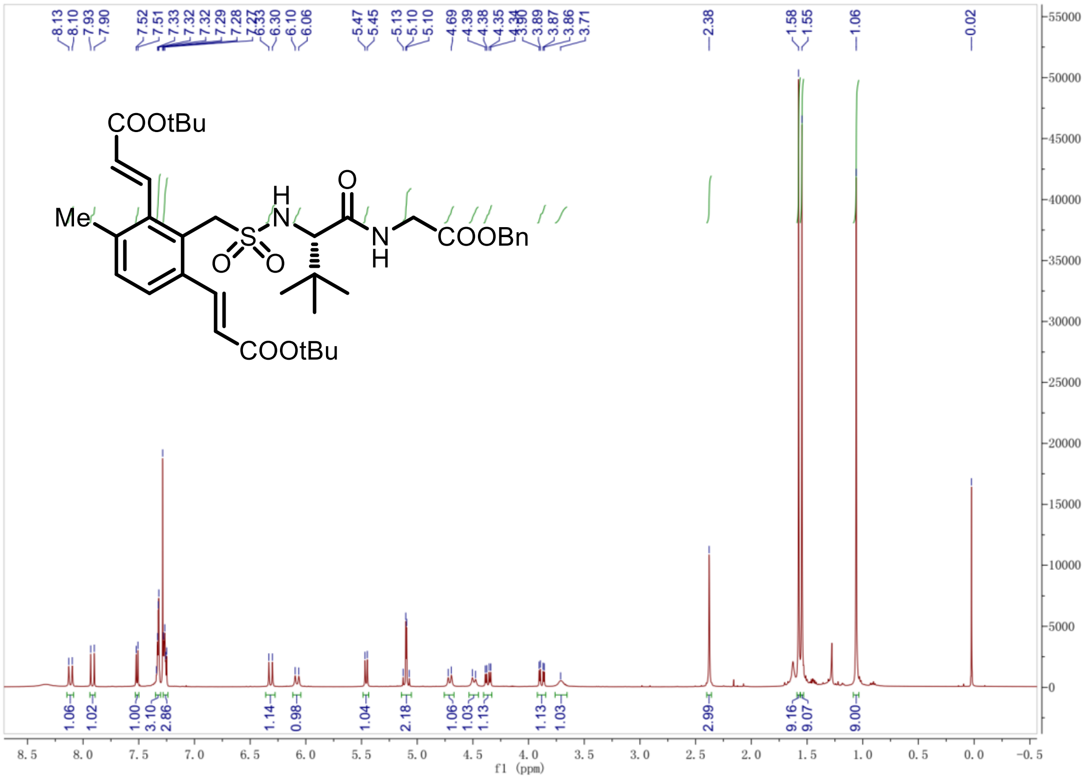

Supplementary Figure 41. ^{1}H NMR (500 MHz, $CDCl_3$) spectrum of **3ga** (di)

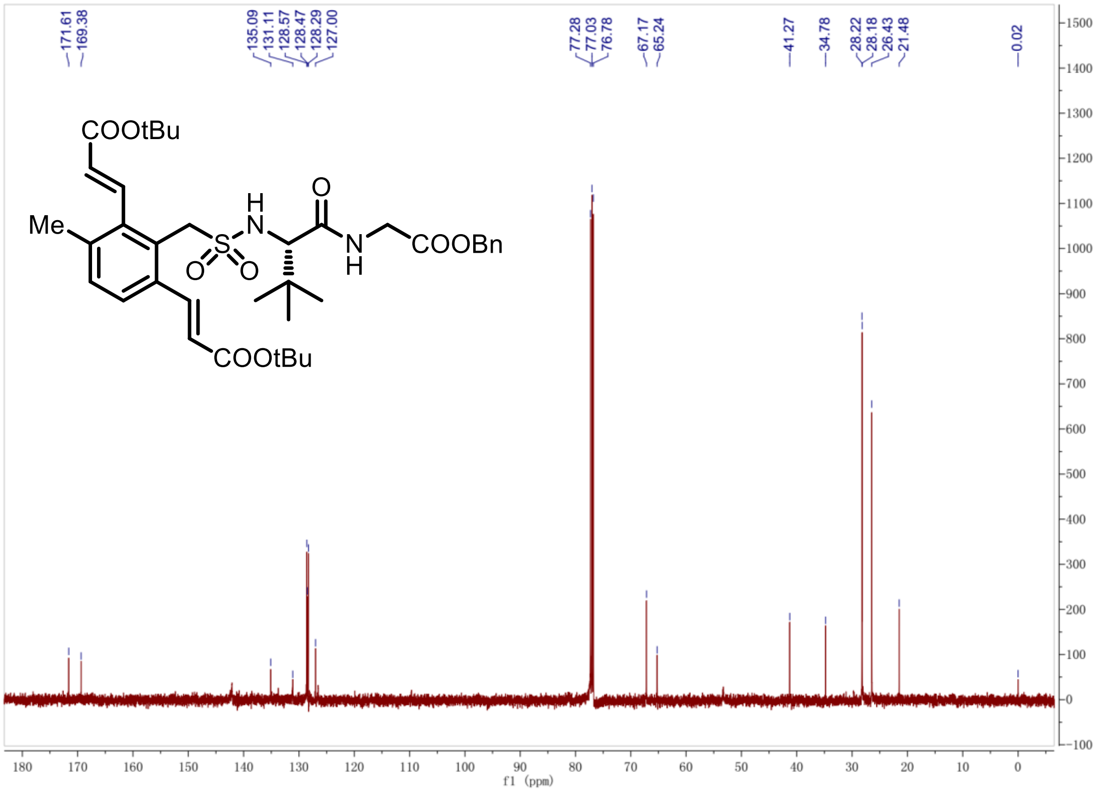

Supplementary Figure 42. ^{13}C NMR (125 MHz, $CDCl_3$) spectrum of **3ga** (di)

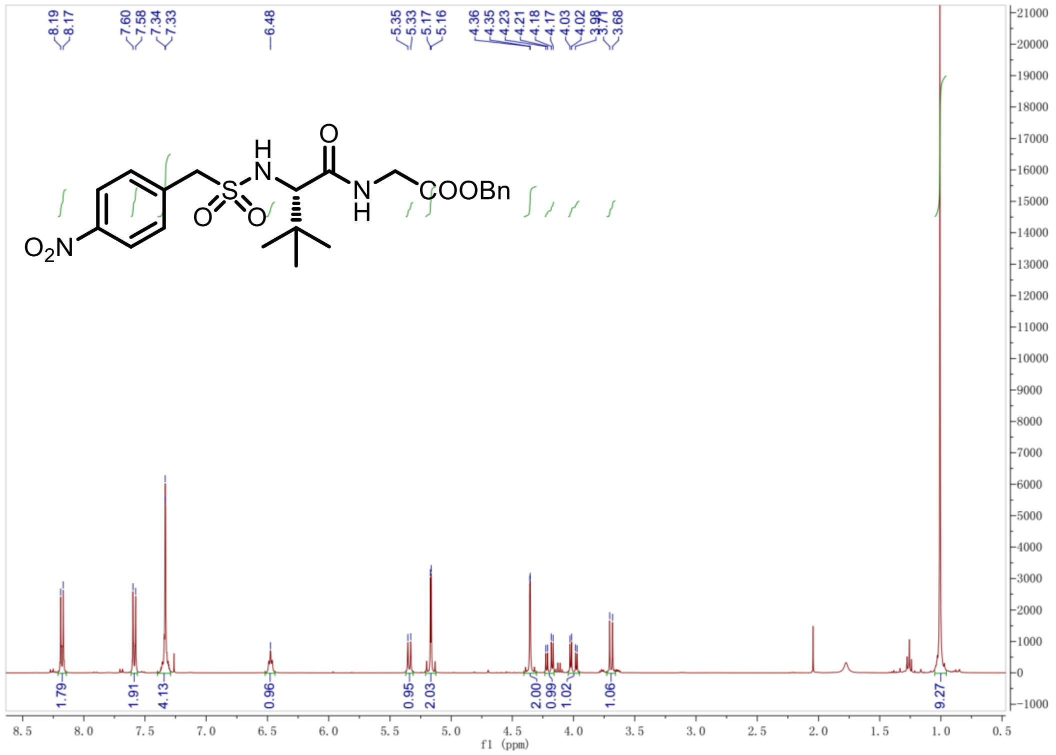

Supplementary Figure 43. ^{1}H NMR (400 MHz, $CDCl_3$) spectrum of compound **1h**

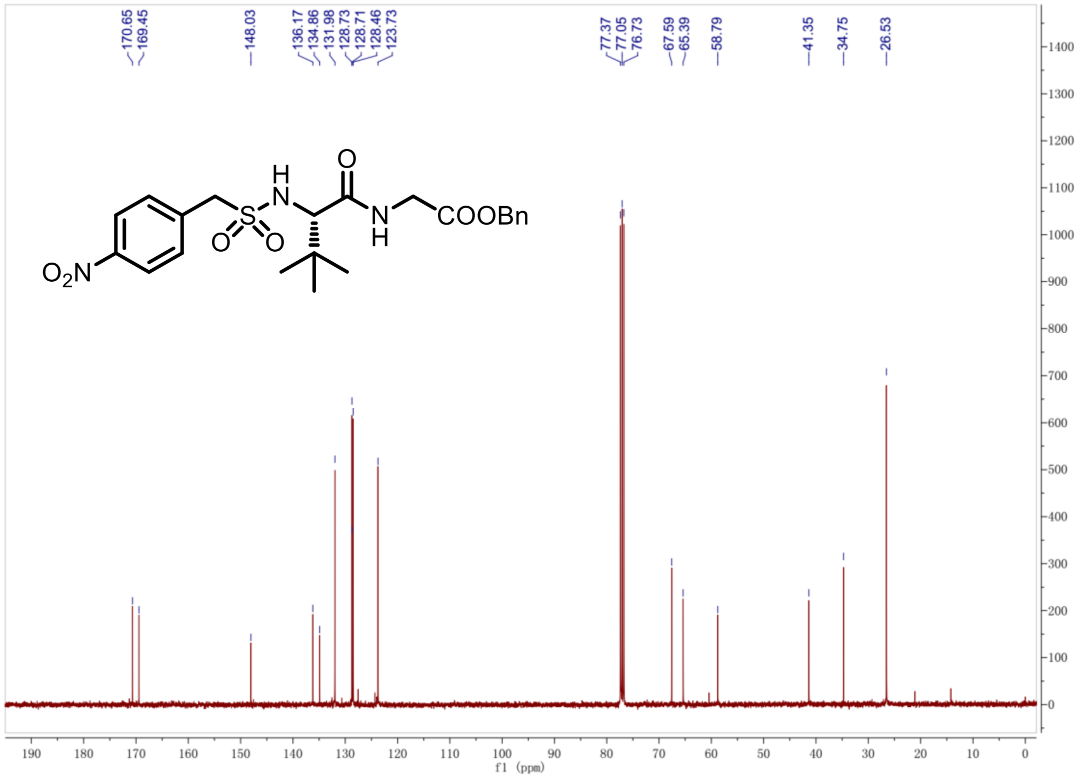

Supplementary Figure 44. ^{13}C NMR (100 MHz, $CDCl_3$) spectrum of compound **1h**

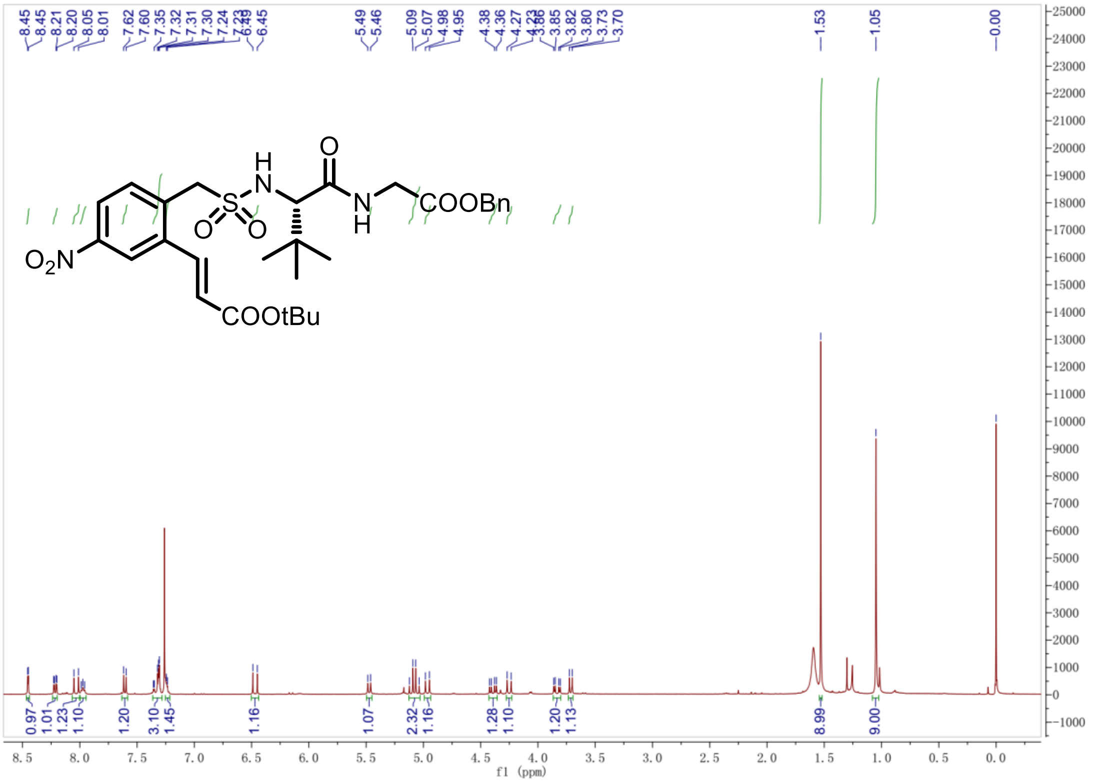

Supplementary Figure 45. ^{1}H NMR (400 MHz, $CDCl_3$) spectrum of **3ha** (mono)

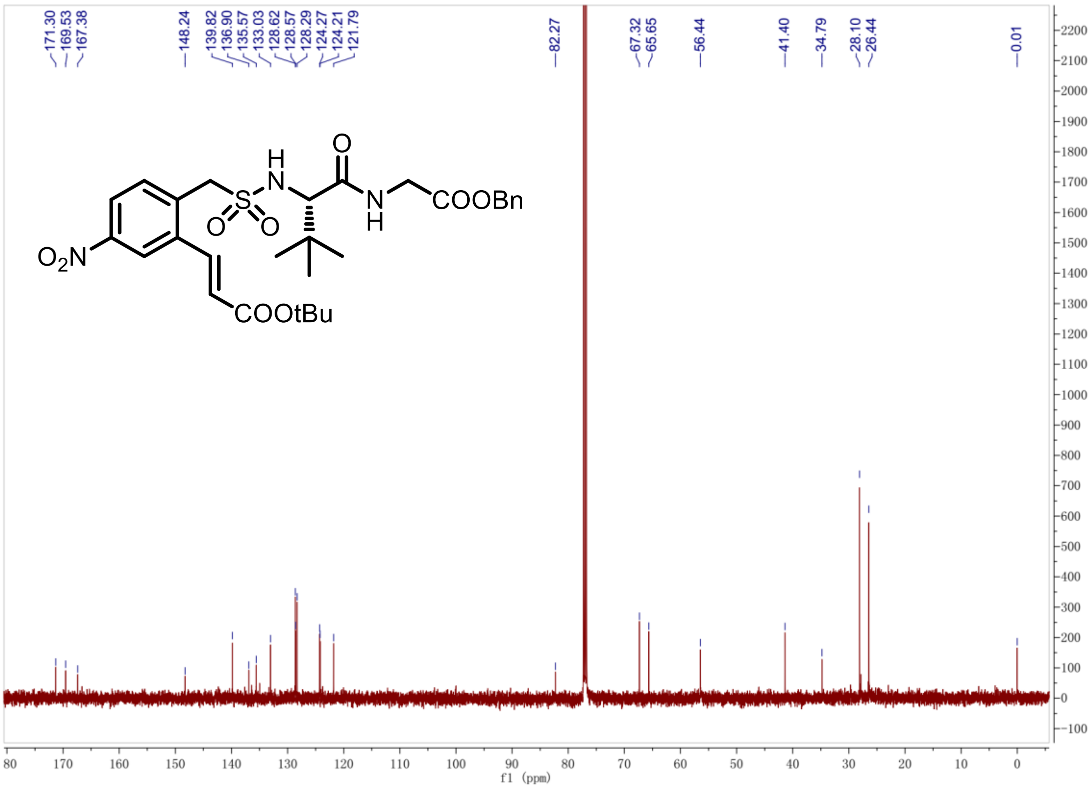

Supplementary Figure 46. ^{13}C NMR (125 MHz, $CDCl_3$) spectrum of **3ha** (mono)

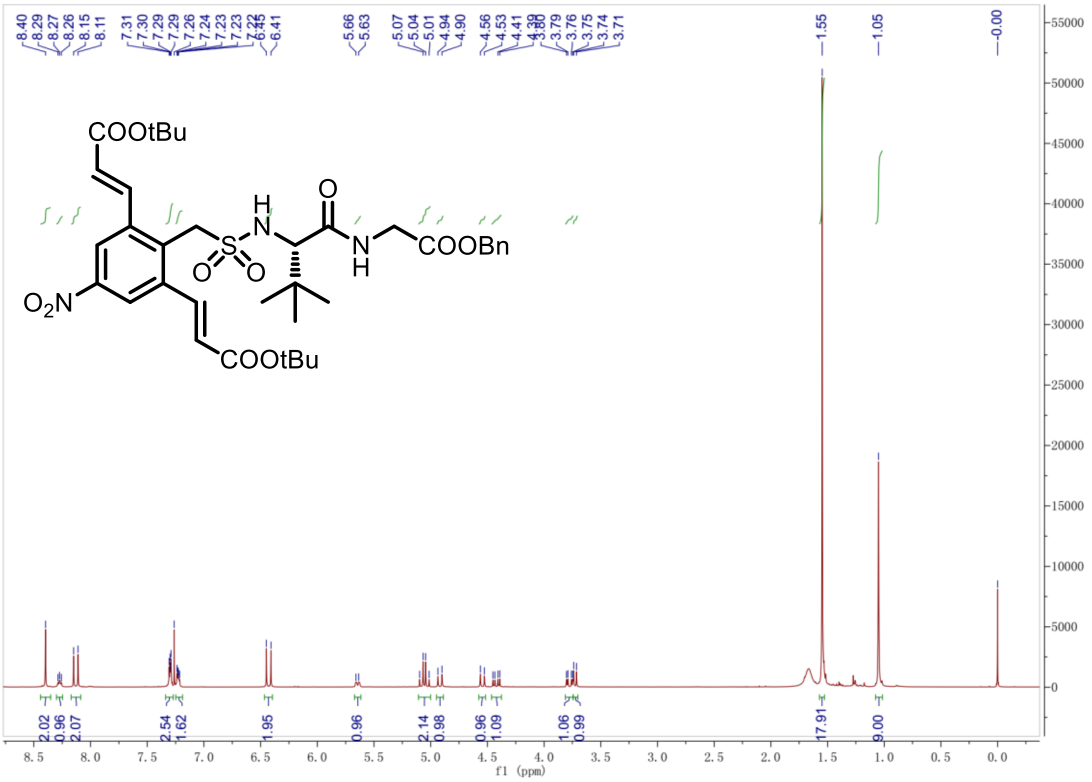

Supplementary Figure 47. ^{1}H NMR (400 MHz, $CDCl_3$) spectrum of **3ha** (di)

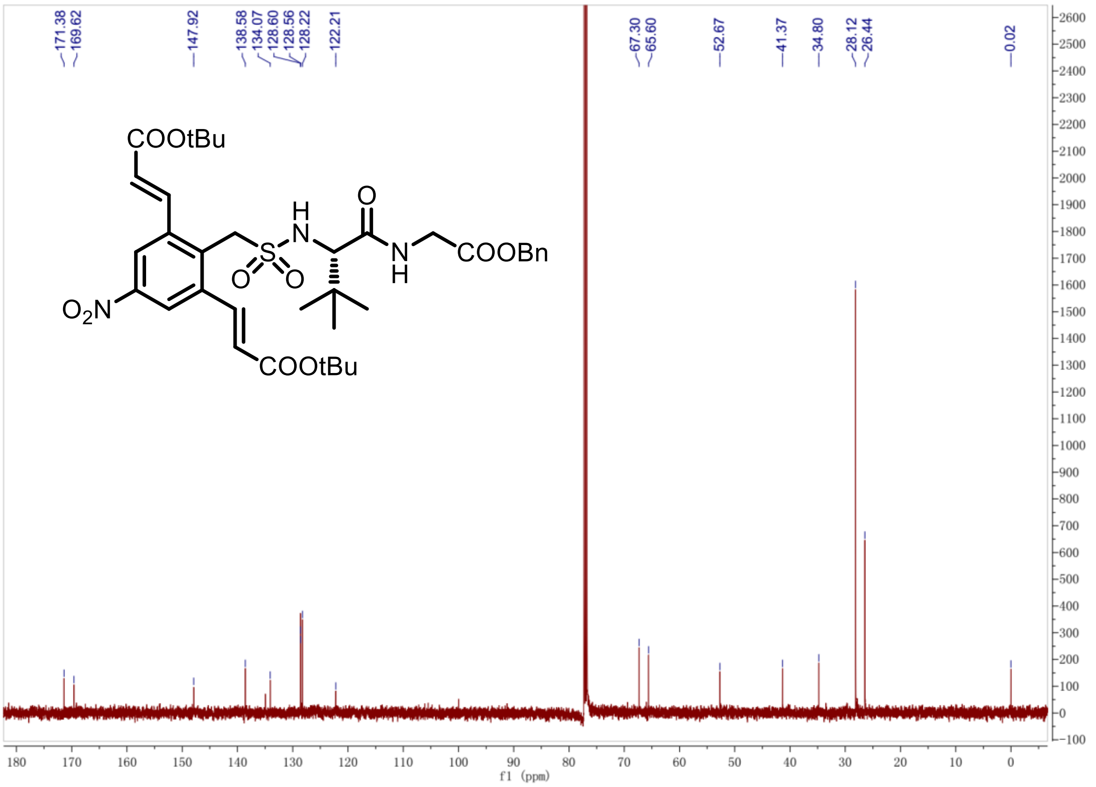

Supplementary Figure 48. ^{13}C NMR (125 MHz, $CDCl_3$) spectrum of **3ha** (di)

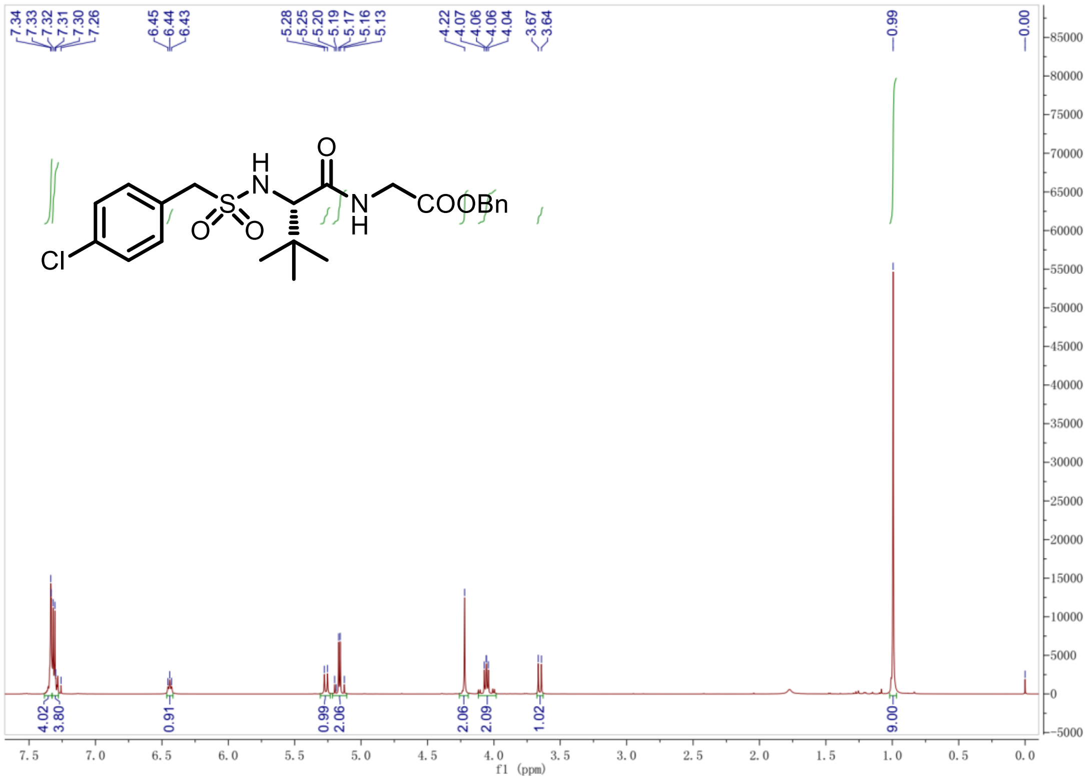

Supplementary Figure 49. ^{1}H NMR (400 MHz, $CDCl_3$) spectrum of compound **1i**

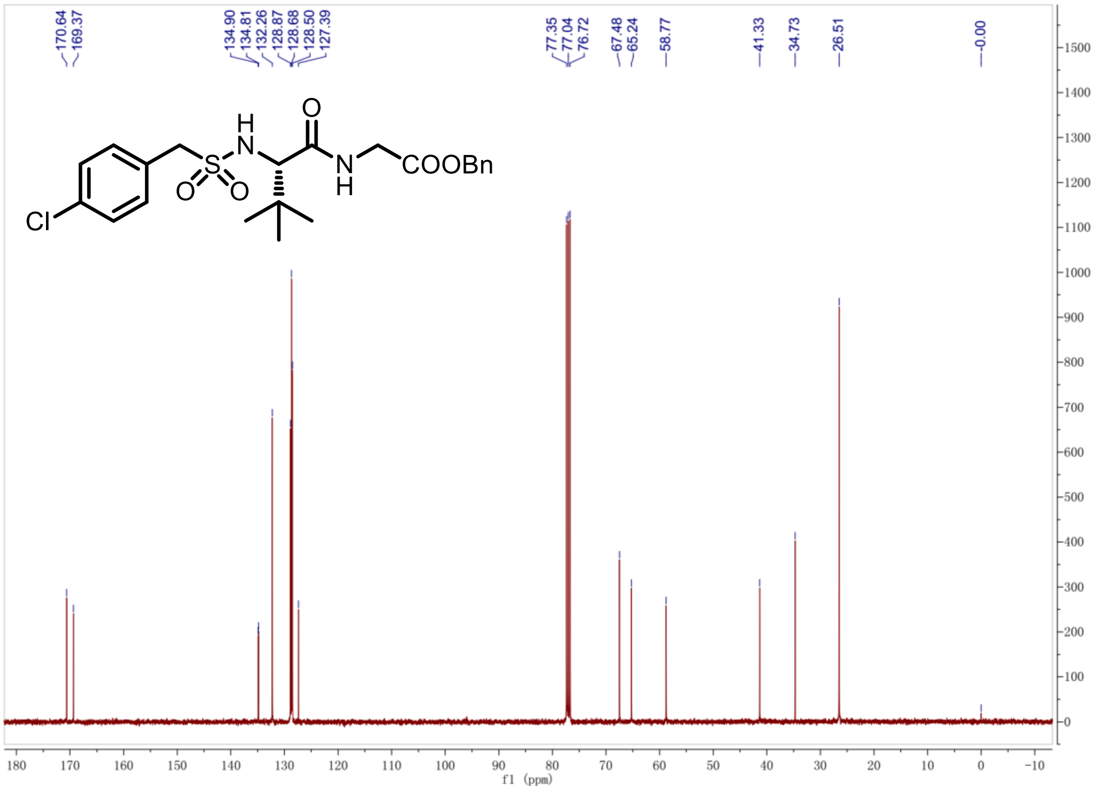

Supplementary Figure 50. ^{13}C NMR (100 MHz, $CDCl_3$) spectrum of compound **1i**

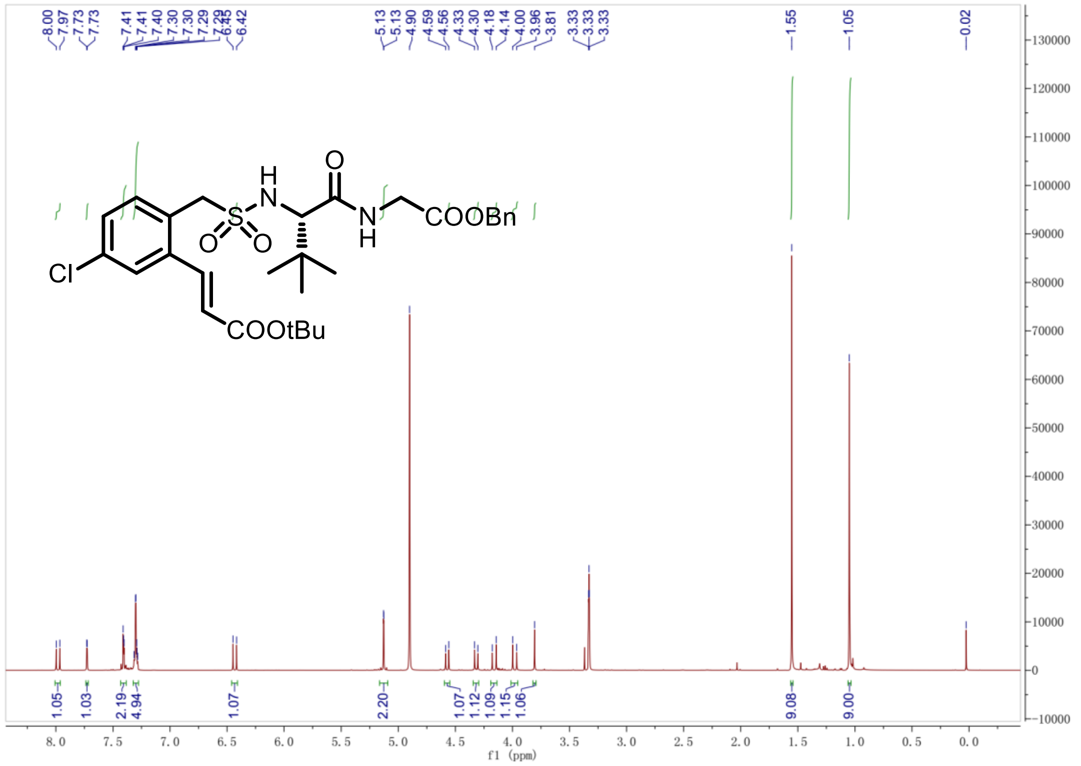

Supplementary Figure 51. ^{1}H NMR (500 MHz, MeOD) spectrum of **3ia** (mono)

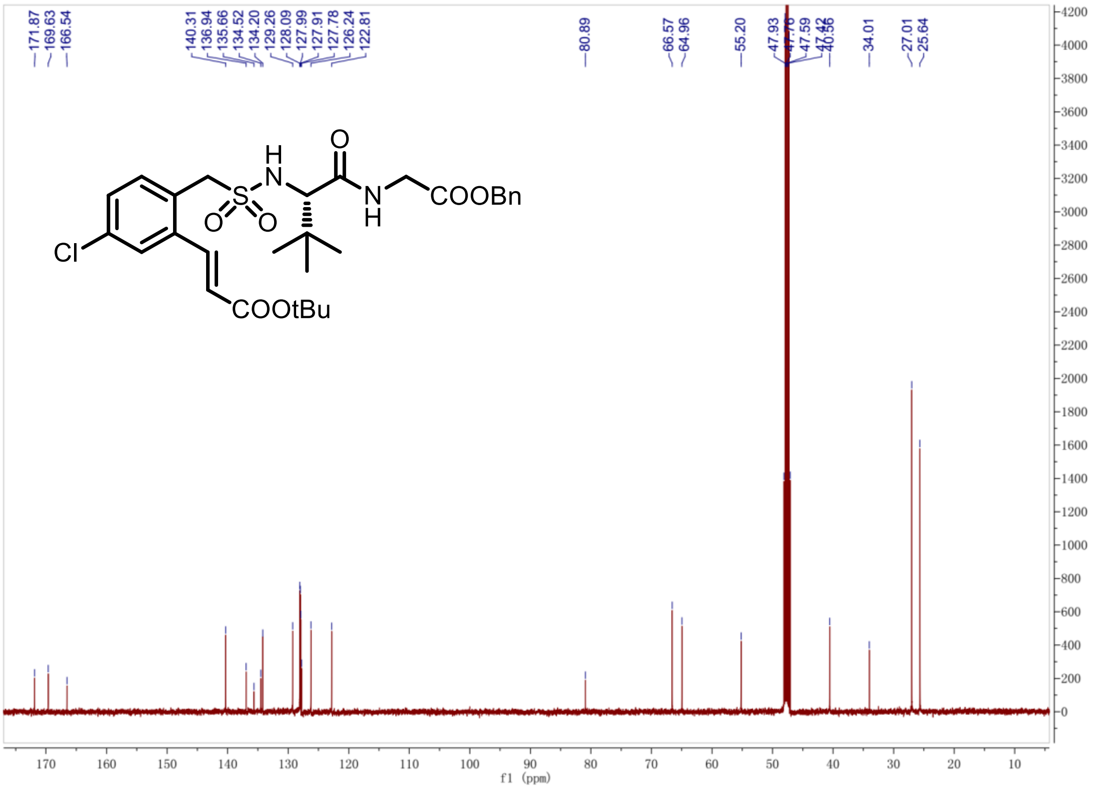

Supplementary Figure 52. ^{13}C NMR (125 MHz, MeOD) spectrum of **3ia** (mono)

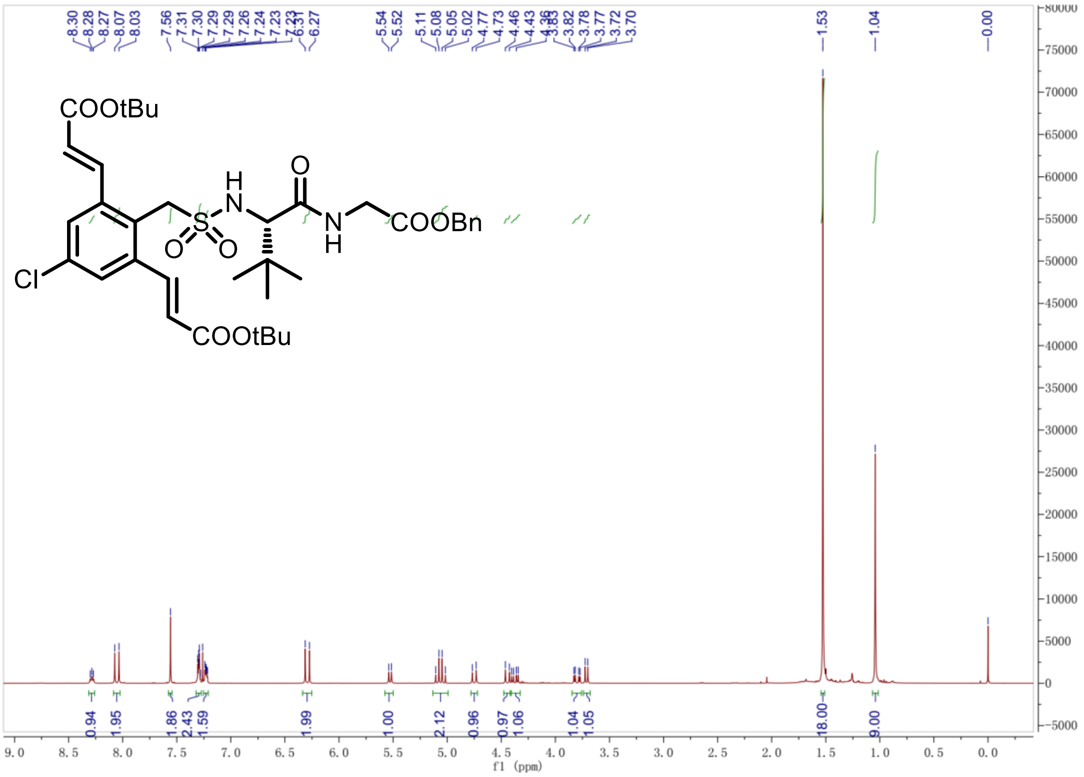

Supplementary Figure 53. ^{1}H NMR (400 MHz, $CDCl_3$) spectrum of **3ia** (di)

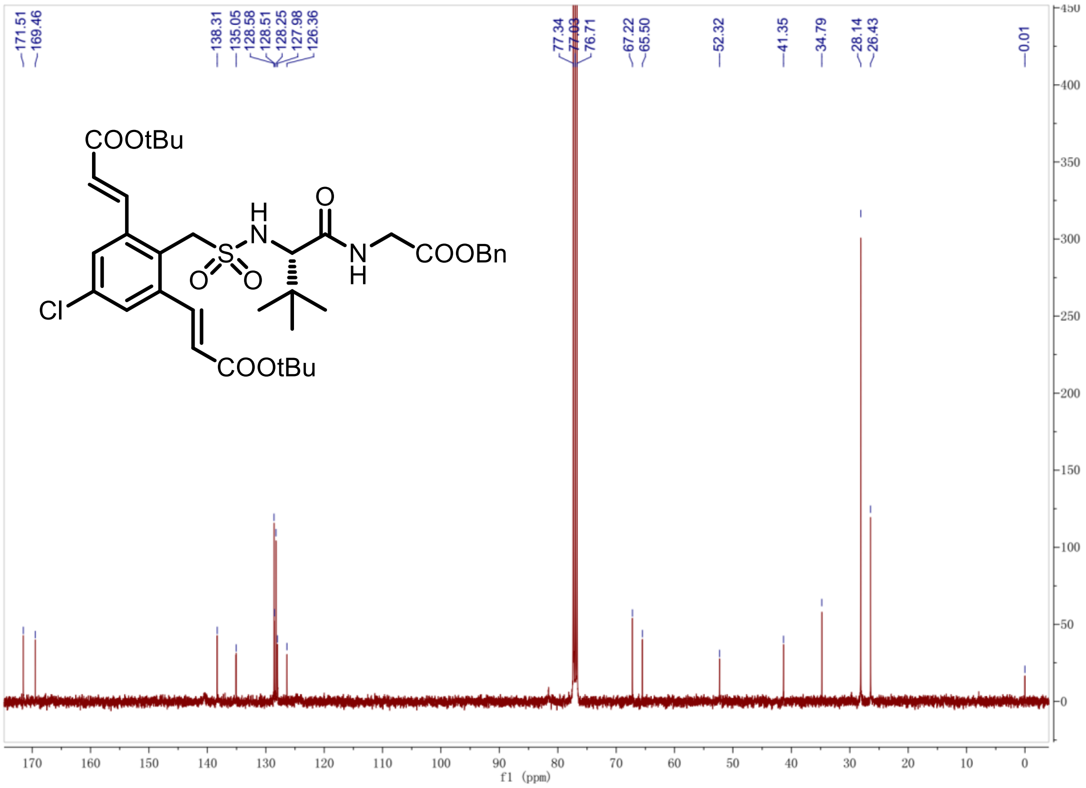

Supplementary Figure 54. ^{13}C NMR (100 MHz, $CDCl_3$) spectrum of **3ia** (di)

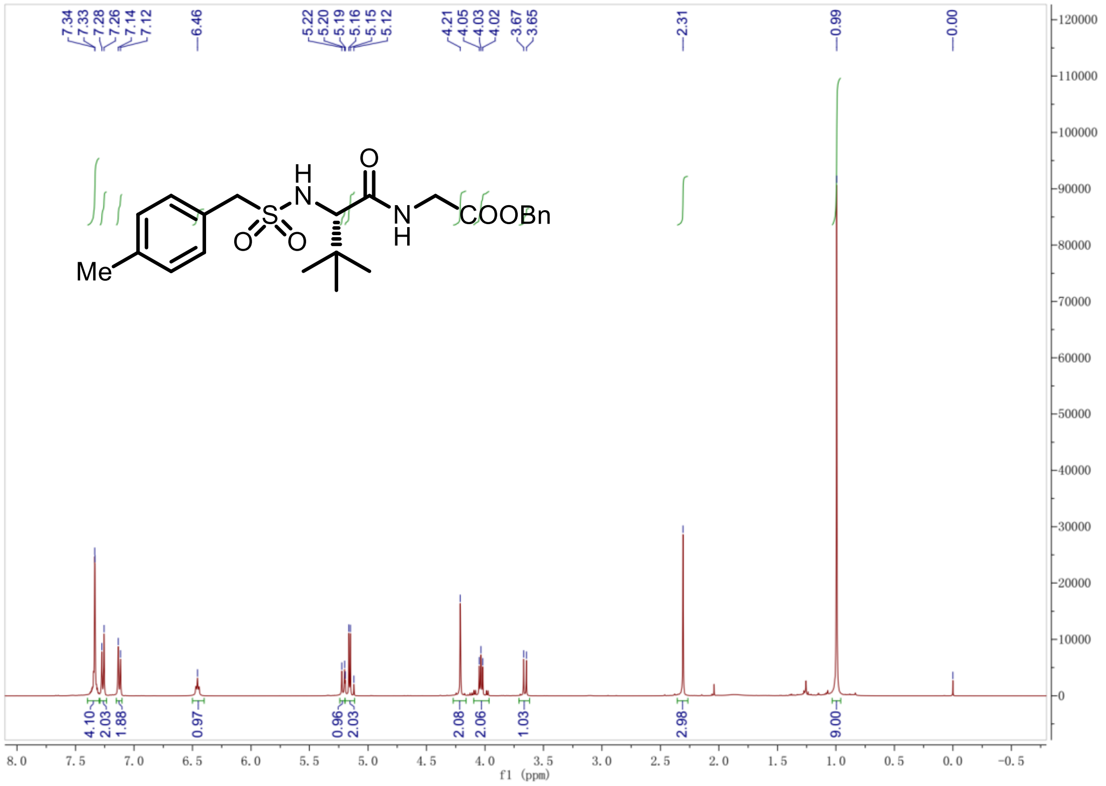

Supplementary Figure 55. ^{1}H NMR (400 MHz, $CDCl_3$) spectrum of compound **1j**

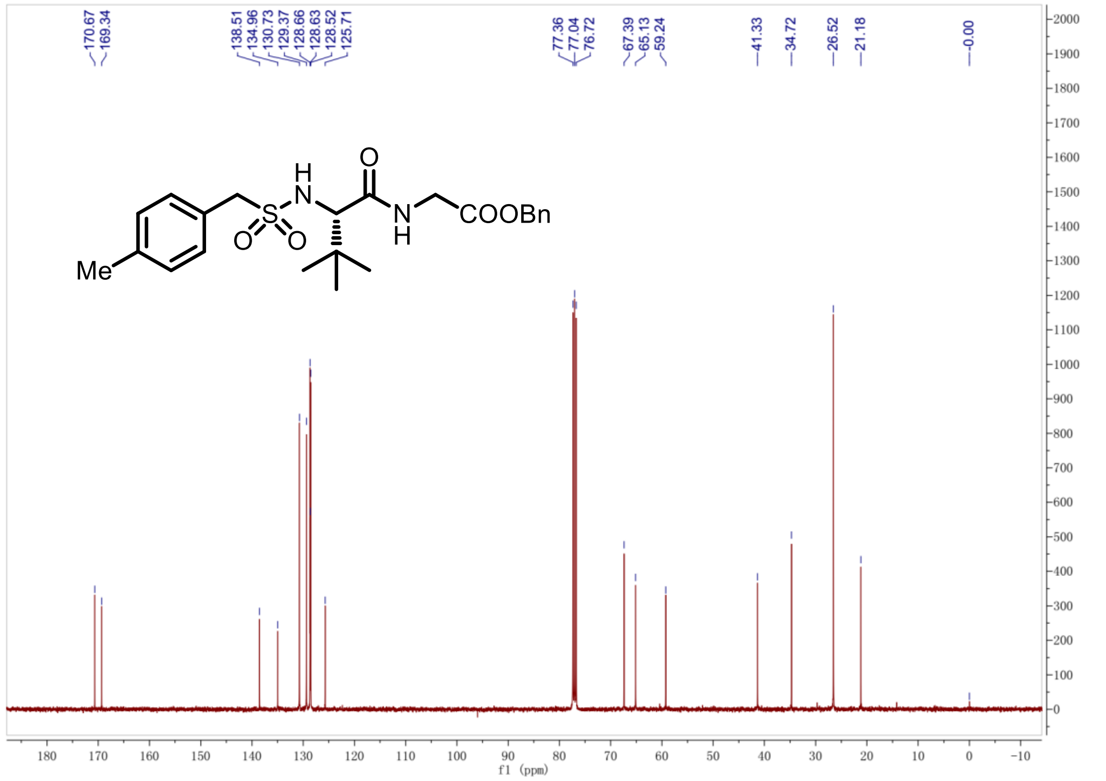

Supplementary Figure 56. ^{13}C NMR (100 MHz, $CDCl_3$) spectrum of compound **1j**

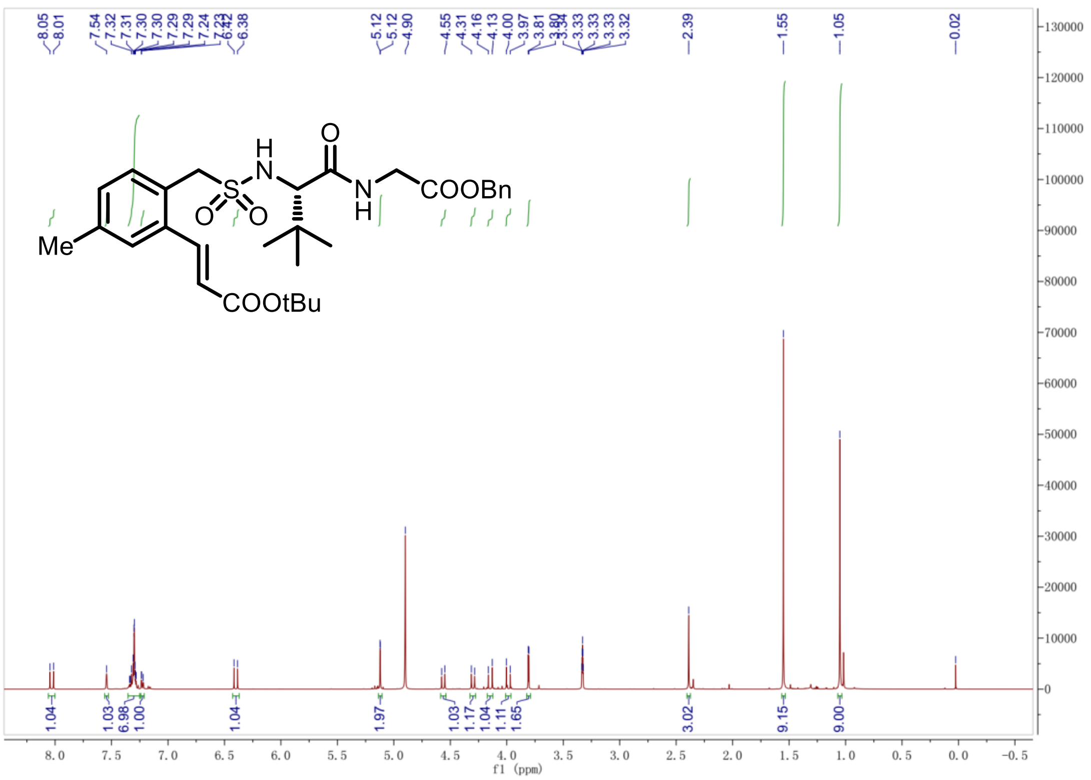

Supplementary Figure 57. ^{1}H NMR (500 MHz, MeOD) spectrum of **3ja** (mono)

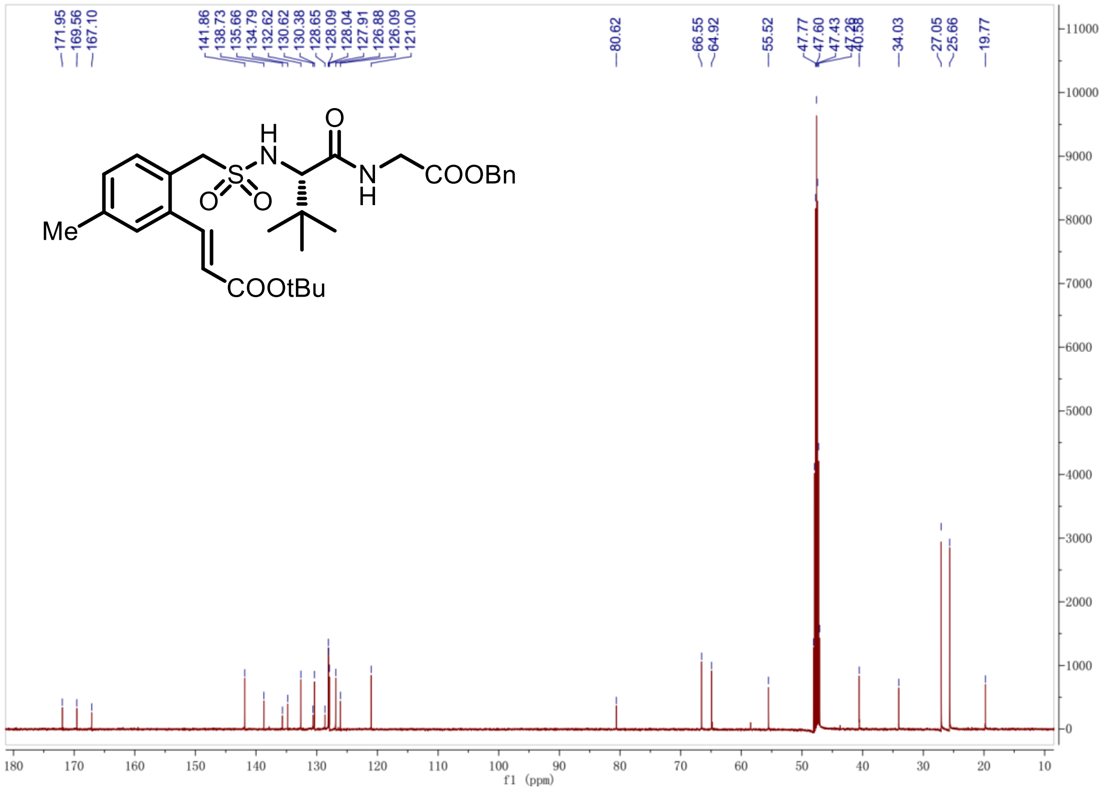

Supplementary Figure 58. ^{13}C NMR (125 MHz, MeOD) spectrum of **3ja** (mono)

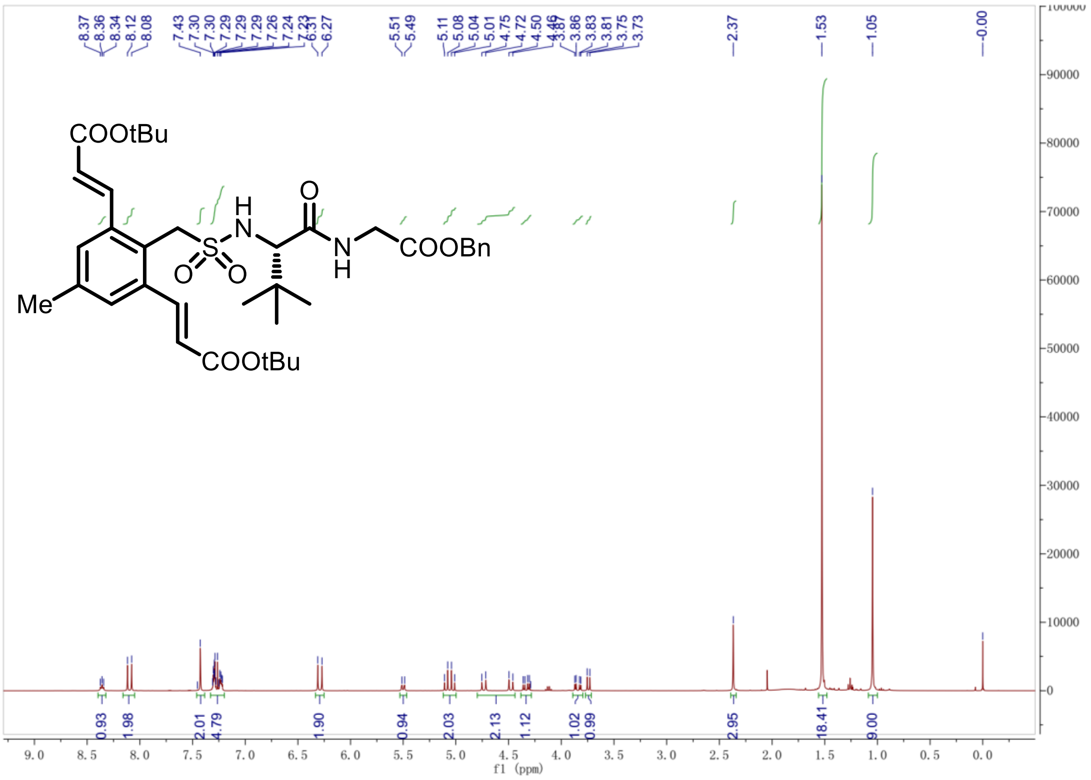

Supplementary Figure 59. ^{1}H NMR (400 MHz, $CDCl_3$) spectrum of **3ja** (di)

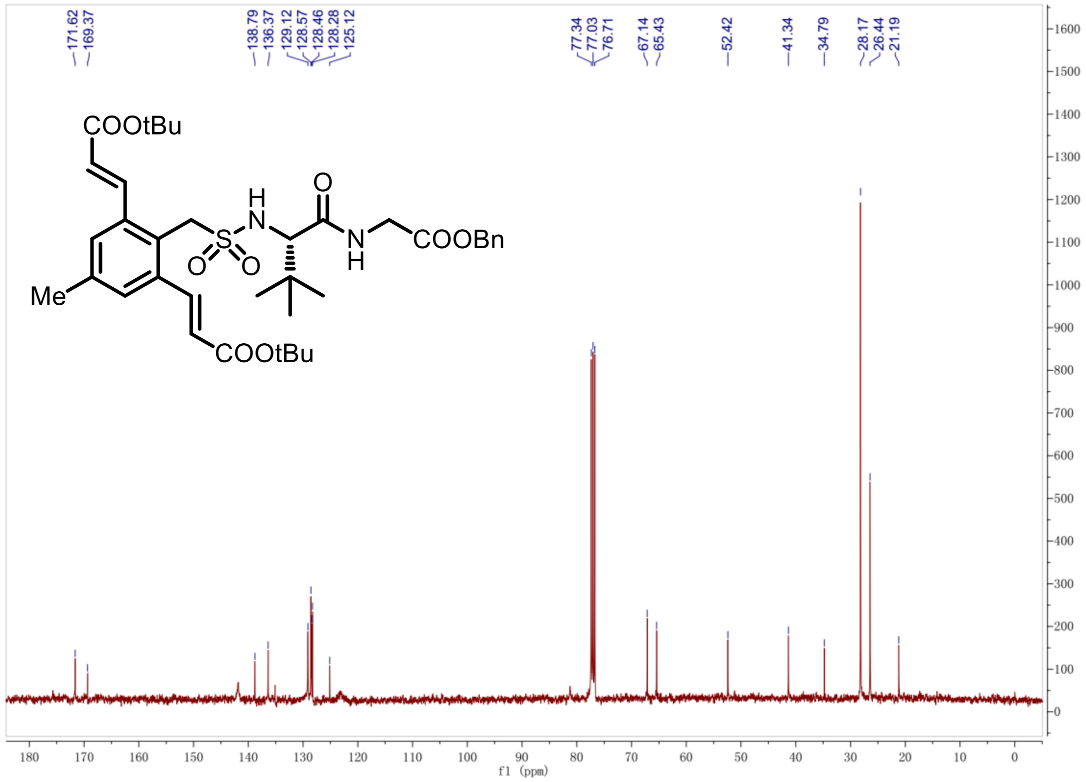

Supplementary Figure 60. ^{13}C NMR (100 MHz, $CDCl_3$) spectrum of **3ja** (di)

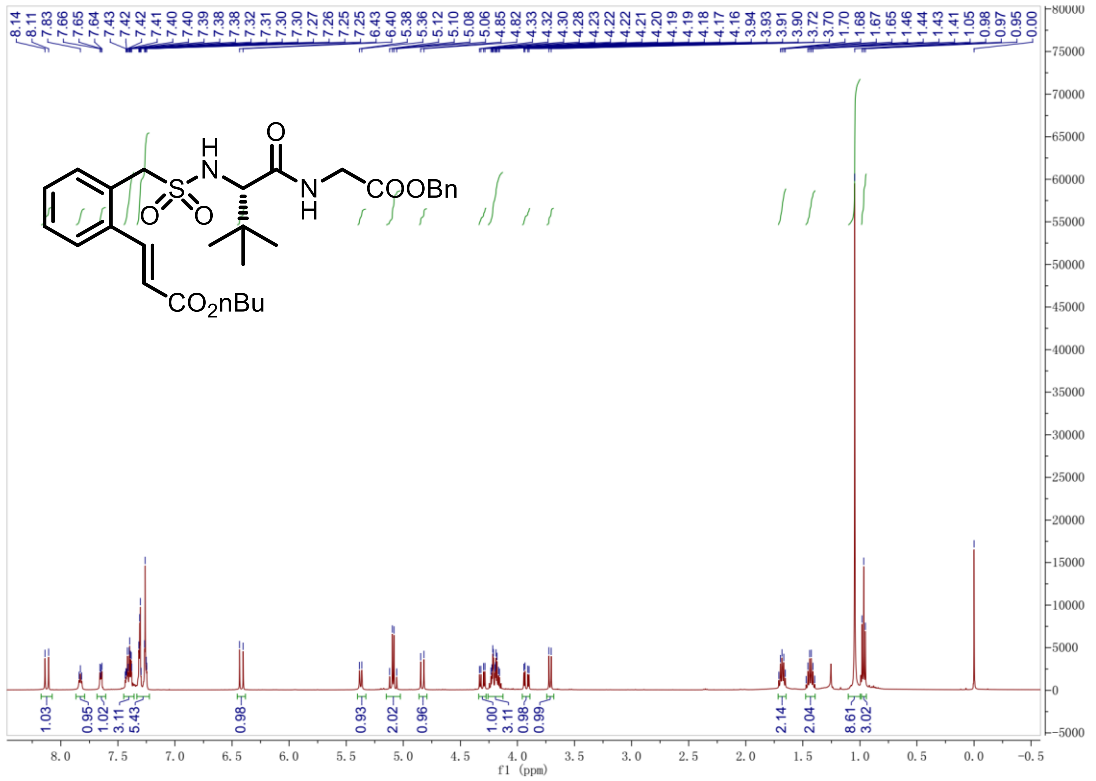

Supplementary Figure 61. ^{1}H NMR (500 MHz, $CDCl_3$) spectrum of **3db** (mono)

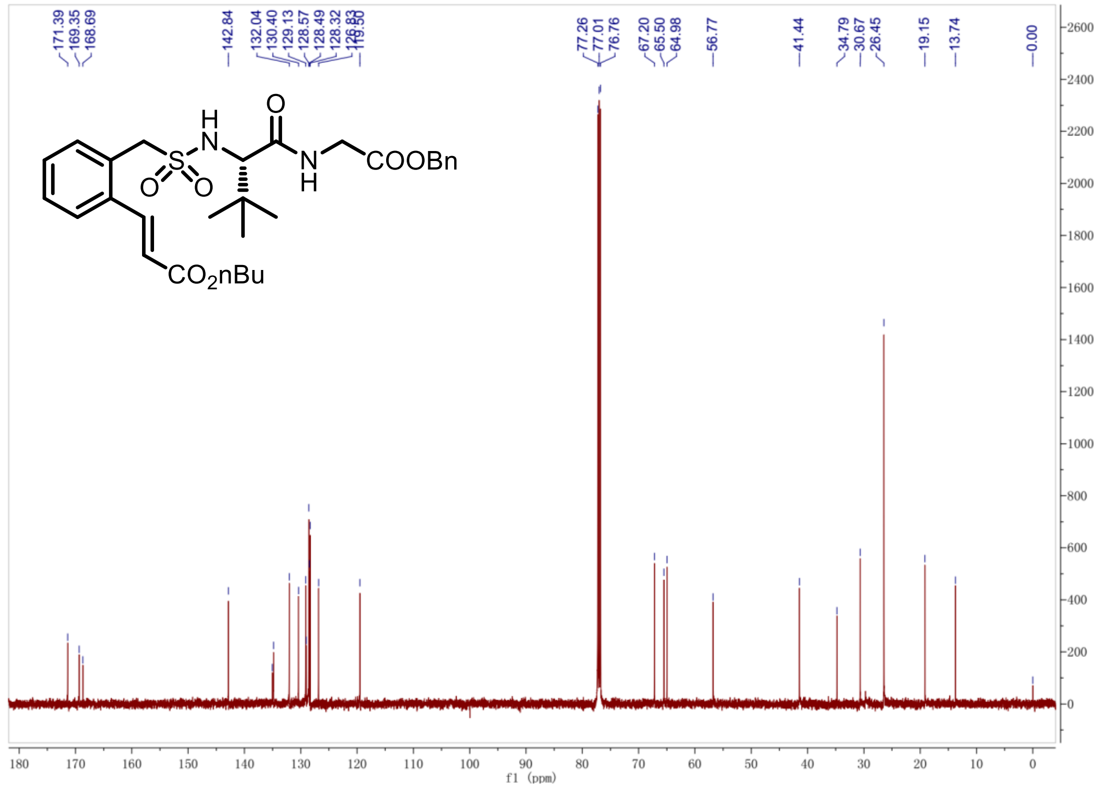

Supplementary Figure 62. ^{13}C NMR (125 MHz, $CDCl_3$) spectrum of **3db** (mono)

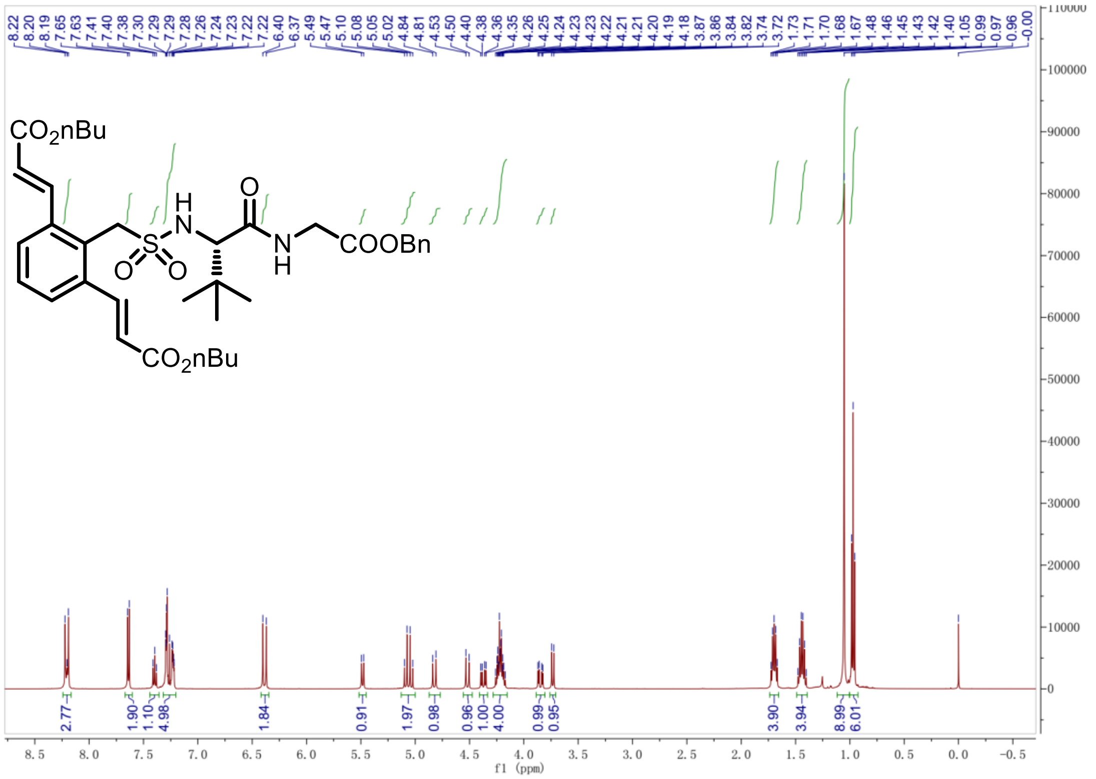

Supplementary Figure 63. ^{1}H NMR (500 MHz, $CDCl_3$) spectrum of **3db** (di)

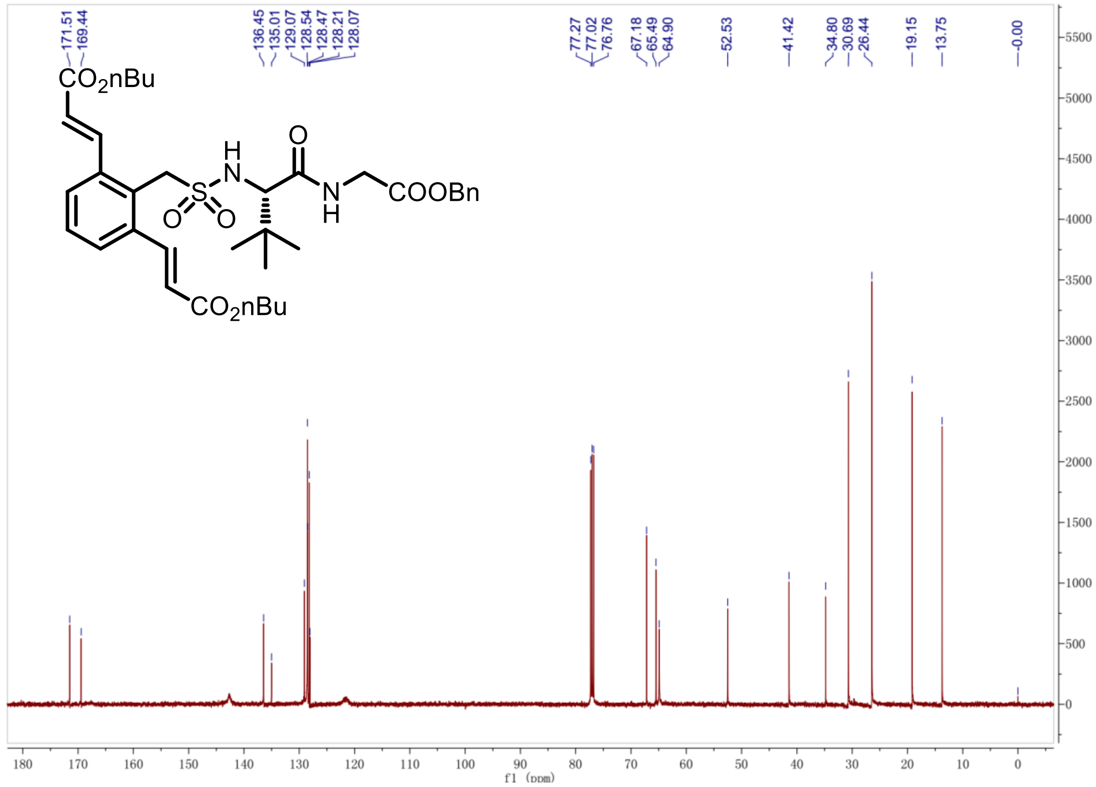

Supplementary Figure 64. ^{13}C NMR (125 MHz, $CDCl_3$) spectrum of **3db** (di)

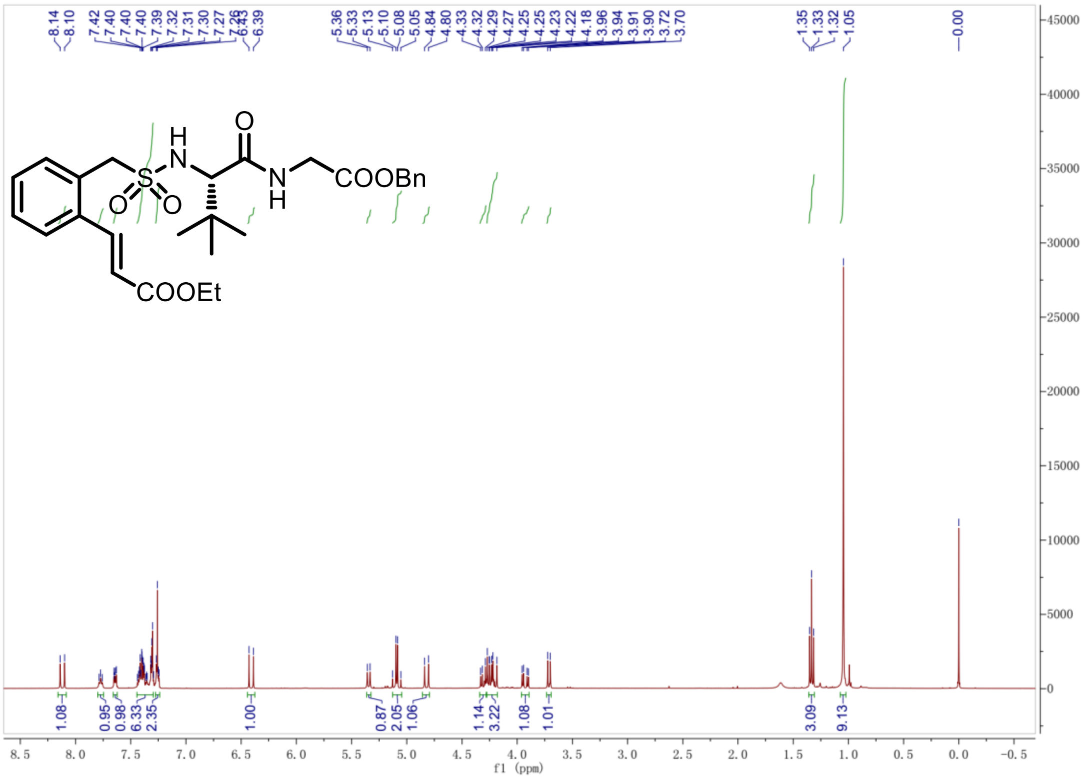

Supplementary Figure 65. ^{1}H NMR (400 MHz, $CDCl_3$) spectrum of **3dc** (mono)

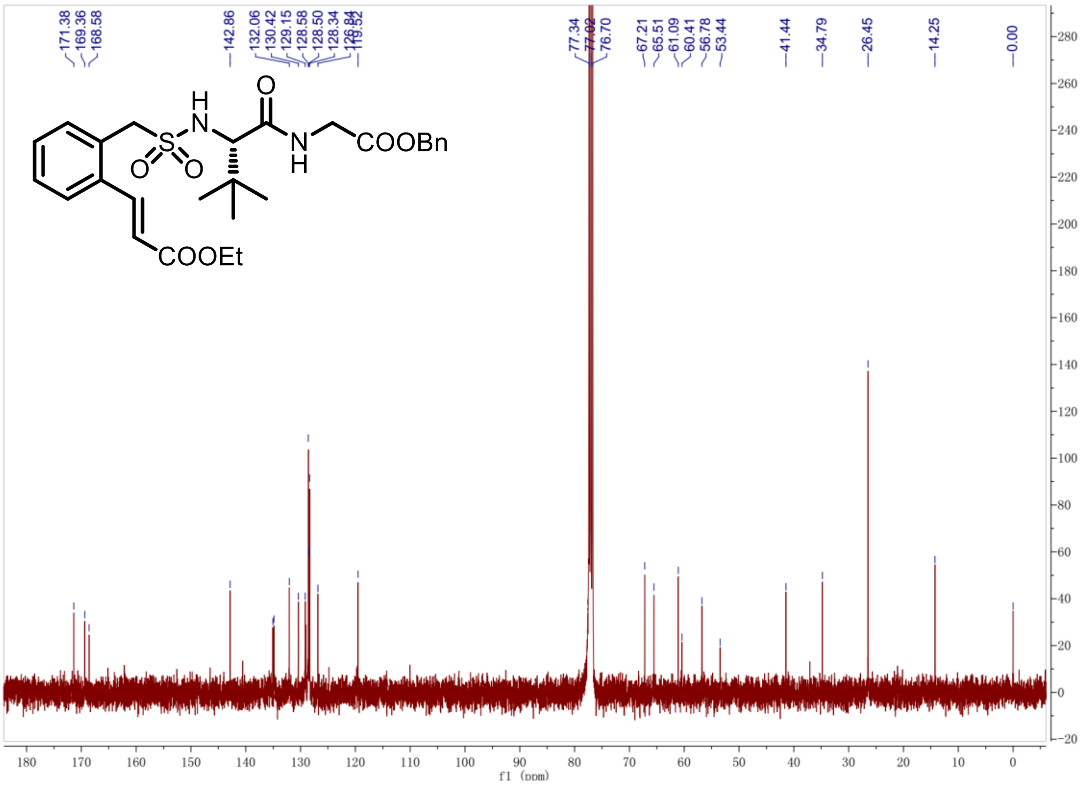

Supplementary Figure 66. ^{13}C NMR (100 MHz, $CDCl_3$) spectrum of **3dc** (mono)

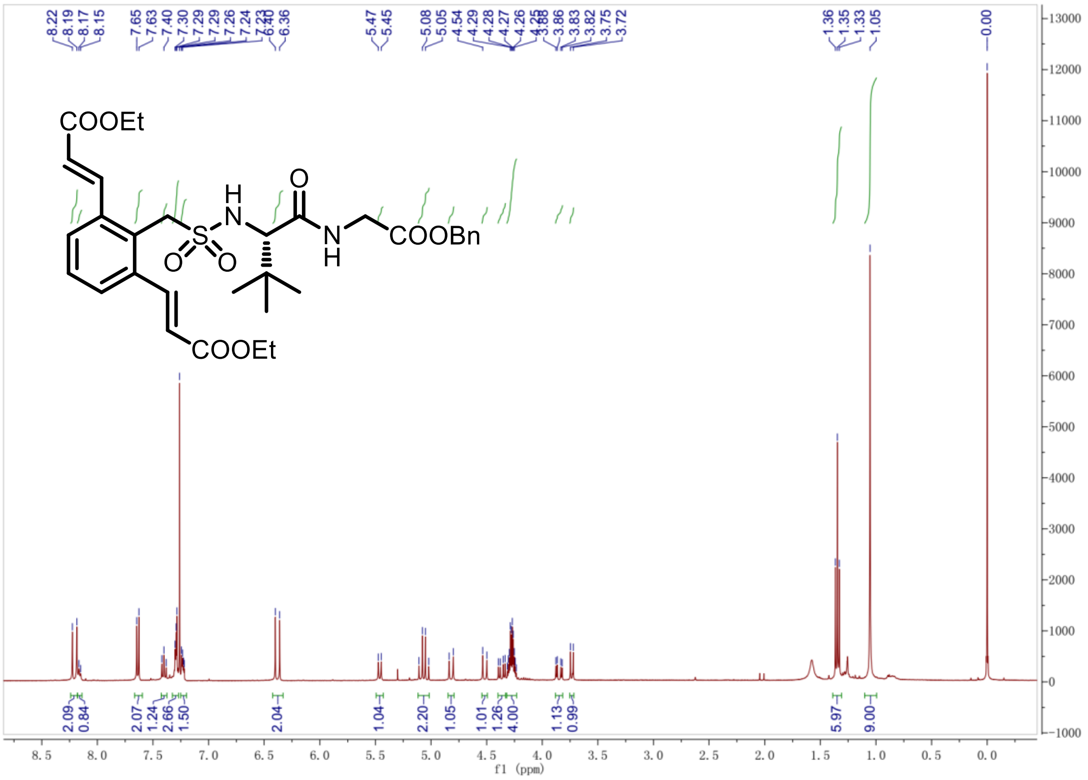

Supplementary Figure 67. ^{1}H NMR (400 MHz, $CDCl_3$) spectrum of **3dc** (di)

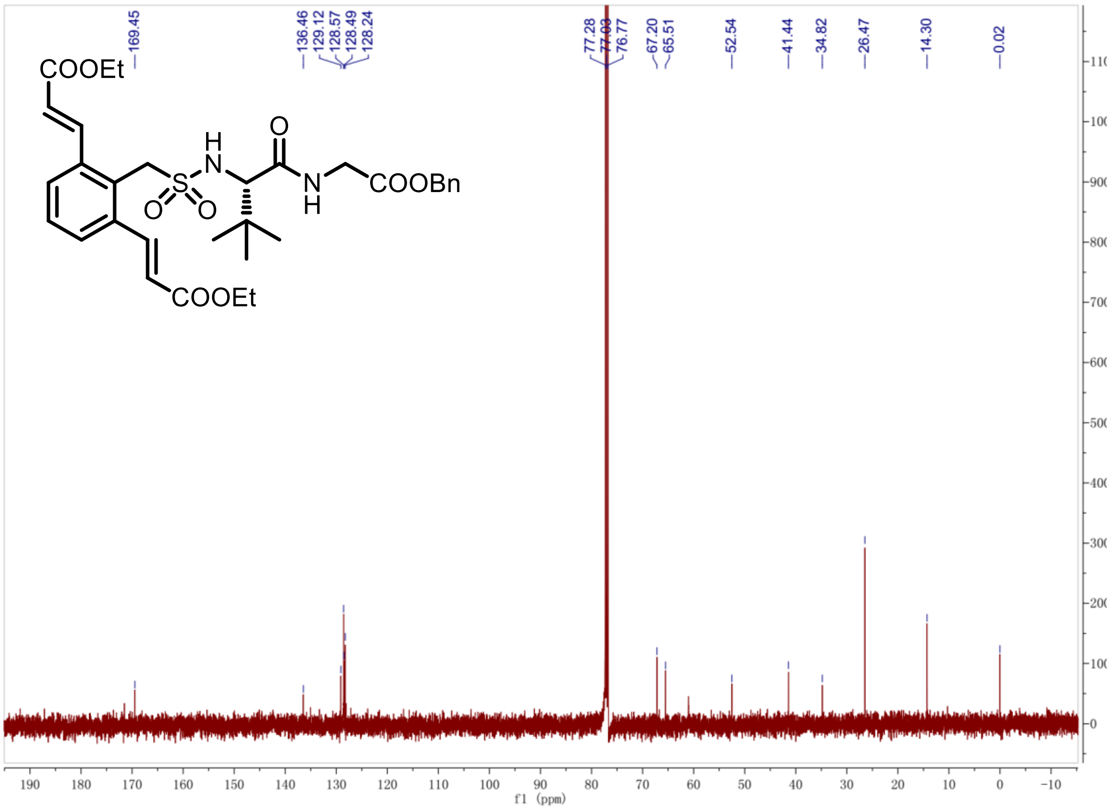

Supplementary Figure 68. ^{13}C NMR (125 MHz, $CDCl_3$) spectrum of **3dc** (di)

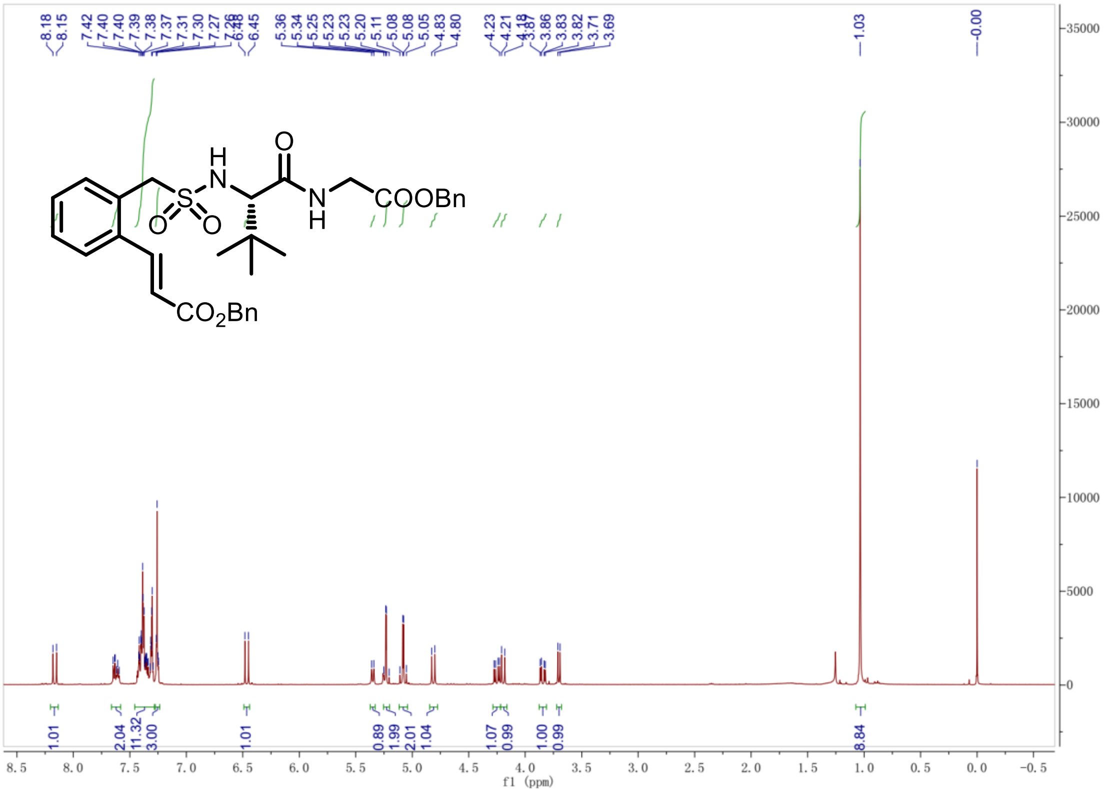

Supplementary Figure 69. ^{1}H NMR (500 MHz, $CDCl_3$) spectrum of **3dd** (mono)

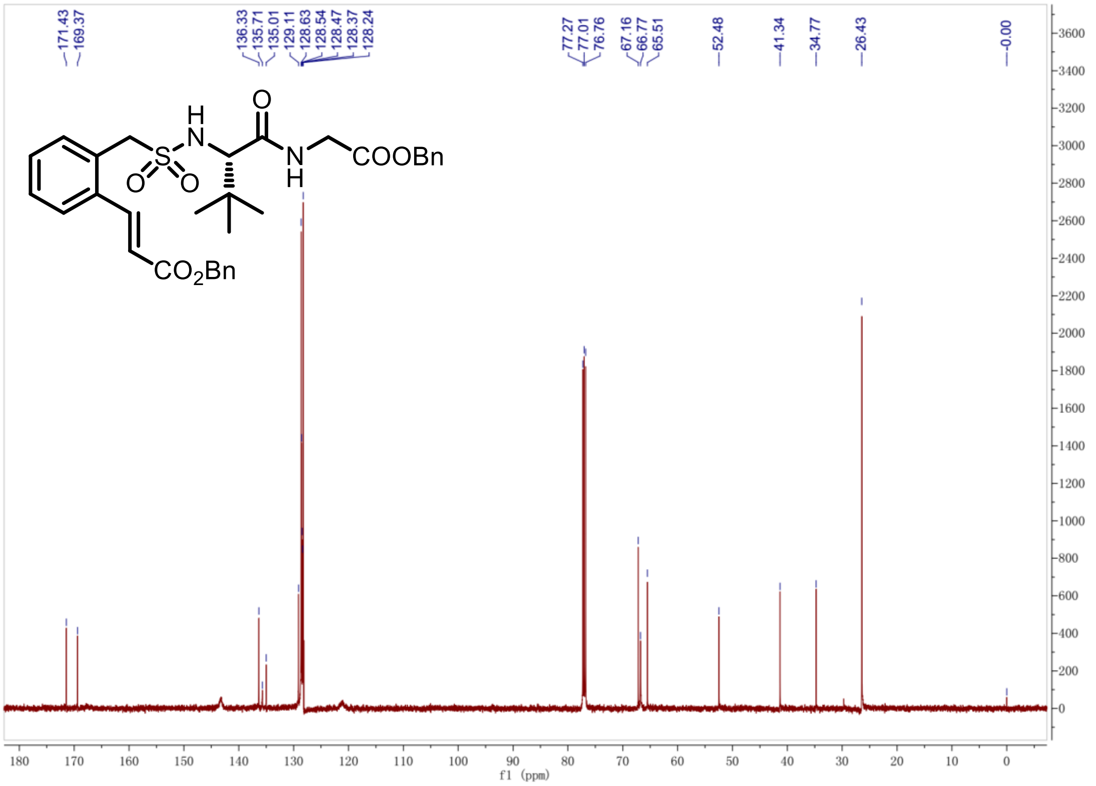

Supplementary Figure 70. ^{13}C NMR (125 MHz, $CDCl_3$) spectrum of **3dd** (mono)

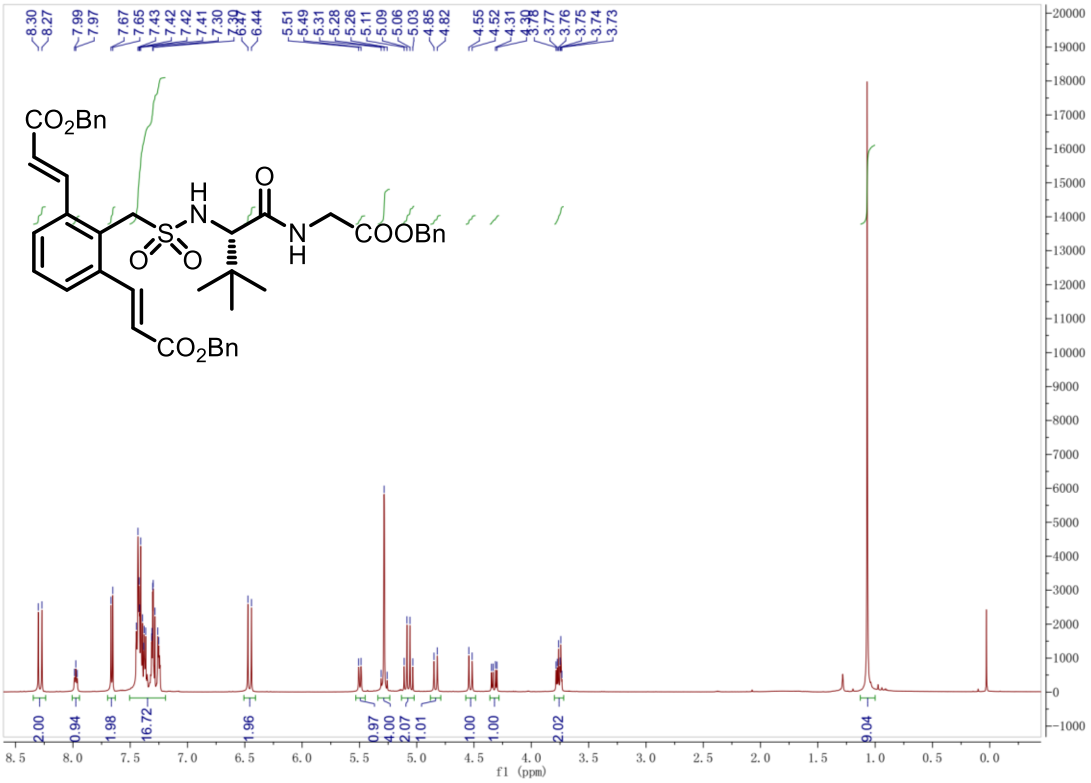

Supplementary Figure 71. ^{1}H NMR (500 MHz, $CDCl_3$) spectrum of **3dd** (di)

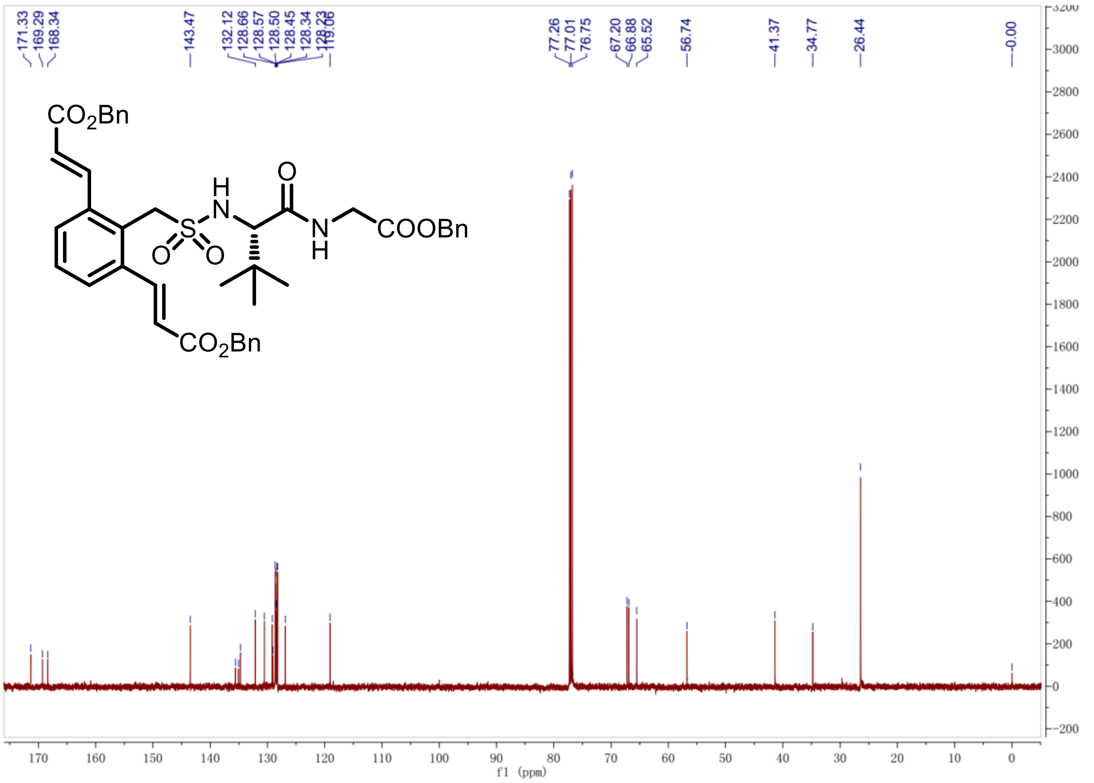

Supplementary Figure 72. ^{13}C NMR (125 MHz, $CDCl_3$) spectrum of **3dd** (di)

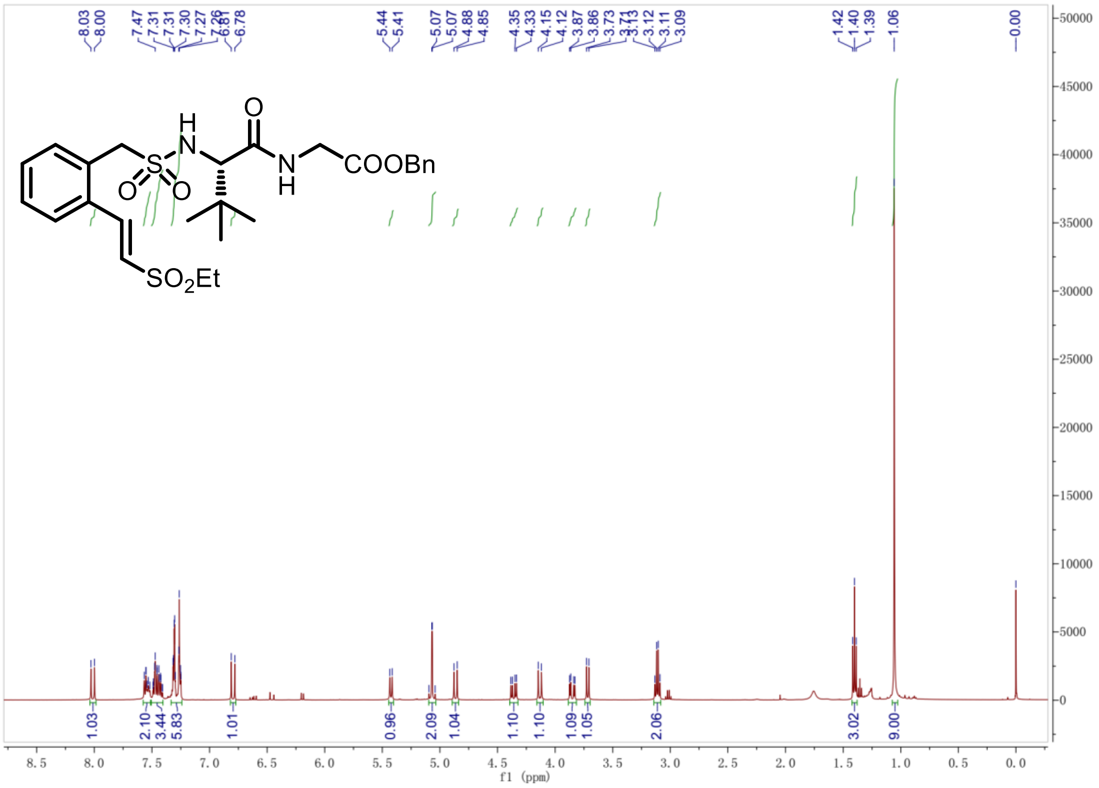

Supplementary Figure 73. ^{1}H NMR (500 MHz, $CDCl_3$) spectrum of **3de** (mono)

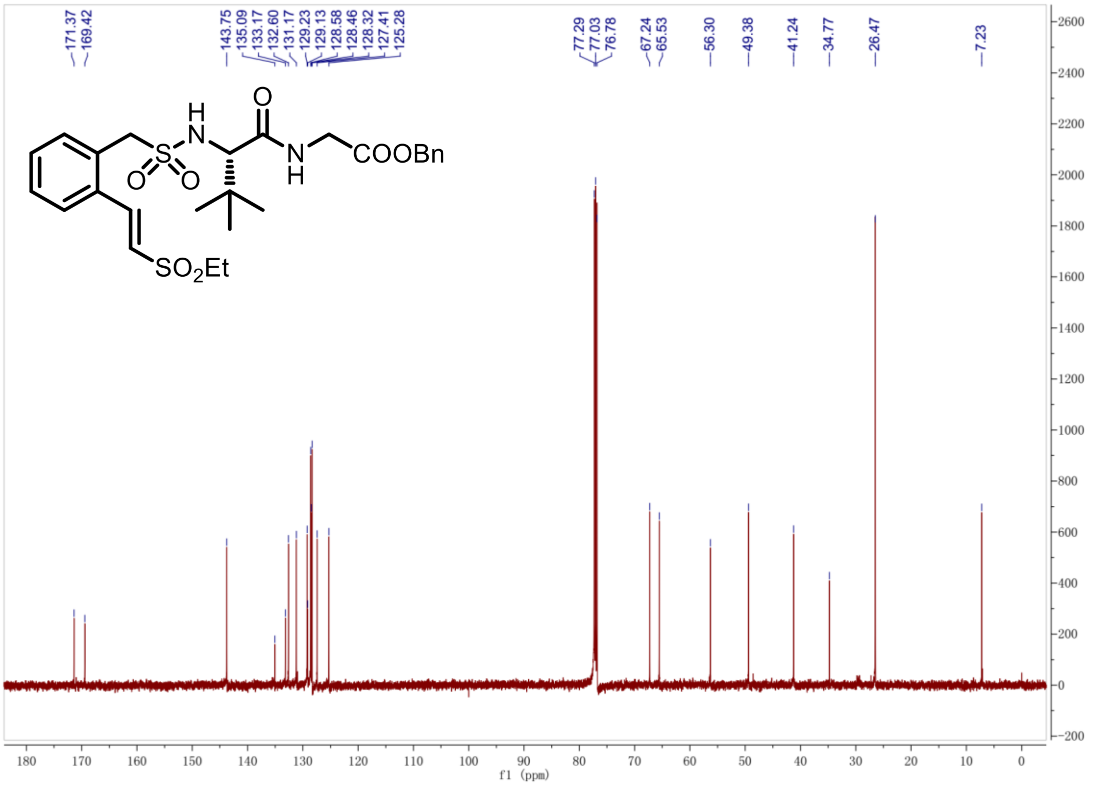

Supplementary Figure 74. ^{13}C NMR (125 MHz, $CDCl_3$) spectrum of **3de** (mono)

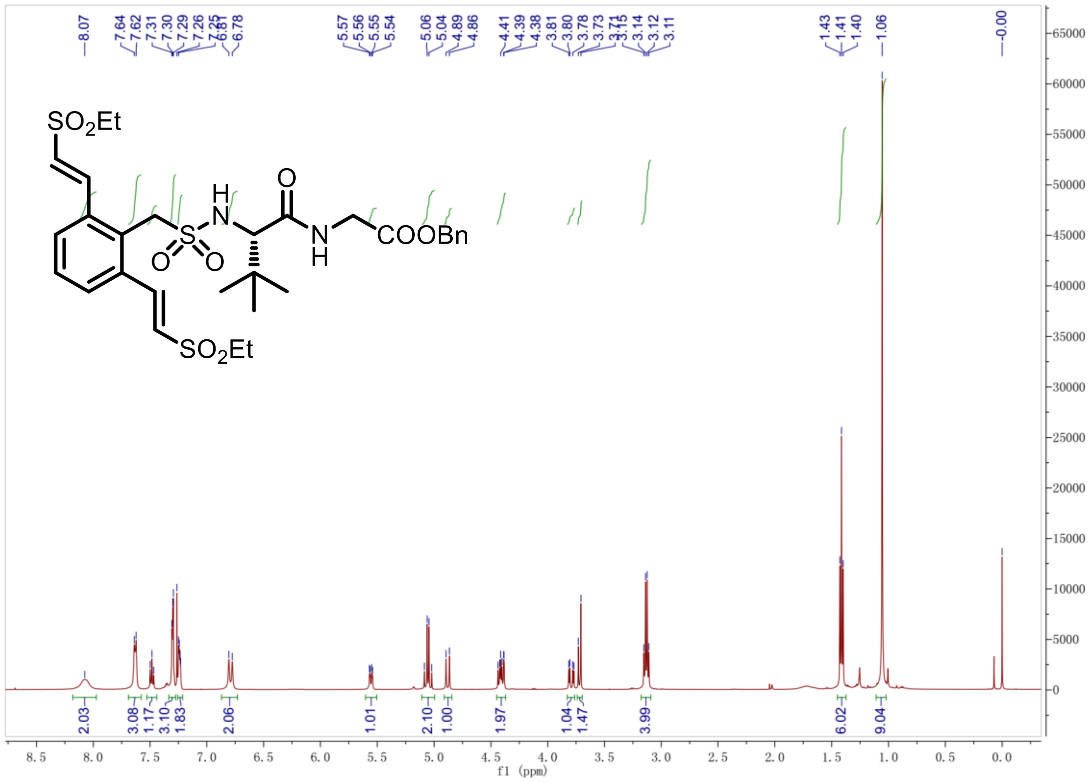

Supplementary Figure 75. ^{1}H NMR (500 MHz, $CDCl_3$) spectrum of **3de** (di)

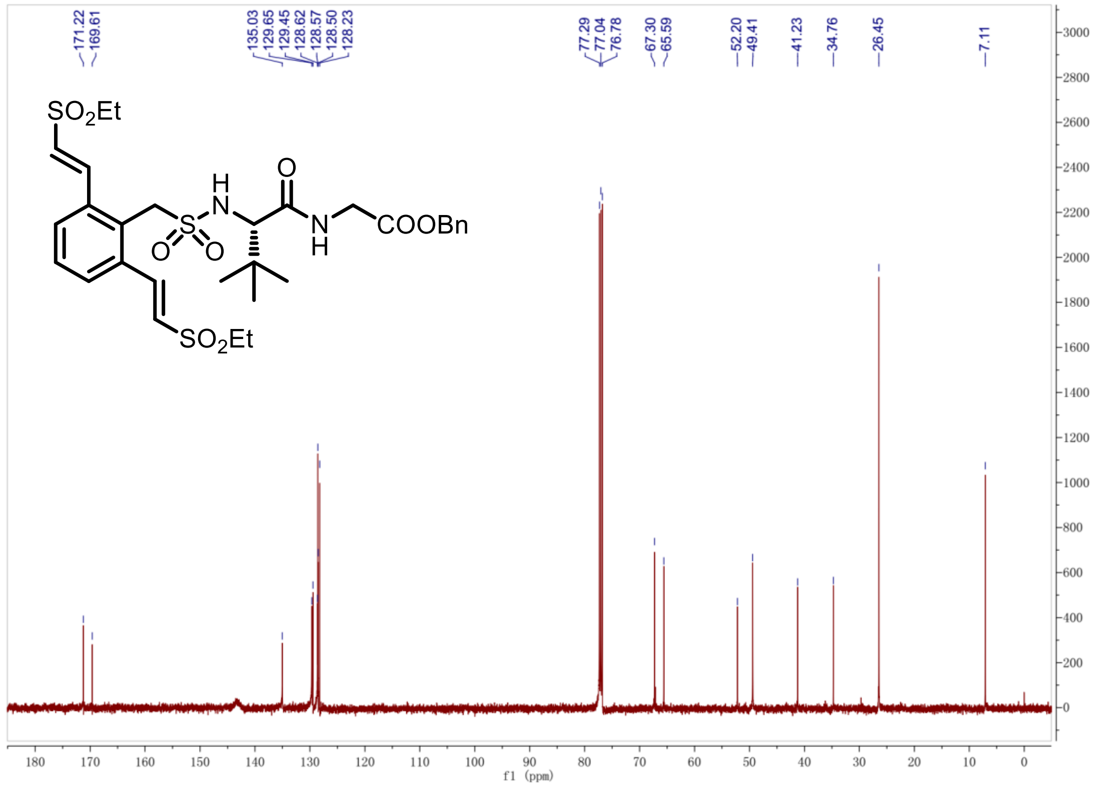

Supplementary Figure 76. ^{13}C NMR (125 MHz, $CDCl_3$) spectrum of **3de** (di)

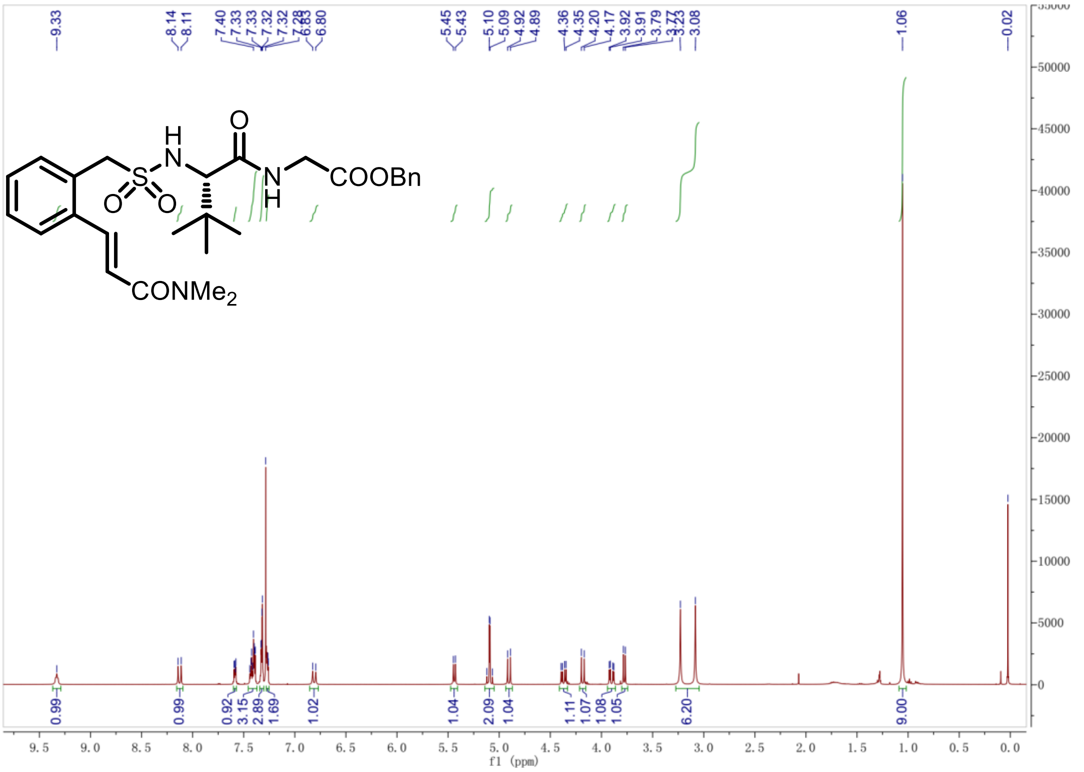

Supplementary Figure 77. ^{1}H NMR (500 MHz, $CDCl_3$) spectrum of **3df** (mono)

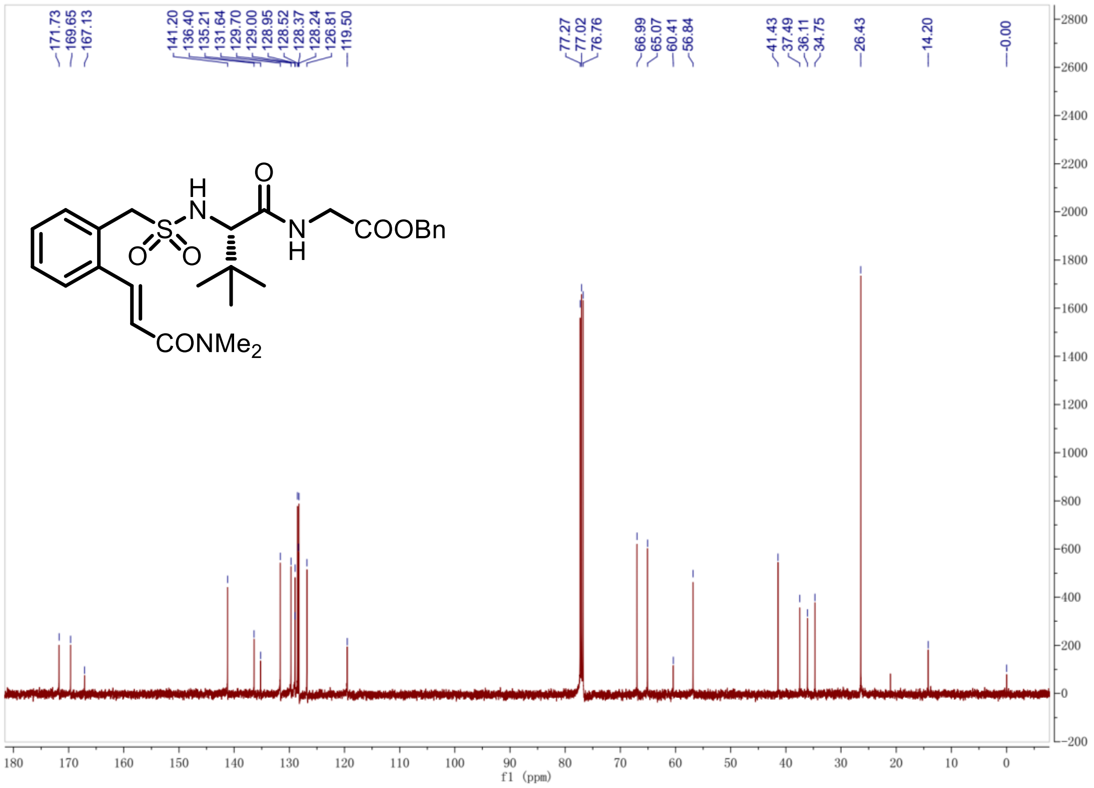

Supplementary Figure 78. ^{13}C NMR (125 MHz, $CDCl_3$) spectrum of **3df** (mono)

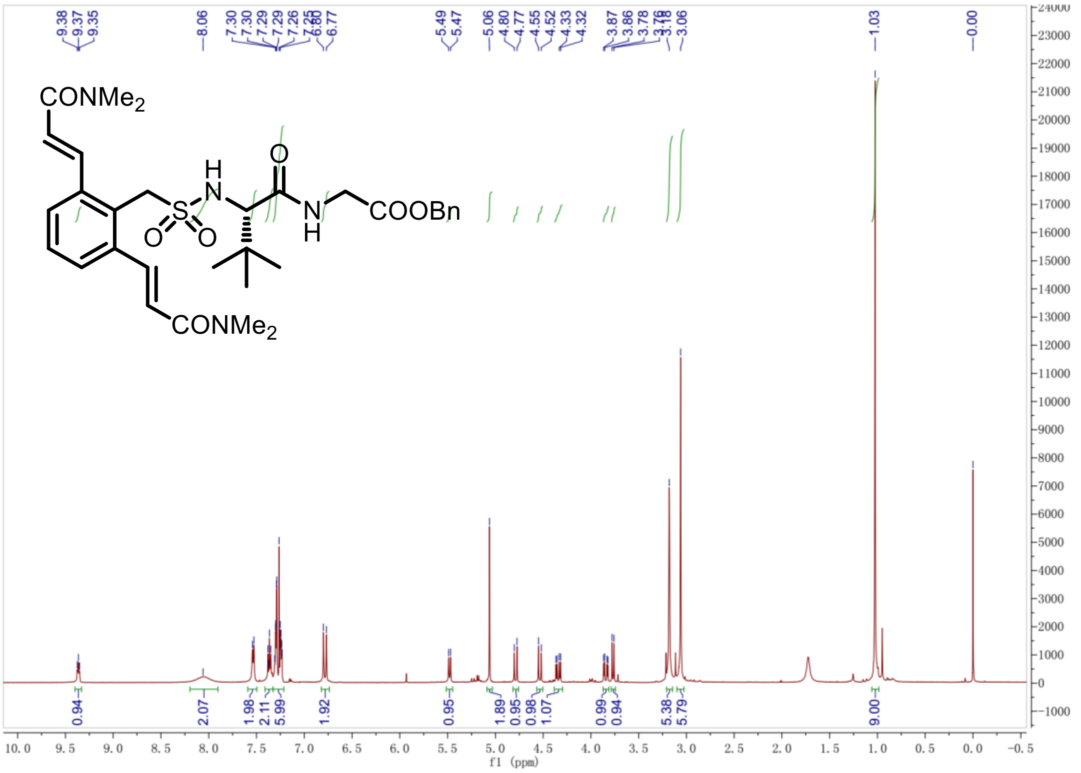

Supplementary Figure 79. ^{1}H NMR (500 MHz, $CDCl_3$) spectrum of **3df** (di)

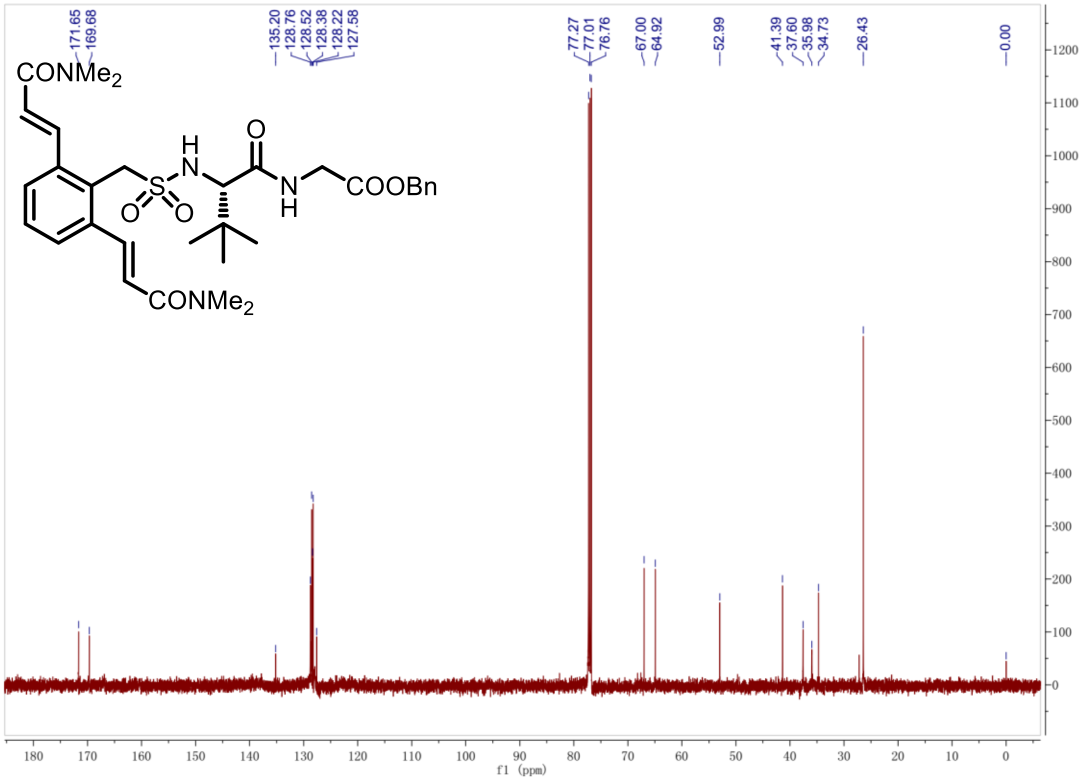

Supplementary Figure 80. ^{13}C NMR (125 MHz, $CDCl_3$) spectrum of **3df** (di)

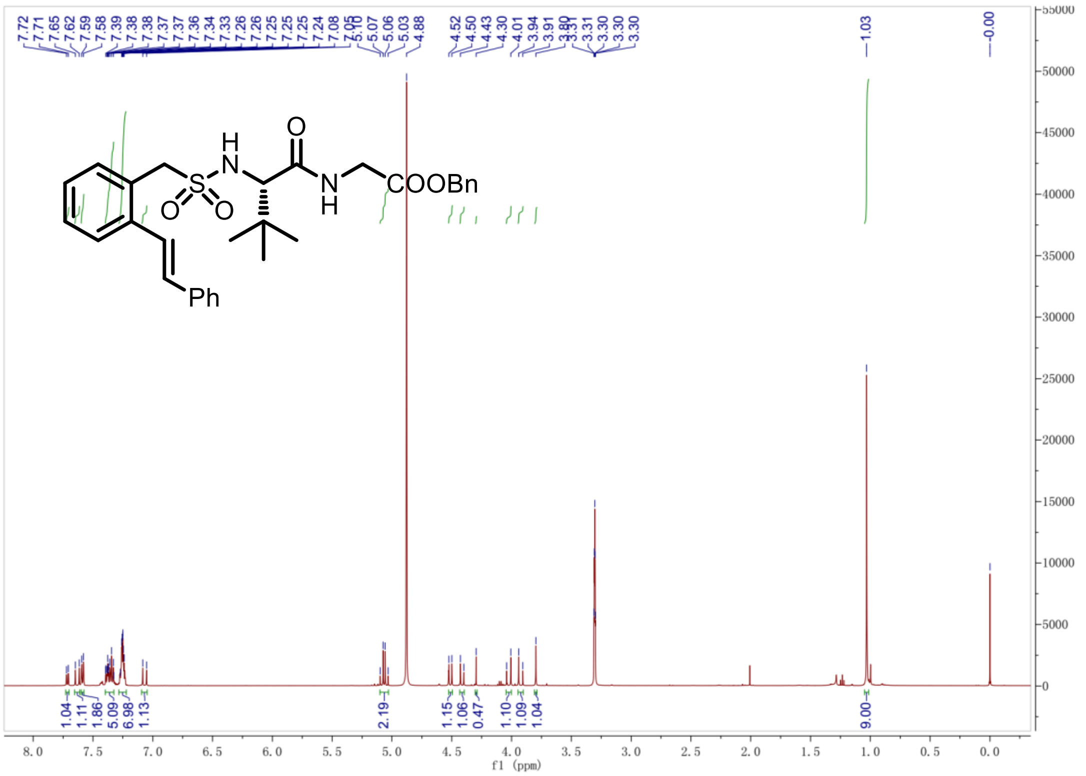

Supplementary Figure 81. ^{1}H NMR (500 MHz, $CDCl_3$) spectrum of **3dg** (mono)

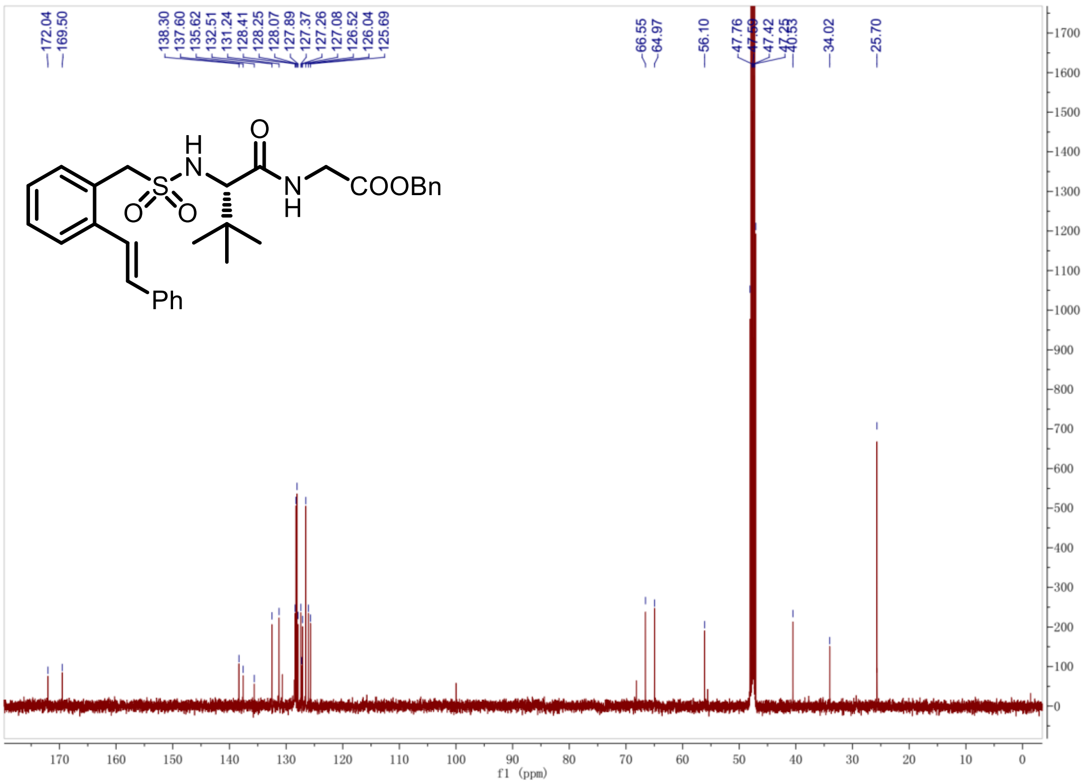

Supplementary Figure 82. ^{13}C NMR (125 MHz, $CDCl_3$) spectrum of **3dg** (mono)

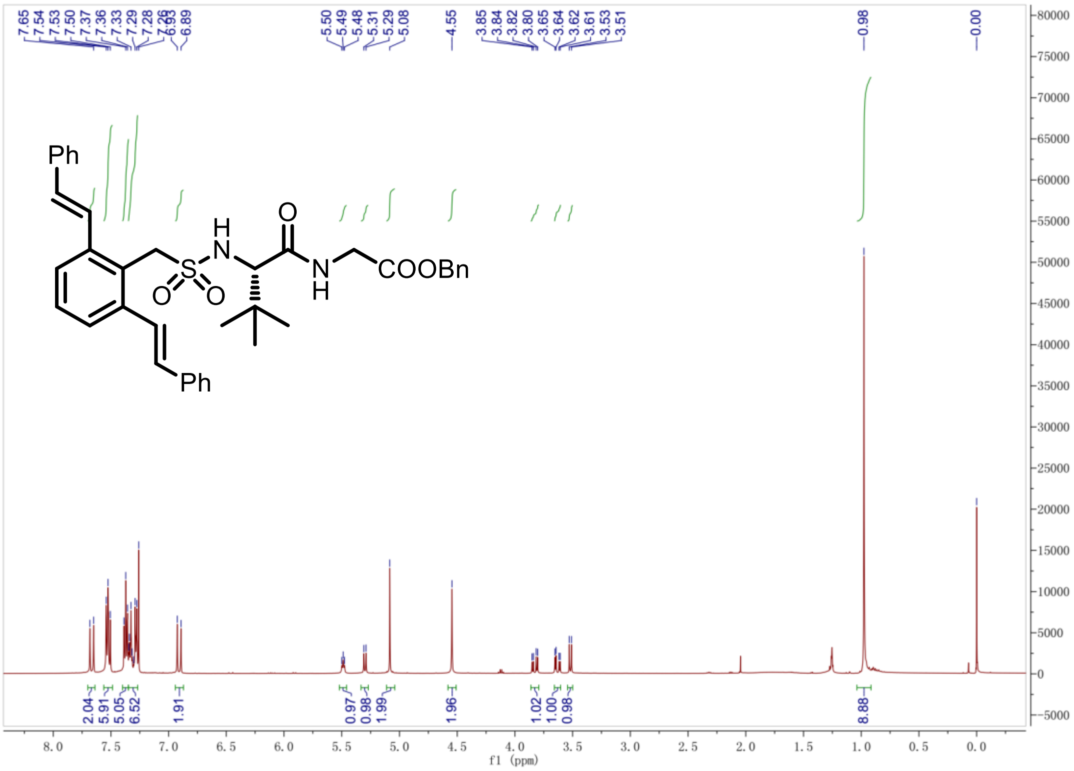

Supplementary Figure 83. ^{1}H NMR (500 MHz, $CDCl_3$) spectrum of **3dg** (di)

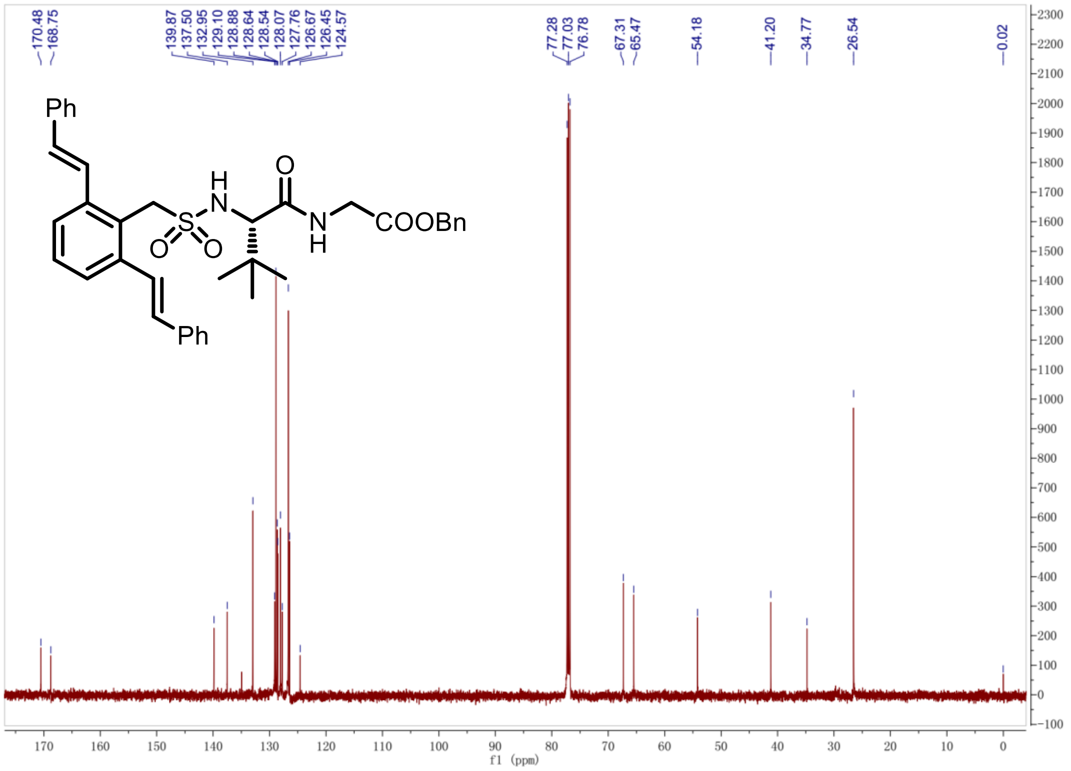

Supplementary Figure 84. ^{13}C NMR (125MHz, $CDCl_3$) spectrum of **3dg** (di)

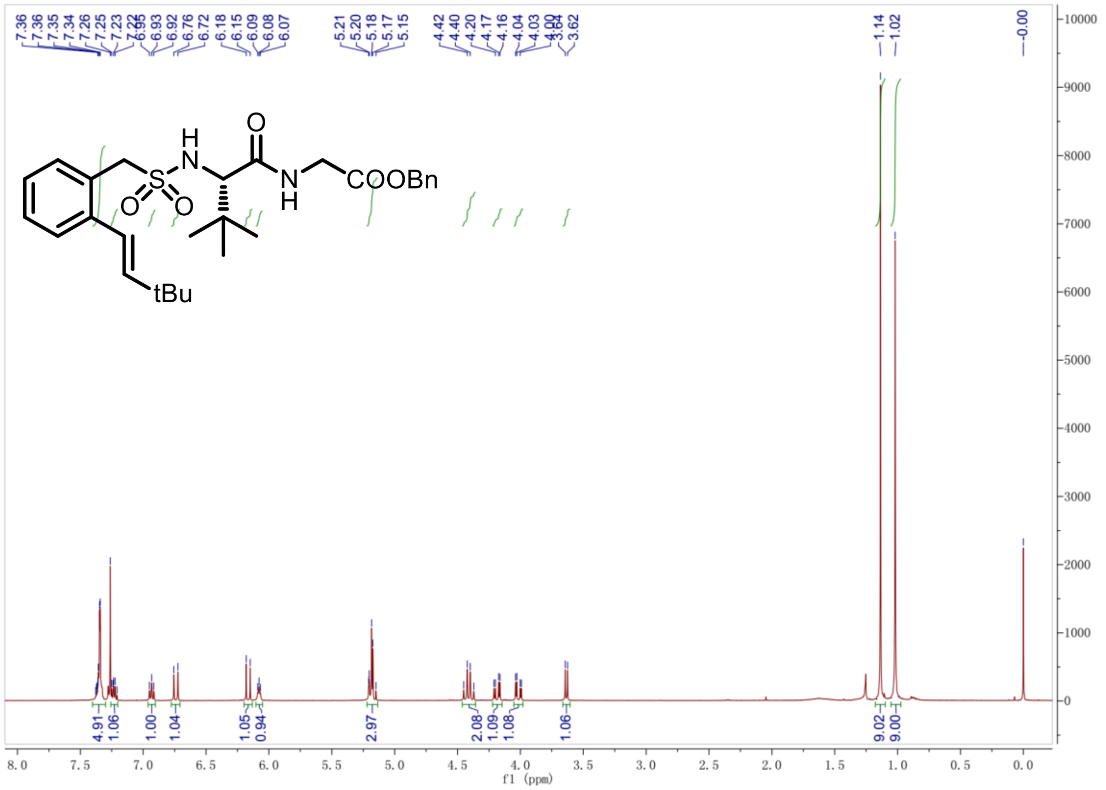

Supplementary Figure 85. ^{1}H NMR (500 MHz, $CDCl_3$) spectrum of **3dh** (mono)

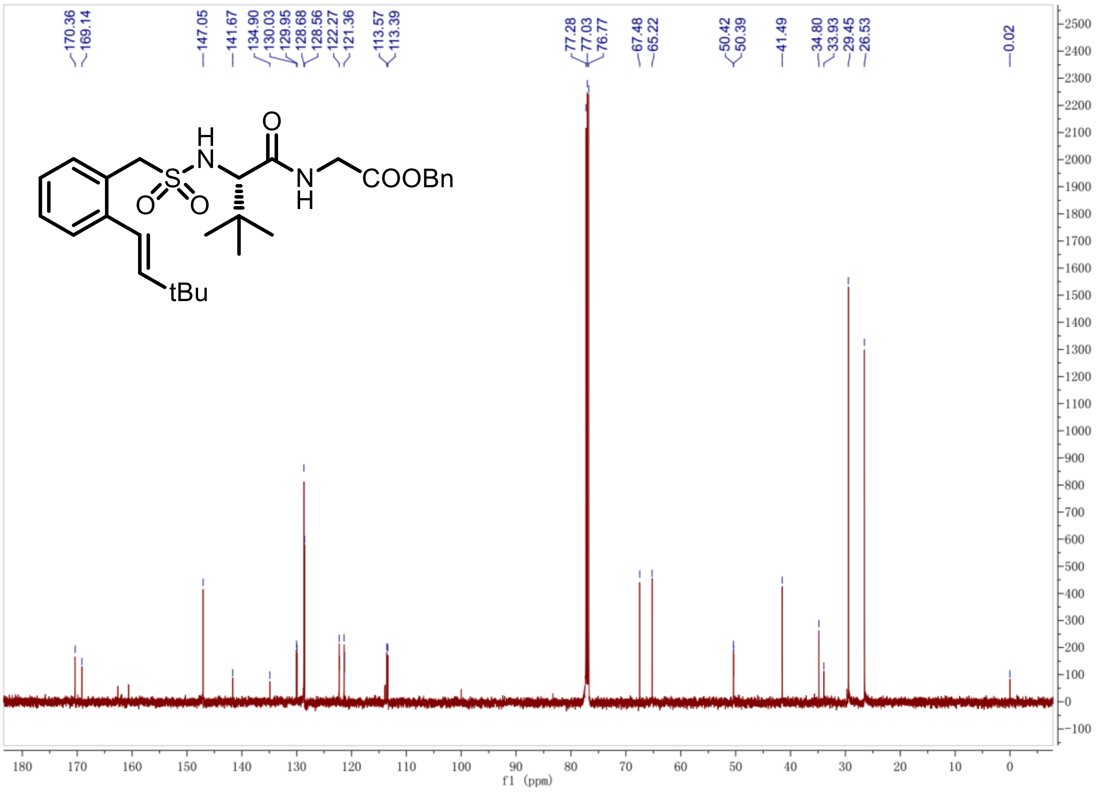

Supplementary Figure 86. ^{13}C NMR (125 MHz, $CDCl_3$) spectrum of **3dh** (mono)

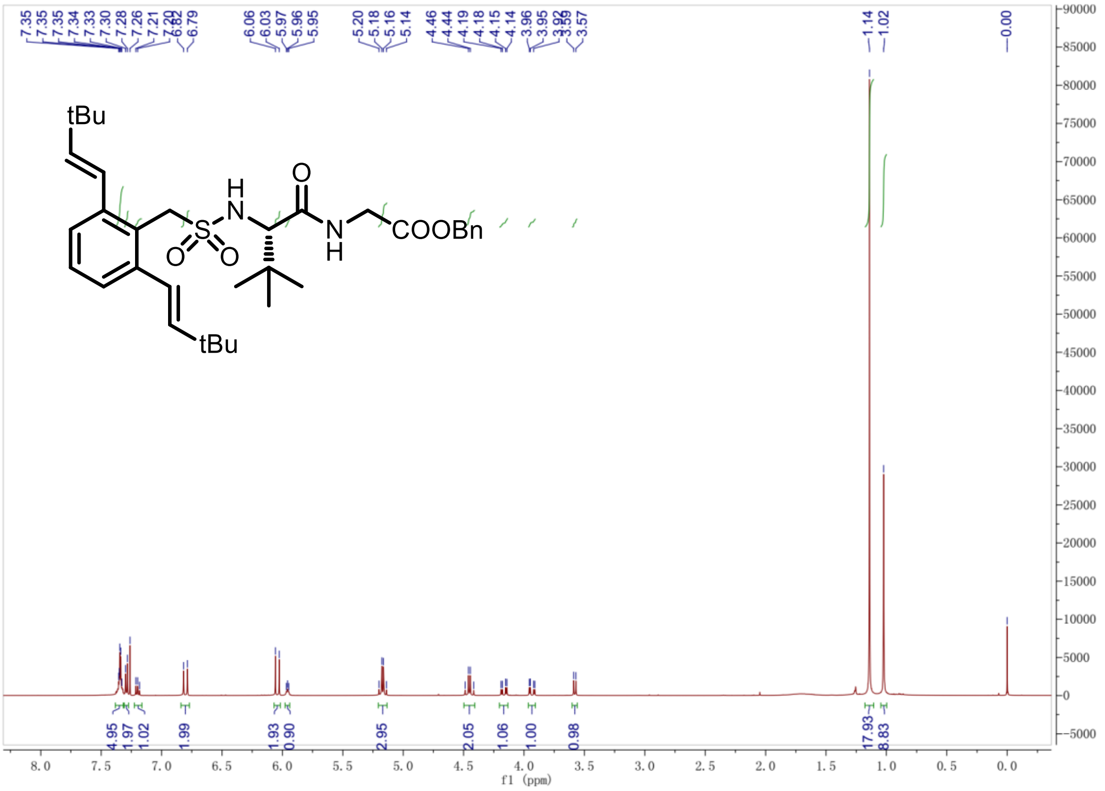

Supplementary Figure 87. ^{1}H NMR (500 MHz, $CDCl_3$) spectrum of **3dh** (di)

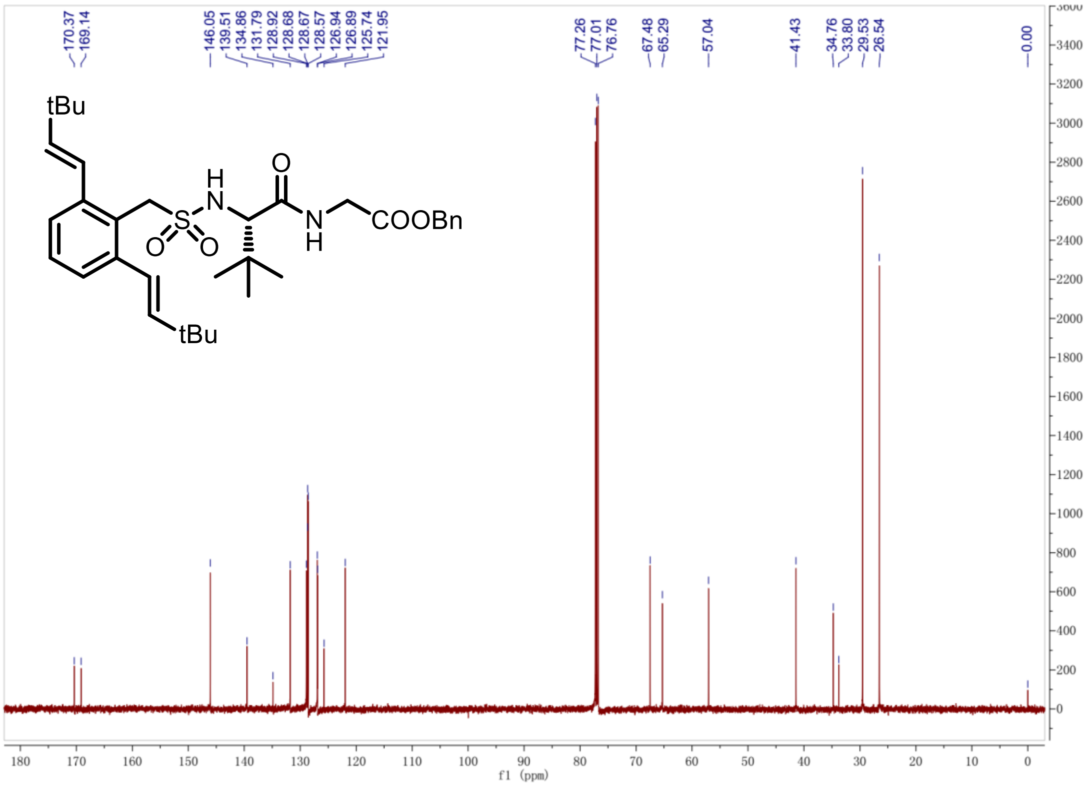

Supplementary Figure 88. ^{13}C NMR (125 MHz, $CDCl_3$) spectrum of **3dh** (di)

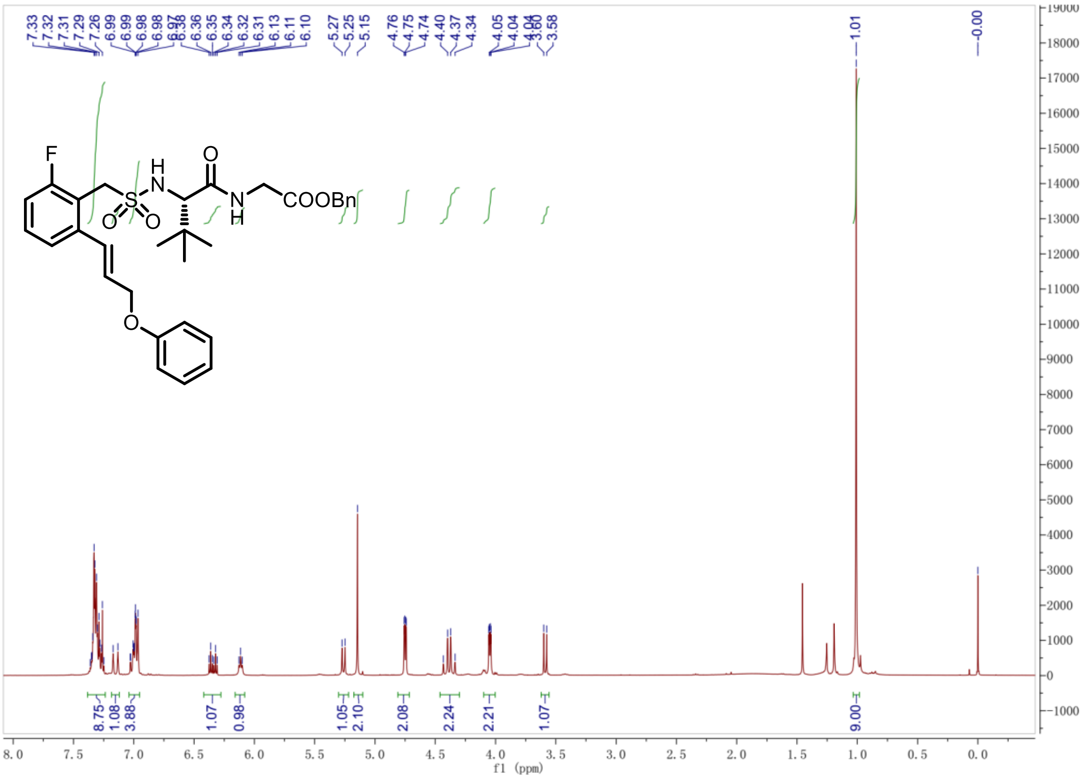

Supplementary Figure 89. ^{1}H NMR (400 MHz, $CDCl_3$) spectrum of compound **3di**

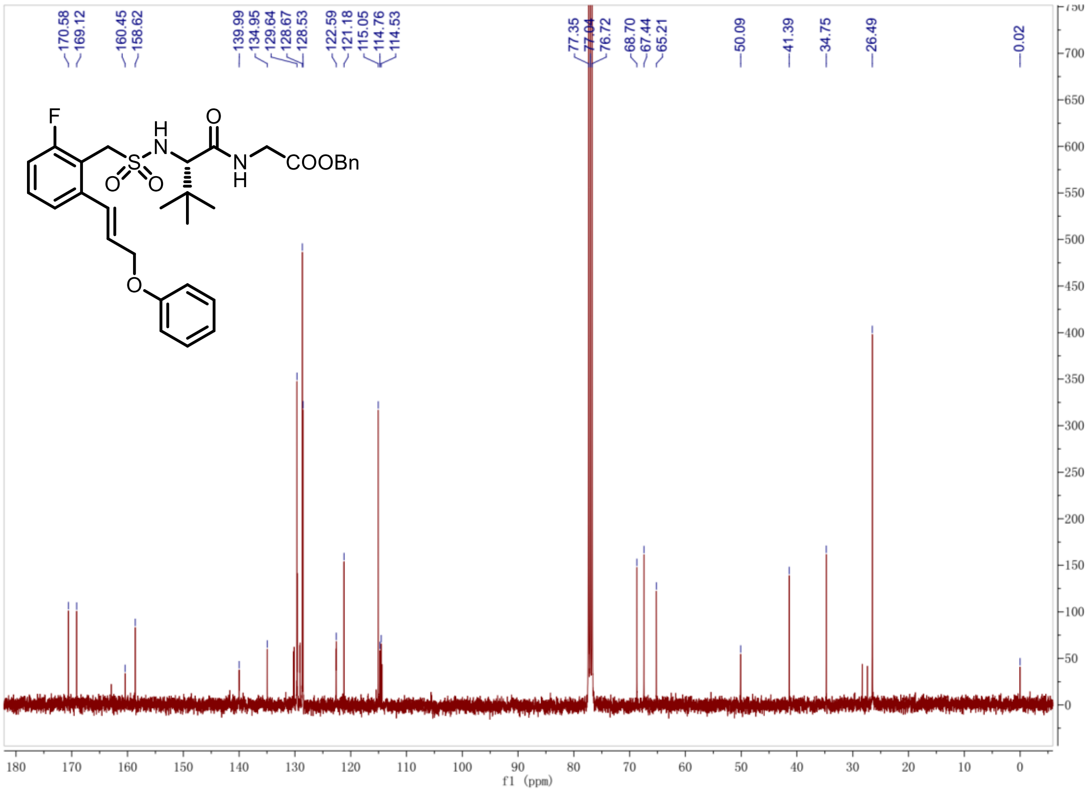

Supplementary Figure 90. ^{13}C NMR (100 MHz, $CDCl_3$) spectrum of compound **3di**

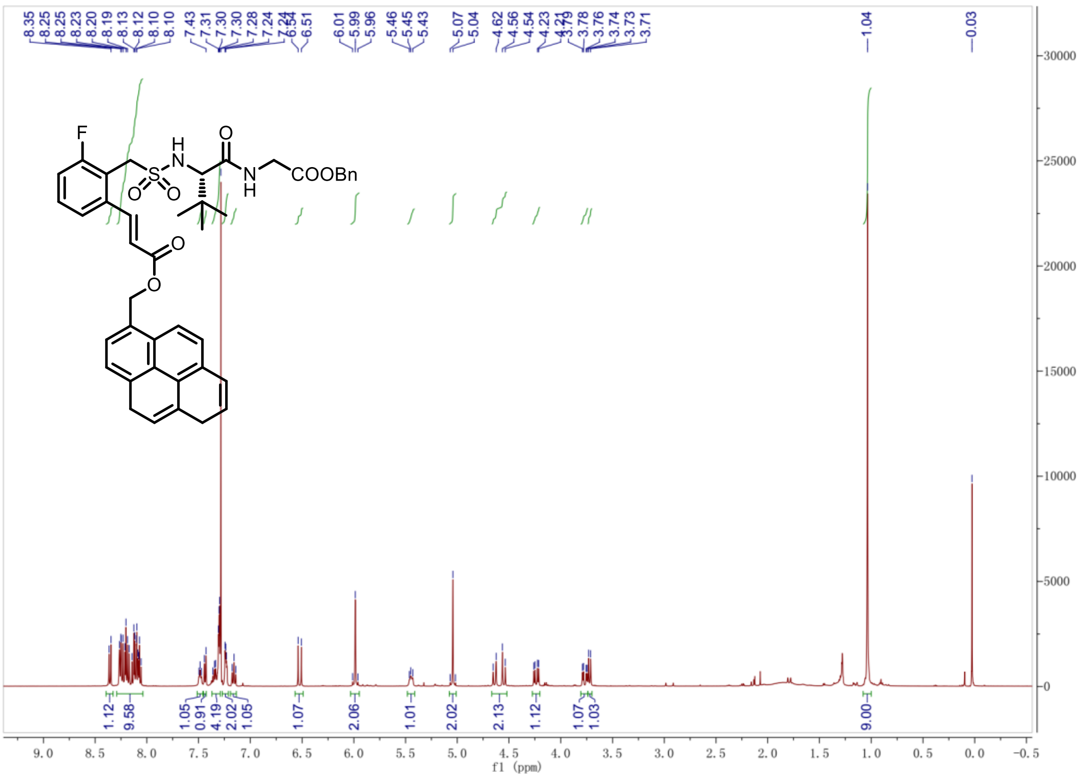

Supplementary Figure 91. ^{1}H NMR (500 MHz, $CDCl_3$) spectrum of compound **3dj**

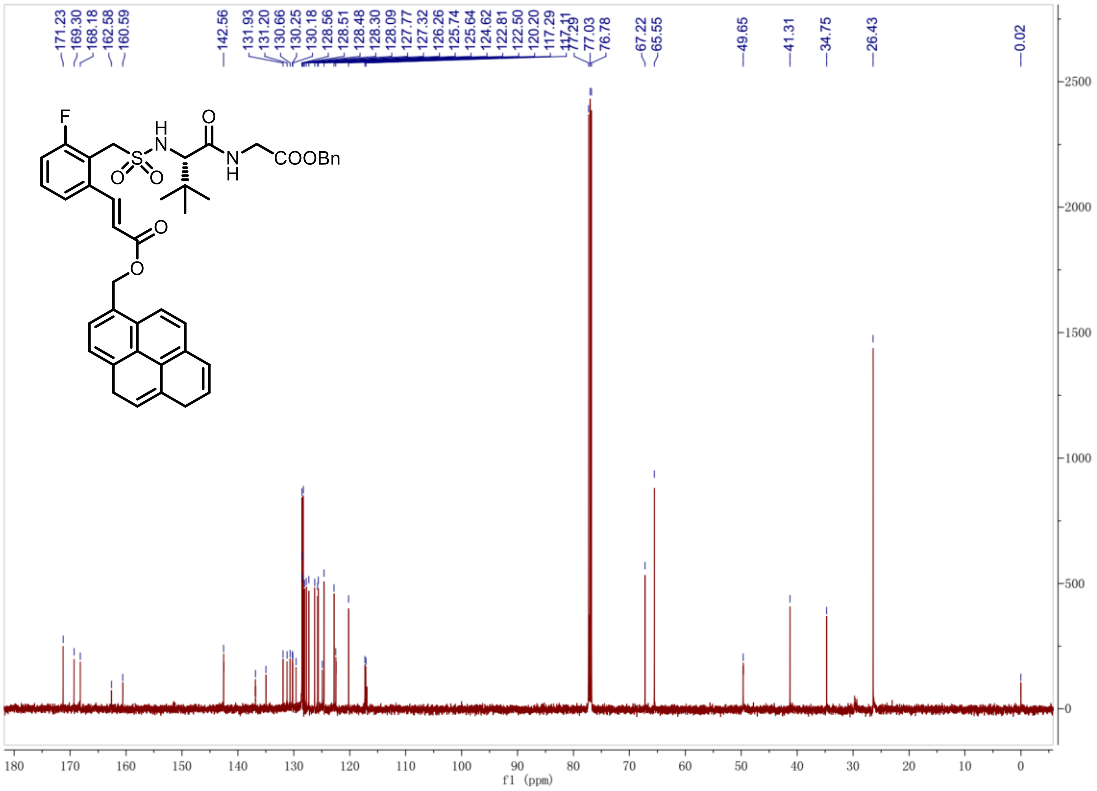

Supplementary Figure 92. ^{13}C NMR (125MHz, $CDCl_3$) spectrum of compound **3dj**

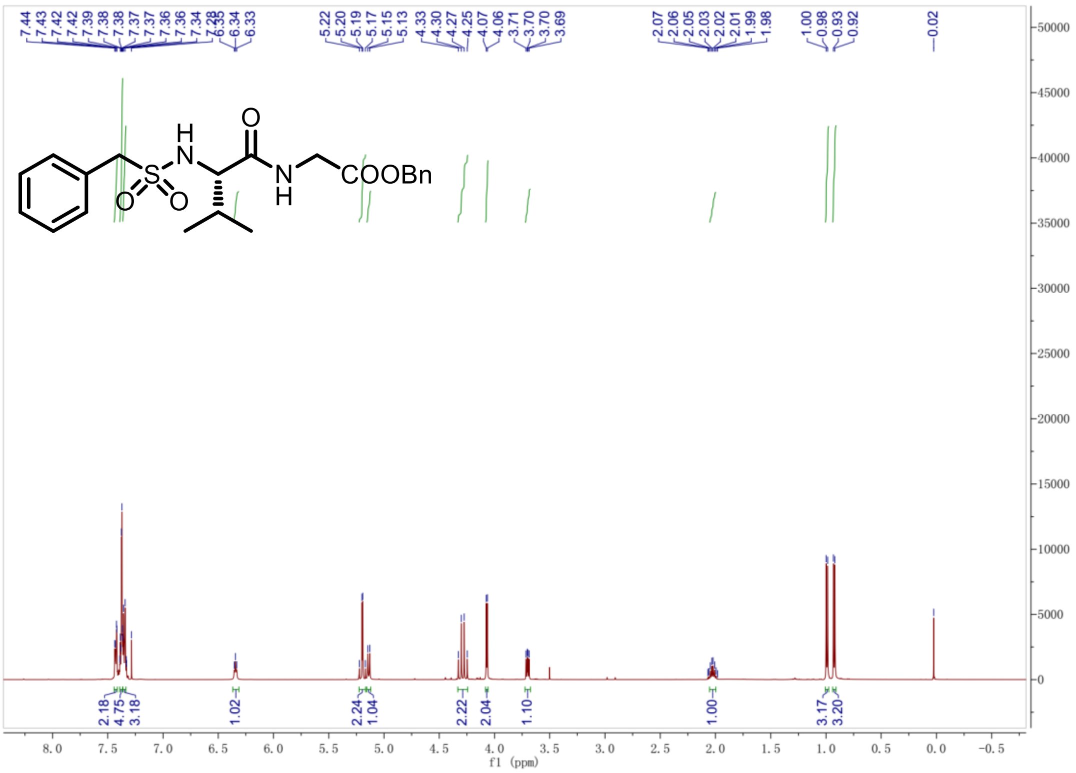

Supplementary Figure 93. ^{1}H NMR (400 MHz, $CDCl_3$) spectrum of compound **1k**

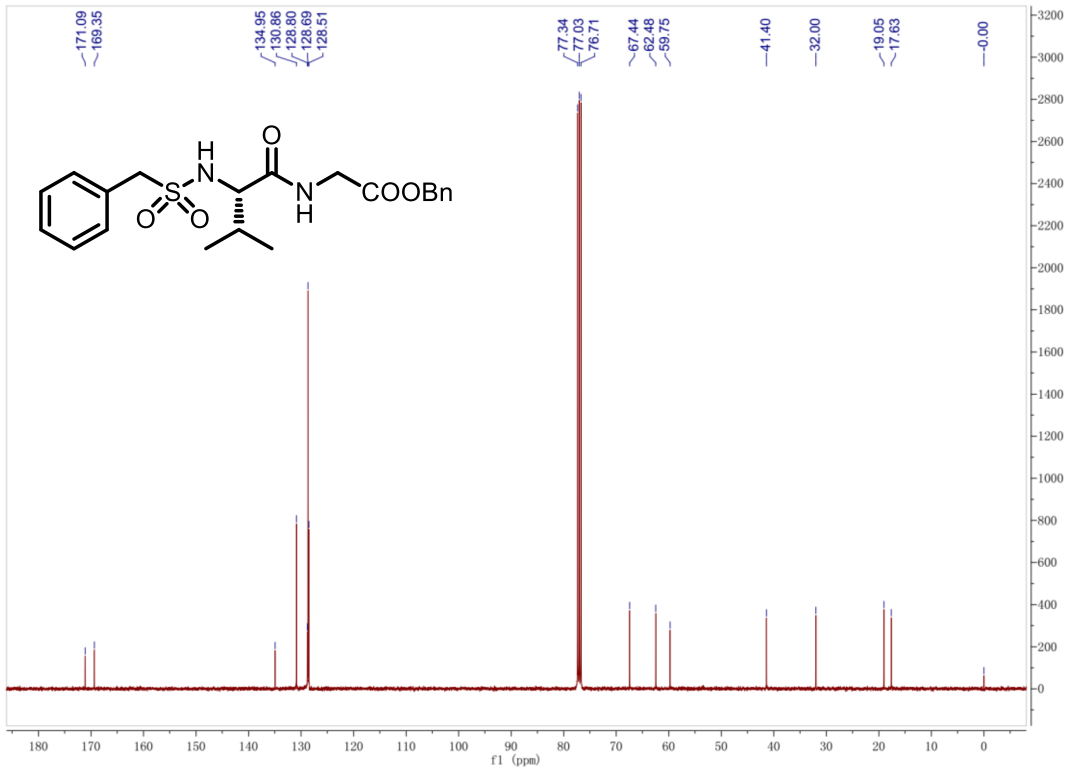

Supplementary Figure 94. ^{13}C NMR (100 MHz, $CDCl_3$) spectrum of compound **1k**

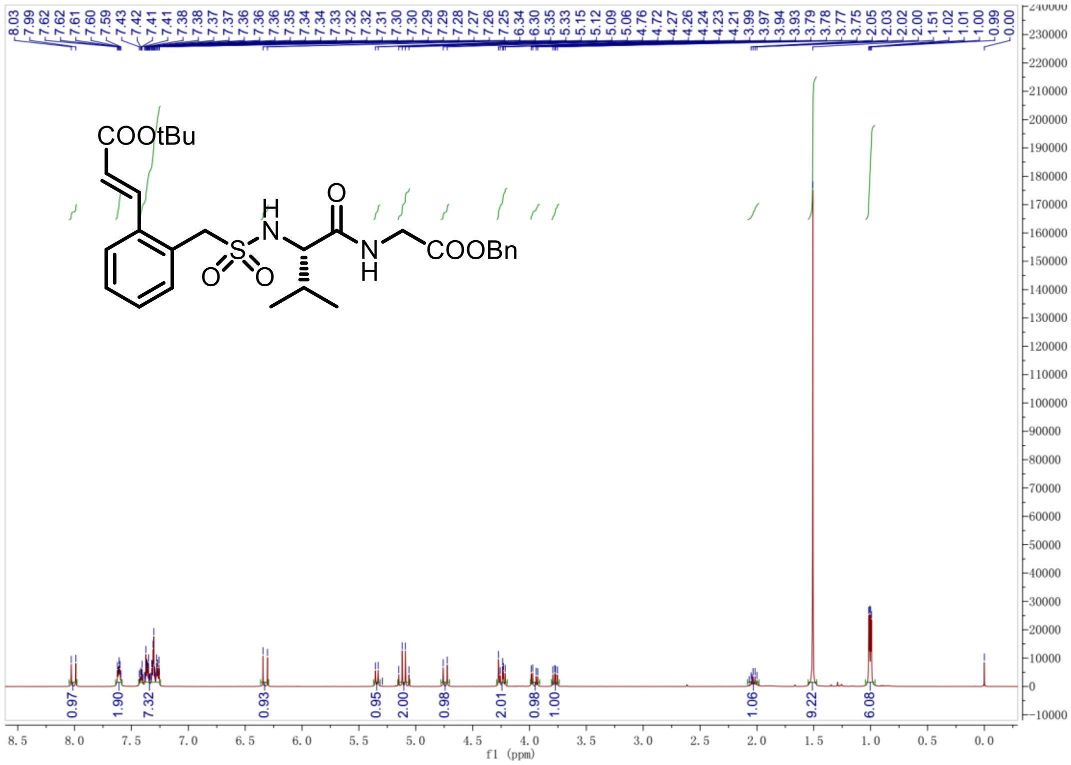

Supplementary Figure 95. ^{1}H NMR (400 MHz, $CDCl_3$) spectrum of **3ka** (mono)

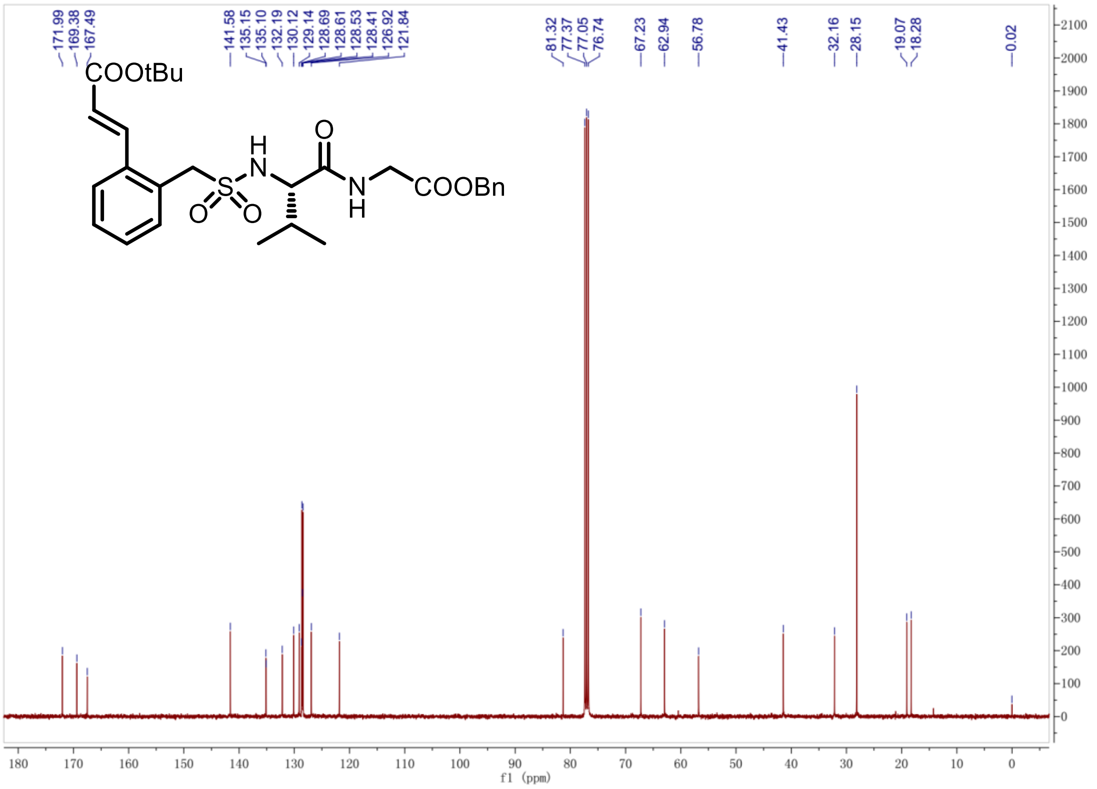

Supplementary Figure 96. ^{13}C NMR (100 MHz, $CDCl_3$) spectrum of **3ka** (mono)

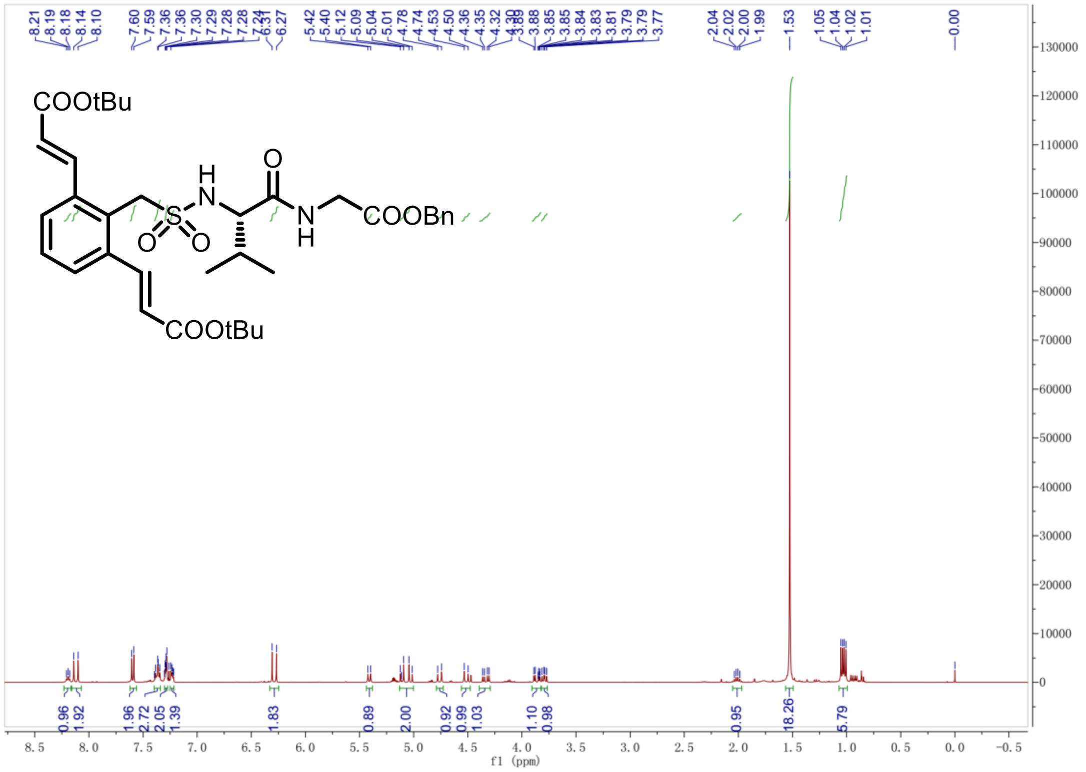

Supplementary Figure 97. ^{1}H NMR (400 MHz, $CDCl_3$) spectrum of **3ka** (di)

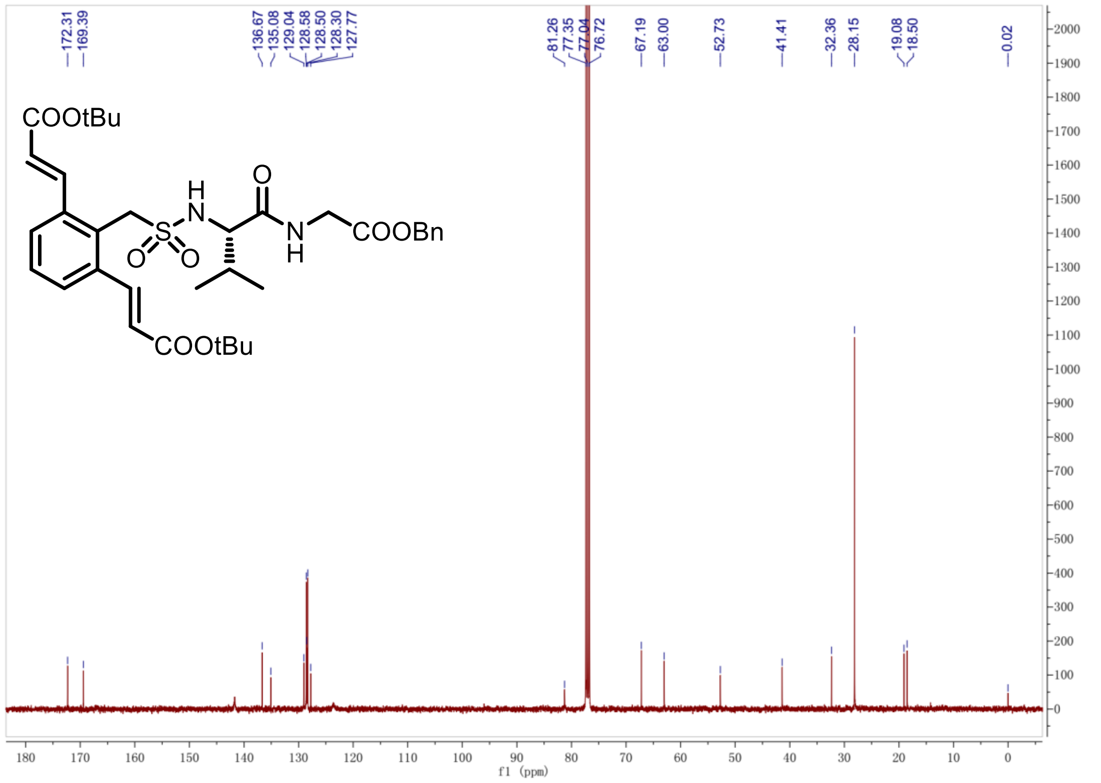

Supplementary Figure 98. ^{13}C NMR (100 MHz, $CDCl_3$) spectrum of **3ka** (di)

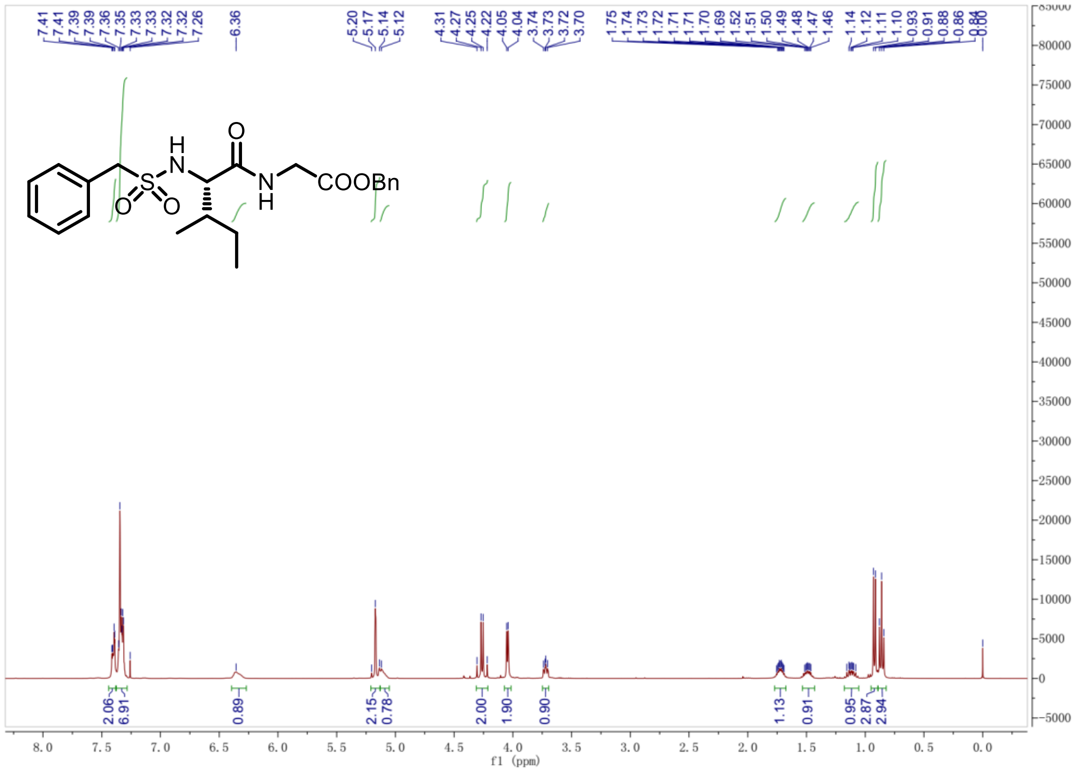

Supplementary Figure 99. ^{1}H NMR (400 MHz, $CDCl_3$) spectrum of compound **1l**

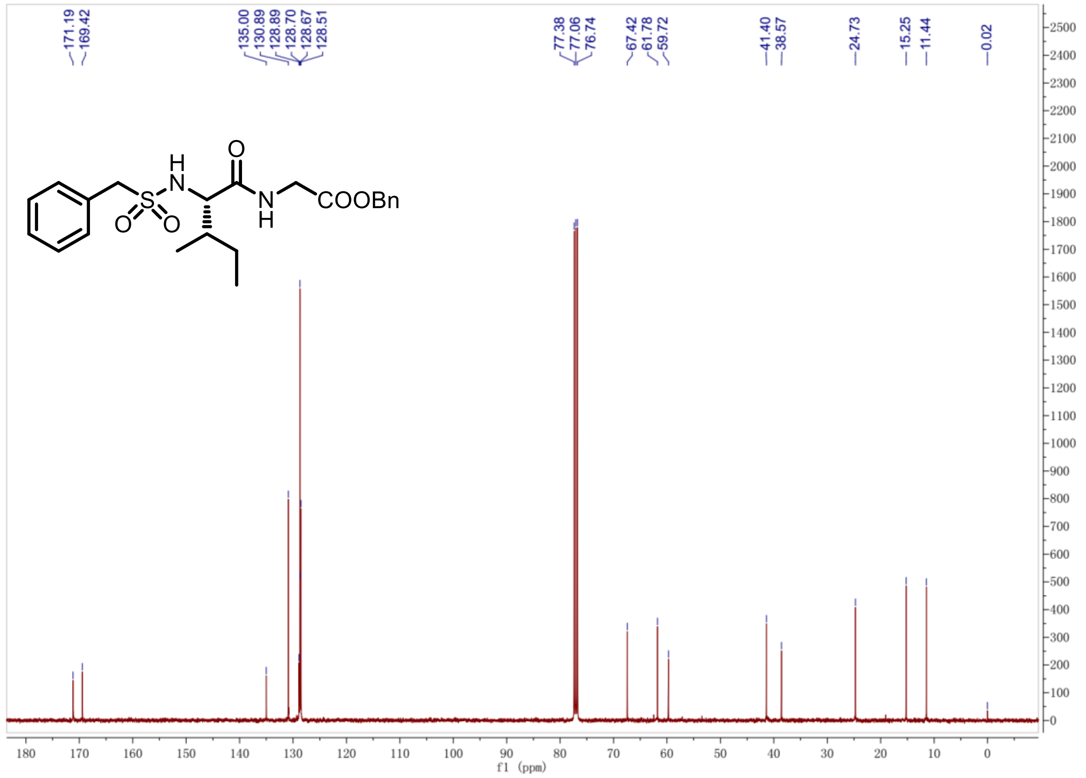

Supplementary Figure 100. ^{13}C NMR (100 MHz, $CDCl_3$) spectrum of compound **1l**

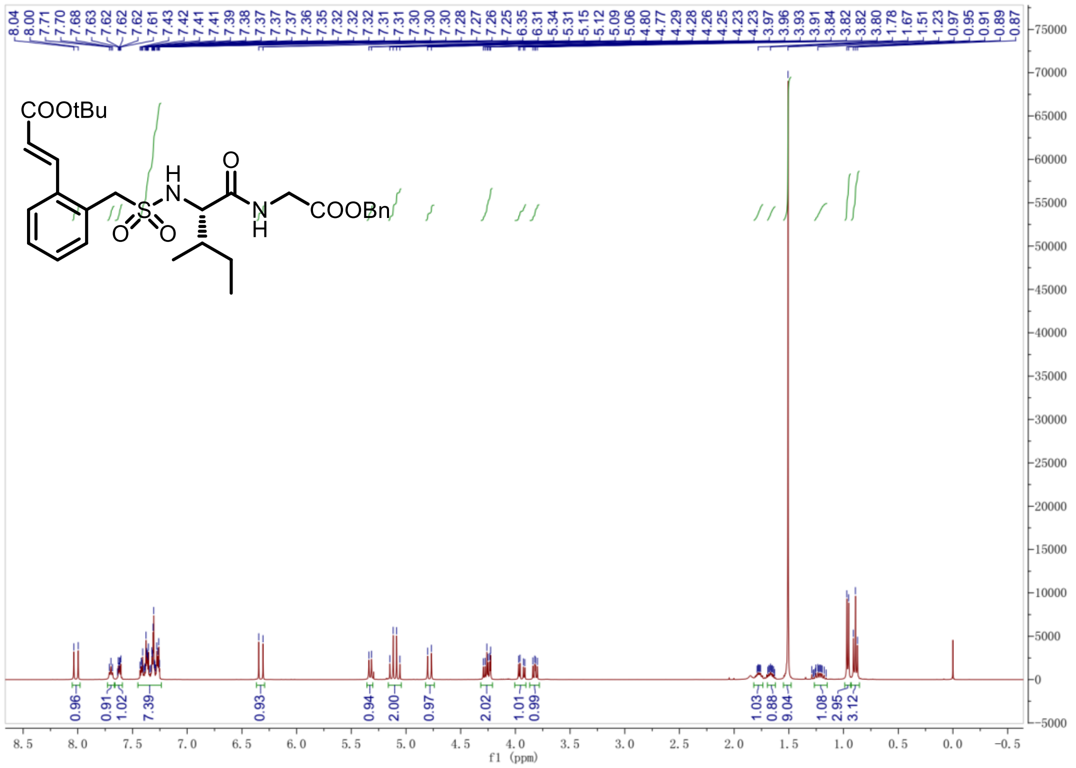

Supplementary Figure 101. ^{1}H NMR (400 MHz, $CDCl_3$) spectrum of **3la** (mono)

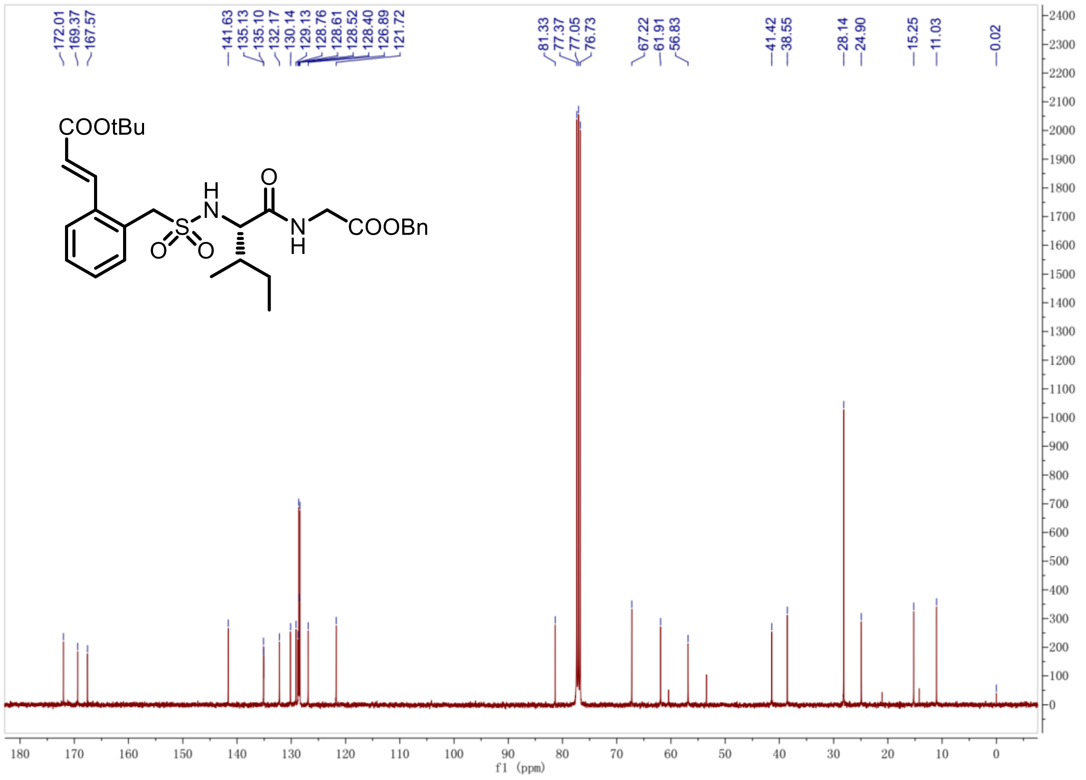

Supplementary Figure 102. ^{13}C NMR (100 MHz, $CDCl_3$) spectrum of **3la** (mono)

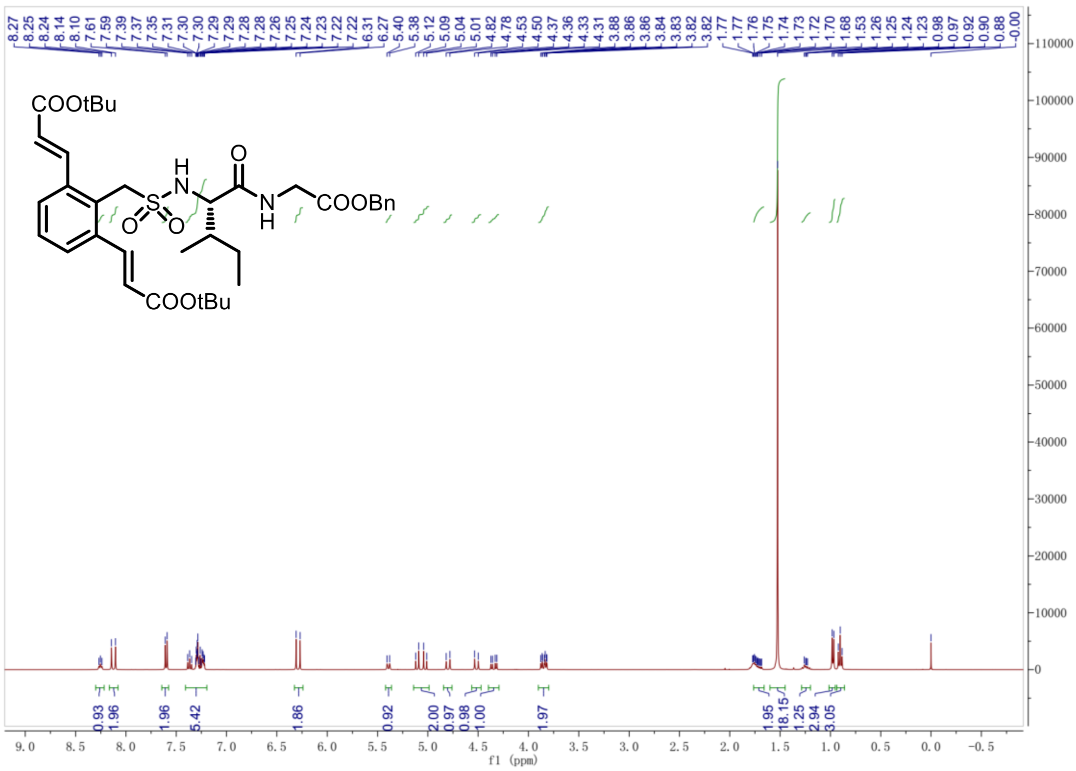

Supplementary Figure 103. ^{1}H NMR (400 MHz, $CDCl_3$) spectrum of **3la** (di)

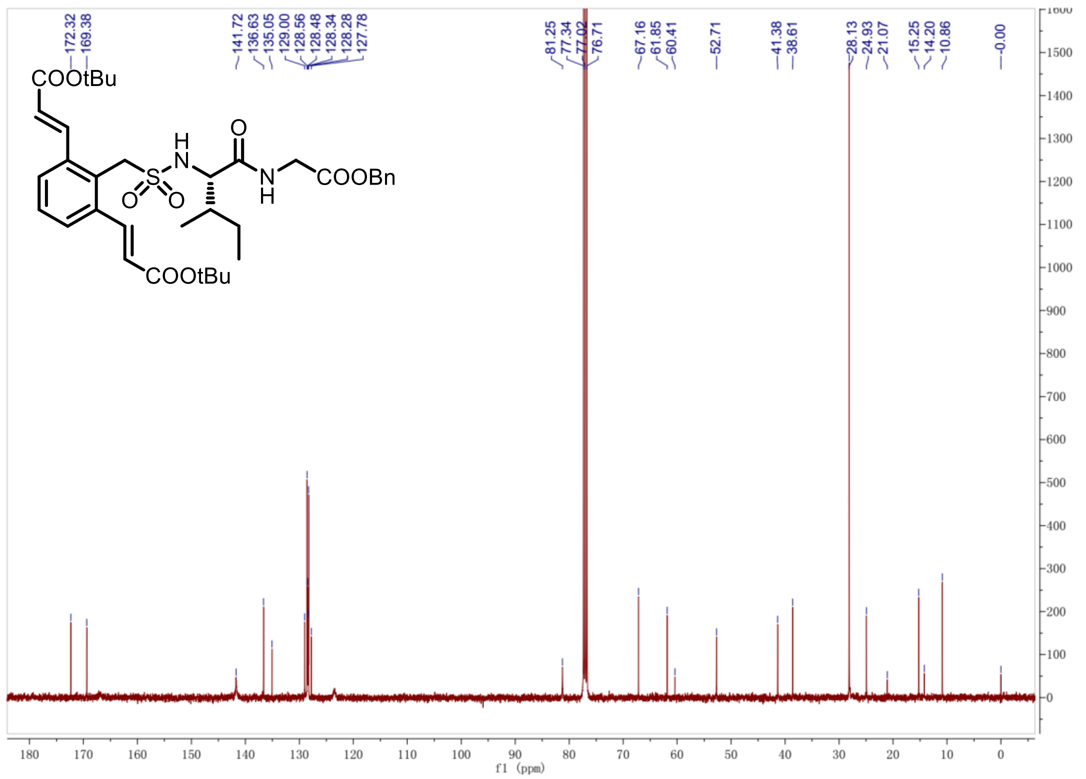

Supplementary Figure 104. ^{13}C NMR (100 MHz, $CDCl_3$) spectrum of **3la** (di)

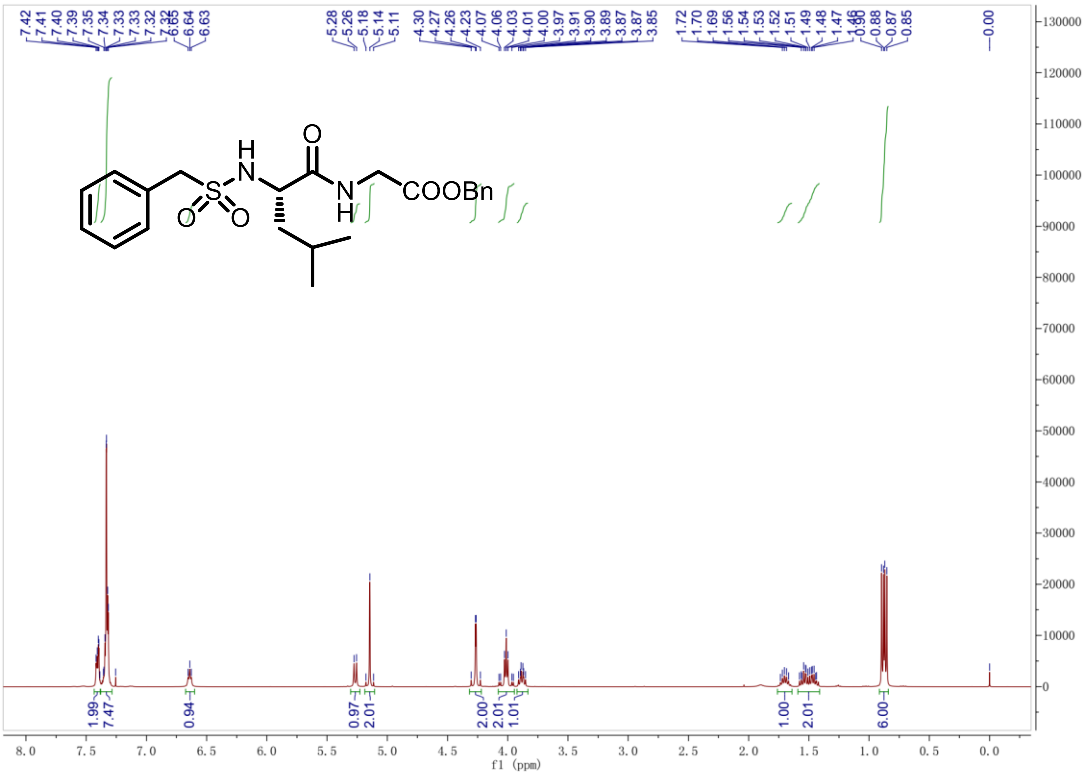

Supplementary Figure 105. ^{1}H NMR (400 MHz, $CDCl_3$) spectrum of compound **1m**

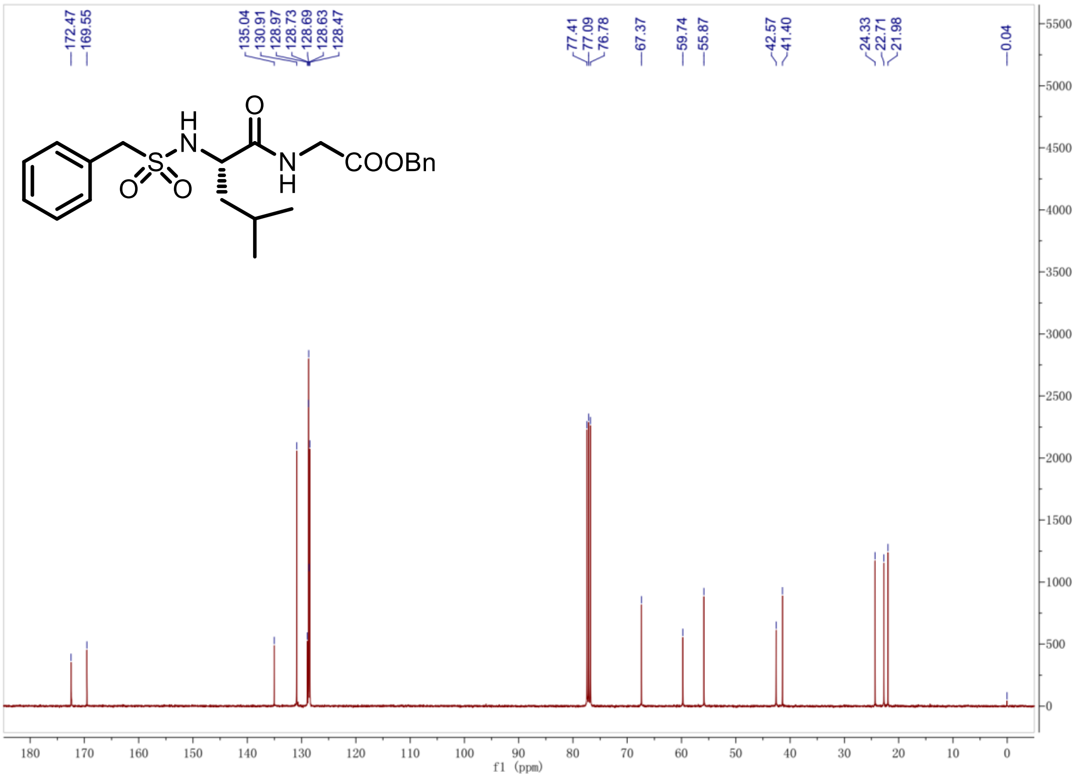

Supplementary Figure 106. ^{13}C NMR (100 MHz, $CDCl_3$) spectrum of compound **1m**

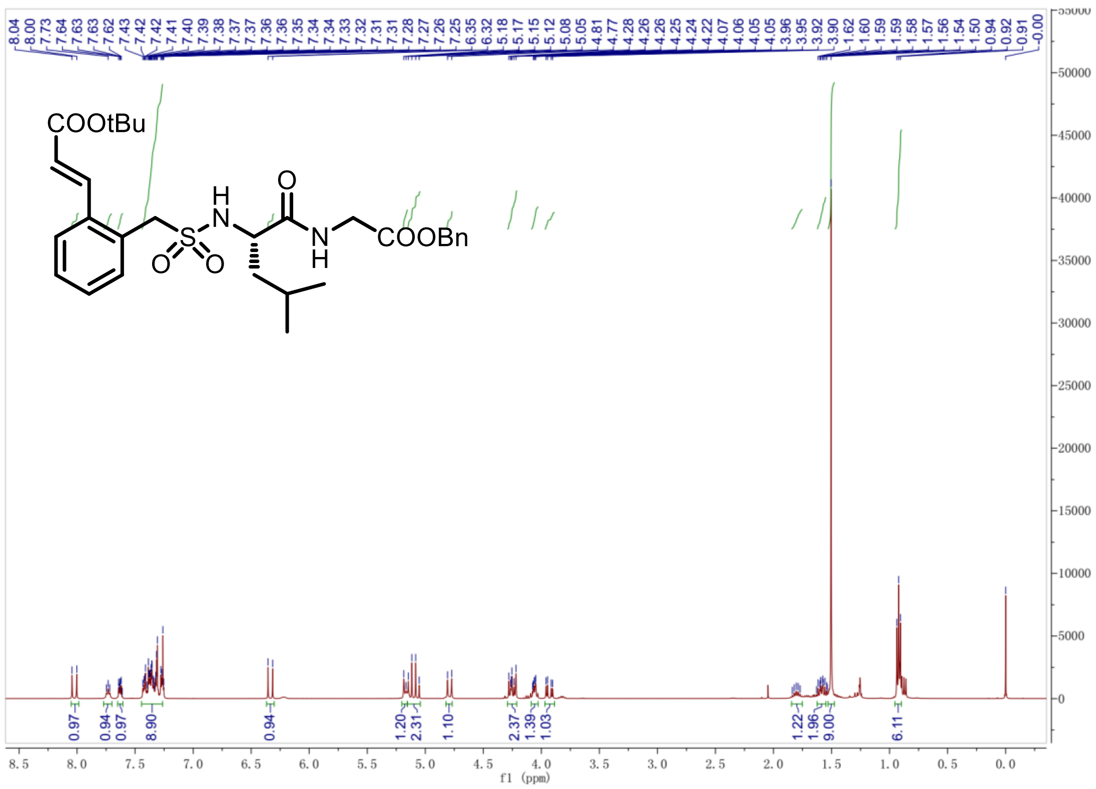

Supplementary Figure 107. ^{1}H NMR (400 MHz, $CDCl_3$) spectrum of **3ma** (mono)

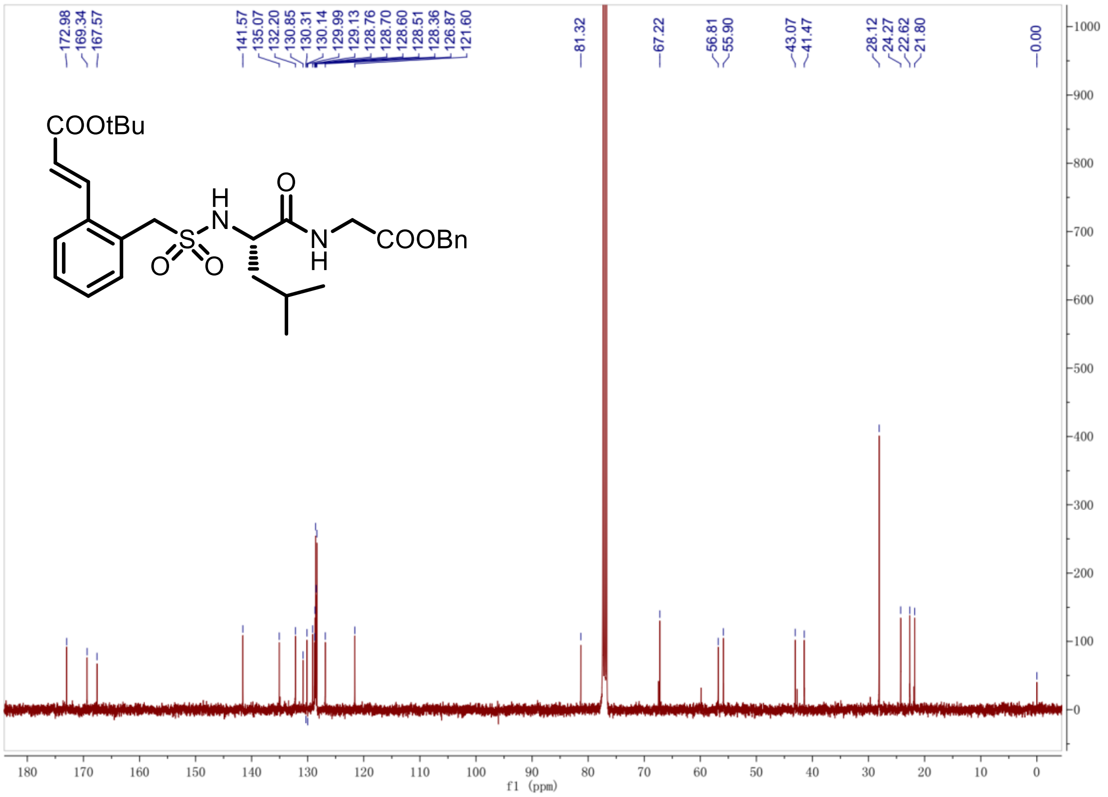

Supplementary Figure 108. ^{13}C NMR (100 MHz, $CDCl_3$) spectrum of **3ma** (mono)

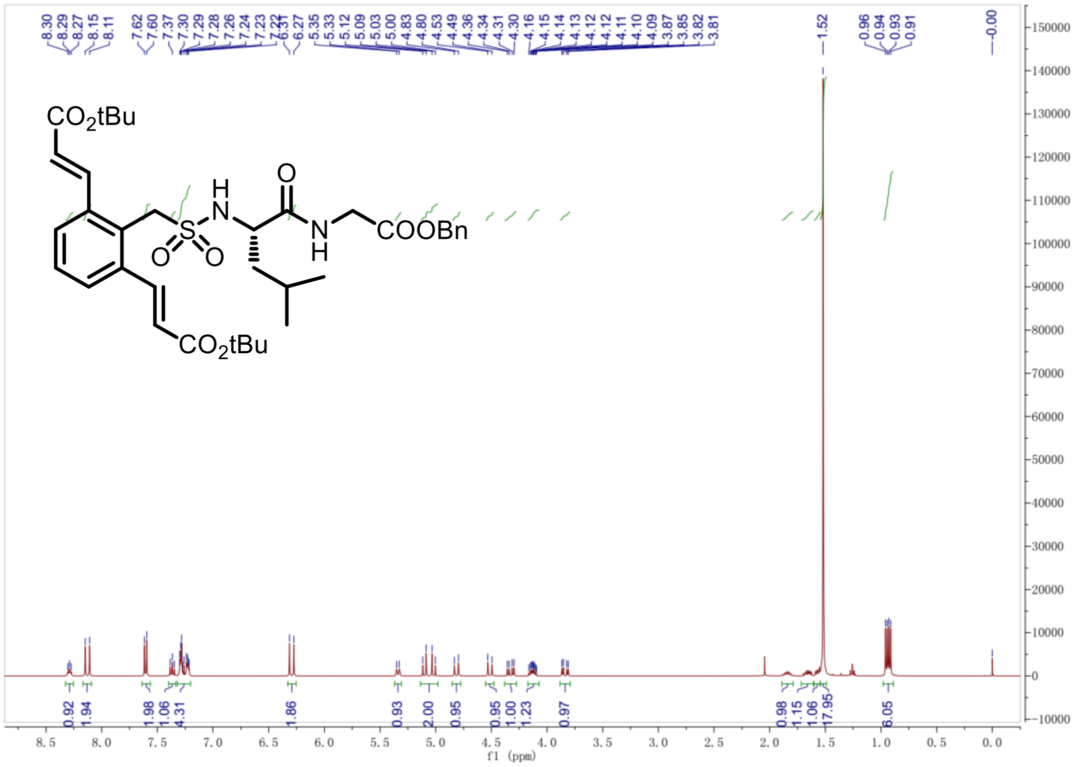

Supplementary Figure 109. ^{1}H NMR (400 MHz, $CDCl_3$) spectrum of **3ma** (di)

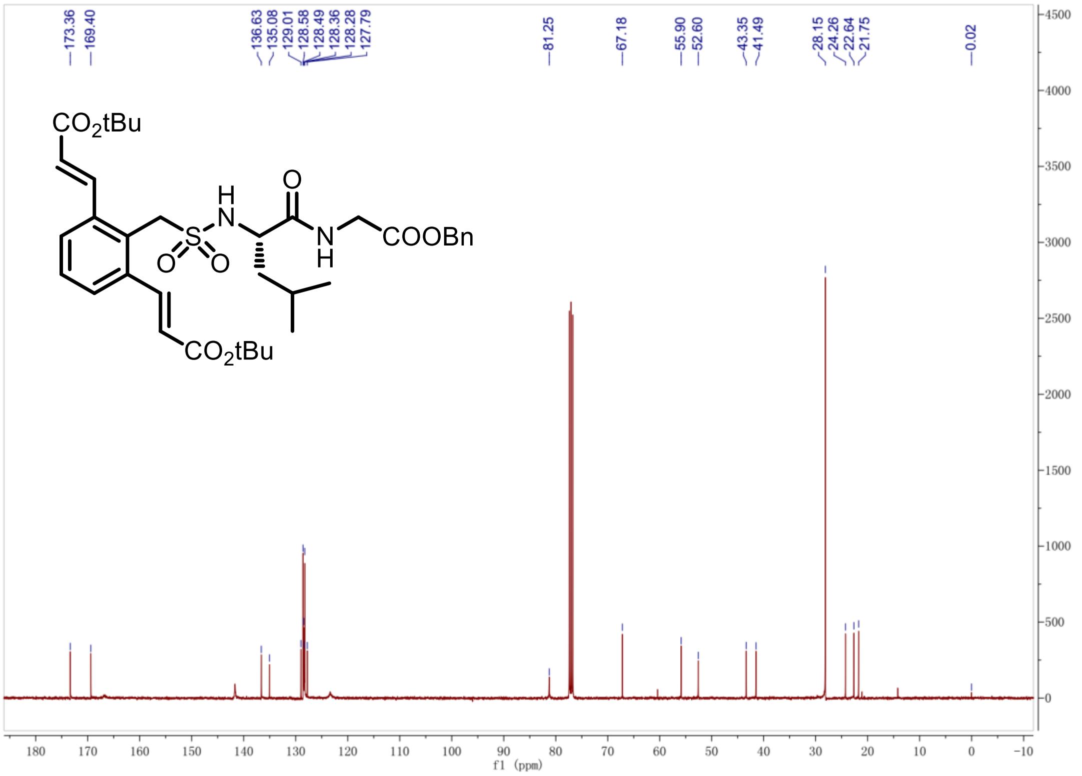

Supplementary Figure 110. ^{13}C NMR (100 MHz, $CDCl_3$) spectrum of **3ma** (di)

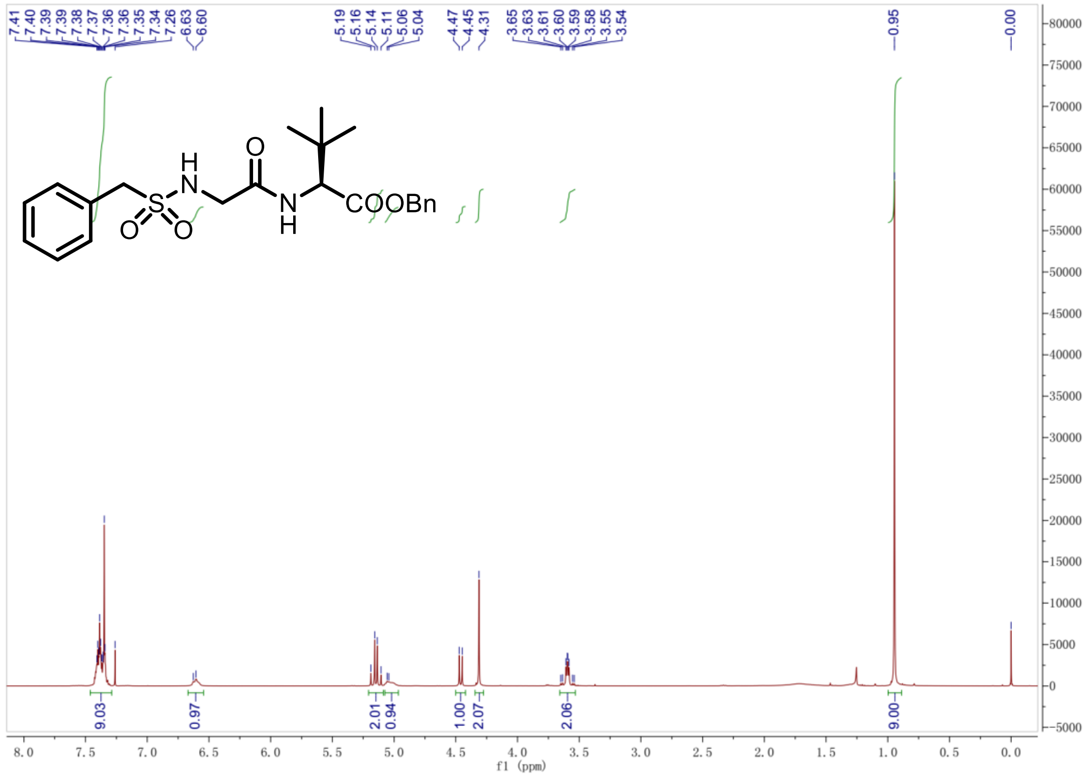

Supplementary Figure 111. ^{1}H NMR (400 MHz, $CDCl_3$) spectrum of compound **1n**

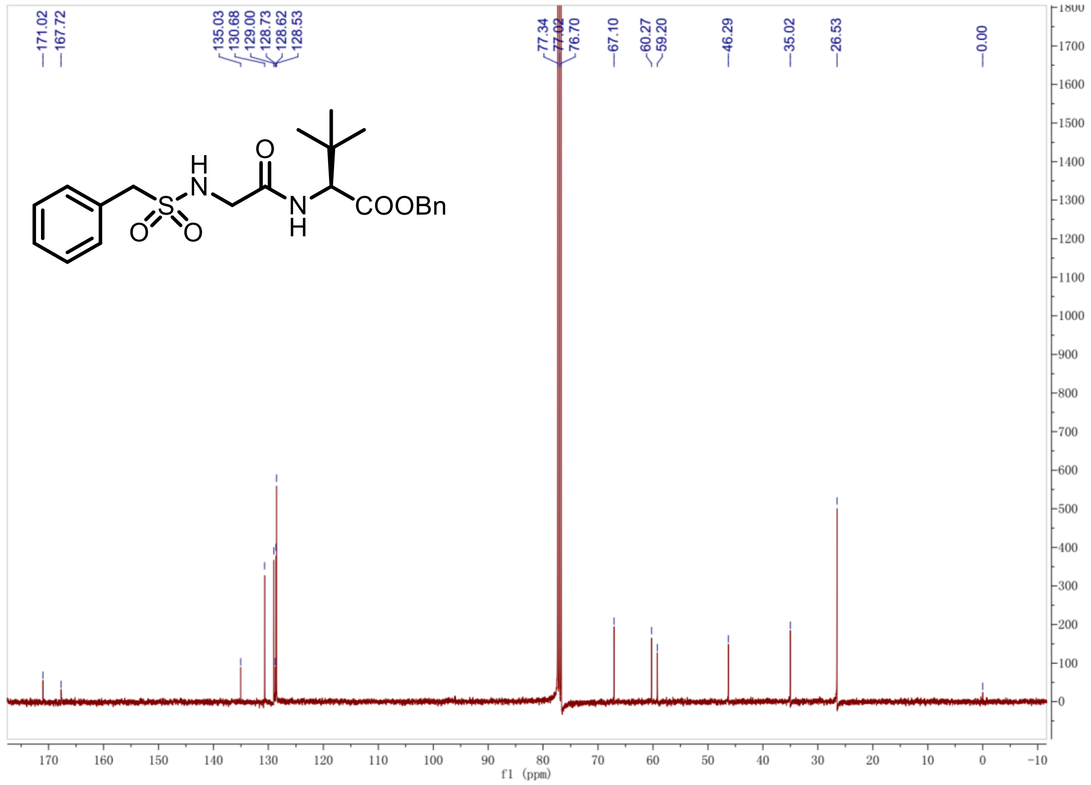

Supplementary Figure 112 ^{13}C NMR (100 MHz, $CDCl_3$) spectrum of compound **1n**

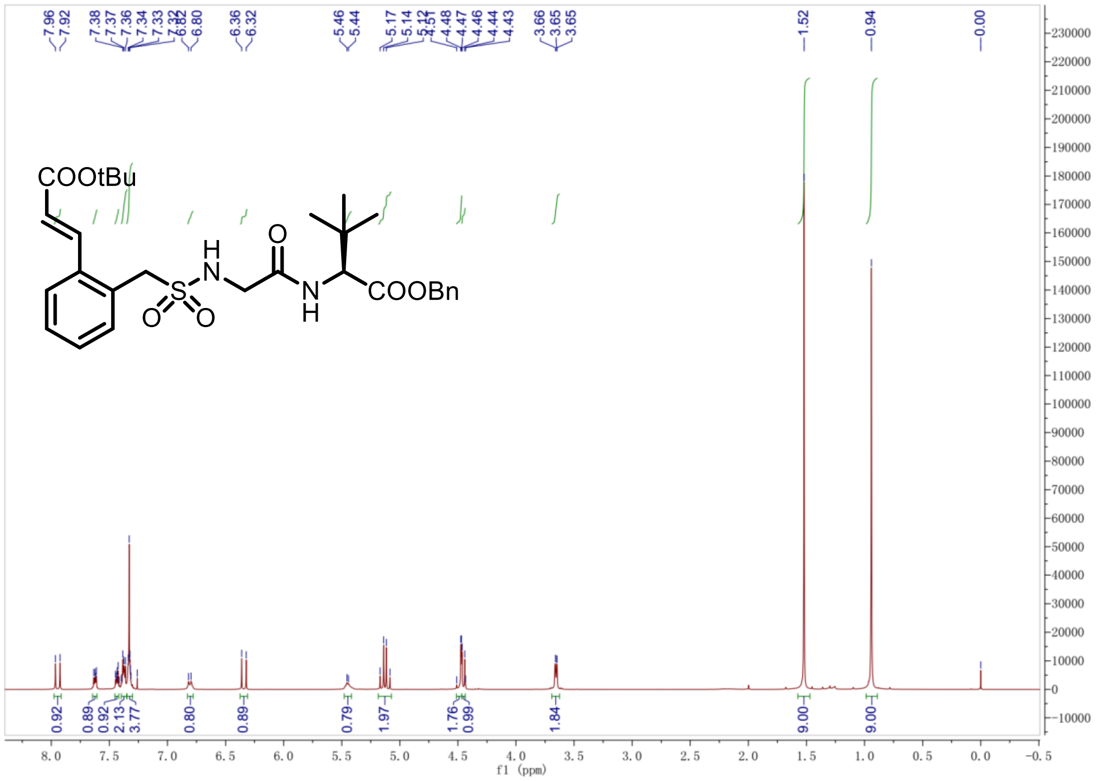

Supplementary Figure 113. ^{1}H NMR (500 MHz, $CDCl_3$) spectrum of **3na** (mono)

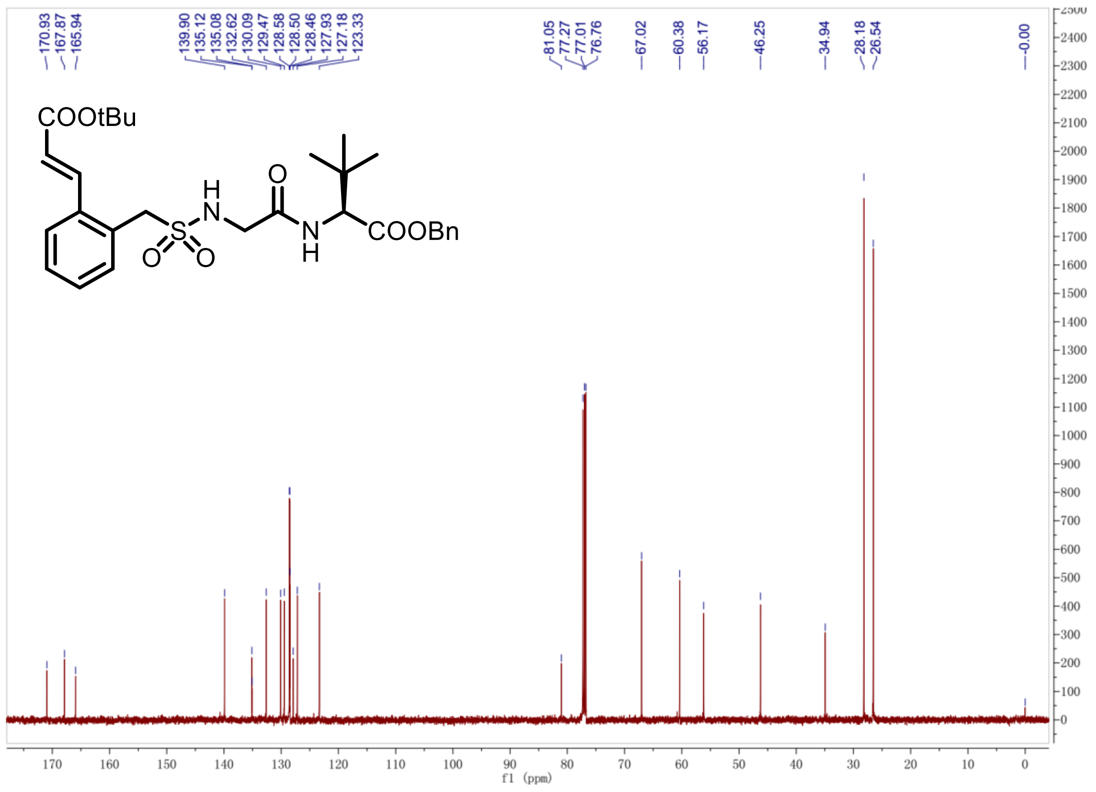

Supplementary Figure 114. ^{13}C NMR (125 MHz, $CDCl_3$) spectrum of **3na** (mono)

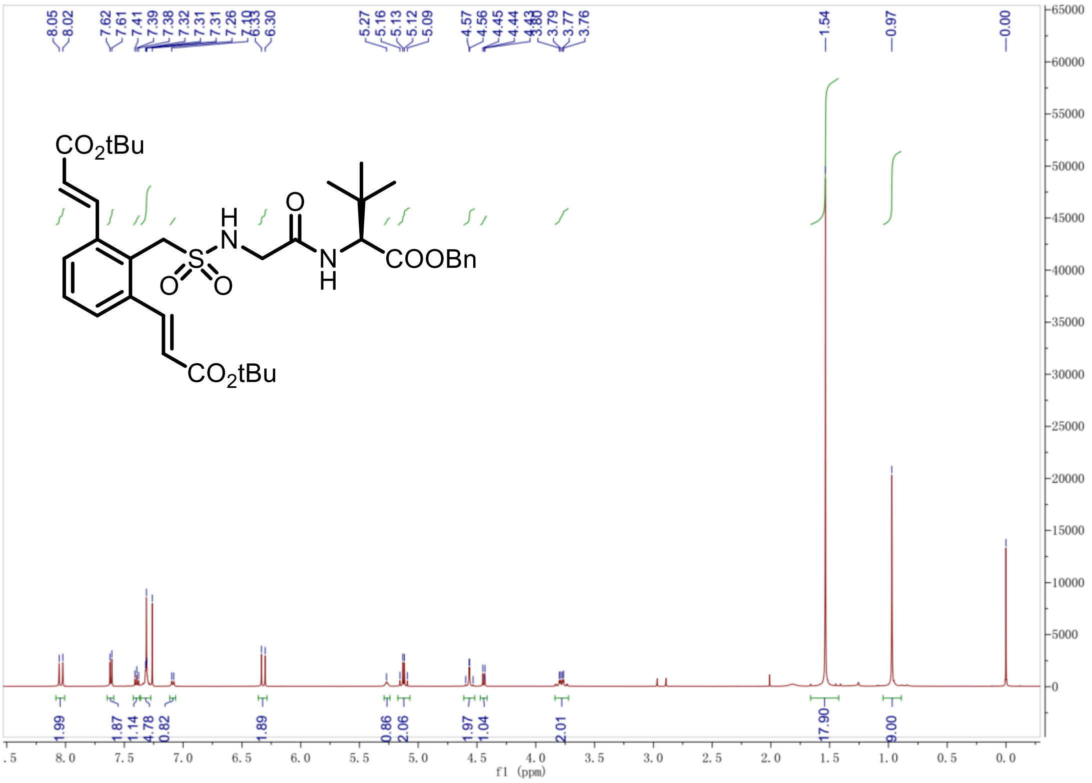

Supplementary Figure 115. ^{1}H NMR (500 MHz, $CDCl_3$) spectrum of **3na** (di)

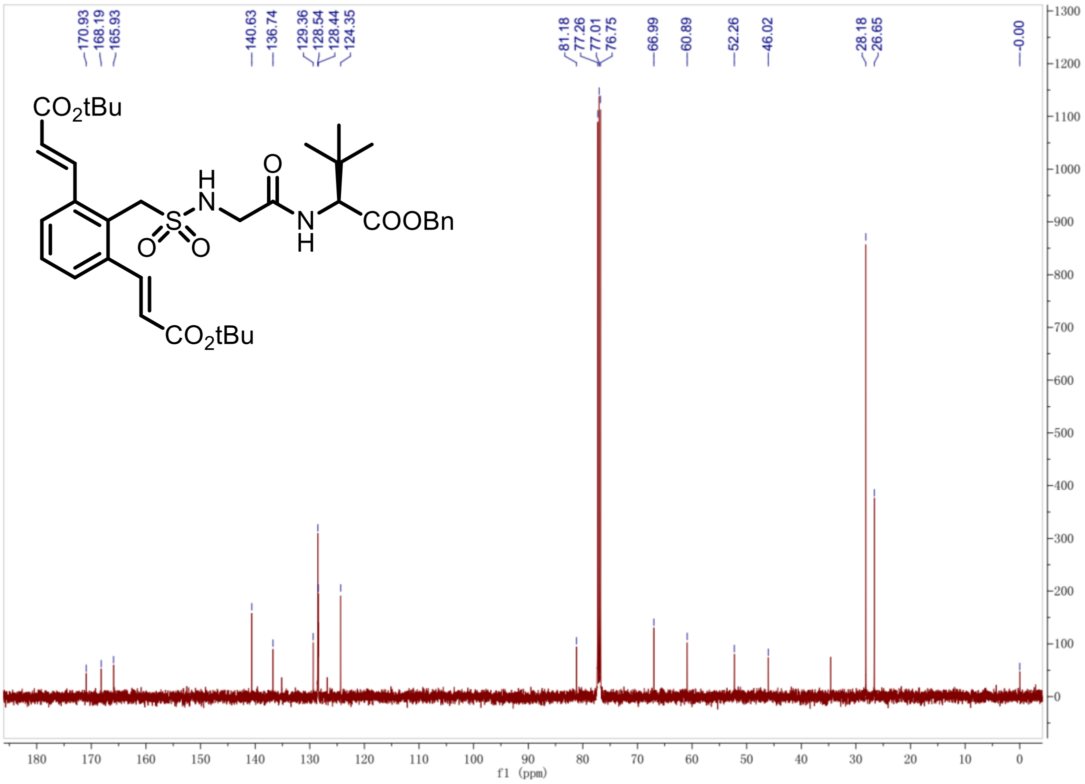

Supplementary Figure 116. ^{13}C NMR (125 MHz, $CDCl_3$) spectrum of **3na** (di)

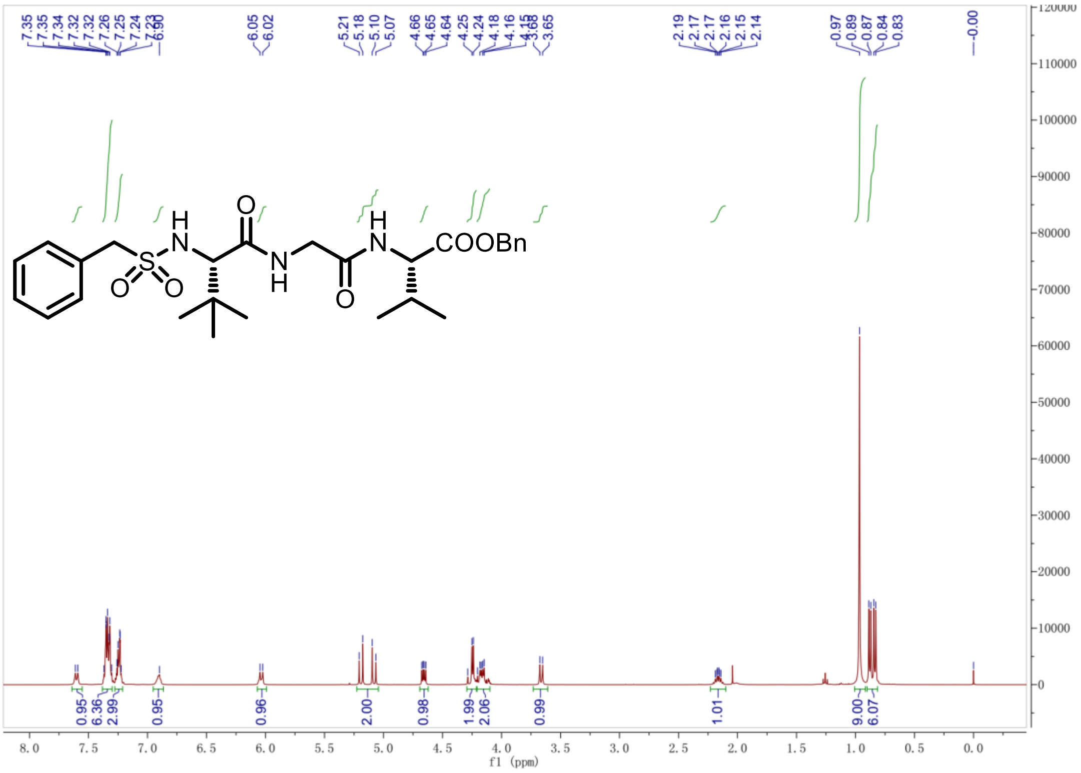

Supplementary Figure 117. ^{1}H NMR (400 MHz, $CDCl_3$) spectrum of compound **1o**

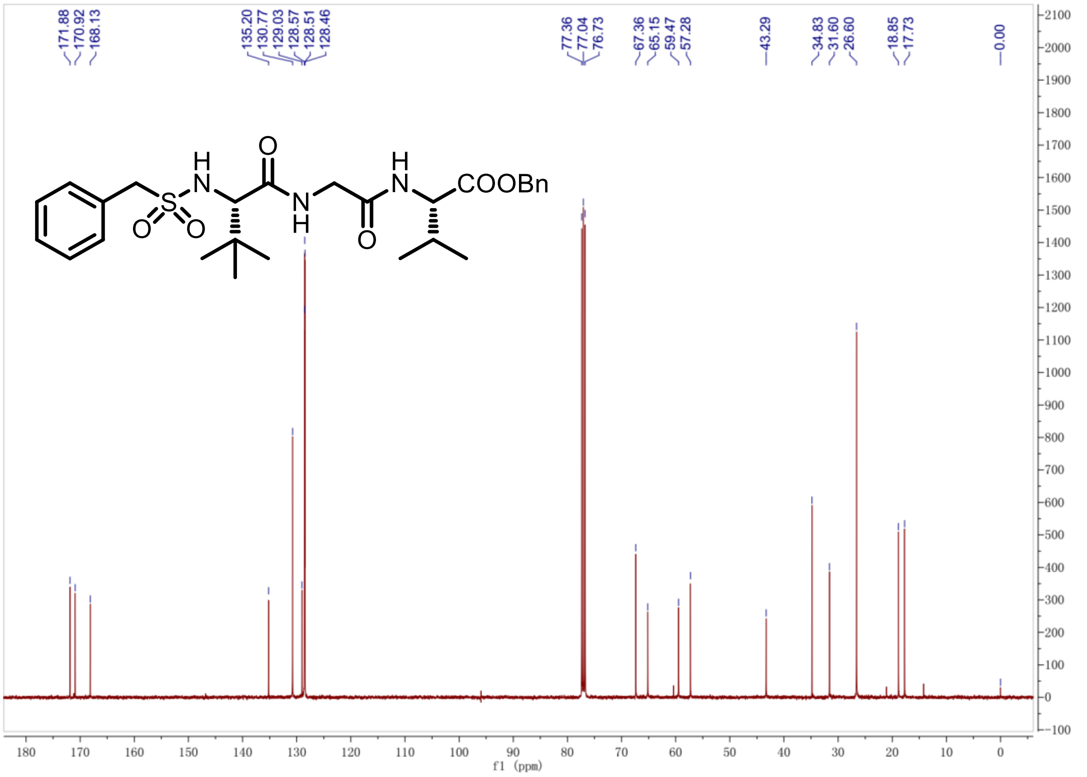

Supplementary Figure 118. ^{13}C NMR (100 MHz, $CDCl_3$) spectrum of compound **1o**

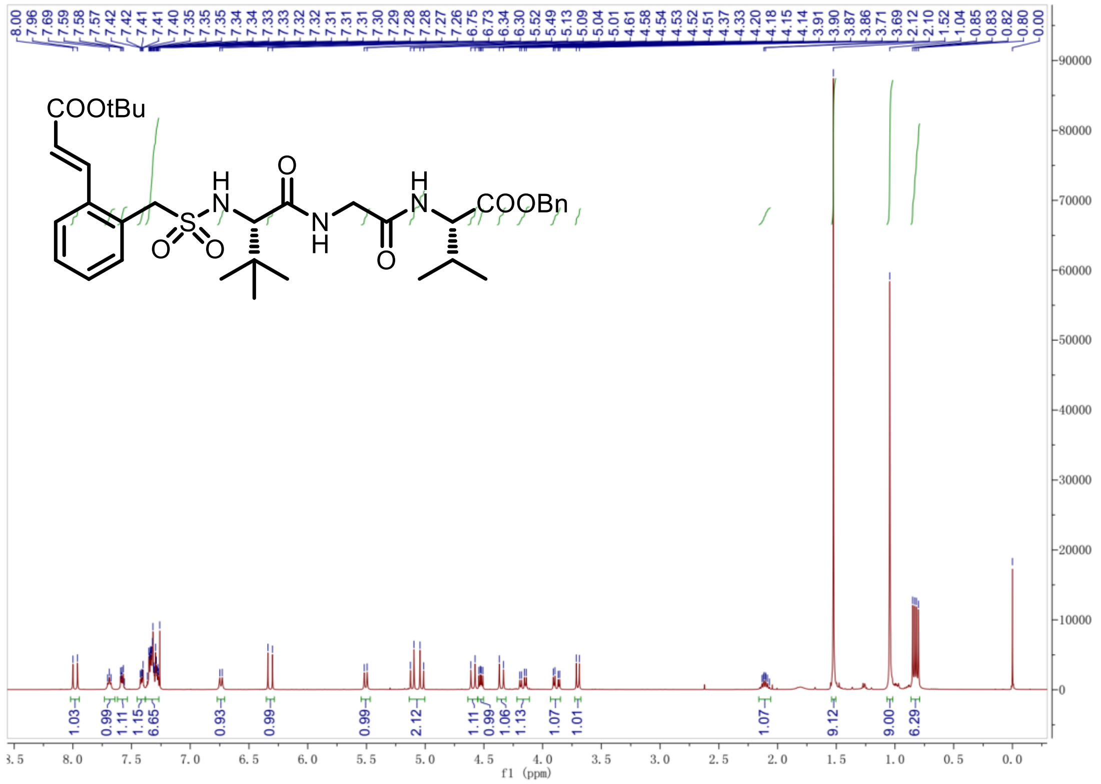

Supplementary Figure 119. ^{1}H NMR (400 MHz, $CDCl_3$) spectrum of **3oa** (mono)

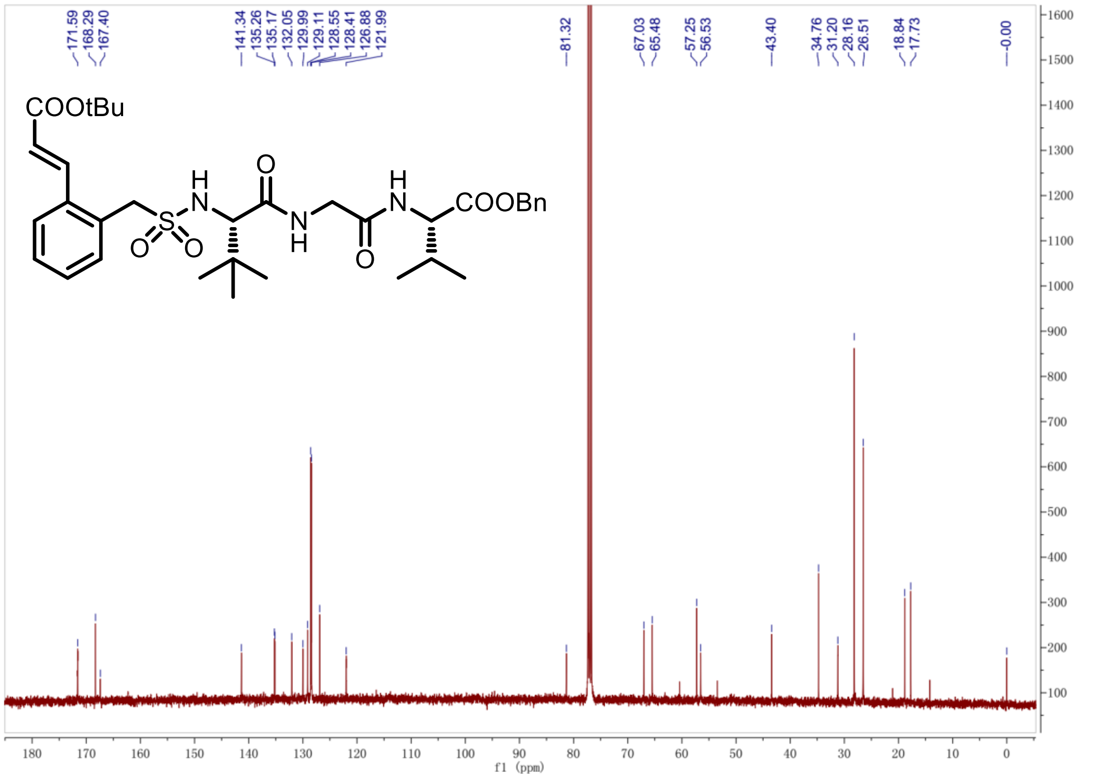

Supplementary Figure 120. ^{13}C NMR (100 MHz, $CDCl_3$) spectrum of **3oa** (mono)

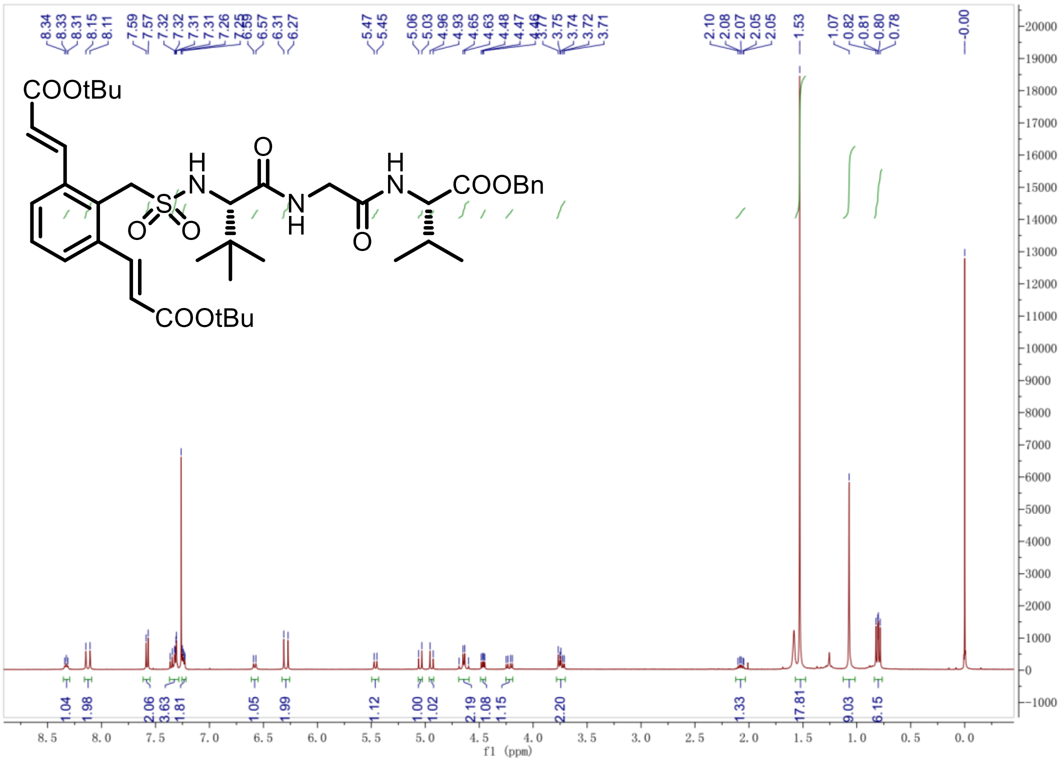

Supplementary Figure 121. ^{1}H NMR (400 MHz, $CDCl_3$) spectrum of **3oa** (di)

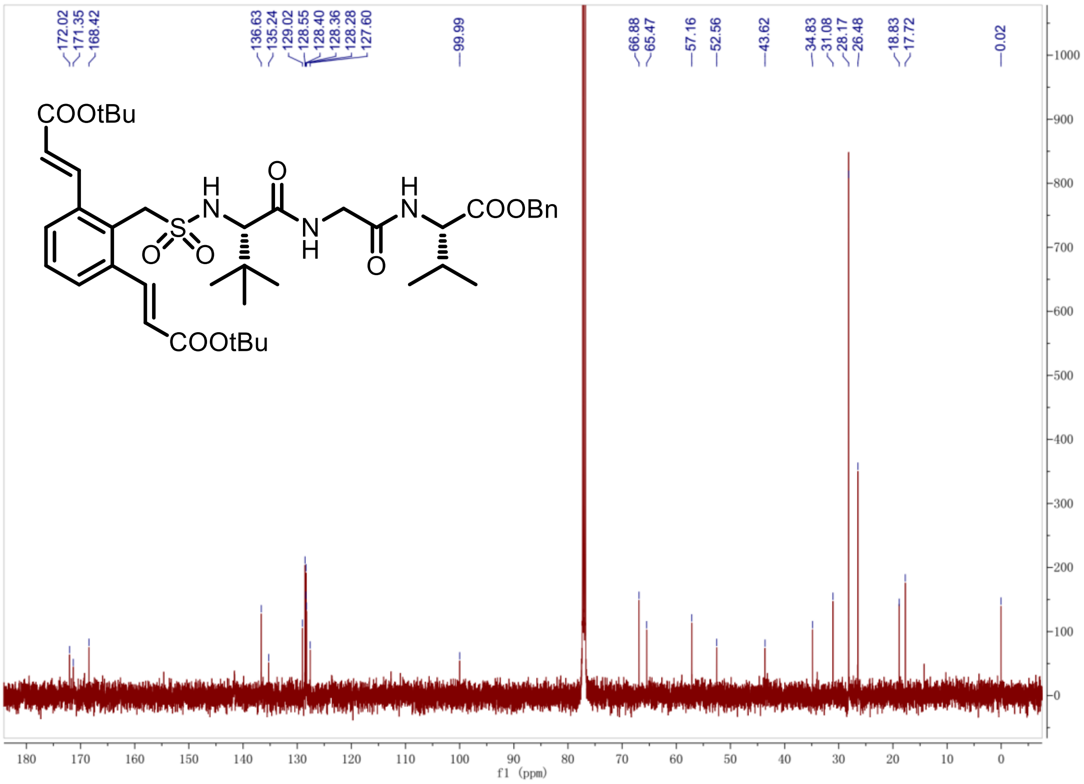

Supplementary Figure 122. ^{13}C NMR (100 MHz, $CDCl_3$) spectrum of **3oa** (di)

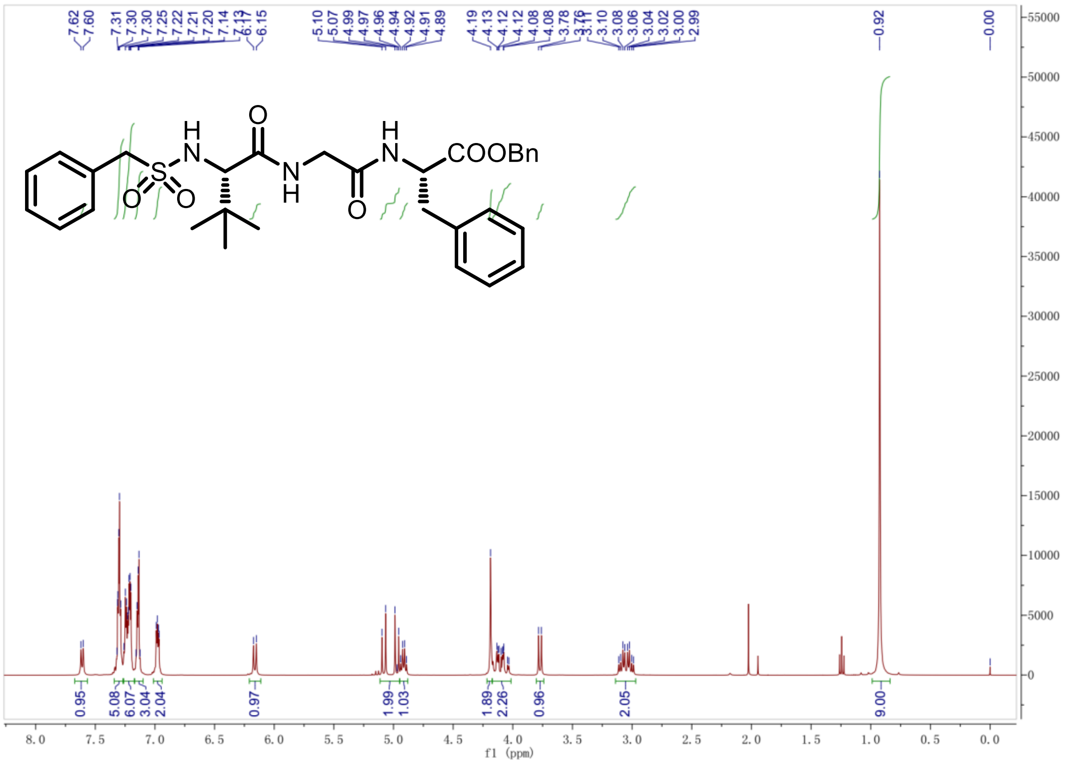

Supplementary Figure 123. ^{1}H NMR (400 MHz, $CDCl_3$) spectrum of compound **1p**

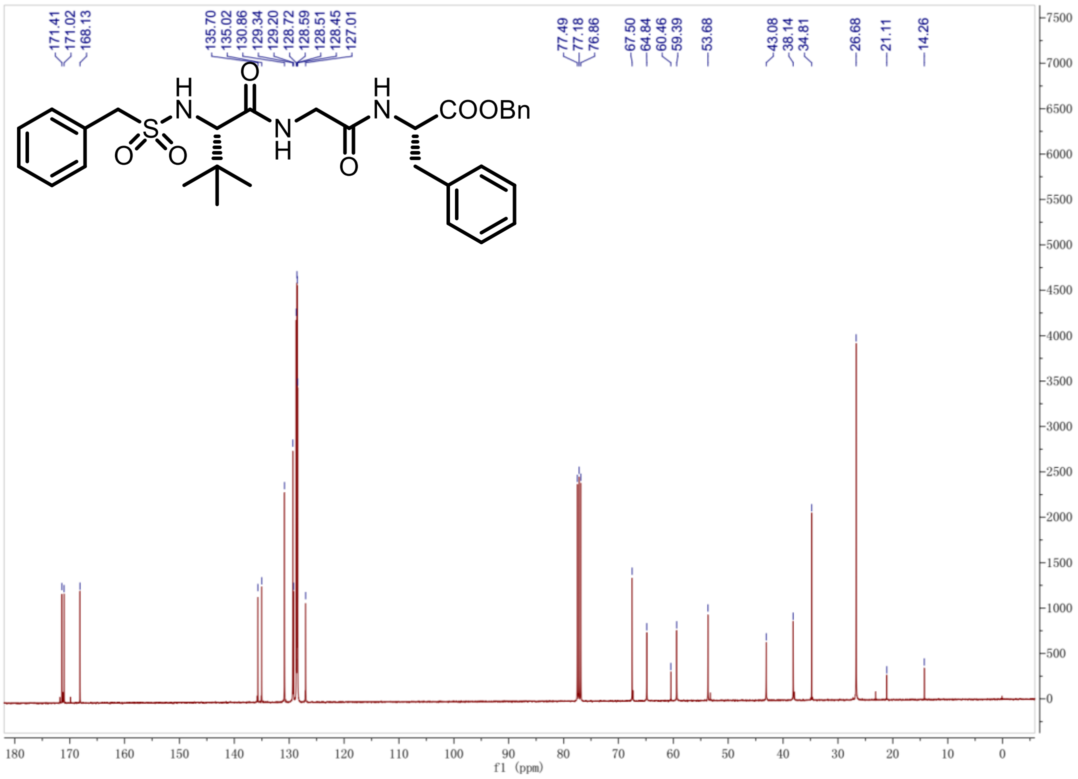

Supplementary Figure 124. ^{13}C NMR (100 MHz, $CDCl_3$) spectrum of compound **1p**

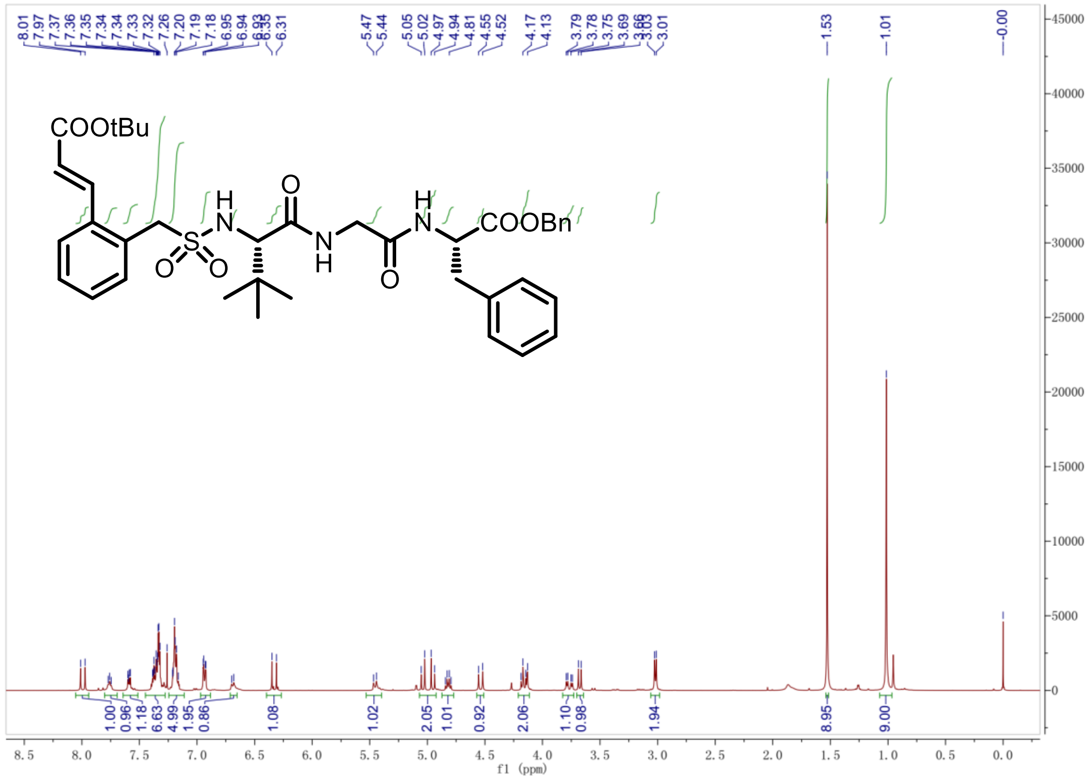

Supplementary Figure 125. ^{1}H NMR (400 MHz, $CDCl_3$) spectrum of **3pa** (mono)

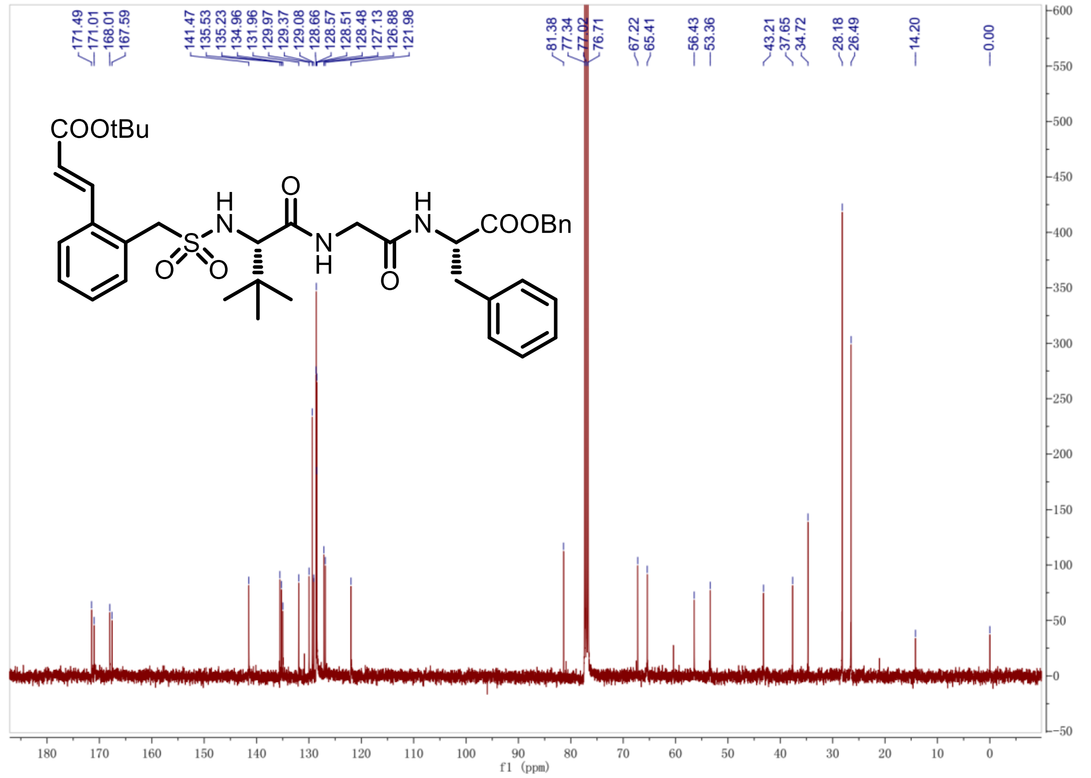

Supplementary Figure 126. ^{13}C NMR (100 MHz, $CDCl_3$) spectrum of **3pa** (mono)

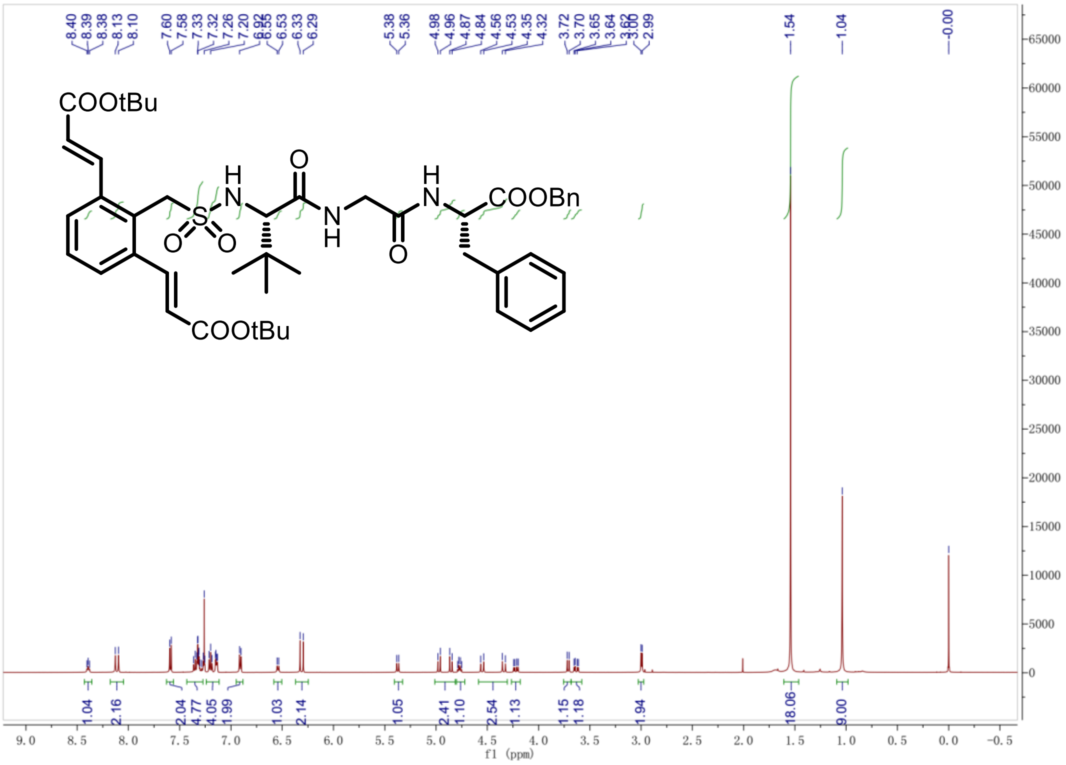

Supplementary Figure 127. ^{1}H NMR (500 MHz, $CDCl_3$) spectrum of **3pa** (di)

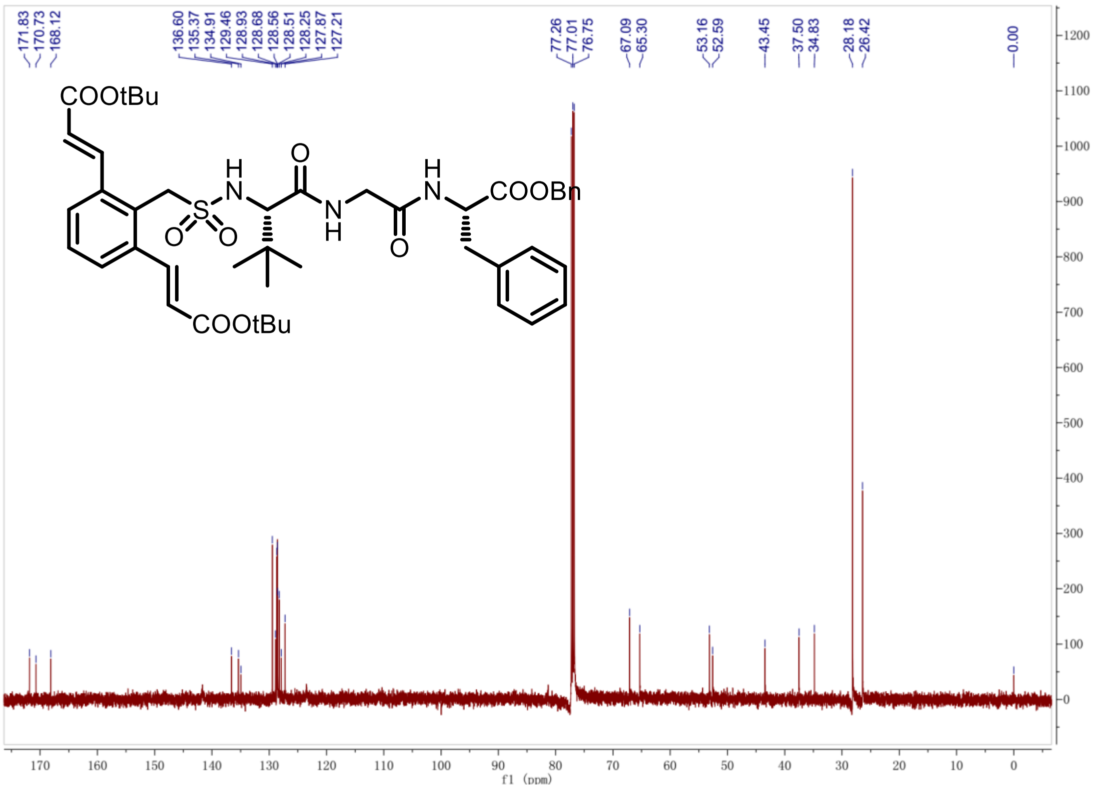

Supplementary Figure 128. ^{13}C NMR (125 MHz, $CDCl_3$) spectrum of **3pa** (di)

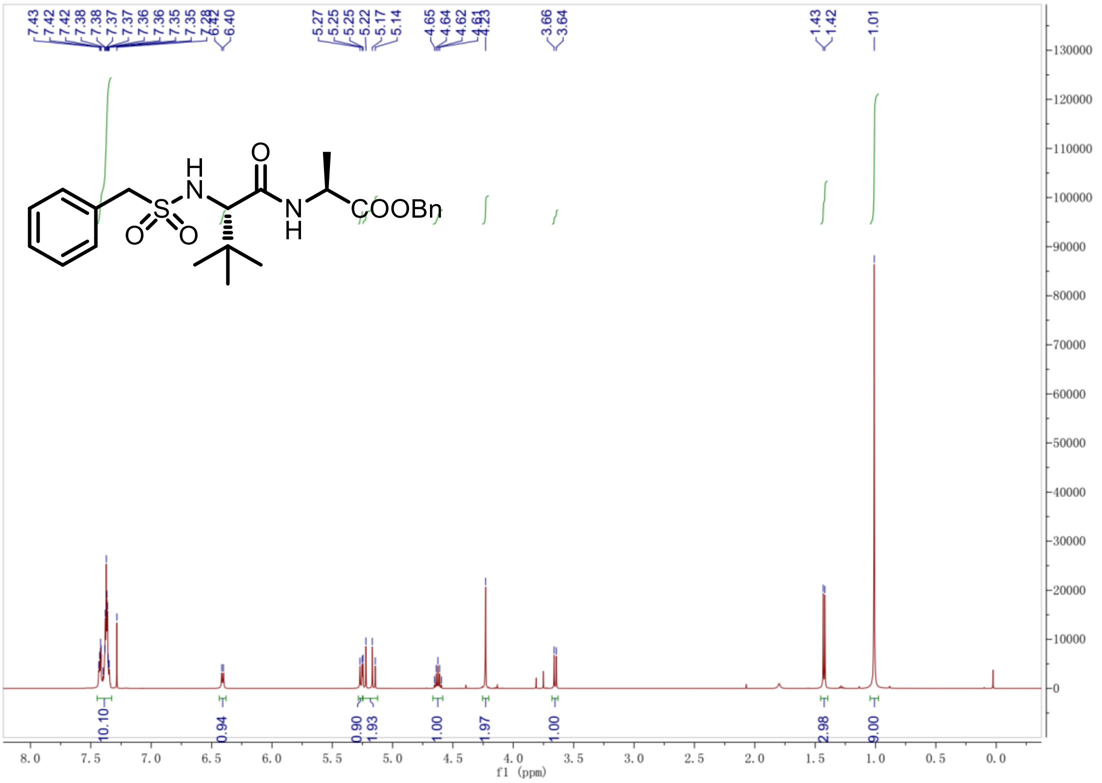

Supplementary Figure 129. ^{1}H NMR (500 MHz, $CDCl_3$) spectrum of compound **3q**

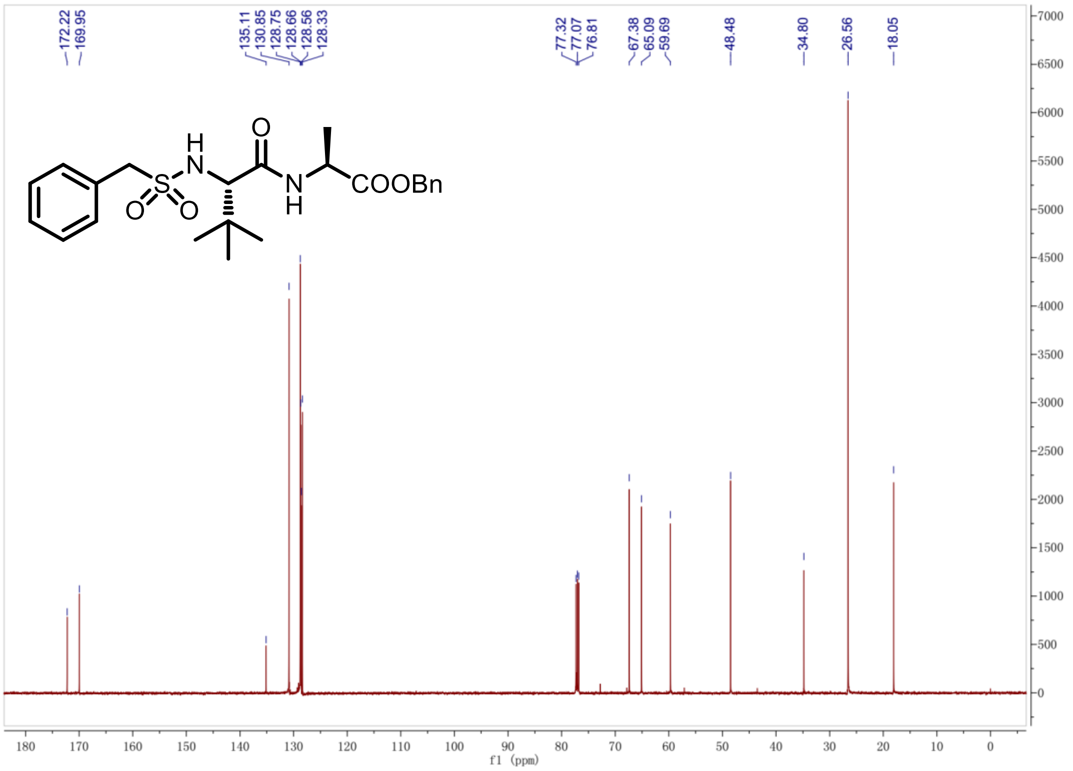

Supplementary Figure 130. ^{13}C NMR (125 MHz, $CDCl_3$) spectrum of compound **3q**

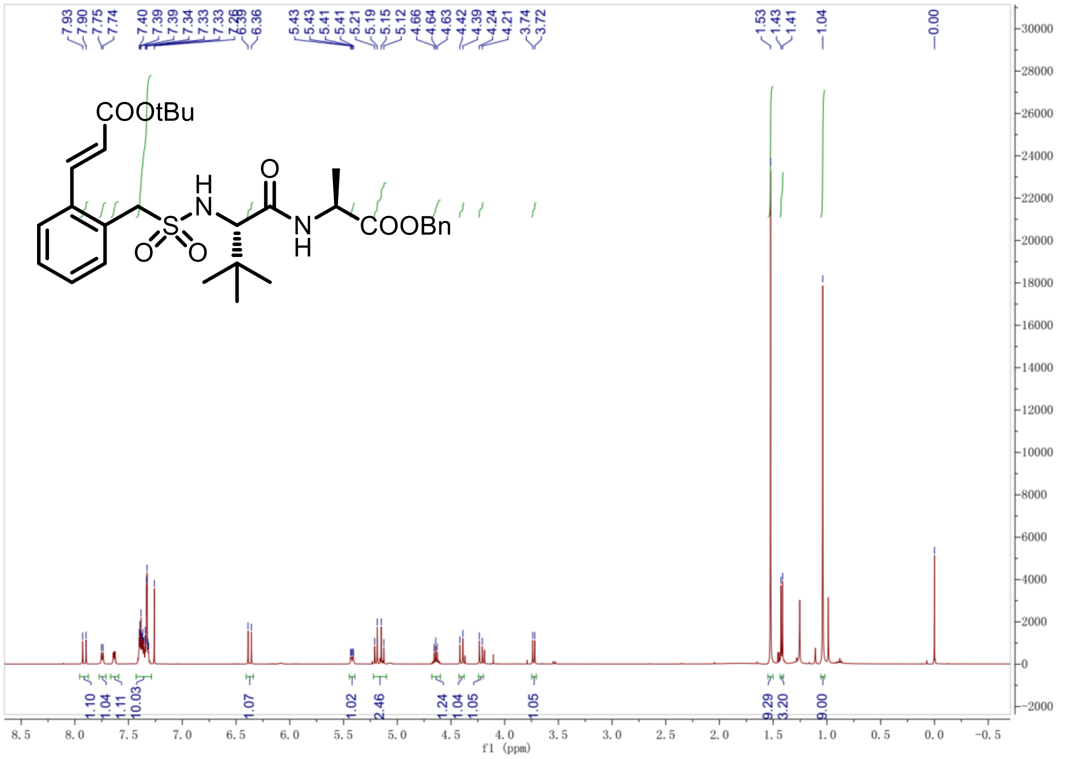

Supplementary Figure 131. ^{1}H NMR (500 MHz, $CDCl_3$) spectrum of **3qa** (mono)

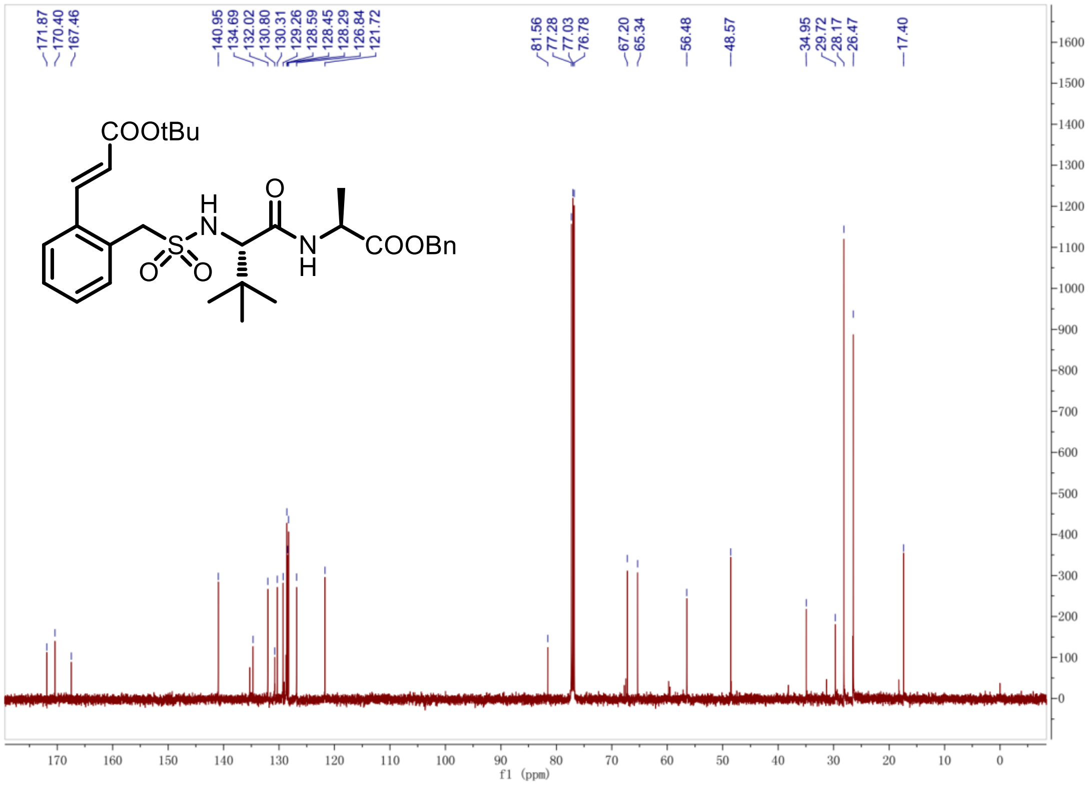

Supplementary Figure 132. ^{13}C NMR (125 MHz, $CDCl_3$) spectrum of **3qa** (mono)

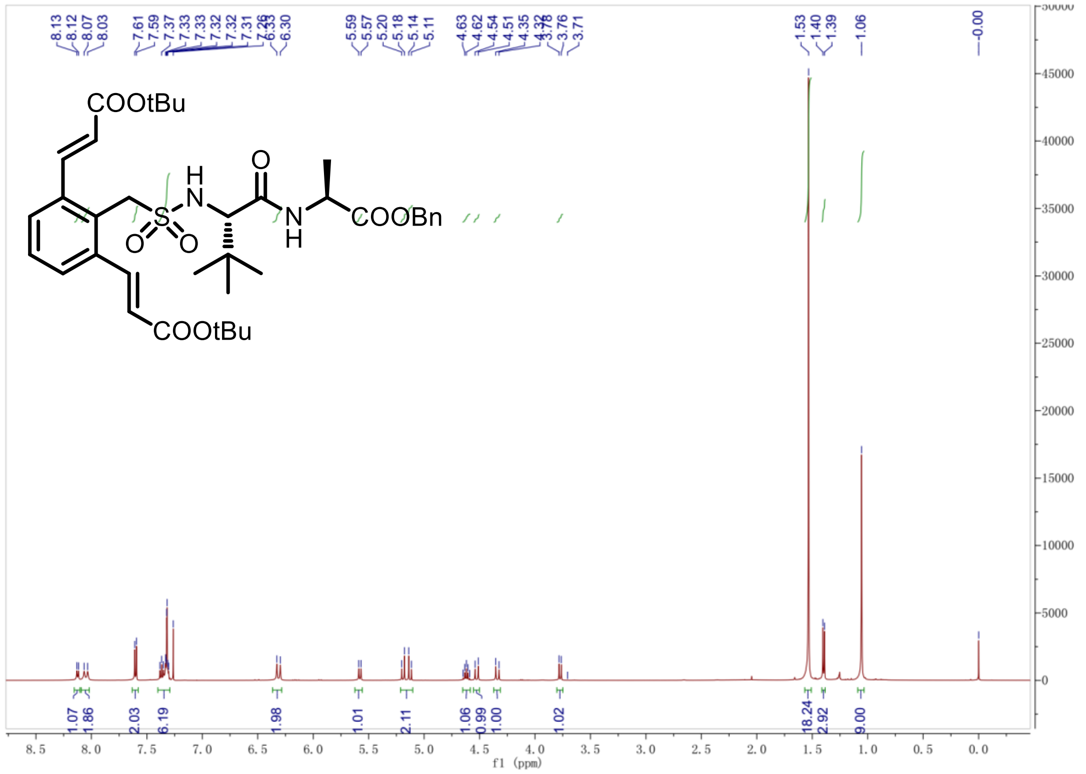

Supplementary Figure 133. ^{1}H NMR (500 MHz, $CDCl_3$) spectrum of **3qa** (di)

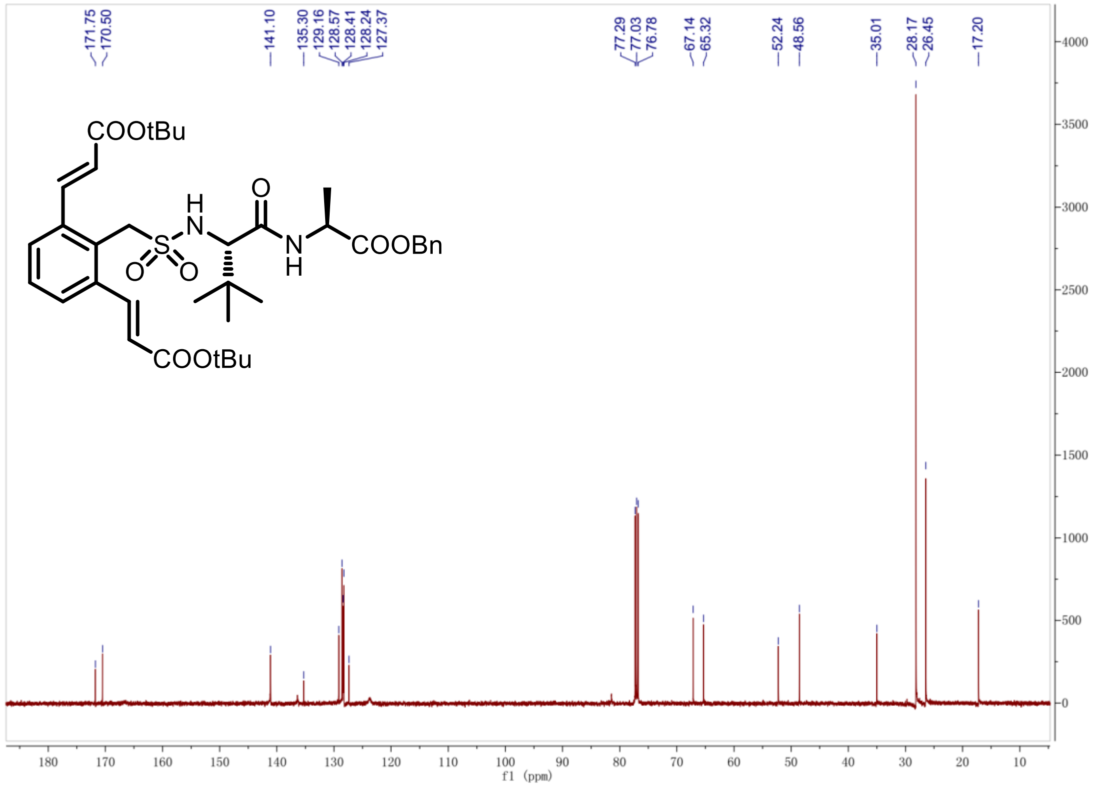

Supplementary Figure 134. ^{13}C NMR (125 MHz, $CDCl_3$) spectrum of **3qa** (di)

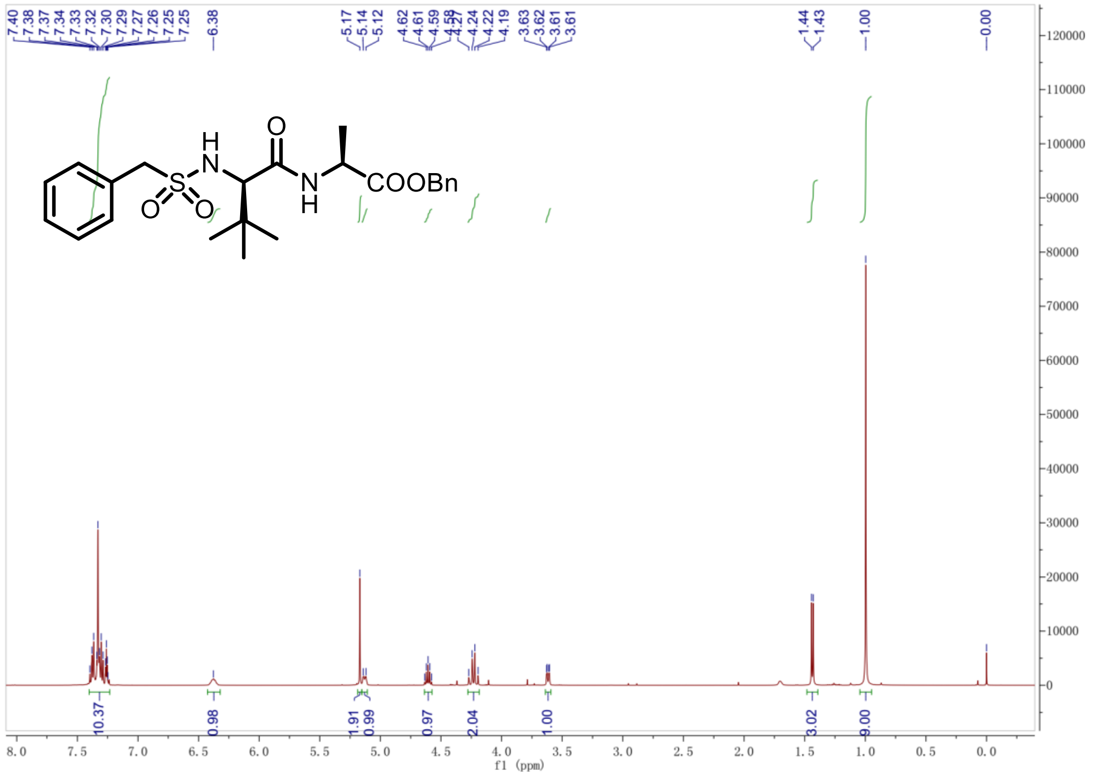

Supplementary Figure 135. ^{1}H NMR (500 MHz, $CDCl_3$) spectrum of **3q'**

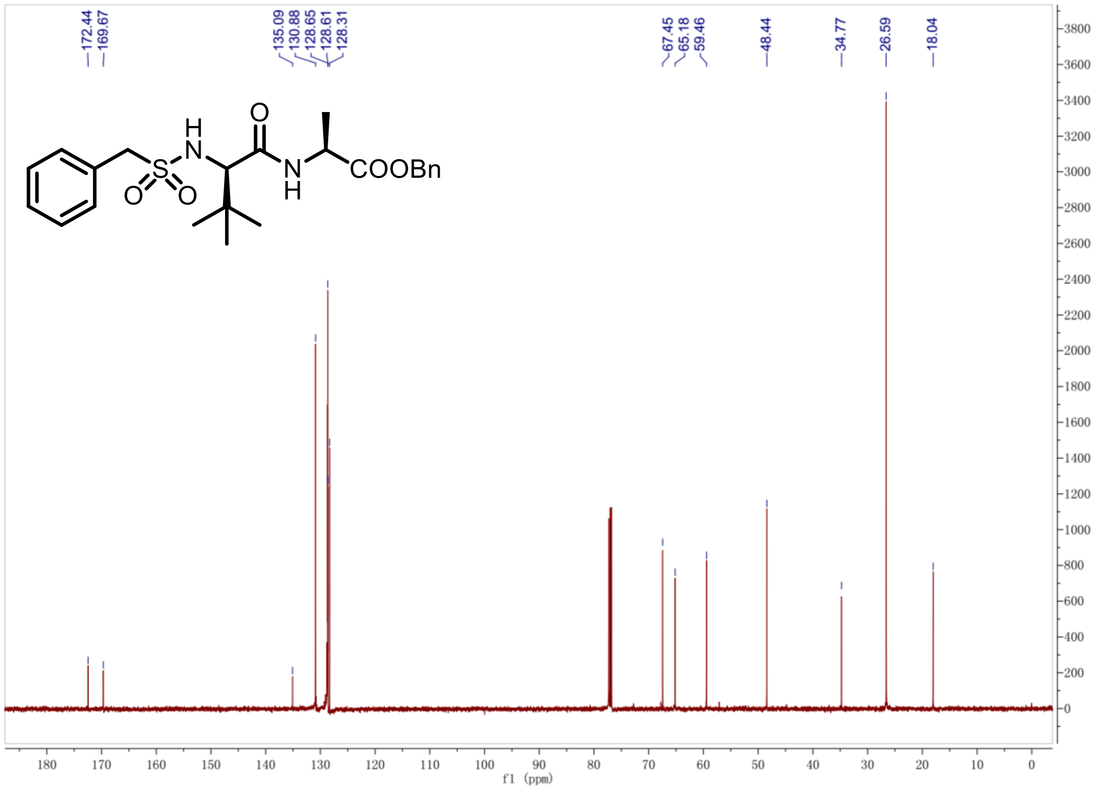

Supplementary Figure 136. ^{13}C NMR (125 MHz, $CDCl_3$) spectrum of **3q'**

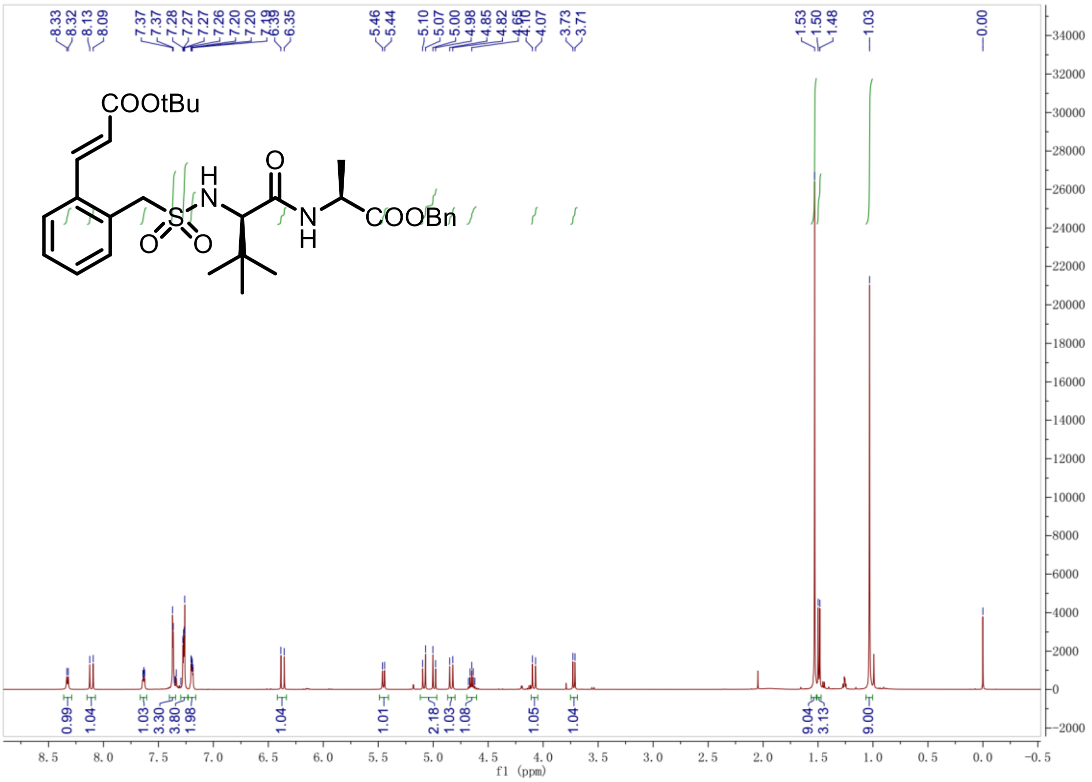

Supplementary Figure 137. ^{1}H NMR (500 MHz, $CDCl_3$) spectrum of **3q’a** (mono)

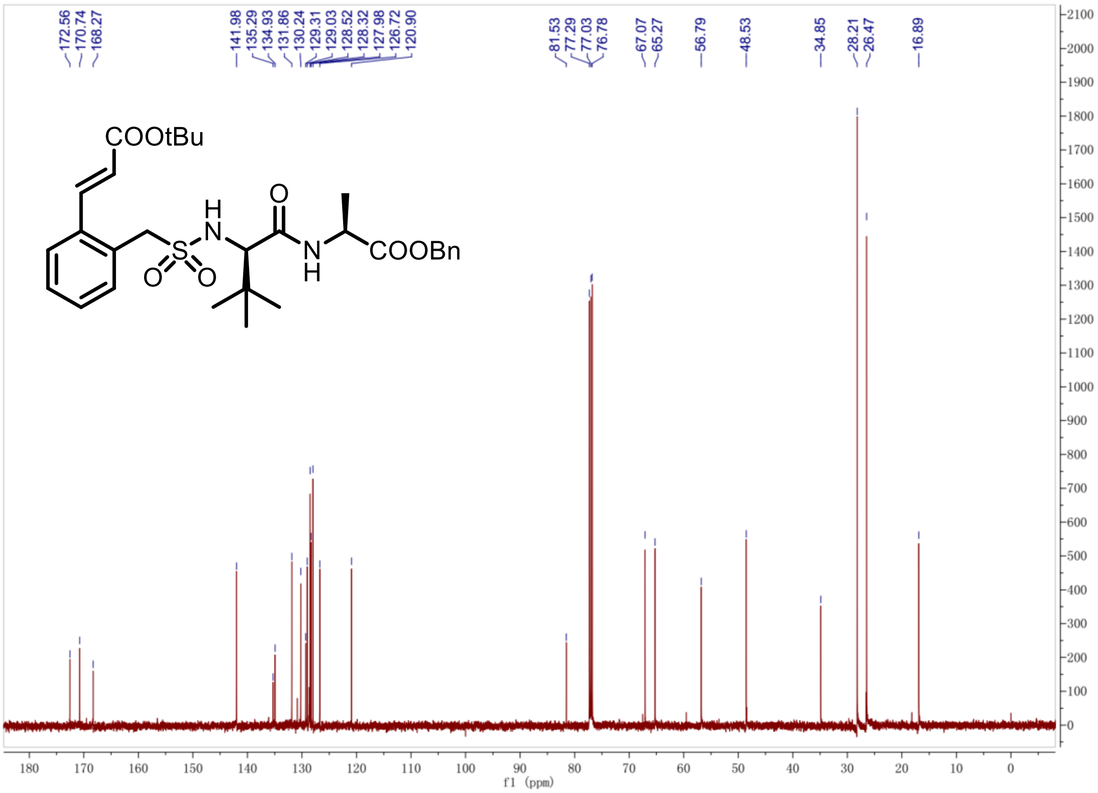

Supplementary Figure 138. ^{13}C NMR (125 MHz, $CDCl_3$) spectrum of **3q’a** (mono)

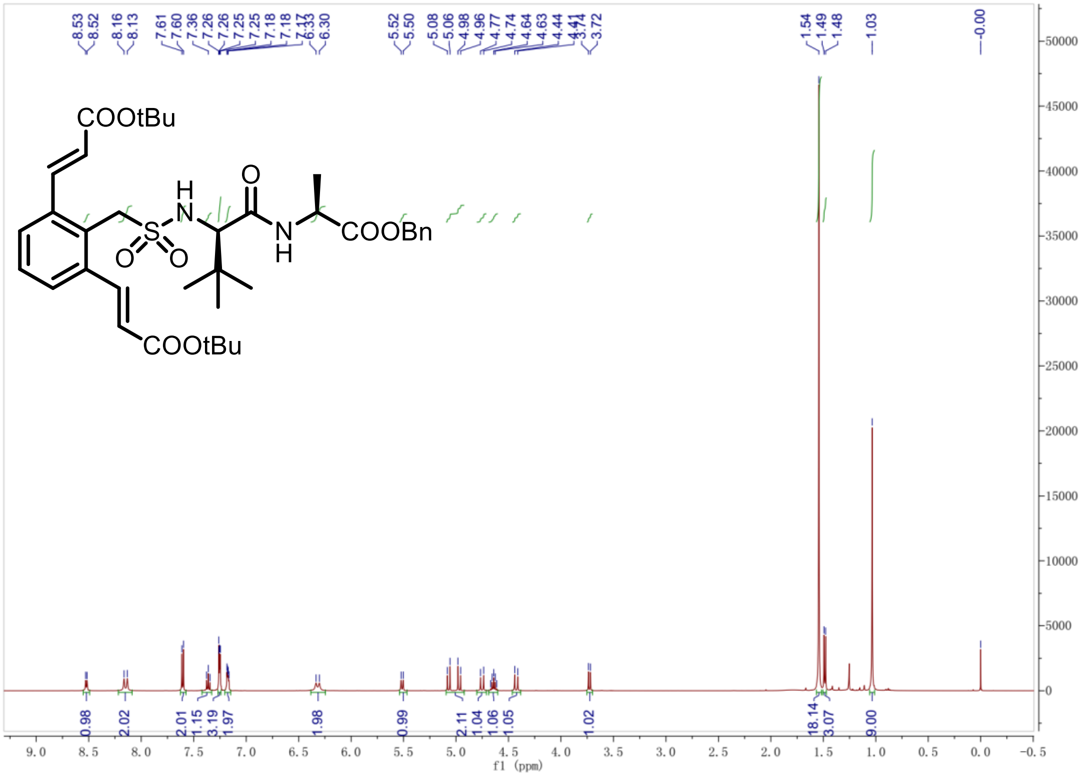

Supplementary Figure 139 ^{1}H NMR (500 MHz, $CDCl_3$) spectrum of **3q’a** (di)

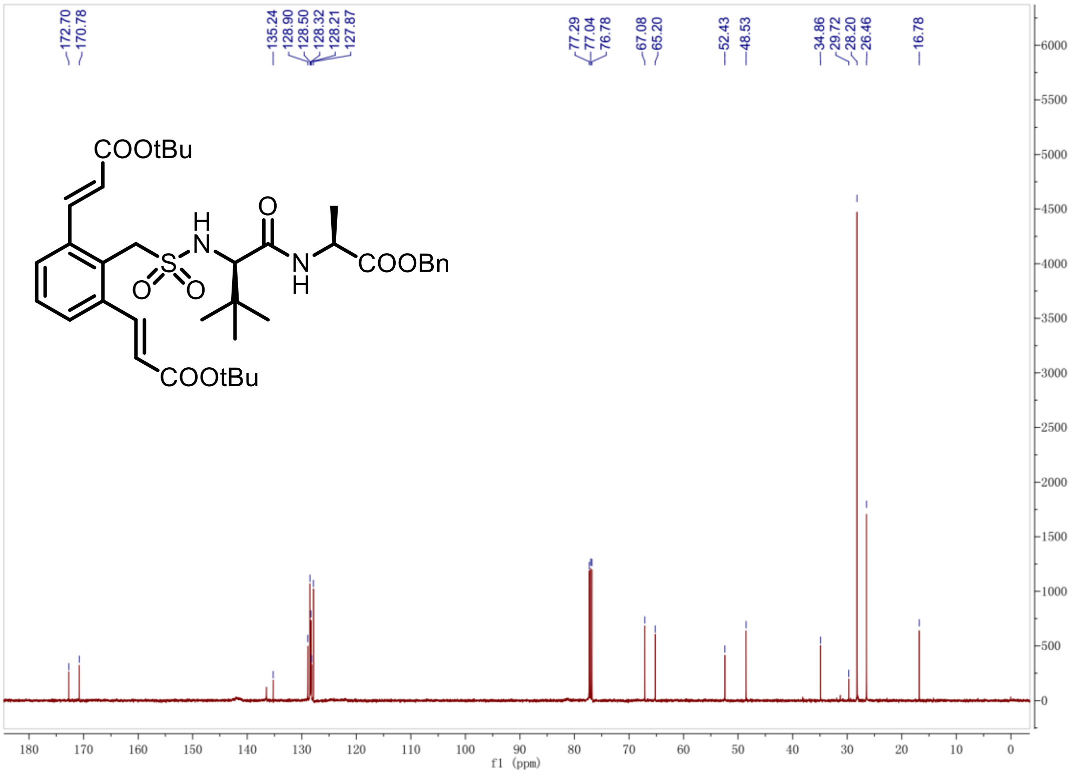

Supplementary Figure 140. ^{13}C NMR (125 MHz, $CDCl_3$) spectrum of **3q’a** (di)

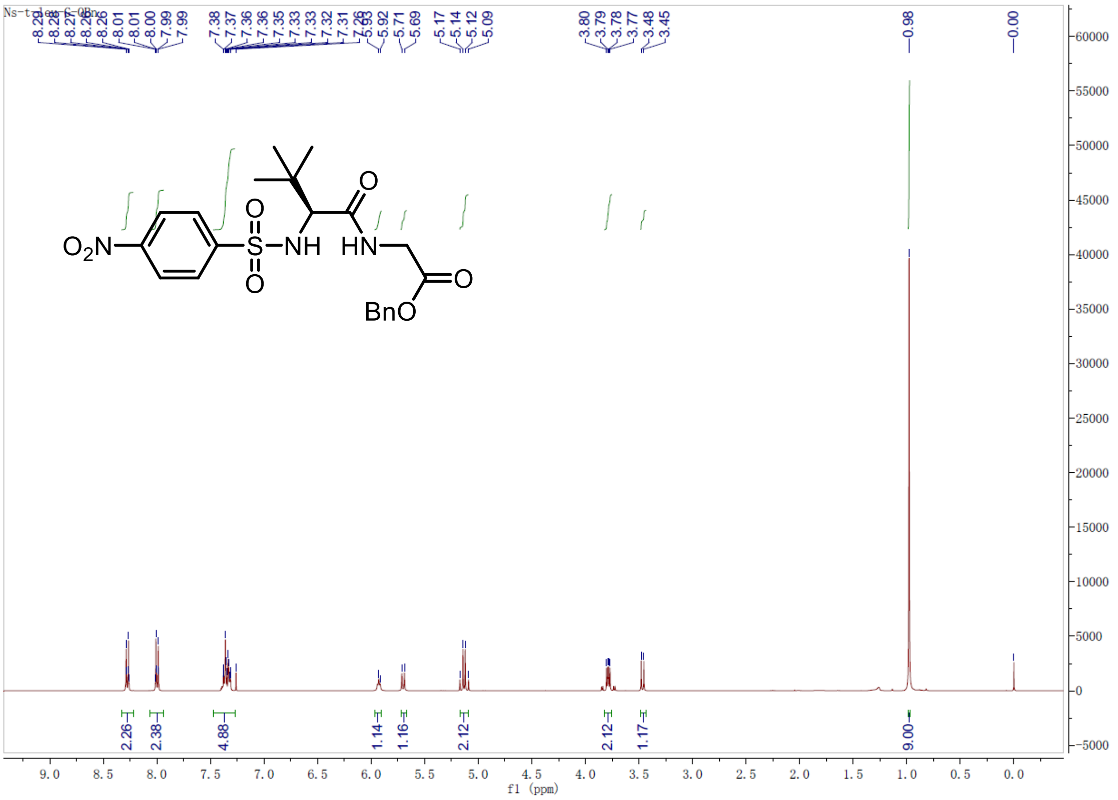

Supplementary Figure 141. ^{1}H NMR (400 MHz, $CDCl_3$) spectrum of compound **4a**

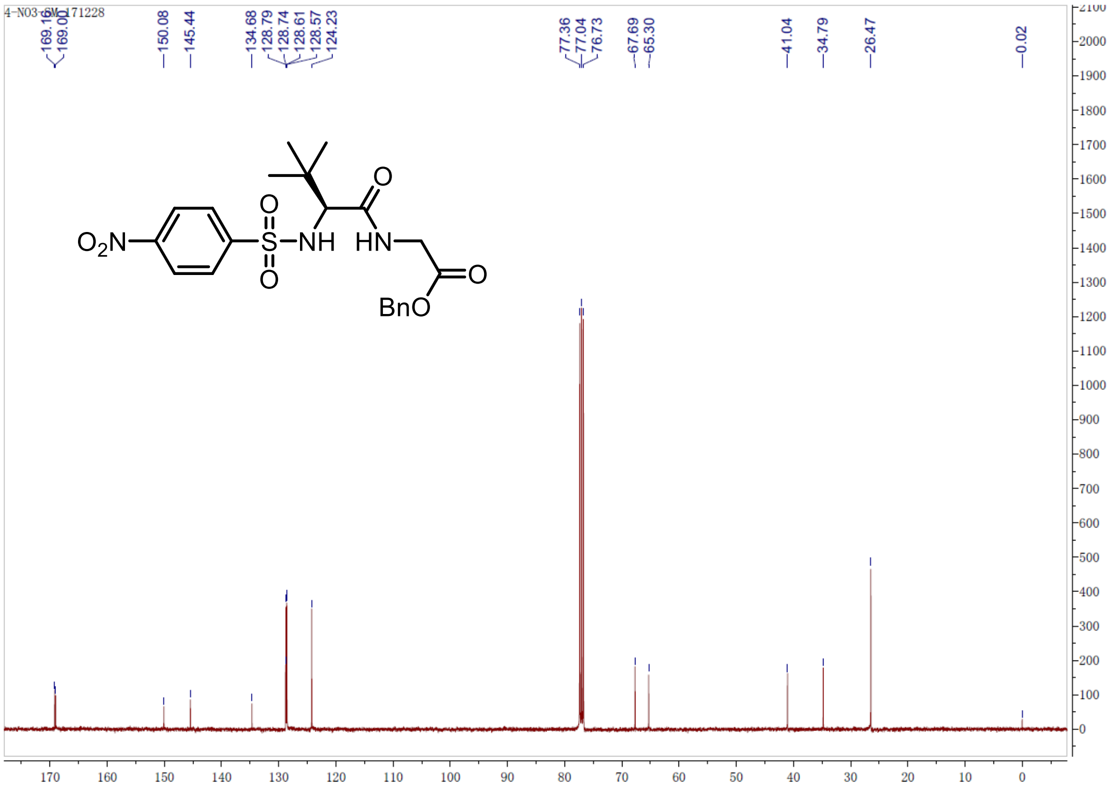

Supplementary Figure 142. ^{13}C NMR (100 MHz, $CDCl_3$) spectrum of compound **4a**

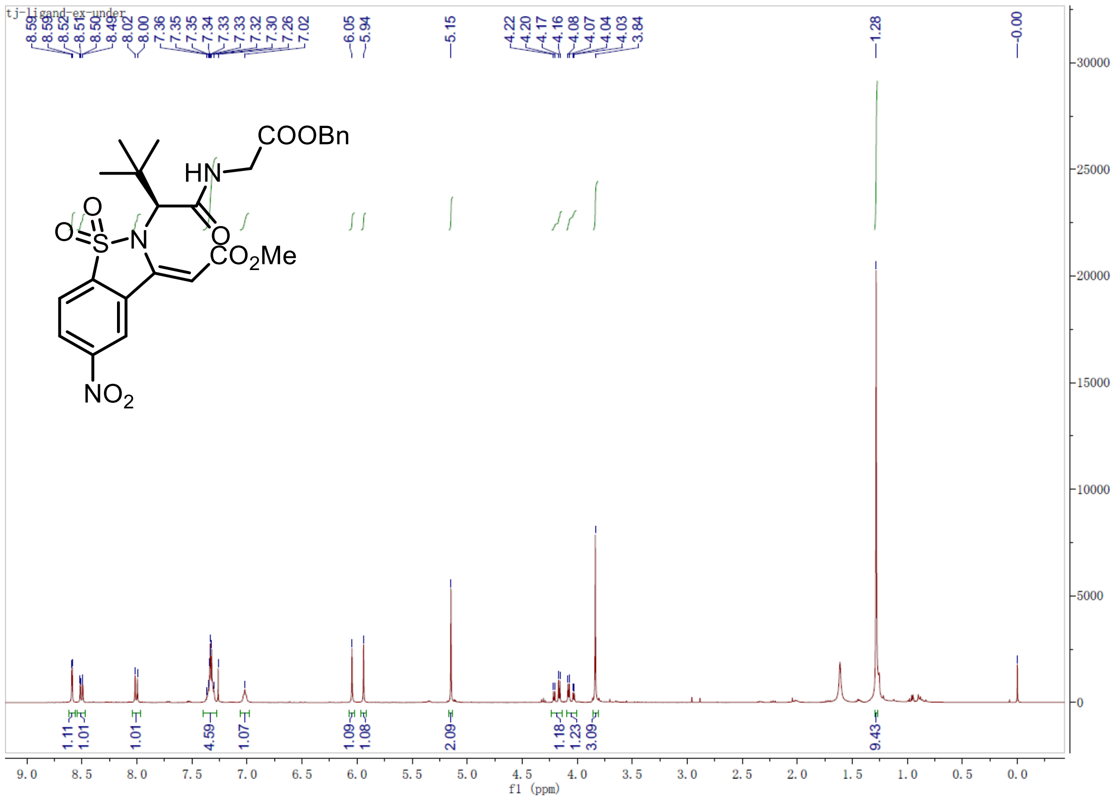

Supplementary Figure 143. ^{1}H NMR (400 MHz, $CDCl_3$) spectrum of compound **5ak**

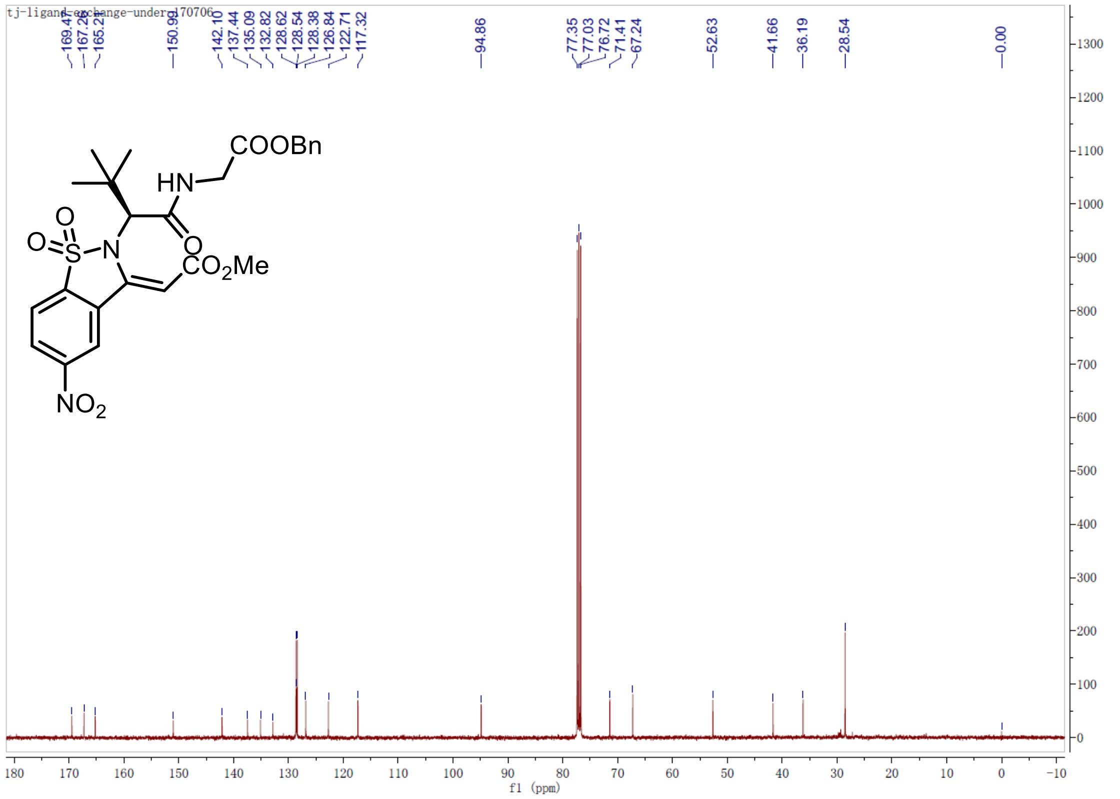

Supplementary Figure 144. ^{13}C NMR (100 MHz, $CDCl_3$) spectrum of compound **5ak**

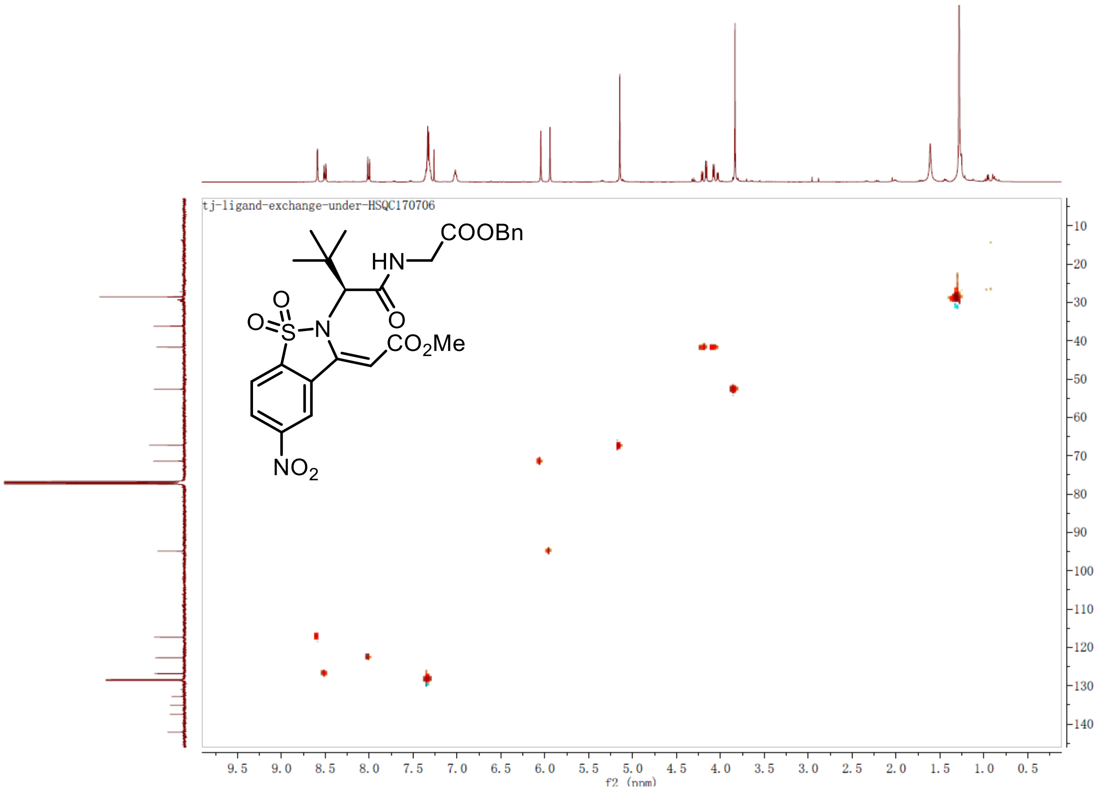

Supplementary Figure 145. HSQC of compound **5ak**

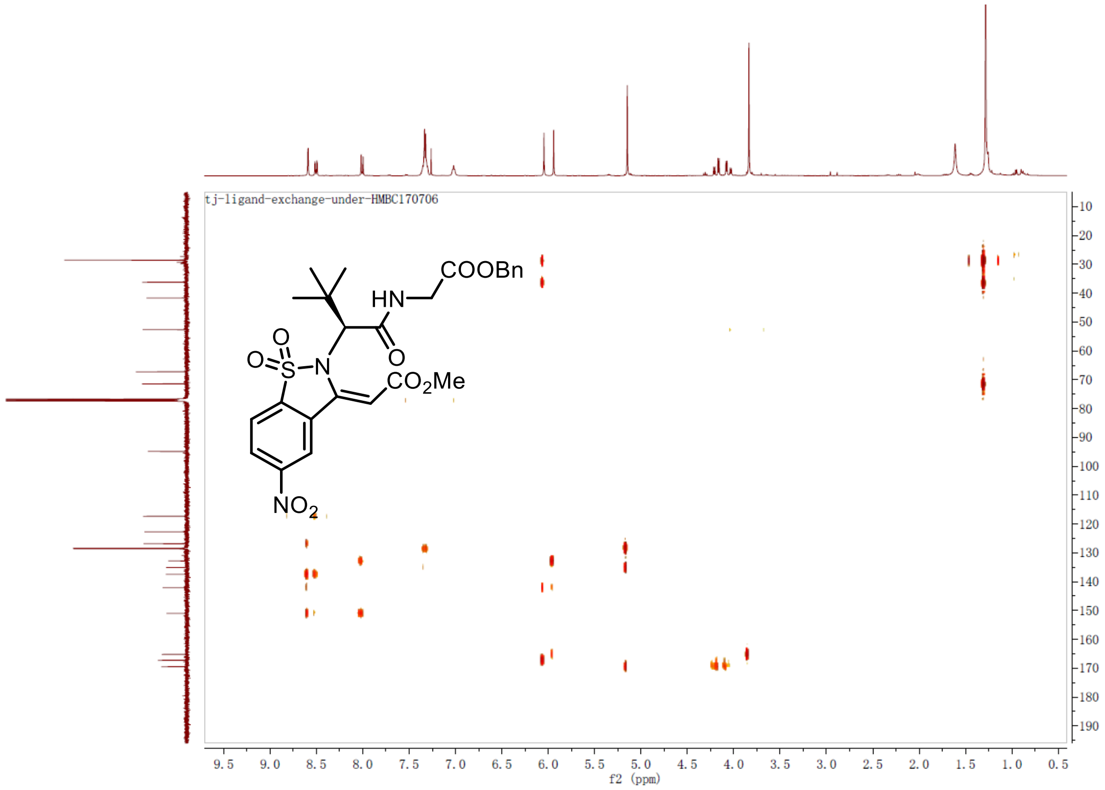

Supplementary Figure 146. HMBC of compound **5ak**

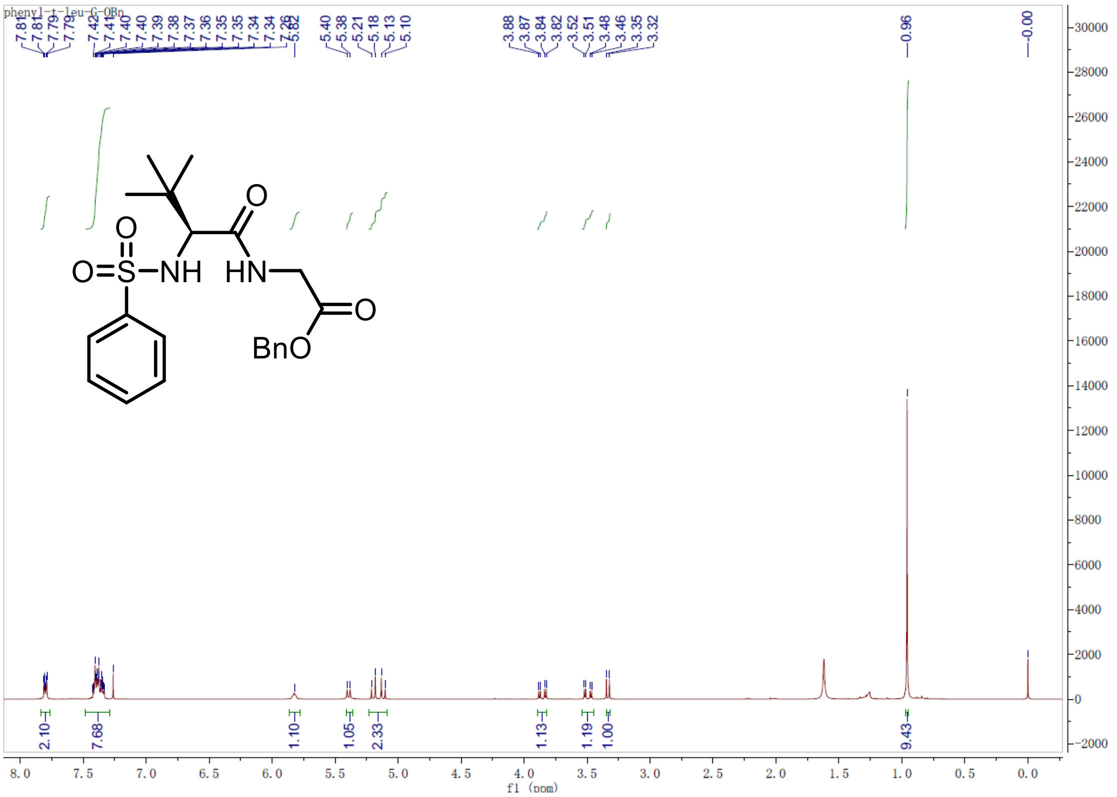

Supplementary Figure 147. ^{1}H NMR (400 MHz, $CDCl_3$) spectrum of compound **4b**

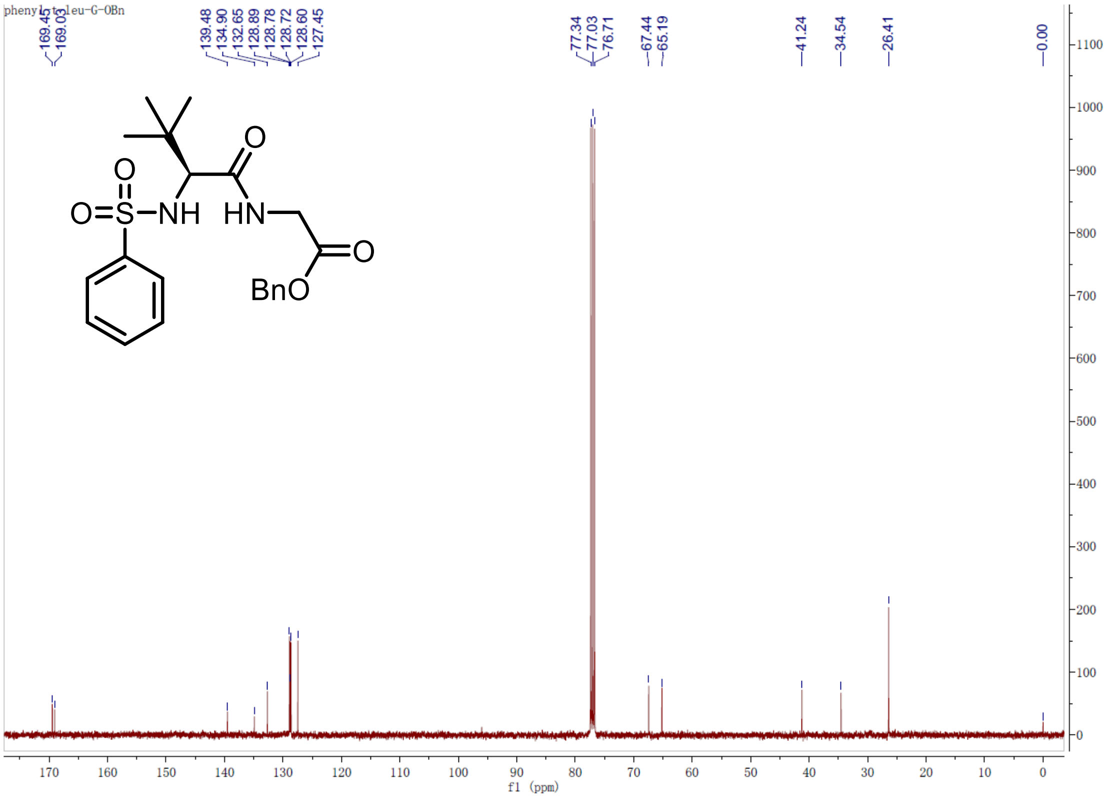

Supplementary Figure 148. ^{13}C NMR (100 MHz, $CDCl_3$) spectrum of compound **4b**

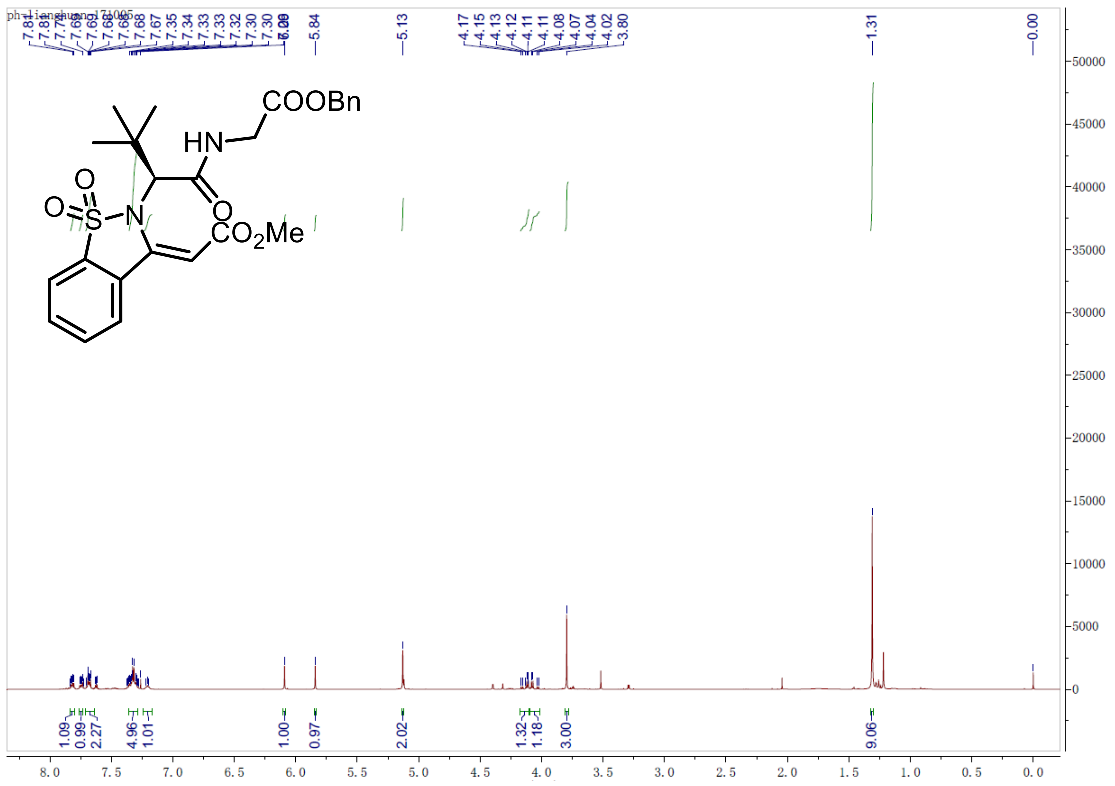

Supplementary Figure 149. ^{1}H NMR (400 MHz, $CDCl_3$) spectrum of compound **5bk**

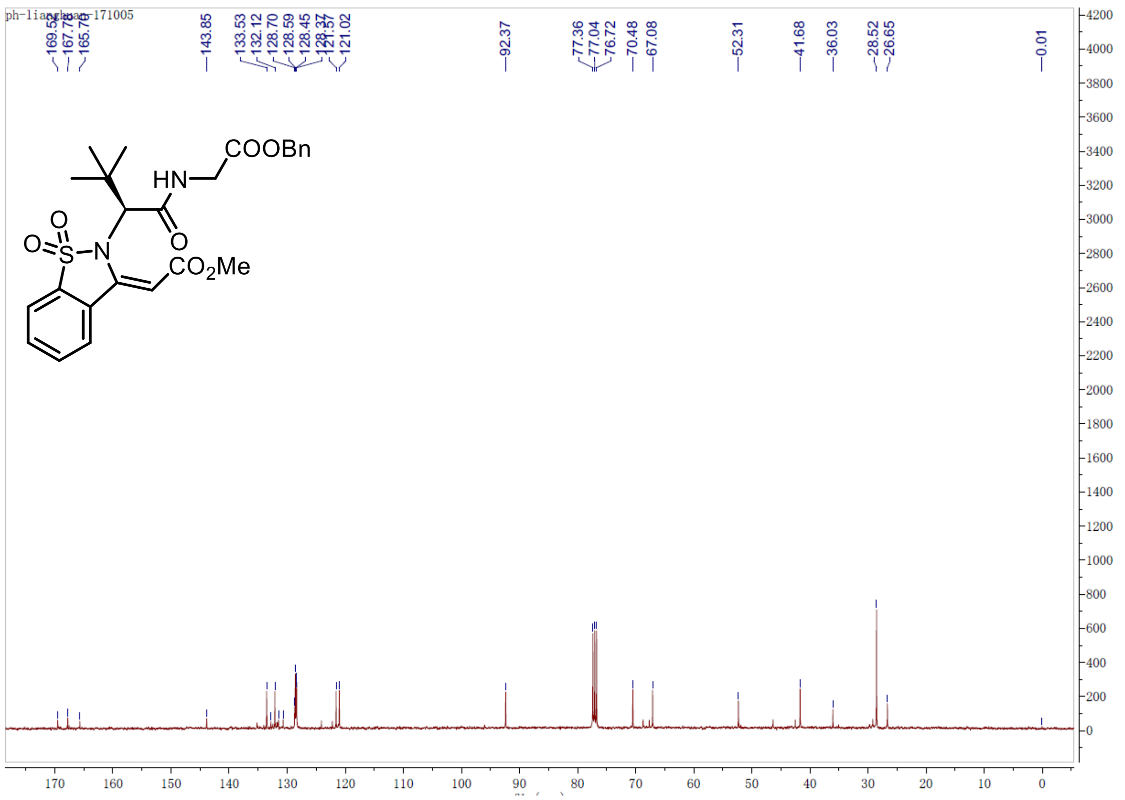

Supplementary Figure 150. ^{13}C NMR (100 MHz, $CDCl_3$) spectrum of compound **5bk**

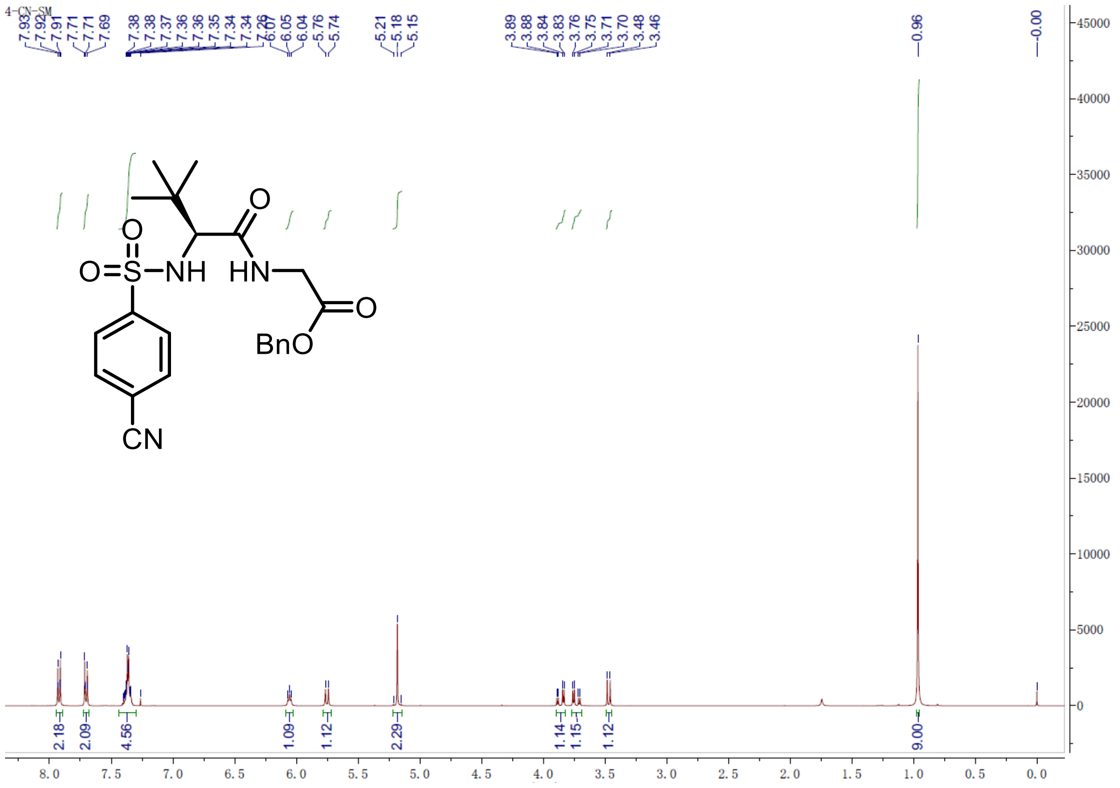

Supplementary Figure 151. ^{1}H NMR (400 MHz, $CDCl_3$) spectrum of compound **4c**

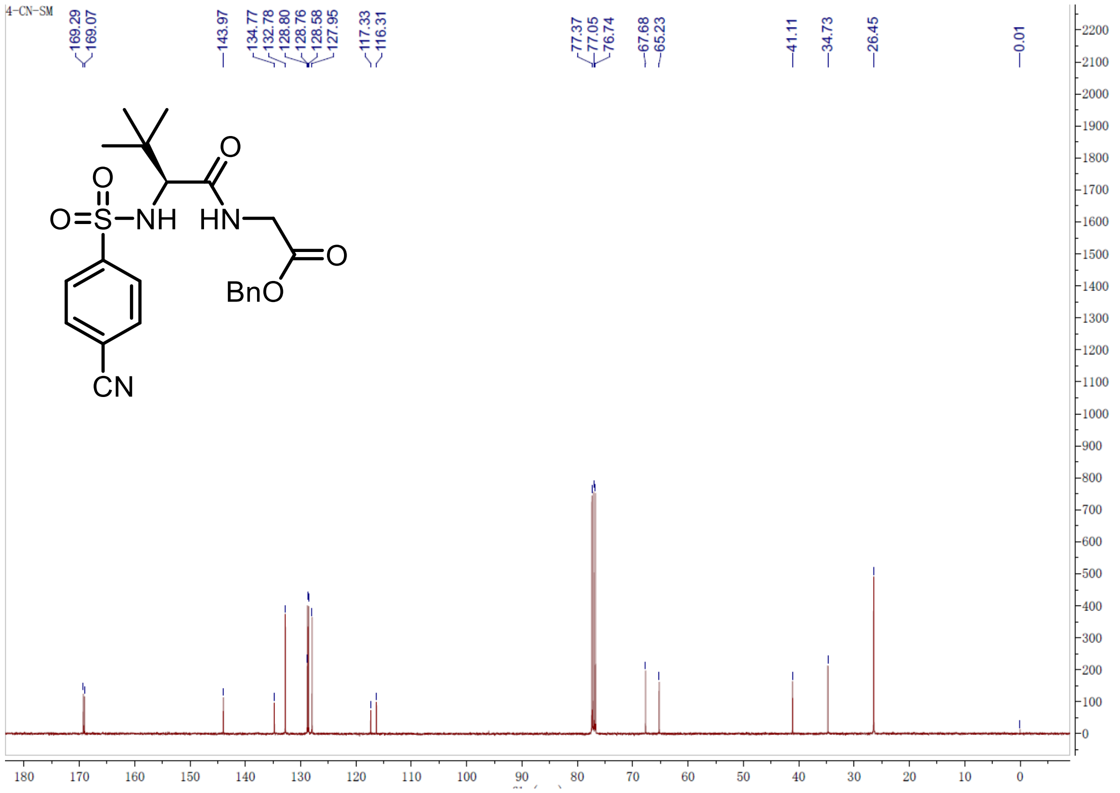

Supplementary Figure 152. ^{13}C NMR (100 MHz, $CDCl_3$) spectrum of compound **4c**

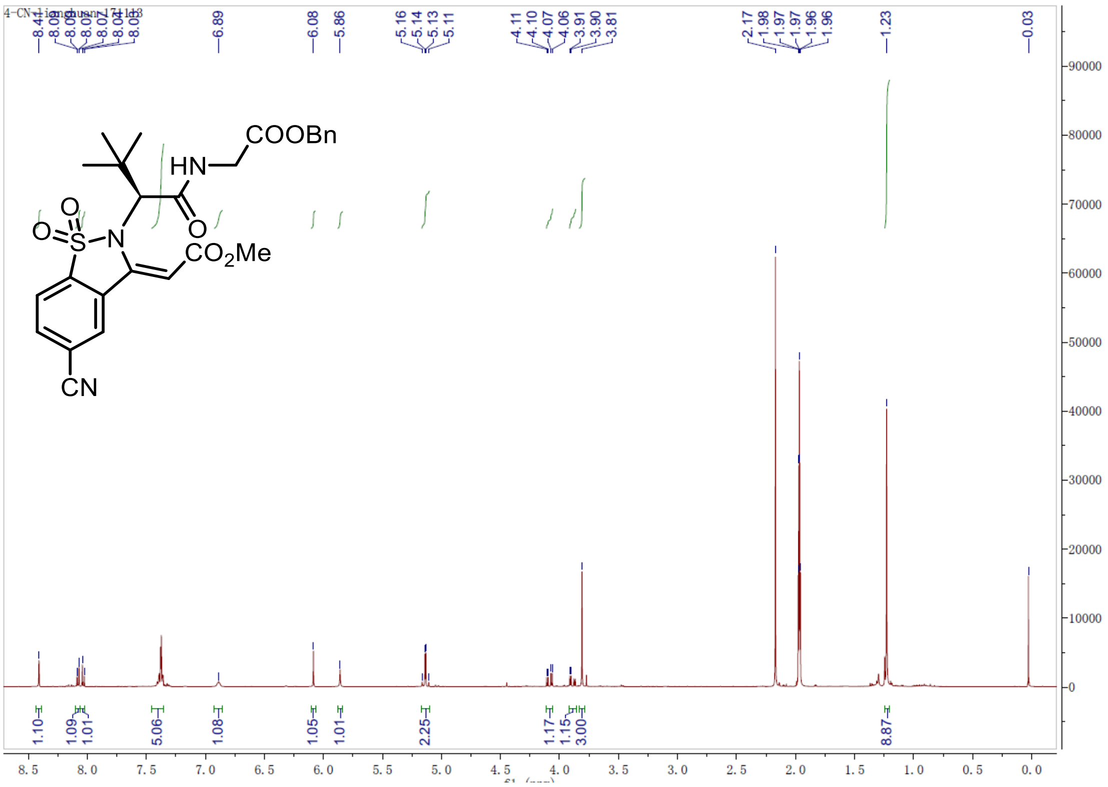

Supplementary Figure 153. ^{1}H NMR (400 MHz, CD_3CN) spectrum of compound **5ck**

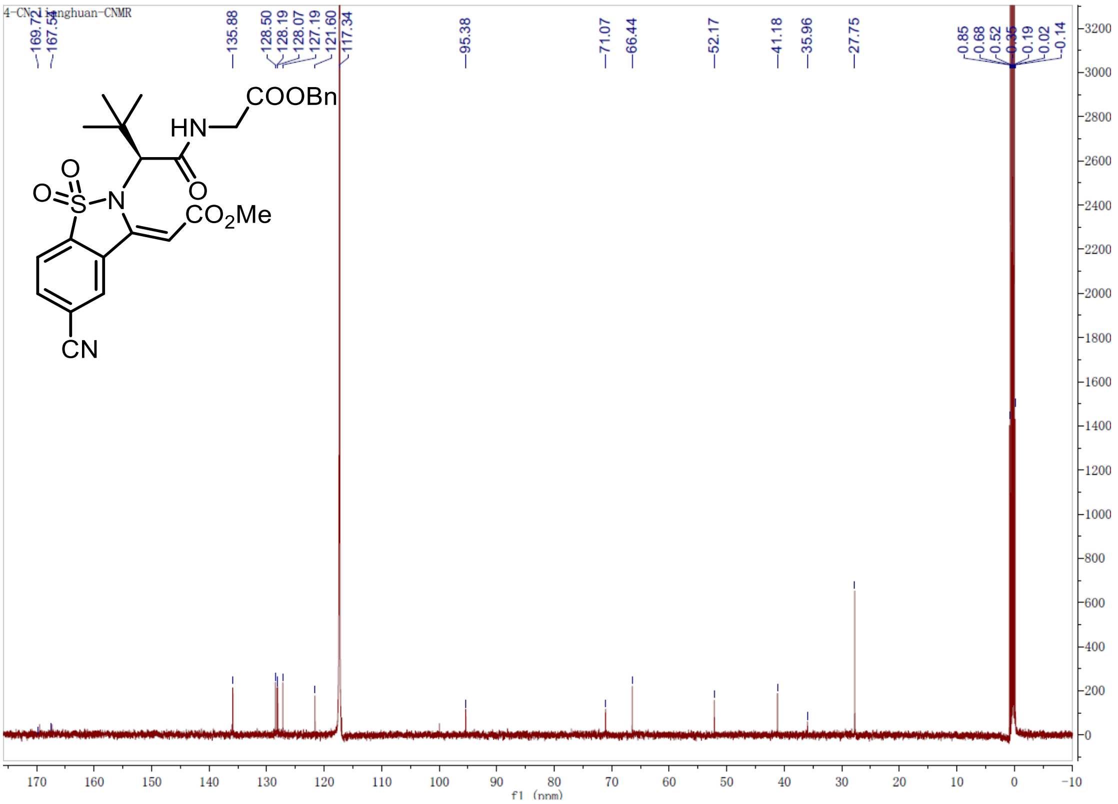

Supplementary Figure 154. ^{13}C NMR (100 MHz, CD_3CN) spectrum of compound **5ck**

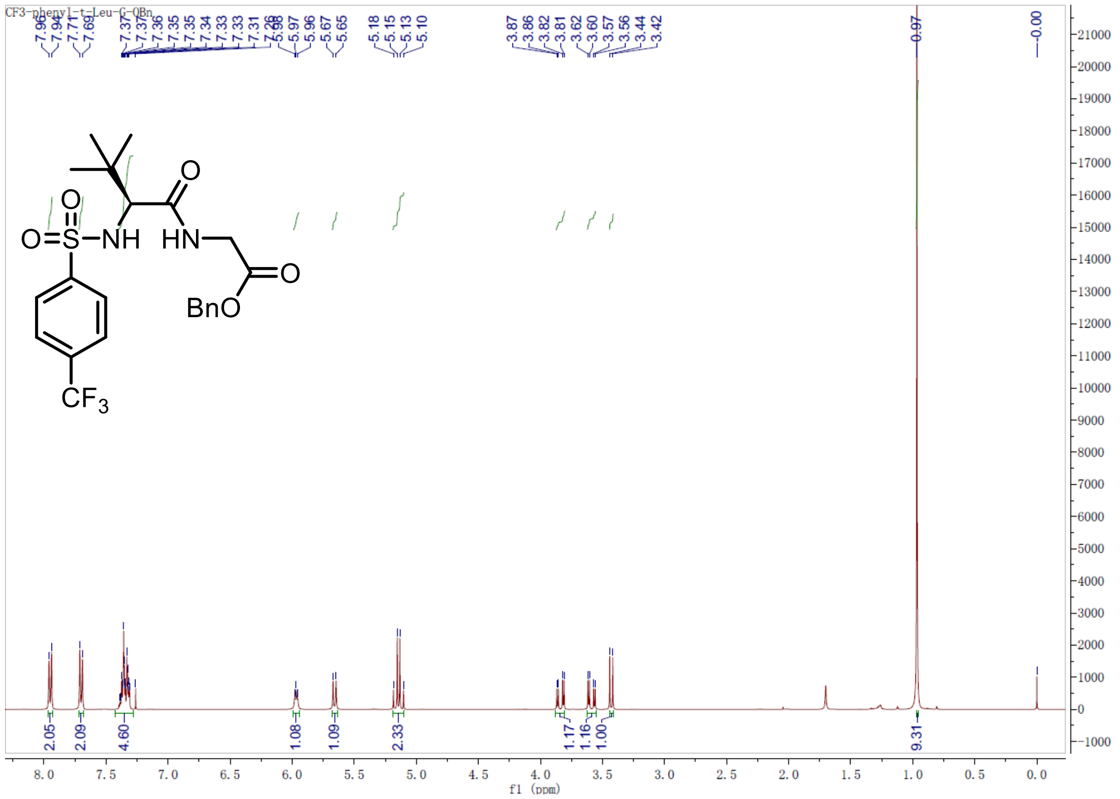

Supplementary Figure 155. ^{1}H NMR (400 MHz, $CDCl_3$) spectrum of compound **4d**

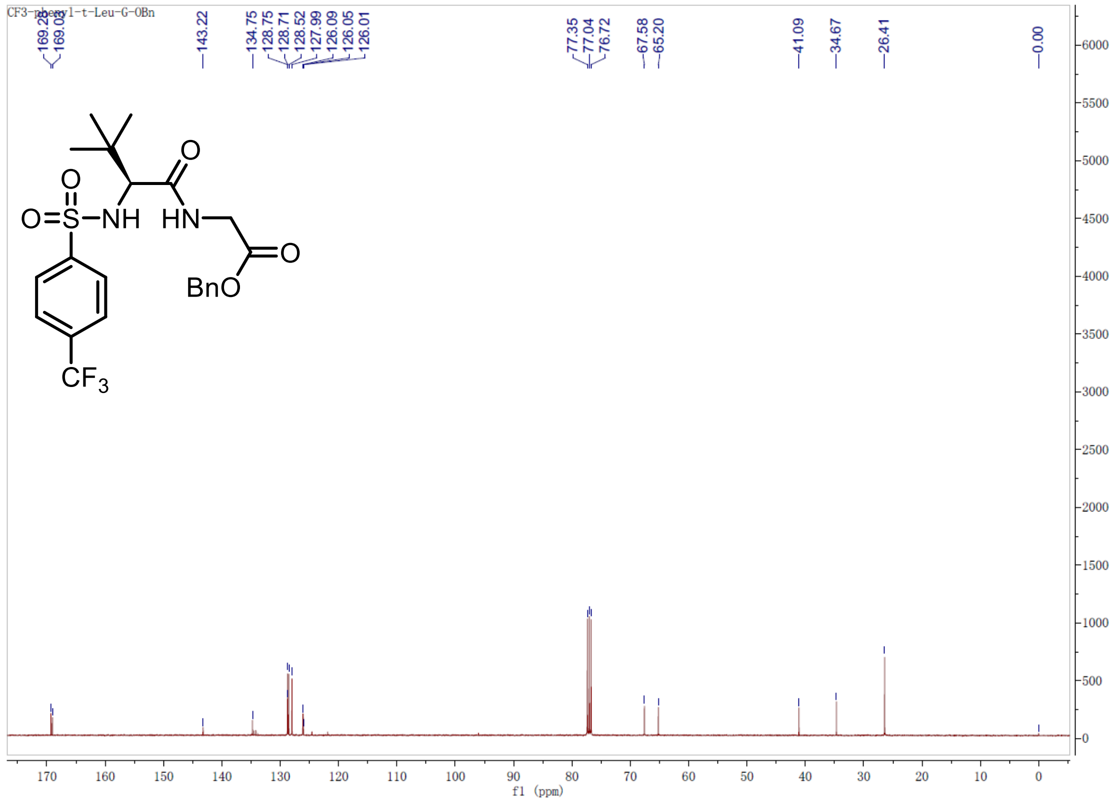

Supplementary Figure 156. ^{13}C NMR (100 MHz, $CDCl_3$) spectrum of compound **4d**

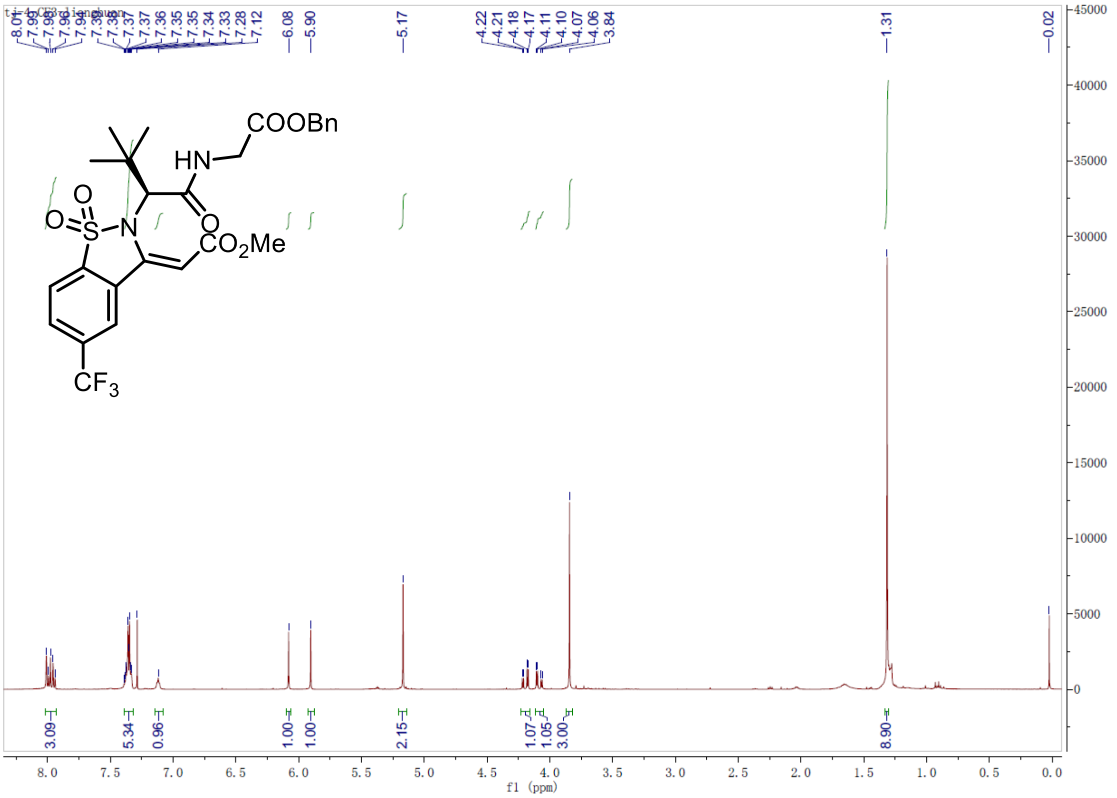

Supplementary Figure 157. ^{1}H NMR (400 MHz, $CDCl_3$) spectrum of compound **5dk**

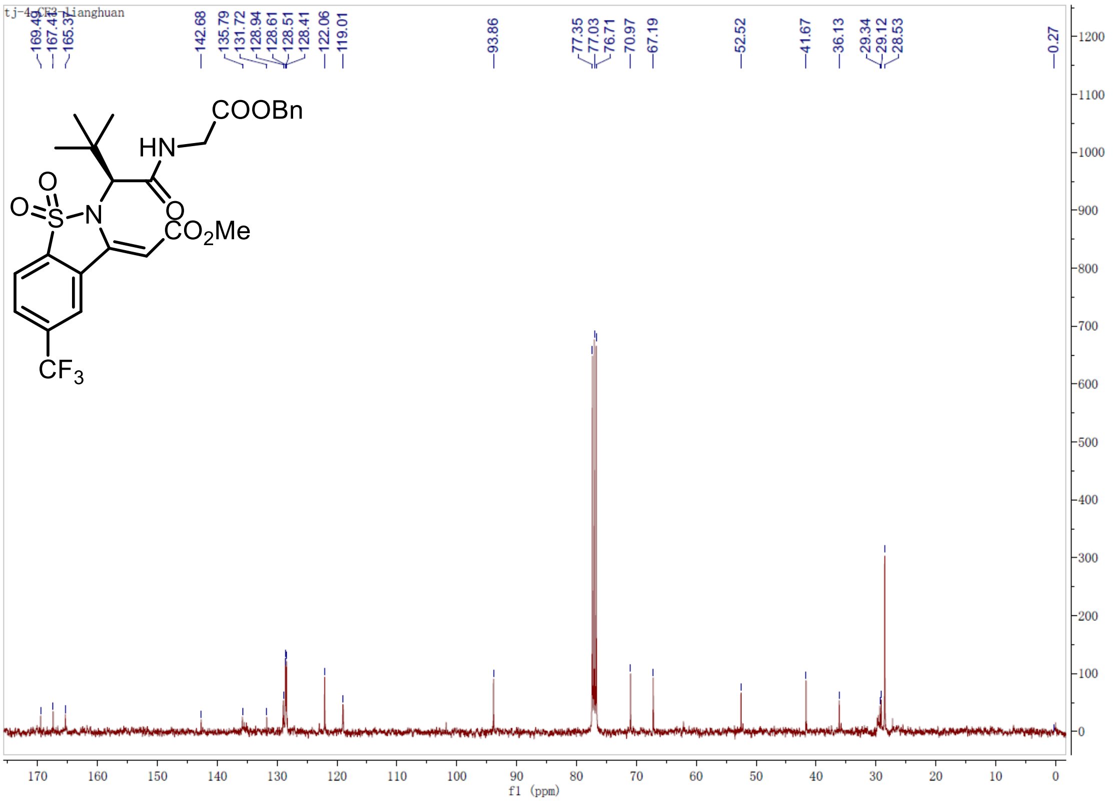

Supplementary Figure 158. ^{13}C NMR (100 MHz, $CDCl_3$) spectrum of compound **5dk**

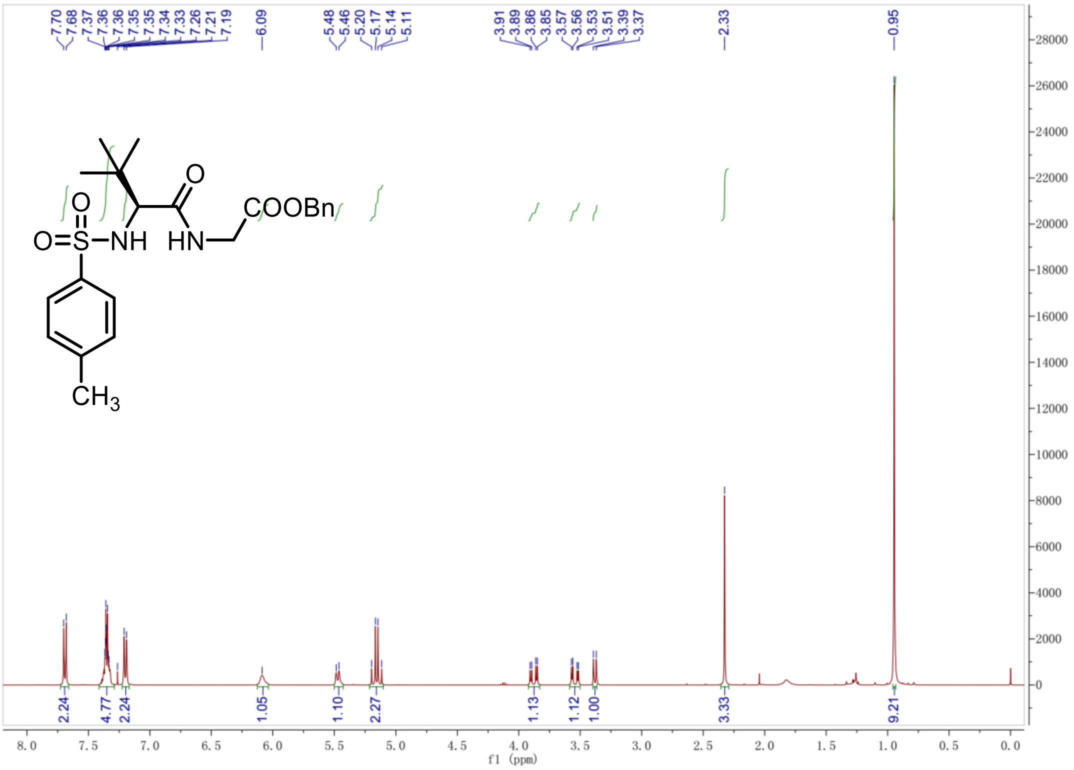

Supplementary Figure 159. ^{1}H NMR (400 MHz, $CDCl_3$) spectrum of compound **4e**

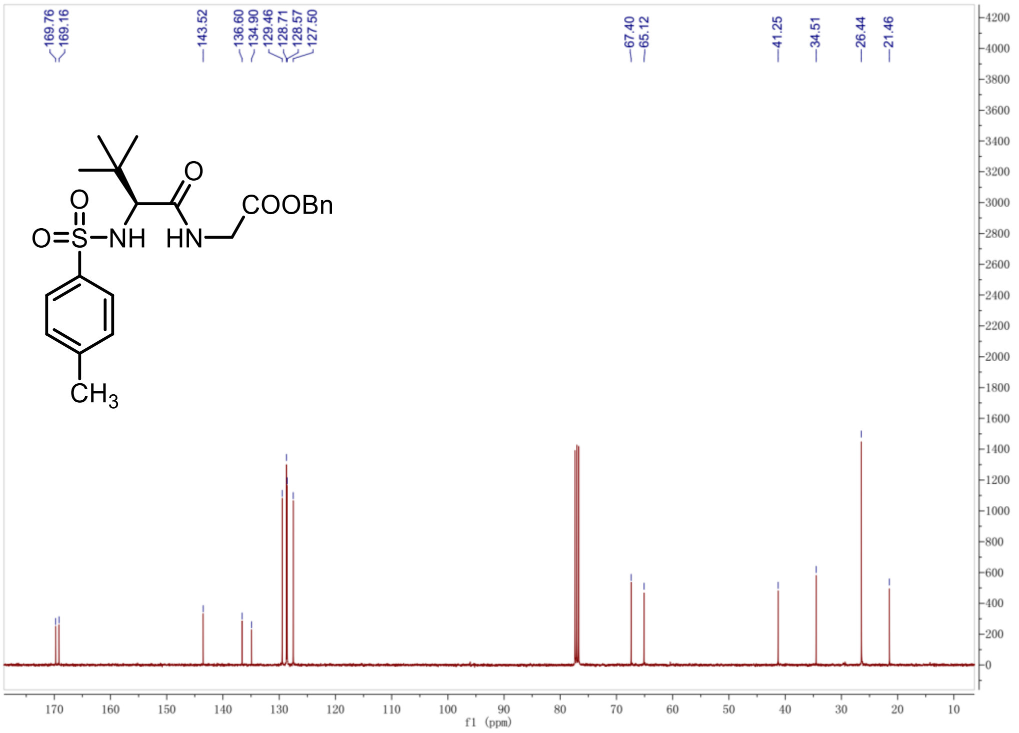

Supplementary Figure 160. ^{13}C NMR (100 MHz, $CDCl_3$) spectrum of compound **4e**

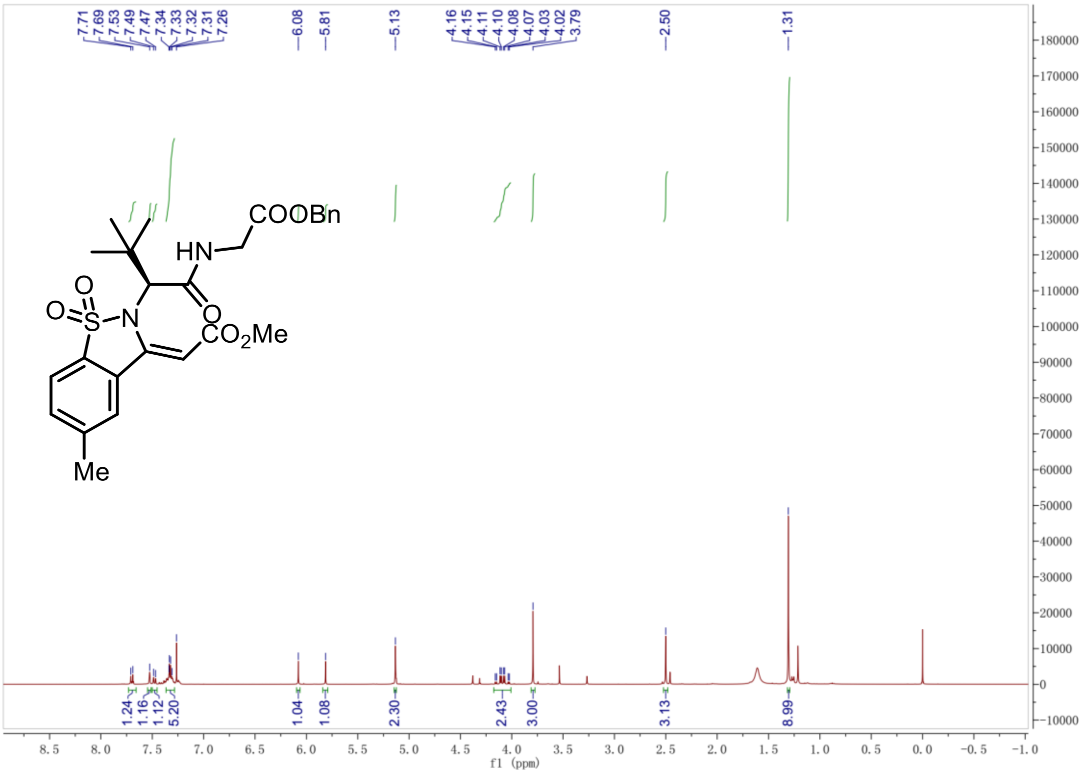

Supplementary Figure 161. ^{1}H NMR (400 MHz, $CDCl_3$) spectrum of compound **5ea**

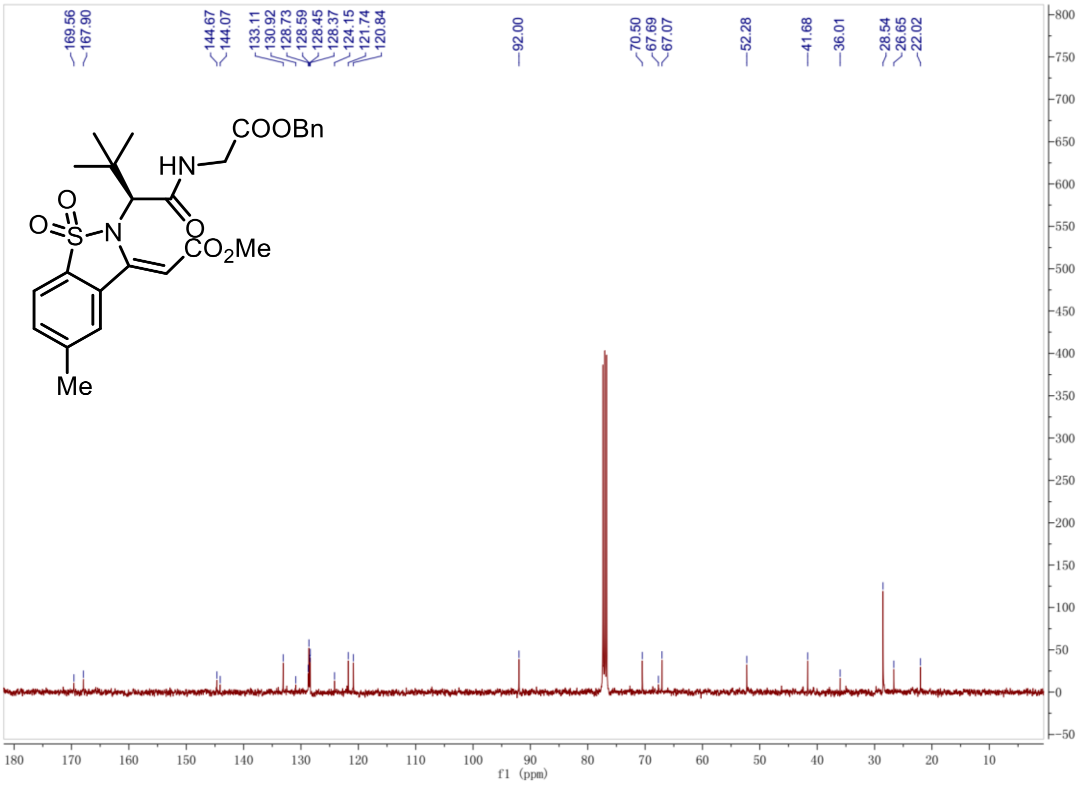

Supplementary Figure 162. ^{13}C NMR (100 MHz, $CDCl_3$) spectrum of compound **5ea**

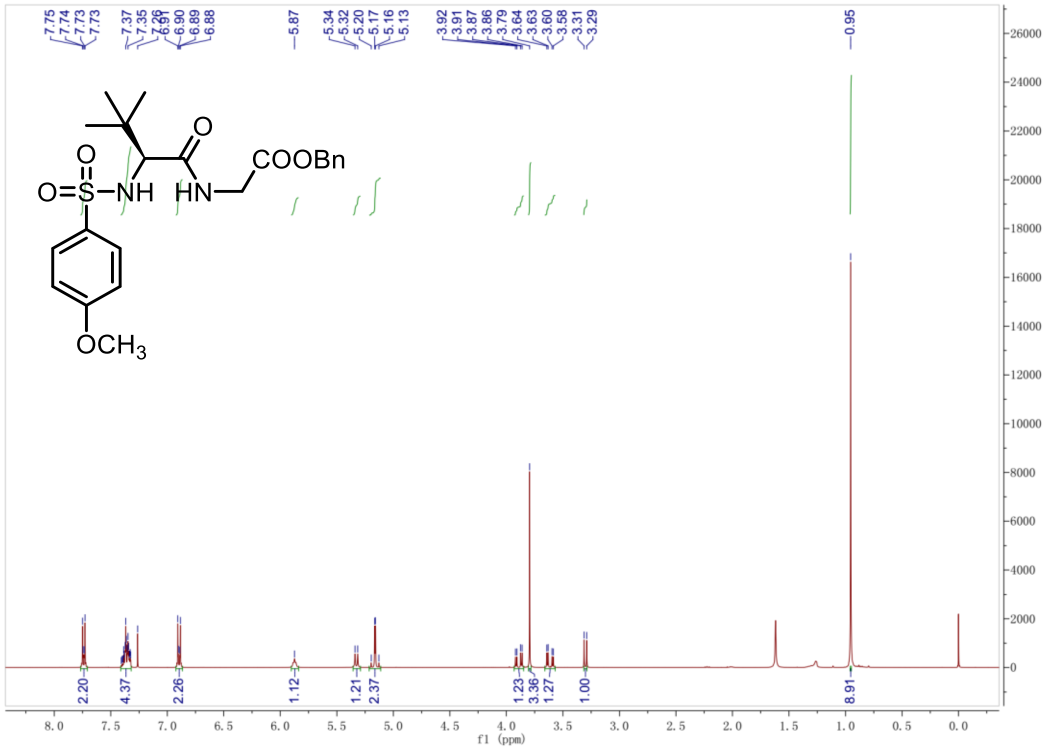

Supplementary Figure 163. ^{1}H NMR (400 MHz, $CDCl_3$) spectrum of compound **4f**

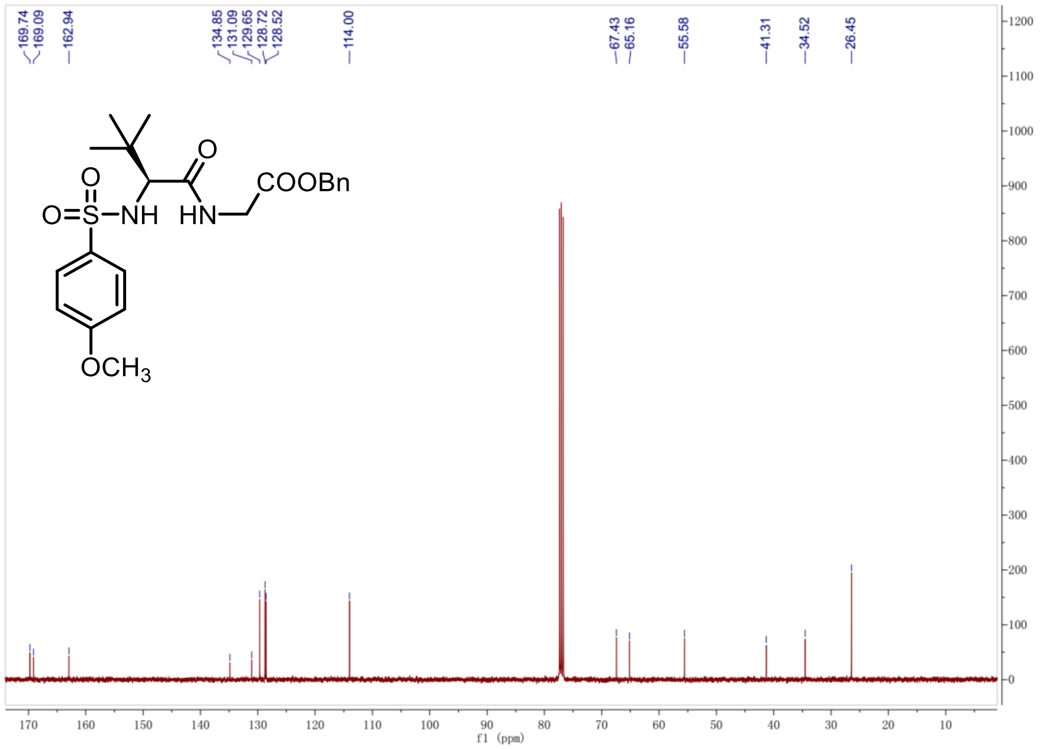

Supplementary Figure 164. ^{13}C NMR (100 MHz, $CDCl_3$) spectrum of compound **4f**

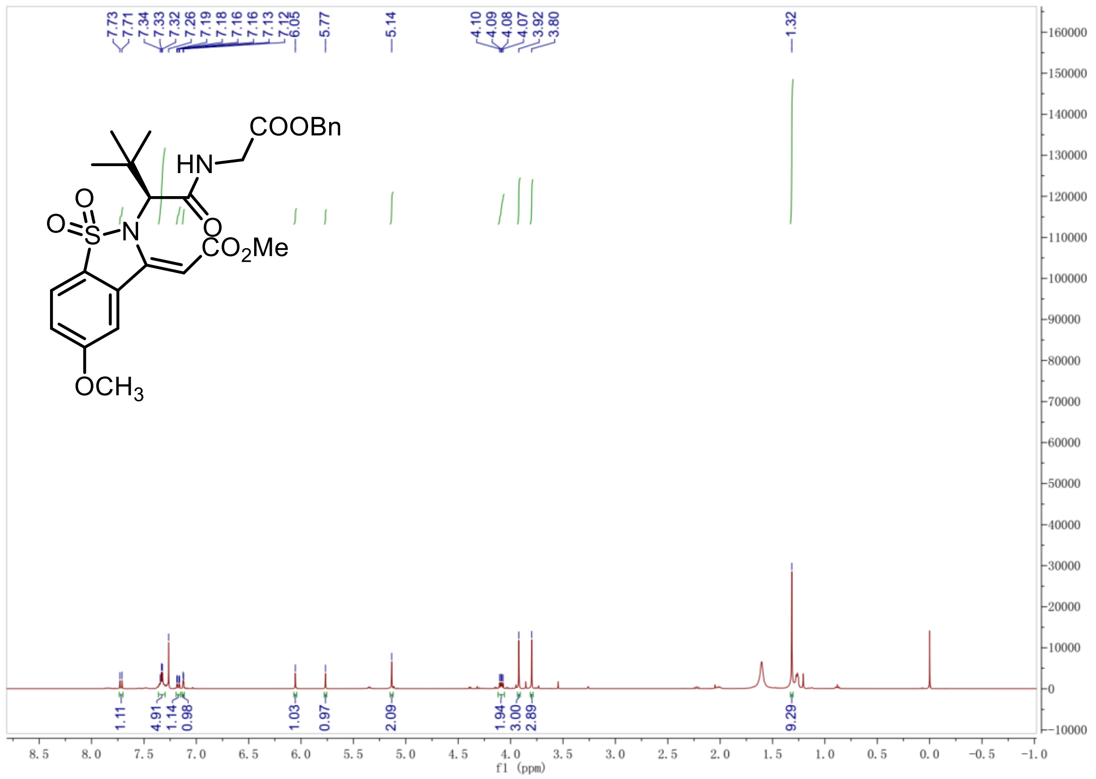

Supplementary Figure 165. ^{1}H NMR (400 MHz, $CDCl_3$) spectrum of compound **5fa**

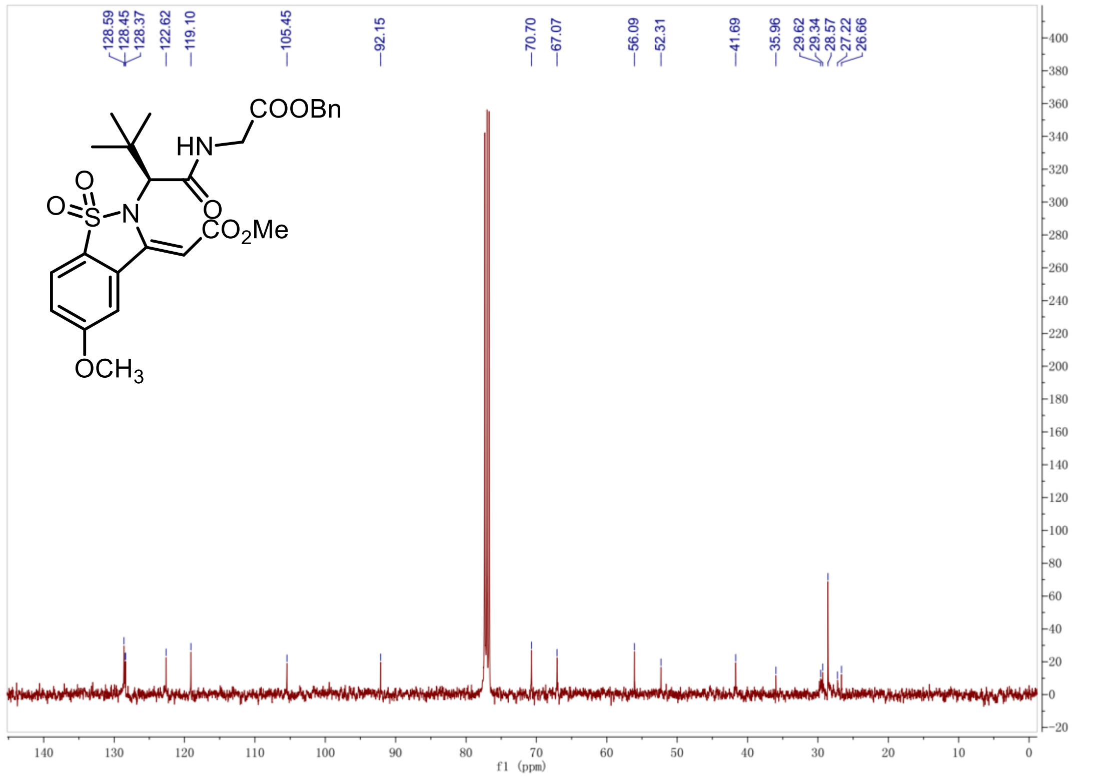

Supplementary Figure 166. ^{13}C NMR (100 MHz, $CDCl_3$) spectrum of compound **5fa**

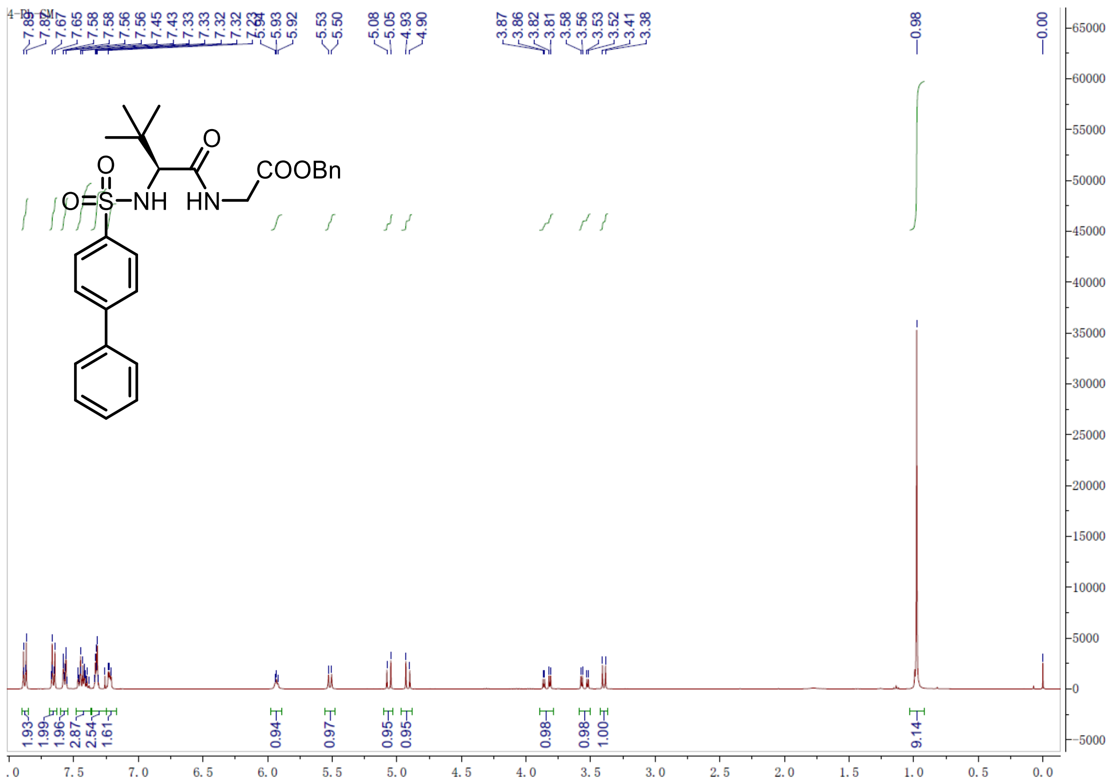

Supplementary Figure 167. ^{1}H NMR (400 MHz, $CDCl_3$) spectrum of compound **4g**

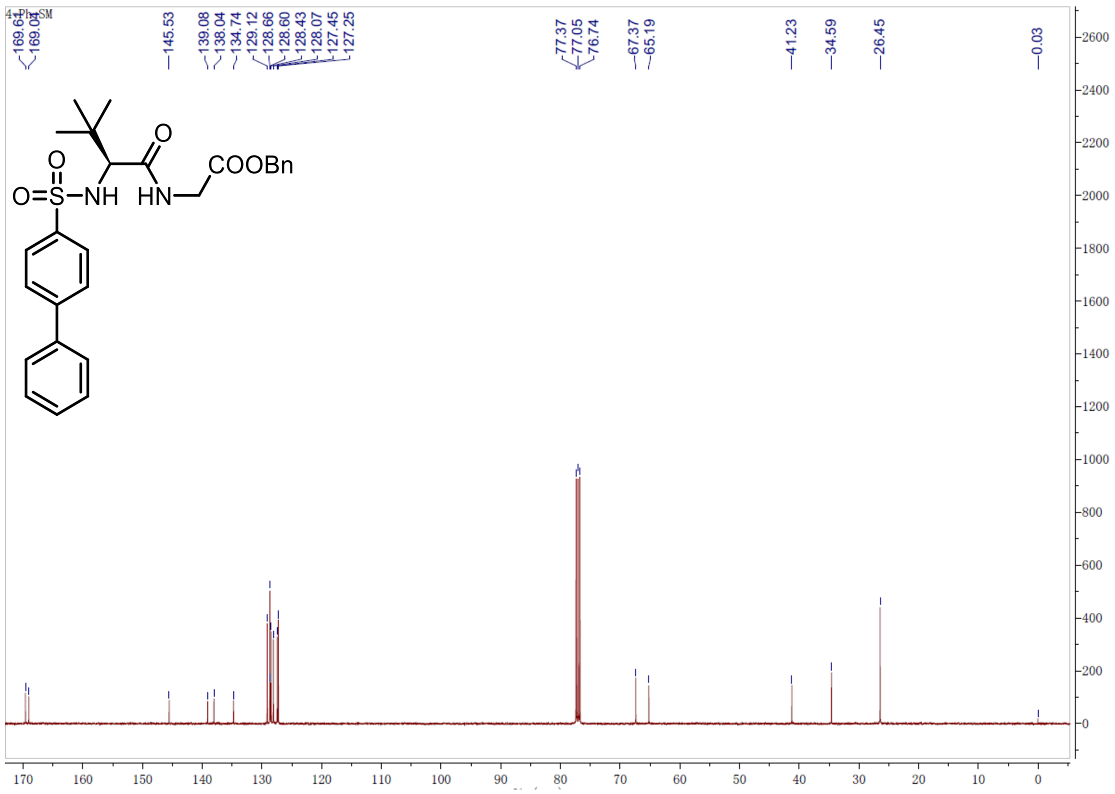

Supplementary Figure 168. ^{13}C NMR (100 MHz, $CDCl_3$) spectrum of compound **4g**

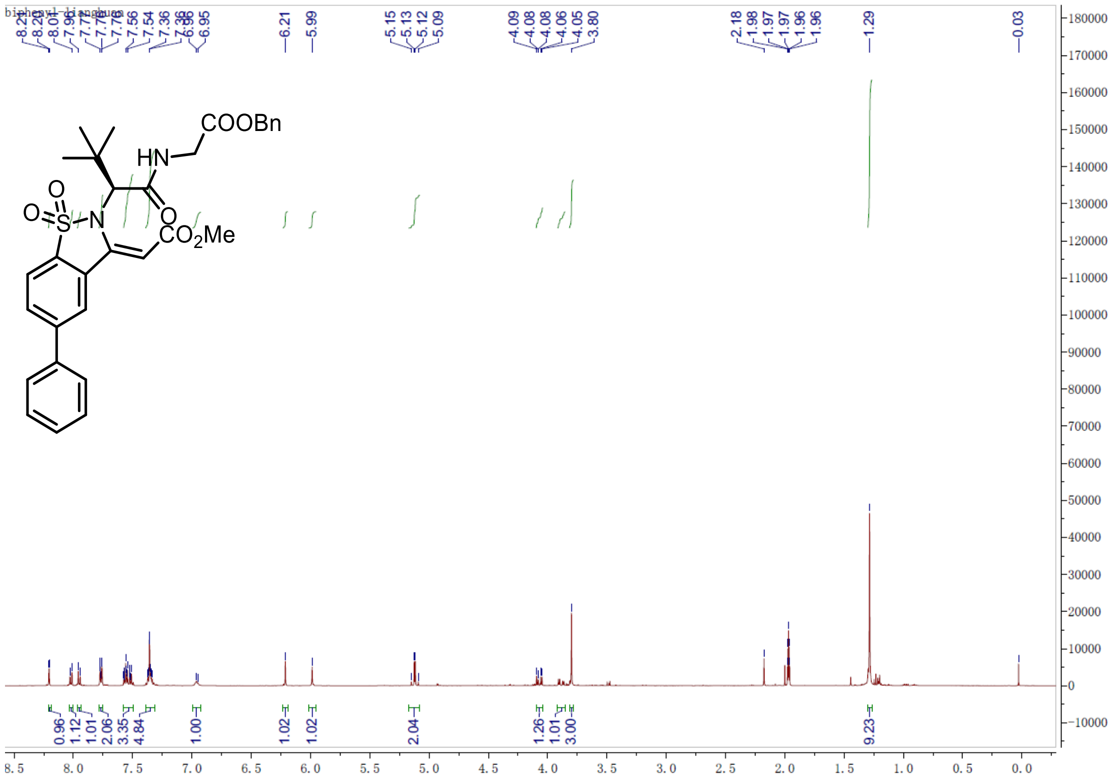

Supplementary Figure 169. ^{1}H NMR (400 MHz, CD_3CN) spectrum of compound **5gk**

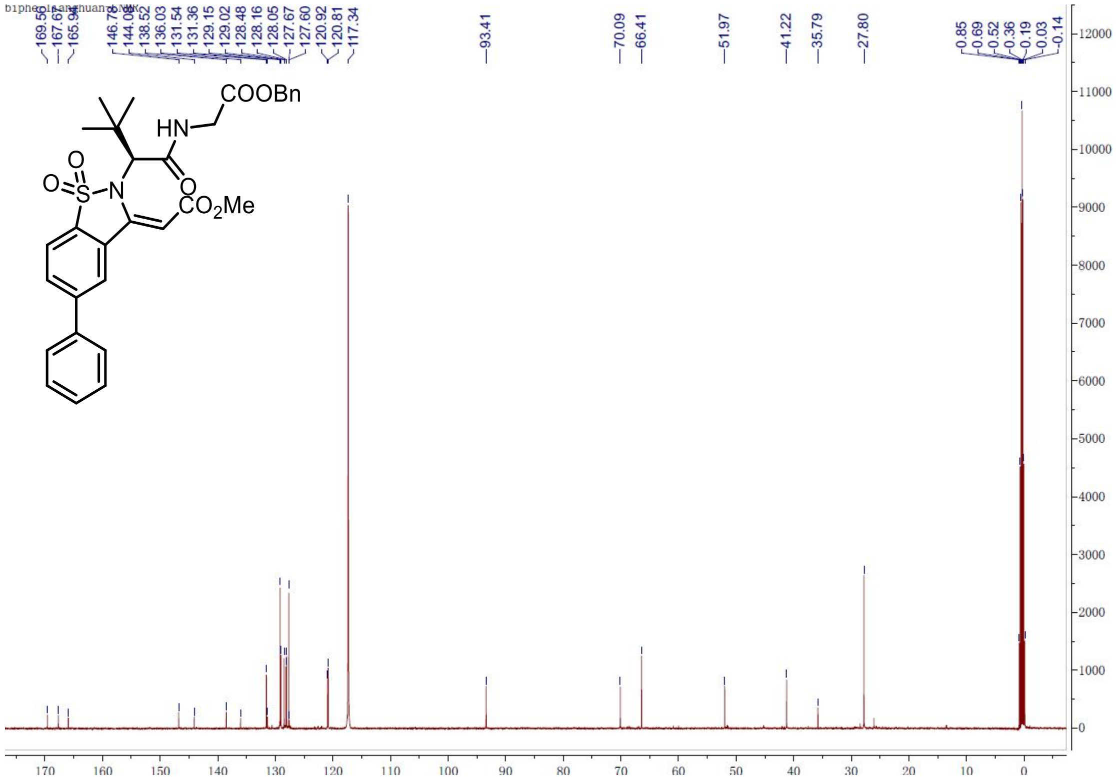

Supplementary Figure 170. ^{13}C NMR (100 MHz, CD_3CN) spectrum of compound **5gk**

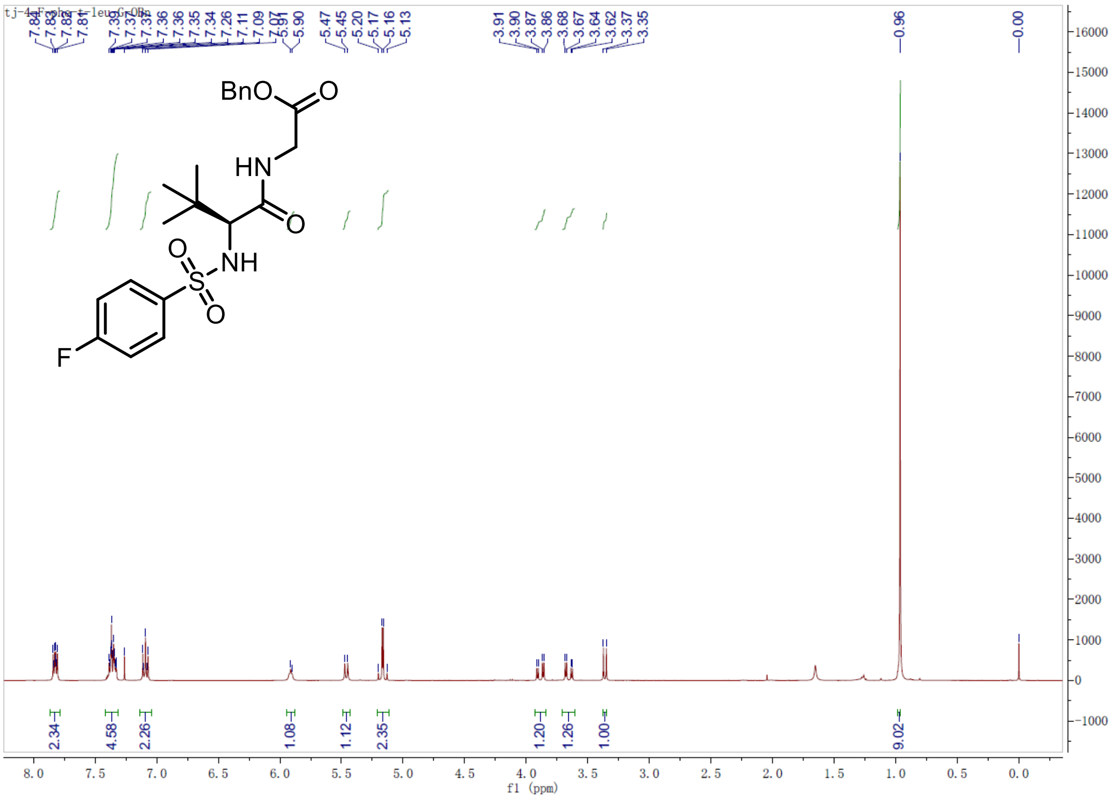

Supplementary Figure 171. ^{1}H NMR (400 MHz, $CDCl_3$) spectrum of compound **4h**

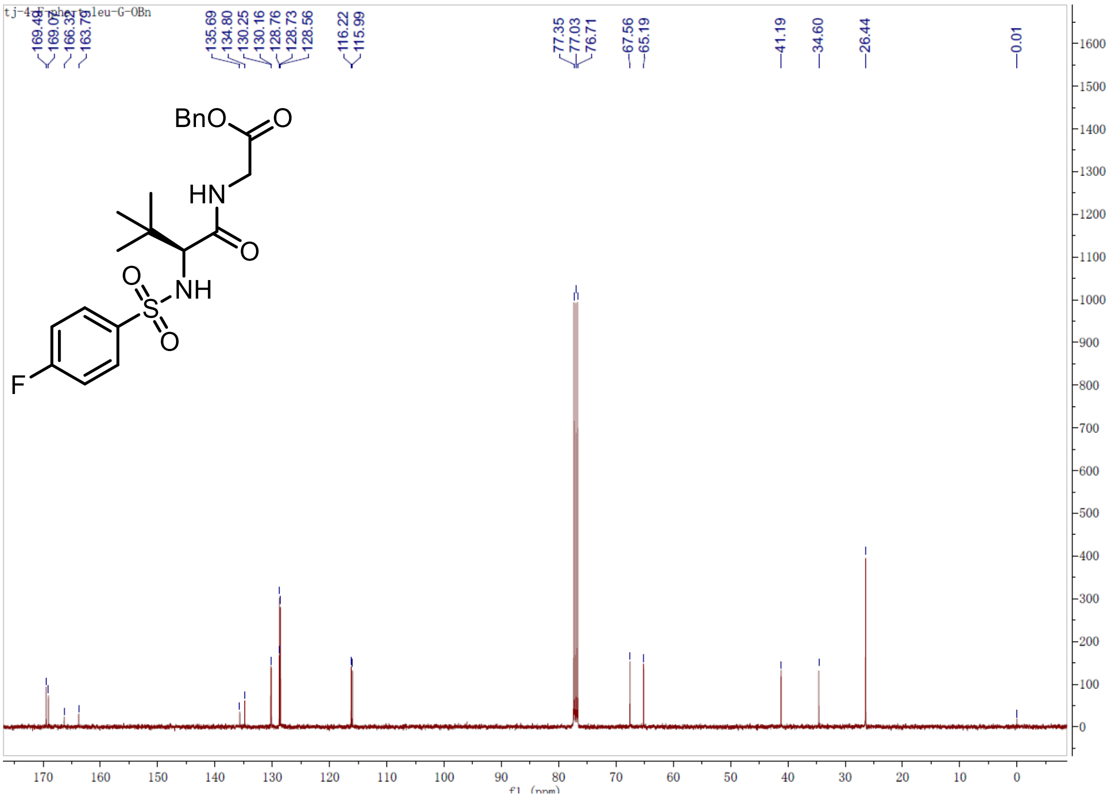

Supplementary Figure 172. ^{13}C NMR (100 MHz, $CDCl_3$) spectrum of compound **4h**

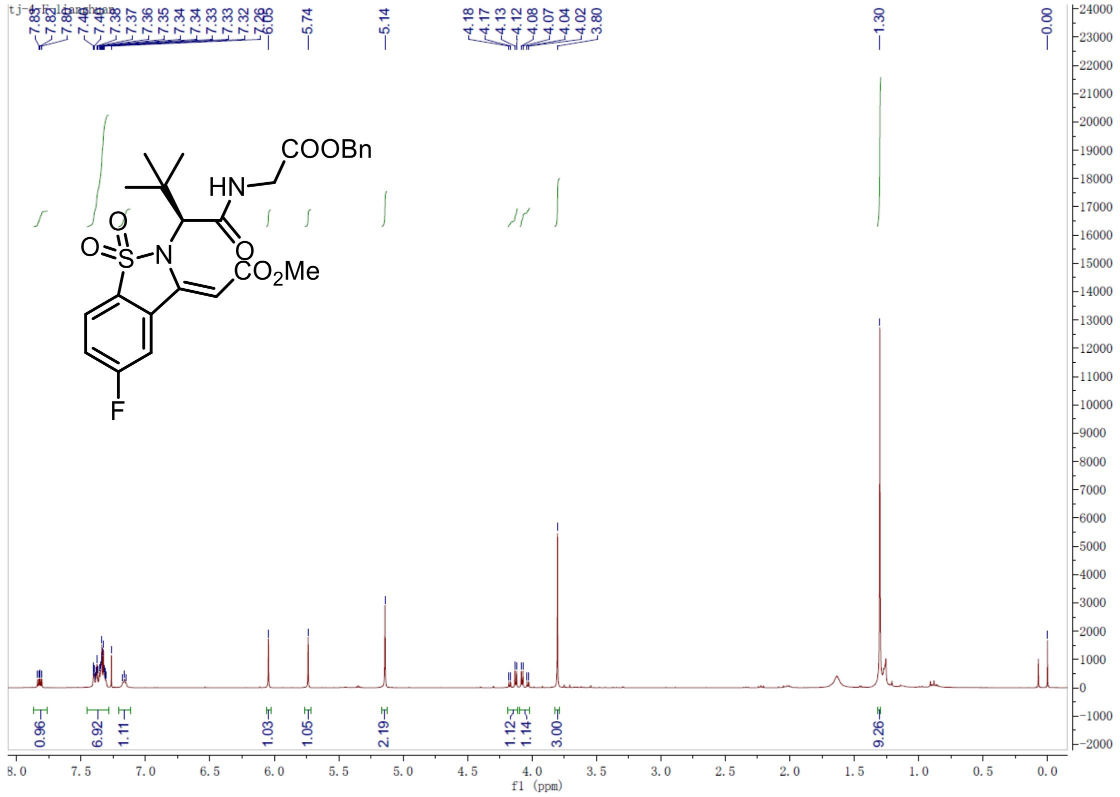

Supplementary Figure 173. ^{1}H NMR (400 MHz, $CDCl_3$) spectrum of compound **5hk**

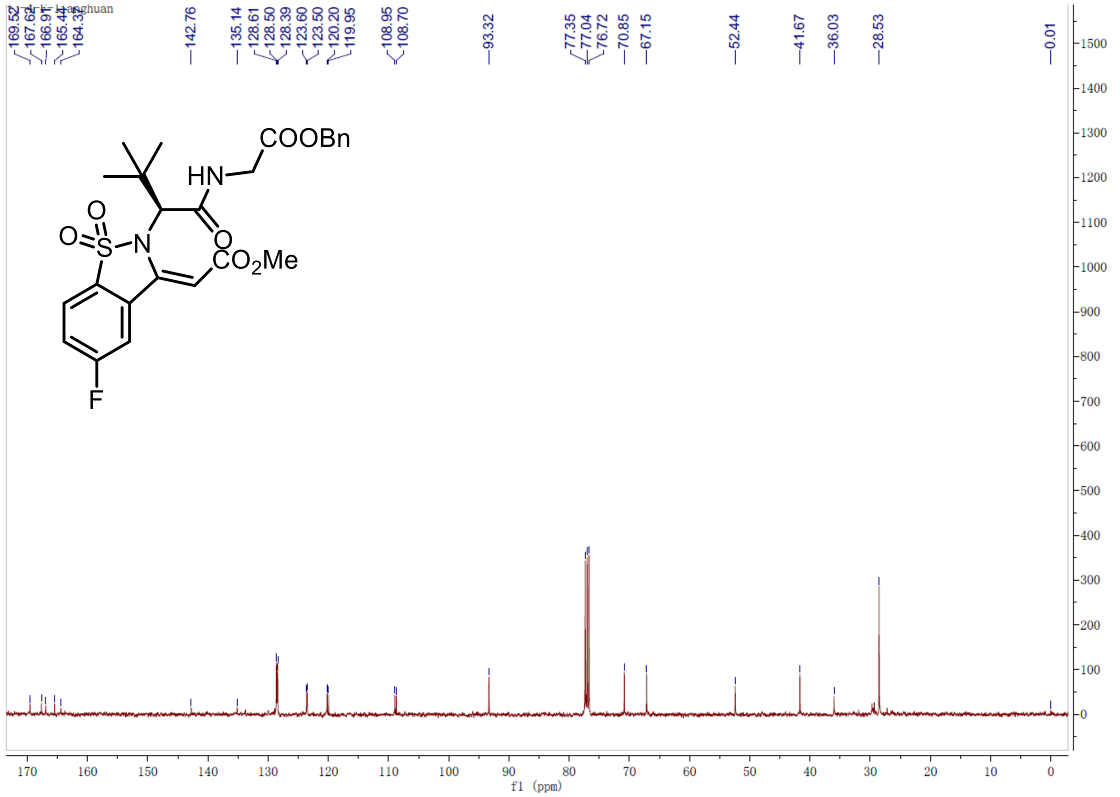

Supplementary Figure 174. ^{13}C NMR (100 MHz, $CDCl_3$) spectrum of compound **5hk**

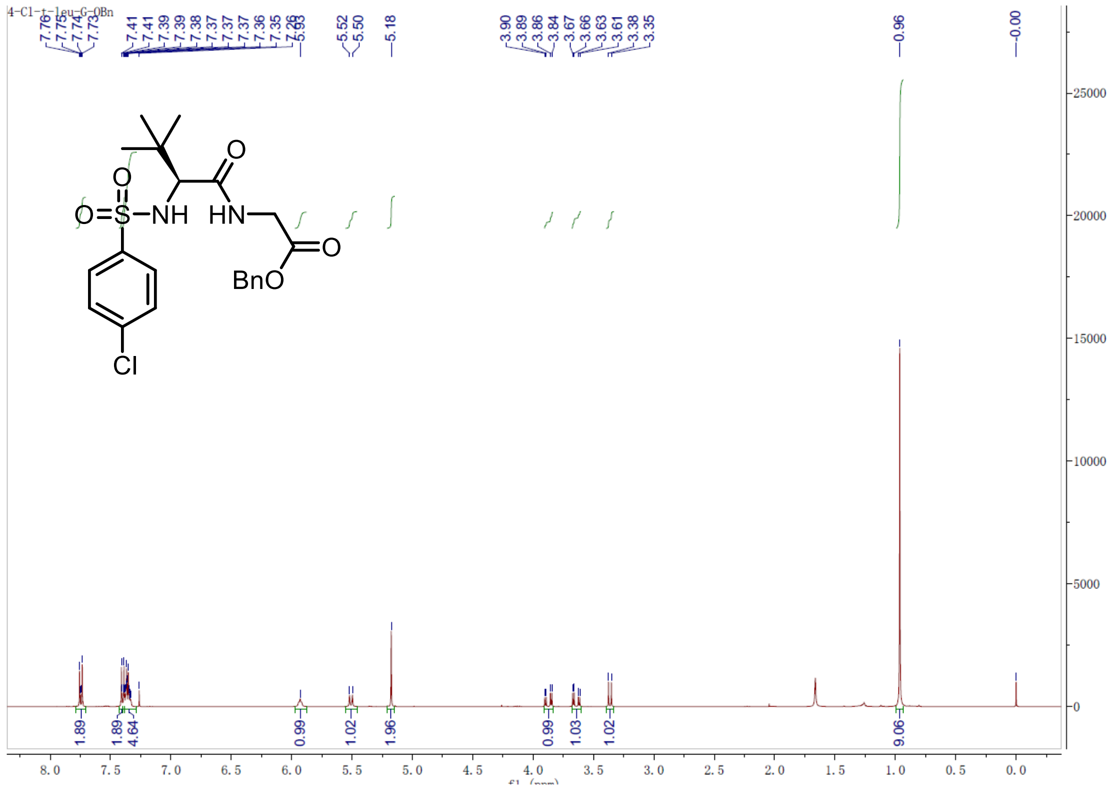

Supplementary Figure 175. ^{1}H NMR (400 MHz, $CDCl_3$) spectrum of compound **4i**

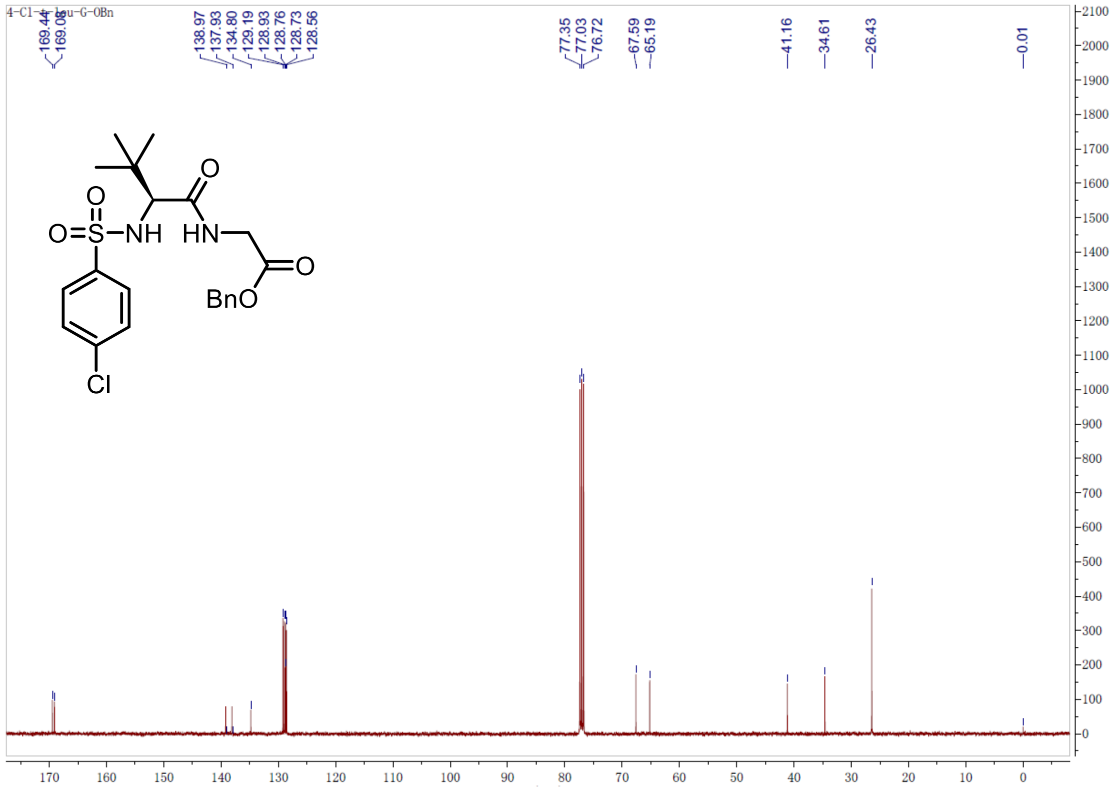

Supplementary Figure 176. ^{13}C NMR (100 MHz, $CDCl_3$) spectrum of compound **4i**

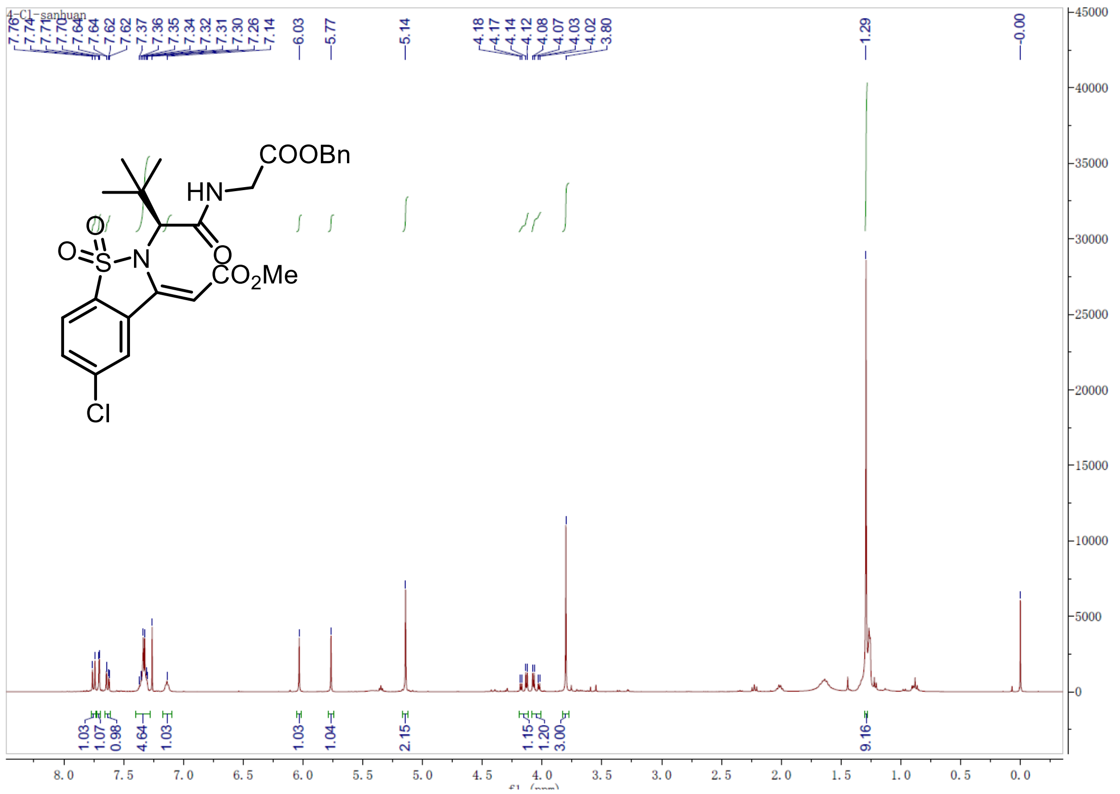

Supplementary Figure 177. ^{1}H NMR (400 MHz, $CDCl_3$) spectrum of compound **5ik**

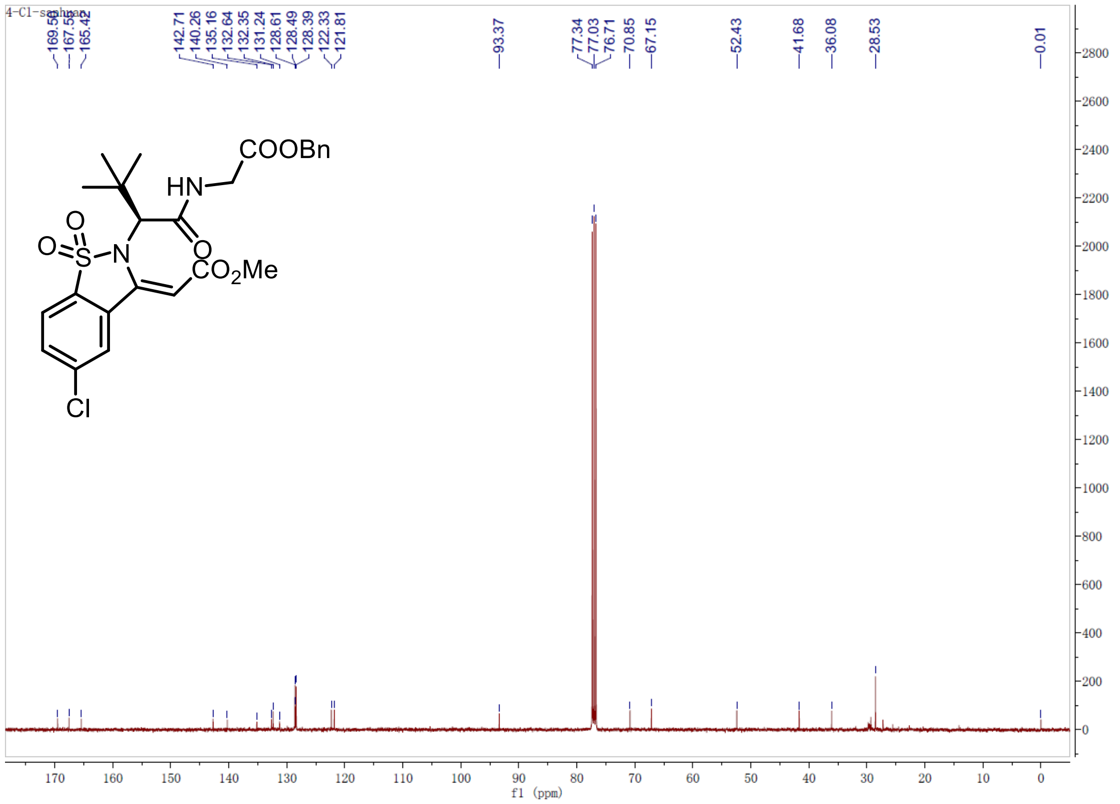

Supplementary Figure 178. ^{13}C NMR (100 MHz, $CDCl_3$) spectrum of compound **5ik**

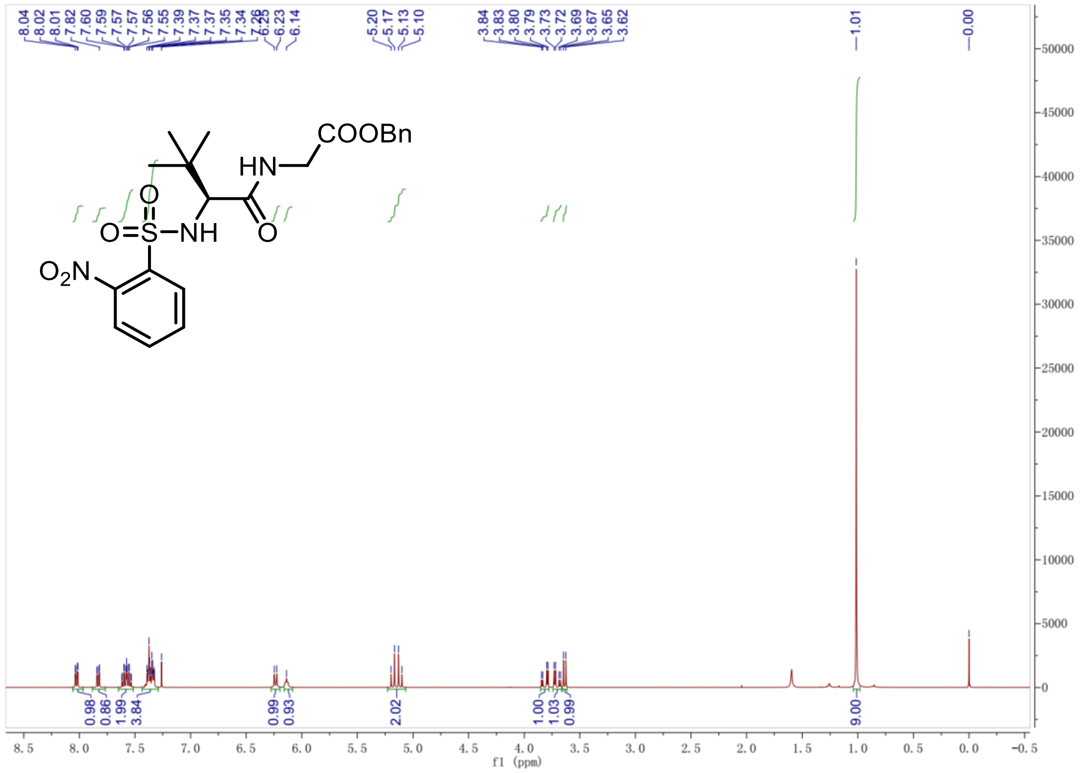

Supplementary Figure 179. ^{1}H NMR (400 MHz, $CDCl_3$) spectrum of compound **4j**

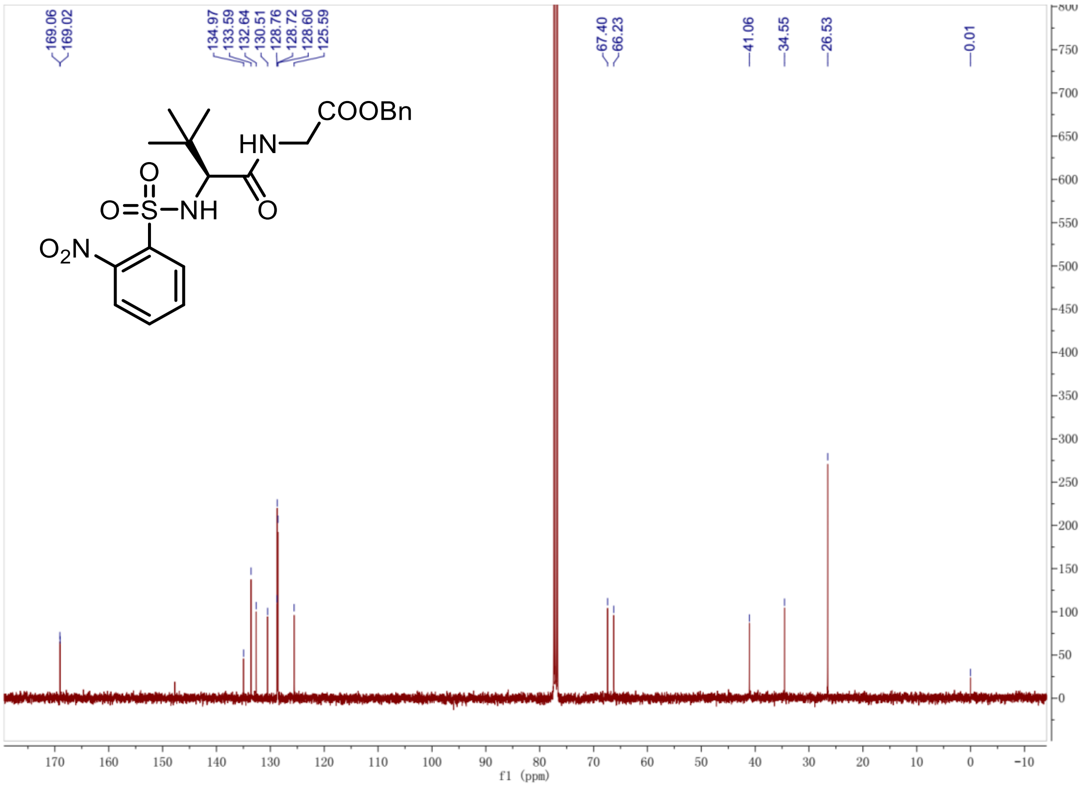

Supplementary Figure 180. ^{13}C NMR (100 MHz, $CDCl_3$) spectrum of compound **4j**

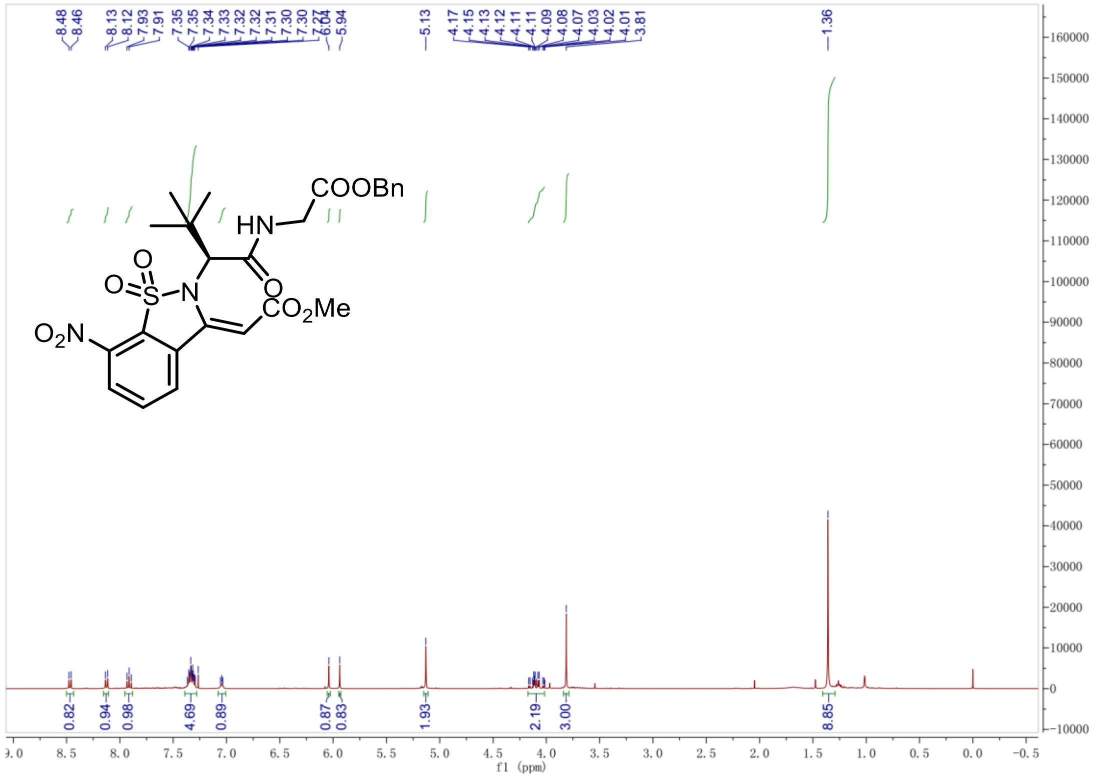

Supplementary Figure 181. ^{1}H NMR (400 MHz, $CDCl_3$) spectrum of compound **5jk**

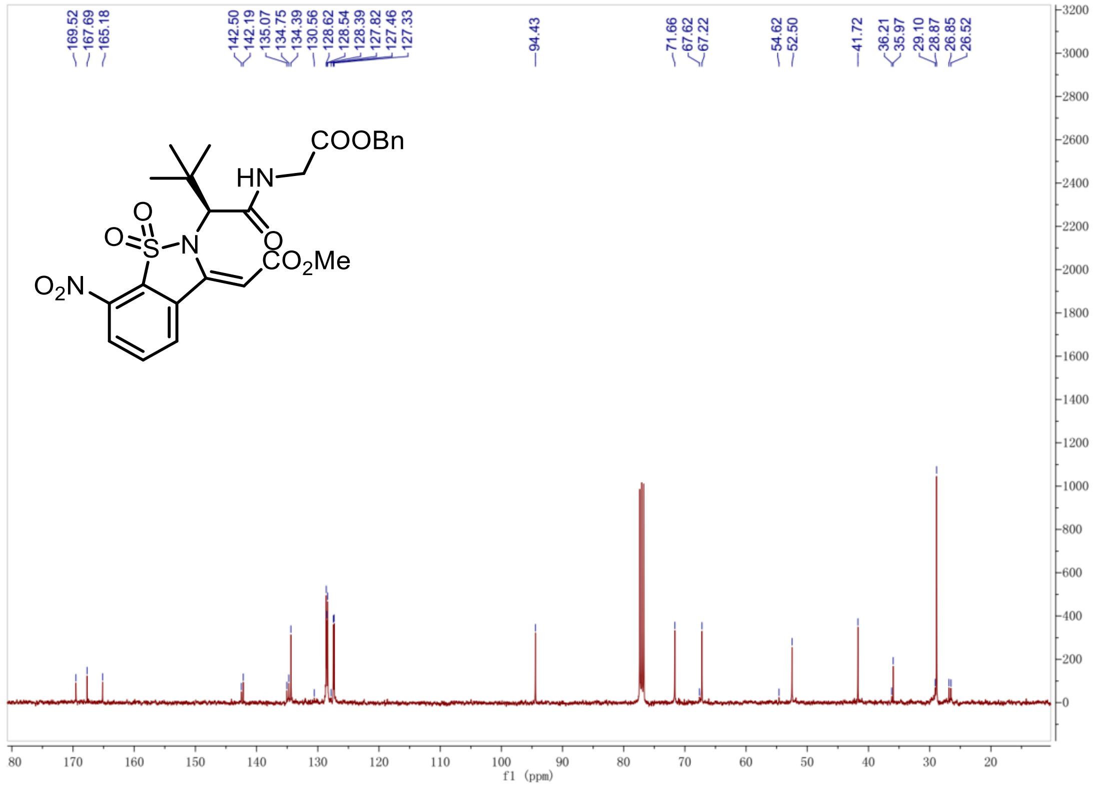

Supplementary Figure 182. ^{13}C NMR (100 MHz, $CDCl_3$) spectrum of compound **5jk**

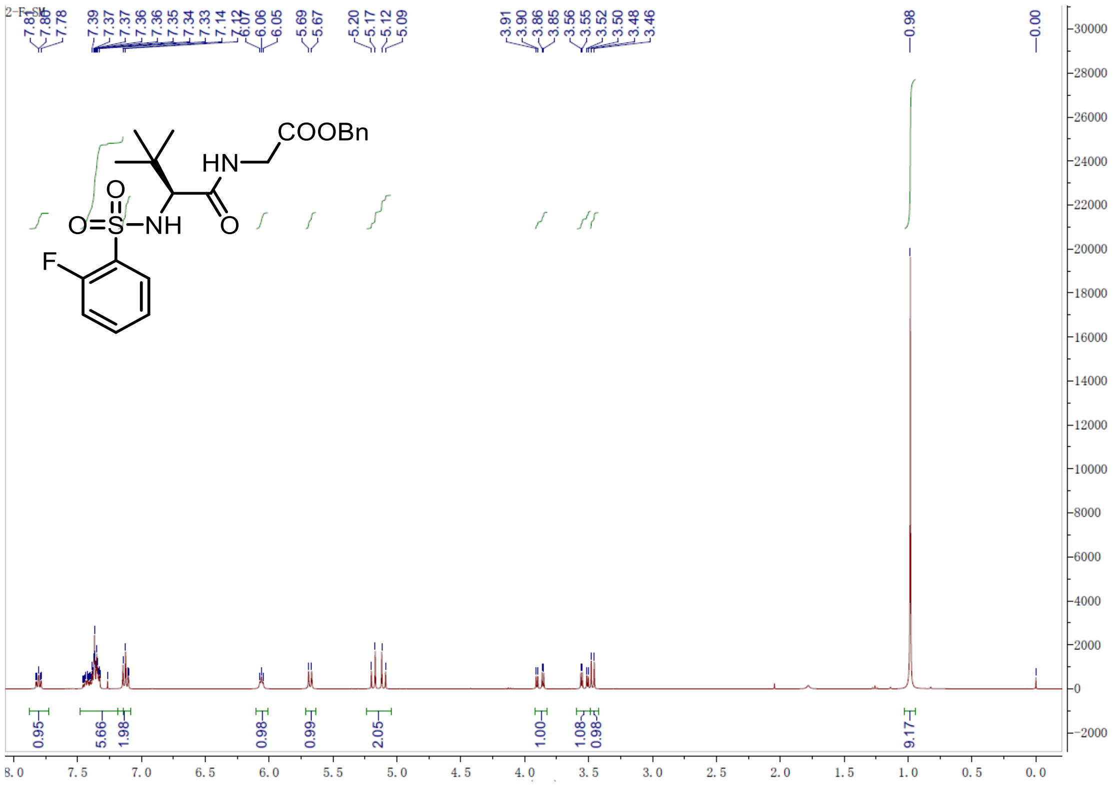

Supplementary Figure 183. ^{1}H NMR (400 MHz, $CDCl_3$) spectrum of compound **4k**

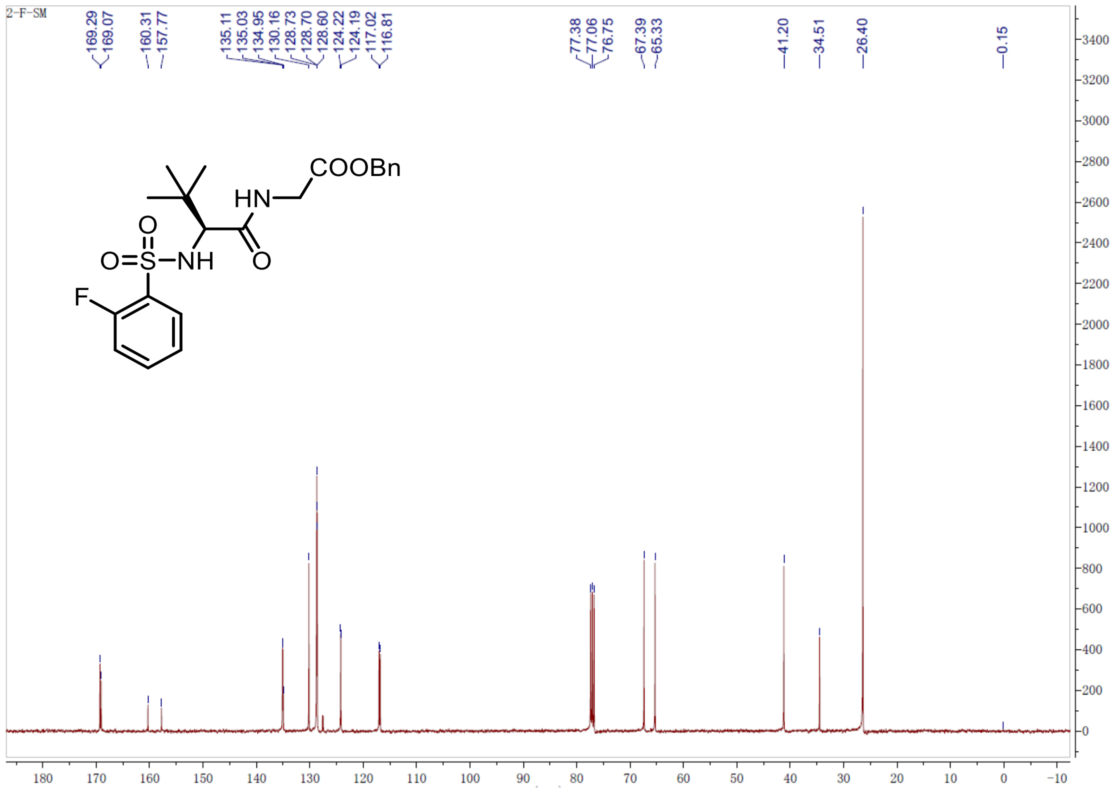

Supplementary Figure 184. ^{13}C NMR (100 MHz, $CDCl_3$) spectrum of compound **4k**

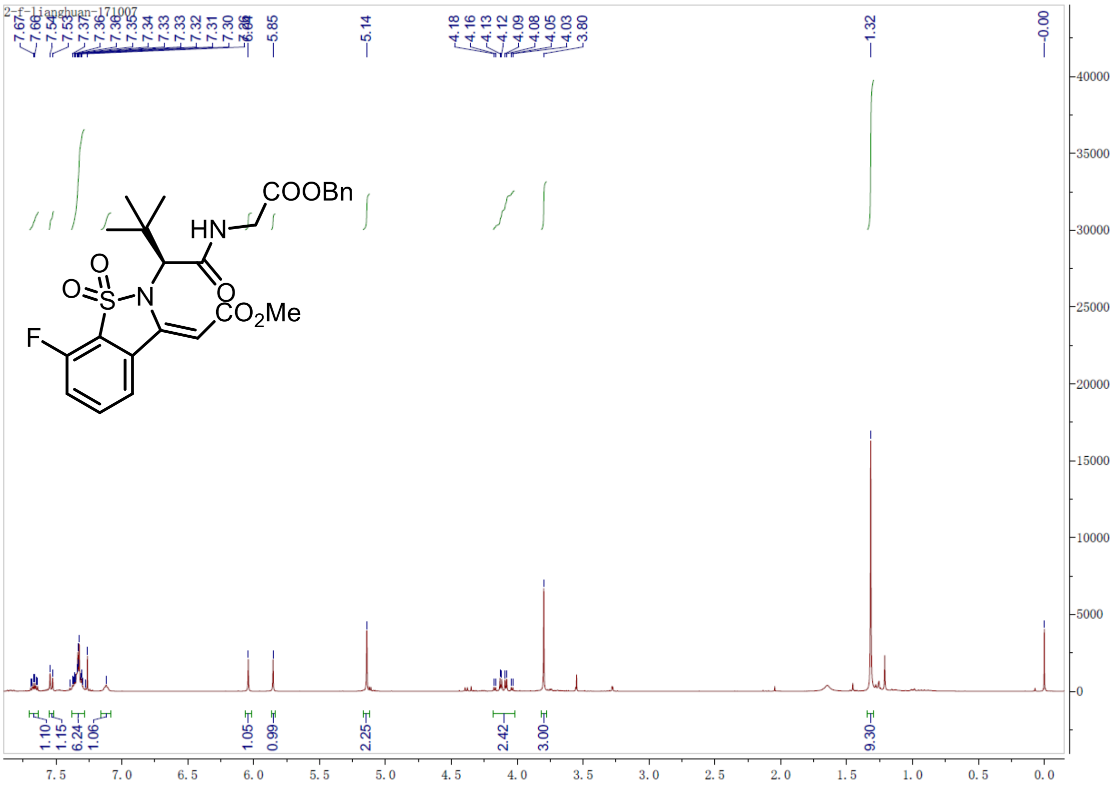

Supplementary Figure 185. ^{1}H NMR (400 MHz, $CDCl_3$) spectrum of compound **5kk**

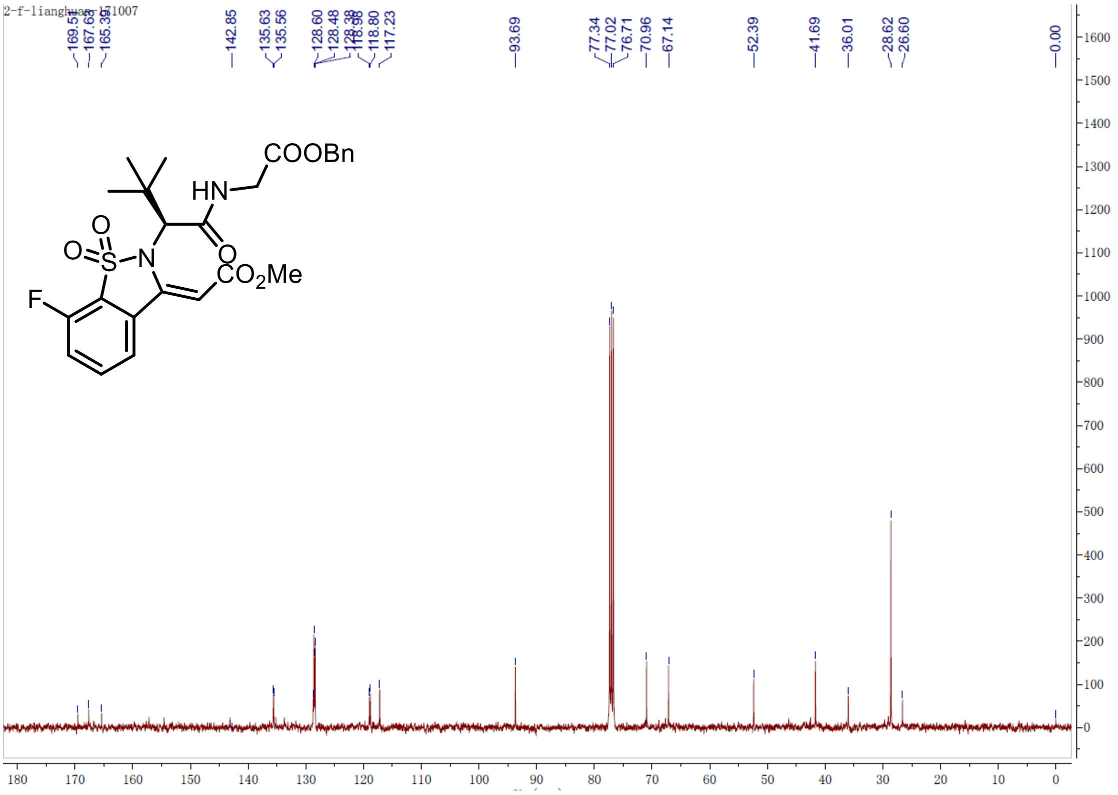

Supplementary Figure 186. ^{13}C NMR (100 MHz, $CDCl_3$) spectrum of compound **5kk**

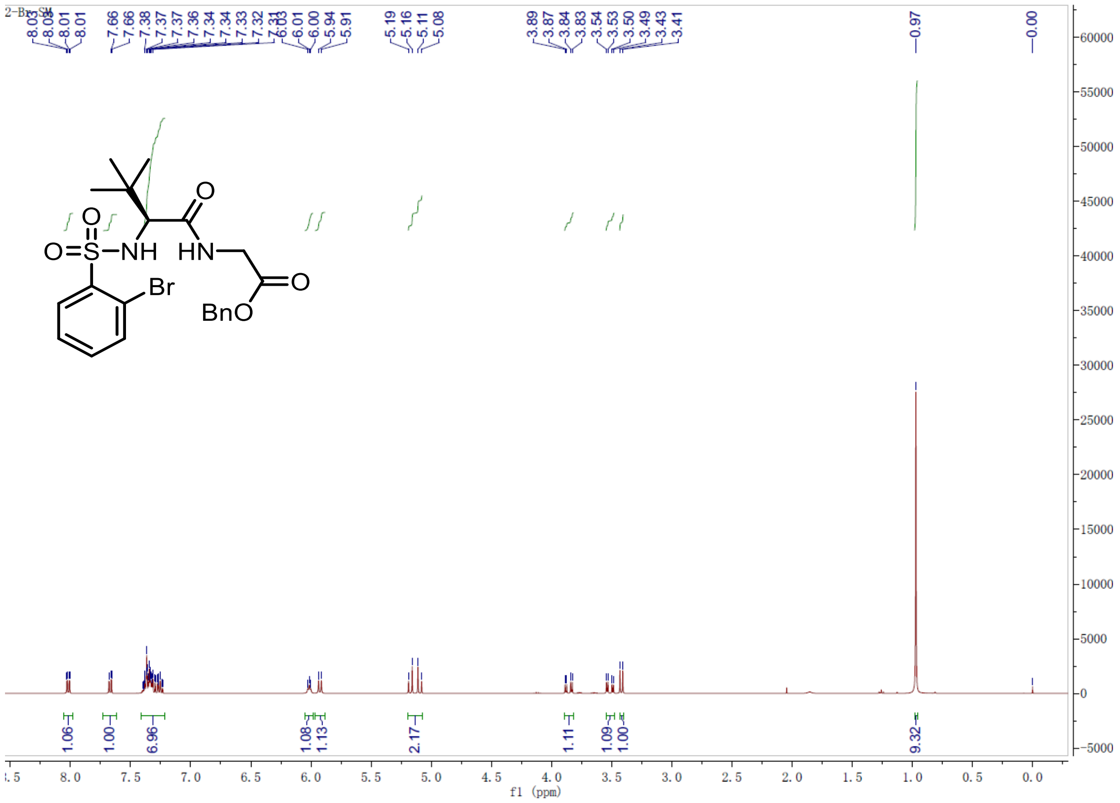

Supplementary Figure 187. ^{1}H NMR (400 MHz, $CDCl_3$) spectrum of compound **4l**

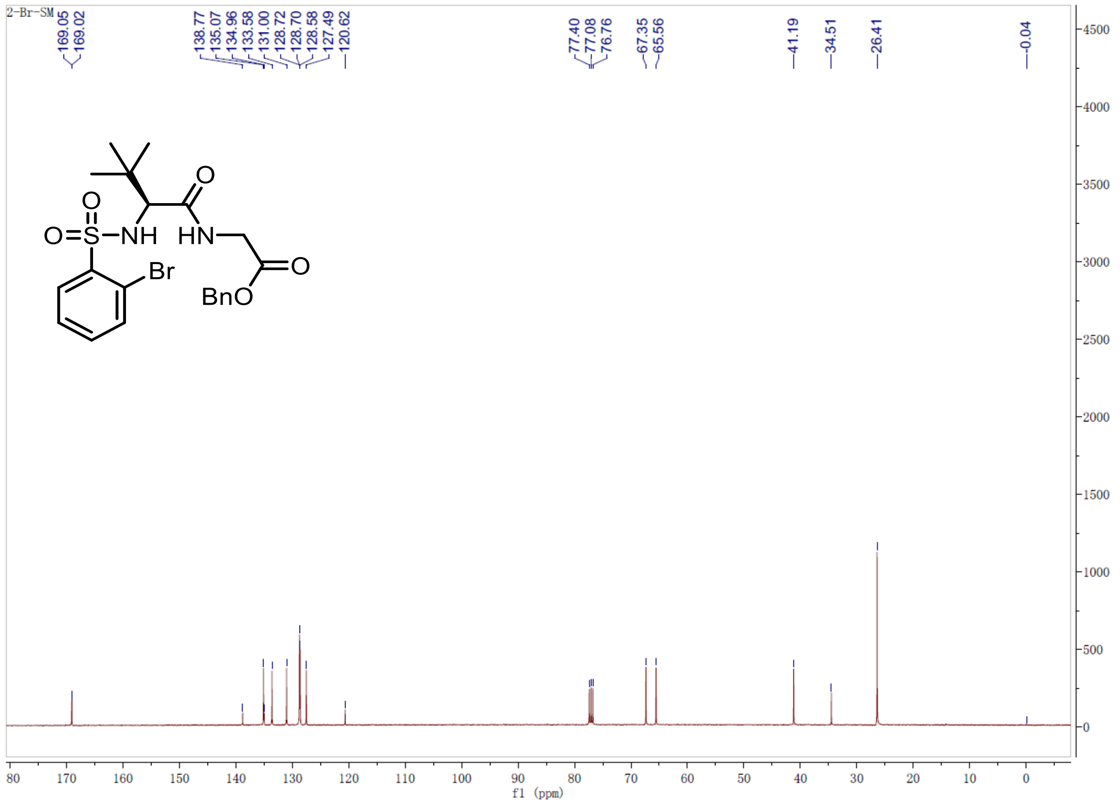

Supplementary Figure 188. ^{13}C NMR (100 MHz, $CDCl_3$) spectrum of compound **4l**

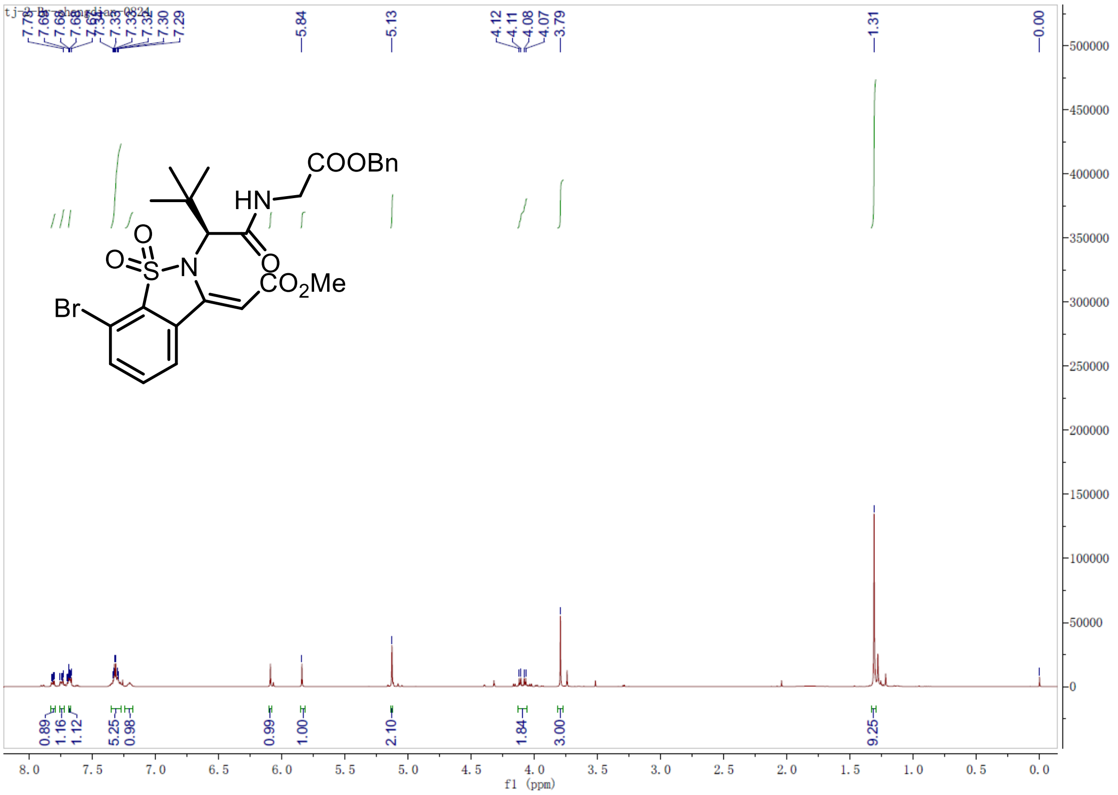

Supplementary Figure 189. ^{1}H NMR (400 MHz, $CDCl_3$) spectrum of compound **5lk**

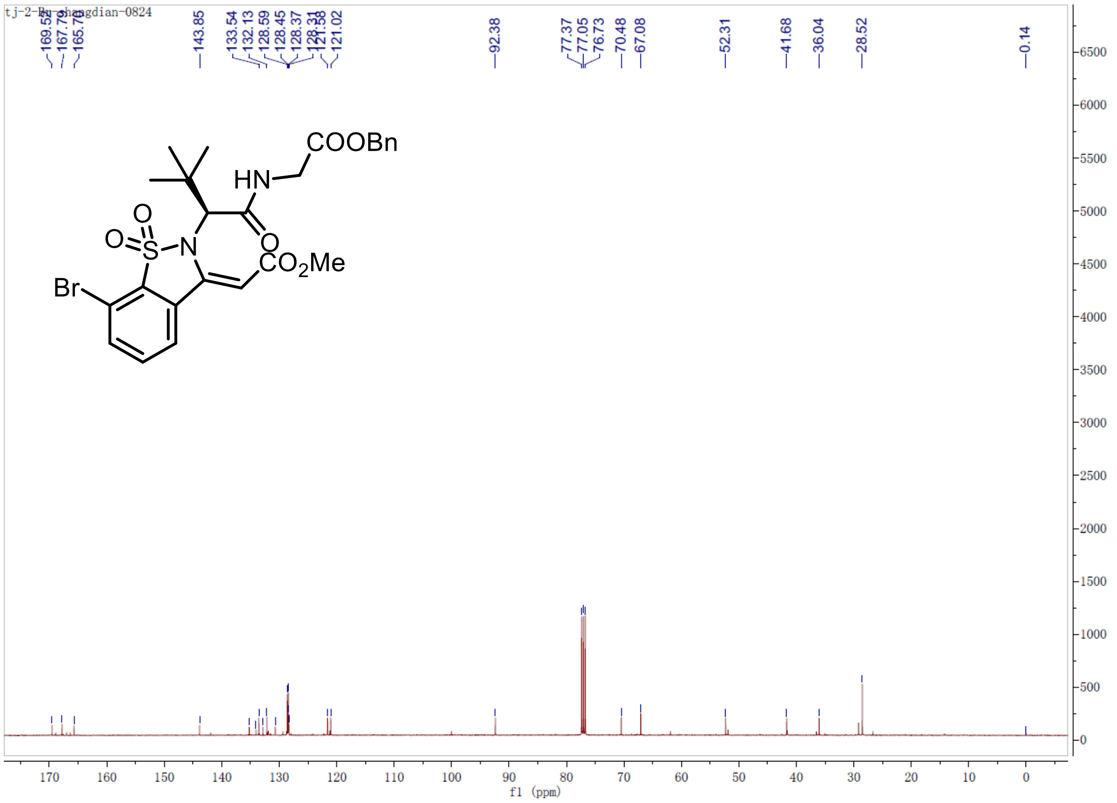

Supplementary Figure 190. ^{13}C NMR (100 MHz, $CDCl_3$) spectrum of compound **5lk**

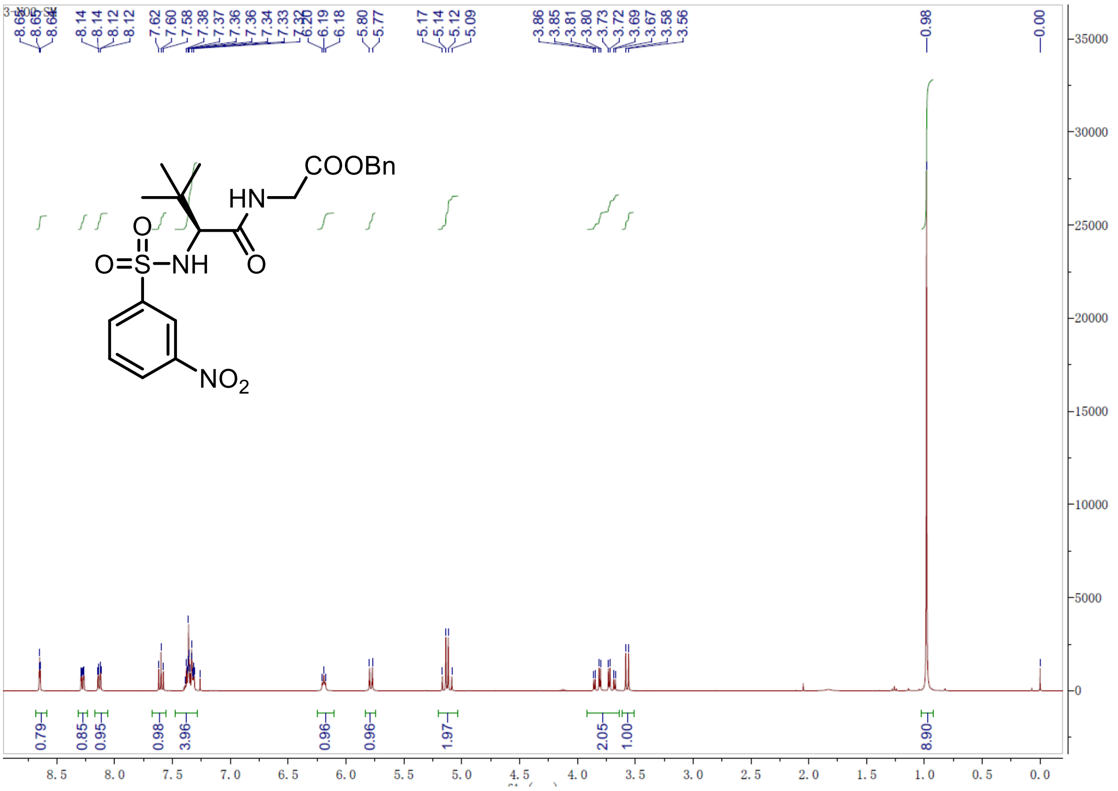

Supplementary Figure 191. ^{1}H NMR (400 MHz, $CDCl_3$) spectrum of compound **4m**

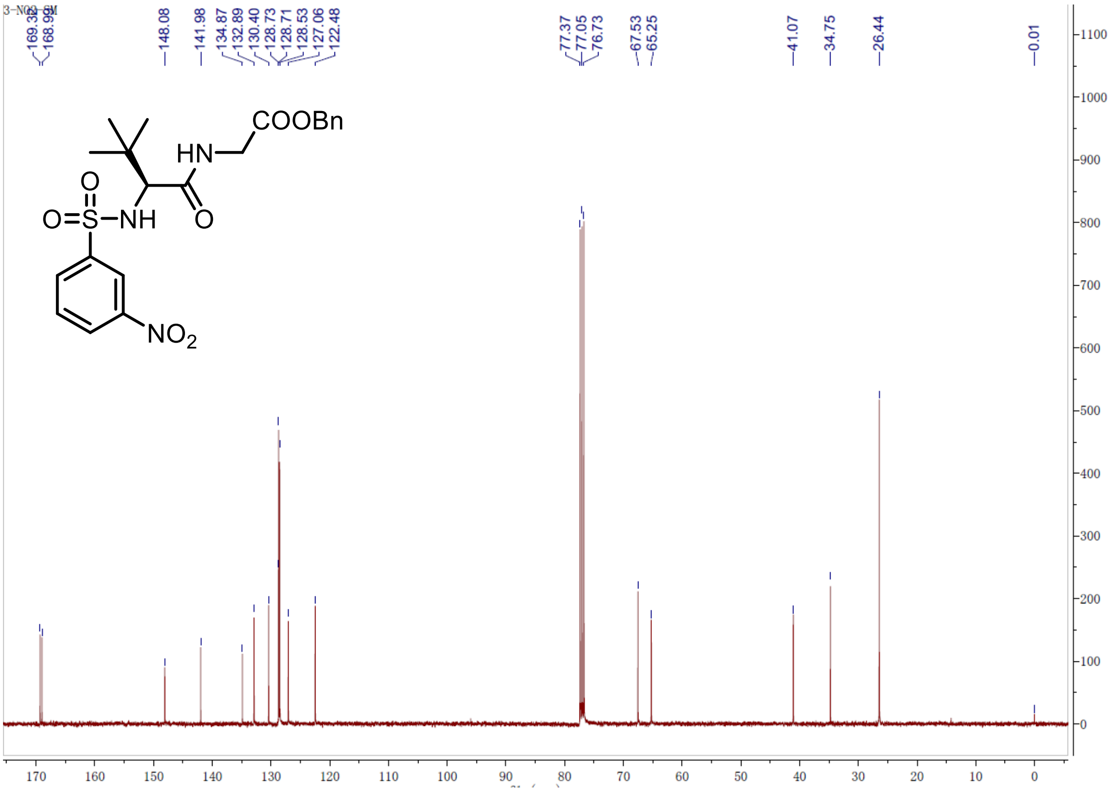

Supplementary Figure 192. ^{13}C NMR (100 MHz, $CDCl_3$) spectrum of compound **4m**

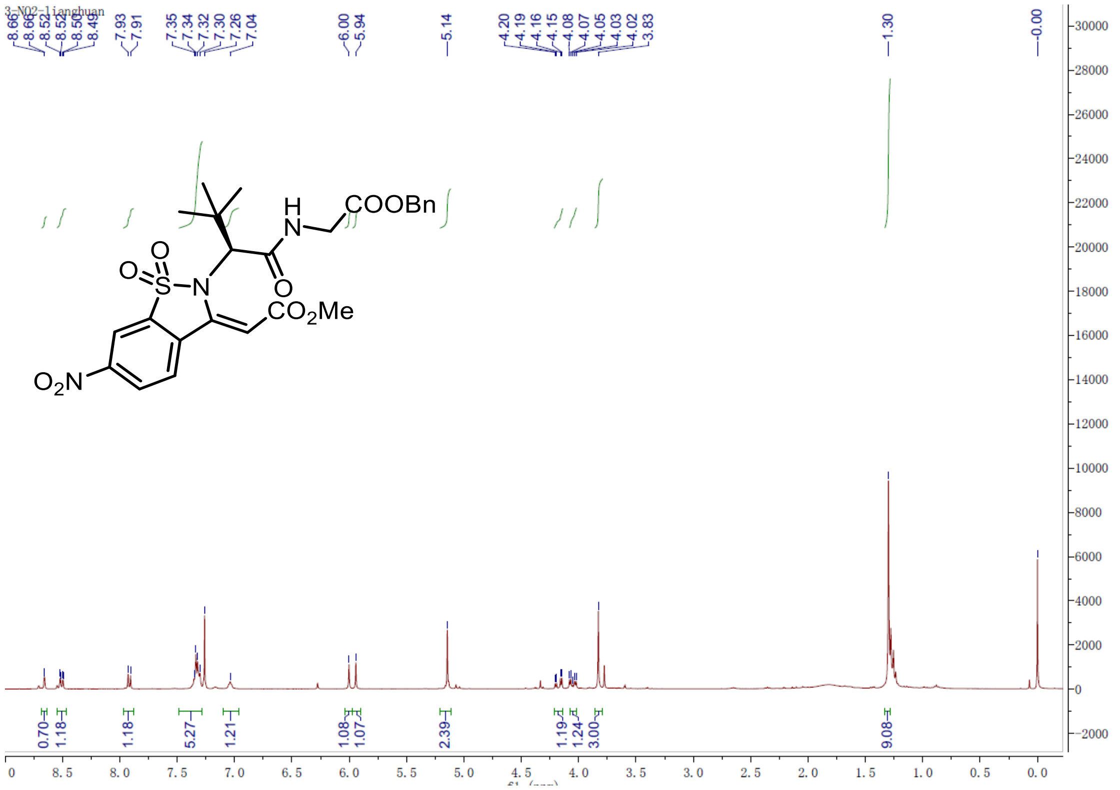

Supplementary Figure 193. ^{1}H NMR (400 MHz, $CDCl_3$) spectrum of compound **5mi**

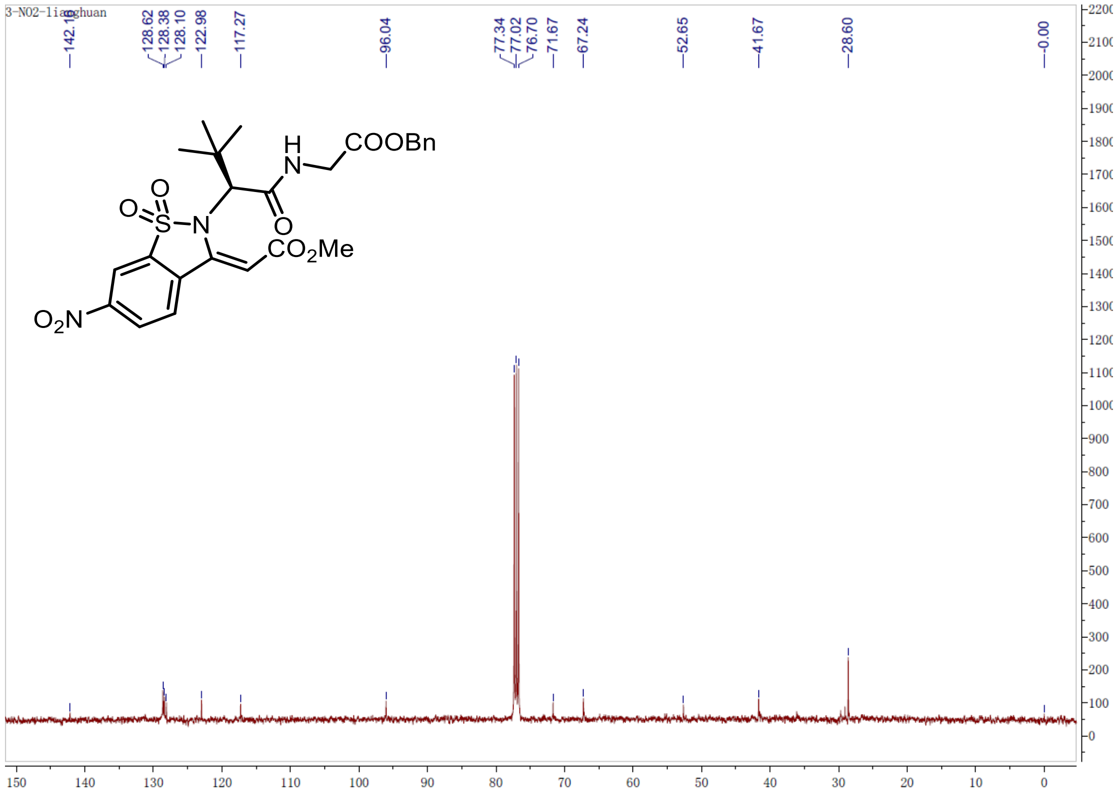

Supplementary Figure 194. ^{13}C NMR (100 MHz, $CDCl_3$) spectrum of compound **5mi**

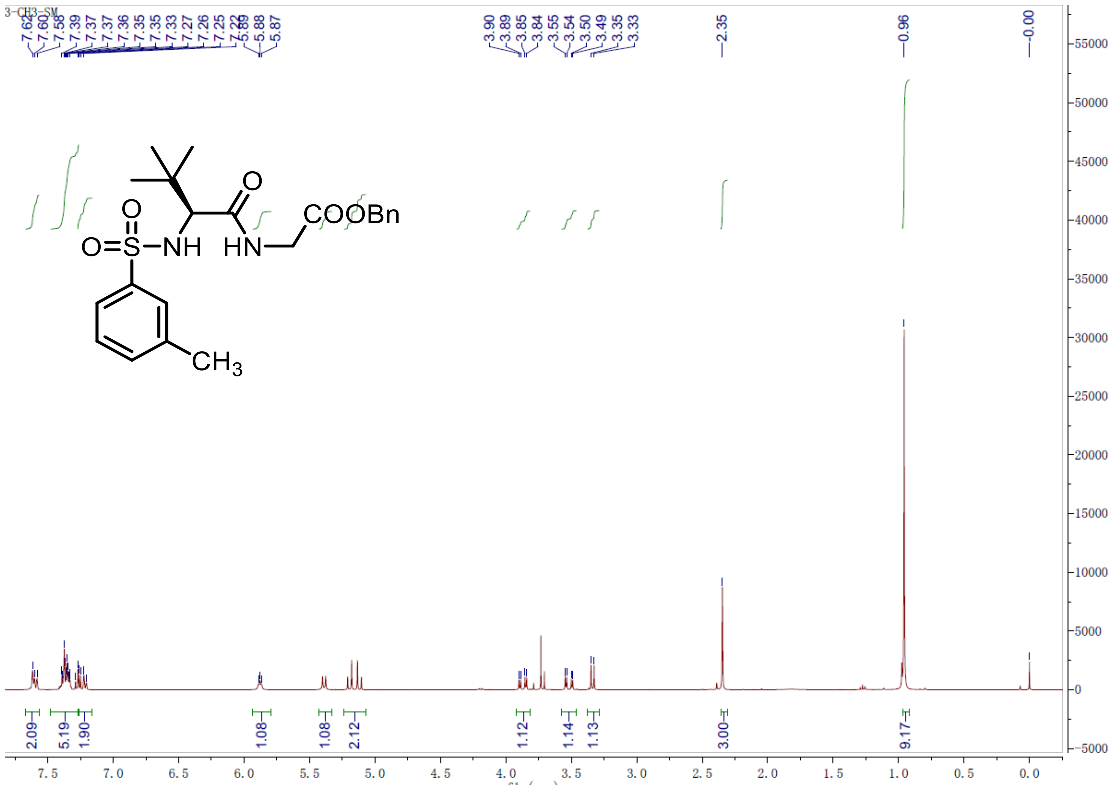

Supplementary Figure 195. ^{1}H NMR (400 MHz, $CDCl_3$) spectrum of compound **4n**

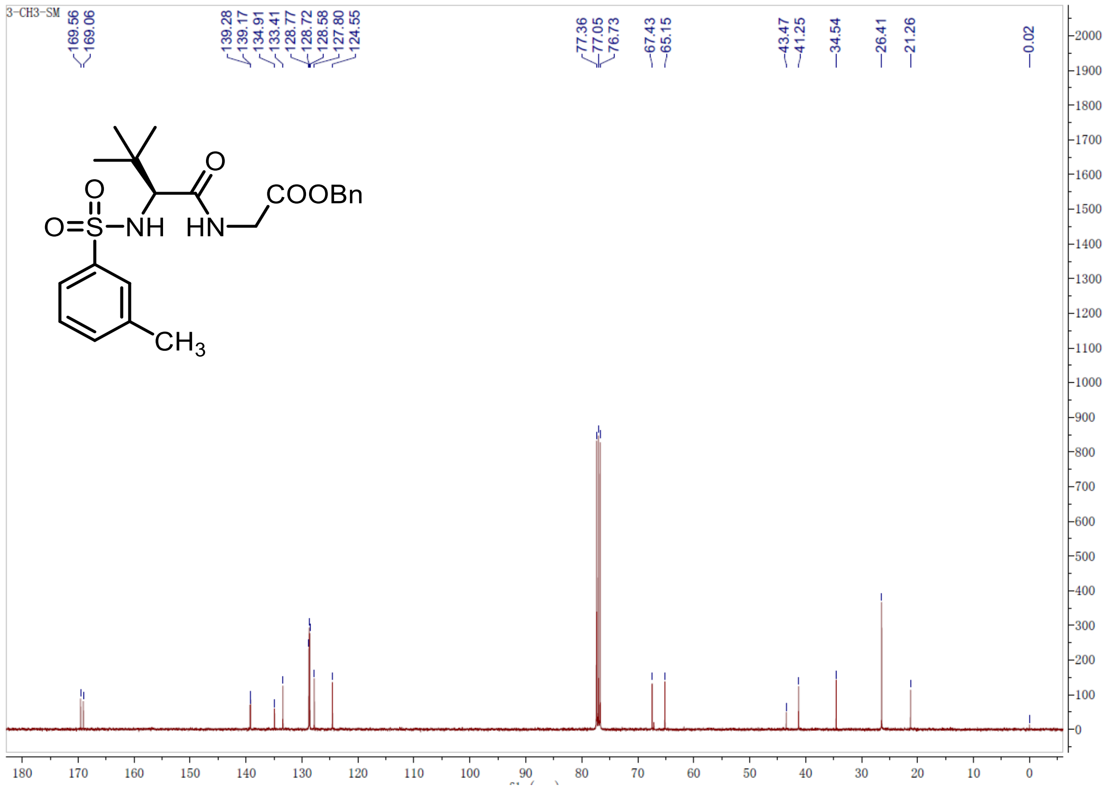

Supplementary Figure 196. ^{13}C NMR (100 MHz, $CDCl_3$) spectrum of compound **4n**

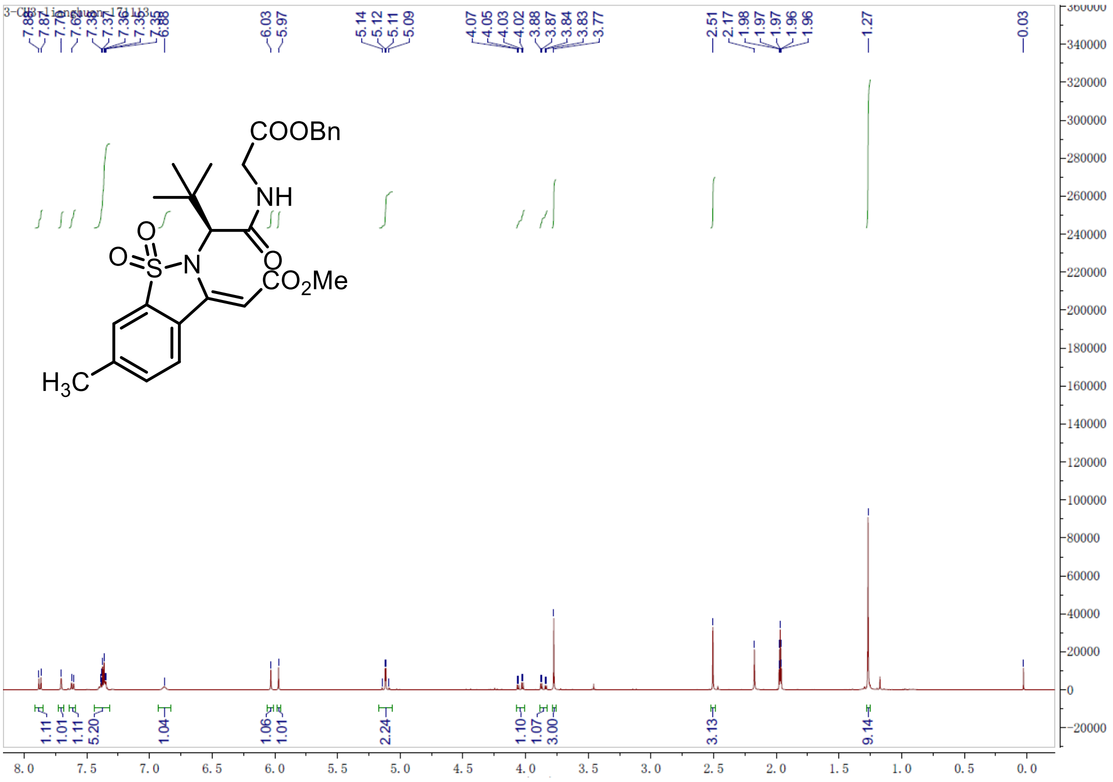

Supplementary Figure 197. ^{1}H NMR (400 MHz, CD_3CN) spectrum of compound **5nk**

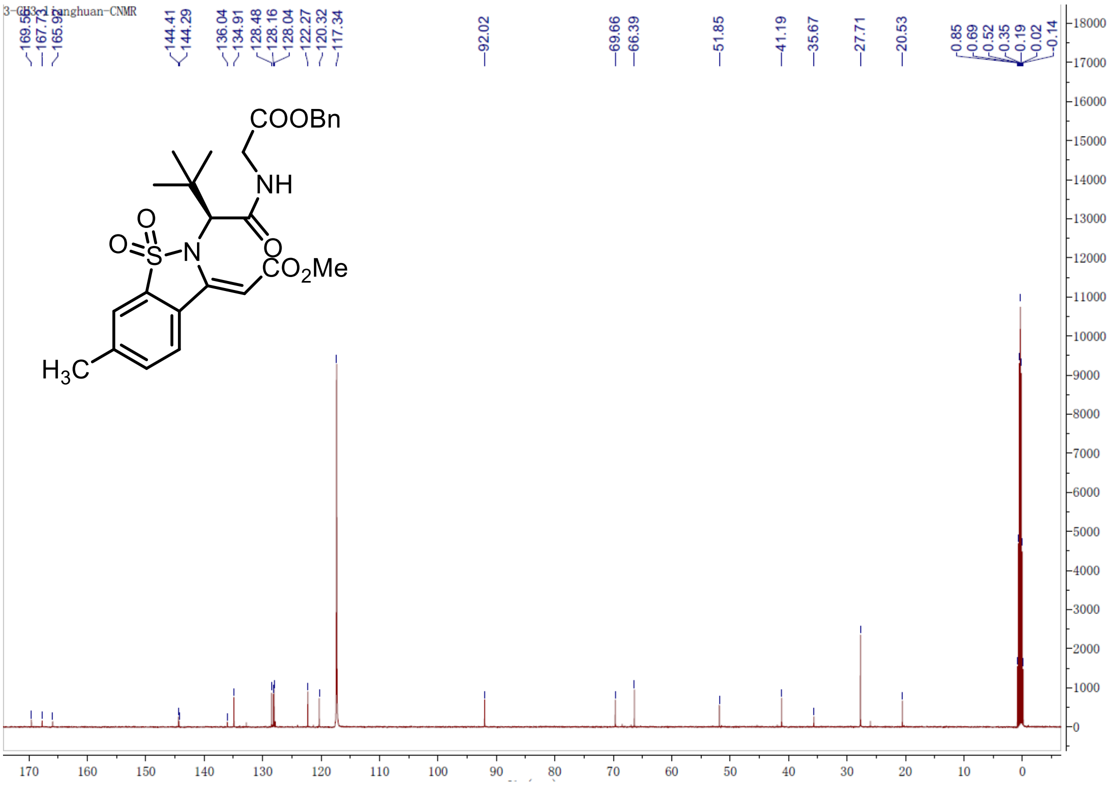

Supplementary Figure 198. ^{13}C NMR (100 MHz, CD_3CN) spectrum of compound **5nk**

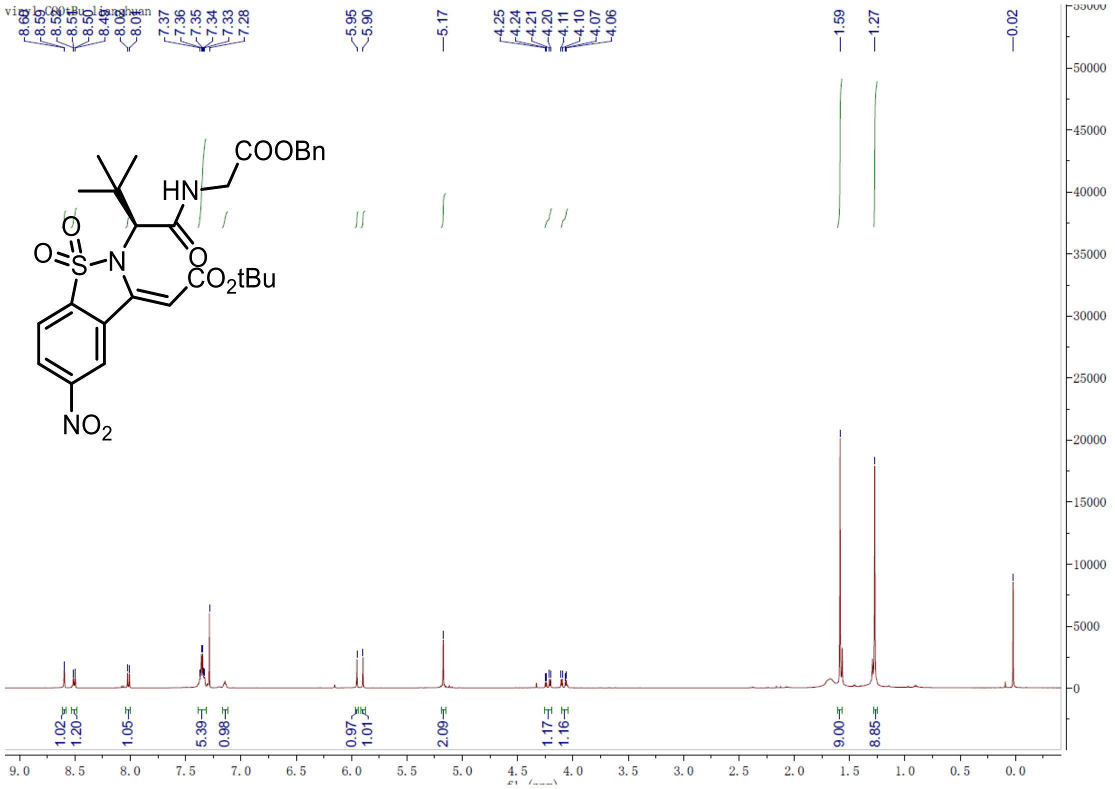

Supplementary Figure 199. ^{1}H NMR (400 MHz, $CDCl_3$) spectrum of compound **5aa**

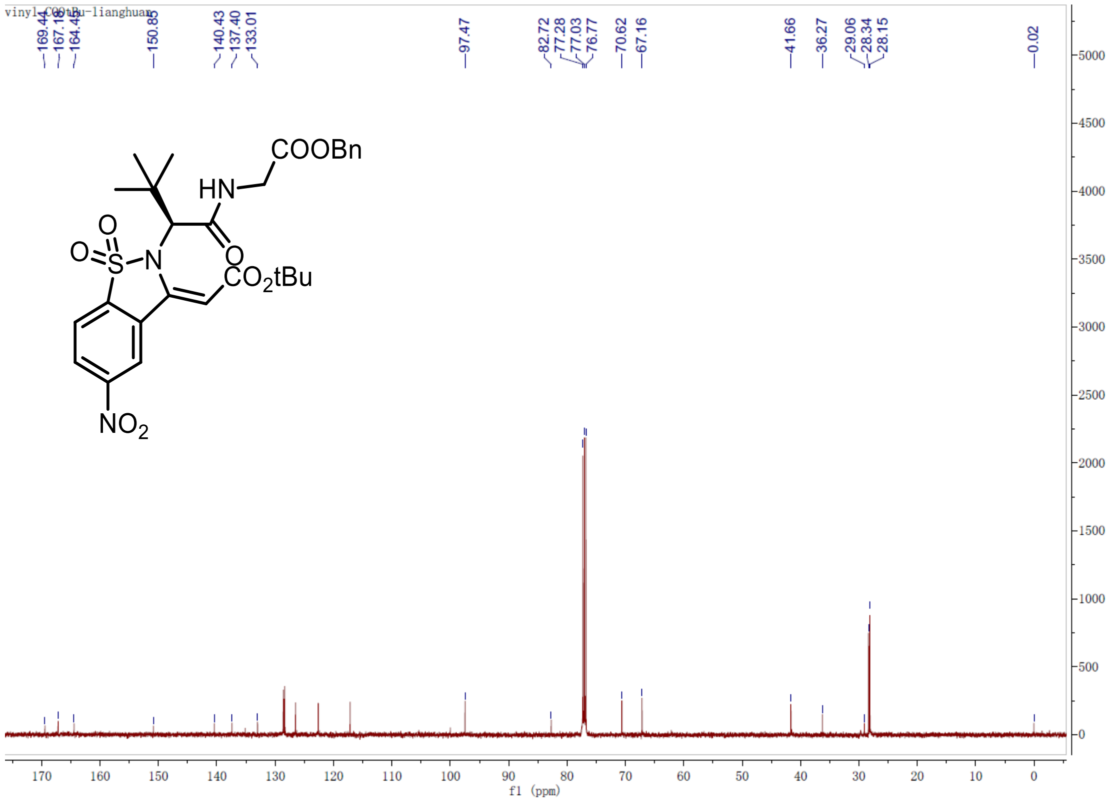

Supplementary Figure 200. ^{13}C NMR (100 MHz, $CDCl_3$) spectrum of compound **5aa**

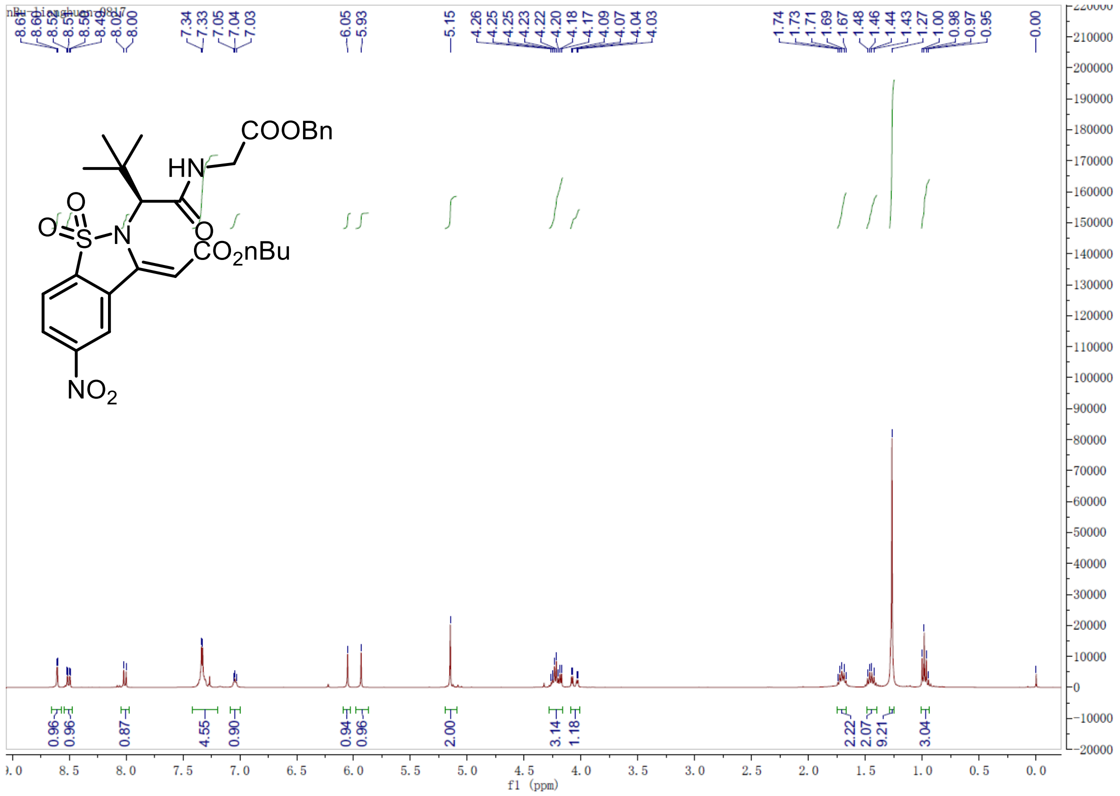

Supplementary Figure 201. ^{1}H NMR (400 MHz, $CDCl_3$) spectrum of compound **5ab**

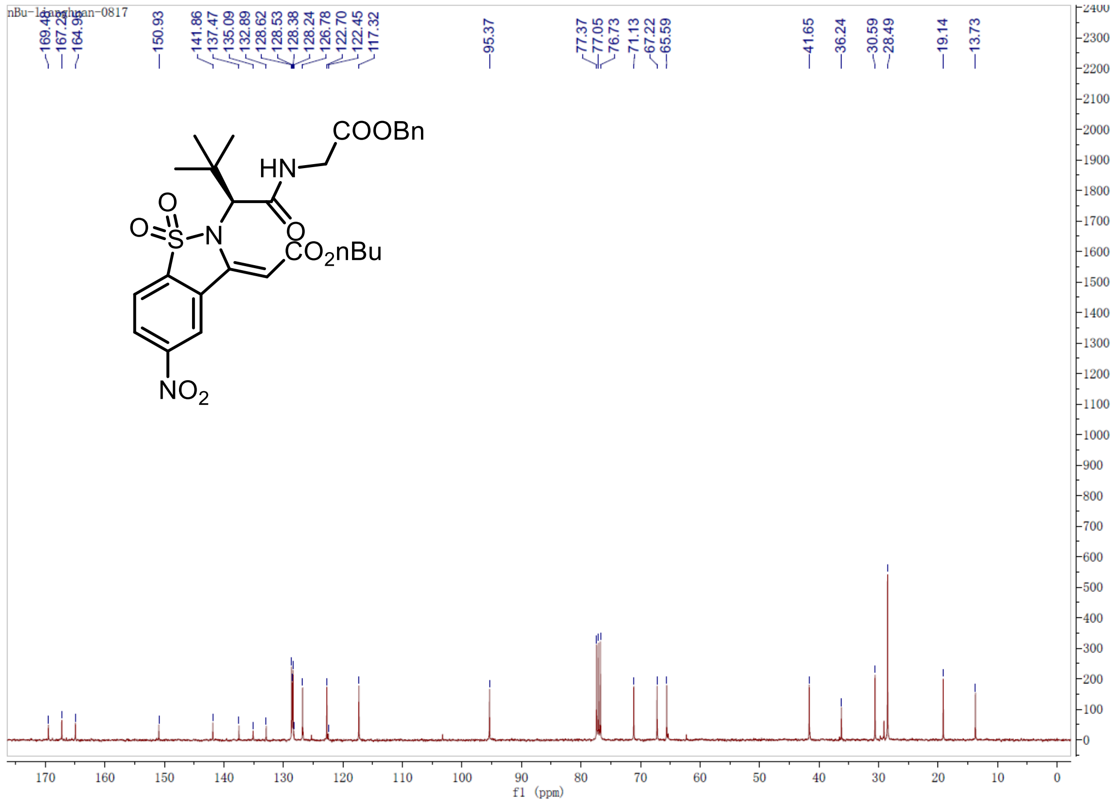

Supplementary Figure 202. ^{13}C NMR (100 MHz, $CDCl_3$) spectrum of compound **5ab**

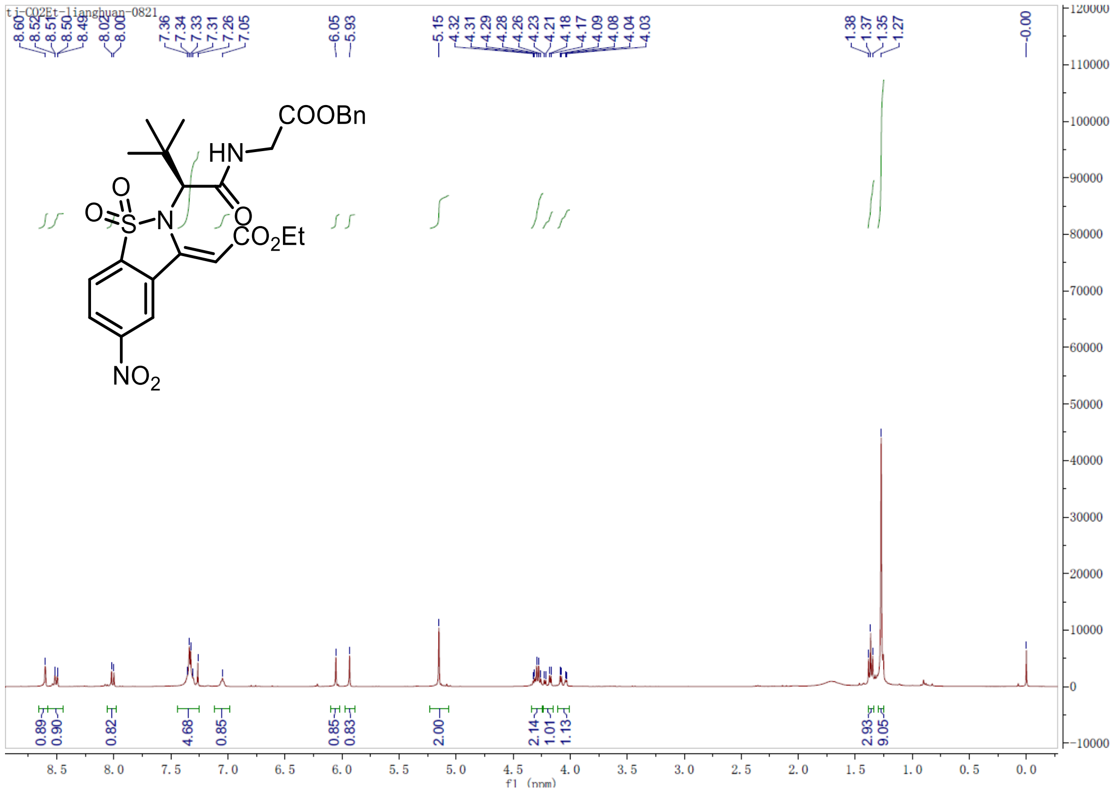

Supplementary Figure 203. ^{1}H NMR (400 MHz, $CDCl_3$) spectrum of compound **5ac**

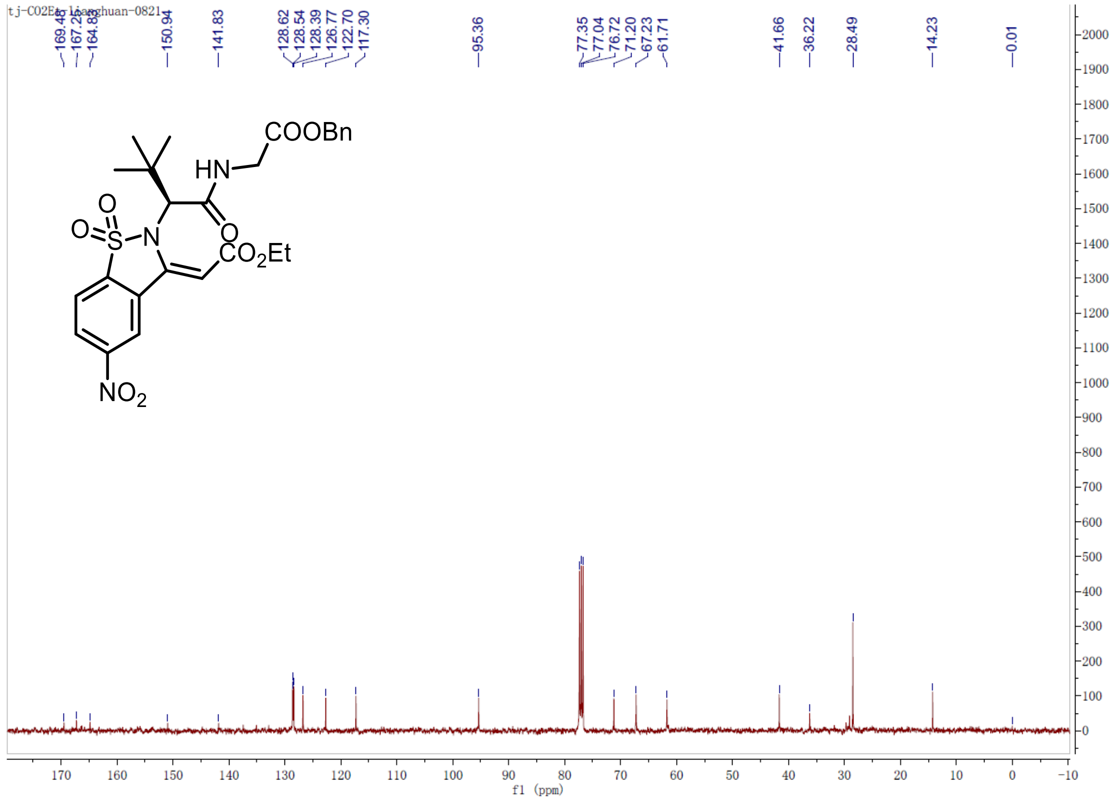

Supplementary Figure 204. ^{13}C NMR (100 MHz, $CDCl_3$) spectrum of compound **5ac**

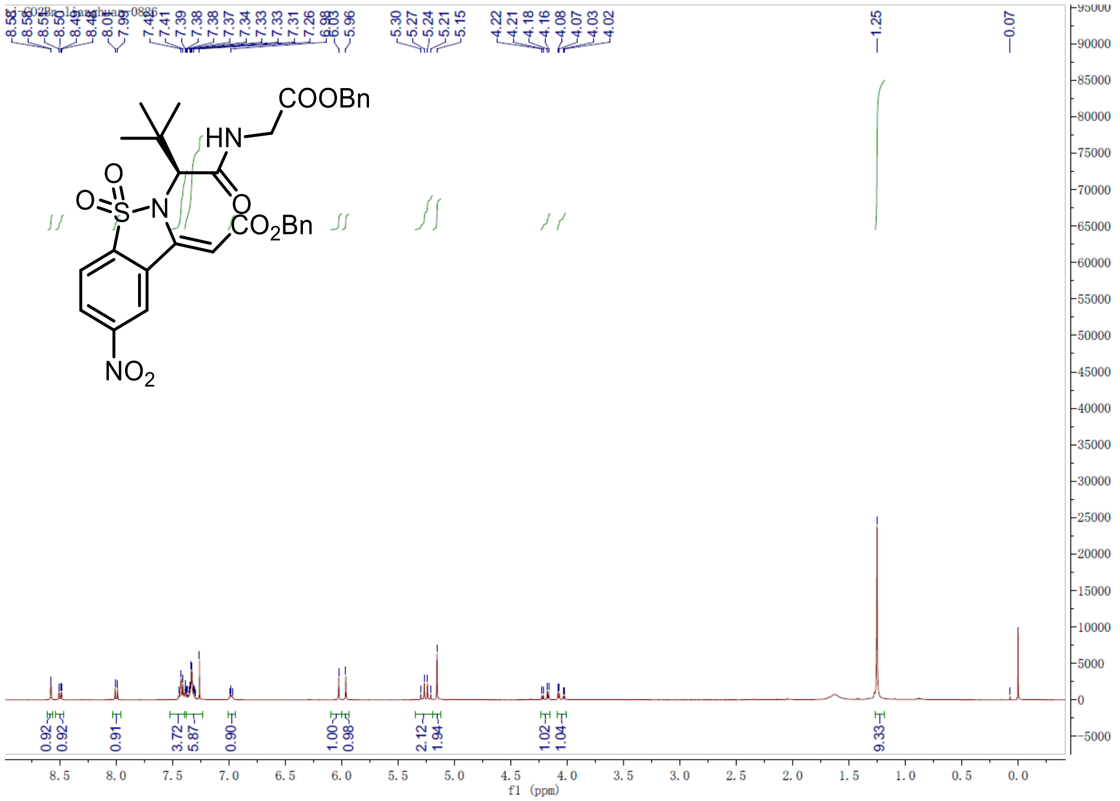

Supplementary Figure 205. ^{1}H NMR (400 MHz, $CDCl_3$) spectrum of compound **5ad**

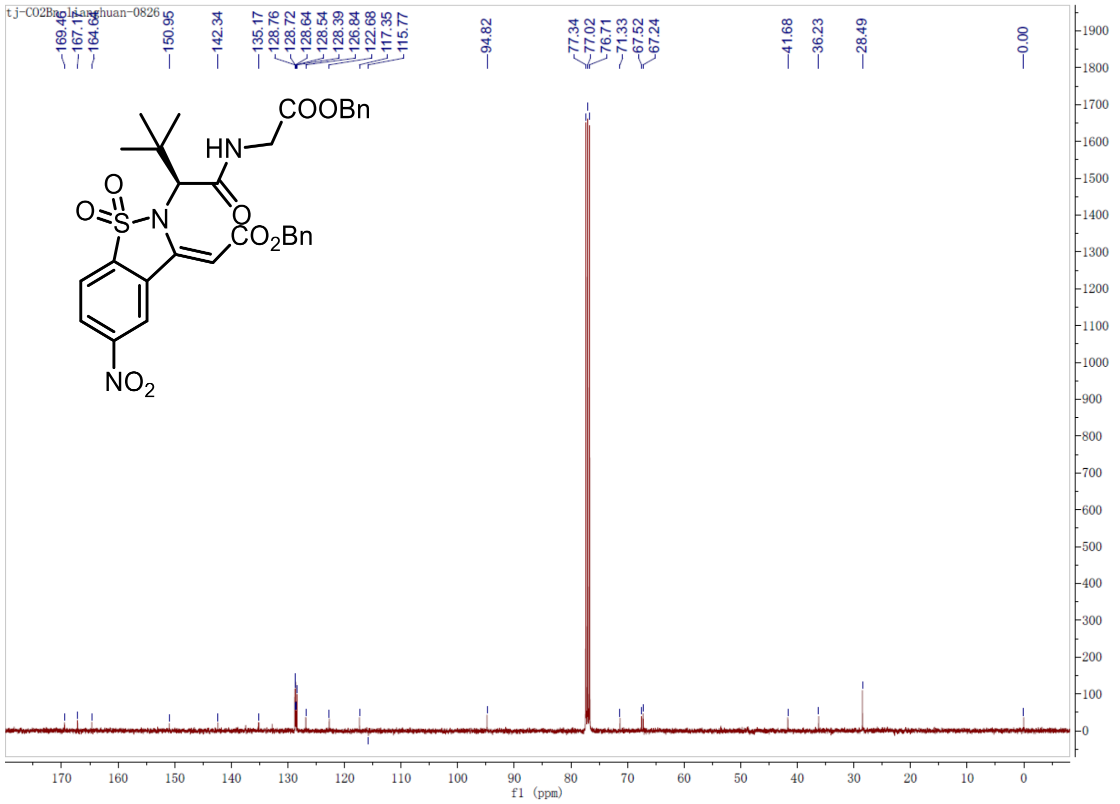

Supplementary Figure 206. ^{13}C NMR (100 MHz, $CDCl_3$) spectrum of compound **5ad**

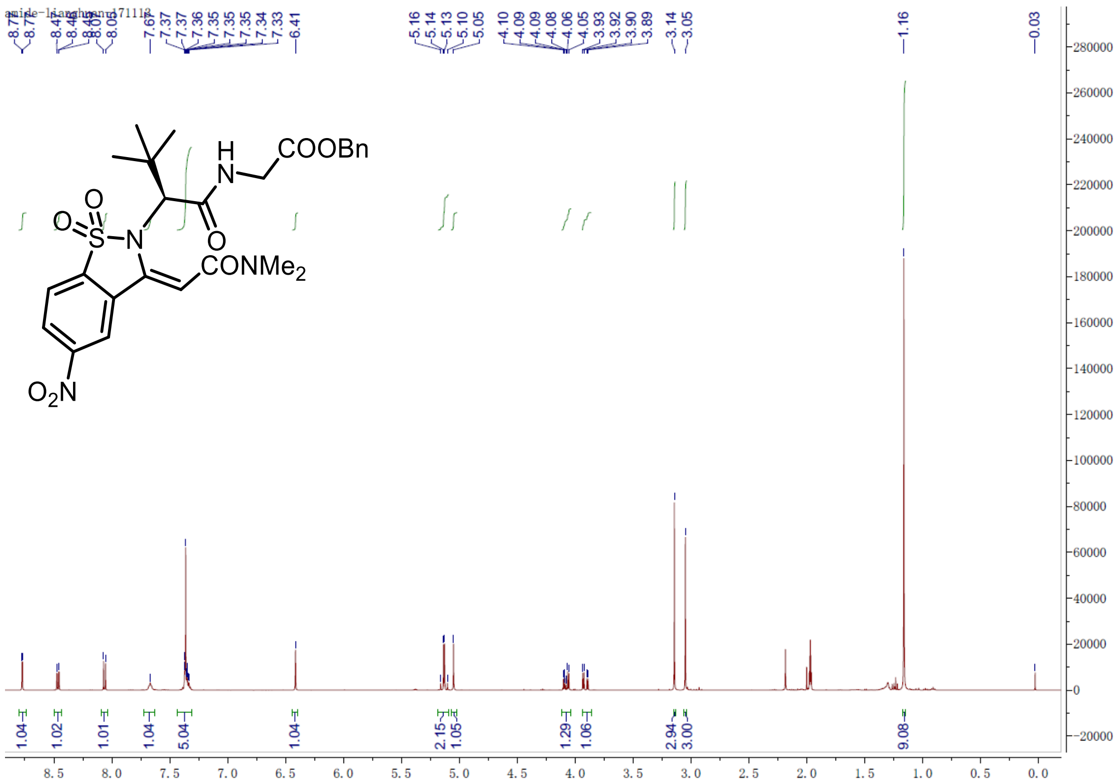

Supplementary Figure 207. ^{1}H NMR (400 MHz, CD_3CN) spectrum of compound **5af**

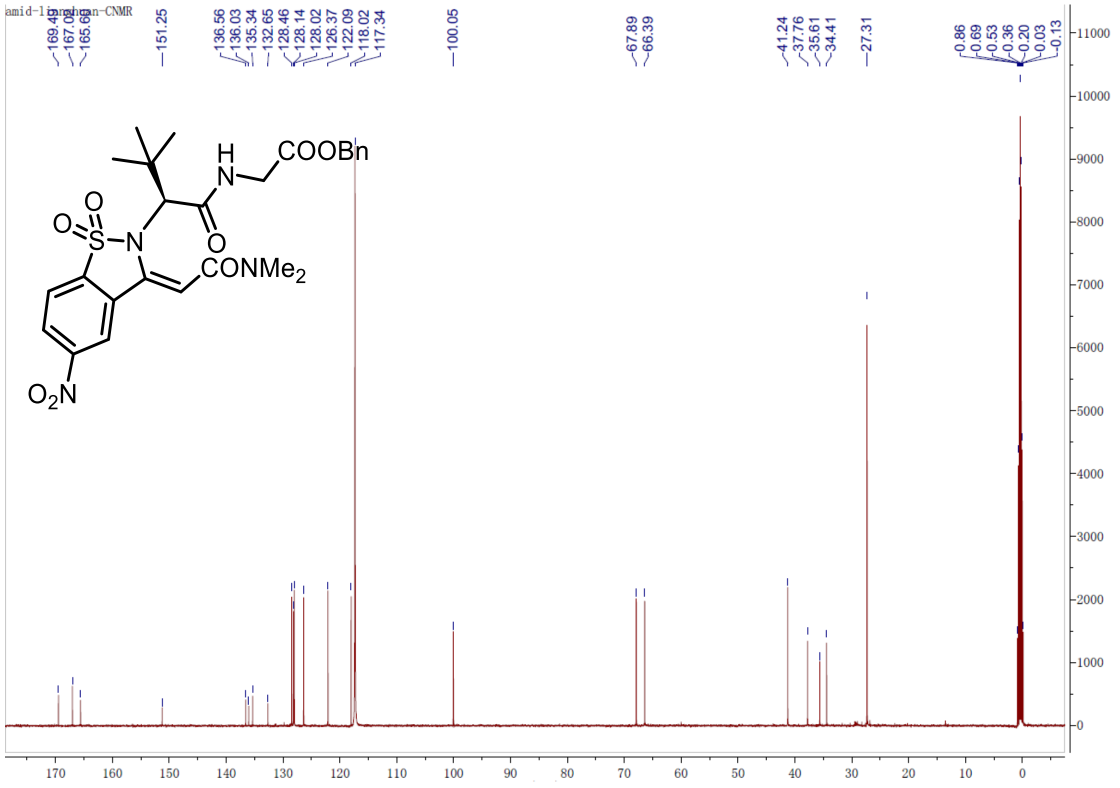

Supplementary Figure 208. ^{13}C NMR (100 MHz, CD_3CN) spectrum of compound **5af**

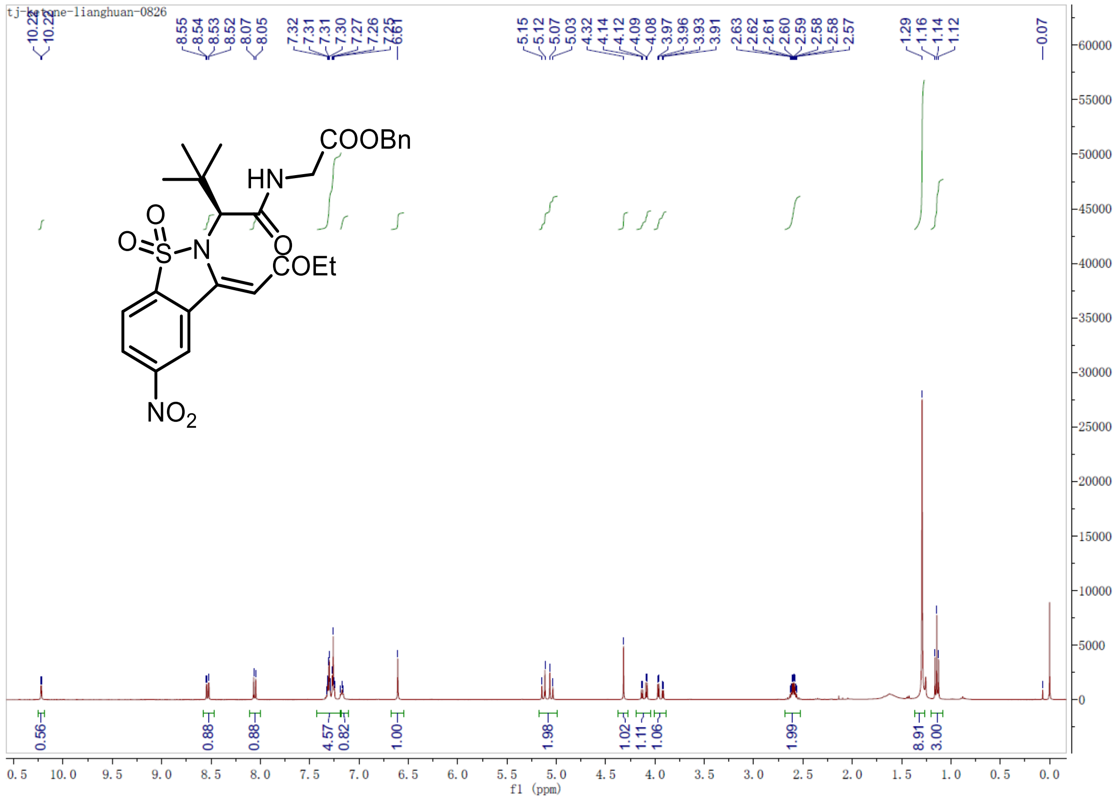

Supplementary Figure 209. ^{1}H NMR (400 MHz, $CDCl_3$) spectrum of compound **5ag**

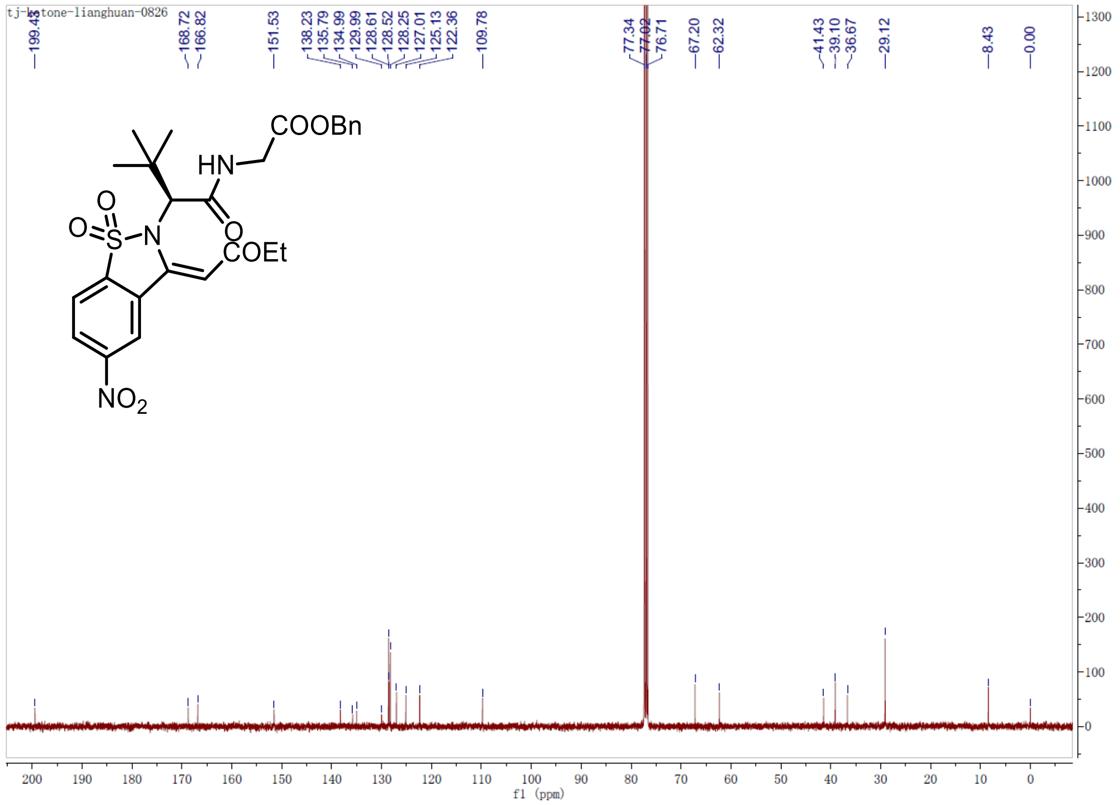

Supplementary Figure 210. ^{13}C NMR (100 MHz, $CDCl_3$) spectrum of compound **5ag**

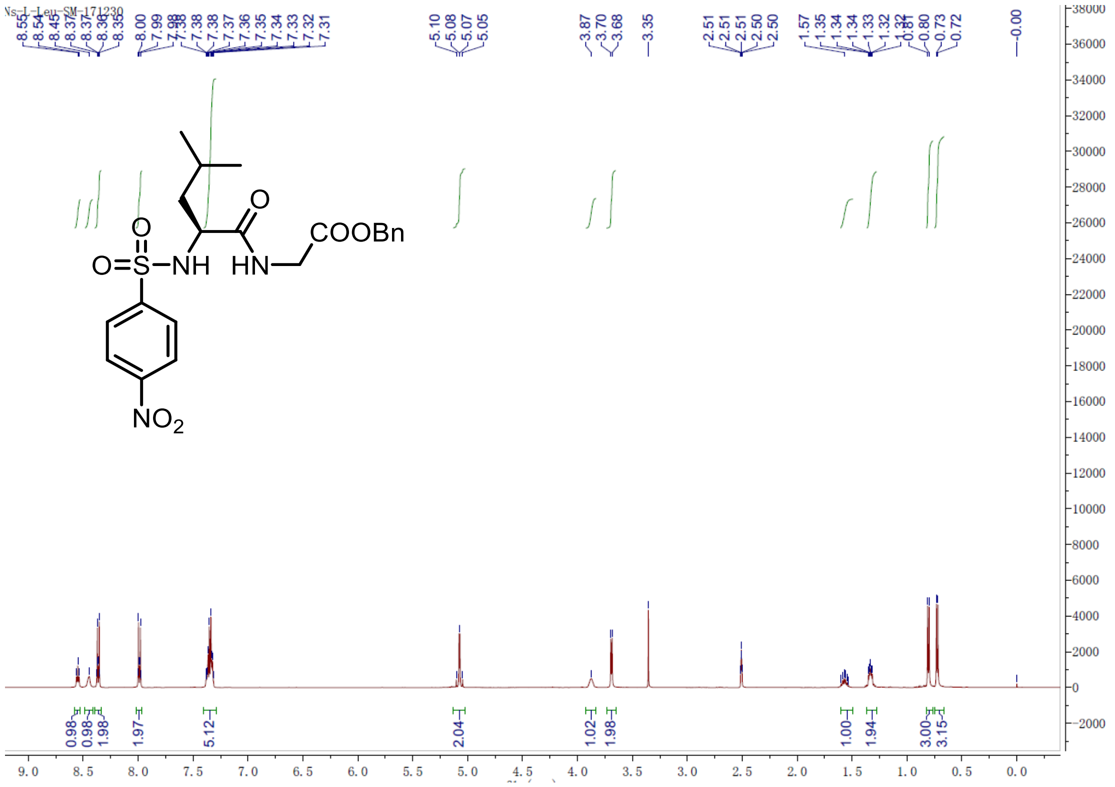

Supplementary Figure 211. ^{1}H NMR (400 MHz, DMSO) spectrum of compound **4o**

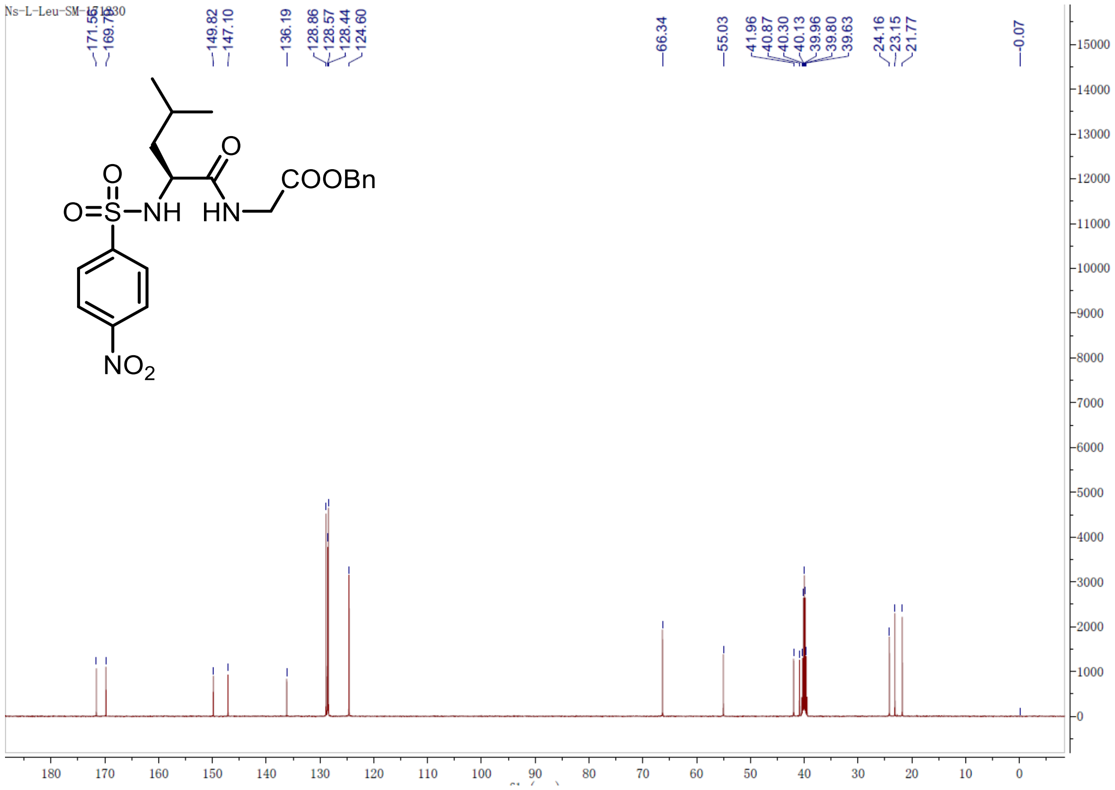

Supplementary Figure 212. ^{13}C NMR (100 MHz, DMSO) spectrum of compound **4o**

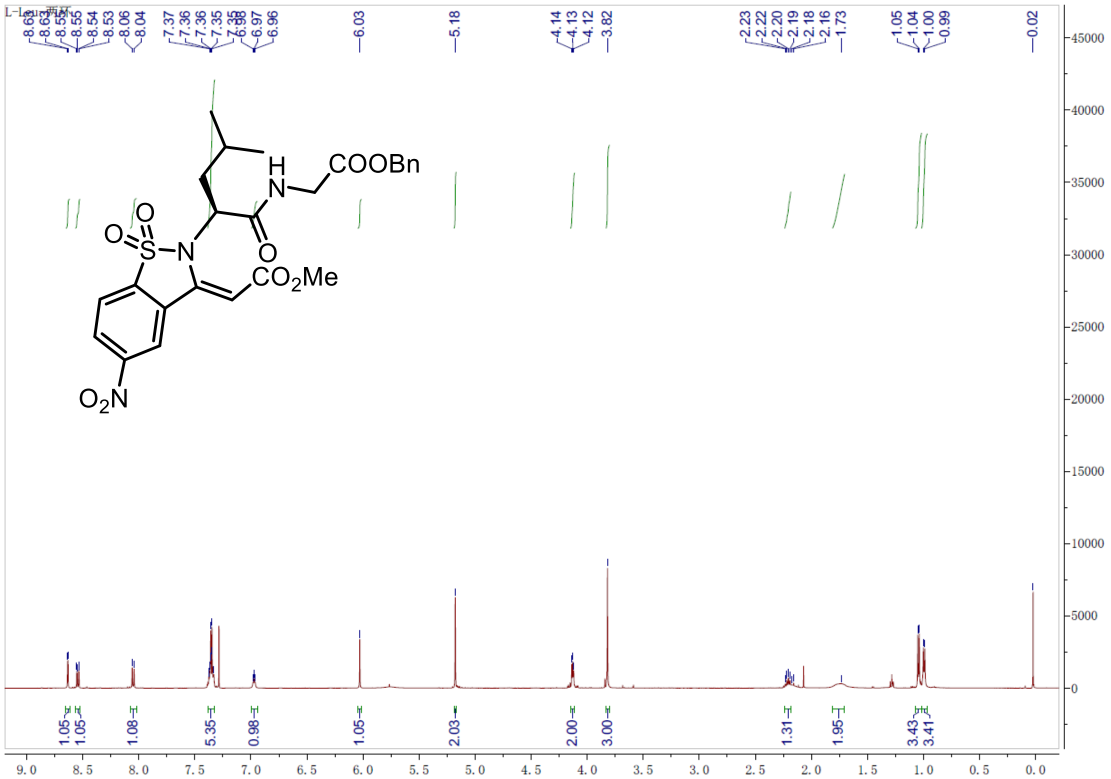

Supplementary Figure 213. ^{1}H NMR (400 MHz, $CDCl_3$) spectrum of compound **5ok**

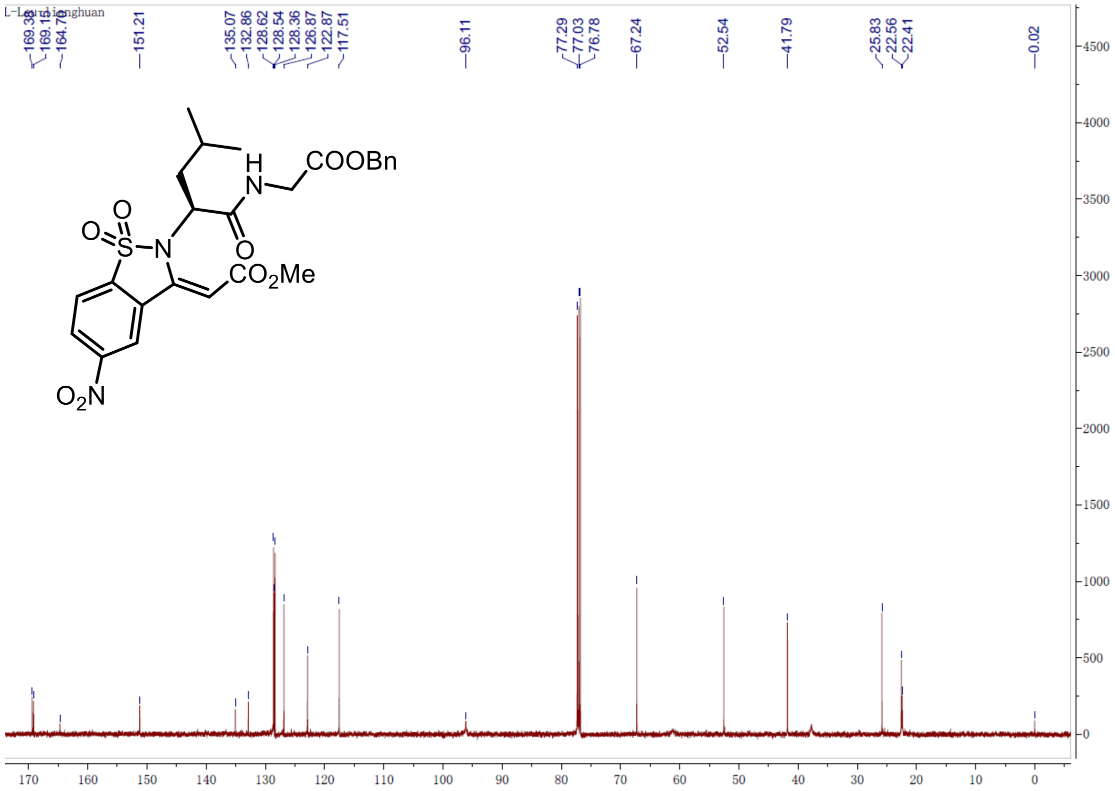

Supplementary Figure 214. ^{13}C NMR (100 MHz, $CDCl_3$) spectrum of compound **5ok**

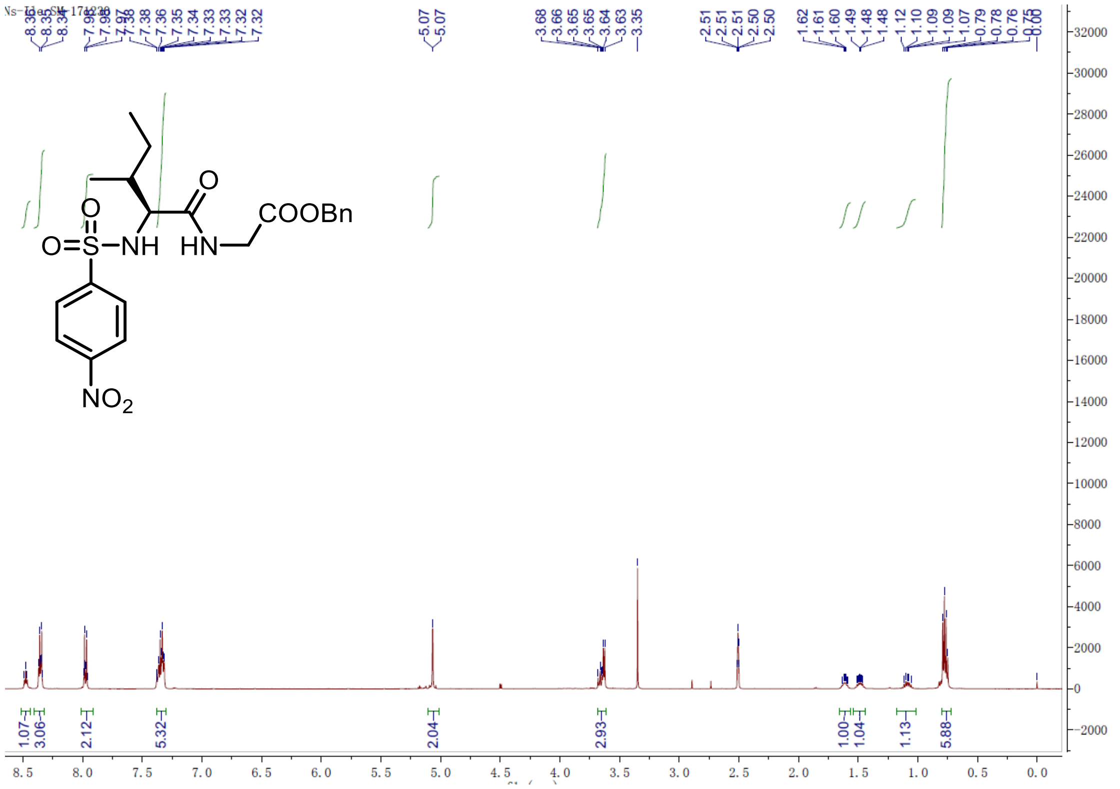

Supplementary Figure 215. ^{1}H NMR (400 MHz, DMSO) spectrum of compound **4p**

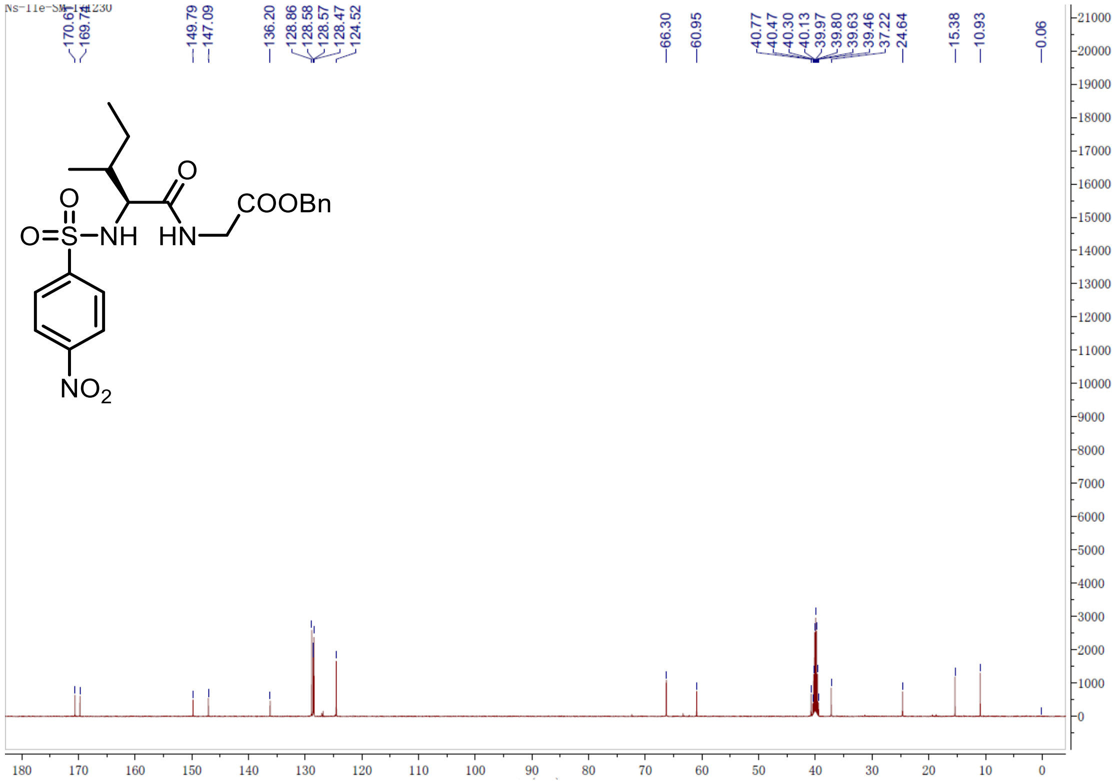

Supplementary Figure 216. ^{13}C NMR (100 MHz, DMSO) spectrum of compound **4p**

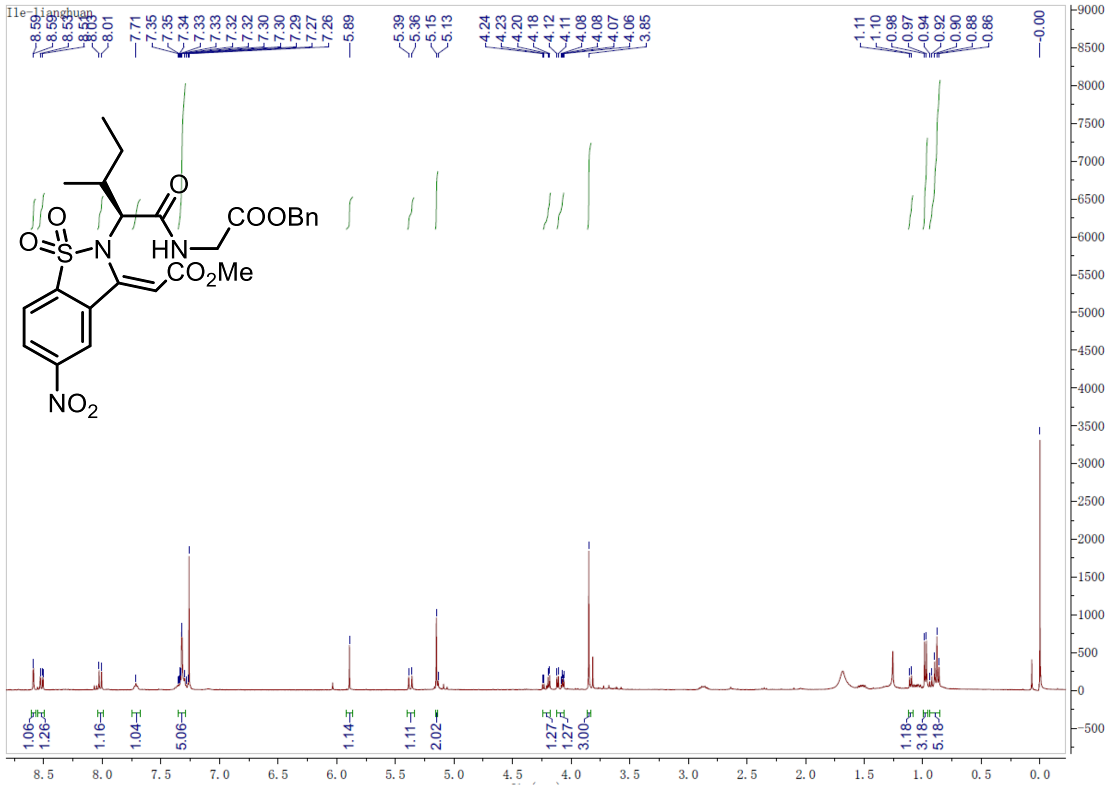

Supplementary Figure 217. ^{1}H NMR (400 MHz, $CDCl_3$) spectrum of compound **5pk**

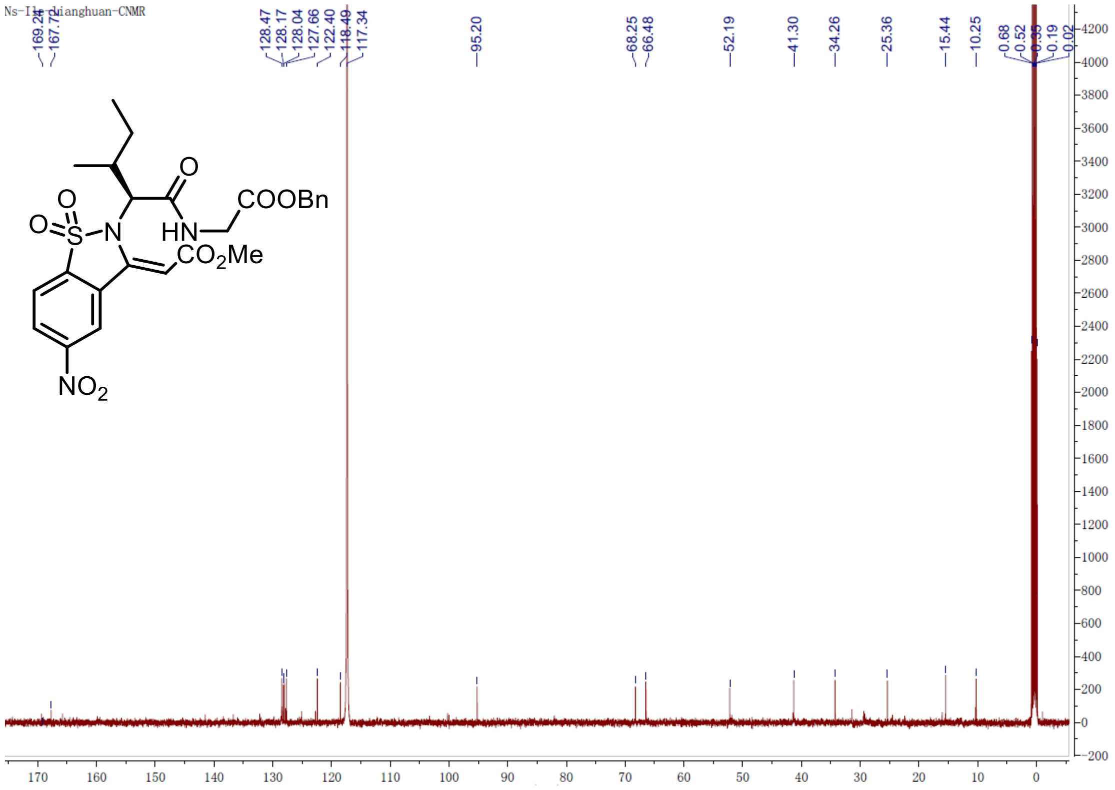

Supplementary Figure 218. ^{13}C NMR (100 MHz, $CDCl_3$) spectrum of compound **5pk**

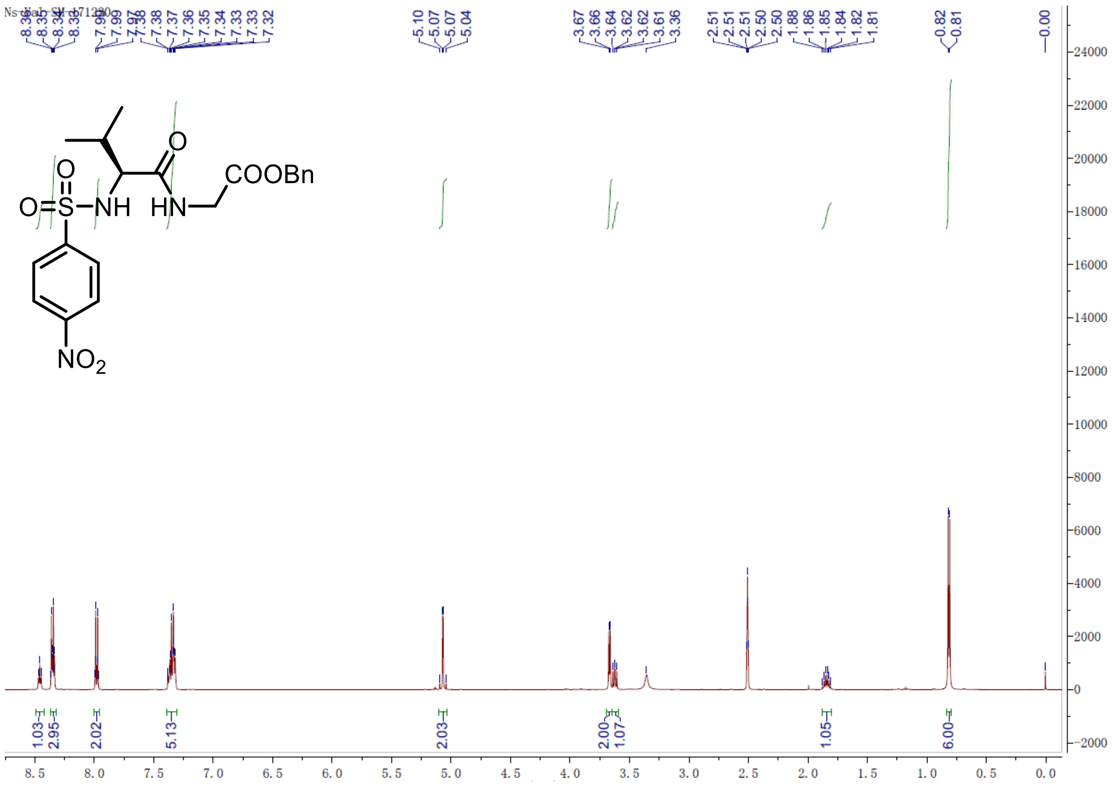

Supplementary Figure 219. ^{1}H NMR (400 MHz, DMSO) spectrum of compound **4q**

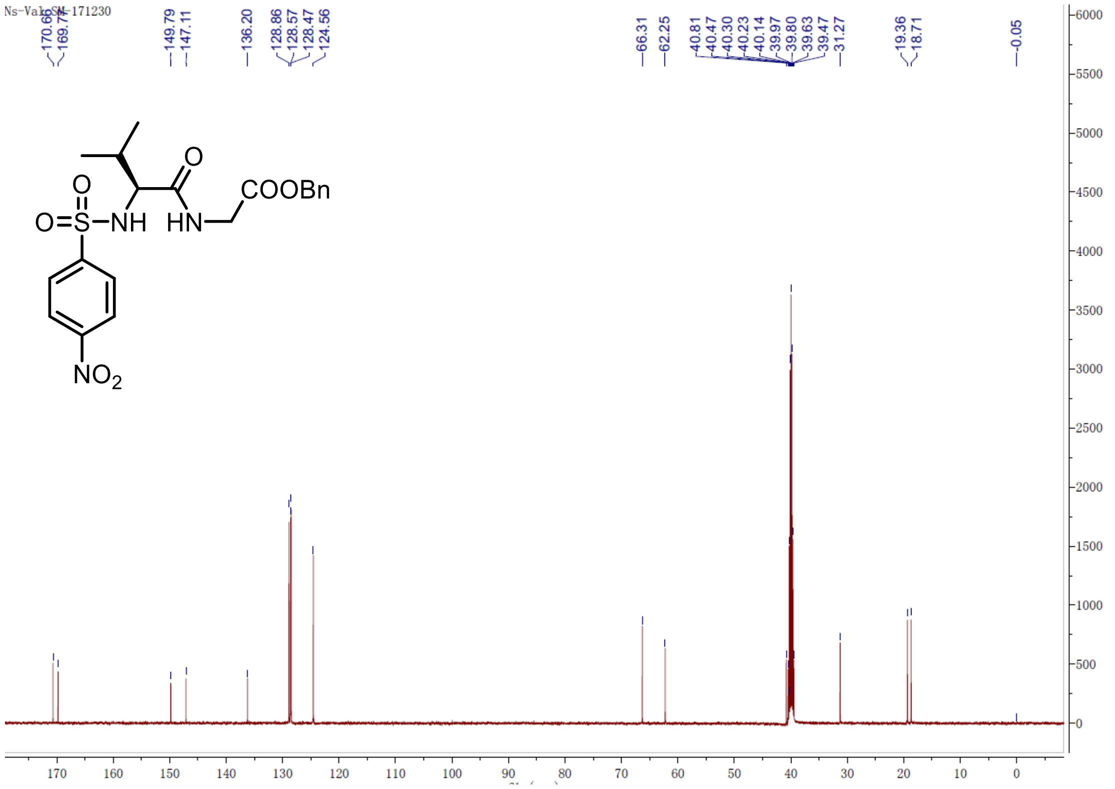

Supplementary Figure 220. ^{13}C NMR (100 MHz, DMSO) spectrum of compound **4q**

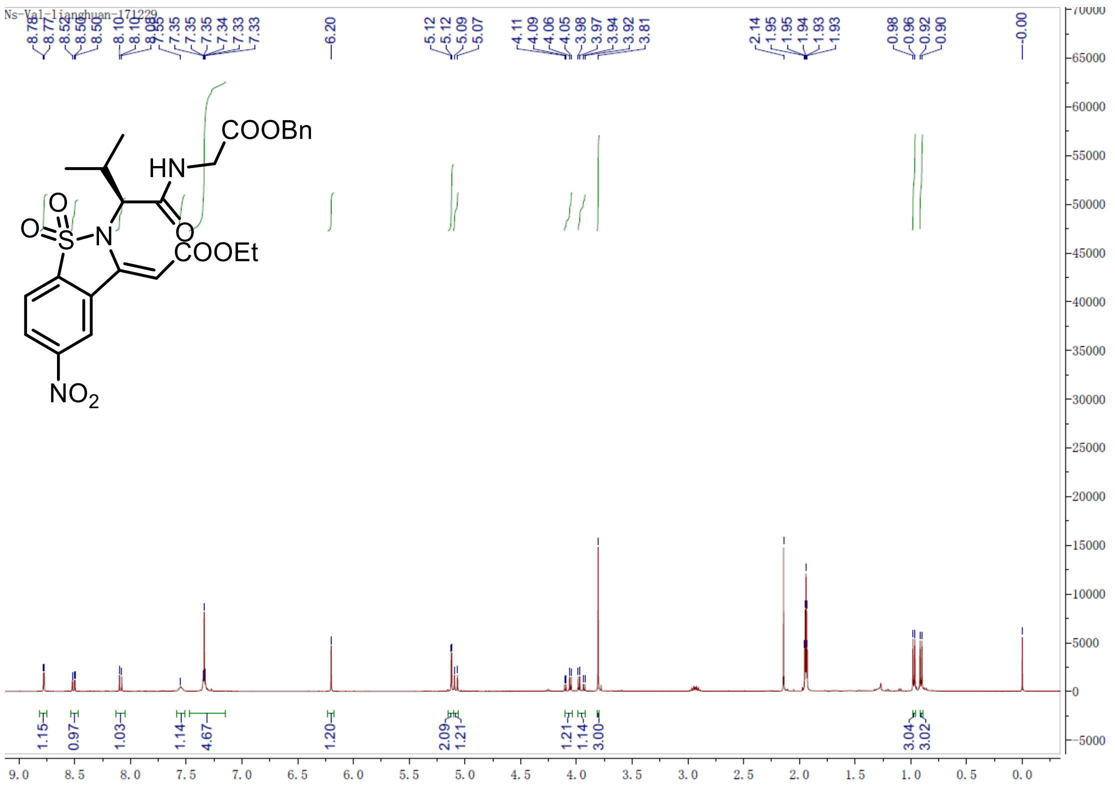

Supplementary Figure 221. ^{1}H NMR (400 MHz, CD_3CN) spectrum of compound **5qk**

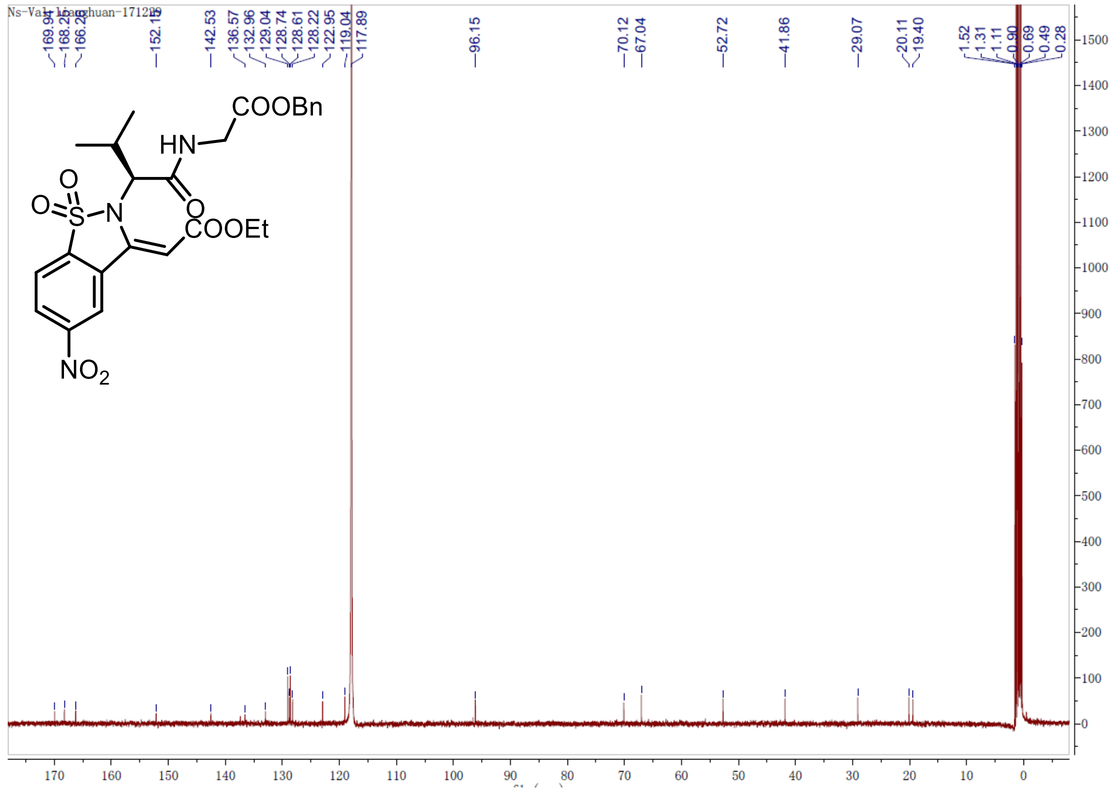

Supplementary Figure 222. ^{13}C NMR (100 MHz, CD_3CN) spectrum of compound **5qk**

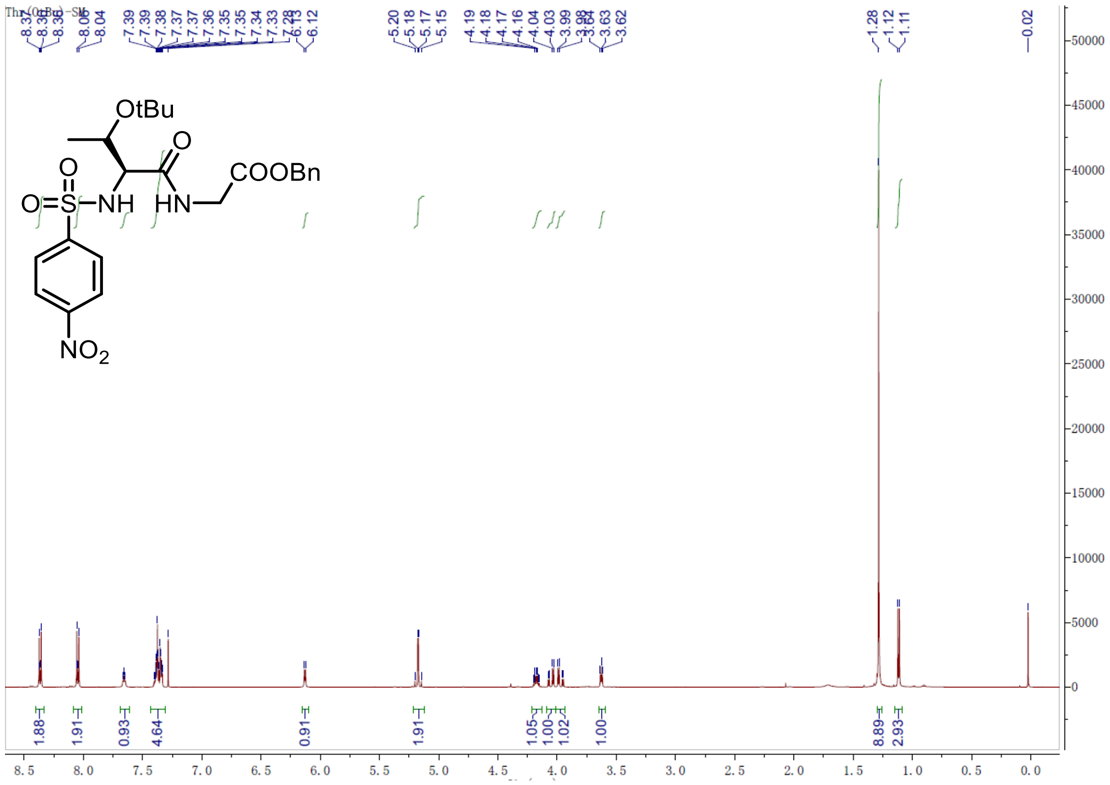

Supplementary Figure 223. ^{1}H NMR (400 MHz, $CDCl_3$) spectrum of compound **4r**

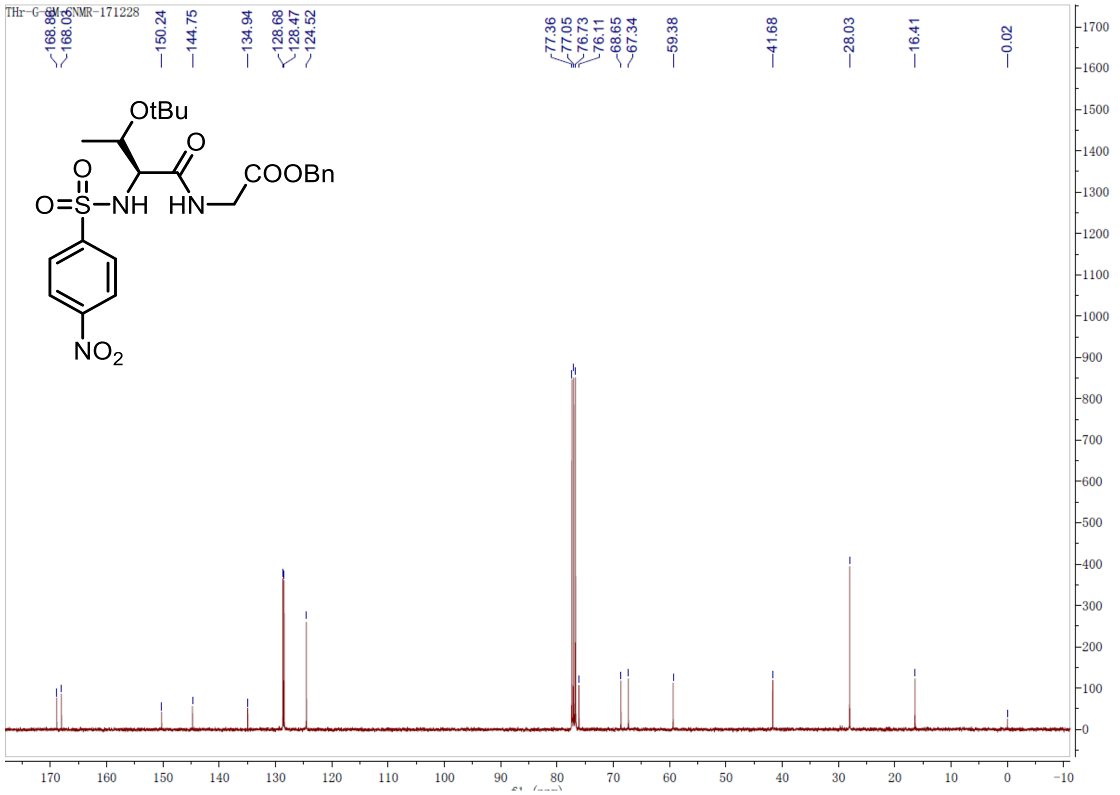

Supplementary Figure 224. ^{13}C NMR (100 MHz, $CDCl_3$) spectrum of compound **4r**

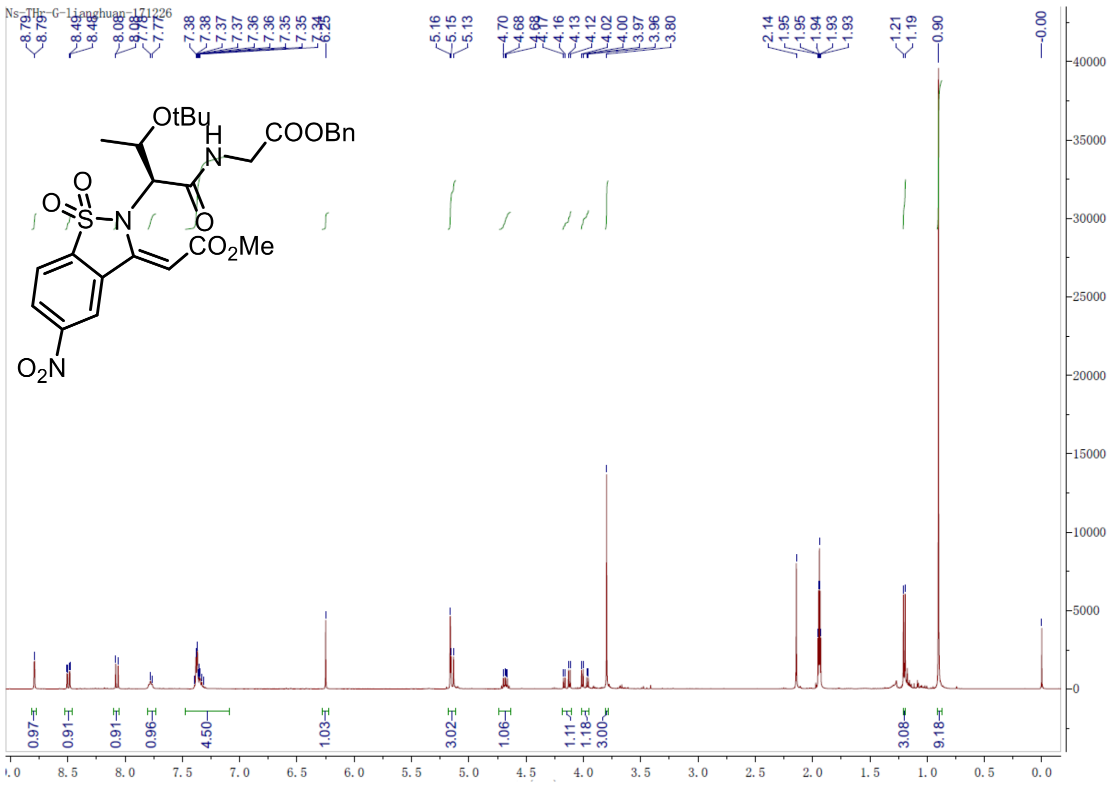

Supplementary Figure 225. ^{1}H NMR (400 MHz, CD_3CN) spectrum of compound **5rk**

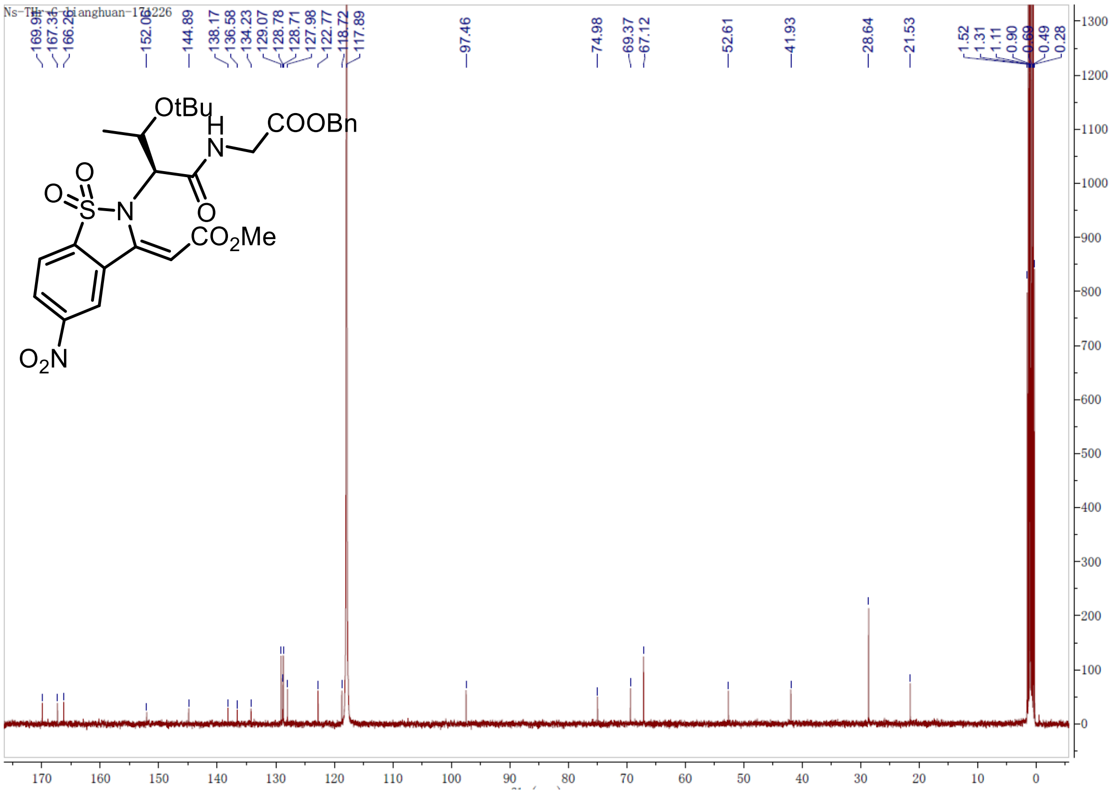

Supplementary Figure 226. ^{13}C NMR (100 MHz, CD_3CN) spectrum of compound **5rk**

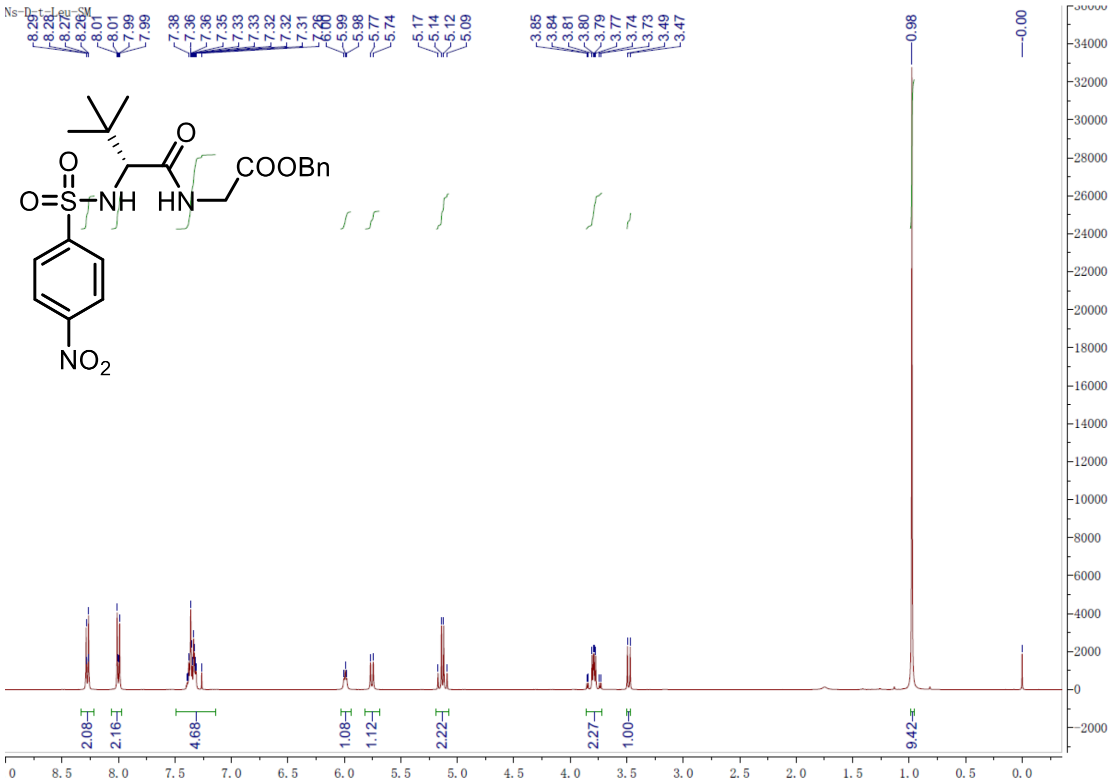

Supplementary Figure 227. ^{1}H NMR (400 MHz, $CDCl_3$) spectrum of compound **4s**

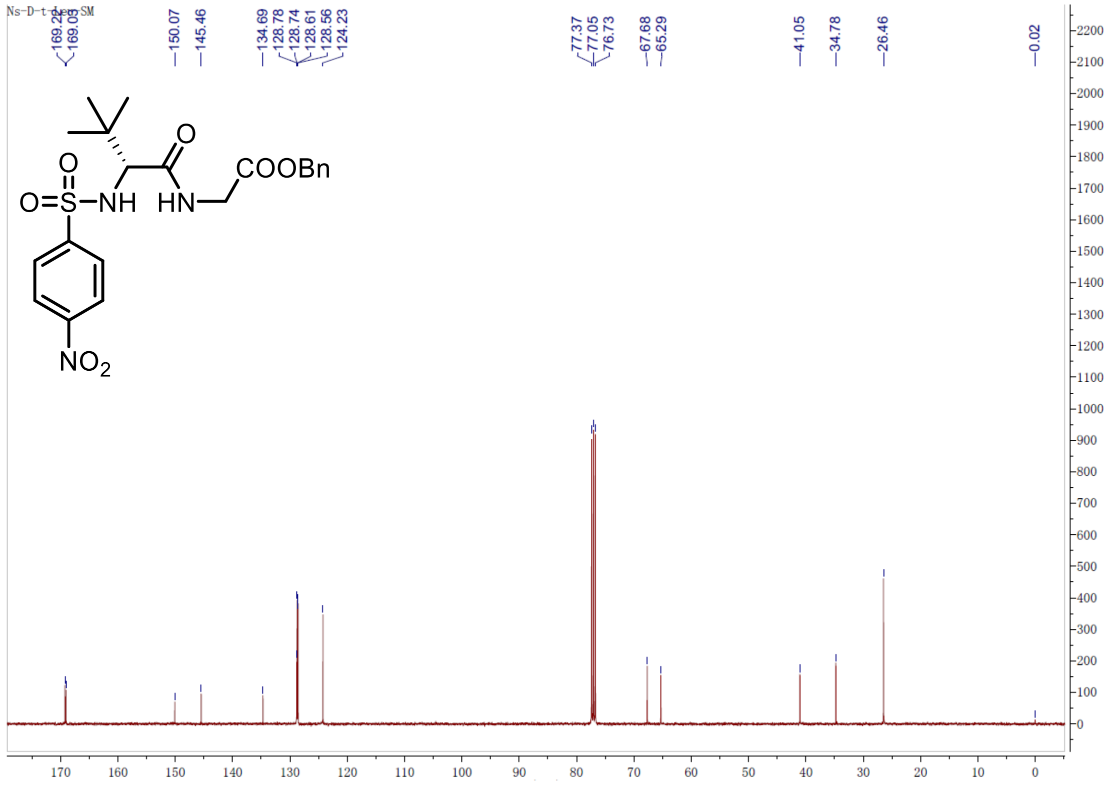

Supplementary Figure 228. ^{13}C NMR (100 MHz, $CDCl_3$) spectrum of compound **4s**

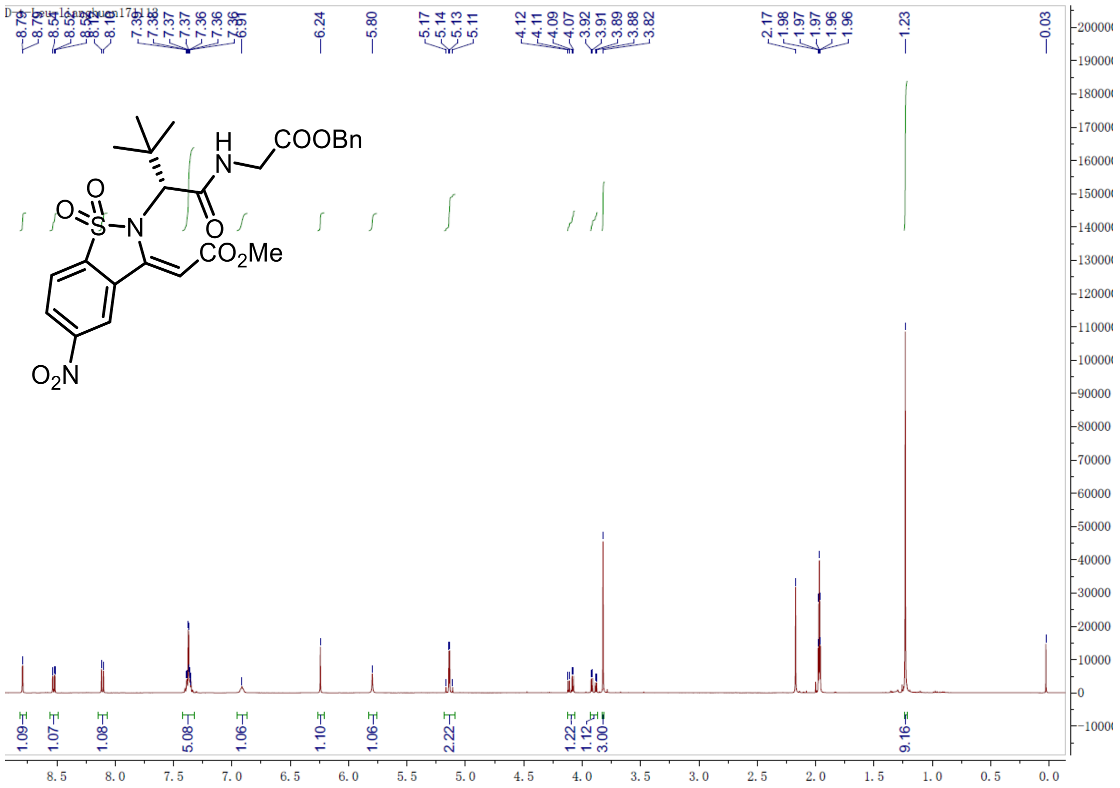

Supplementary Figure 229. ^{1}H NMR (400 MHz, CD_3CN) spectrum of compound **5sk**

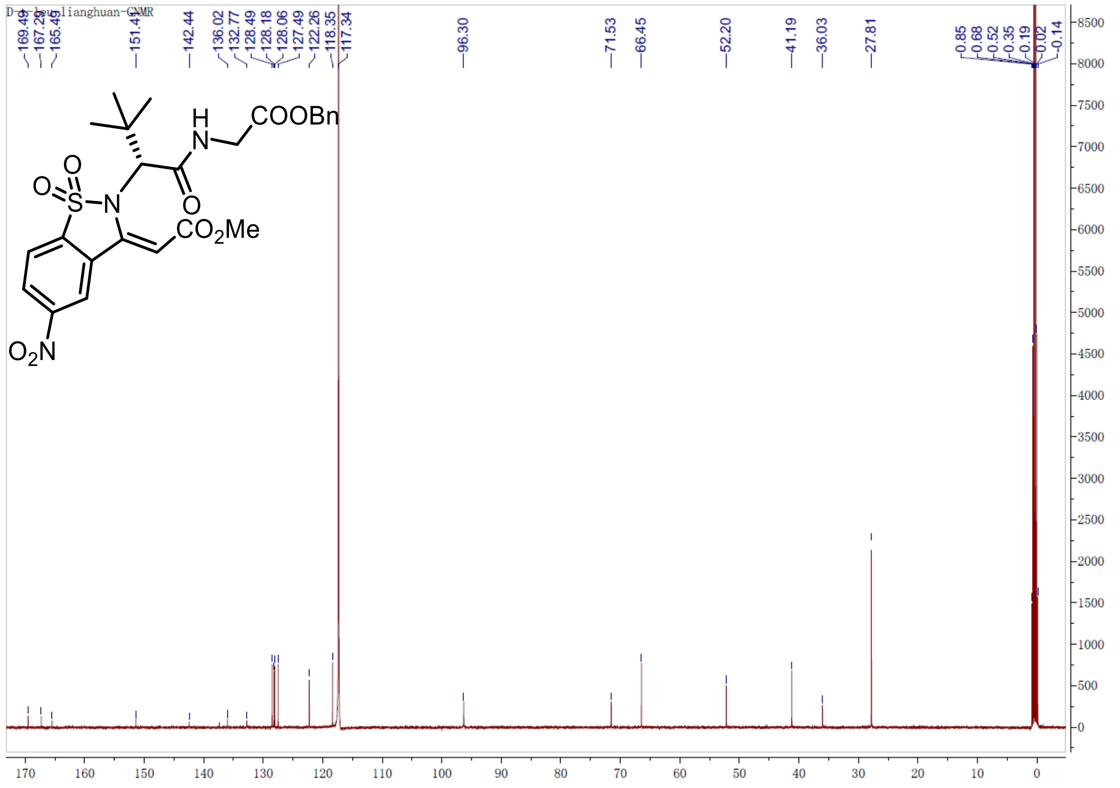

Supplementary Figure 230. ^{13}C NMR (100 MHz, CD_3CN) spectrum of compound **5sk**

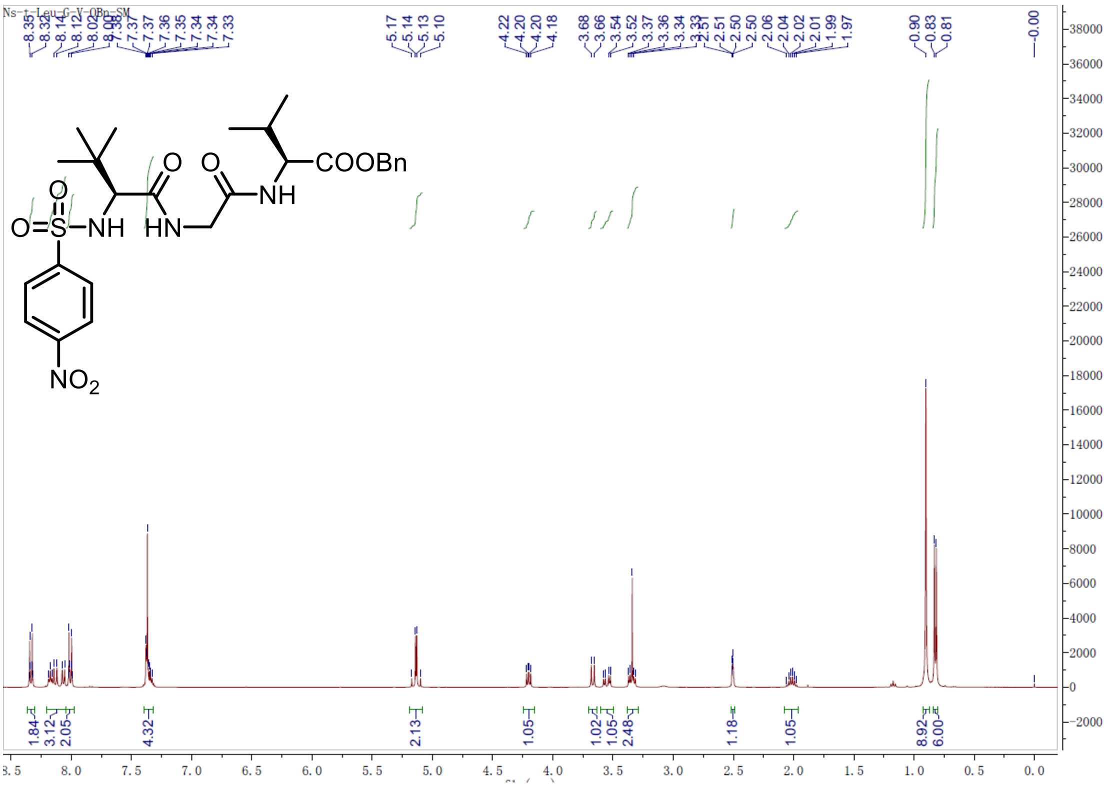

Supplementary Figure 231. ^{1}H NMR (400 MHz, $CDCl_3$) spectrum of compound **4t**

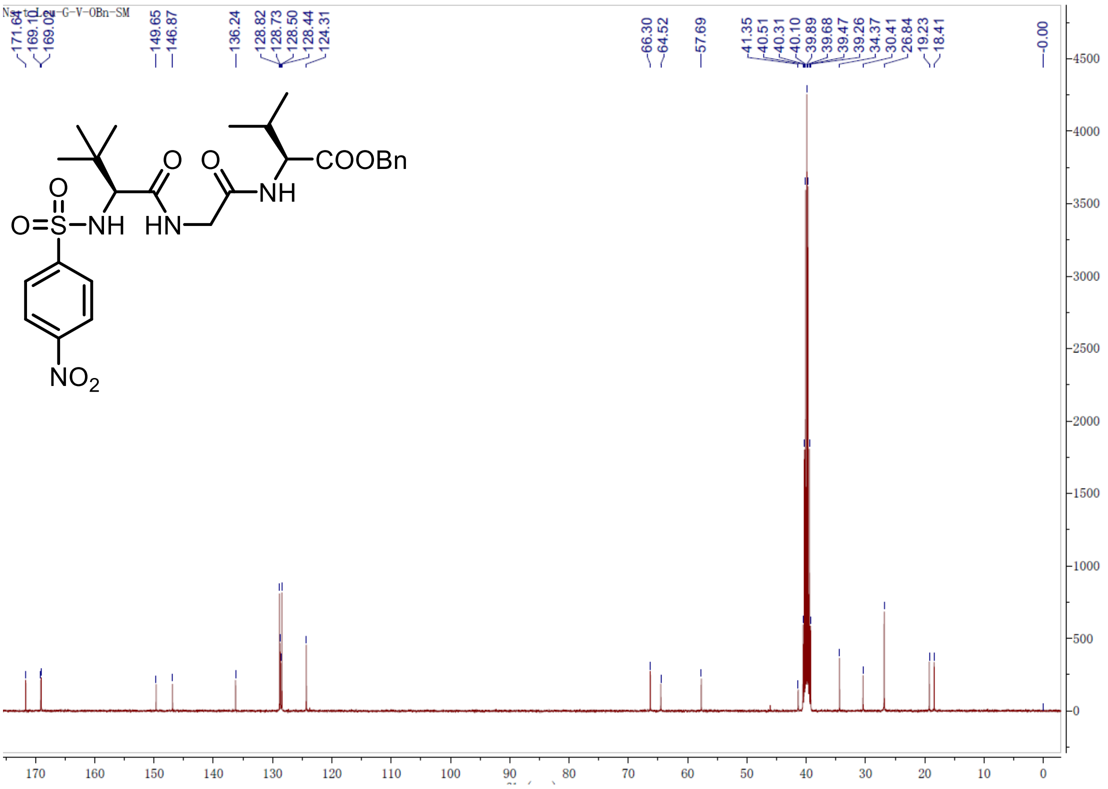

Supplementary Figure 232. ^{13}C NMR (100 MHz, $CDCl_3$) spectrum of compound **4t**

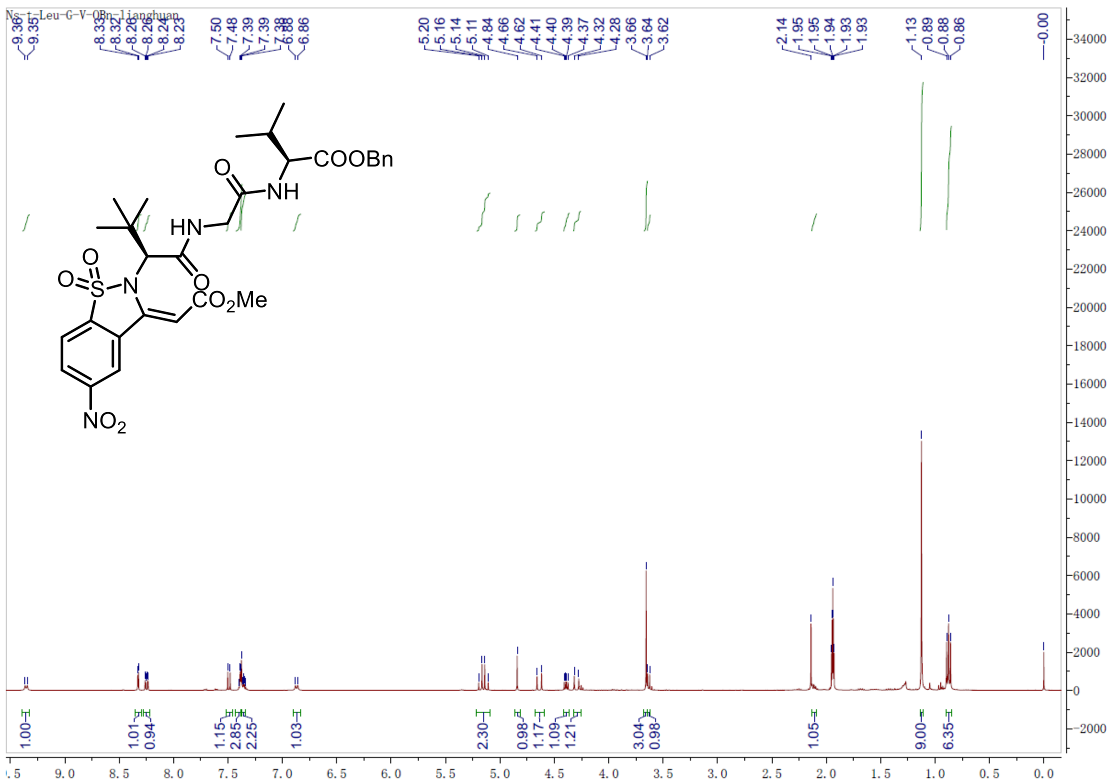

Supplementary Figure 233. ^{1}H NMR (400 MHz, CD_3CN) spectrum of compound **5tk**

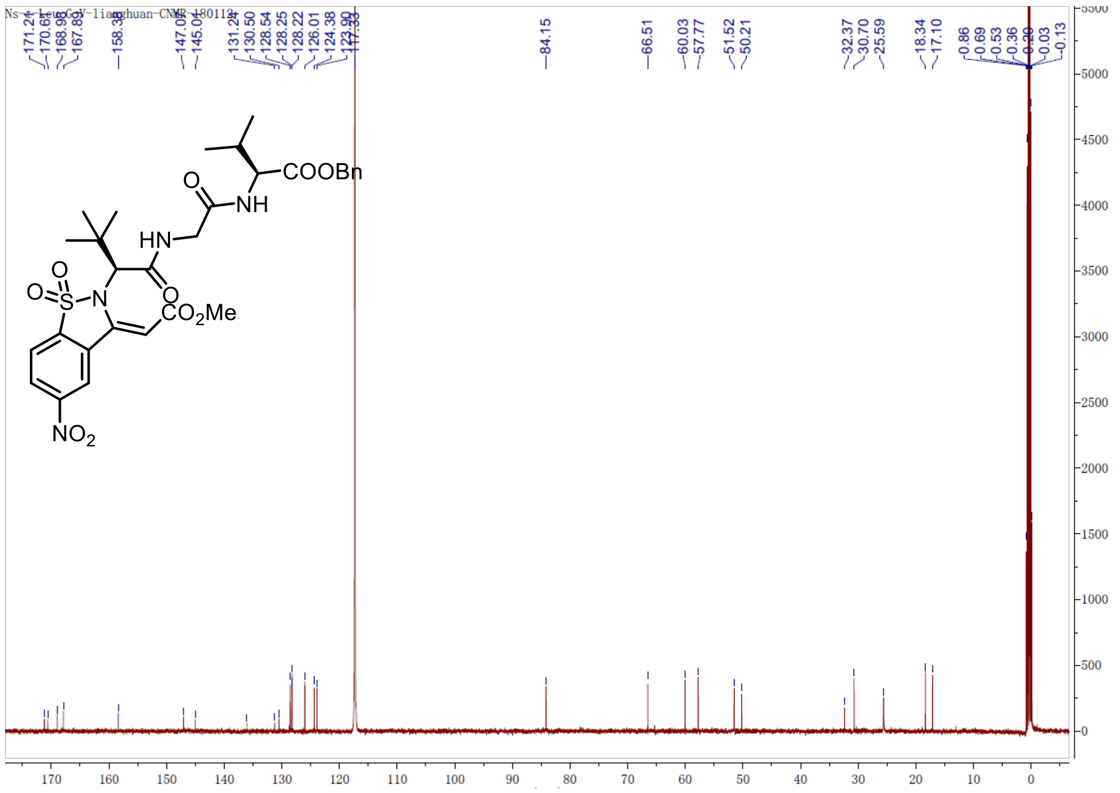

Supplementary Figure 234. ^{13}C NMR (100 MHz, CD_3CN) spectrum of compound **5tk**

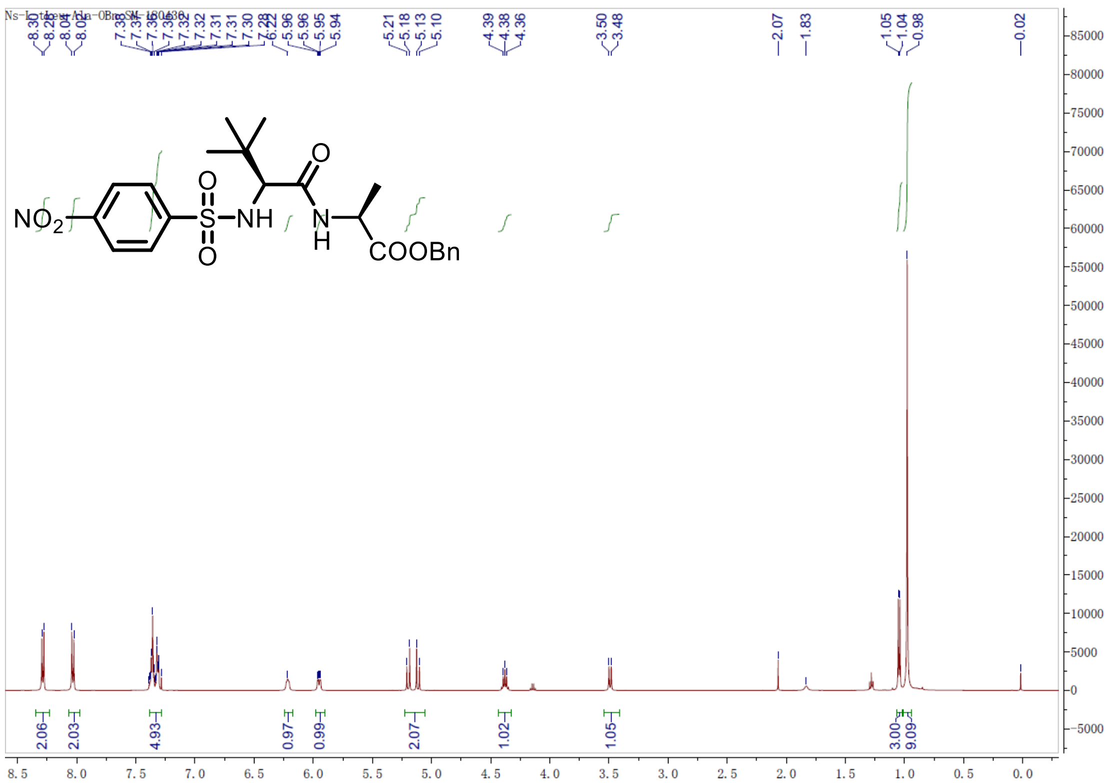

Supplementary Figure 235. ^{1}H NMR (400 MHz, $CDCl_3$) spectrum of compound **4u**

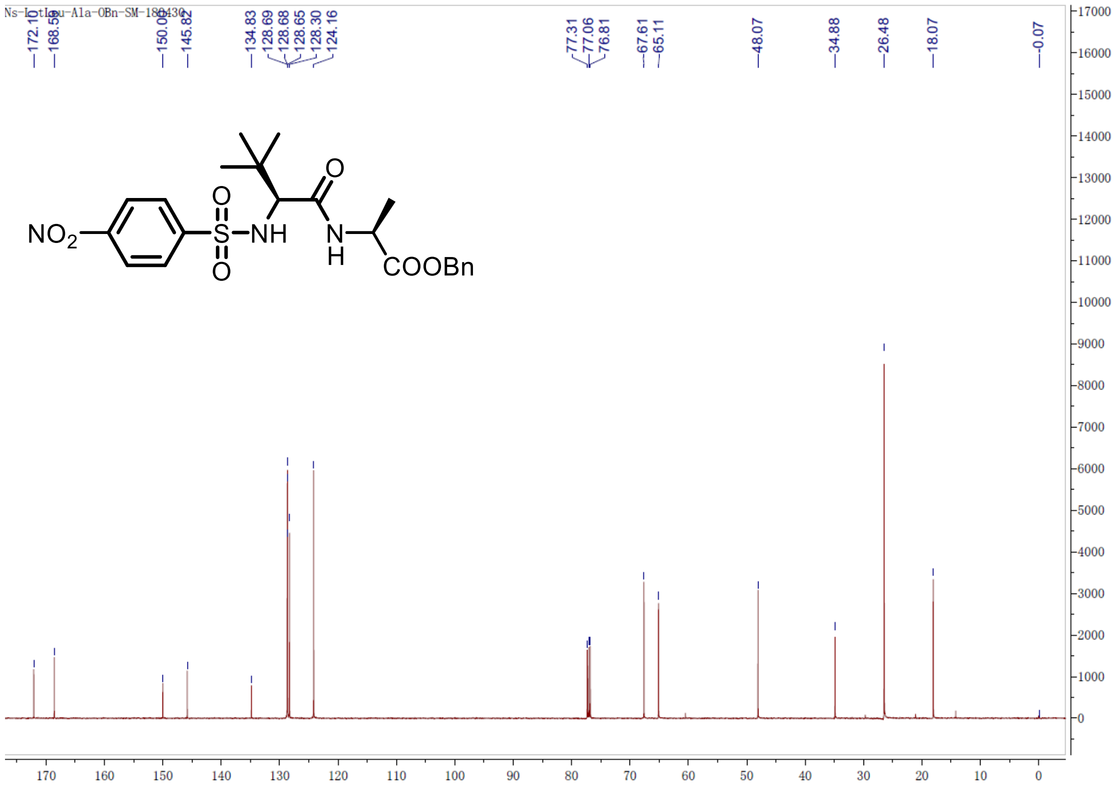

Supplementary Figure 236. ^{13}C NMR (100 MHz, $CDCl_3$) spectrum of compound **4u**

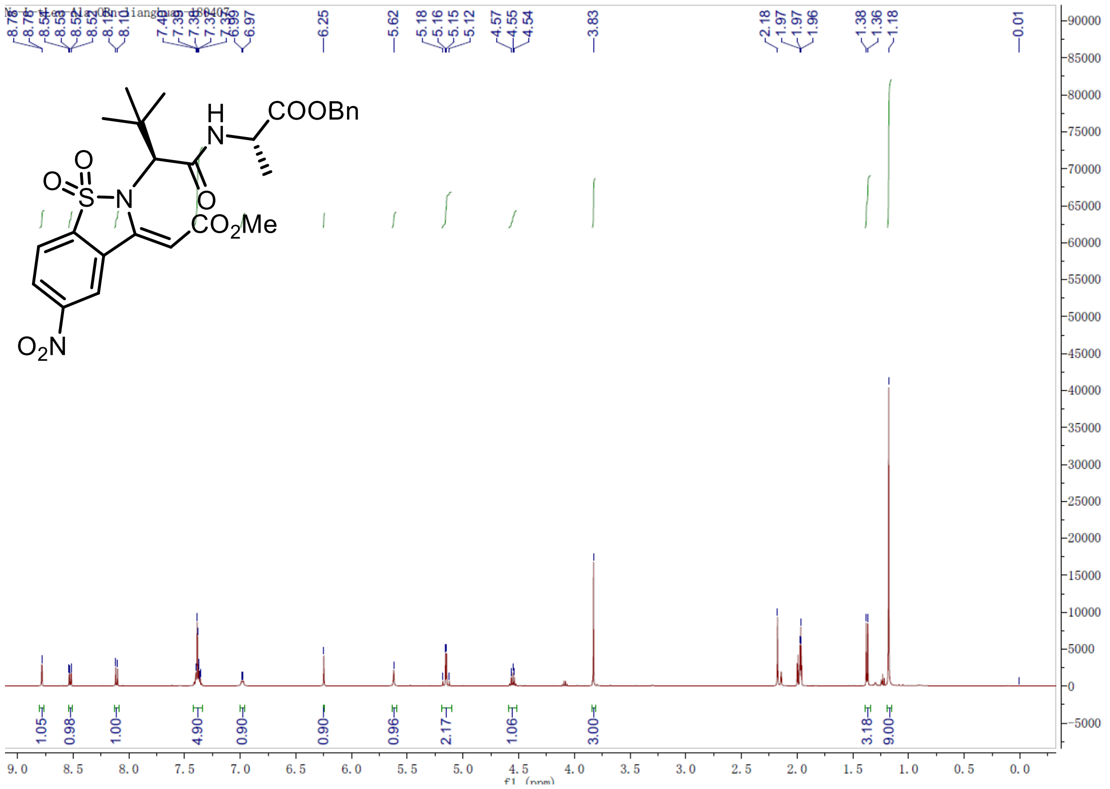

Supplementary Figure 237. ^{1}H NMR (400 MHz, CD_3CN) spectrum of compound **5uk**

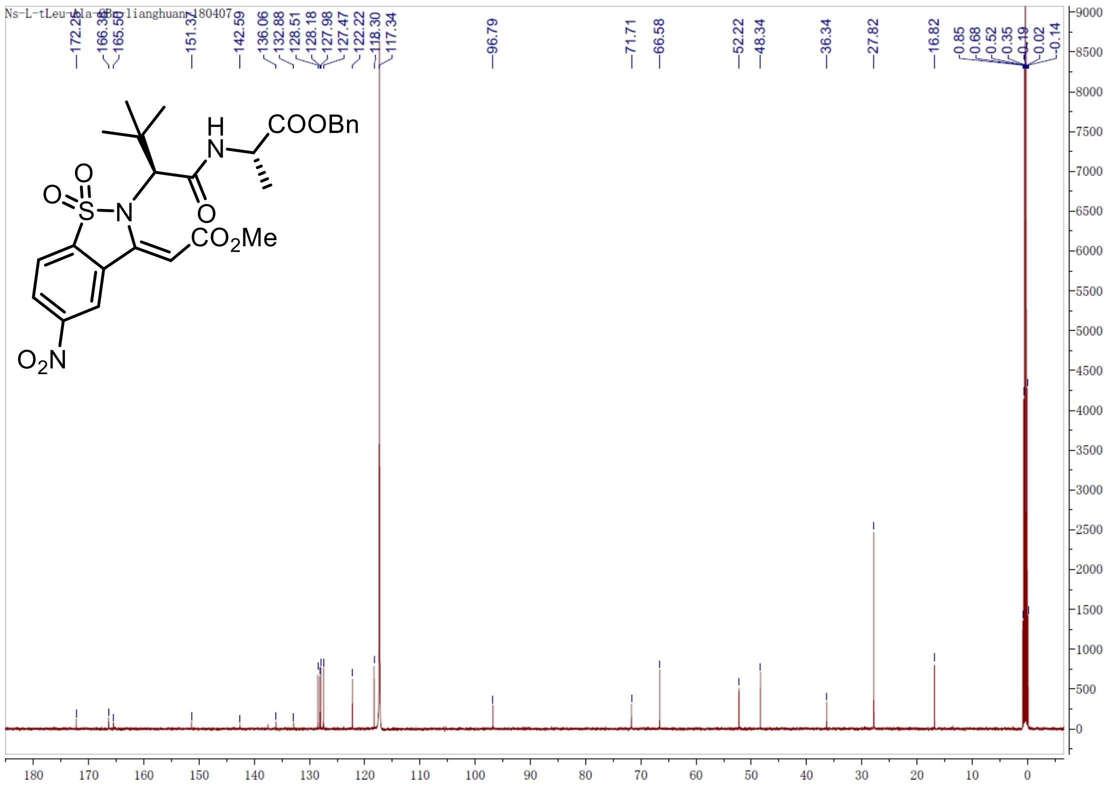

Supplementary Figure 238. ^{13}C NMR (100 MHz, CD_3CN) spectrum of compound **5uk**

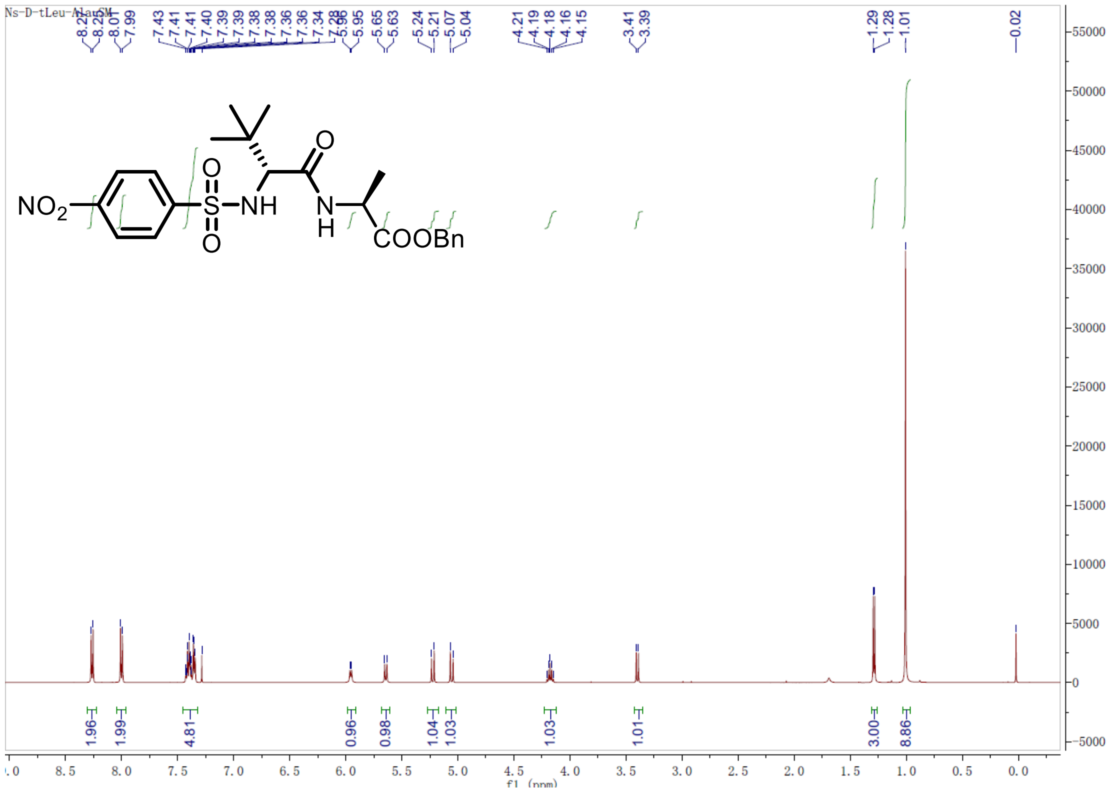

Supplementary Figure239. ^{1}H NMR (400 MHz, $CDCl_3$) spectrum of compound **4v**

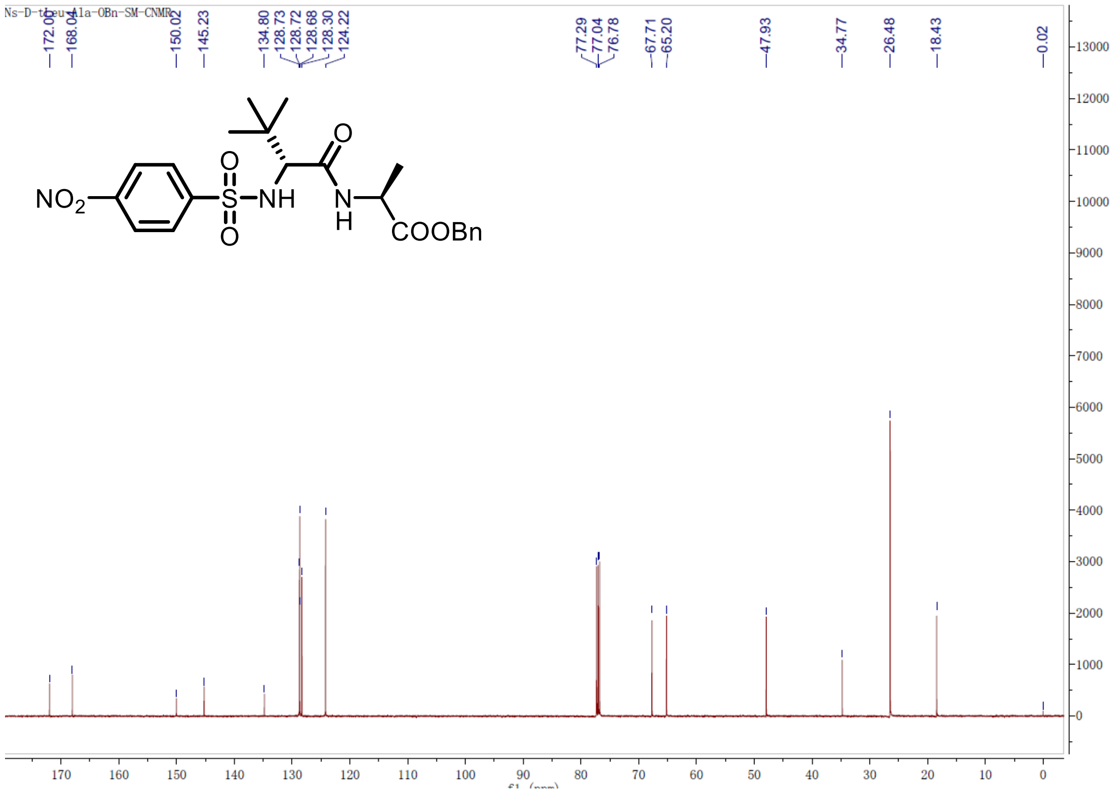

Supplementary Figure 240. ^{13}C NMR (100 MHz, $CDCl_3$) spectrum of compound **4v**

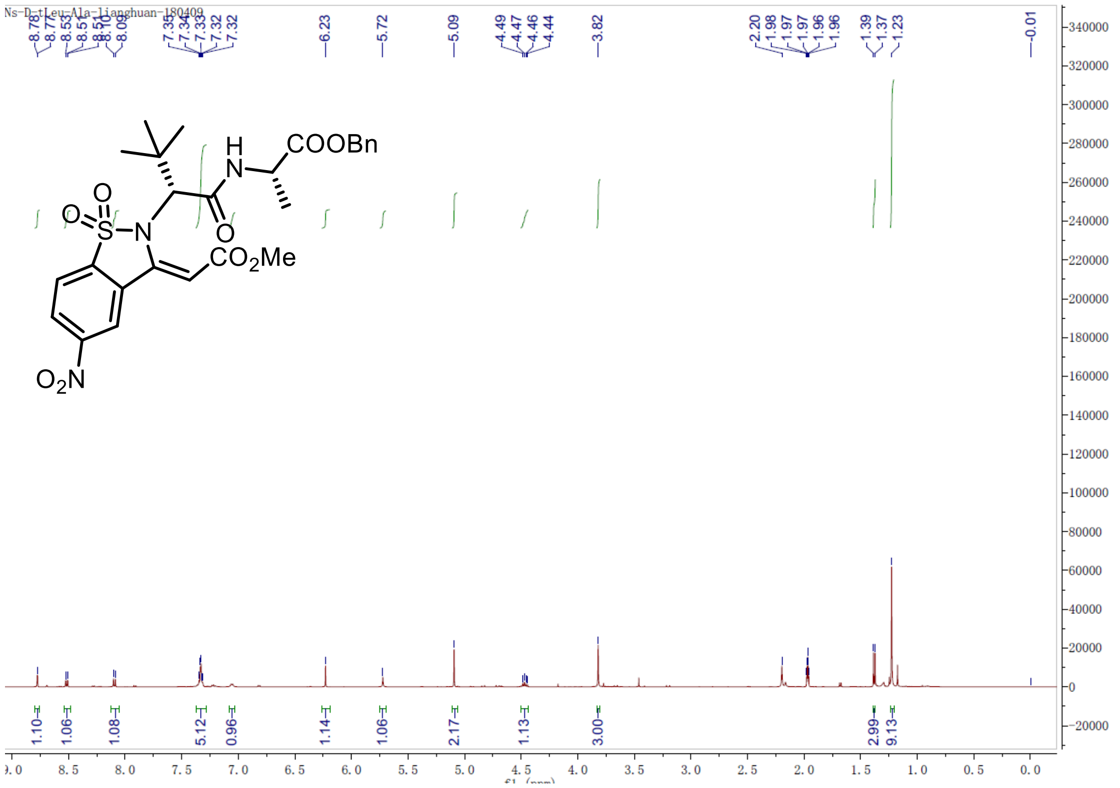

Supplementary Figure 241. ^{1}H NMR (400 MHz, CD_3CN) spectrum of compound **5vk**

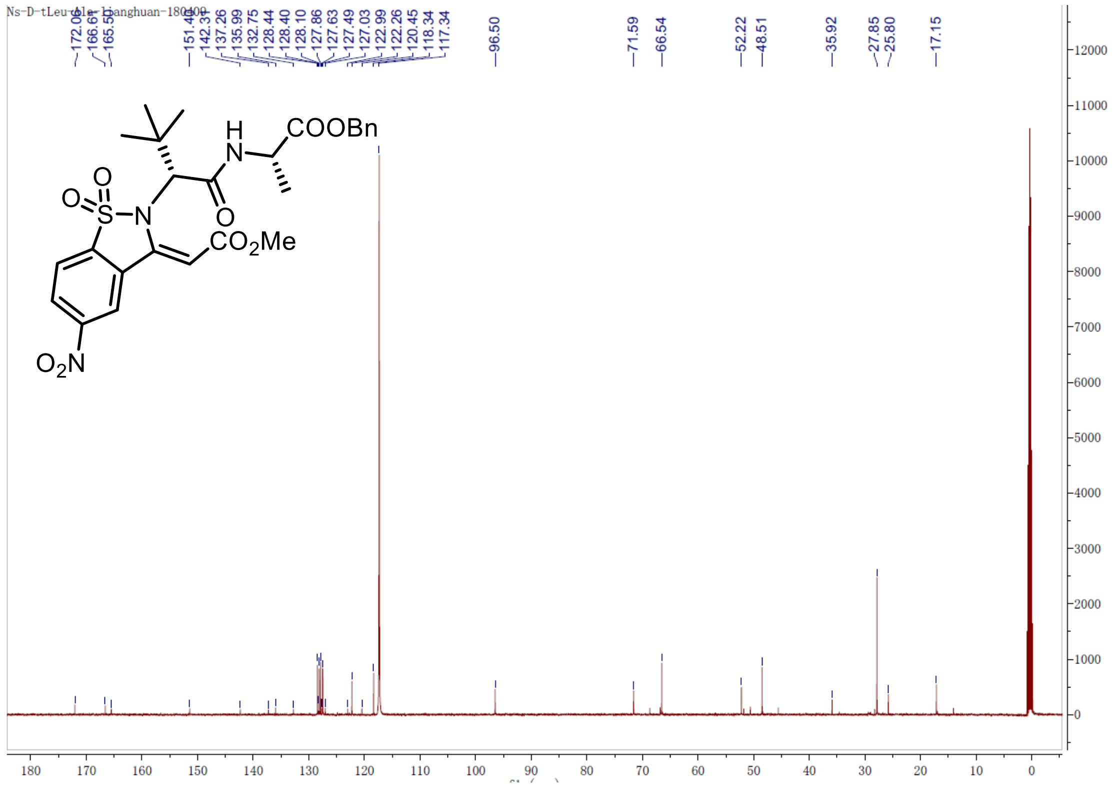

Supplementary Figure 242. ^{13}C NMR (100 MHz, CD_3CN) spectrum of compound **5vk**

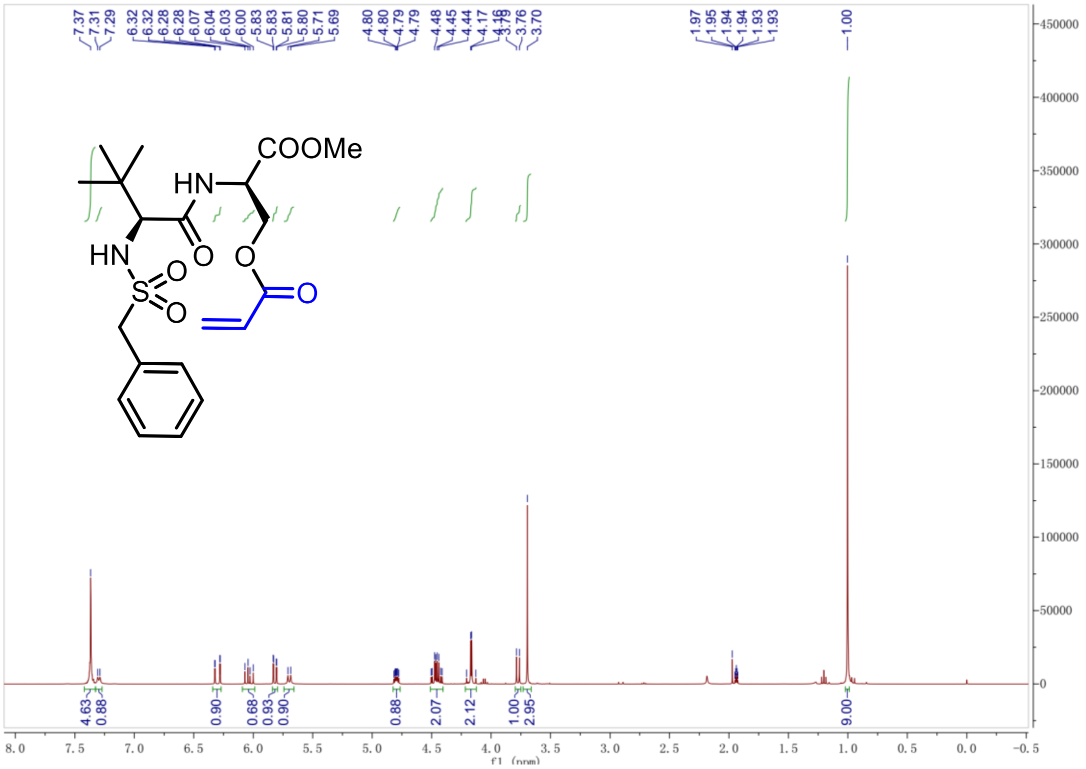

Supplementary Figure 243. ^{1}H NMR (400 MHz, CD_3CN) spectrum of compound **8a**

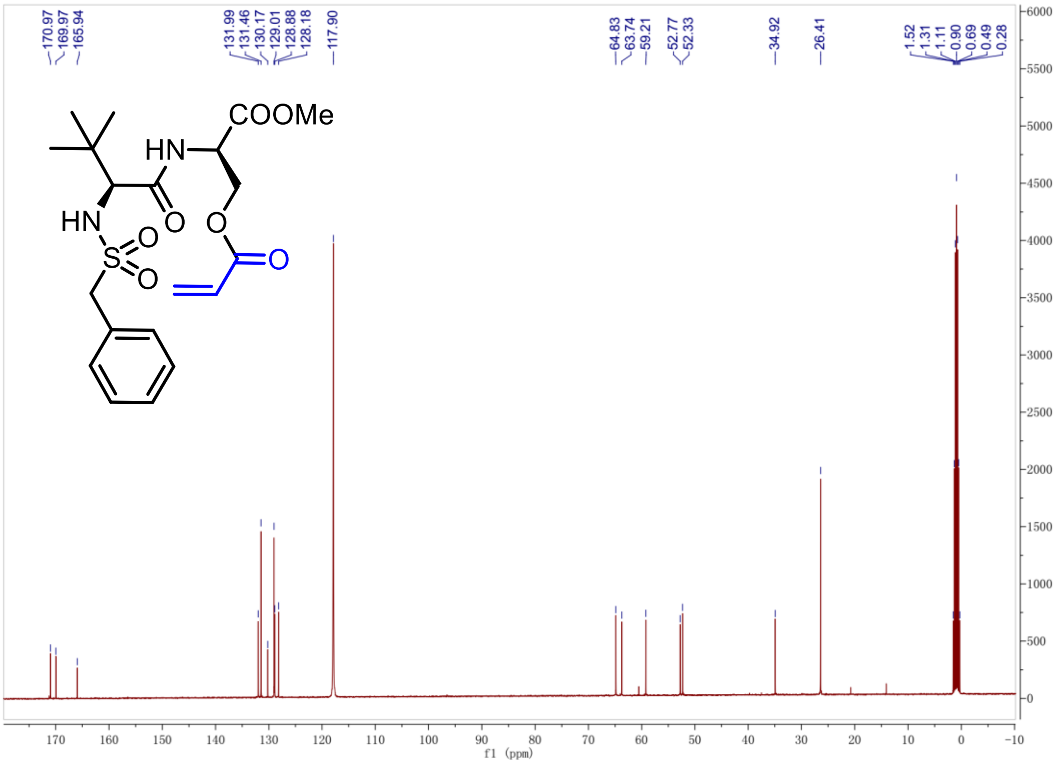

Supplementary Figure 244. ^{13}C NMR (100 MHz, CD_3CN) spectrum of compound **8a**

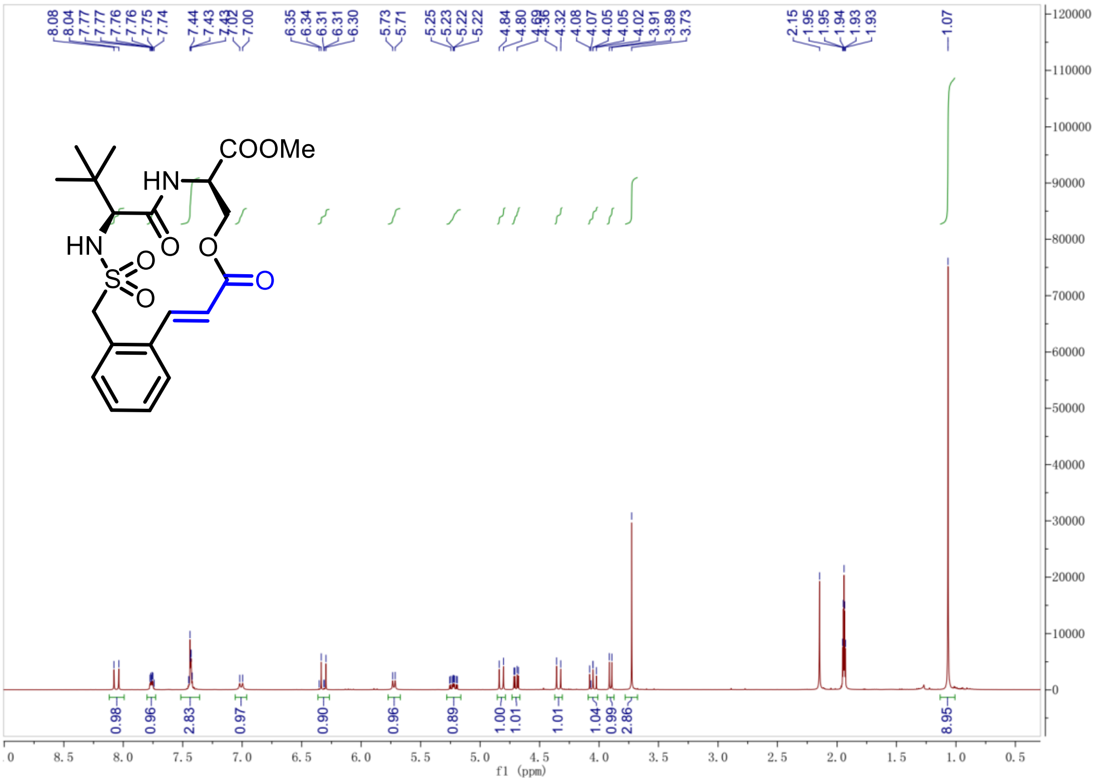

Supplementary Figure 245. ^{1}H NMR (400 MHz, CD_3CN) spectrum of compound **9a**

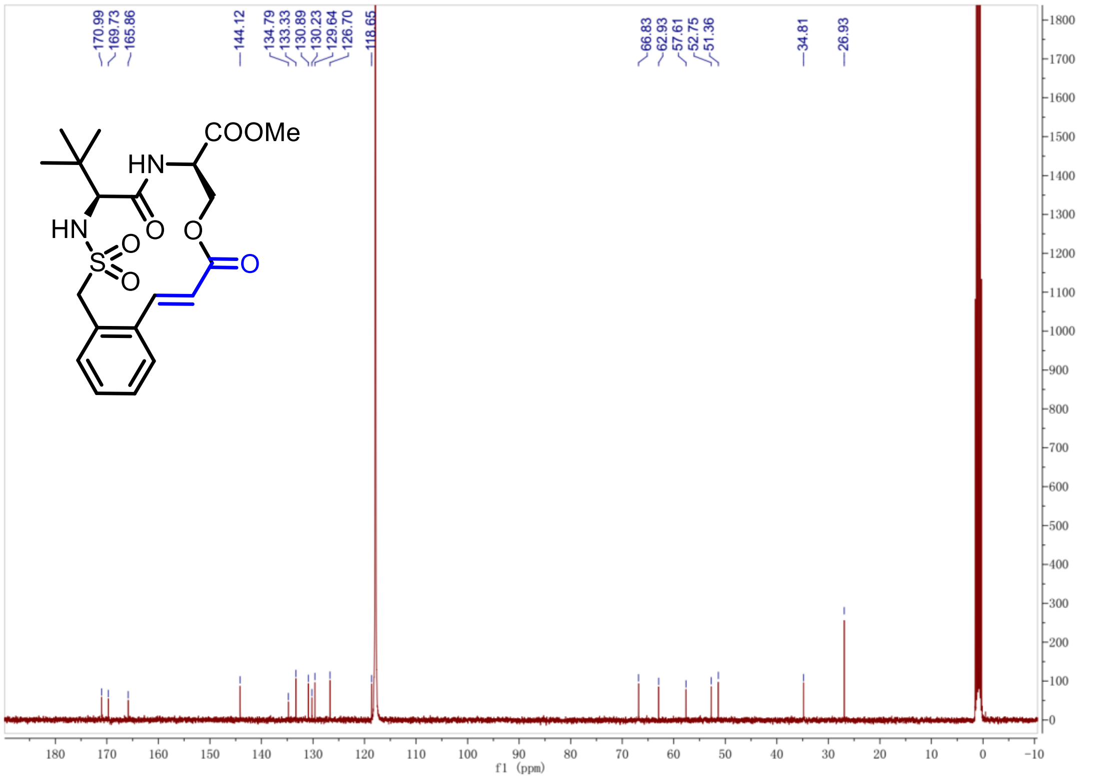

Supplementary Figure 246. ^{13}C NMR (100 MHz, CD_3CN)) spectrum of compound **9a**

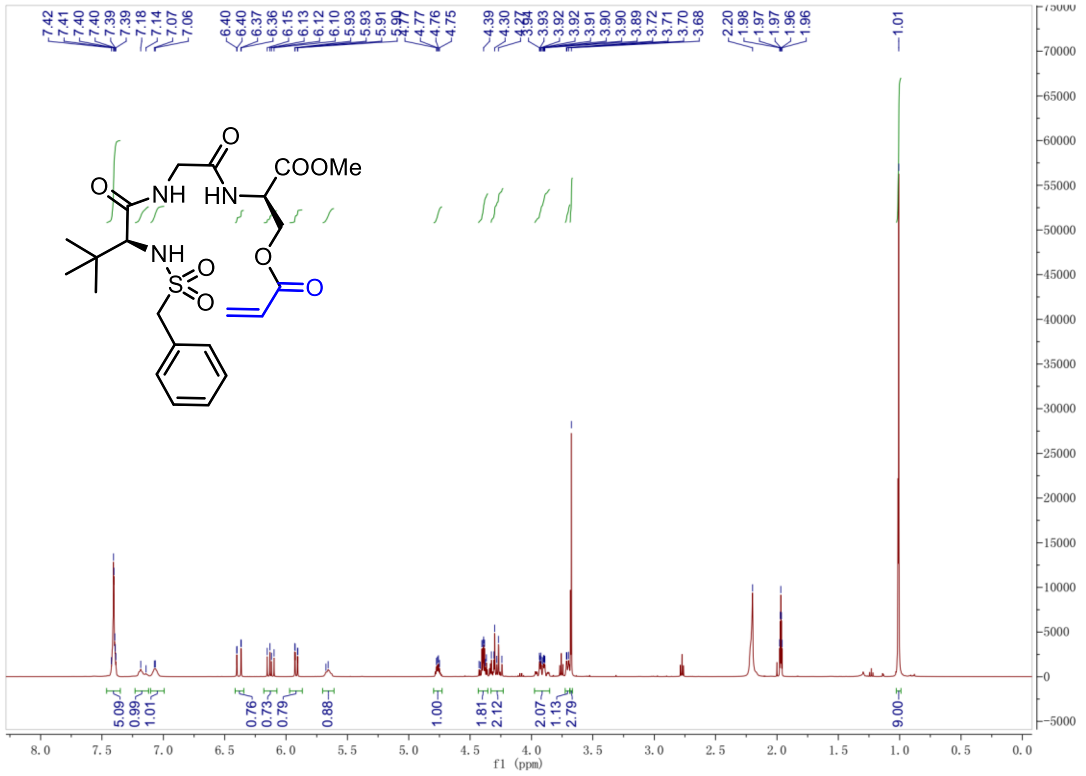

Supplementary Figure 247. ^{1}H NMR (500 MHz, CD_3CN)) spectrum of compound **8b**

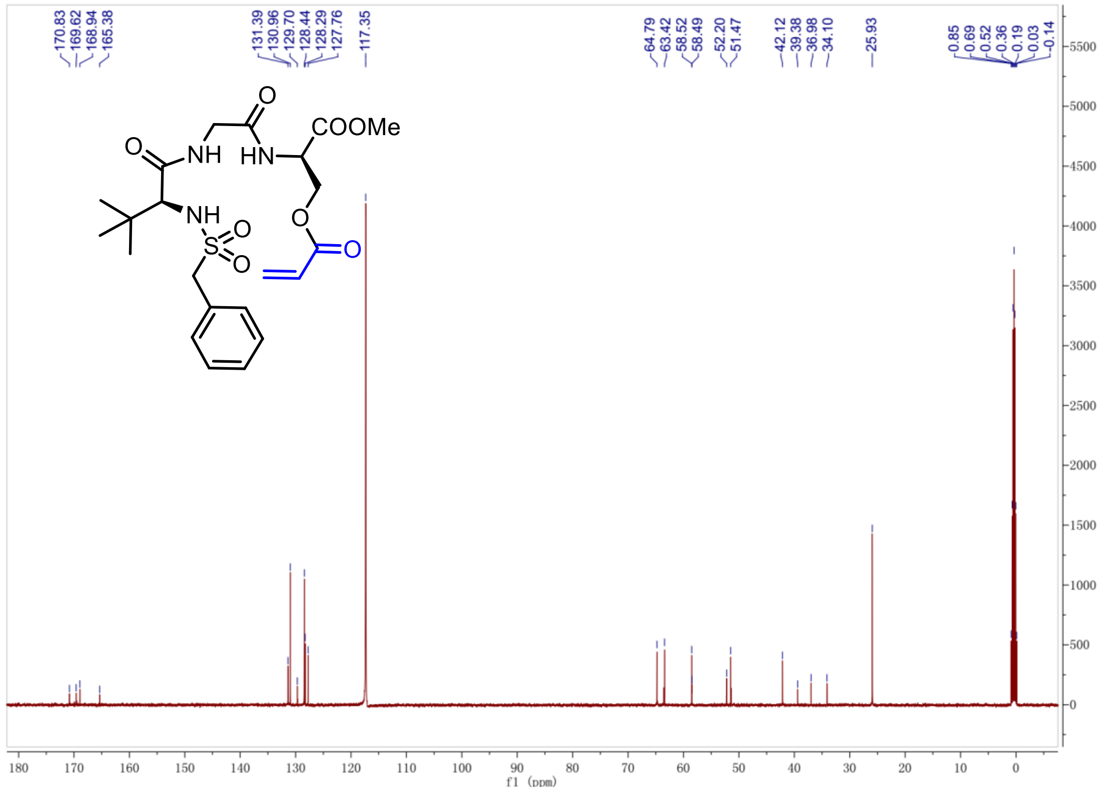

Supplementary Figure 248 ^{13}C NMR (125 MHz, CD_3CN)) spectrum of compound **8b**

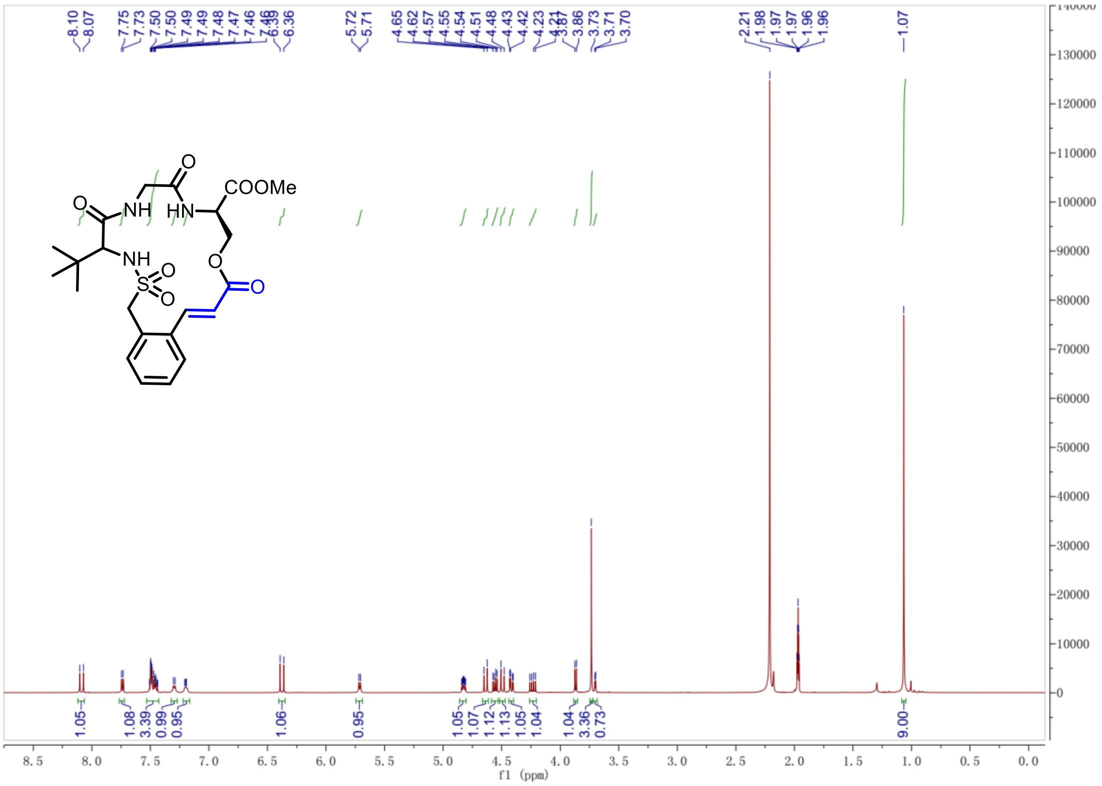

Supplementary Figure 249. ^{1}H NMR (500 MHz, CD_3CN) spectrum of compound **9b**

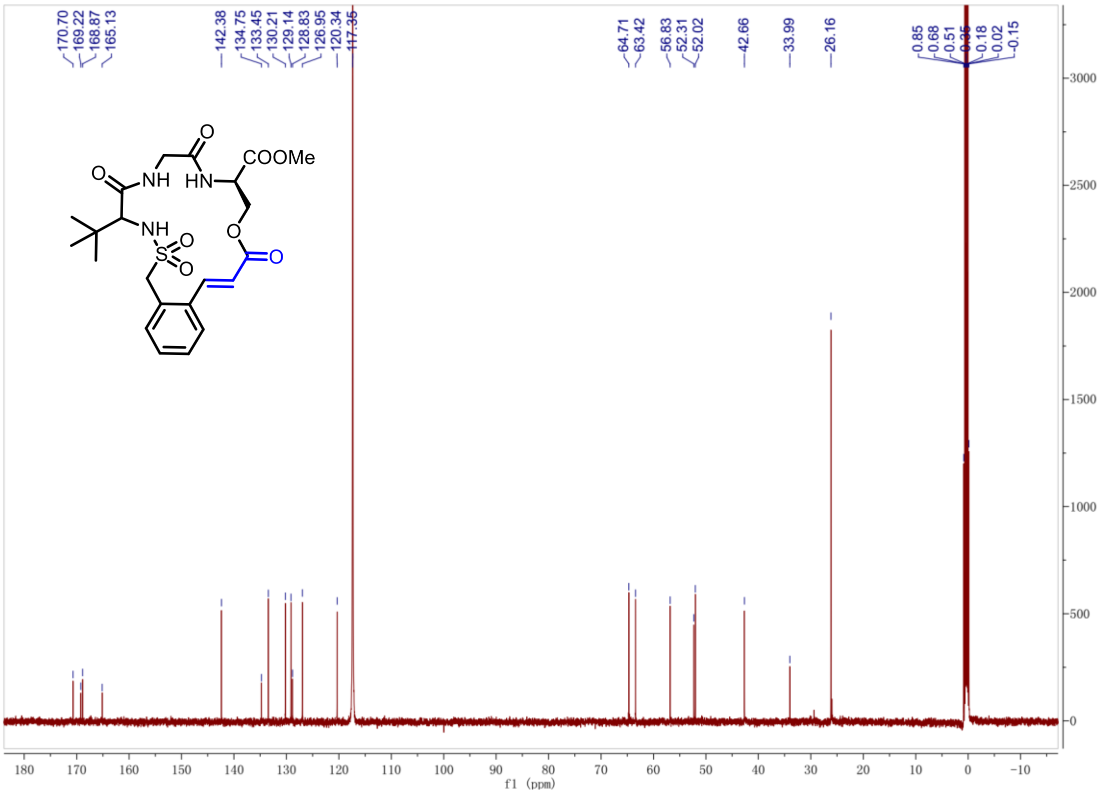

Supplementary Figure 250. ^{13}C NMR (125MHz, CD_3CN) spectrum of compound **9b**

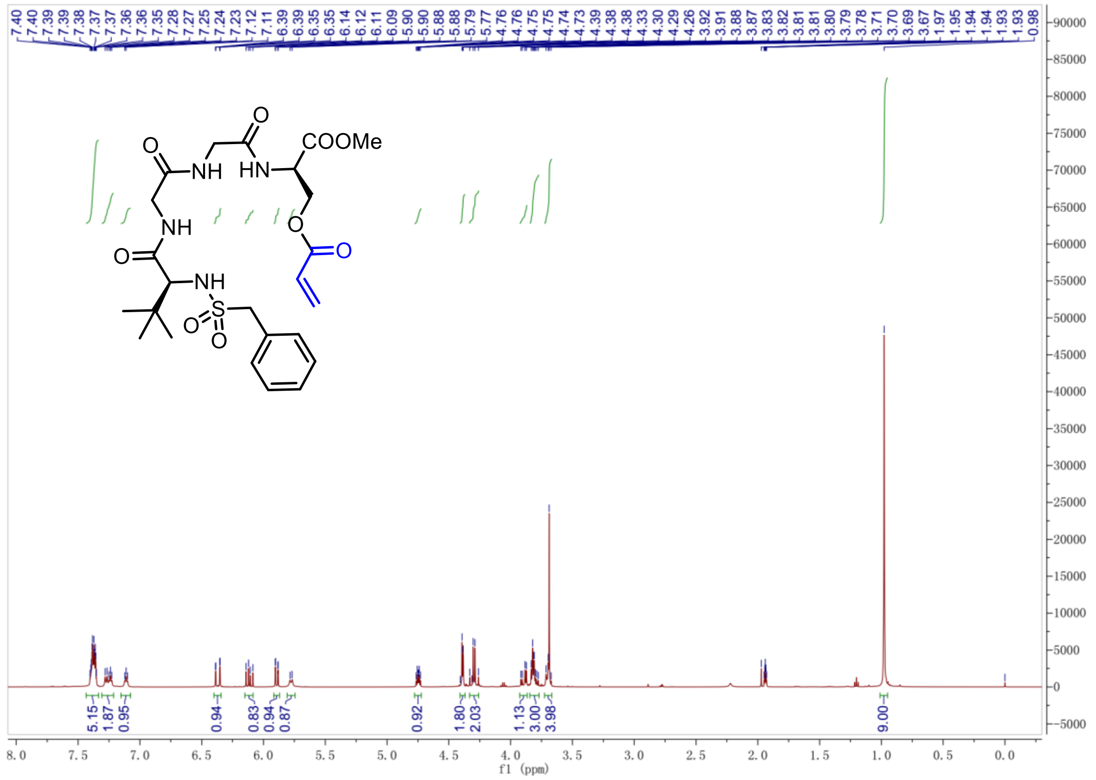

Supplementary Figure 251. ^{1}H NMR (500 MHz, CD_3CN) spectrum of compound **8c**

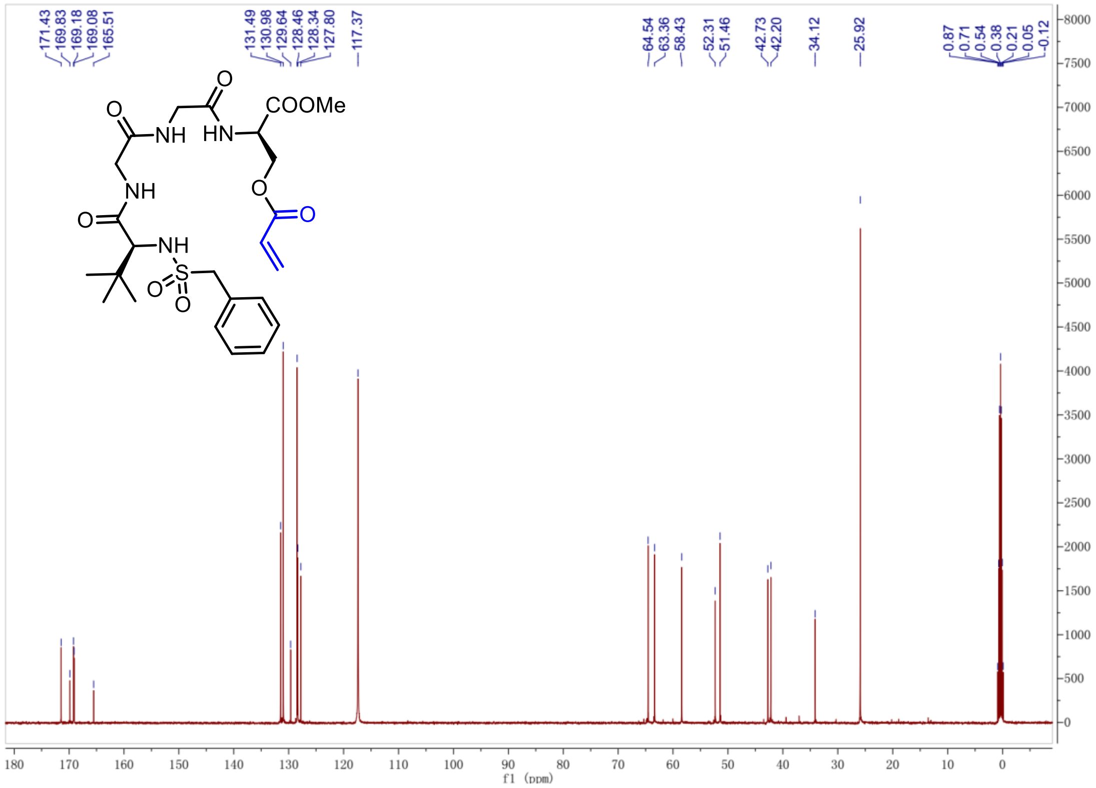

Supplementary Figure 252. ^{13}C NMR (125 MHz, CD_3CN) spectrum of compound **8c**

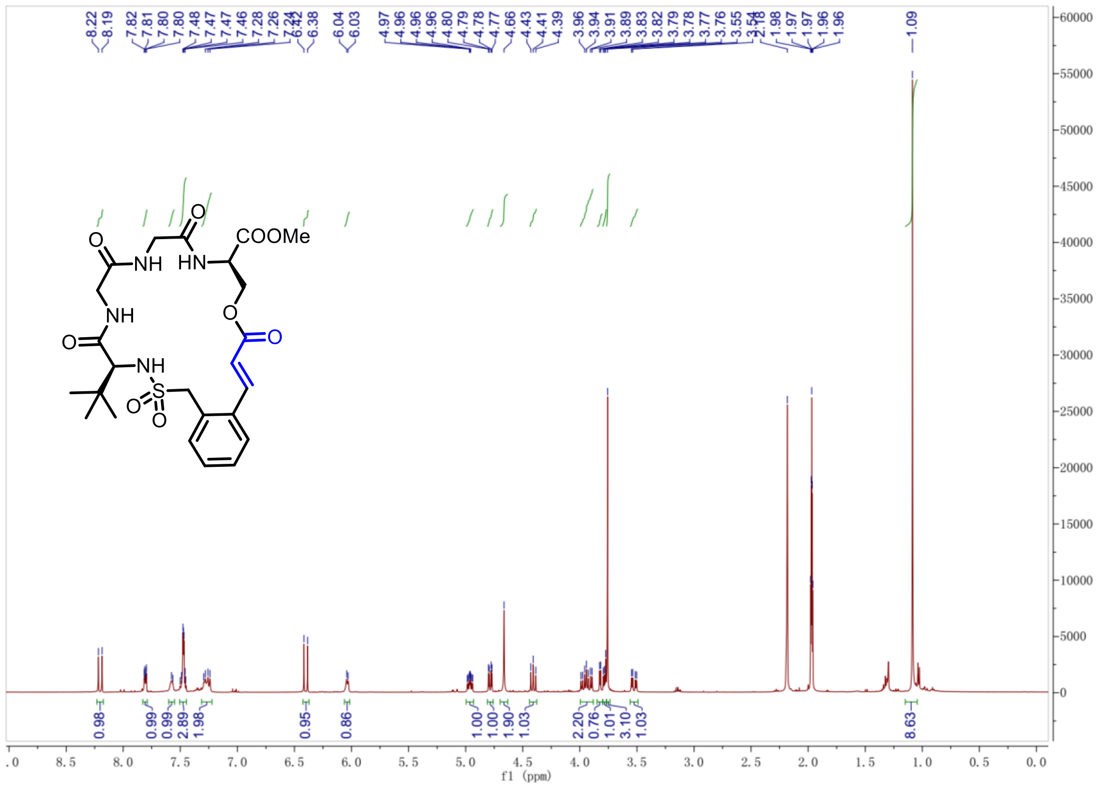

Supplementary Figure 253. ^{1}H NMR (500 MHz, CD_3CN) spectrum of compound **9c**

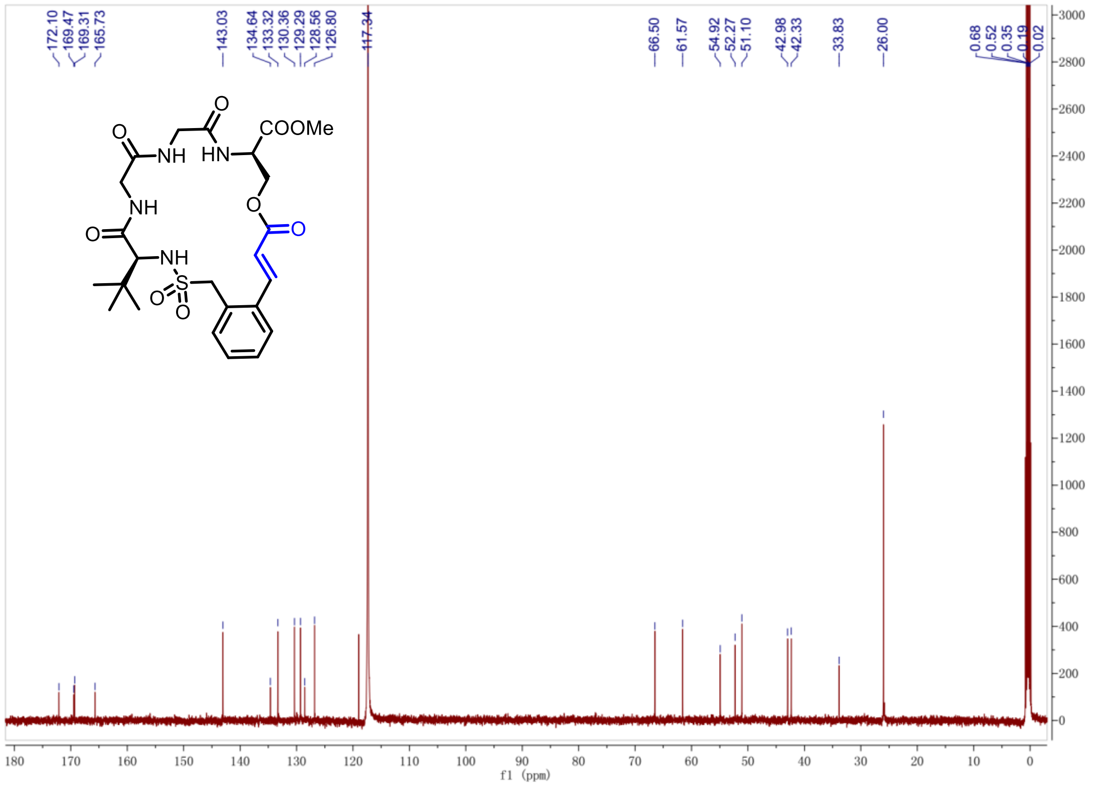

Supplementary Figure 254. ^{13}C NMR (125 MHz, CD_3CN)spectrum of compound **9c**

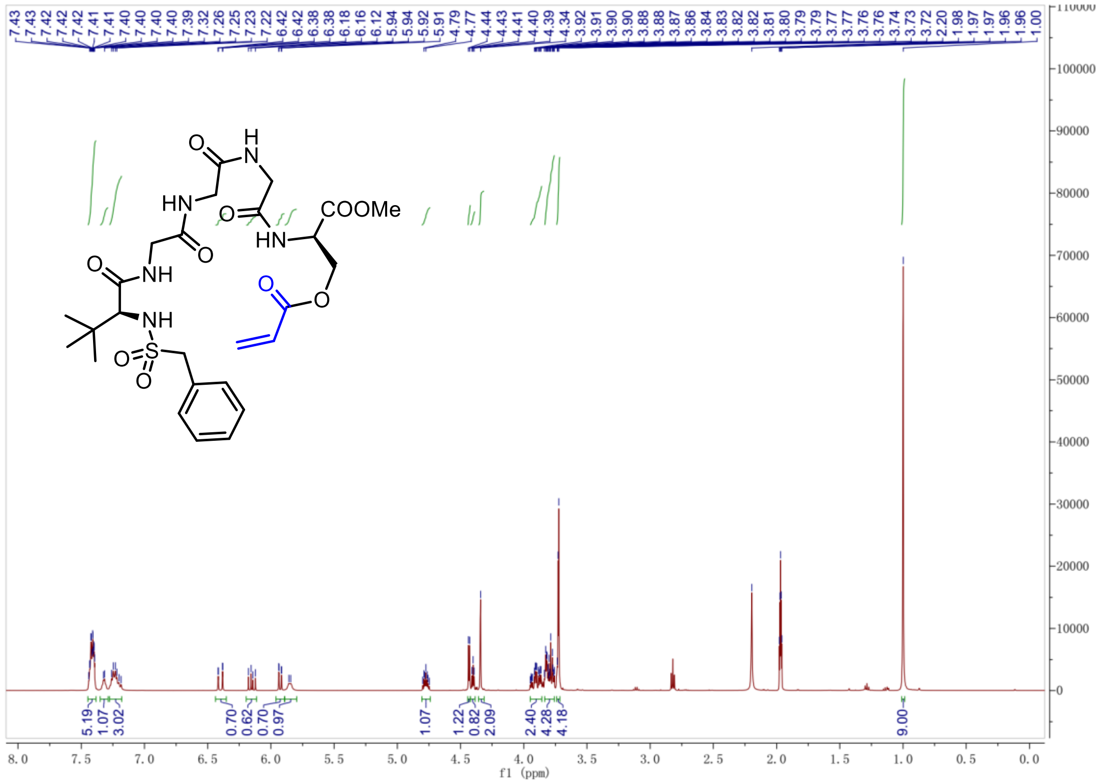

Supplementary Figure 255. ^{1}H NMR (500 MHz, CD_3CN) spectrum of compound **8d**

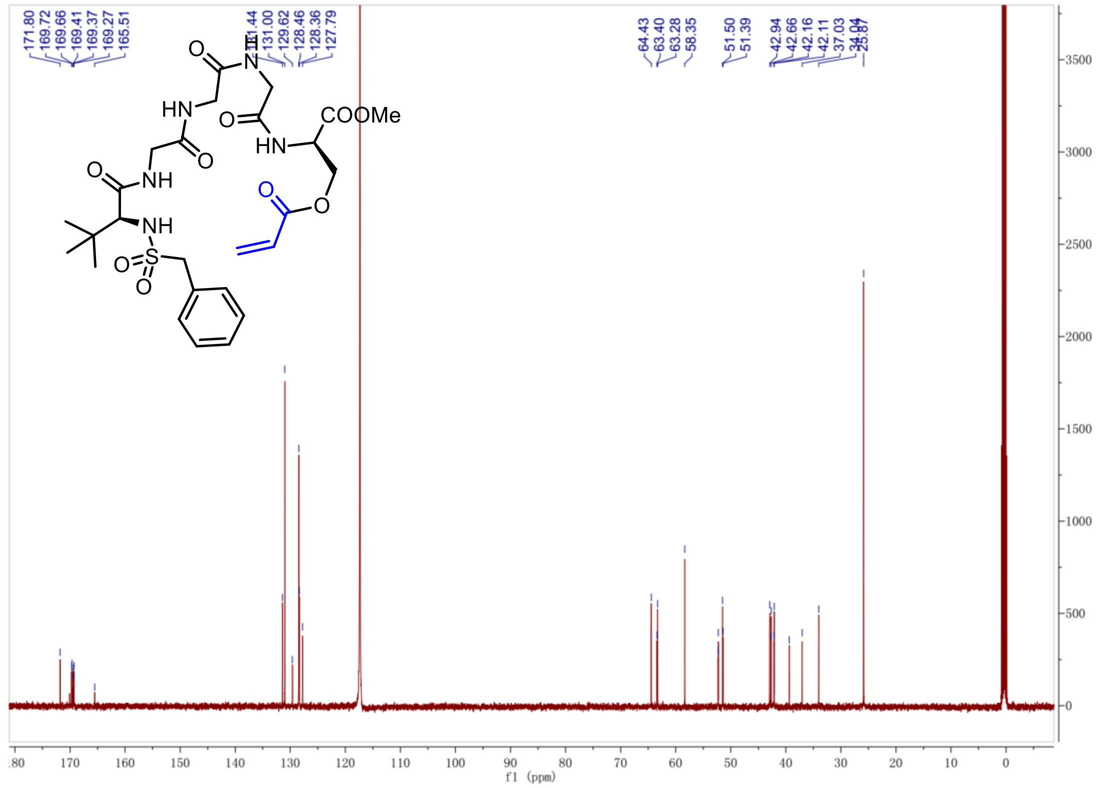

Supplementary Figure 256. ^{13}C NMR (125 MHz, CD_3CN) spectrum of compound **8d**

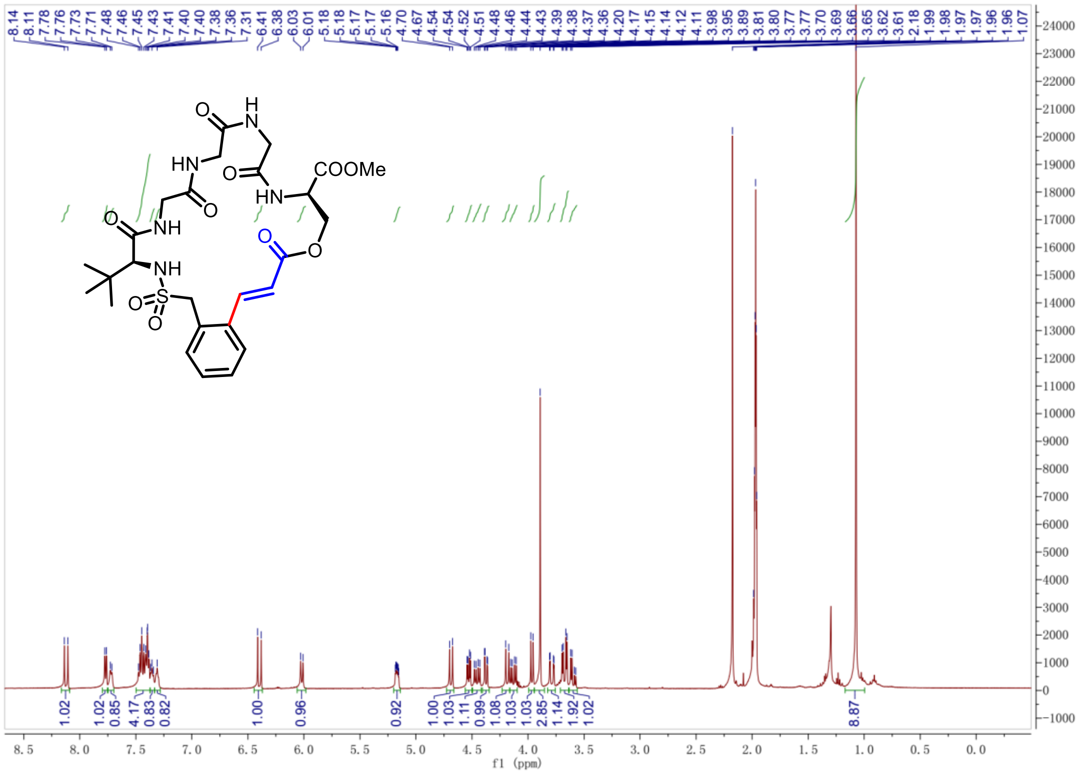

Supplementary Figure 257. ^{1}H NMR (500 MHz, CD_3CN) spectrum of compound **9d**

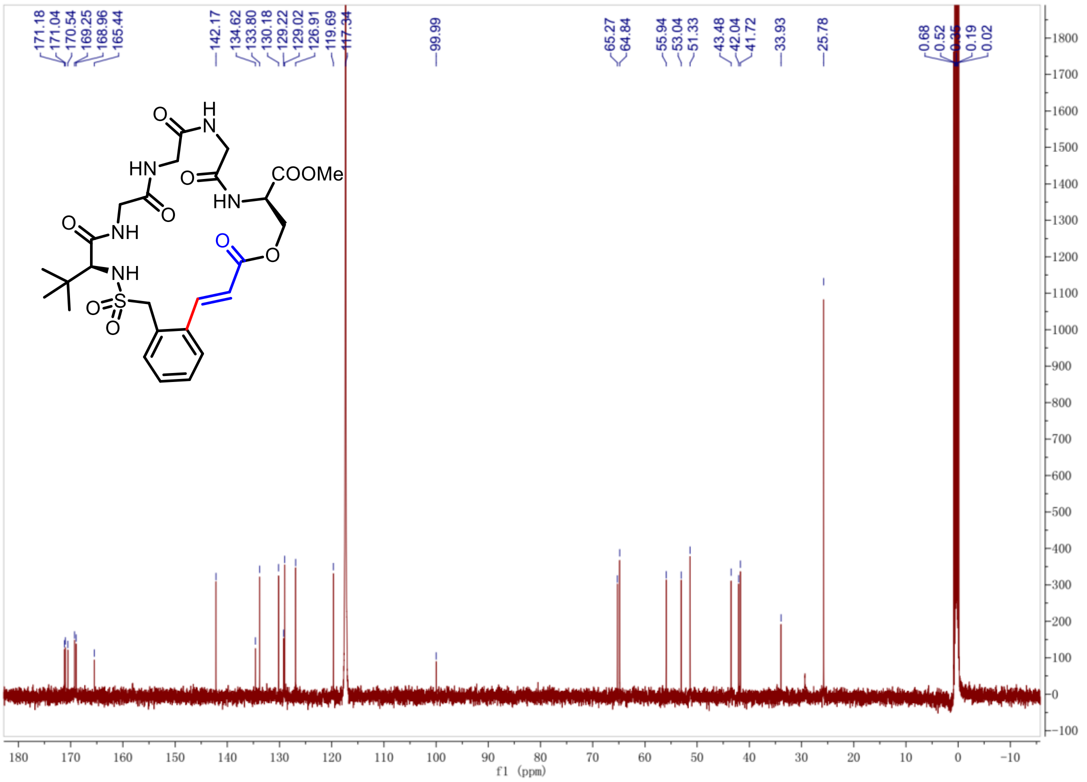

Supplementary Figure 258. ^{13}C NMR (125 MHz, CD_3CN) spectrum of compound **9d**

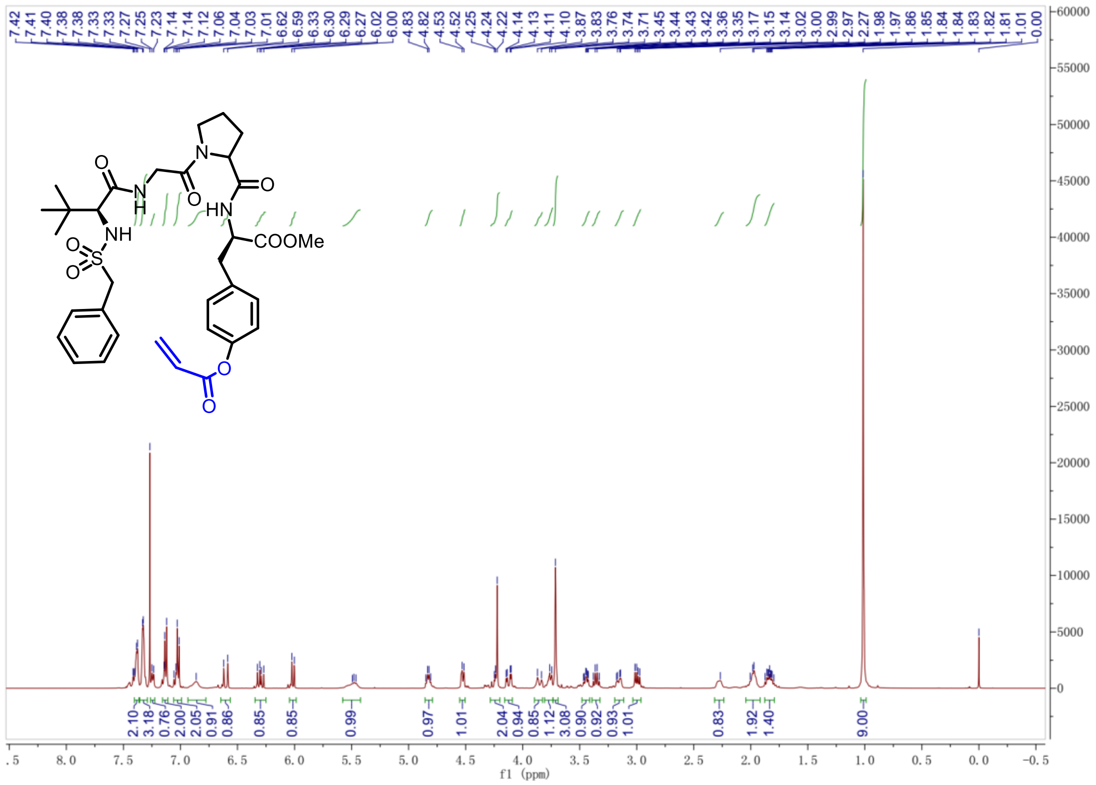

Supplementary Figure 259. ^{1}H NMR (500 MHz, $CDCl_3$) spectrum of compound **8e**

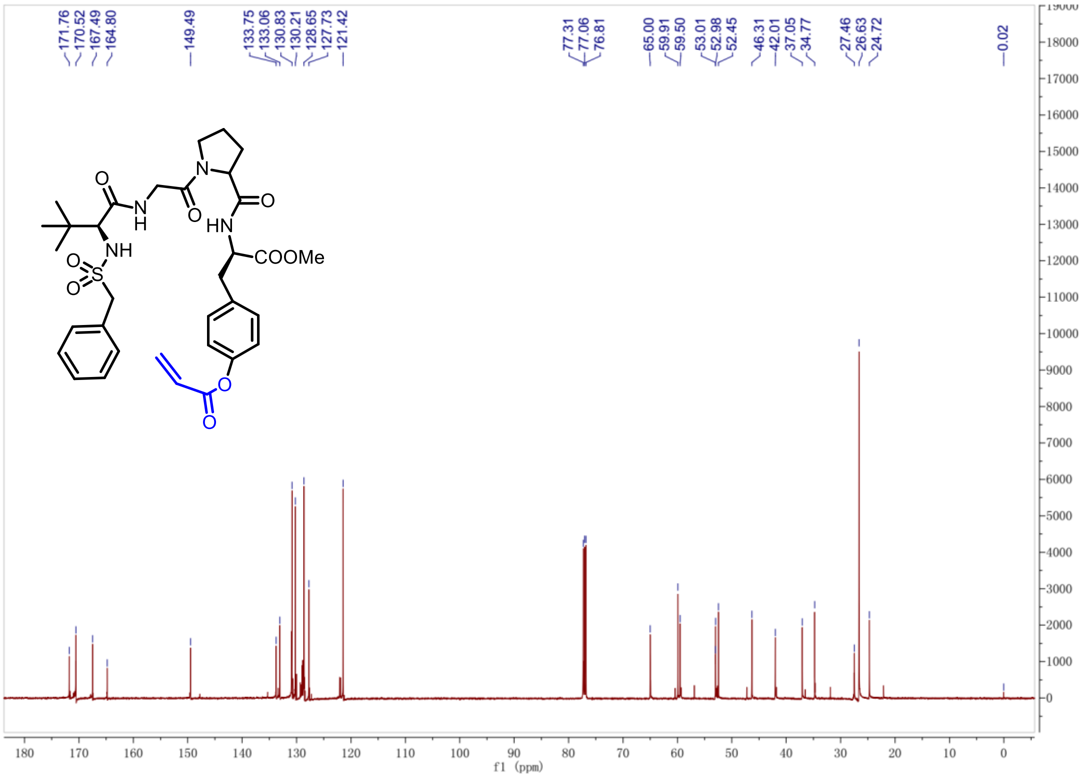

Supplementary Figure 260. ^{13}C NMR (125MHz, $CDCl_3$) spectrum of compound **8e**

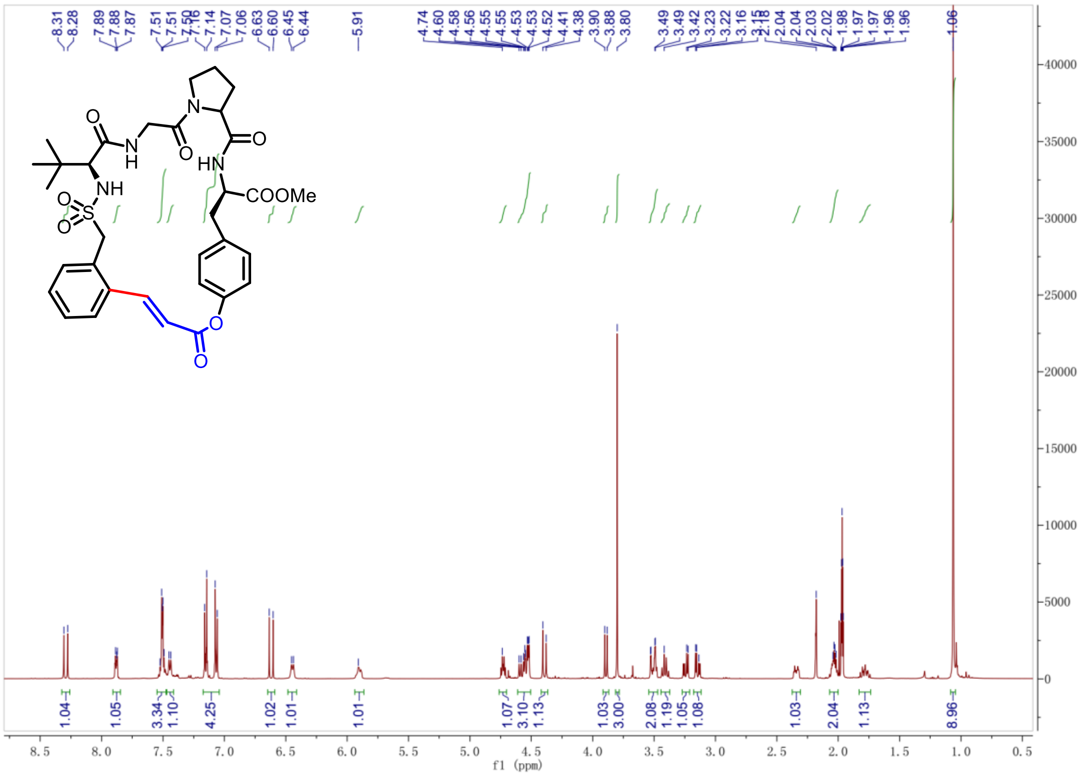

Supplementary Figure 261. ^{1}H NMR (500 MHz, CD_3CN) spectrum of compound **9e**

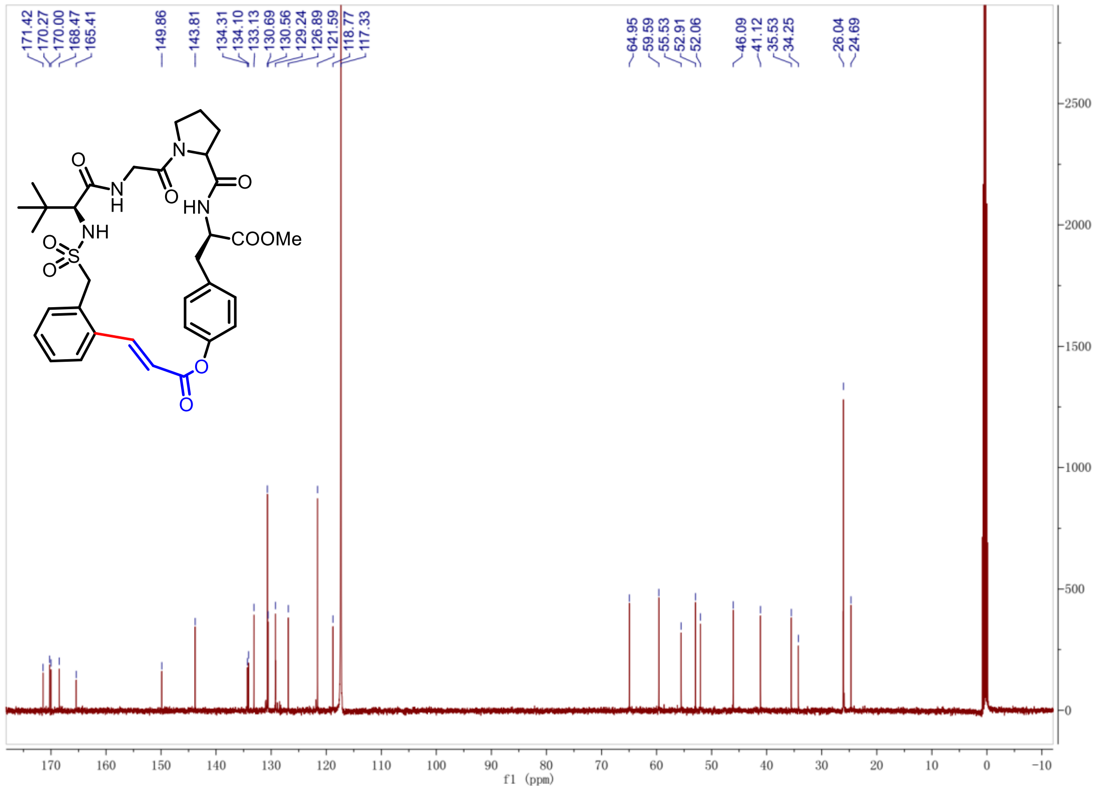

Supplementary Figure 262. ^{13}C NMR (125 MHz, CD_3CN) spectrum of compound **9e**

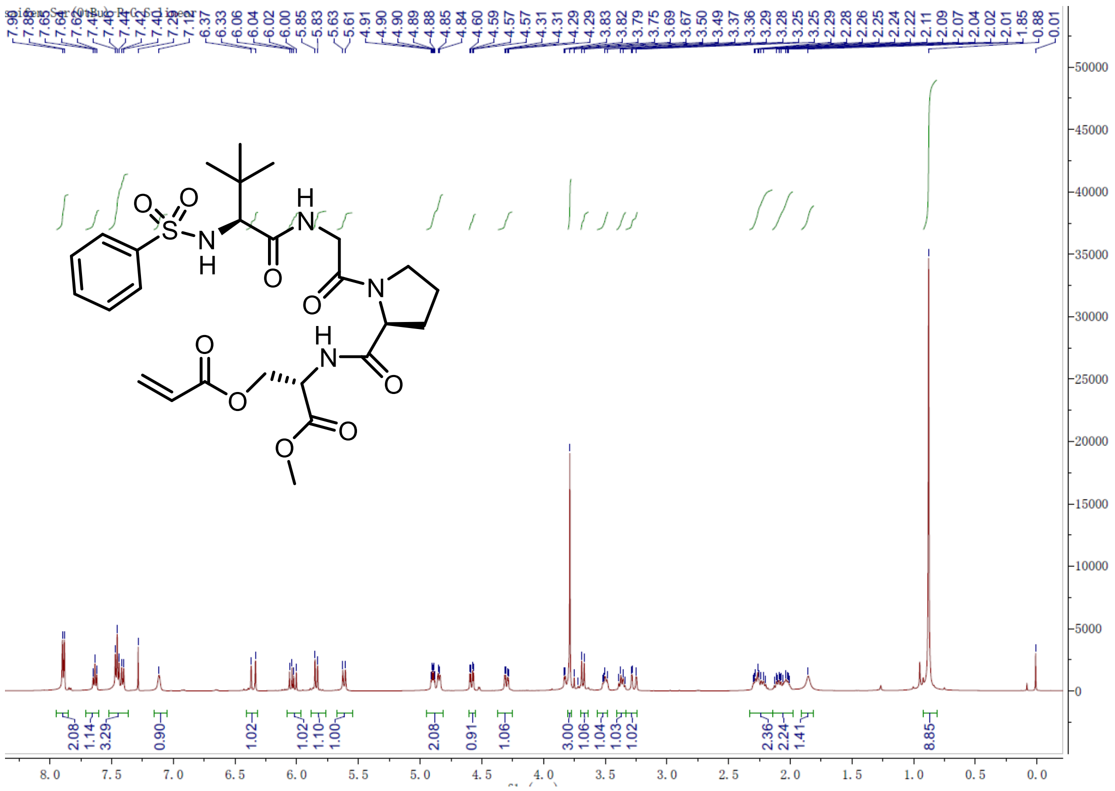

Supplementary Figure 263. ^{1}H NMR (400 MHz, $CDCl_3$) spectrum of compound **8f**

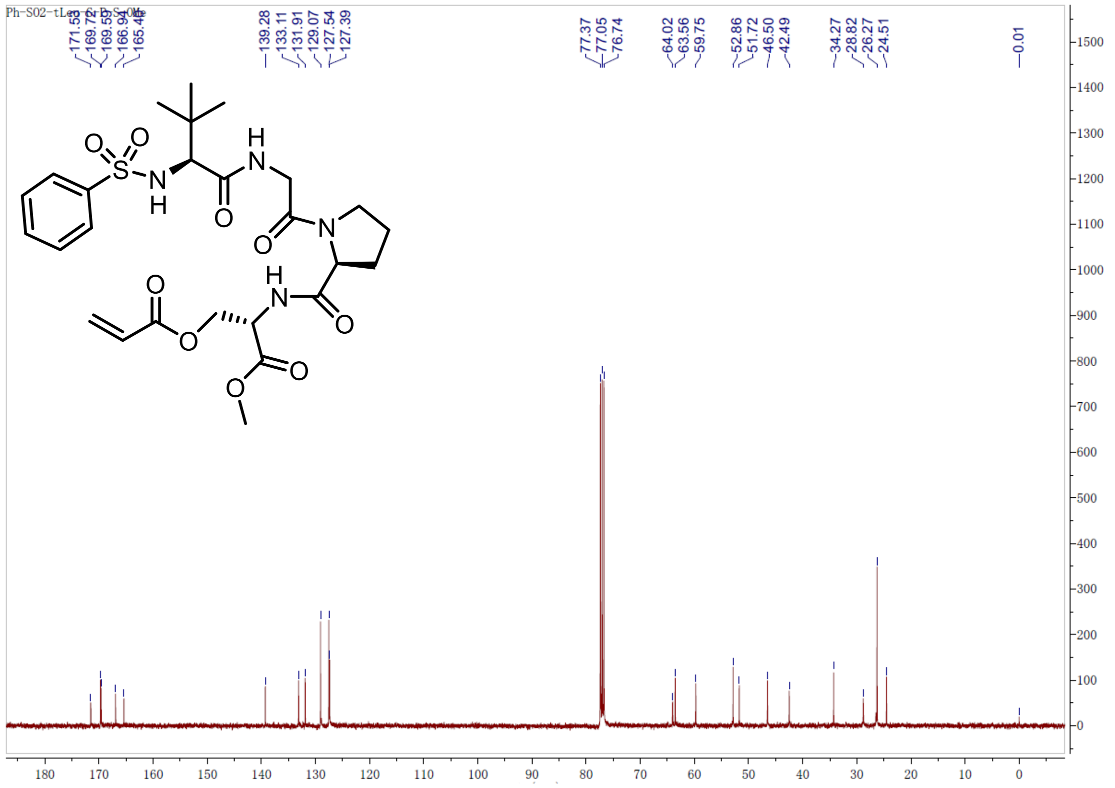

Supplementary Figure 264. ^{13}C NMR (100 MHz, $CDCl_3$) spectrum of compound **8f**

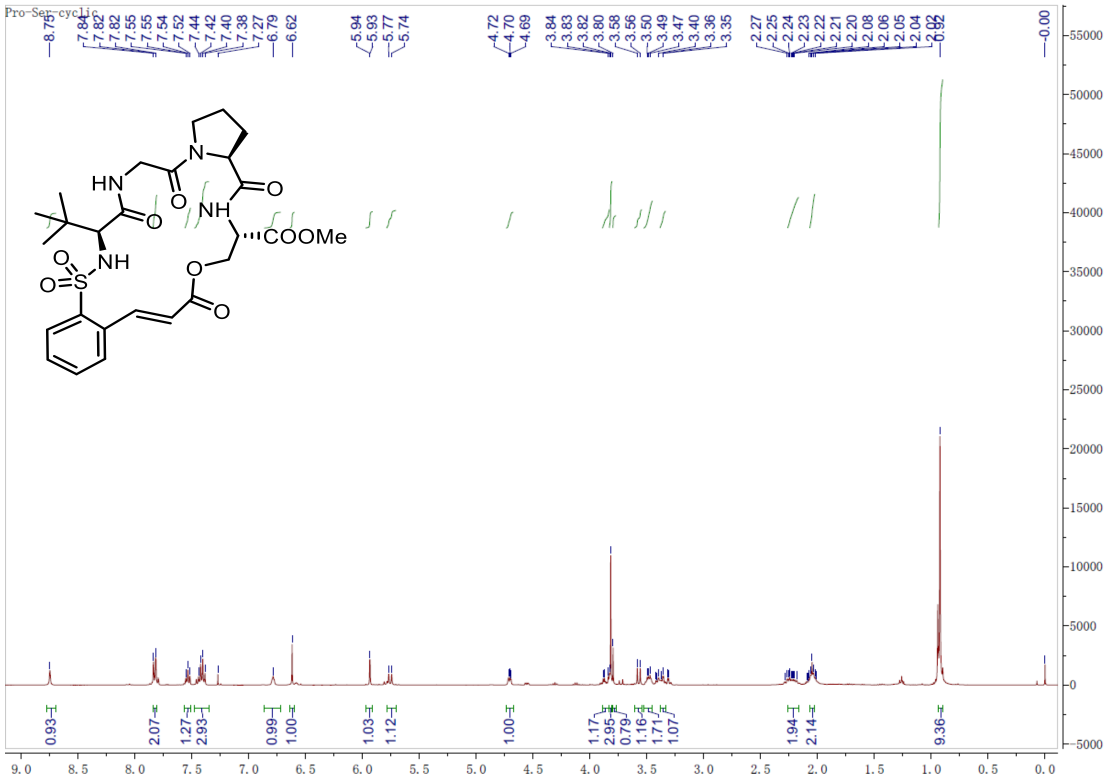

Supplementary Figure 265. ^{1}H NMR (400 MHz, $CDCl_3$) spectrum of compound **9f**

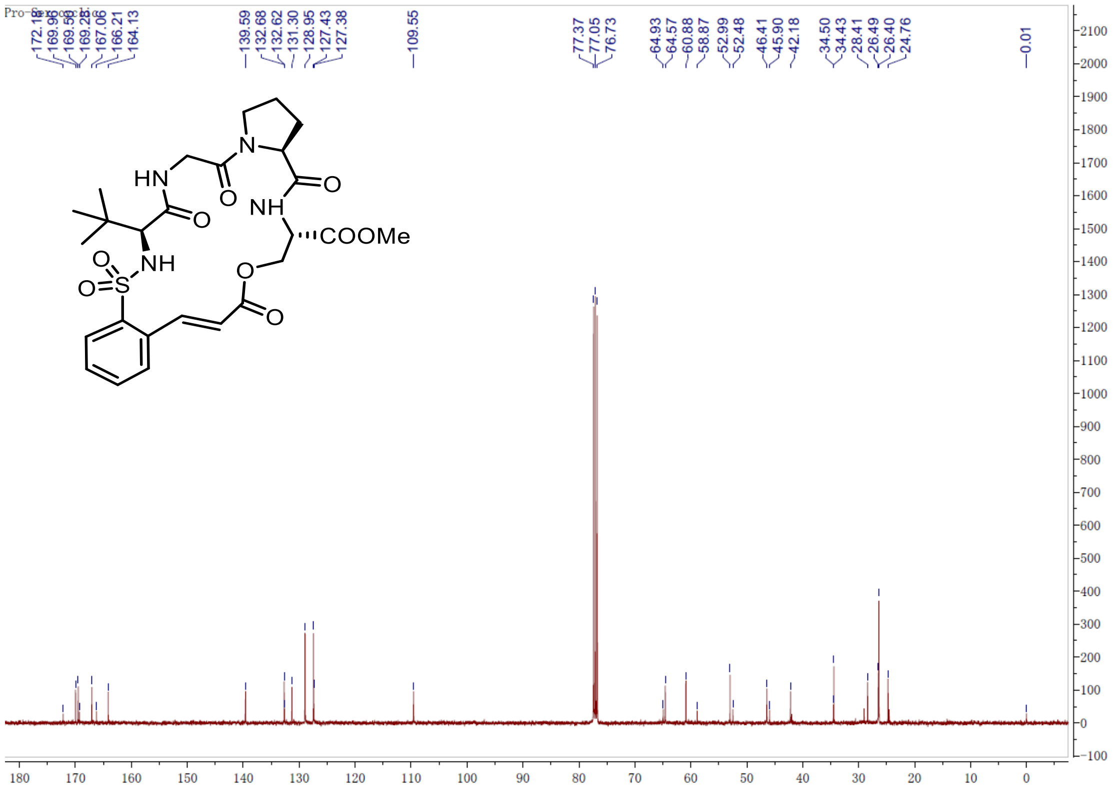

Supplementary Figure 266. ^{13}C NMR (100 MHz, $CDCl_3$) spectrum of compound **9f**

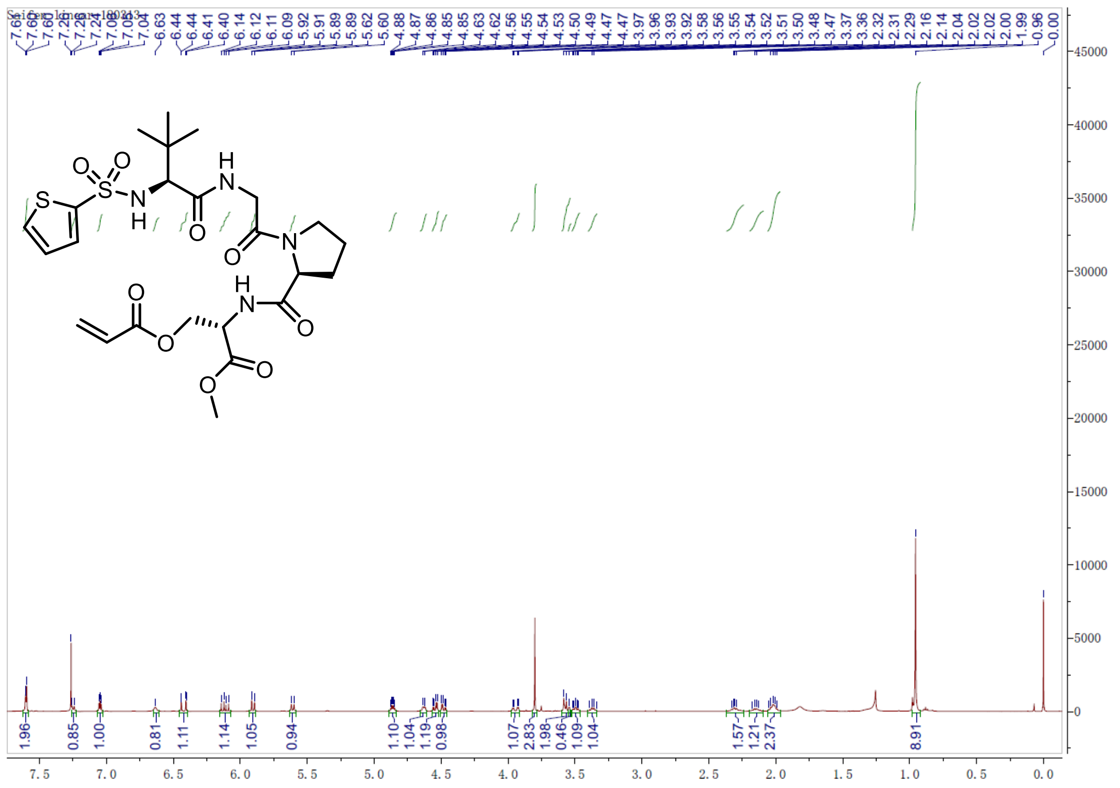

Supplementary Figure 267. ^{1}H NMR (400 MHz, $CDCl_3$) spectrum of compound **8g**

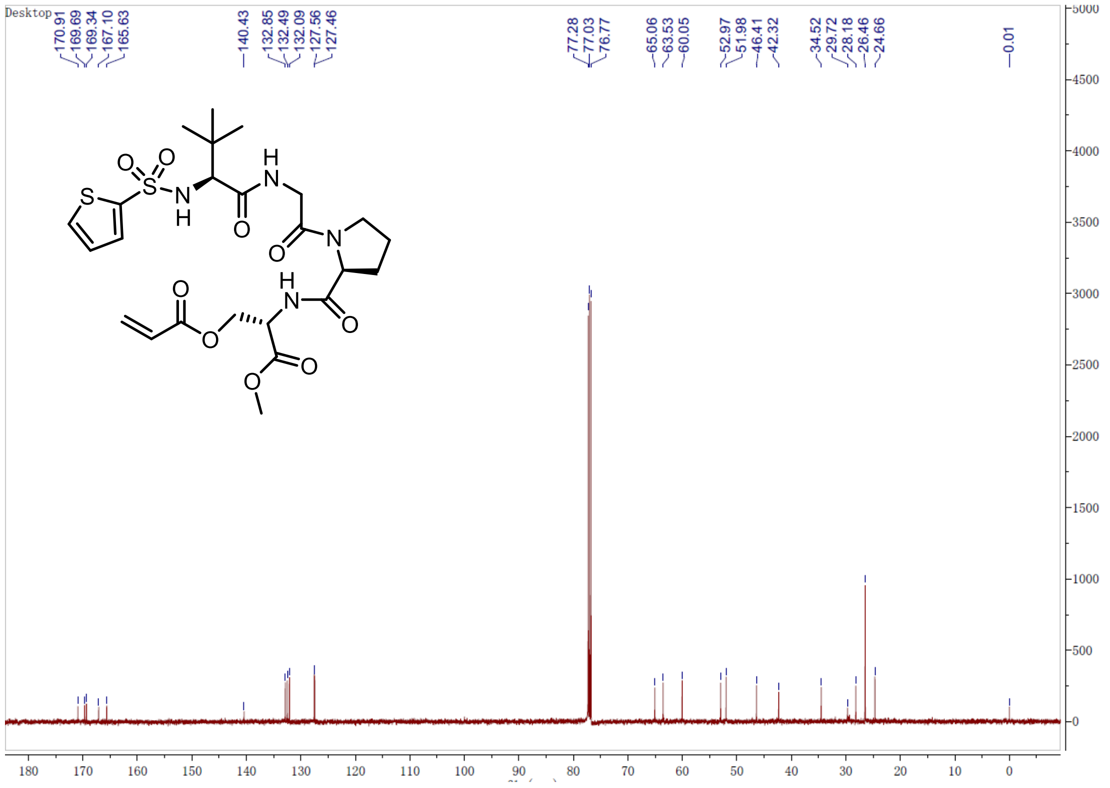

Supplementary Figure 268. ^{13}C NMR (100 MHz, $CDCl_3$) spectrum of compound **8g**

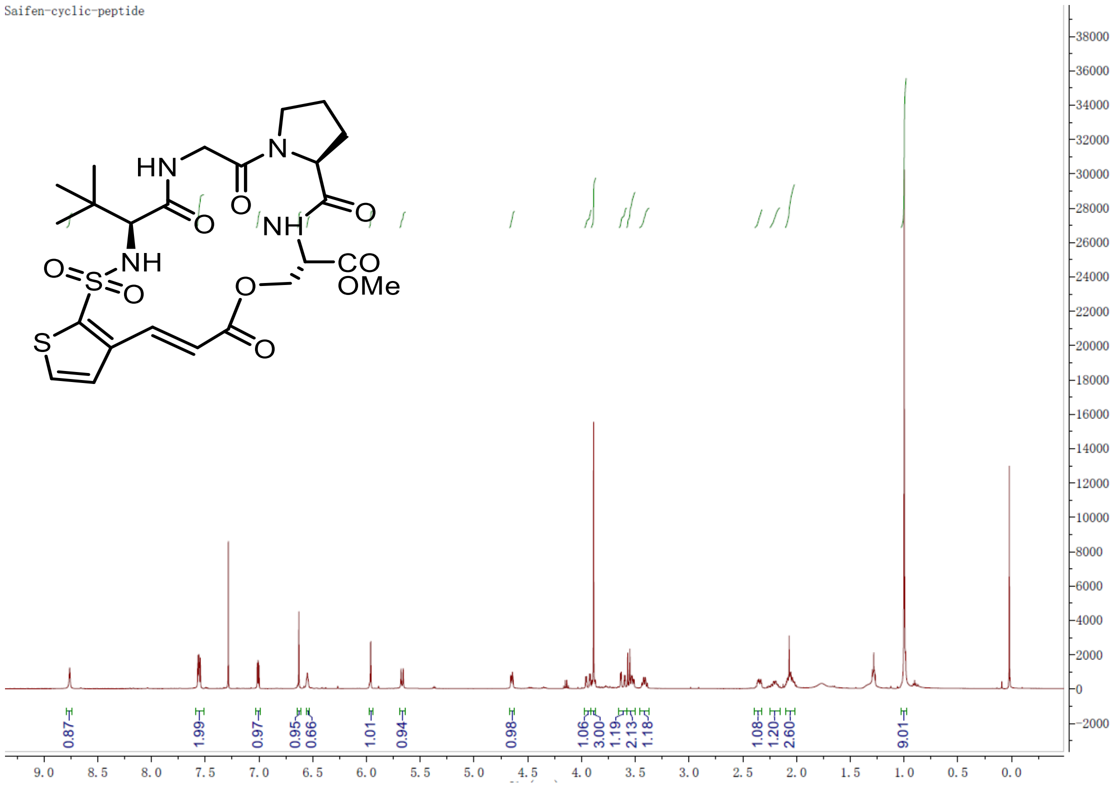

Supplementary Figure 269. ^{1}H NMR (400 MHz, $CDCl_3$) spectrum of compound **9g**

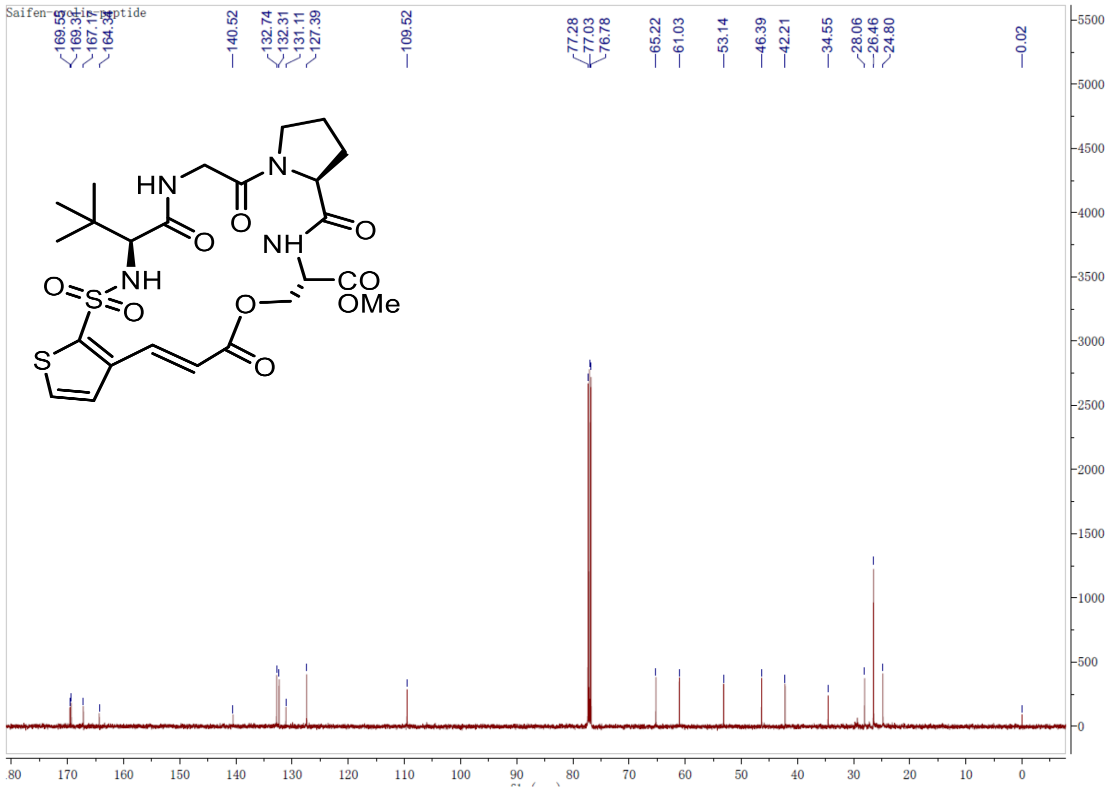

Supplementary Figure 270. ^{13}C NMR (100 MHz, $CDCl_3$) spectrum of compound **9g**

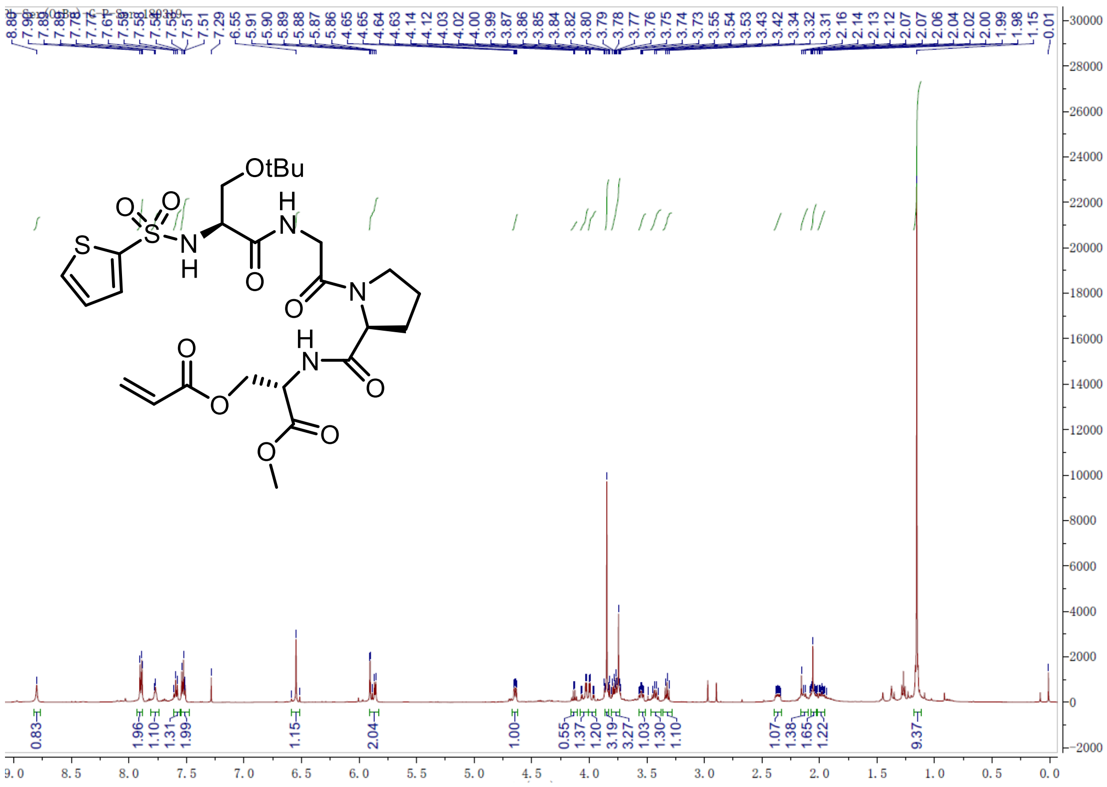

Supplementary Figure 271. ^{1}H NMR (400 MHz, $CDCl_3$) spectrum of compound **8h**

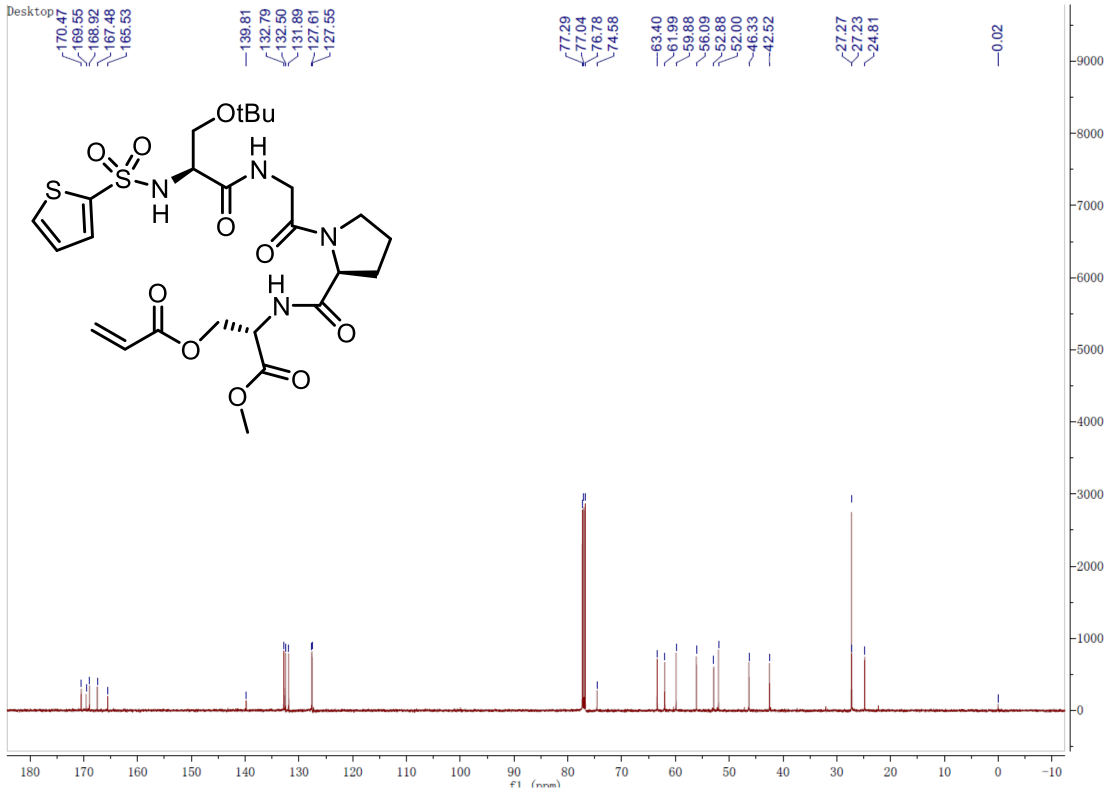

Supplementary Figure 272. ^{13}C NMR (100 MHz, $CDCl_3$) spectrum of compound **8h**

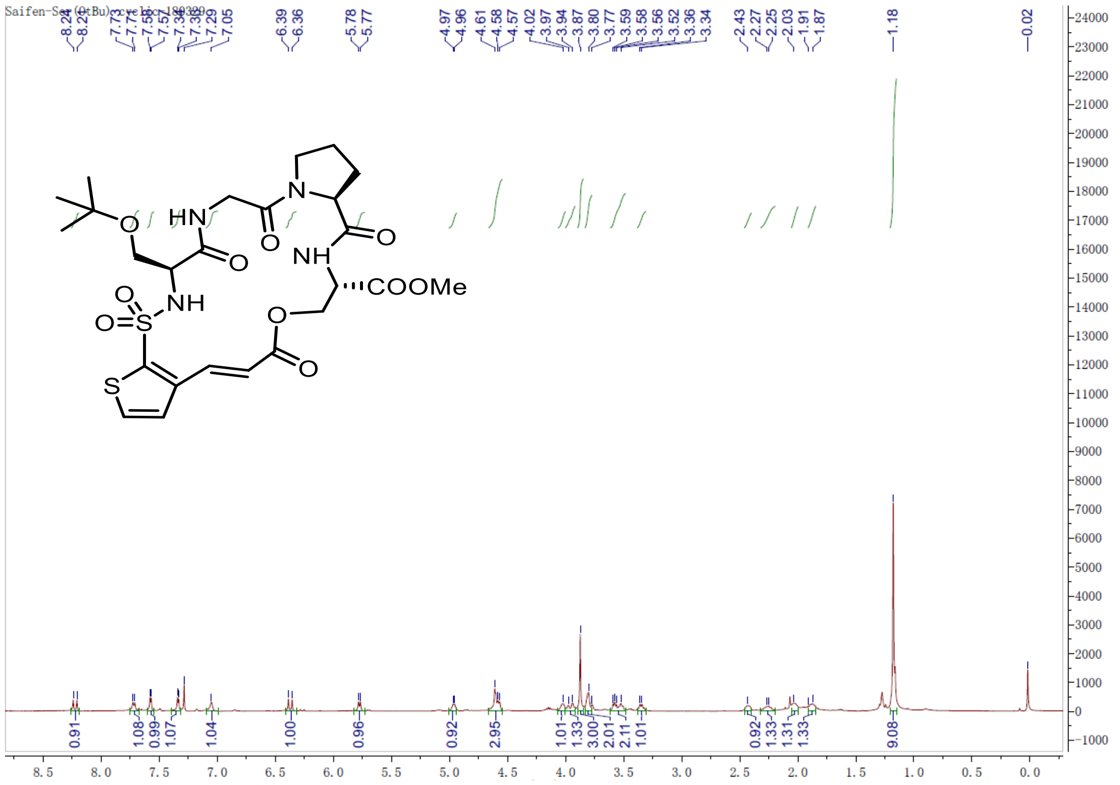

Supplementary Figure 273. ^{1}H NMR (400 MHz, $CDCl_3$) spectrum of compound **9h**

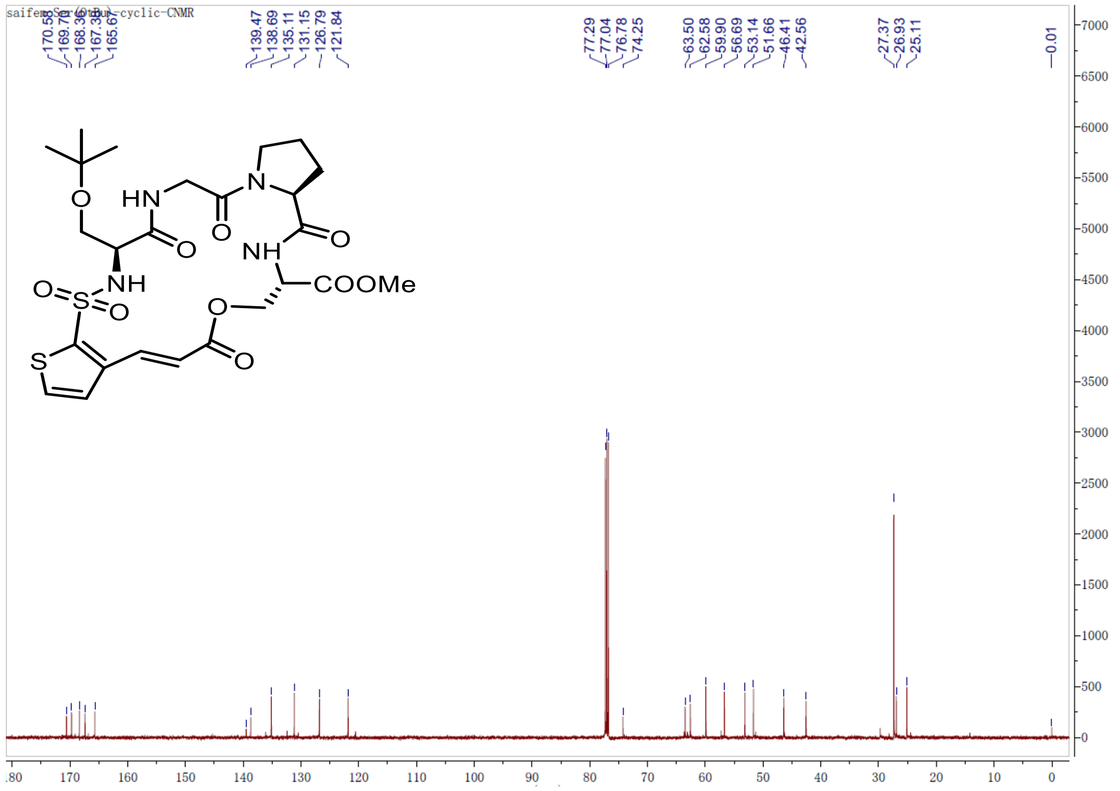

Supplementary Figure 274. ^{13}C NMR (100 MHz, $CDCl_3$) spectrum of compound **9h**

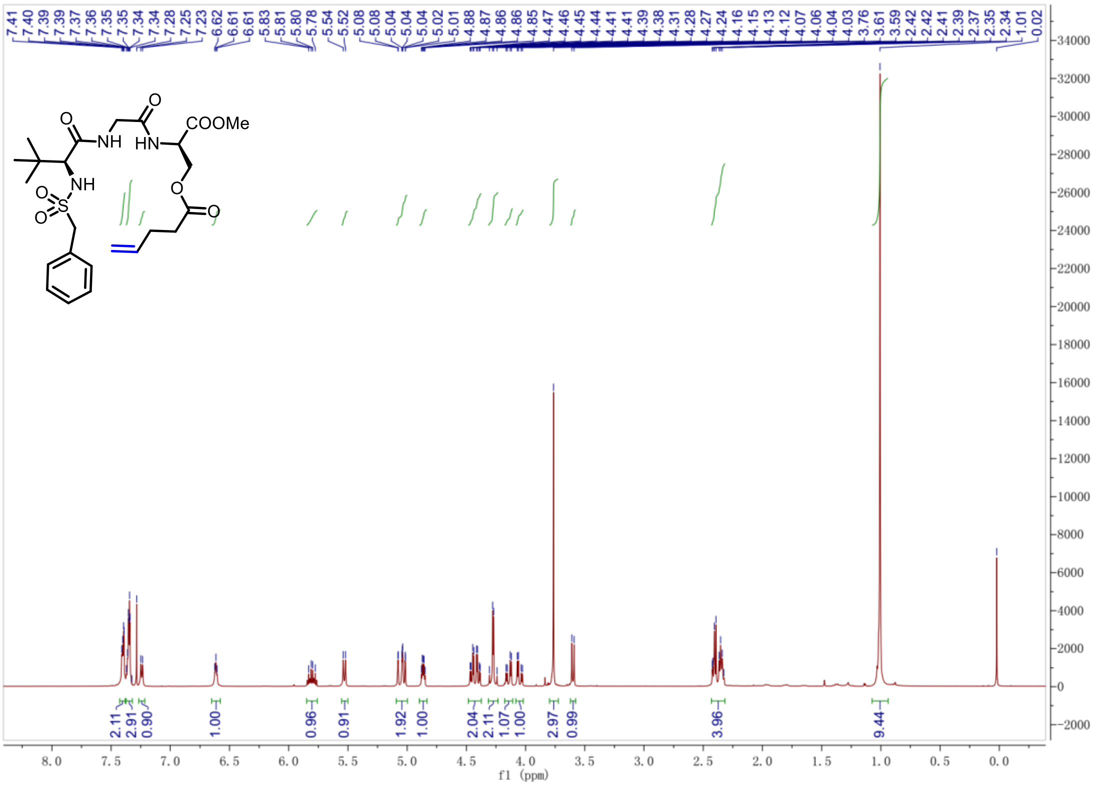

Supplementary Figure 275. ^{1}H NMR (500 MHz, $CDCl_3$) spectrum of compound **8i**

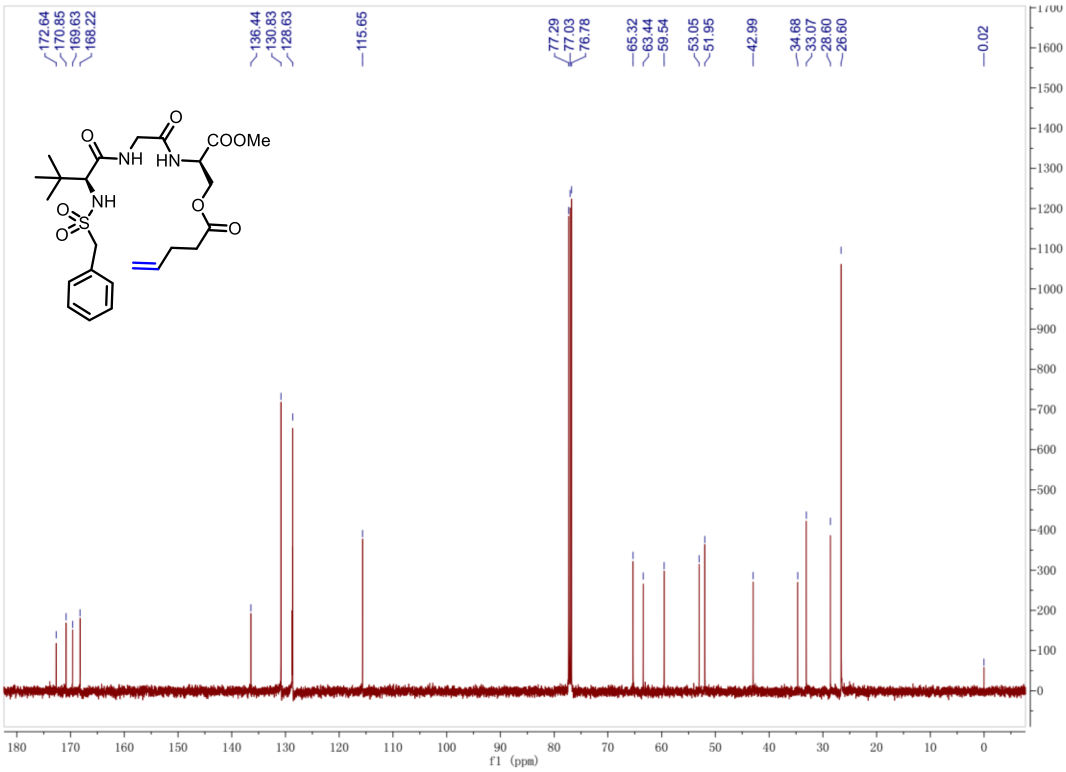

Supplementary Figure 276. ^{13}C NMR (125 MHz, $CDCl_3$) spectrum of compound **8i**

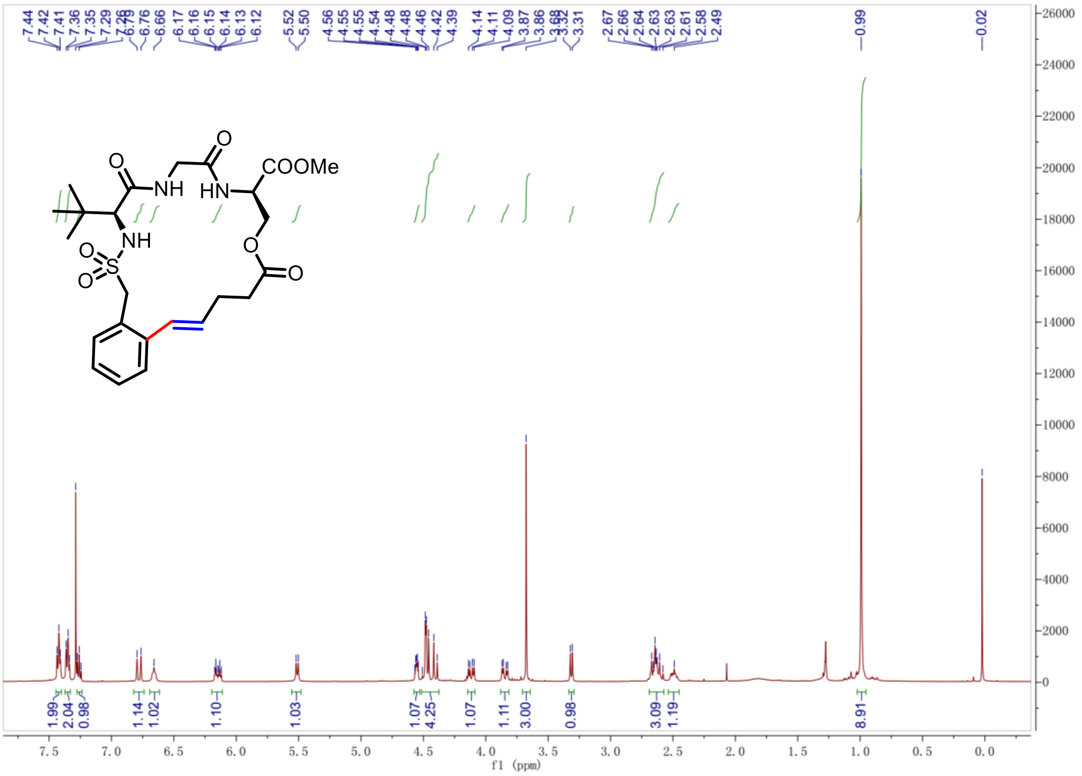

Supplementary Figure 277. ^{1}H NMR (500 MHz, $CDCl_3$) spectrum of compound **9i**

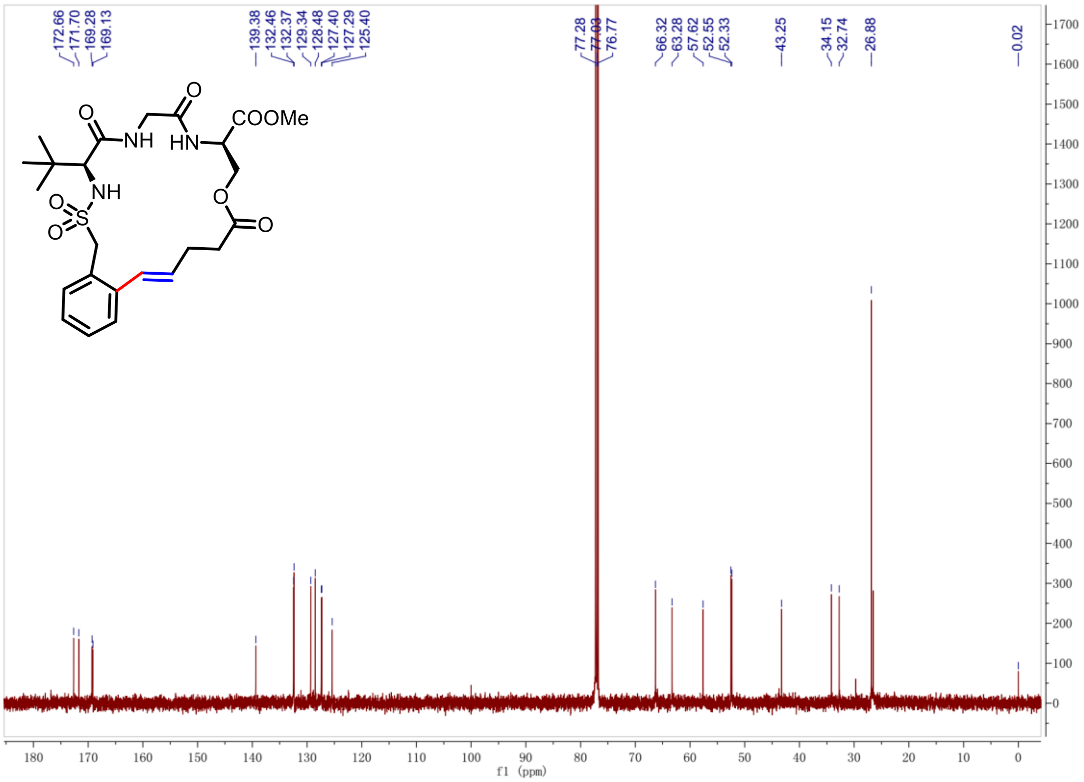

Supplementary Figure 278. ^{13}C NMR (125MHz, $CDCl_3$) spectrum of compound **9i**

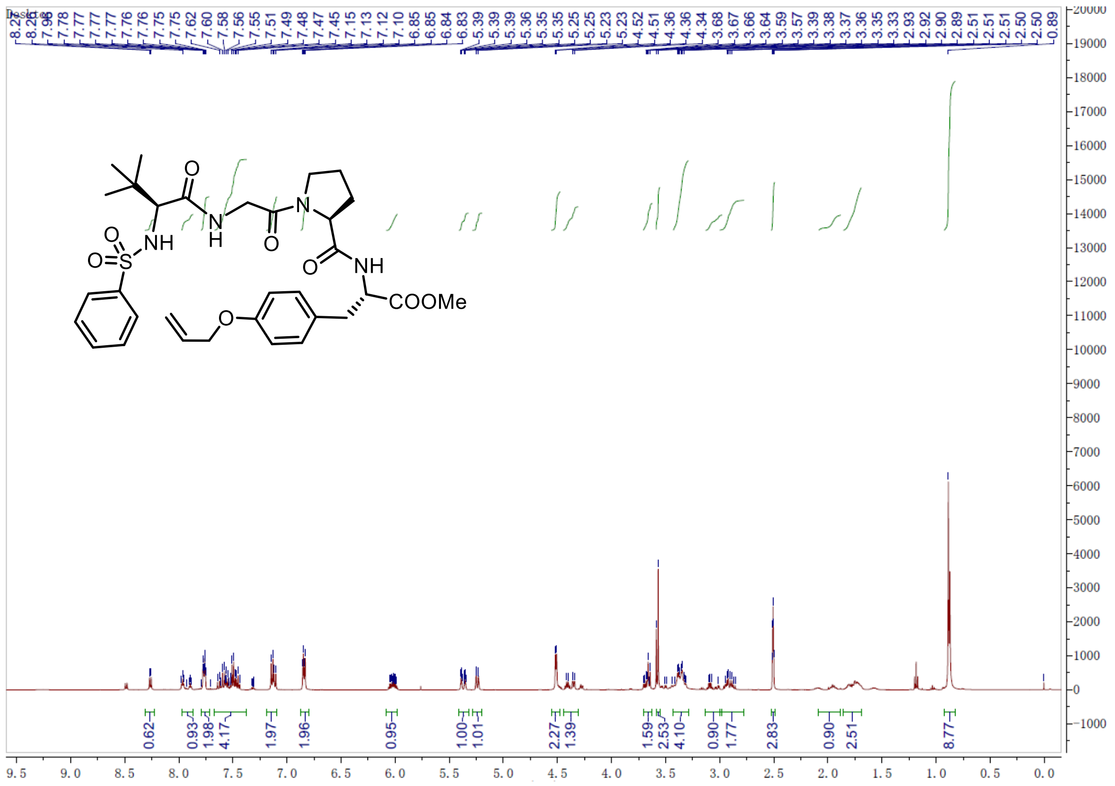

Supplementary Figure 279. ^{1}H NMR (400 MHz, $CDCl_3$) spectrum of compound **8j**

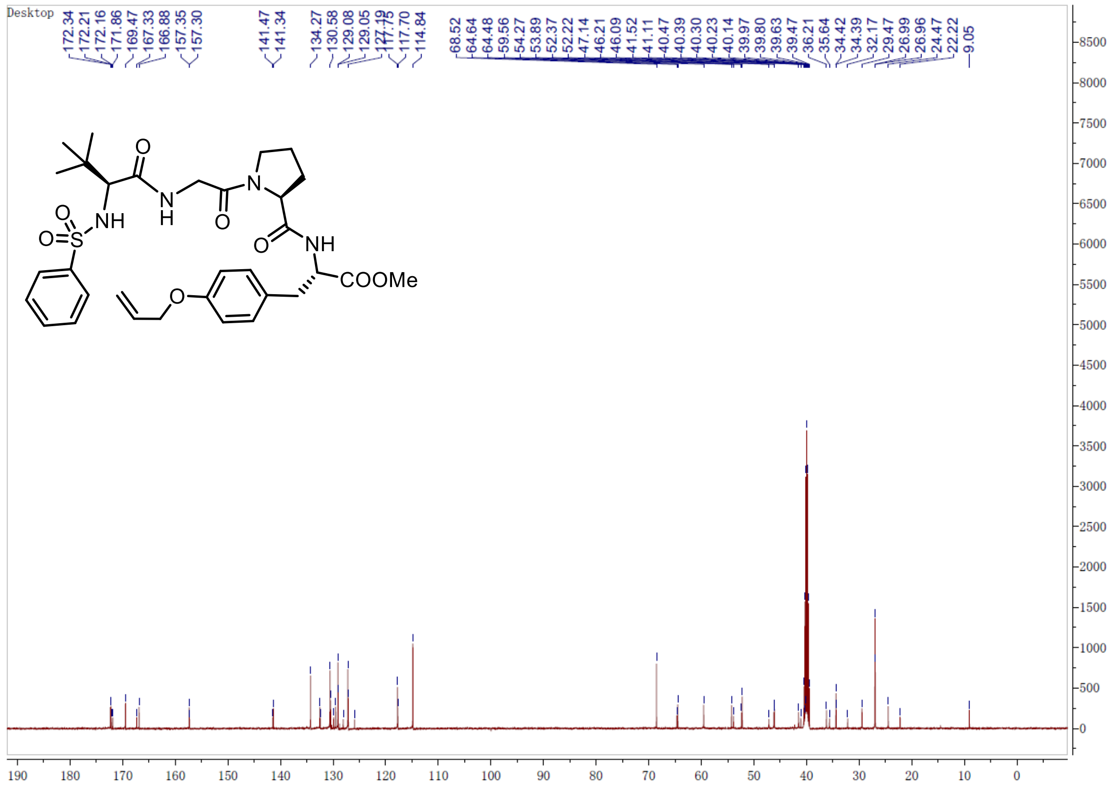

Supplementary Figure 280. ^{13}C NMR (100 MHz, $CDCl_3$) spectrum of compound **8j**

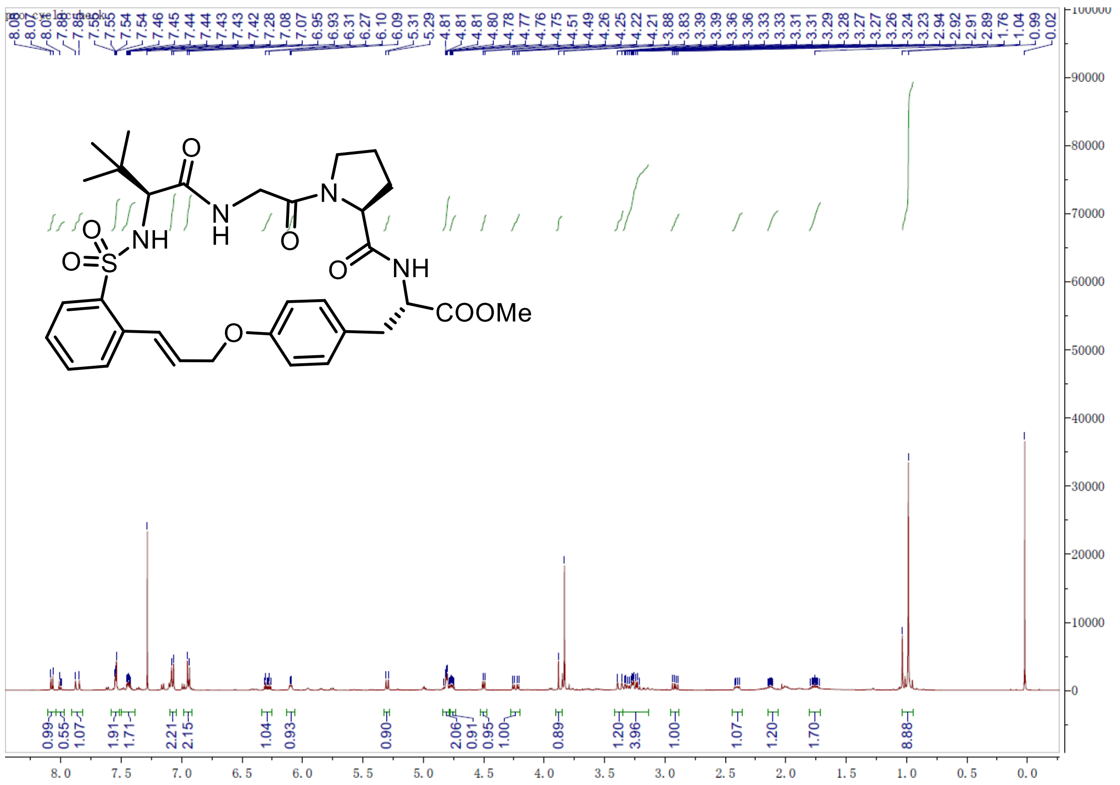

Supplementary Figure 281. ^{1}H NMR (400 MHz, $CDCl_3$) spectrum of compound **9j**

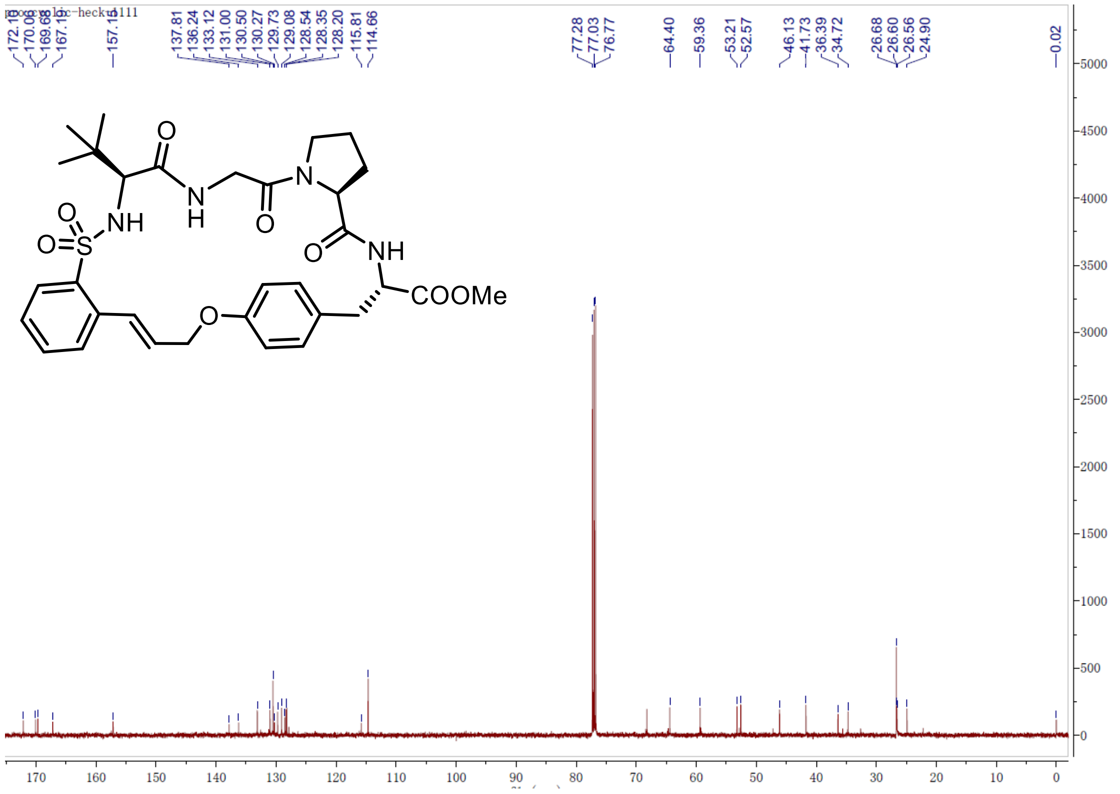

Supplementary Figure 282. ^{13}C NMR (100 MHz, $CDCl_3$) spectrum of compound **9j**

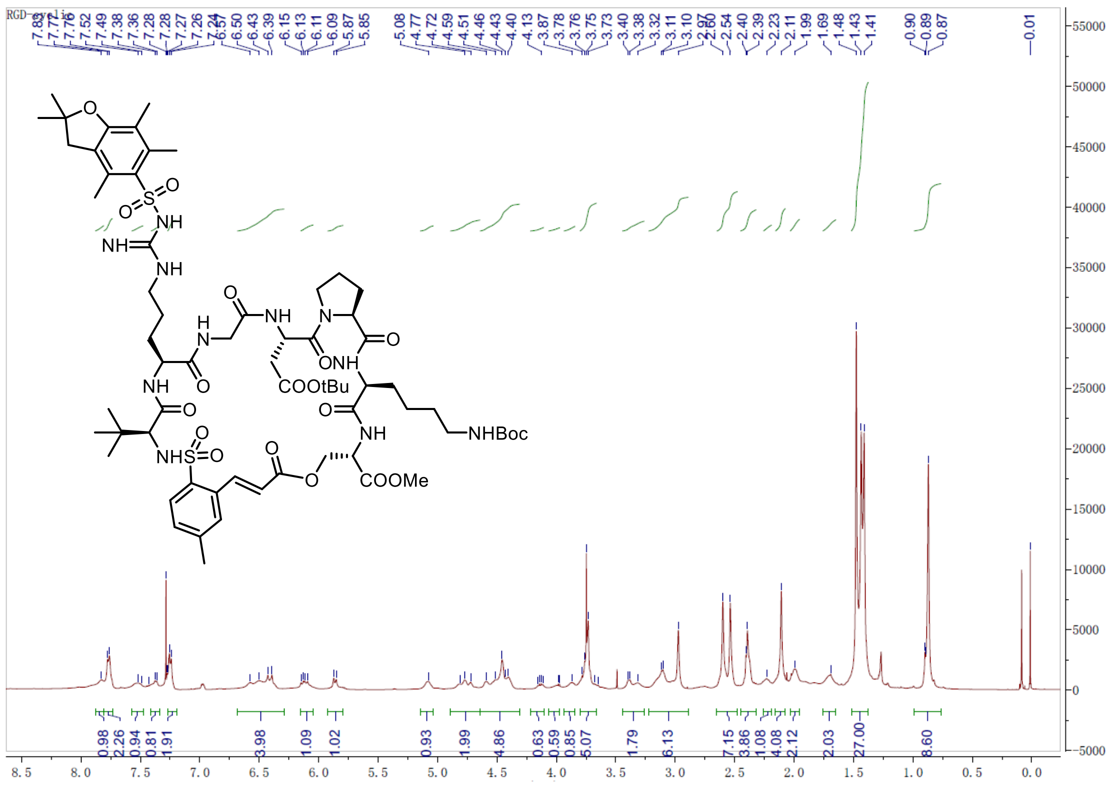

Supplementary Figure 283. ^{1}H NMR (400 MHz, $CDCl_3$) spectrum of compound **9k'**

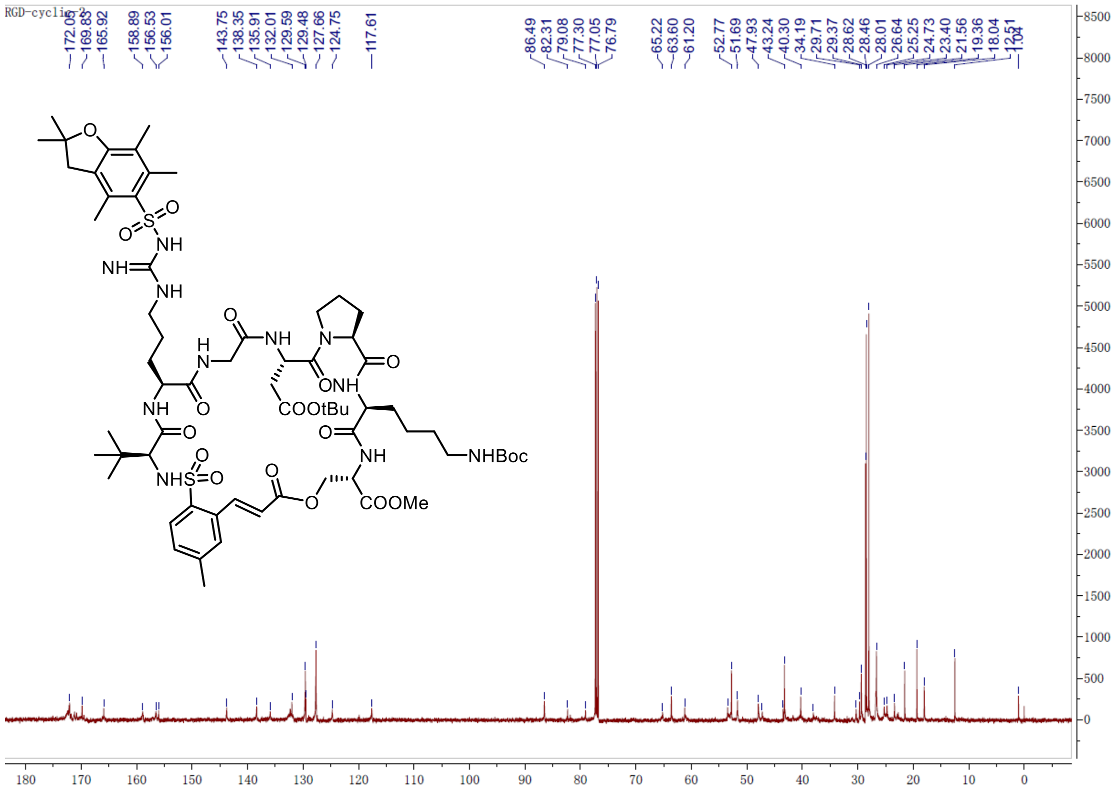

Supplementary Figure 284. ^{13}C NMR (100 MHz, $CDCl_3$) spectrum of compound **9k'**

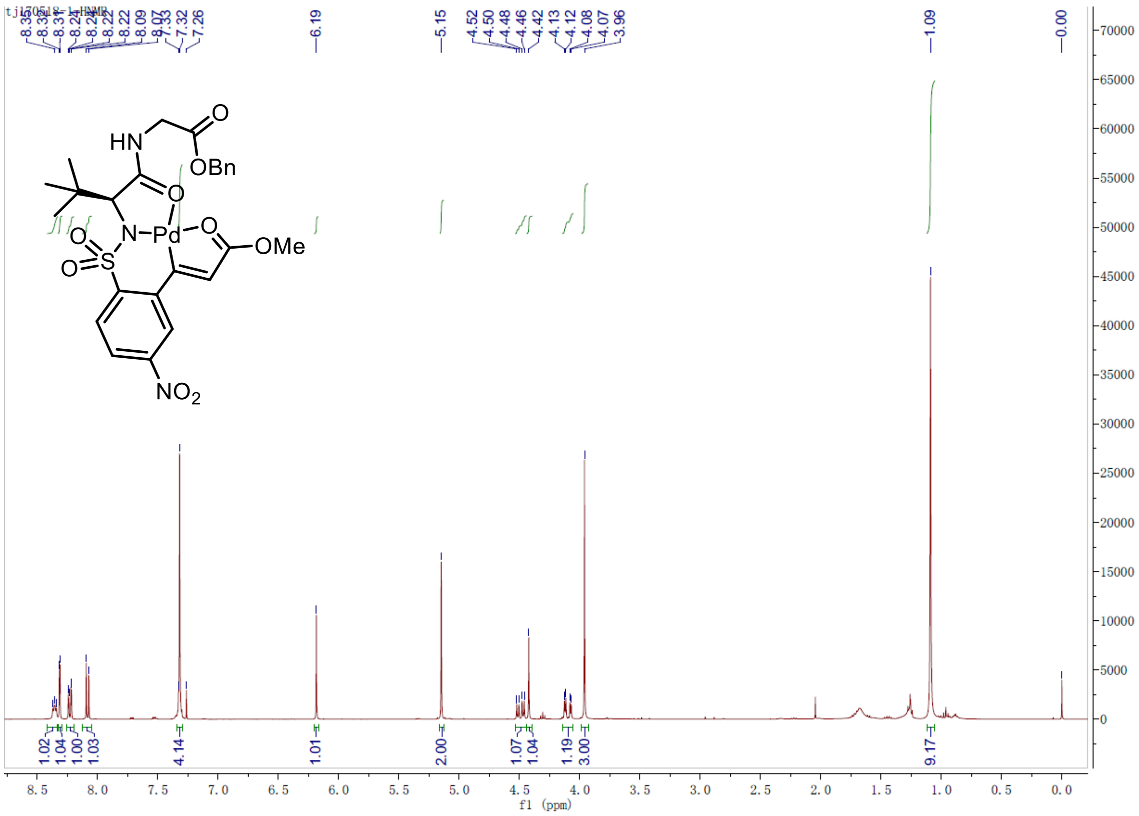

Supplementary Figure 285. ^{1}H NMR (400 MHz, $CDCl_3$) spectrum of compound **7aa**

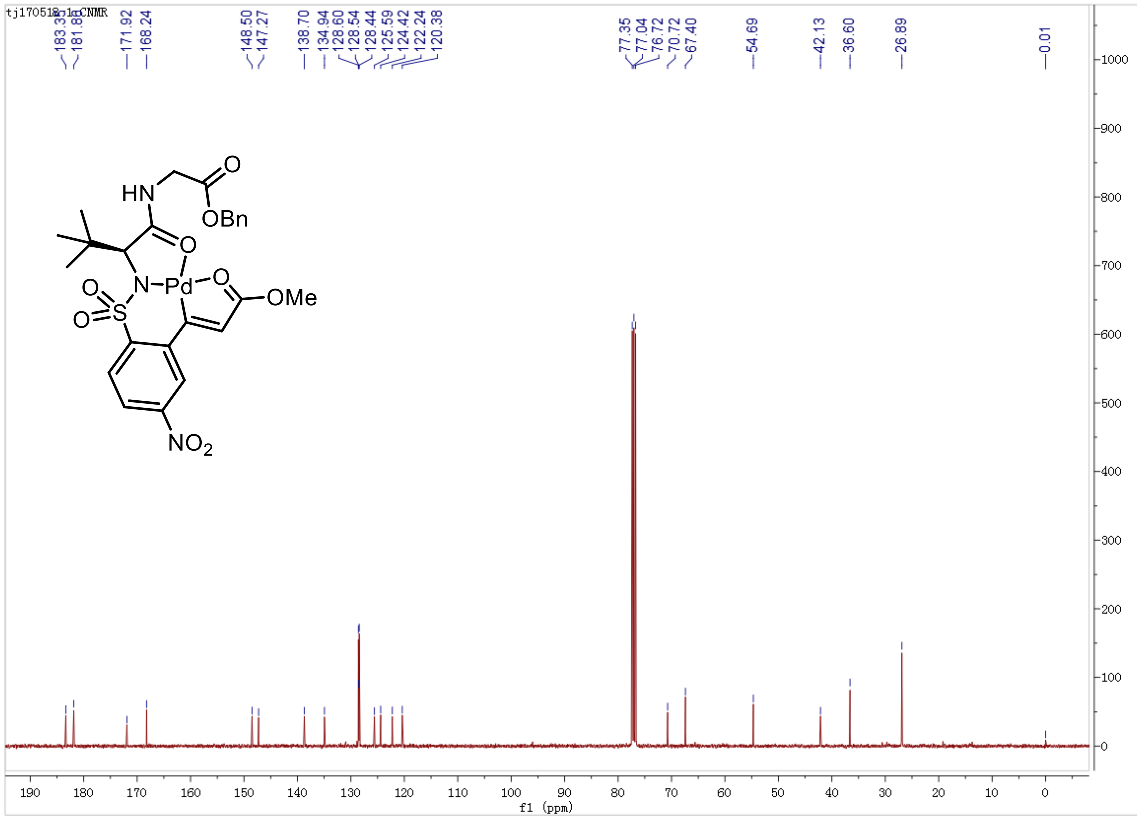

Supplementary Figure 286. ^{13}C NMR (100 MHz, $CDCl_3$) spectrum of compound **7ak**

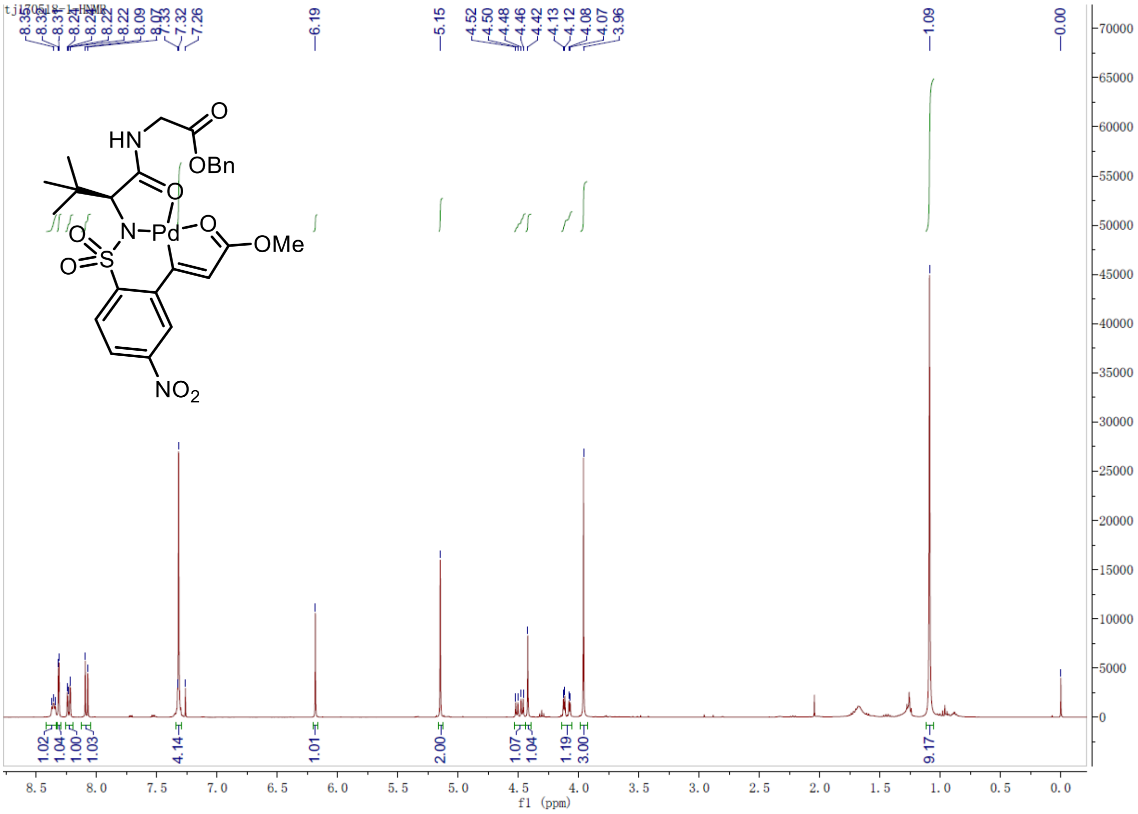

Supplementary Figure 287. ^{1}H NMR (400 MHz, $CDCl_3$) spectrum of compound **7ak**

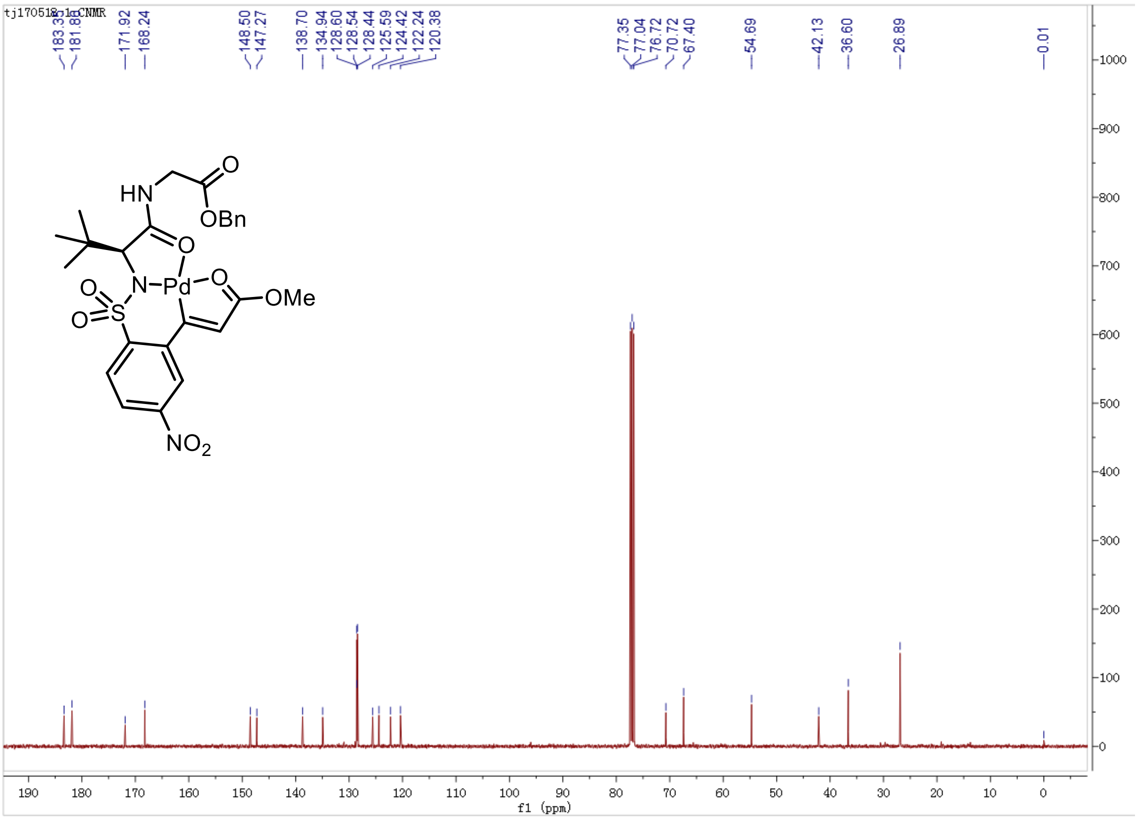

Supplementary Figure 288. ^{13}C NMR (400 MHz, $CDCl_3$) spectrum of compound **7ak**

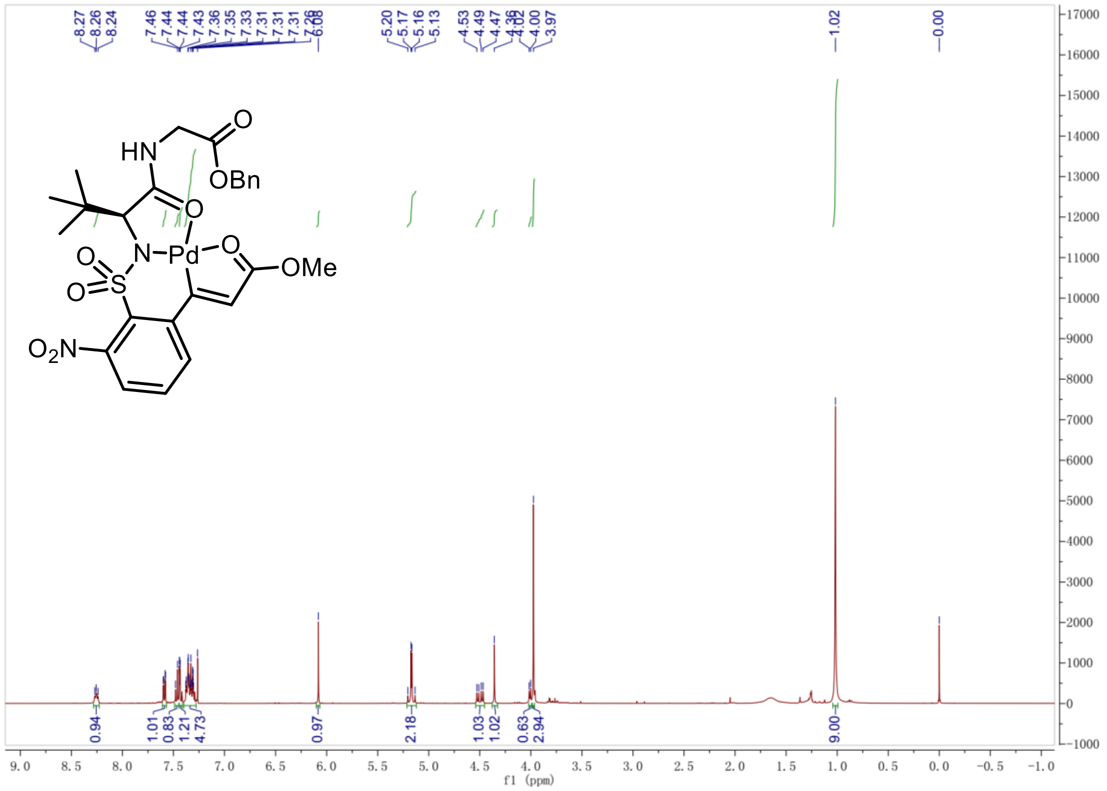

Supplementary Figure 289. ^{1}H NMR (400 MHz, $CDCl_3$) spectrum of compound **7jk**

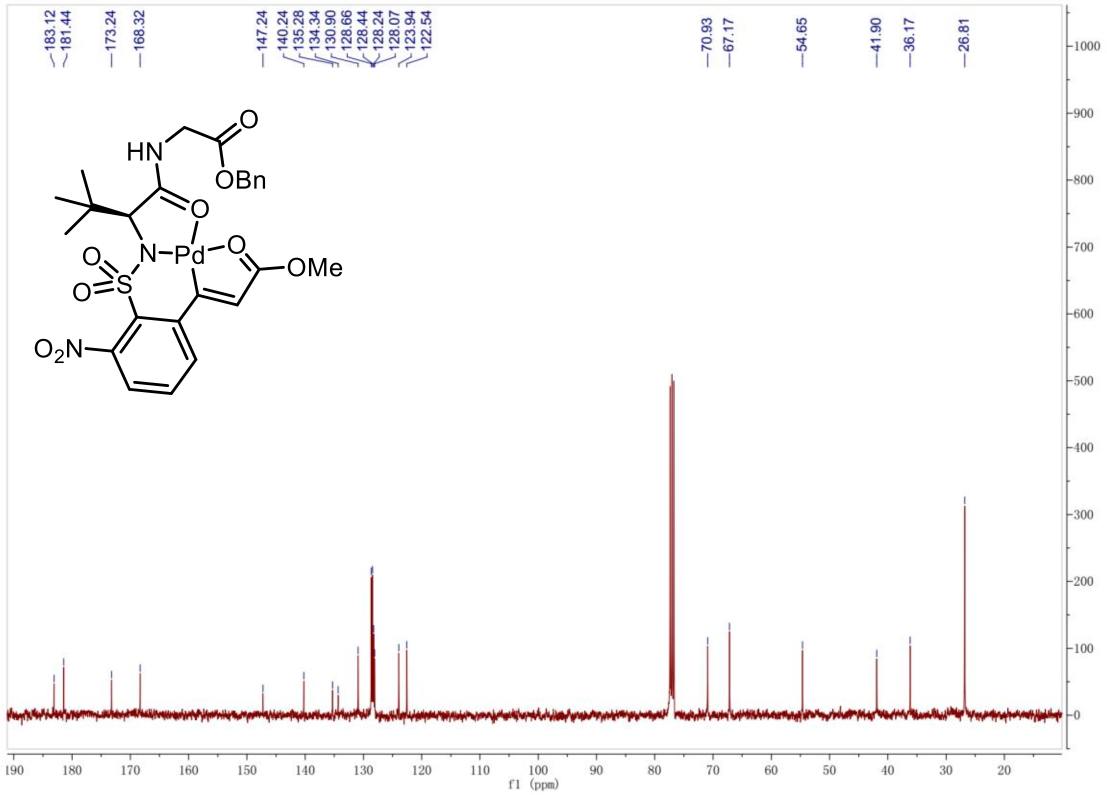

Supplementary Figure 290. ^{13}CNMR (400 MHz, $CDCl_3$) spectrum of compound **7jk**

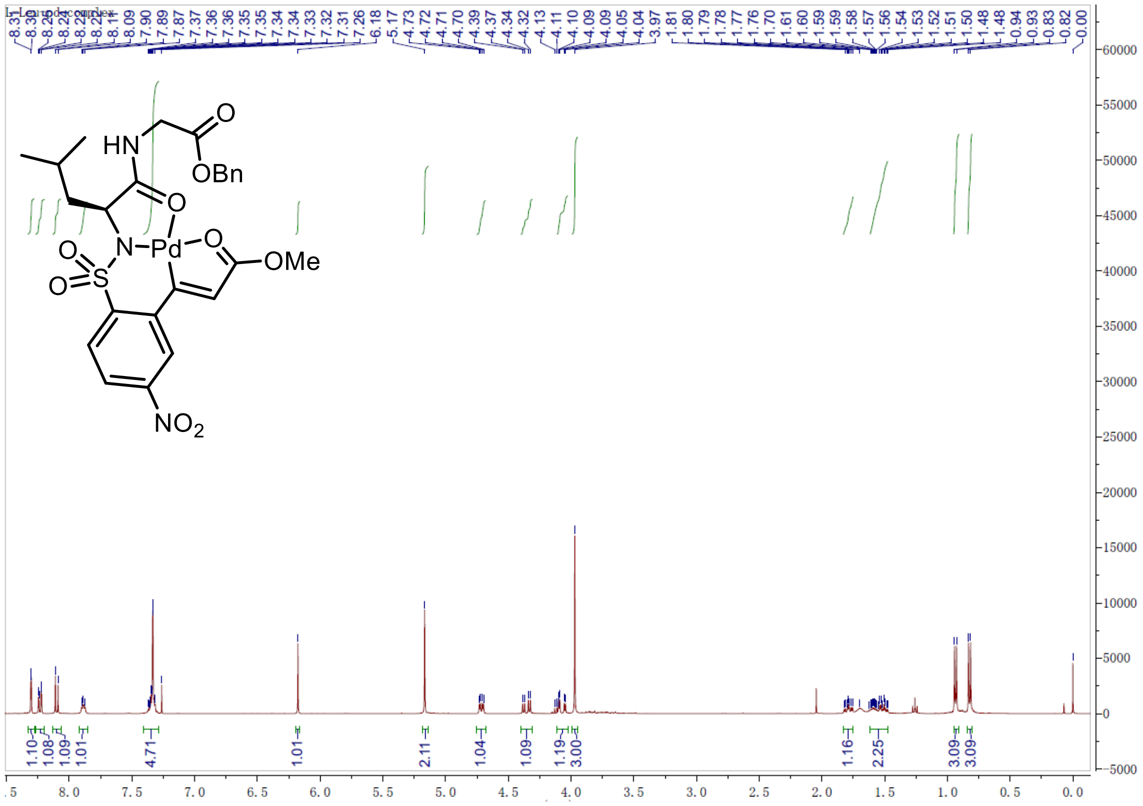

Supplementary Figure 291. ^{1}H NMR (400 MHz, $CDCl_3$) spectrum of compound **7ok**

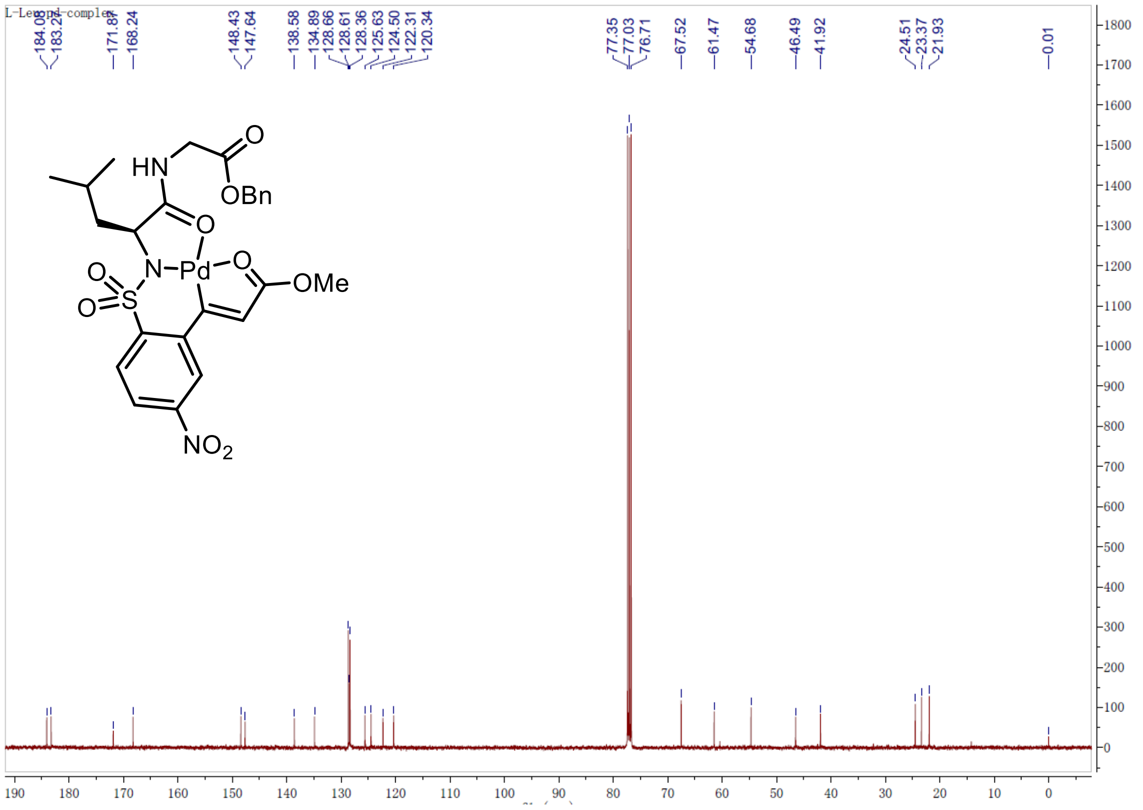

Supplementary Figure 292. ^{13}CNMR (400 MHz, $CDCl_3$) spectrum of compound **7ok**

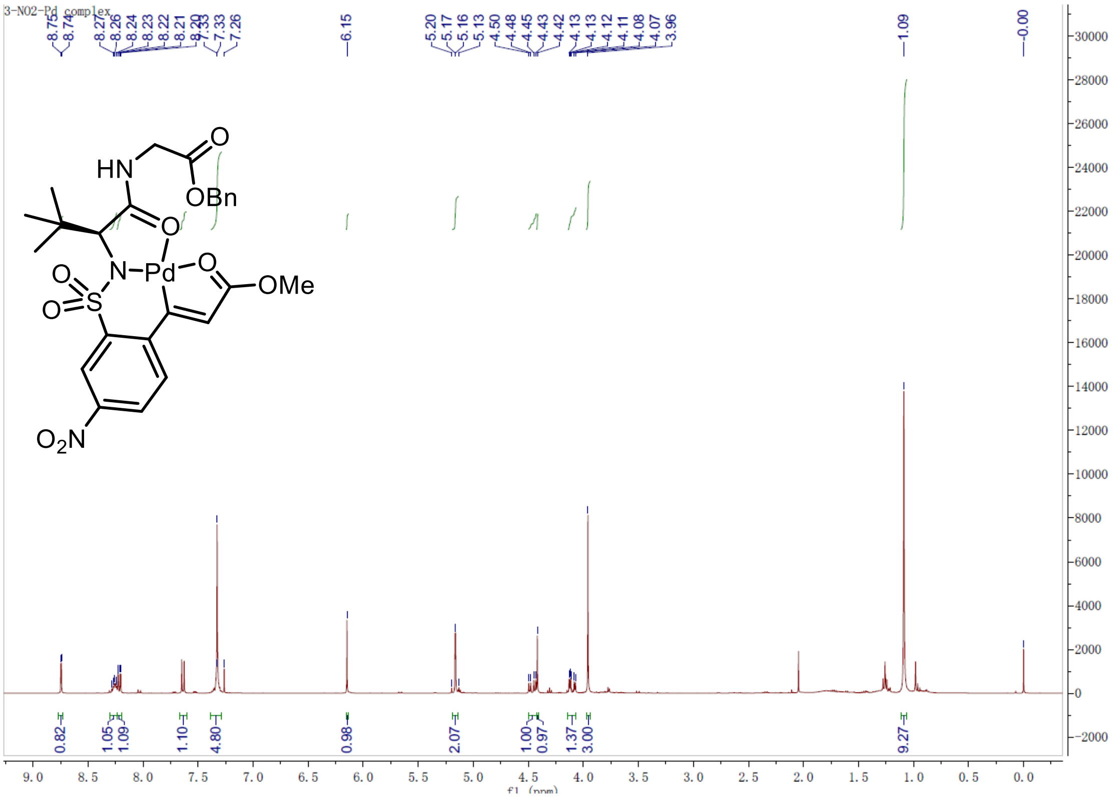

Supplementary Figure 293. ^{1}H NMR (400 MHz, $CDCl_3$) spectrum of compound **7mk**

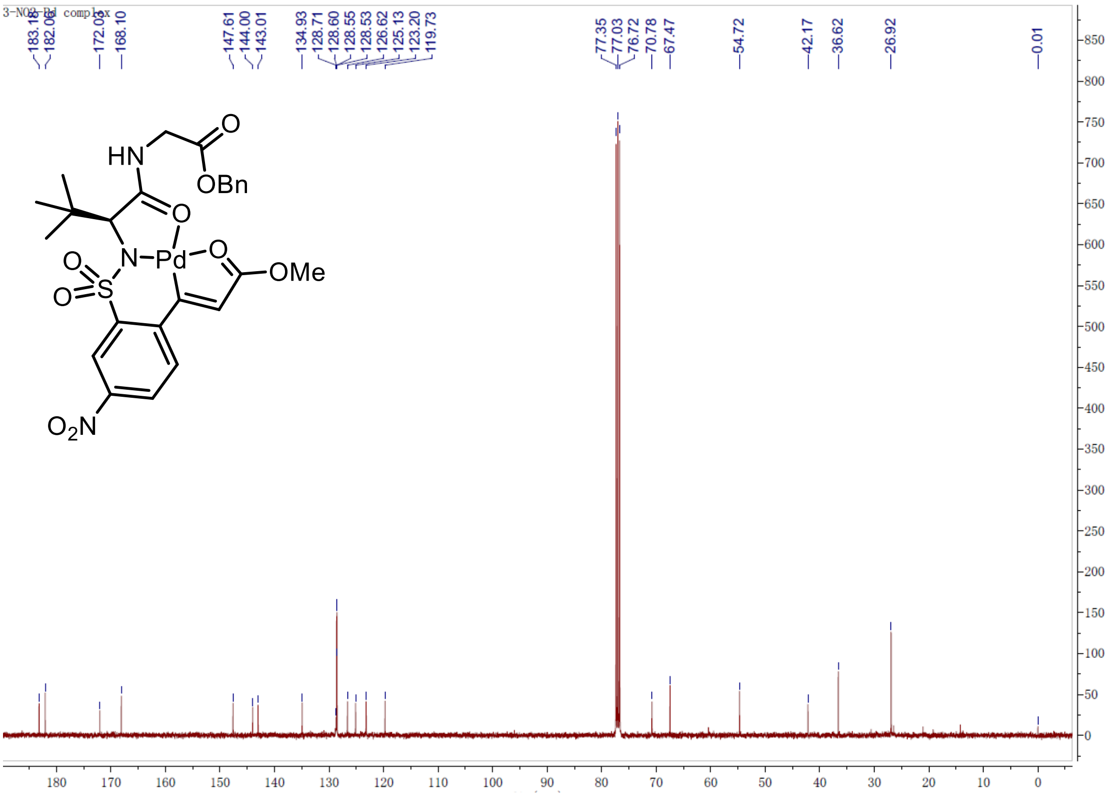

Supplementary Figure 294. ^{13}C NMR (100 MHz, $CDCl_3$) spectrum of compound **7mk**

Compound 9k

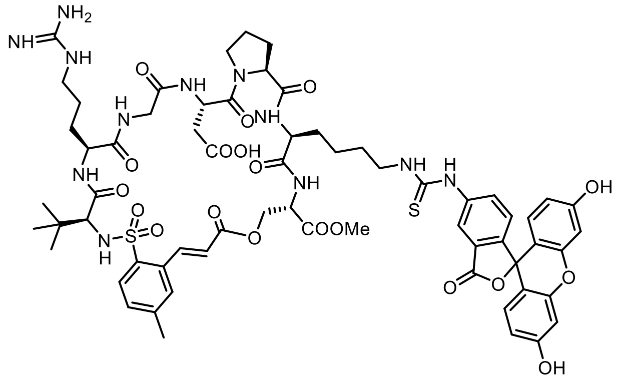

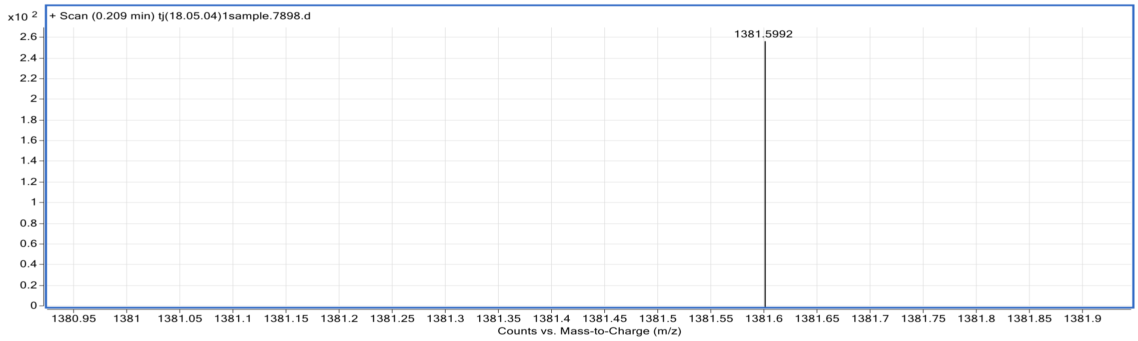

Supplementary Figure 295. HRMS (ESI) $[M+H]^+$ m/z calcd for $C_{64}H_{77}N_{12}O_9S_2$ 1381.5979, found 1381.5992

Crystallographic data of compound 7mk

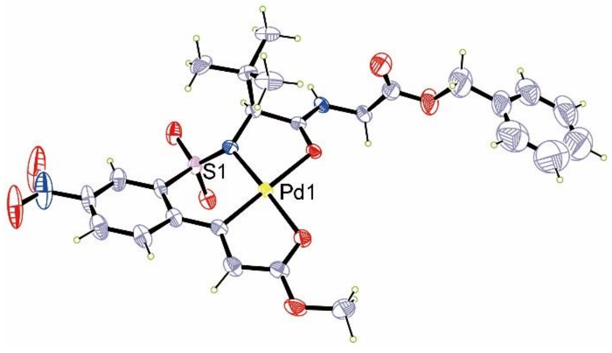

Supplementary Figure 296. X-ray crystallographic analysis of compound **7mk**. Detailed data has been deposited in The Cambridge Crystallographic Data Centre (CCDC) with the accession number of 1816804.
